# Site-Selective Distal Arylation of Sugars Enabled by Cyclic Acetals

**DOI:** 10.1021/jacs.6c08408

**Published:** 2026-06-11

**Authors:** Shuai Zhang, Julia Ordóñez, Christian O. Blanco, Laura Talavera, Niteshlal Kasdekar, Enrique Gómez-Bengoa, David Crich, Ruben Martin

**Affiliations:** † 202569 Institute of Chemical Research of Catalonia (ICIQ), The Barcelona Institute of Science and Technology, Av. Països Catalans 16, 43007 Tarragona, Spain; ¶ Departament de Química Analítica i Química Orgànica, 160665 Universitat Rovira i Virgili, c/Marcel·lí Domingo, 1, 43007 Tarragona, Spain; ‡ Department of Pharmaceutical and Biomedical Sciences, University of Georgia, Athens, Georgia 30602, United States; ¥ Department of Organic Chemistry I, Universidad Pais Vasco, UPV/EHU, Apdo 1072, 20080, San Sebastian, Spain; £ ICREA, Passeig Lluís Companys, 23, 08010 Barcelona, Spain

## Abstract

Herein, we describe a photoinduced Ni-catalyzed protocol that leverages the potential of cyclic acetals as vehicles for accessing glycosides bearing carbon–carbon linkages distal to the anomeric position. This method offers a new gateway to elusive compounds with high site selectivity, providing new opportunities in sugar-based therapeutics with lipophilic side chains.

## Graphical abstract

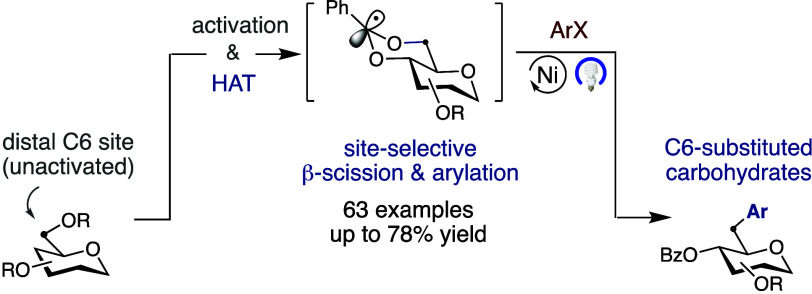

Driven by the pivotal role of carbohydrates in biological processes,^1^ chemists have been challenged to design catalytic routes that modify the core of monosaccharides for the discovery of new sugar-based therapeutics.^2^ Among these, *C*-glycosides have gained momentum in the drug discovery pipeline, with potencies that oftentimes surpasses that of their conventional *O*-glycoside analogues (Scheme 1).^3^ At present, the majority of nonenzymatic routes for accessing *C*-glycosides capitalize on multistep sequences with tailored glycosyl precursors containing anomeric leaving groups or unsaturated glycals (*path a*).^3,4^ Although carbohydrates with C–C linkages distal to the anomeric carbon might offer new opportunities in medicinal chemistry settings, the design of catalytic techniques that allow access to C6-substituted architectures from simple sugars with neat C–O extrusion still remains an unexplored endeavor (*path b*).^5,6^

**Scheme 1:**
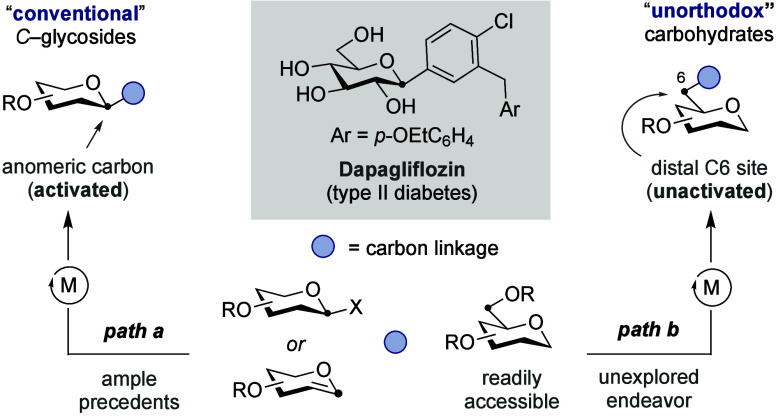
Synthetic Routes toward Abnormal Sugars

Recently, photoredox catalysis and hydrogen-atom transfer (HAT) have offered new tactics for the modification of carbohydrates via *sp* ^*3*^ C–O scission aided by activating groups or *sp* ^*3*^ C–H cleavage occurring either at the anomeric carbon or adjacent to a suitably protected *sp* ^*3*^ C–O linkage (Scheme 2, *left*).^7,8^ Given the presence of multiple *sp* ^*3*^ C–O and C–H sites in a carbohydrate, it was not clear whether it would be possible to override the innate proclivity for bond formation at secondary *sp* ^*3*^ sites while accessing sugars bearing distal C–C architectures at C6 with high modularity, generality, and selectivity.^7^ If successful, this would constitute a worthwhile endeavor with complementary reactivity and selectivity to canonical methods that modify carbohydrates. In addition, it could offer new chemical space using vectors left unexplored by conventional means and new opportunities to access sugars with lipophilic side-chains in inhibitor design.^9^ To this end, we wondered whether we could harness the potential of readily accessible cyclic acetals as one-electron handles via photoinduced HAT (**I**) and a site-selective β-scission (**II**) with the versatility and modularity of Ni catalysts for bond-formation (Scheme 2, *right*).^10,11^ Herein, we report the successful realization of this goal, culminating in a broadly applicable protocol that is distinguished by its mild conditions, selectivity, and generality, even with challenging combinations.

**Scheme 2:**
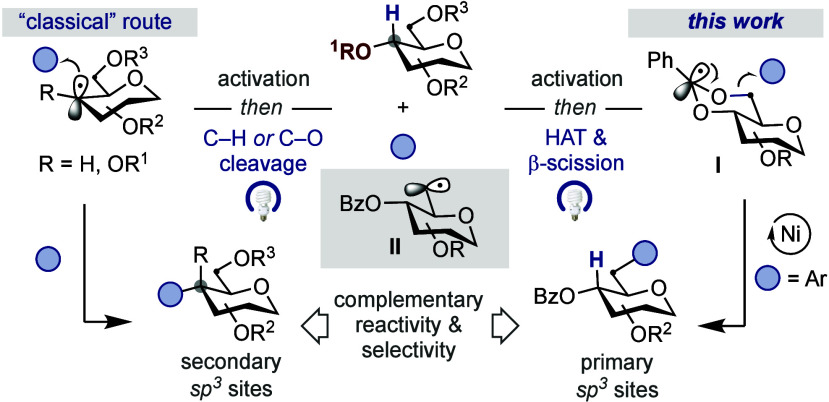
C–C Bond-Formation Distal to Anomeric Site

Roberts and Crich previously described methods to enable radical fragmentation of cyclic acetals,^12^ offering an alternative to the venerable Hanessian–Hullar reaction that operates via ionic fragmentation.^13^ However, the ability to merge the propensity of acetals for radical fragmentation with a subsequent C–C bond formation still remained an unexplored scenario, yet a worthwhile endeavor, in the carbohydrate arena. Consequently, we started our investigations by evaluating the viability for enabling a C6-arylation from **1** and **2** via DFT (Scheme 3).^14^ As expected,^12^ open-shell species arising from HAT at the acetalic *sp* ^*3*^ C–H site in both **1** and **2** exhibited similar fragmentation activation energies and comparable stabilities. However, C6-arylation was only observed when utilizing **2** as a substrate, whereas significant amounts of **1ab** and **1ac** were detected from **1**, the formation of which likely results from oxidation of **III** prior to addition of water. These results suggested that the destabilization of **III-cat** or **IV-cat** might be critical for success. Indeed, the computed relative energies revealed that the latter was 3.6 kcal/mol less stable than its former carbon analogue, a destabilization that is ascribed to electronic effects due to the presence of a more electronegative oxygen atom in the cyclohexane ring.^15^ Therefore, the reduced cation stability of **IV-cat** and the slightly lower propensity of **III** for fragmentation (ΔΔG^‡^ = +21.2 kcal/mol) when compared to **IV** (ΔΔG^‡^ = +20.1 kcal/mol) might favor productive fragmentation of the latter prior to interception with an aryl halide in the context of a Ni-catalyzed endeavor. Additional DFT calculations revealed that the observed selectivity might also arise from an interplay between radical stability and C–O bond strength, thus offering a rationale that goes beyond reported reactivity patterns.^16^

**Scheme 3:**
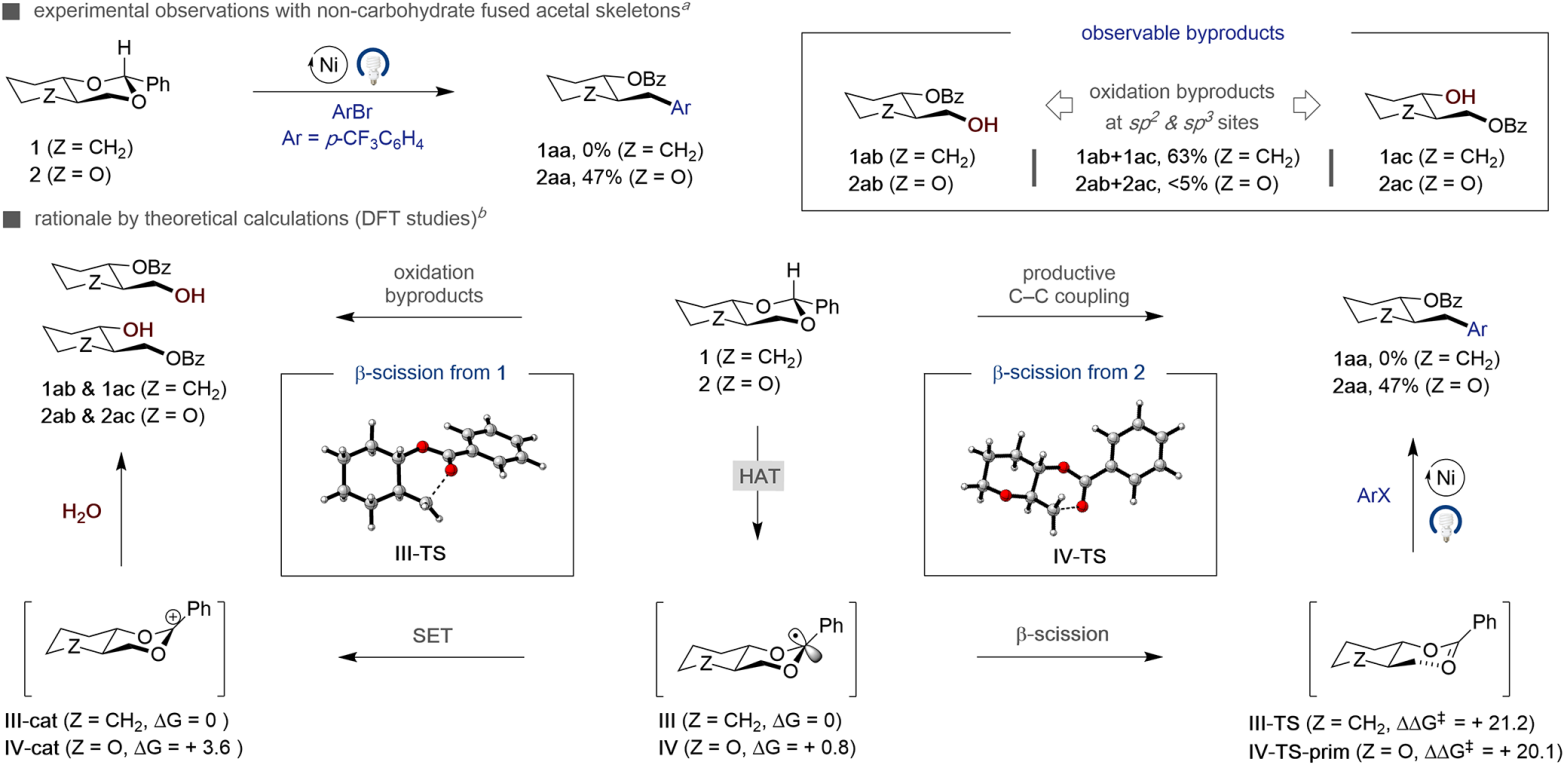
Initial Observations and Theoretical Calculations^a^

a See the Supporting Information for details.
b UM06-2x/6-311++G­(d,p)-IEFPCM­(2-propanol) Free energies are given in kcal/mol.

Encouraged by these findings, we evaluated the reaction of **3a**—readily accessible from a commercially available precursor—with **4a** (Table 1). After some experimentation,^16^ a protocol consisting of NiBr_2_·dme (5 mol %), Ir­[dF­(CF_3_)­ppy]_2_dtbbpyPF_6_ (**PC1**, 0.2 mol %), **L1** (6 mol %), NH_4_Cl (10 mol %), and Na_2_CO_3_ (2.0 equiv) in PhH/*t*-AmOH (1:1) under 456 nm delivered **5a** in 74% isolated yield (entry 1). Under the limits of detection, we did not observe byproducts arising from oxidation of **I** followed by nucleophilic attack of water. As shown in entries 2–4, a 1:1 mixture of PhH/*t*-AmOH showed superior results. Notably, substituents at C2 and C9 in the 1,10-phenanthroline backbone had a deleterious impact on reactivity (entry 5), whereas **L3** or **L4** possessing fragments at C4 or C7 resulted in lower yields of **5a** and side-reactions arising from oxidation of **I**. Moreover, traces of **5a** were found when extending the denticity of the ligand (**L5**, entry 8). In addition, Ni precatalysts or bases other than NiBr_2_·dme or Na_2_CO_3_ resulted in lower reactivity (entries 9 and 10), and control experiments revealed that all of parameters were critical for the reaction to occur (entry 11).

**Table 1:**
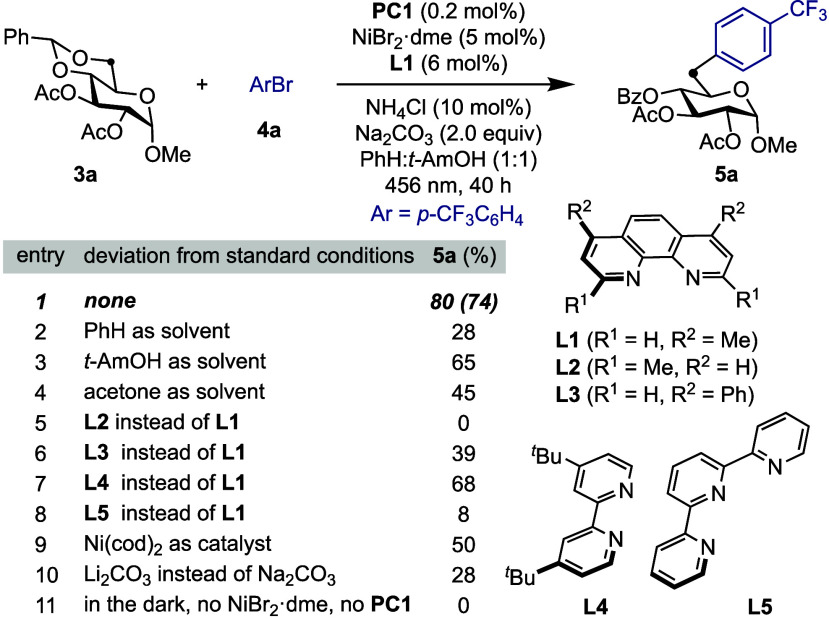
Optimization of the Reaction Conditions^a^

a Conditions: **3a** (0.24 mmol), **4a** (0.2 mmol), Ir­[dF­(CF_3_)­ppy]_2_dtbbpyPF_6_ (0.2 mol %), NiBr_2_·dme (5 mol %), **L1** (6 mol %), NH_4_Cl (10 mol %), Na_2_CO_3_ (0.4 mmol) in PhH/*t*-AmOH (1:1, 2.0 mL) at rt for 40 h under 456 nm. ^1^H NMR yields using 1,1,2,2-tetrachloroethane as standard; isolated yield in parentheses.

With optimized conditions in hand, we turned our attention to studying the generality of the protocol (Table 2). As shown, the method was found to be widely applicable across a variety of substituted saccharides, including substrates bearing free alcohols (**5d, 5k, 5l, 5m, 5z**). Notably, the protocol was not limited to sugar derivatives possessing heteroatoms at the anomeric carbon, as **5o**, **5p**, **5r**, **5s** and **5u** could all be within reach. The ability to access **5w**–**5z** or **5o** illustrates the complementarity of our protocol with known protocols aimed at preparing *C*-glycosides at the anomeric carbon,^3,4^ offering a valuable entry point to obtain sugar architectures with multiple carbon linkages at C1 and C6. Equally interesting was the observation that similar yields were observed with α-d-mannopyranoses (**5j, 5k**), thus showing that the saccharide configuration does not have a significant impact on reactivity. Moreover, amino groups (**5t**) or even epoxides (**5u**, **5v**) posed no problems, obtaining compounds with high synthetic versatility. Importantly, the protocol could be applied to advanced intermediates such as Empagliflozin (**5w, 5z**), Dapagliflozin (**5x**), Ipragliflozin (**5y**), Salidroside (**5za**), Phlorizin (**5zb**), Salicin (**5zc**), or Maltoside (**5zd**) derivatives, showcasing the impact that the method might have in the context of in late-stage functionalization.^17^ In particular, selective functionalization of a single primary hydroxyl group at the nonreducing terminus of a 1*→*4 linked di- or higher saccharide as in **5zd** highlights a unique advantage of the acetal fragmentation method.

**Table 2:**
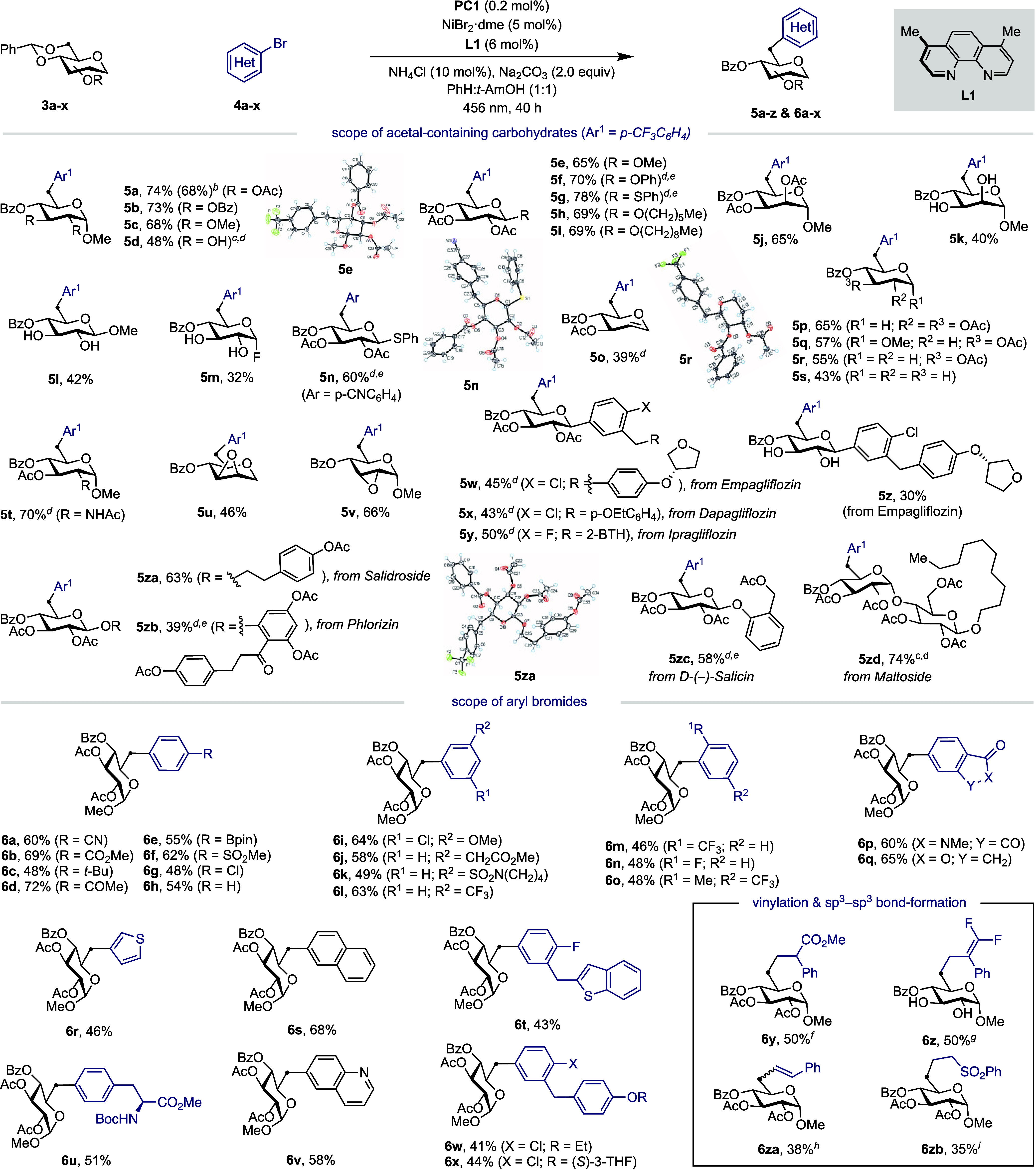
Cyclic Acetals as Enabling Vehicles for Forging C6-Arylated Sugars via Dual Catalysis^a^

a As shown in Table 1 (entry 1). Isolated yields, average of two independent runs.

b 0.50 mmol scale.

c **4a** (1.2 equiv).

d 0.05 M.

e **4a** (2.0 equiv).

f Using methyl 2-phenyl acrylate (0.20 mmol); 2:1 dr.

g Using 3,3,3-trifluoroprop-1-en-2-yl)­benzene (0.20 mmol).

h Using β-bromostyrene (0.20 mmol); 1.6:1 *E/Z*.

i (Vinylsulfonyl)­benzene (0.20 mmol).

As shown in Table 2 (*bottom*), the method could also be applied to a wide range of aryl and heteroaryl bromides regardless of whether electron-donating or electron-withdrawing groups were present or not. In addition, a wide range of functional groups could be easily accommodated, including nitriles (**6a**), esters (**6b**, **6j**), ketones (**6d**), sulfones (**6f**), sulfonamides (**6k**), carbamates (**6u**), and lactones (**6q**). Additionally, boronic esters (**6e**) and halides (**6g**, **6i**, **6t**, **6w**, **6x**) could be tolerated, leaving ample room for further functionalization via conventional metal-catalyzed cross-coupling reactions.^18^ Moreover, aryl halides possessing weak benzylic *sp* ^*3*^ C–H bonds that are a priori susceptible for competing HAT processes posed no problems, and the targeted products **6i**, **6w**, and **6x** could all be obtained in good yields. As shown for **6m**–**6o**, the method could also be implemented with *ortho*-substituted aryl bromides or even heteroaryl bromides (**6p–6r**, **6t** and **6v**), albeit in slightly lower yields.

On the basis of these results, we next evaluated whether our protocol could be extended to carbohydrate mimics that result from the substitution of the endocyclic oxygen of conventional saccharides by a sulfur or a nitrogen atom. As shown in Scheme 4 (*top left*), this turned out to be the case. Specifically, the method could be applied to both 1-deoxynojirimycin and thioglucose derivatives, resulting in C6-substituted derivatives **7a** and **7b**, thereby providing direct access to medicinally relevant thiosulfates and iminosugars.^9,19^ In addition, even simple, nonfused cyclic acetals such as the rhamnoside **3aa** possessing a pending acetate group could be employed as substrate en route to **8a** in 40% yield. It is worth noting that the utilization of 2-phenyl-1,3-dioxane itself as substrate, lacking the C–O bond at C5, resulted in negligible conversion to products, thus reinforcing the rationale depicted in Scheme 3.^16^

**Scheme 4:**
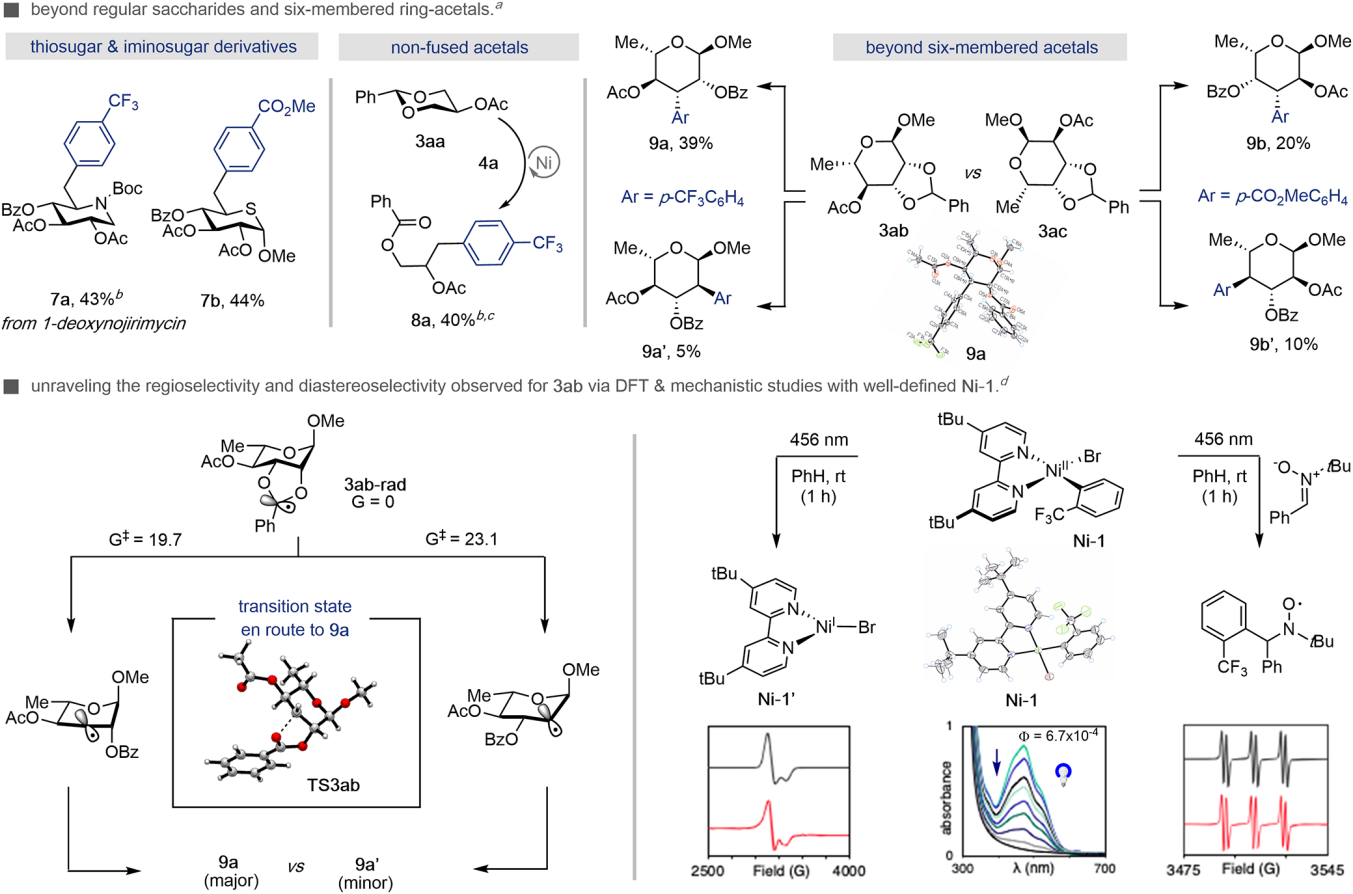
Beyond Regular Saccharides and Rationale of Regioselectivity and Diastereoselectivity via DFT Studies^a^

a As shown in Table 1 (entry 1). Yield of isolated compounds; average of at least two independent runs.
b 0.05 M.
c
**3aa** (3.0 equiv).
d UM06-2X/6-311++G­(d,p)-IEFPCM­(2-Propanol). Free energies are given in kcal/mol.

Driven by the generality and applicability of our protocol, we wondered whether we could promote C–C bond formation using five-membered ring-acetals at other ring positions than C6 and the anomeric carbon. A priori, one might expect little discrimination in the β-scission of **3ab**, leading to mixtures of regioisomers (Scheme 4, *top right*).^12a,20^ However, exposure of **3ab** to **4a** under our optimized reaction conditions resulted in **9a**:**9a**′ in an 8:1 ratio, with C–C bond formation occurring predominantly at C3 and with equatorial selectivity. A similar scenario was found for fucoside **3ac**, resulting in the formation of two regioisomers in lower selectivity than that shown for **3ab**. Although in lower yields, the ability to couple **3ab** and **3ac** can hardly be underestimated, indicating that our protocol could be utilized for accessing *C*-glycosides “*a la carte*” depending on where the acetal is located within the saccharide backbone. Intrigued by the origin of regioselectivity and diastereoselectivity en route to **9a**, we turned our attention to DFT calculations for guidance (Scheme 4, *bottom left*). Although the transition state for the fragmentation of 2-phenyl-1,3-dioxolane (**3ac**) could not be located, β-scission of **3ab** via **TS3ab** was particularly favorable (19.7 kcal/mol), with even lower energies than those found for six-membered ring analogues (Scheme 3).^14^ These findings suggest that the ring strain in **3ab** arising from the fused five-membered ring acetal and the pyranose ring facilitates ring-opening. In line with our experimental observations, fragmentation revealed a preference for C3 over C2 fragmentation (19.7 vs 23.1 kcal/mol). In addition, the most stable conformation of the resulting open-shell intermediate arising from β-scission positions both the benzoyl and methoxy group in axial position.

To probe the mechanistic intricacies of the reaction, we turned our attention to studying the photochemical behavior of well-defined oxidative addition species. Although the synthesis of the latter bearing **L1** was shown to be particularly problematic, the preparation of **Ni-1** containing catalytically active **L4** (Table 1) was easily accomplished by exposure of Ni­(cod)**L4** to **4m** in THF at rt, the structure of which was characterized by X-ray diffraction (Scheme 4, *bottom right*).^16^ Irradiation of **Ni-1** at 456 nm resulted in the clean formation of **Ni-1′**, as judged by EPR spectroscopy. While EPR spin-trapping experiment with phenyl *N-tert*-butylnitrone (PBN) confirmed the release of aryl radicals, this pathway represents a minor, unproductive process given the low quantum yields observed for such a process (6.7 × 10^–4^).^21^ Indeed, Stern–Volmer quenching studies showed that the excited state of **PC1** is most efficiently quenched by exogeneous bromide ions, and the resulting bromine radicals are likely responsible for promoting HAT at the acetal moiety.^16,22^ Moreover, the intermediacy of open-shell species **II** arising from β-scission of **I** (Scheme 2) is supported by conducting the reaction of **3a** with **4m** in the presence of PBN, when a characteristic pattern for alkyl-substituted nitroxide radicals was observed by EPR spectroscopy.^16^

In summary, we have developed a site-selective photoinduced strategy that streamlines the access to complex saccharides bearing carbon–carbon linkages at positions other than the anomeric carbon. This technology leverages the potential of cyclic acetals as enabling vehicles to forge the targeted C–C linkage via the intermediacy of open-shell species, and it is characterized by its mild conditions and broad applicability, with regioselectivity and diastereoselectivity being dictated depending on the location of the acetal motif. This method provides a new, direct gateway to structurally complex saccharides with high chemoselectivity and stereochemical control and, in doing so, opens up new chemical space for exploration in the design of sugar-based therapeutics.

## Supplementary Material



## Notes

Experimental procedures and spectral and crystallographic data (PDF)
