# Supplementary material for: Site-Selective Distal Arylation of Sugars Enabled by Cyclic Acetals
Source: J Am Chem Soc. 2026 Jun 11;148(24):24609–16. doi: 10.1021/jacs.6c08408 (PMC13307320; doi:10.1021/jacs.6c08408)
Supplement: Supplementary file 1 [file ja6c08408_si_001.pdf]

Supporting Information

Site-Selective Distal Arylation of Sugars Enabled by Cyclic Acetals

Shuai Zhang,[†¶] Julia Ordóñez,[†¶] Christian O. Blanco,[†] Laura Talavera,[†¶] Niteshlal Kasdekar,[‡] Enrique Gómez-Bengoa,[*¥] David Crich,[*‡] and Ruben Martin[*†£]

[†] Institute of Chemical Research of Catalonia (ICIQ), The Barcelona Institute of Science and Technology, Av. Països Catalans 16, 43007 Tarragona, Spain

[¶] Departament de Química Analítica i Química Orgànica, Universitat Rovira i Virgili, c/Marcel·lí Domingo, 1, 43007 Tarragona, Spain

[‡] Department of Pharmaceutical and Biomedical Sciences, University of Georgia, Athens, Georgia 30602, United States

[¥] Department of Organic Chemistry I, Universidad Pais Vasco, UPV/EHU, Apdo 1072, 20080, San Sebastian, Spain

[£] ICREA, Passeig Lluís Companys, 23, 08010 Barcelona, Spain

rmartinromo@iciq.es

***General Considerations* 1**

***Photochemical Reaction Setup* 2**

***Optimization of the Reaction Conditions* 3**

***Synthesis of Starting Materials* 5**

***C6-substituted Glycosides via dual Ni/Photoredox Catalysis* 15**

***Mechanistic Experiments* 56**

***X-Ray Crystallographic Data* 63**

***Computational Studies* 73**

***References* 99**

***NMR Spectra* 101**

General Considerations

Reagents: Reactions were performed in 12 mL reaction vials. All reactions were carried out under Nitrogen/Argon atmosphere unless otherwise stated. Commercially available materials were used as received without further purification. Anhydrous benzene (99.8% purity) and *tert*-amyl alcohol (99% purity) were purchased from Sigma Aldrich. {Ir[dF(CF_3)ppy]$_2$(dtbbpy)}PF_6 was synthesize according to a literature procedure.[1] Ammonium chloride (NH_4Cl) and sodium carbonate (Na_2CO_3) were dried under vacuum at 70 ºC and stored in the glovebox.

Analytical methods: ^{1}H NMR and ^{13}C NMR spectra are included for all compounds. ^{1}H and ^{13}C NMR spectra were recorded on Bruker 300 MHz, Bruker 400 MHz and 500 MHz at 20 ºC. All ^{1}H NMR spectra are reported in parts per million (ppm) downfield of TMS and were measured relative to the signals for $CHCl_3$ (7.26 ppm) unless otherwise indicated. All ^{13}C NMR spectra are reported in ppm relative to TMS, were calibrated using the signal of residual $CHCl_3$ (77.16 ppm), ^{19}F NMR was obtained with ^{1}H decoupling unless otherwise indicated. Coupling constants, *J*, are reported in Hertz. Flash chromatography was performed with Sigma-Aldrich silica gel, pore size 60 Å (230-400 mesh). Thin layer chromatography was used to monitor reaction progress and analyze fractions from column chromatography. To this purpose TLC Silica gel 60 F254 Aluminium sheets from Sigma-Aldrich were used and visualization was achieved using UV irradiation and/or staining with a potassium permanganate solution. The yields reported refer to isolated yields and represent an average of at least two independent runs. High-resolution mass spectra (HRMS) were recorded on a Waters LCT Premier spectrometer or in a MicroTOF Focus, Bruker Daltonics spectrometer using electrospray ionization (ESI). Infra-red (IR) spectra measurements were carried out on a Bruker Optics FT-IR Alpha spectrometer equipped with a DTGS detector, KBr beam splitter at 4 cm^{-1} resolution using a one bounce ATR accessory with diamond windows. Melting points were measured using open glass capillaries in a Büchi B540 apparatus, reported uncorrected.

Photochemical Reaction Setup

UFO photoreactor

A custom-made photoreactor made with a 3D-printer[2] was equipped with a 456 nm Kessil lamp (PR160L-456 nm, 40 W) used on full intensity. A fan was installed below the set-up to cool down the reaction through a small slit at the bottom of the photoreactor. The inner part of the set-up was covered with UV-shielding paper. The system allows 8 reaction tubes to be placed simultaneously. The system was placed on top of a magnetic stirrer (~650 rpm) with cooling from an external fan placed in front of the system at 10 cm.

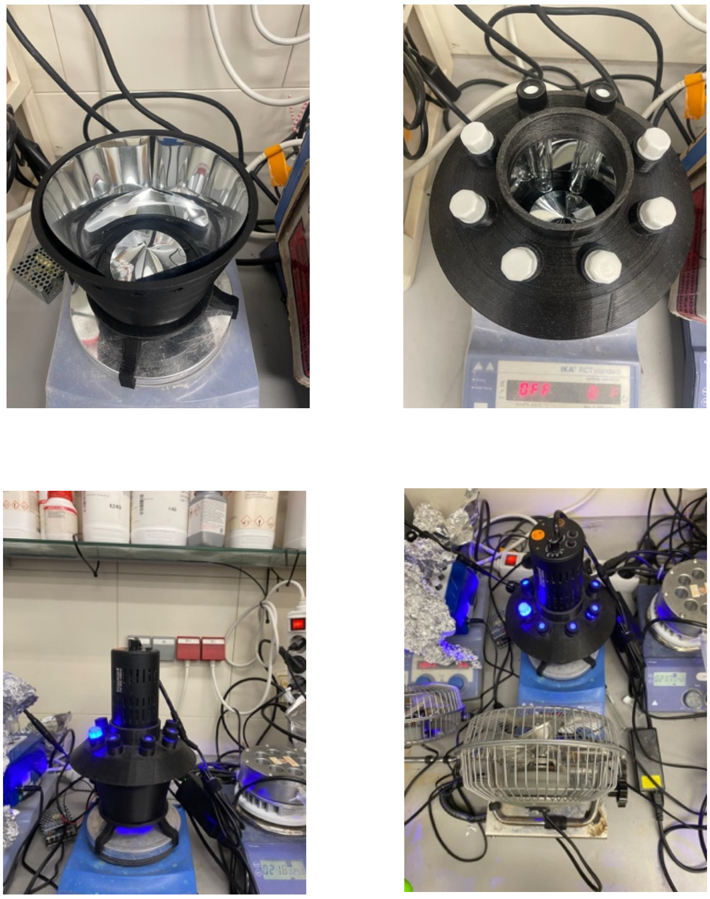

Figure S1. UFO photochemical set-up

Optimization of the Reaction Conditions

General procedure A: To an oven dried 12 mL reaction vial equipped with a Teflon-coated stirring bar, the corresponding photocatalyst (0.4 μmol, 0.2 mol%) was added as a 2 mg/mL solution in dichloromethane. The solvent was evaporated under vacuum for 30 min and the corresponding ligand (0.12 mmol, 6 mol%) and **3a** (87.9 mg, 0.24 mmol, 1.2 equivalents) were added sequentially. Subsequently, the vial was brought inside a nitrogen-filled glovebox and the corresponding nickel precatalyst (0.01 mmol, 5 mol%), HAT reagent (0.02 mmol, 10 mol%) and base (0.4 mmol, 2 equivalents) were added. Then, the vial was closed and taken outside the glovebox where solvent (0.1 M) and 1-bromo-4-(trifluoromethyl)benzene (**4a**, 28 μL, 0.2 mmol, 1 equivalent) were added sequentially via syringe. The reaction was stirred at 650 rpm under 456 nm light irradiation for 40 h. After the reaction was completed, the crude was filtered through a short celite pad eluting with ethyl acetate and concentrated under vacuum. The crude was analyzed by ^{1}H NMR using 1,3,5-trimethoxybenzene as internal standard.

Table S1. Optimization of the Reaction Conditions.

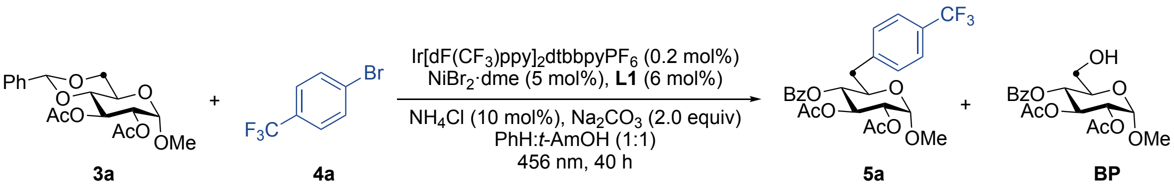

entry	deviation from standard conditions	**5a** (%)[a]	**BP** (%)[a]
1	none	80 (74)[b]	4
2	NH_4Br instead of NH_4Cl	77	4
3	NaCl instead of NH_4Cl	73	6
4	without Ir[dF(CF_3)ppy]$_2$dtbbpyPF$_6$ or in the darkness	0	0
5	PhH as solvent instead of PhH:*t*-AmOH	28	7
6	*t*-AmOH as solvent instead of PhH:*t*-AmOH	65	3
7	Acetone as solvent instead of PhH:*t*-AmOH	45	4
8	Toluene as solvent instead of PhH:*t*-AmOH	trace	3
9	*t*-AmOH/Acetone as solvent instead of PhH:*t*-AmOH	51	6
10	Benzene/Acetone as solvent instead of PhH:*t*-AmOH	61	5
11	**L2** as ligand instead of **L1**	0	18
12	**L3** as ligand instead of **L1**	39	4
13	**L4** as ligand instead of **L1**	68	6
14	**L5** as ligand instead of **L1**	8	8
15	without ligand	trace	2
16	Ni(acac)$_2$ as catalyst	44	6
17	Ni(cod)$_2$ as catalyst	50	7
18	without Ni	0	5
19	K_2CO_3 as base instead of Na_2CO_3	62	7
20	Li_2CO_3 as base instead of Na_2CO_3	28	17
21	Mg(OEt)$_2$ as base instead of Na_2CO_3	23	0
22	without base	8	15

Conditions: **3a** (0.24 mmol), **4a** (0.2 mmol), Ir[dF(CF_3)ppy]$_2$dtbbpyPF$_6$ (0.2 mol%), $NiBr_2$·dme (5 mol%), L_1 (6 mol%), NH_4Cl (10 mol%), Na_2CO_3 (0.4 mmol) in C_6H_6/*t*-AmOH (1:1, 2.0 mL) at room temperature for 40 h under 456 nm Blue-LED irradiation. [a] ^{1}H NMR yields using 1,3,5-trimethoxybenzene or 1,1,2,2-tetrachloroethane as internal standard. [b] Isolated yield.

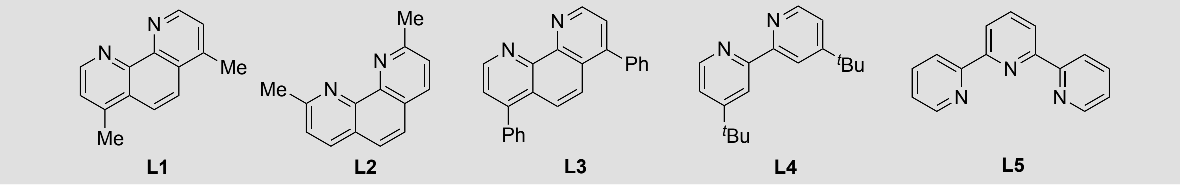

Synthesis of Starting Materials

Synthesis of saccharides

3f, **3h**, **3i**, **3p**, **3q**, **3r**, **3w**, **3x**, **3y**, **3za**, **3zb**, **3zc** and **3zd** were prepared according to the below Procedure B or Procedure C. **2**[3], **3a**[4], **3e**[5], **3g**[6], **3j**[7], **3l**[8], **3t**[9], **3ad**[10] and **3ae**[11] were prepared according to known literature procedure and all spectroscopic data agreed with previously reported data.

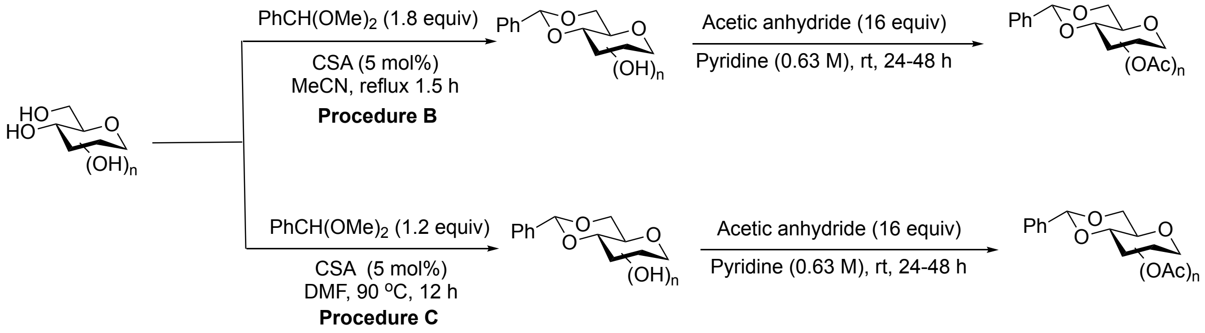

Figure S2. General procedure of saccharides synthesis.

General procedure B for the synthesis of saccharides. To a 100 mL round bottom flask it was added the corresponding carbohydrate (5.0 mmol, 1.0 equivalent) and acetonitrile (20 mL). After the mixture was stirred vigorously at room temperature for 10 minutes, benzaldehyde dimethyl acetal (1.35 mL, 9.0 mmol, 1.8 equivalents) and 10-camphorsulfonic acid (CSA) (58 mg, 0.25 mmol, 5 mol%) were added. After the mixture was stirred for 1.5 h at reflux, it was allowed to reach room temperature. The crude was neutralized by addition of a few drops of NEt_3 (0.05 mL), and was diluted with EtOAc (100 mL). Then, the organic layers were washed with water (3 x 30 mL), and the organic layers were separated and dried over anhydrous Na_2SO_4. The resulting mixture was filtered through a Pad of Celite and used it directly into the next step without further purification. To a solution of the above mixture in pyridine (8 mL, 0.63 M), acetic anhydride (7.6 mL, 80 mmol, 16 equivalents) was added dropwise at rt and the reaction mixture was stirred for 24 h. Afterwards, the reaction was diluted with EtOAc and quenched with HCl (1 M) aqueous solution and washed with HCl (1 M), saturated aqueous $NaHCO_3$ solution and brine. Then, the crude was dried over anhydrous Na_2SO_4, filtered, concentrated under vacuum and purified by crystallization (EtOAc/hexane) to afford the corresponding saccharides.

General procedure C for the synthesis of saccharides. To a 100 mL round bottom flask it was added the corresponding carbohydrate (5.0 mmol, 1.0 equivalent) and DMF (20 mL). After the mixture was stirred vigorously at room temperature for 10 minutes, benzaldehyde dimethyl acetal (0.9 mL, 6.0 mmol, 1.8 equiv) and 10-camphorsulfonic acid (CSA) (58 mg, 0.25 mmol, 5 mol%) were added. After the mixture was stirred for 1.5 h at 90 ºC, it was allowed to reach room temperature. The crude was neutralized by addition of a few drops of NEt_3 (0.05 mL),

and was diluted with EtOAc (100 mL). Then, the organic layers were washed with water (3 x 30 mL), and the organic layers were separated and dried over anhydrous Na_2SO_4. The resulting mixture was filtered through a Pad of Celite and used it directly into the next step without further purification. To a solution of the above mixture in pyridine (8 mL, 0.63 M), acetic anhydride (7.6 mL, 80 mmol, 16 equivalents) was added dropwise at rt and the reaction mixture was stirred for 24 h. Afterwards, the reaction was diluted with EtOAc and quenched with HCl (1 M) aqueous solution and washed with HCl (1 M), saturated aqueous $NaHCO_3$ solution and brine. Then, the crude was dried over anhydrous Na_2SO_4, filtered, concentrated under vacuum and purified by crystallization (EtOAc/hexane) to afford the corresponding saccharides.

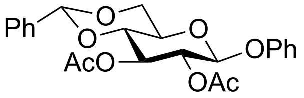

Phenyl 2,3-di-*O*-acetyl-4,6-*O*-benzylidene-β-D-glucopyranoside **(3f):** Following general procedure B, using (2*R*,3*S*,4*S*,5*R*,6*S*)-2-(hydroxymethyl)-6-phenoxytetrahydro-2*H*-pyran-3,4,5-triol (1.28 g, 5.0 mmol, 1.0 equivalent), the crude was purified by crystallization (EtOAc/hexane) to afford **3f** as a white solid (1.66 g, 78% yield). **^{1}H NMR (500 MHz, $CDCl_3$)** δ 7.48 – 7.42 (m, 2H), 7.41 – 7.35 (m, 3H), 7.35 – 7.28 (m, 2H), 7.12 – 7.05 (m, 1H), 7.04 – 6.98 (m, 2H), 5.54 (s, 1H), 5.41 (app t, *J* = 9.4 Hz, 1H), 5.29 (dd, *J* = 9.2, 7.8 Hz, 1H), 5.20 (d, *J* = 7.8 Hz, 1H), 4.41 (dd, *J* = 10.5, 4.9 Hz, 1H), 3.83 (dt, *J* = 12.7, 9.9 Hz, 2H), 3.67 (td, *J* = 9.8, 5.0 Hz, 1H), 2.08 (s, 3H), 2.07 (s, 3H) ppm. **^{13}C NMR (126 MHz, $CDCl_3$)** δ 170.3, 169.7, 157.0, 136.8, 129.8, 129.3, 128.4, 126.3, 123.5, 117.1, 101.7, 99.8, 78.3, 72.3, 71.9, 68.7, 66.7, 20.9, 20.8 ppm. **IR (neat)** 2878, 1747, 1598, 1489, 1374, 1219, 1058, 1030, 999, 969, 757, 693 cm^{-1}. **HRMS (ESI) m/z [M+Na]$^+$:** ($C_{23}H_{24}NaO_8$) calcd. 451.1363, found. 451.1350. **MP** 219.6-220.1 °C.

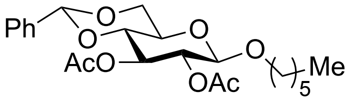

Hexyl 2,3-di-*O*-acetyl-4,6-*O*-benzylidene-β-D-glucopyranoside (3h): Following general procedure B, using (2*R*,3*R*,4*S*,5*S*,6*R*)-2-(hexyloxy)-6-(hydroxymethyl)tetrahydro-2*H*-pyran-3,4,5-triol (1.32 g, 5.0 mmol, 1.0 equivalent), the crude was purified by crystallization (EtOAc/hexane) to afford **3h** as a white solid (1.62 g, 74% yield). **^{1}H NMR (500 MHz, $CDCl_3$)** δ 7.49 – 7.42 (m, 2H), 7.42 – 7.33 (m, 3H), 5.52 (s, 1H), 5.34 (app t, *J* = 9.5 Hz, 1H), 5.02 (dd, *J* = 9.3, 7.9 Hz, 1H), 4.59 (d, *J* = 7.9 Hz, 1H), 4.39 (dd, *J* = 10.5, 5.0 Hz, 1H), 3.89 (dt, *J* = 9.6, 6.4 Hz, 1H), 3.83 (app t, *J* = 10.3 Hz, 1H), 3.72 (app t, *J* = 9.5 Hz, 1H), 3.58 – 3.48 (m, 2H), 2.08 (s, 6H), 2.07 (s, 6H), 1.64 – 1.51 (m, 2H), 1.38 – 1.25 (m, 6H), 0.94 – 0.88 (m, 3H) ppm. **^{13}C NMR (126 MHz, $CDCl_3$)** δ 170.3, 169.7, 137.0, 129.2, 128.4, 126.3, 101.6, 101.6, 78.5, 72.5, 72.0, 70.6, 68.7, 66.5, 31.6, 29.5, 25.6, 22.7, 20.9, 20.8, 14.1 ppm. **IR (neat)** 2932, 2971, 1750, 1372, 1233, 1215, 1089, 1029, 966, 761, 696 cm^{-1}. **HRMS (ESI) m/z [M+Na]$^+$:** ($C_{23}H_{32}NaO_8$) calcd. 459.1989, found. 459.1990. **MP** 112.3-113.2 °C.

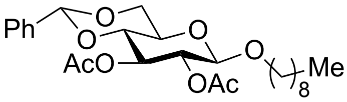

Nonyl 2,3-di-*O*-acetyl-4,6-*O*-benzylidene-β-D-glucopyranoside (3i): Following general procedure B, using (2*R*,3*S*,4*S*,5*R*,6*R*)-2-(hydroxymethyl)-6-(nonyloxy)tetrahydro-2*H*-pyran-3,4,5-triol (1.53 g, 5.0 mmol, 1.0 equivalent), the crude was purified by crystallization (EtOAc/hexane) to afford **3i** as a white solid (1.75 g, 73% yield). **^{1}H NMR (500 MHz, $CDCl_3$)** δ 7.47 – 7.40 (m, 2H), 7.39 – 7.31 (m, 3H), 5.50 (s, 1H), 5.32 (app t, *J* = 9.5 Hz, 1H), 4.99 (dd, *J* = 9.3, 7.9 Hz, 1H), 4.57 (d, *J* = 7.9 Hz, 1H), 4.36 (dd, *J* = 10.6, 5.0 Hz, 1H), 3.90 – 3.76 (m, 2H), 3.70 (app t, *J* = 9.6 Hz, 1H), 3.56 – 3.45 (m, 2H), 2.05 (s, 3H), 2.05 (s, 3H), 1.60 – 1.51 (m, 2H), 1.33 – 1.23 (m, 12H), 0.88 (t, *J* = 6.9 Hz, 3H) ppm. **^{13}C NMR (126 MHz, $CDCl_3$)** δ 170.3, 169.7, 137.0, 129.2, 128.4, 126.3, 101.6, 101.6, 78.6, 72.5, 72.0, 70.6, 68.7, 66.5, 32.0, 29.7, 29.6, 29.4, 29.4, 25.9, 22.8, 20.9, 20.8, 14.2 ppm. **IR (neat)** 2922, 2853, 1750, 1371, 1233, 1215, 1089, 1029, 966, 761, 697 cm^{-1}. **HRMS (ESI) m/z [M+Na]$^+$:** ($C_{26}H_{38}NaO_8$) calcd. 501.2459, found. 501.2444. **MP** 109.9-110.6 °C.

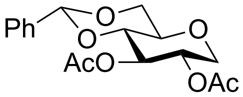

2,3-Di-*O*-acetyl-1,5-anhydro-4,6-*O*-benzylidene-D-glucitol (3p): Following general procedure B, using (2*R*,3*S*,4*R*,5*S*)-2-(hydroxymethyl)tetrahydro-2*H*-pyran-3,4,5-triol (0.82 g, 5.0 mmol, 1.0 equivalent), the crude was purified by crystallization (EtOAc/hexane) to afford **3p** as a white solid (0.8 g, 50% yield). **^{1}H NMR (500 MHz, $CDCl_3$)** δ 7.47 – 7.40 (m, 2H), 7.40 – 7.31 (m, 3H), 5.50 (s, 1H), 5.33 (app t, *J* = 9.5 Hz, 1H), 5.04 (ddd, *J* = 10.6, 9.3, 5.8 Hz, 1H), 4.34 (dd, *J* = 10.5, 5.0 Hz, 1H), 4.13 (dd, *J* = 11.1, 5.9 Hz, 1H), 3.72 (app t, *J* = 10.3 Hz, 1H), 3.63 (app t, *J* = 9.5 Hz, 1H), 3.47 (ddd, *J* = 10.0, 9.3, 5.0 Hz, 1H), 3.40 (dd, *J* = 11.2, 10.6 Hz, 1H), 2.07 (s, 3H), 2.05 (s, 3H) ppm. **^{13}C NMR (126 MHz, $CDCl_3$)** δ 170.3, 137.0, 129.9, 129.2, 128.4, 126.3, 101.6, 79.0, 72.6, 71.6, 69.8, 68.8, 67.6, 21.0, 20.9 ppm. **IR (neat)** 2983, 2945, 2869, 1739, 1368, 1240, 1220, 1096, 982, 966, 921, 767, 700 cm^{-1}. **HRMS (ESI) m/z [M+Na]$^+$:** ($C_{17}H_{20}NaO_7$) calcd. 359.1101, found. 359.1104. **MP** 118.0-118.9 °C.

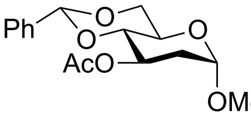

Methyl 3-*O*-acetyl-4,6-*O*-benzylidene-2-deoxy-α-D-arabino-hexopyranoside (3q): Following general procedure B, using (2*R*,3*S*,4*R*,6*S*)-2-(hydroxymethyl)-6-methoxytetrahydro-2*H*-pyran-3,4-diol (0.89 g, 5.0 mmol, 1.0 equivalent), the crude was purified by crystallization (EtOAc/hexane) to afford **3q** as a white solid (0.8 g, 52% yield). **^{1}H NMR (500 MHz, $CDCl_3$)** δ 7.50 – 7.44 (m, 2H), 7.39 – 7.32 (m, 3H), 5.56 (s, 1H), 5.34 (ddd, *J* = 11.1, 9.6, 5.3 Hz, 1H), 4.82 – 4.78 (m, 1H), 4.27 (dd, *J* = 10.3, 4.8 Hz, 1H), 3.91 (dddd, *J* = 10.2, 9.4, 4.8, 0.6 Hz, 1H), 3.77 (app t, *J* = 10.3 Hz, 1H), 3.67 (app t, *J* = 9.5 Hz, 1H), 3.36 (s, 3H), 2.36 (ddd, *J* = 13.1, 5.4, 1.2 Hz, 1H), 2.05 (s, 3H), 1.77 (ddd, *J* = 13.0, 11.2, 3.8 Hz,

1H) ppm. **^{13}C NMR (126 MHz, $CDCl_3$)** δ 170.2, 137.4, 129.2, 128.4, 126.3, 101.9, 98.8, 80.6, 69.2, 68.1, 63.1, 54.9, 35.7, 21.3 ppm. **IR (neat)** 2950, 2897, 2840, 1729, 1368, 1240, 1089, 1036, 977, 921, 768, 700 cm^{-1}. **HRMS (ESI) m/z [M+Na]$^+$:** ($C_{16}H_{20}NaO_6$) calcd. 331.1152, found. 331.1151. **MP** 126.5-127.8 °C.

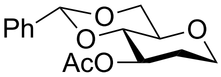

3-*O*-Acetyl-1,5-anhydro-4,6-*O*-benzylidene-2-deoxy-D-arabinohexitol (3r): To a solution of (2*R*,4a*R*,8*R*,8a*S*)-2-phenylhexahydropyrano[3,2-*d*][1,3]dioxin-8-ol[12] (1.39 g, 5.9 mmol, 1 equiv) in pyridine (9.4 mL, 0.63 M), acetic anhydride (4.4 mL, 47 mmol, 8 equivalents) was added dropwise at rt and the reaction mixture was stirred for 48 h. Afterwards, the reaction was diluted with EtOAc and quenched with HCl (1 M) aqueous solution and washed with HCl (1 M) aqueous solution, saturated aqueous Na_2CO_3 solution and brine, dried over anhydrous $MgSO_4$, filtered and concentrated under vacuum. The crude was purified by crystallization (EtOAc/hexane) to afford **3r** as a white solid (1.12 g, 68% yield). **^{1}H NMR (400 MHz, $CDCl_3$)** δ 7.50 – 7.44 (m, 2H), 7.39 – 7.34 (m, 3H), 5.56 (s, 1H), 5.06 (ddd, *J* = 11.0, 9.4, 5.2 Hz, 1H), 4.30 (dd, *J* = 10.4, 4.9 Hz, 1H), 3.99 (ddd, *J* = 11.9, 5.4, 1.5 Hz, 1H), 3.73 (app t, *J* = 10.3 Hz, 1H), 3.67 – 3.60 (m, 2H), 3.44 (ddd, *J* = 10.0, 9.2, 4.9 Hz, 1H), 2.24 – 2.20 (ddt, *J* = 13.2, 5.3, 2.1 Hz, 1H), 2.07 (s, 3H), 1.79 (tdd, *J* = 12.9, 11.0, 5.3 Hz, 1H) ppm.**^{13}C NMR (101 MHz, $CDCl_3$)** δ 170.6, 137.5, 129.2, 128.4, 126.3, 101.8, 80.5, 72.0, 71.2, 69.0, 66.2, 31.7, 21.3 ppm. **IR (neat)** 2942, 2871, 1736, 1419, 1239, 1095, 974, 761, 700, 654 cm^{-1}. **HRMS (ESI) m/z [M+Na]$^+$:** ($C_{15}H_{18}NaO_5$) calcd. 301.1046, found. 301.1053. **MP** 94.5-95.6 °C.

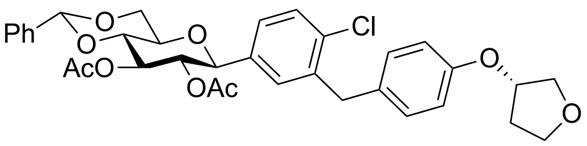

4-Chloro-3-(4-(((*S*)-tetrahydrofuran-3-yl)oxy)benzyl)phenyl) 2,3-di-*O*-acetyl-4,6-*O*-benzylidene-β-D-glucopyranoside (3w): Following general procedure B, using (2*S*,3*R*,4*R*,5*S*,6*R*)-2-(4-chloro-3-(4-(((*S*)-tetrahydrofuran-3-yl)oxy)benzyl)phenyl)-6-(hydroxymethyl)tetrahydro-2*H*-pyran-3,4,5-triol (2.25 g, 5.0 mmol, 1.0 equivalent), the crude was purified by crystallization (EtOAc/hexane) to afford **3w** as a white solid (2.34 g, 75% yield). **^{1}H NMR (400 MHz, $CDCl_3$)** δ 7.49 – 7.43 (m, 2H), 7.41 – 7.33 (m, 4H), 7.21 – 7.16 (m, 1H), 7.11 (d, *J* = 2.1 Hz, 1H), 7.06 (d, *J* = 8.6 Hz, 2H), 6.79 (d, *J* = 8.6 Hz, 2H), 5.55 (s, 1H), 5.41 (app t, *J* = 9.4 Hz, 1H), 5.08 (app t, *J* = 9.5 Hz, 1H), 4.89 (ddt, *J* = 6.4, 4.6, 2.3 Hz, 1H), 4.42 (d, *J* = 9.8 Hz, 1H), 4.38 (dd, *J* = 10.4, 4.8 Hz, 1H), 4.09 – 3.94 (m, 5H), 3.89 (td, *J* = 8.2, 4.6 Hz, 1H), 3.81 (td, *J* = 9.9, 2.7 Hz, 2H), 3.68 (td, *J* = 9.6, 4.8 Hz, 1H), 2.24 – 2.10 (m, 2H), 2.04 (s, 3H), 1.74 (s, 3H) ppm. **^{13}C NMR (101 MHz, $CDCl_3$)** δ 170.4, 169.2, 156.1, 139.1, 137.0, 135.5, 134.8, 131.7, 130.1, 130.0, 130.0, 129.3, 128.4, 126.3, 126.3, 115.6, 101.7, 80.5, 79.0, 73.4, 73.3, 73.1, 71.4, 68.8, 67.3, 38.4, 33.2, 21.0, 20.5 ppm. **IR (neat)** 2936, 2872, 1742,

1507, 1372, 1239, 1109, 1042, 1006, 920, 831, 761, 702 cm^{-1}. **HRMS (ESI) m/z [M+Na]$^+$:** ($C_{34}H_{35}ClNaO_9$) calcd. 645.1862, found. 645.1887. **MP** 177.7-178.8 °C.

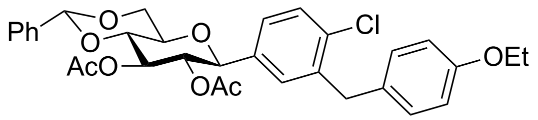

4-Chloro-3-(4-ethoxybenzyl)phenyl 2,3-di-*O*-acetyl-4,6-*O*-benzylidene-β-D-glucopyranoside (3x): Following general procedure C, using (2*S*,3*R*,4*R*,5*S*,6*R*)-2-(4-chloro-3-(4-ethoxybenzyl)phenyl)-6-(hydroxymethyl)tetrahydro-2*H*-pyran-3,4,5-triol (2.04 g, 5.0 mmol, 1.0 equivalent), the crude was purified by crystallization (EtOAc/hexane) to afford **3x** as a white solid (1.83 g, 63% yield). **^{1}H NMR (300 MHz, $CDCl_3$)** δ 7.50 – 7.43 (m, 2H), 7.40 – 7.33 (m, 4H), 7.21 – 7.16 (m, 1H), 7.11 – 7.03 (m, 3H), 6.85 – 6.78 (m, 2H), 5.55 (s, 1H), 5.40 (app t, *J* = 9.4 Hz, 1H), 5.13 – 5.03 (m, 1H), 4.39 (dd, *J* = 14.0, 10.0 Hz, 2H), 4.07 – 3.93 (m, 4H), 3.81 (td, *J* = 9.8, 1.9 Hz, 2H), 3.72 – 3.63 (m, 1H), 2.04 (s, 3H), 1.73 (s, 3H), 1.40 (t, *J* = 7.0 Hz, 3H) ppm. **^{13}C NMR (75 MHz, $CDCl_3$)** δ 170.4, 169.2, 157.7, 139.3, 137.0, 135.4, 134.8, 131.2, 130.1, 130.0, 129.9, 129.3, 128.4, 126.3, 126.2, 114.7, 101.7, 80.5, 79.0, 73.4, 73.1, 71.4, 68.8, 63.6, 38.4, 21.0, 20.5, 15.0 ppm. **IR (neat)** 2978, 2891, 1742, 1509, 1476, 1371, 1240, 1112, 1041, 1006, 921, 829, 761, 703 cm^{-1}. **HRMS (ESI) m/z [M+Na]$^+$:** ($C_{32}H_{33}ClNaO_8$) calcd. 603.1756, found. 603.1752. **MP** 163.5-164.8 °C.

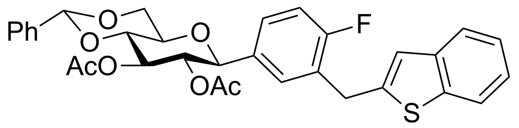

3-(Benzo[b]thiophen-2-ylmethyl)-4-fluorophenyl 2,3-di-*O*-acetyl-4,6-*O*-benzylidene-β-D-glucopyranoside (3y): Following general procedure C, using (2*S*,3*R*,4*R*,5*S*,6*R*)-2-(3-(benzo[*b*]thiophen-2-ylmethyl)-4-fluorophenyl)-6-(hydroxymethyl)tetrahydro-2*H*-pyran-3,4,5-triol (2.02 g, 5.0 mmol, 1.0 equivalent), the crude was purified by crystallization (EtOAc/hexane) to afford **3y** as a white solid (2.02 g, 70% yield). **^{1}H NMR (400 MHz, $CDCl_3$)** δ 7.77 – 7.72 (m, 1H), 7.69 – 7.65 (m, 1H), 7.49 – 7.44 (m, 2H), 7.41 – 7.35 (m, 3H), 7.34 – 7.24 (m, 4H), 7.08 (t, *J* = 8.8 Hz, 1H), 6.99 (s, 1H), 5.55 (s, 1H), 5.42 (app t, *J* = 9.4 Hz, 1H), 5.11 (app t, *J* = 9.5 Hz, 1H), 4.45 (d, *J* = 9.8 Hz, 1H), 4.39 (dd, *J* = 10.4, 4.8 Hz, 1H), 4.24 (q, *J* = 16.0 Hz, 2H), 3.82 (td, *J* = 9.8, 4.4 Hz, 2H), 3.69 (td, *J* = 9.6, 4.8 Hz, 1H), 2.04 (s, 3H), 1.73 (s, 3H) ppm. **^{13}C NMR (101 MHz, $CDCl_3$)** δ 169.8 (d, *J* = 114.9 Hz), 161.0 (d, *J* = 248.1 Hz), 143.1, 140.1, 139.9, 137.0, 132.8 (d, *J* = 3.6 Hz), 130.2 (d, *J* = 4.5 Hz), 129.3, 128.4, 127.5 (d, *J* = 8.5 Hz), 126.8 (d, *J* = 16.2 Hz), 126.3, 124.4, 124.0, 123.2, 122.3, 122.1, 115.9 (d, *J* = 22.5 Hz), 101.7, 80.5, 79.0, 73.5, 73.1, 71.4, 68.8, 29.9 (d, *J* = 3.5 Hz), 20.9, 20.4 ppm. **^{19}F NMR (376 MHz, $CDCl_3$)** δ -117.4 ppm. **IR (neat)** 2885, 1745, 1499, 1371, 1220, 1102, 1030, 960, 831, 746, 698, 601 cm^{-1}. **HRMS (ESI) m/z [M+Na]$^+$:** ($C_{32}H_{29}FNaO_7S$) calcd. 599.1510, found. 599.1493. **MP** 184.2-185.4 °C.

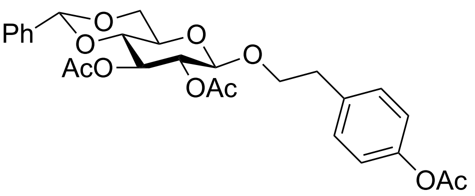

4-Acetoxyphenylethyl 2,3-di-*O*-acetyl-4,6-*O*-benzylidene-β-D-glucopyranoside (3za): Following general procedure B, using (2*R*,3*S*,4*S*,5*R*,6*R*)-2-(hydroxymethyl)-6-(4-hydroxyphenethoxy)tetrahydro-2*H*-pyran-3,4,5-triol (1.5 g, 5.0 mmol, 1.0 equivalent), the crude was purified by crystallization (EtOAc/hexane) to afford **3za** as a white solid (2.1 g, 80% yield). **^{1}H NMR (500 MHz, CDCl$_3$)** δ 7.46 – 7.40 (m, 2H), 7.38 – 7.33 (m, 3H), 7.23 – 7.17 (m, 2H), 7.02 – 6.96 (m, 2H), 5.49 (s, 1H), 5.29 (app t, *J* = 9.5 Hz, 1H), 5.00 (dd, *J* = 9.3, 7.8 Hz, 1H), 4.57 (d, *J* = 7.8 Hz, 1H), 4.36 (dd, *J* = 10.5, 5.0 Hz, 1H), 4.11 (ddd, *J* = 9.5, 6.6, 5.8 Hz, 1H), 3.79 (app t, *J* = 10.3 Hz, 1H), 3.73 – 3.63 (m, 2H), 3.51 (td, *J* = 9.7, 5.0 Hz, 1H), 2.94 – 2.81 (m, 2H), 2.28 (s, 3H), 2.04 (s, 3H), 1.92 (s, 3H) ppm. **^{13}C NMR (126 MHz, CDCl$_3$)** δ 170.3, 169.7, 169.7, 149.3, 136.9, 136.2, 130.1, 129.3, 128.4, 126.3, 121.5, 101.6, 101.4, 78.5, 72.3, 71.9, 70.7, 68.7, 66.5, 35.5, 21.2, 20.9, 20.7 ppm. **IR (neat)** 2887, 1749, 1727, 1509, 1369, 1239, 1215, 1092, 1029, 991, 911, 763, 701 cm^{-1}. **HRMS (ESI) m/z [M+H]$^+$:** ($C_{27}H_{31}O_{10}$) calcd. 515.1912, found. 515.1899. **MP** 151.9-153.4 °C.

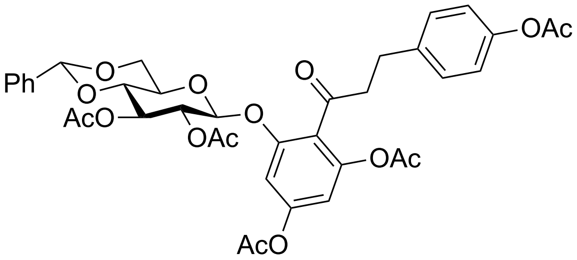

3,5-Diacetoxy-2-(4-acetoxyphenylpropanoyl)phenyl 2,3-di-*O*-acetyl-4,6-*O*-benzylidene-β-D-glucopyranoside (3zb): Following general procedure C, using 1-(2,4-dihydroxy-6-(((2*S*,3*R*,4*S*,5*S*,6*R*)-3,4,5-trihydroxy-6-(hydroxymethyl)tetrahydro-2*H*-pyran-2-yl)oxy)phenyl)-3-(4-hydroxyphenyl)propan-1-one (2.2 g, 5.0 mmol, 1.0 equivalent), pyridine (20 mL, 0.25 M) and acetic anhydride (15 mL, 160 mmol, 32 equivalents), the crude was purified by crystallization (EtOAc/hexane) to afford **3zb** as a white solid (1.84 g, 50% yield). **^{1}H NMR (400 MHz, CDCl$_3$)** δ 7.48 – 7.41 (m, 2H), 7.40 – 7.33 (m, 3H), 7.26 (d, *J* = 8.5 Hz, 3H), 7.04 – 6.95 (m, 2H), 6.77 (d, *J* = 2.0 Hz, 1H), 6.71 (d, *J* = 2.0 Hz, 1H), 5.52 (s, 1H), 5.36 (app t, *J* = 9.3 Hz, 1H), 5.23 (dd, *J* = 9.1, 7.7 Hz, 1H), 5.13 (d, *J* = 7.7 Hz, 1H), 4.41 (dd, *J* = 10.5, 4.8 Hz, 1H), 3.79 (td, *J* = 9.8, 8.3 Hz, 2H), 3.67 (td, *J* = 9.6, 4.8 Hz, 1H), 3.20 – 3.10 (m, 1H), 3.07 – 2.98 (m, 1H), 2.97 – 2.88 (m, 2H), 2.30 (s, 3H), 2.28 (s, 3H), 2.09 (s, 3H), 2.06 (s, 3H), 2.01 (s, 3H) ppm. **^{13}C NMR (101 MHz, CDCl$_3$)** δ 200.5, 170.1, 169.7, 168.8, 168.5, 154.5, 152.1, 149.1, 148.1, 138.8, 136.7, 129.7, 129.4, 128.4, 126.3, 123.2, 121.6, 111.9, 106.8, 101.8, 99.9, 78.0, 71.6, 71.5, 68.5, 66.9, 45.9, 28.9, 21.3, 21.3, 20.9, 20.7, 20.6 ppm. **IR (neat)**

1749, 1708, 1609, 1368, 1187, 1061, 1026, 906, 762, 695 cm^{-1}. **HRMS (ESI) m/z [M+Na]$^+$:** ($C_{38}H_{38}NaO_{15}$) calcd. 757.2103, found. 757.2091. **MP** 180.5-181.7 °C.

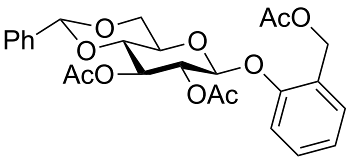

(2-(Acetoxymethyl)phenyl 2,3-di-*O*-acetyl-4,6-*O*-benzylidene-β-D-glucopyranoside (3zc): Following general procedure C, using (2*R*,3*S*,4*S*,5*R*,6*S*)-2-(hydroxymethyl)-6-(2-(hydroxymethyl)phenoxy)tetrahydro-2*H*-pyran-3,4,5-triol (1.43 g, 5.0 mmol, 1.0 equivalent), the crude was purified by crystallization (EtOAc/hexane) to afford **3zc** as a white solid (1.25 g, 50% yield). **^{1}H NMR (400 MHz, CDCl$_3$)** δ 7.49 – 7.42 (m, 2H), 7.41 – 7.34 (m, 4H), 7.33 – 7.28 (m, 1H), 7.14 – 7.03 (m, 2H), 5.53 (s, 1H), 5.41 (app t, *J* = 9.2 Hz, 1H), 5.32 (dd, *J* = 8.9, 7.4 Hz, 1H), 5.20 (d, *J* = 7.5 Hz, 1H), 5.11 (q, *J* = 12.9 Hz, 2H), 4.40 (dd, *J* = 10.5, 4.9 Hz, 1H), 3.84 (td, *J* = 9.9, 6.3 Hz, 2H), 3.67 (td, *J* = 9.7, 4.9 Hz, 1H), 2.16 – 2.05 (m, 9H) ppm. **^{13}C NMR (101 MHz, CDCl$_3$)** δ 170.8, 170.2, 169.6, 154.6, 136.8, 129.8, 129.7, 129.3, 128.4, 126.3, 126.3, 123.6, 115.8, 101.7, 99.9, 78.2, 72.1, 71.8, 68.6, 66.6, 61.2, 21.1, 20.9, 20.8 ppm. **IR (neat)** 2928, 1748, 1457, 1374, 1232, 1027, 969, 756, 696 cm^{-1}. **HRMS (ESI) m/z [M+Na]$^+$:** ($C_{26}H_{28}NaO_{10}$) calcd. 523.1575, found: 523.1558. **MP** 157.9-159.8 °C.

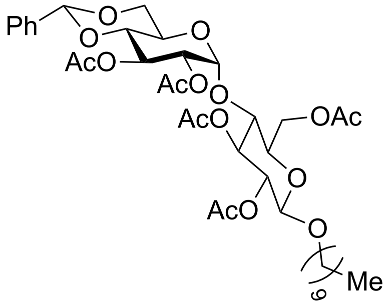

Decyl [(2,3-di-*O*-acetyl-4,6-*O*-benzylidene-α-D-glucopyranosyl)-(1→4)-(2,3,6-tri-*O*-acetyl-β-D-glucopyranoside)] (3zd): Following general procedure C, using (2*R*,3*R*,4*S*,5*S*,6*R*)-2-(((2*R*,3*S*,4*R*,5*R*,6*R*)-6-(decyloxy)-4,5-dihydroxy-2-(hydroxymethyl)tetrahydro-2*H*-pyran-3-yl)oxy)-6-(hydroxymethyl)tetrahydro-2*H*-pyran-3,4,5-triol (2.41 g, 5.0 mmol, 1.0 equivalent), pyridine (20 mL, 0.25 M) and acetic anhydride (15 mL, 160 mmol, 32 equivalents), the crude was purified by crystallization (EtOAc/hexane) to afford **3zd** as a white solid (2.03 g, 52% yield). **^{1}H NMR (300 MHz, CDCl$_3$)** δ 7.46 – 7.38 (m, 2H), 7.38 – 7.30 (m, 3H), 5.51 – 5.41 (m, 2H), 5.35 (d, *J* = 4.1 Hz, 1H), 5.25 (dd, *J* = 9.4, 8.8 Hz, 1H), 4.88 (dd, *J* = 10.2, 4.1 Hz, 1H), 4.81 (dd, *J* = 9.5, 7.9 Hz, 1H), 4.56 – 4.47 (m, 2H), 4.31 – 4.19 (m, 2H), 4.00 (dd, *J* = 9.6, 8.8 Hz, 1H), 3.91 – 3.78 (m, 2H), 3.77 – 3.57 (m, 3H), 3.45 (dt, *J* = 9.7, 6.7 Hz, 1H), 2.11 (s, 3H), 2.05 (s, 3H), 2.04 (s, 3H), 2.01 (s, 3H), 2.01 (s, 3H), 1.62 – 1.49 (m, 2H), 1.25 (s, 14H), 0.92 – 0.83 (m, 3H) ppm. **^{13}C NMR (75 MHz, CDCl$_3$)** δ 170.9, 170.4, 170.4, 169.9, 169.8, 136.8, 129.2, 128.3, 126.3, 101.7, 100.4, 96.6,

78.9, 75.7, 73.0, 72.4, 72.2, 71.0, 70.3, 68.6, 68.6, 63.8, 62.8, 32.0, 29.7, 29.7, 29.5, 29.4, 25.9, 22.8, 21.1, 20.9, 20.9, 20.8, 20.7, 14.2 ppm. **IR (neat)** 2926, 2852, 1743, 1368, 1227, 1030, 980, 701 cm^{-1}. **HRMS (ESI) m/z [M+Na]$^+$:** ($C_{39}H_{56}NaO_{16}$) calcd. 803.3461, found. 803.3444. **MP** 163.1-164.0 °C.

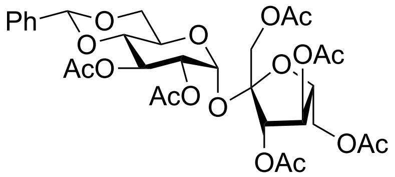

1',2,3,3',4',6'-Hexa-*O*-acetyl-4,6-*O*-benzylidenesucrose (3ze) (Khan, R. *Carbohydr. Res.* **1974**, *32*, 375-379) To a stirred solution of sucrose (1.7 g, 4.97 mmol) in dry pyridine (40 mL), benzylidene bromide (1.9 mL, 10.93 mmol) was added, and the mixture was heated at 85 ºC for 1 h. After TLC and mass spectrometry analysis, an additional equivalent of benzylidene bromide (0.86 mL, 4.97 mmol) was added, and the reaction temperature was raised to 95°C for 0.5 h. The mixture was then treated with acetic anhydride (5 mL) at 0 °C and stirred at rt overnight. The solution was poured into ice-water and extracted with chloroform (three times), and the combined organic layers were washed with 1 N HCl, water, brine, and dried (Na_2SO_4). The solution was concentrated and subjected to column chromatography over silica gel (gradient 20→40% ethyl acetate in hexanes) to isolate compound **1** (0.87 g, 26%) as a white crystalline solid. **TLC**: R_f = 0.30 (40:60 EtOAc:Hexanes; UV, H_2SO_4:EtOH). $[\alpha]_D^{20}$ = (+) 43.8 (*c*, 1%, $CHCl_3$) **^{1}H NMR (500 MHz, $CDCl_3$)** δ 7.43 (dd, *J* = 6.7, 2.8 Hz, 2H, Ar), 7.37 – 7.32 (m, 3H, Ar), 5.69 (d, *J* = 3.9 Hz, 1H, H1'), 5.54 (t, *J* = 9.9 Hz, 1H, H3'), 5.50 (s, 1H, PhCH), 5.45 (d, *J* = 5.8 Hz, 1H, H3), 5.37 (t, *J* = 5.8 Hz, 1H, H4), 4.87 (dd, *J* = 10.1, 3.9 Hz, 1H, H2'), 4.37 (dd, *J* = 10.4, 4.9 Hz, 1H, H6'eq), 4.32 (dd, *J* = 12.0, 3.8 Hz, 1H, H6a), 4.25 (dd, *J* = 12.0, 6.4 Hz, 1H, H6b), 4.22 (td, *J* = 6.2, 3.9 Hz, 1H, H5), 4.20 (dt, *J* = 9.9, 3.6 Hz, 1H, H5'), 4.19 (d, *J* = 12.1 Hz, 1H, H1a), 4.15 (d, *J* = 12.1 Hz, 1H, H1, H1b), 3.74 (t, *J* = 10.3 Hz, 1H, H6'ax), 3.67 (t, *J* = 9.7 Hz, 1H, H4'), 2.19 (s, 3H, $COCH_3$), 2.11 (s, 3H, $COCH_3$), 2.10 (s, 3H, $COCH_3$), 2.09 (s, 3H, $COCH_3$), 2.07 (s, 3H, $COCH_3$), 2.05 (s, 3H, $COCH_3$). **^{13}C NMR (126 MHz, $CDCl_3$)** δ 170.7 (C=O), 170.4 (C=O), 170.2 (C=O), 169.9 (C=O), 169.9 (C=O), 169.9 (C=O), 137.0, 129.1, 128.3, 126.3 (aromatic), 104.1 (C2), 101.7 (PhCH), 90.6 (C1'), 79.3 (C5'), 79.0 (C4'), 75.7 (C3), 74.9 (C4), 71.1 (C2'), 68.8 (C3'), 68.5 (C6'), 63.6 (C1), 63.5 (C6), 63.2 (C5), 20.9 (CH_3), 20.8 (CH_3), 20.7 (CH_3), 20.7 (CH_3), 20.6 (CH_3), 20.6 (CH_3). **HRMS-ESI (m/z):** [M+Na]$^+$ calculated for $[C_{31}H_{38}O_{17}Na]^+$: 705.2001, found: 705.1985

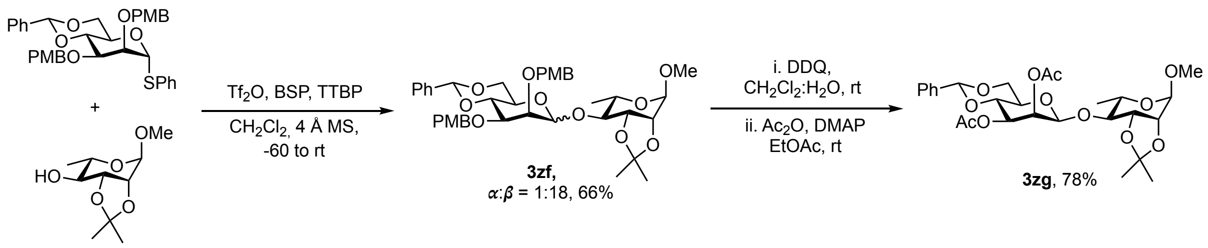

Methyl 2,3-di-*O*-*p*-methoxybenzyl-4,6-*O*-benzylidiene-*β*-D-mannopyranosyl-(1→4)-2,3-*O*-isopropylidene-*β*-L-rhamnopyranoside (3zf): To a stirred solution containing thioglycoside **2** (1.82 g, 3.03 mmol; Crich, D. Li, H. *J. Org. Chem.* **2002**, *67*, 4640-4646), TTBP (1.51 g, 6.06 mmol), BSP (0.63 g, 3.03 mmol), and activated 4 Å powdered molecular sieves in CH_2Cl_2 (30 mL, 0.1 M) was added Tf_2O (0.51 mL, 3.03 mmol) at -60 °C under nitrogen. After 5 min, a solution of the glycosyl acceptor **3** (0.33 g, 1.51 mmol) in CH_2Cl_2 (1.0 M) was added. The reaction mixture was stirred at -60 °C for 1 h and then warmed to 0 °C, quenched with triethylamine (0.5 mL), diluted with EtOAc, and filtered. The filtrate was washed with water, the water phase was extracted with EtOAc three times, combined, washed with brine, dried (Na_2SO_4), and concentrated. The crude reaction mixture was subjected to column chromatography on silica gel (gradient 10→35% ethyl acetate in hexanes) to yield mixture of anomers (712 mg, 66%, *α:β*= 1:18). Repeated chromatography over silica gel gave a mixture of anomers (343 mg) and the pure *β*-mannoside **4** (314 mg) as a white foam. **TLC**: R_f = 0.35 (30:70 EtOAc:Hexanes; UV, H_2SO_4:EtOH). $[\alpha]_D^{20}$ = (-) 41.5 (*c*, 1%, $CHCl_3$). **^{1}H NMR (500 MHz, $CDCl_3$)** δ 7.51 (d, *J* = 7.0 Hz, 2H, Ar), 7.39 – 7.32 (m, 5H, Ar), 7.22 (d, *J* = 8.3 Hz, 2H, Ar), 6.85 (m, 4H, Ar), 5.62 (s, 1H, PhCH), 4.98 (br s, 1H, H1'), 4.88 (br s, 1H, H1), 4.85 (d, *J* = 11.8 Hz, 1H, $PMPCH_2$), 4.75 (d, *J* = 11.8 Hz, 1H, $PMPCH_2$), 4.60 (d, *J* = 12.0 Hz, 1H, $PMPCH_2$), 4.54 (d, *J* = 12.1 Hz, 1H, $PMPCH_2$), 4.27 (dd, *J* = 10.4, 4.7 Hz, 1H, H6'eq), 4.17 (t, *J* = 9.5 Hz, 1H, H4'), 4.13 – 4.08 (m, 2H, H2,H4), 3.96 (t, *J* = 10.3 Hz, 1H, H6'ax), 3.93 (s, 1H, H2'), 3.81 (s, 3H, OCH_3), 3.80 (s, 3H, OCH_3), 3.69 – 3.64 (m, 2H, H3, H5), 3.60 (dd, *J* = 9.9, 2.7 Hz, 1H, H3'), 3.40 (s, 3H, OCH_3), 3.35 – 3.28 (m, 1H, H5'), 1.51 (s, 3H, CH_3), 1.36 (d, *J* = 6.1 Hz, 3H, CH_3), 1.35 (s, 3H, CH_3). **^{13}C NMR (126 MHz, $CDCl_3$)** δ 159.3, 159.2, 137.7, 130.9, 130.6, 130.1, 129.2, 128.9, 128.2, 126.1, 113.8, 113.6, 109.4 (aromatic), 101.4 (PhCH), 100.2 (C1'), 98.0 (C1), 78.8 (C4'), 78.5 (C2), 77.8 (C3), 77.6 (C2') 76.2 (C3'), 75.9 (C4), 74.5 ($PMPCH_2$), 71.9 ($PMPCH_2$), 68.7 (C6'), 67.8 (C5'), 64.3 (C5), 55.3 (OCH_3), 55.0 (OCH_3), 27.9 (CH_3), 26.5 (CH_3), 17.8 (CH_3). **HRMS-ESI (m/z):** $[M+Na]^+$ calculated for $[C_{39}H_{48}O_{12}Na]^+$: 731.3038, found: 731.3025.

Methyl 2,3-di-*O*-acetyl-4,6-*O*-benzylidiene-*β*-D-mannopyranosyl-(1→4)-2,3-*O*-isopropylidene-*β*-L-rhamnopyranoside (3zg): To an ice-cold stirred solution of **3zf** (296 mg,

0.427 mmol) in CH_2Cl_2:H_2O (10:1, 4 mL), DDQ (386 mg, 1.67 mmol) was added and stirring was continued for 6 h with a gradual increase in temperature to rt. After complete consumption of the starting material, the reaction mixture was quenched with saturated aqueous $NaHCO_3$, diluted with chloroform and washed with water. The water phase was extracted with chloroform (twice), and the combined organic layers were washed with water, brine, dried, and concentrated. TLC and HRMS analysis showed a mixture of the desired diol and a *p*-methoxybenzoate ester, therefore the crude mixture was dissolved in methanol (3 mL), treated with NaOMe for 1 h, neutralized with IRC 120 H^+ resin, filtered, and concentrated. The residue was dissolved in ethyl acetate, treated with Ac_2O (0.14 mL, 1.66 mmol) and DMAP (50 mg, 0.414 mmol) and stirred for 1 h, after which it was concentrated under vacuum, and purified by silica gel column chromatography (gradient 10→40% ethyl acetate in hexanes) to isolate compound **5** (179 mg, 78%) as a white solid. **TLC**: R_f = 0.30 (30:70 EtOAc:Hexanes; UV, H_2SO_4:EtOH). $[\alpha]_D^{20}$ = (+) 93.2 (*c*, 1%, $CHCl_3$). **^{1}H NMR (500 MHz, $CDCl_3$)** δ 7.48 – 7.42 (m, 2H), 7.35 (m, 3H), 5.55 (s, 1H, PhCH), 5.54 (dd, *J* = 3.4, 0.9 Hz, 1H, H2'), 5.26 (s, 1H, H1'), 5.16 (dd, *J* = 10.3, 2.7 Hz, 1H, H3'), 4.83 (s, 1H, H1), 4.30 (dd, *J* = 10.4, 4.7 Hz, 1H, H6'eq), 4.14 (dd, *J* = 7.2, 5.5 Hz, 1H, H3), 4.08 (d, *J* = 5.5 Hz, 1H, H2), 4.01 (t, *J* = 9.8 Hz, 1H, H4'), 3.92 (t, *J* = 10.3 Hz, 1H, H6'ax), 3.64 (dd, *J* = 10.0, 7.3 Hz, 1H, H4), 3.62 – 3.58 (m, 1H, H5), 3.48 (td, *J* = 9.6, 4.9 Hz, 1H, H5'), 3.34 (s, 3H, OCH_3), 2.16 (s, 3H, $COCH_3$), 2.02 (s, 3H, $COCH_3$), 1.51 (s, 3H, CH_3), 1.34 (s, 3H, CH_3), 1.23 (d, *J* = 5.8 Hz, 3H, CH_3). **^{13}C NMR (126 MHz, $CDCl_3$)** δ 170.2 (C=O), 170.0 (C=O), 137.1, 129.3, 128.3, 126.2, 109.5 (aromatic), 101.9 (PhCH), 98.0 (C1), 96.9 (C1'), 78.0 (C4), 77.8 (C3), 76.2 (C2), 75.9 (C4'), 70.4 (C2'), 69.8 (C3'), 68.6 (C6'), 67.4 (C5'), 63.8 (C5), 54.8 (OCH_3), 27.9 (CH_3), 26.4 (CH_3), 20.8 (CH_3), 17.4 (CH_3). **HRMS-ESI (m/z):** $[M+Na]^+$ calculated for $[C_{33}H_{42}O_{12}Na]^+$: 575.2099, found: 575.2090

C6-substituted Glycosides via dual Ni/Photoredox Catalysis

General procedure D: To an oven dried 12 mL reaction vial equipped with a Teflon-coated stirring bar, {Ir[dF(CF_3)ppy]$_2$(dtbbpy)}PF_6 (0.4 mg, 0.4 µmol, 0.2 mol%) was added as a 2 mg/mL solution in dichloromethane. Solvent was evaporated under vacuum for 30 minutes and then, 4,7-dimethyl-1,10-phenanthroline (2.5 mg, 0.12 mmol, 6 mol%), the corresponding aryl bromide (*if solid*, **4**, 0.2 mmol, 1 equivalent) and the corresponding sugar derivative (**3**, 0.24 mmol, 1.2 equivalents) were added. Subsequently, the reaction vial was brought inside a nitrogen-filled glovebox and $NiBr_2$·dme (3.1, 0.01 mmol, 5 mol%), NH_4Cl (1.1, 0.02 mmol, 10 mol%) and Na_2CO_3 (42.4 mg, 0.4 mmol, 2 equivalents) were added. Then, the vial was closed and taken outside the glovebox where benzene:*tert*-amyl alcohol (1:1 mL, 0.1 M) and the corresponding aryl bromide (*if liquid*, **4**, 0.2 mmol, 1 equivalent) were added sequentially via syringe. The reaction was stirred at 650 rpm under 456 nm light irradiation for 40 h using the UFO photorreactor. After the reaction was completed, the crude was filtered through a short celite pad eluting with ethyl acetate and concentrated under vacuum. The crude was purified by flash column chromatography.

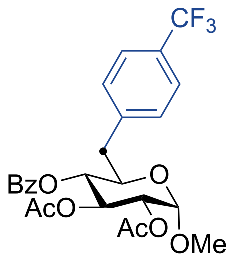

(2*S*,3*R*,4*S*,5*R*,6*R*)-5-(benzoyloxy)-2-methoxy-6-(4-(trifluoromethyl)benzyl)tetrahydro-2*H*-pyran-3,4-diyl diacetate (5a): Following general procedure D, using **3a** (87.9 mg, 0.24 mmol, 1.2 equivalents) and 1-bromo-4-(trifluoromethyl)benzene (**4a**, 28 µL, 0.2 mmol, 1 equivalent), the crude was purified by column chromatography (hexane:EtOAc, 8:1 to 6:1) to afford **5a** as a white solid (75.7 mg, 74% yield). In an independent experiment, 74.9 mg (73% yield) were obtained, giving an average yield of 74%. **^{1}H NMR (500 MHz, $CDCl_3$)** δ 8.02 – 7.96 (m, 2H), 7.63 – 7.55 (m, 1H), 7.51 – 7.42 (m, 4H), 7.35 – 7.27 (m, 2H), 5.65 (dd, *J* = 10.2, 9.4 Hz, 1H), 5.19 (app t, *J* = 9.7 Hz, 1H), 4.93 (dd, *J* = 10.2, 3.7 Hz, 1H), 4.88 (d, *J* = 3.7 Hz, 1H), 4.10 (td, *J* = 9.5, 3.4 Hz, 1H), 3.07 (s, 3H), 2.93 (dd, *J* = 14.2, 3.4 Hz, 1H), 2.86 (dd, *J* = 14.2, 9.1 Hz, 1H), 2.06 (s, 3H), 1.89 (s, 3H) ppm. **^{13}C NMR (126 MHz, $CDCl_3$)** δ 170.3, 170.1, 165.7, 141.6 (q, *J* = 1.3 Hz), 133.7, 130.0, 129.9, 129.1, 129.0 (q, *J* = 32.3 Hz), 128.7, 125.2 (q, *J* = 3.7 Hz), 124.3 (q, *J* = 272.0 Hz), 96.7, 73.0, 71.3, 70.0, 69.7, 55.2, 37.7, 20.8, 20.7 ppm. **^{19}F NMR (376 MHz, $CDCl_3$)** δ -62.5 ppm. **HRMS (ESI) m/z [M+Na]$^+$:** ($C_{25}H_{25}F_3NaO_8$) calcd. 533.1394, found. 533.1400. **IR (neat)** 2943, 2848, 1748, 1619, 1452, 1370, 1325, 1220, 1162, 1119, 1026, 907, 851, 819, 710, 639, 598 cm^{-1}. **MP** 50.1-52.0 °C.

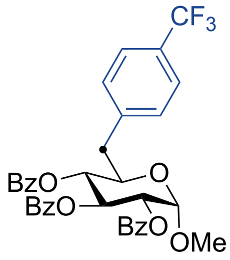

(2*S*,3*R*,4*S*,5*R*,6*R*)-2-methoxy-6-(4-(trifluoromethyl)benzyl)tetrahydro-2*H*-pyran-3,4,5-triyl tribenzoate (5b): Following general procedure D, using **3b** (117.7 mg, 0.24 mmol, 1.2 equivalents) and 1-bromo-4-(trifluoromethyl)benzene (**4a**, 28 µL, 0.2 mmol, 1 equivalent), the crude was purified by column chromatography (hexane:EtOAc, 12:1 to 10:1) to afford **5b** as a white solid (93.4 mg, 74% yield). In an independent experiment, 92.5 mg (73% yield) were obtained, giving an average yield of 73%. **^{1}H NMR (400 MHz, $CDCl_3$)** δ 8.00 – 7.94 (m, 4H), 7.90 – 7.85 (m, 2H), 7.56 – 7.48 (m, 4H), 7.44 – 7.34 (m, 7H), 7.32 – 7.27 (m, 2H), 6.14 (app t, *J* = 9.9 Hz, 1H), 5.49 (app t, *J* = 9.7 Hz, 1H), 5.27 (dd, *J* = 10.2, 3.7 Hz, 1H), 5.14 (d, *J* = 3.7 Hz, 1H), 4.28 (ddd, *J* = 9.9, 8.8, 3.7 Hz, 1H), 3.13 (s, 3H), 3.03 (dd, *J* = 14.2, 3.7 Hz, 1H), 2.97 (dd, *J* = 14.1, 8.7 Hz, 1H) ppm. **^{13}C NMR (101 MHz, $CDCl_3$)** δ 166.0, 165.9, 165.7, 141.7 (q, *J* = 1.2 Hz), 133.6, 133.5, 133.2, 130.0, 129.9, 129.8, 129.4, 129.2, 129.1, 129.0 (q, *J* = 32.1 Hz), 128.6, 128.5, 128.4, 125.2 (q, *J* = 3.7 Hz), 124.4 (q, *J* = 271.9 Hz), 96.9, 72.8, 72.3, 70.6, 69.9, 55.4, 37.8 ppm. **^{19}F NMR (376 MHz, $CDCl_3$)** δ -62.5 ppm. **HRMS (ESI) m/z [M+Na]$^{+}$:** ($C_{35}H_{29}F_3NaO_8$) calcd. 657.1707, found. 657.1687. **IR (neat)** 2959, 2896, 1720, 1602, 1451, 1325, 1261, 1160, 1092, 907, 849, 704, 643 cm^{-1}. **MP** 141.8-143.5 °C.

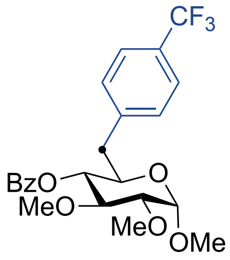

(2*R*,3*R*,4*S*,5*R*,6*S*)-4,5,6-trimethoxy-2-(4-(trifluoromethyl)benzyl)tetrahydro-2*H*-pyran-3-yl benzoate (5c): Following general procedure D, using **3c** (74.5 mg, 0.24 mmol, 1.2 equivalents) and 1-bromo-4-(trifluoromethyl)benzene (**4a**, 28 µL, 0.2 mmol, 1 equivalent), the crude was purified by column chromatography (hexane:EtOAc, 5:1) to afford **5c** as a colourless oil (62.0 mg, 68% yield). In an independent experiment, 61.6 mg (68% yield) were obtained, giving an average yield of 68%. **^{1}H NMR (400 MHz, $CDCl_3$)** δ 8.11 – 8.02 (m, 2H), 7.60 (ddt, *J* = 8.0, 6.9, 1.3 Hz, 1H), 7.52 – 7.43 (m, 4H), 7.31 (d, *J* = 8.0 Hz, 2H), 5.09 (dd, *J* = 10.0, 9.2 Hz, 1H), 4.78 (d, *J* = 3.6 Hz, 1H), 3.98 (td, *J* = 9.5, 3.4 Hz, 1H), 3.70 (app t, *J* = 9.4 Hz, 1H), 3.53 (s, 3H), 3.47 (s, 3H), 3.36 (dd, *J* = 9.6, 3.7 Hz, 1H), 3.10 (s, 3H), 2.90 (dd, *J* = 14.1, 3.4 Hz, 1H), 2.81 (dd, *J* = 14.1, 9.2 Hz, 1H) ppm. **^{13}C NMR (101 MHz, $CDCl_3$)** δ 165.7, 142.2, 142.2 (q, *J* = 1.1 Hz), 133.5, 129.9, 129.8, 129.8, 128.8 (q, *J* = 32.2 Hz), 128.6, 125.1 (q, *J* = 3.8 Hz), 124.4 (q, *J* = 271.7 Hz), 97.6, 81.7, 81.3, 74.6, 69.9, 61.1, 59.4, 55.2, 37.9 ppm. **^{19}F**

NMR (376 MHz, CDCl$_3$) δ -62.5 ppm. **HRMS (ESI) m/z [M+Na]$^+$:** ($C_{23}H_{25}F_3NaO_6$) calcd. 477.1495, found. 477.1481. **IR (neat)** 2932, 2837, 1724, 1618, 1602, 1452, 1324, 1264, 1159, 1103, 1064, 953, 851, 709, 647 cm^{-1}.

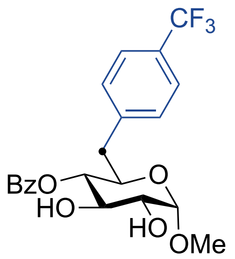

(2*R*,3*S*,4*R*,5*R*,6*S*)-4,5-dihydroxy-6-methoxy-2-(4-(trifluoromethyl)benzyl)tetrahydro-2*H*-pyran-3-yl benzoate (5d): Following general procedure D, using **3d** (56.5 mg, 0.2 mmol, 1.0 equivalent), 1-bromo-4-(trifluoromethyl)benzene (**4a**, 34 µL 0.24 mmol, 1.2 equivalents) and benzene:*tert*-amyl alcohol (1:1, 4.0 mL, 0.05 M), the crude was purified by column chromatography (DCM:MeOH, 40:1 to 30:1) to afford **5d** as a white solid (40.9 mg, 48% yield). In an independent experiment, 41.4 mg (49% yield) were obtained, giving an average yield of 48%. **^{1}H NMR (400 MHz, CDCl$_3$)** δ 8.06 – 8.01 (m, 2H), 7.60 (ddt, *J* = 8.8, 7.3, 1.3 Hz, 1H), 7.52 – 7.42 (m, 4H), 7.32 (d, *J* = 8.0 Hz, 2H), 5.02 (app t, *J* = 9.5 Hz, 1H), 4.72 (d, *J* = 3.7 Hz, 1H), 4.04 (td, *J* = 9.4, 3.3 Hz, 1H), 3.95 (app t, *J* = 9.3 Hz, 1H), 3.66 (dd, *J* = 9.4, 3.9 Hz, 1H), 3.13 (s, 3H), 3.04 – 2.70 (m, 3H), 2.47 (s, 1H) ppm. **^{13}C NMR (101 MHz, CDCl$_3$)** δ 166.5, 142.0 (q, *J* = 1.3 Hz), 133.7, 130.0, 129.8, 129.5, 129.5 – 128.3 (m), 128.7, 125.2 (q, *J* = 3.8 Hz), 124.4 (q, *J* = 271.9 Hz), 99.0, 75.0, 73.2, 73.1, 70.0, 55.4, 37.8 ppm. **^{19}F NMR (376 MHz, CDCl$_3$)** δ -62.5 ppm. **HRMS (ESI) m/z [M+Na]$^+$:** ($C_{21}H_{21}F_3NaO_6$) calcd. 449.1182, found. 449.1181. **IR (neat)** 3490, 3288, 2959, 2914, 2897, 1724, 1617, 1453, 1323, 1272, 1121, 852, 707 cm^{-1}. **MP** 119.0-120.9 °C.

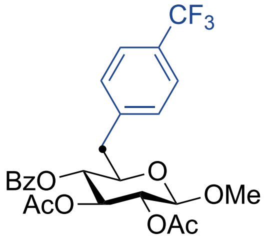

(2*R*,3*R*,4*S*,5*R*,6*R*)-5-(benzoyloxy)-2-methoxy-6-(4-(trifluoromethyl)benzyl)tetrahydro-2*H*-pyran-3,4-diyl diacetate (5e): Following general procedure D, using **3e** (87.9 mg, 0.24 mmol, 1.2 equivalents) and 1-bromo-4-(trifluoromethyl)benzene (**4a**, 28 µL, 0.2 mmol, 1 equivalent), the crude was purified by column chromatography (hexane:EtOAc, 8:1 to 6:1) to afford **5e** as a white solid (66.2 mg, 68% yield). In an independent experiment, 65.8 mg (64% yield) were obtained, giving an average yield of 65%. **^{1}H NMR (500 MHz, CDCl$_3$)** δ 8.00 – 7.93 (m, 2H), 7.60 (ddt, *J* = 8.7, 7.1, 1.3 Hz, 1H), 7.50 – 7.43 (m, 4H), 7.34 – 7.29 (m, 2H), 5.39 (app t, *J* = 9.6 Hz, 1H), 5.22 (app t, *J* = 9.6 Hz, 1H), 5.04 (dd, *J* = 9.8, 8.0 Hz, 1H), 4.37 (d, *J* = 7.9 Hz, 1H), 3.78 (ddd, *J* = 9.7, 7.3, 4.9 Hz, 1H), 3.37 (s, 3H), 3.01 – 2.92 (m, 2H), 2.04

(s, 3H), 1.89 (s, 3H) ppm. **^{13}C NMR (126 MHz, $CDCl_3$)** δ 170.3, 169.6, 165.5, 141.2 (q, *J* = 1.4 Hz), 133.8, 129.9, 129.1 (q, *J* = 32.3 Hz), 129.0, 128.7, 125.2 (q, *J* = 3.7 Hz), 124.3 (q, *J* = 271.8 Hz), 101.6, 74.3, 72.7, 72.7, 71.6, 56.9, 37.8, 20.8, 20.6 ppm. **^{19}F NMR (376 MHz, $CDCl_3$)** δ -62.5 ppm. **HRMS (ESI) m/z [M+Na]$^+$:** ($C_{25}H_{25}F_3NaO_8$) calcd. 533.1394, found. 533.1387. **IR (neat)** 2943, 2856, 1751, 1619, 1449, 1366, 1325, 1219, 1165, 1063, 900, 821, 721, 599 cm^{-1}. **MP** 109.9-113.0 °C.

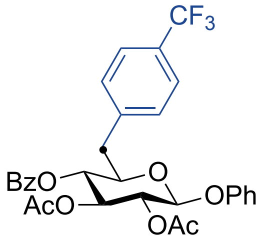

(2*S*,3*R*,4*S*,5*R*,6*R*)-5-(benzoyloxy)-2-phenoxy-6-(4-(trifluoromethyl)benzyl)tetrahydro-2*H*-pyran-3,4-diyl diacetate (5f): Following general procedure D, using **3f** (85.7 mg, 0.2 mmol, 1.0 equivalent), 1-bromo-4-(trifluoromethyl)benzene (**4a**, 56 µL, 0.4 mmol, 2.0 equivalents) and benzene:*tert*-amyl alcohol (1:1, 4.0 mL, 0.05 M), the crude was purified by column chromatography (hexane:EtOAc, 8:1 to 6:1) to afford **5f** as a white solid (79.6 mg, 70% yield). In an independent experiment, 80.0 mg (70% yield) were obtained, giving an average yield of 70%. **^{1}H NMR (400 MHz, $CDCl_3$)** δ 8.06 – 7.99 (m, 2H), 7.66 – 7.60 (m, 1H), 7.52 – 7.46 (m, 4H), 7.30 (d, *J* = 8.0 Hz, 2H), 7.11 – 7.05 (m, 2H), 7.02 – 6.96 (m, 1H), 6.58 – 6.52 (m, 2H), 5.49 (app t, *J* = 9.5 Hz, 1H), 5.34 (td, *J* = 9.6, 3.1 Hz, 2H), 4.91 (d, *J* = 7.9 Hz, 1H), 3.87 (td, *J* = 9.5, 3.5 Hz, 1H), 3.00 (dd, *J* = 14.1, 3.5 Hz, 1H), 2.94 (dd, *J* = 14.1, 9.3 Hz, 1H), 2.06 (s, 3H), 1.94 (s, 3H) ppm. **^{13}C NMR (101 MHz, $CDCl_3$)** δ 170.3, 169.5, 165.6, 156.8, 141.2 (q, *J* = 1.1 Hz), 133.9, 130.1, 130.0, 129.5, 129.3 (q, *J* = 32.2 Hz), 128.9, 128.8, 125.4 (q, *J* = 3.7 Hz), 124.4 (q, *J* = 271.9 Hz), 123.3, 116.8, 99.3, 75.3, 72.7, 72.6, 71.6, 37.8, 20.8, 20.7 ppm. **^{19}F NMR (376 MHz, $CDCl_3$)** δ -62.5 ppm. **HRMS (ESI) m/z [M+Na]$^+$:** ($C_{30}H_{27}F_3NaO_8$) calcd. 595.1550, found. 595.1538. **IR (neat)** 3085, 2958, 2874, 1747, 1602, 1495, 1370, 1325, 1220, 1121, 1068, 899, 821, 757, 707 cm^{-1}. **MP** 154.0-155.1 °C.

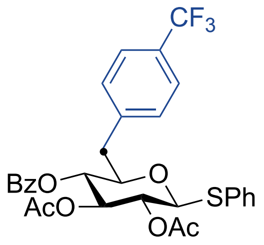

(2*S*,3*R*,4*S*,5*R*,6*R*)-5-(benzoyloxy)-2-(phenylthio)-6-(4-(trifluoromethyl)benzyl)tetrahydro-2*H*-pyran-3,4-diyl diacetate (5g): Following general procedure D, using **3g** (88.9 mg, 0.2 mmol, 1.0 equivalent), 1-bromo-4-(trifluoromethyl)benzene (**4a**, 56 µL, 0.4 mmol, 2.0 equivalents) and benzene:*tert*-amyl alcohol

(1:1, 4.0 mL, 0.05 M), the crude was purified by column chromatography (hexane:EtOAc, 8:1 to 6:1) to afford **5g** as a white solid (92.2 mg, 78% yield). In an independent experiment, 91.8 mg (78% yield) were obtained, giving an average yield of 78%. **^{1}H NMR (500 MHz, CDCl$_3$)** δ 8.02 – 7.96 (m, 2H), 7.63 – 7.58 (m, 1H), 7.50 – 7.45 (m, 4H), 7.29 (d, *J* = 8.0 Hz, 2H), 7.25 – 7.20 (m, 1H), 7.17 – 7.13 (m, 2H), 7.13 – 7.08 (m, 2H), 5.44 (app t, *J* = 9.4 Hz, 1H), 5.25 (app t, *J* = 9.6 Hz, 1H), 5.09 (dd, *J* = 10.1, 9.3 Hz, 1H), 4.68 (d, *J* = 10.1 Hz, 1H), 3.82 (ddd, *J* = 9.8, 8.5, 3.9 Hz, 1H), 3.00 – 2.90 (m, 2H), 2.09 (s, 3H), 1.90 (s, 3H) ppm. **^{13}C NMR (126 MHz, CDCl$_3$)** δ 170.2, 169.5, 165.5, 141.2 (q, *J* = 1.3 Hz), 133.9, 132.4, 132.2, 130.0, 129.9, 129.1 (d, *J* = 32.4 Hz), 129.0, 128.9, 128.8, 128.2, 125.3 (q, *J* = 3.8 Hz), 124.3 (q, *J* = 272.0 Hz), 86.4, 78.8, 73.8, 72.4, 70.4, 37.9, 20.9, 20.6 ppm. **^{19}F NMR (471 MHz, CDCl$_3$)** δ -62.3 ppm. **HRMS (ESI) m/z [M+Na]$^+$:** ($C_{30}H_{27}F_3NaO_7S$) calcd. 611.1322, found. 611.1320. **IR (neat)** 3084, 2943, 2880, 1737, 1585, 1479, 1439, 1373, 1325, 1231, 1125, 1023, 903, 828, 743, 704 cm^{-1}. **MP** 120.0-120.8 °C.

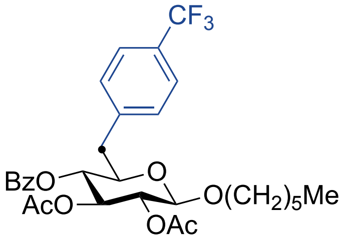

(2*R*,3*R*,4*S*,5*R*,6*R*)-5-(benzoyloxy)-2-(hexyloxy)-6-(4-(trifluoromethyl)benzyl)tetrahydro-2*H*-pyran-3,4-diyl diacetate (5h): Following general procedure D, using **3h** (104.8 mg, 0.24 mmol, 1.2 equivalents) and 1-bromo-4-(trifluoromethyl)benzene (**4a**, 28 μL, 0.2 mmol, 1 equivalent), the crude was purified by column chromatography (hexane:EtOAc, 10:1) to afford **5h** as a white solid (80.0 mg, 69% yield). In an independent experiment, 80.6 mg (69% yield) were obtained, giving an average yield of 69%. **^{1}H NMR (400 MHz, CDCl$_3$)** δ 7.96 (dd, *J* = 8.3, 1.4 Hz, 2H), 7.62 – 7.56 (m, 1H), 7.50 – 7.42 (m, 4H), 7.31 (d, *J* = 8.0 Hz, 2H), 5.39 (app t, *J* = 9.6 Hz, 1H), 5.22 (app t, *J* = 9.6 Hz, 1H), 5.04 (dd, *J* = 9.7, 8.0 Hz, 1H), 4.43 (d, *J* = 8.0 Hz, 1H), 3.77 (ddd, *J* = 9.7, 7.3, 4.9 Hz, 1H), 3.66 (dt, *J* = 9.7, 6.4 Hz, 1H), 3.35 (dt, *J* = 9.7, 6.7 Hz, 1H), 3.01 – 2.89 (m, 2H), 2.03 (s, 3H), 1.89 (s, 3H), 1.55 – 1.42 (m, 2H), 1.31 – 1.21 (m, 6H), 0.88 (t, *J* = 6.9 Hz, 3H) ppm. **^{13}C NMR (101 MHz, CDCl$_3$)** δ 170.4, 169.5, 165.5, 141.4 (q, *J* = 1.4 Hz), 133.8, 129.9, 129.9, 129.0 (q, *J* = 32.2 Hz), 129.0, 128.7, 125.2 (q, *J* = 3.7 Hz), 124.3 (q, *J* = 272.0 Hz), 100.9, 74.3, 72.8, 72.8, 71.8, 70.2, 37.9, 31.5, 29.4, 25.6, 22.7, 20.8, 20.7, 14.1 ppm. **^{19}F NMR (376 MHz, CDCl$_3$)** δ -62.6 ppm. **HRMS (ESI) m/z [M+Na]$^+$:** ($C_{30}H_{35}F_3NaO_8$) calcd. 603.2176, found. 603.2190. **IR (neat)** 2930, 2862, 1749, 1722, 1616, 1373, 1323, 1217, 1164, 1109, 1045, 896, 821, 710, 598 cm^{-1}. **MP** 97.9-101.0 °C.

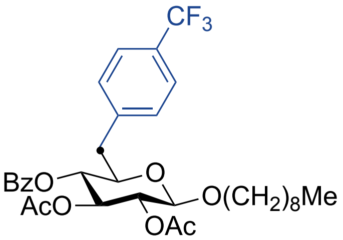

(2*R*,3*R*,4*S*,5*R*,6*R*)-5-(benzoyloxy)-2-(nonyloxy)-6-(4-(trifluoromethyl)benzyl)tetrahydro-2*H*-pyran-3,4-diyl diacetate (5i): Following general procedure D, using **3i** (114.9 mg, 0.24 mmol, 1.2 equivalents) and 1-bromo-4-(trifluoromethyl)benzene (**4a**, 28 µL, 0.2 mmol, 1 equivalent), the crude was purified by column chromatography (hexane:EtOAc, 10:1 to 8:1) to afford **5i** as a white solid (85.8 mg, 69% yield). In an independent experiment, 85.6 mg (69% yield) were obtained, giving an average yield of 69%. **^{1}H NMR (400 MHz, $CDCl_3$)** δ 8.00 – 7.93 (m, 2H), 7.59 (ddt, *J* = 8.7, 7.1, 1.3 Hz, 1H), 7.51 – 7.41 (m, 4H), 7.31 (d, *J* = 8.0 Hz, 2H), 5.39 (app t, *J* = 9.6 Hz, 1H), 5.22 (app t, *J* = 9.6 Hz, 1H), 5.04 (dd, *J* = 9.7, 8.0 Hz, 1H), 4.43 (d, *J* = 8.0 Hz, 1H), 3.77 (ddd, *J* = 9.7, 7.4, 4.9 Hz, 1H), 3.66 (dt, *J* = 9.6, 6.4 Hz, 1H), 3.35 (dt, *J* = 9.7, 6.7 Hz, 1H), 3.07 – 2.86 (m, 2H), 2.03 (s, 3H), 1.89 (s, 3H), 1.44 – 1.14 (m, 14H), 0.88 (t, *J* = 6.8 Hz, 3H) ppm. **^{13}C NMR (101 MHz, $CDCl_3$)** δ 170.4, 169.5, 165.5, 141.4 (q, *J* = 1.3 Hz), 133.8, 129.9, 129.0 (q, *J* = 32.2 Hz), 129.0, 128.7, 125.2 (q, *J* = 3.7 Hz), 124.3 (d, *J* = 271.9 Hz), 100.9, 74.3, 72.8, 72.8, 71.8, 70.2, 37.8, 32.0, 29.7, 29.5, 29.4, 29.4, 25.9, 22.8, 20.8, 20.6, 14.2 ppm. **^{19}F NMR (376 MHz, $CDCl_3$)** δ -62.5 ppm. **HRMS (ESI) m/z [M+Na]$^{+}$:** ($C_{33}H_{41}F_3NaO_8$) calcd. 645.2646, found. 645.2656. **IR (neat)** 2924, 2855, 1746, 1717, 1618, 1455, 1372, 1325, 1271, 1219, 1120, 1064, 901, 823, 712, 652, 600 cm^{-1}. **MP** 66.0-66.9 °C.

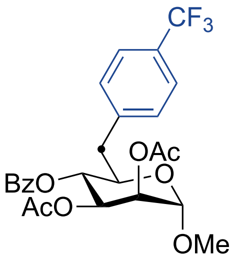

(2*S*,3*S*,4*S*,5*R*,6*R*)-5-(benzoyloxy)-2-methoxy-6-(4-(trifluoromethyl)benzyl)tetrahydro-2*H*-pyran-3,4-diyl diacetate (5j): Following general procedure D, using **3j** (87.9 mg, 0.24 mmol, 1.2 equivalents) and 1-bromo-4-(trifluoromethyl)benzene (**4a**, 28 µL, 0.2 mmol, 1 equivalent), the crude was purified by column chromatography (hexane:DCM, 1:2.5 to 1:2) to afford **5j** as a colourless oil (66.6 mg, 65% yield). In an independent experiment, 65.6 mg (64% yield) were obtained, giving an average yield of 65%. **^{1}H NMR (400 MHz, $CDCl_3$)** δ 8.04 – 7.94 (m, 2H), 7.64 – 7.55 (m, 1H), 7.53 – 7.41 (m, 4H), 7.35 (d, *J* = 8.0 Hz, 2H), 5.55 – 5.43 (m, 2H), 5.26 (dd, *J* = 3.1, 1.7 Hz, 1H), 4.63 (d, *J* = 1.7 Hz, 1H), 4.10 (ddd, *J* = 9.1, 7.8, 4.8

Hz, 1H), 3.08 (s, 3H), 3.00 – 2.89 (m, 2H), 2.17 (s, 3H), 1.88 (s, 3H) ppm. **^{13}C NMR (101 MHz, $CDCl_3$)** δ 170.2, 170.1, 165.9, 142.0 (q, *J* = 1.3 Hz), 133.7, 129.9, 129.8, 129.2, 128.9 (q, *J* = 32.5 Hz), 128.7, 125.2 (q, *J* = 3.8 Hz), 124.3 (q, *J* = 271.7 Hz), 98.6, 71.0, 70.5, 70.0, 69.2, 55.1, 37.8, 21.0, 20.7 ppm. **^{19}F NMR (376 MHz, $CDCl_3$)** δ -62.5 ppm. **HRMS (ESI) m/z [M+Na]$^+$:** ($C_{25}H_{25}F_3NaO_8$) calcd. 533.1394, found. 533.1386. **IR (neat)** 2936, 2858, 1750, 1727, 1619, 1452, 1370, 1323, 1266, 1218, 1112, 1065, 967, 906, 848, 710, 634 cm^{-1}.

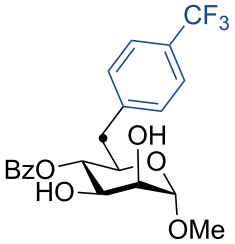

(2*R*,3*S*,4*R*,5*S*,6*S*)-4,5-dihydroxy-6-methoxy-2-(4-(trifluoromethyl)benzyl)tetrahydro-2*H*-pyran-3-yl benzoate (5k): Following general procedure D, using **3k** (56.5 mg, 0.2 mmol, 1.0 equivalent) and 1-bromo-4-(trifluoromethyl)benzene (**4a**, 56 µL, 0.4 mmol, 2.0 equivalents) and benzene:*tert*-amyl alcohol (1:1, 4.0 mL, 0.05 M), the crude was purified by column chromatography (hexane:EtOAc, 1:1) to afford **5k** as a white solid (34.1 mg, 40% yield). In an independent experiment, 34.7 mg (40% yield) were obtained, giving an average yield of 40%. **^{1}H NMR (400 MHz, $CDCl_3$)** ^{1}H NMR (400 MHz, $CDCl_3$) δ 8.05 – 7.99 (m, 2H), 7.60 (ddt, *J* = 7.8, 7.0, 1.3 Hz, 1H), 7.51 – 7.43 (m, 4H), 7.38 – 7.33 (m, 2H), 5.22 (app t, *J* = 9.6 Hz, 1H), 4.71 (d, *J* = 1.5 Hz, 1H), 4.07 – 3.97 (m, 3H), 3.36 (s, 1H), 3.10 (s, 1H), 3.05 (s, 3H), 3.01 – 2.96 (m, 1H), 2.91 (dd, *J* = 14.1, 9.0 Hz, 1H) ppm. **^{13}C NMR (101 MHz, $CDCl_3$)** δ 167.5, 142.3 (q, *J* = 1.4 Hz), 142.3, 133.8, 130.0, 129.8, 129.3, 128.8 (q, *J* = 32.3 Hz), 128.7, 125.2 (q, *J* = 3.8 Hz), 124.4 (q, *J* = 271.8 Hz), 100.5, 74.6, 70.9, 70.7, 70.3, 55.0, 38.0 ppm. **^{19}F NMR (376 MHz, $CDCl_3$)** δ -62.5 ppm. **HRMS (ESI) m/z [M+Na]$^+$:** ($C_{21}H_{21}F_3NaO_6$) calcd. 449.1182, found. 449.1172. **IR (neat) 3511, 3407, 2908, 1723, 1330, 1263, 1112, 1065, 1016, 845, 710** cm^{-1}. **MP** 88.6-89.4 °C.

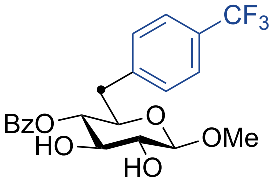

(2*R*,3*S*,4*R*,5*R*,6*R*)-4,5-dihydroxy-6-methoxy-2-(4-(trifluoromethyl)benzyl)tetrahydro-2*H*-pyran-3-yl benzoate (5l): Following general procedure D, using **3l** (56.5 mg, 0.2 mmol, 1.0 equivalent) and 1-bromo-4-(trifluoromethyl)benzene (**4a**, 34 µL, 0.24 mmol, 1.2 equivalents), the crude was purified by column chromatography (hexane:EtOAc, 1:1) to afford **5l** as a white solid (35.7 mg, 42% yield). In an independent experiment, 35.9 mg (42% yield) were obtained, giving an average yield of 42%. **^{1}H NMR (500 MHz, $CDCl_3$)** δ 8.07 – 8.01 (m, 2H), 7.63 – 7.56 (m, 1H), 7.50 – 7.43 (m, 4H), 7.31 (d, *J* = 8.0 Hz, 2H), 5.02 (app t, *J* = 9.6 Hz, 1H), 4.72 (d, *J* = 3.9 Hz, 1H), 4.04 (td, *J* = 9.5, 3.3 Hz, 1H), 3.95 (app t, *J* = 9.3 Hz, 1H), 3.66 (dd, *J* = 9.5, 3.9 Hz, 1H), 3.12 (s, 3H), 2.95 (dd, *J* = 14.2, 3.3 Hz, 1H), 2.84 (dd, *J* = 14.2, 9.1

Hz, 1H), 2.53 (s, 1H) ppm. **^{13}C NMR (126 MHz, $CDCl_3$)** δ 166.5, 142.0 (q, J = 1.4 Hz), 133.7, 130.0, 129.8, 129.5, 128.9 (q, J = 32.4 Hz), 128.6, 125.2 (q, J = 3.7 Hz), 125.2, 125.1, 124.3 (q, J = 271.7 Hz), 98.9, 75.0, 73.2, 73.0, 70.0, 55.4, 37.8 ppm. **^{19}F NMR (376 MHz, $CDCl_3$)** δ -62.5 ppm. **HRMS (ESI) m/z [M+Na]$^+$:** ($C_{21}H_{21}F_3NaO_6$) calcd. 449.1182, found. 449.1175. **IR (neat)** 3485, 3279, 2915, 1723, 1453, 1323, 1272, 1160, 1121, 1066, 1038, 989, 852, 707 cm^{-1}. **MP** 128.8-129.8 °C.

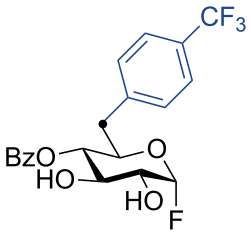

(2*R*,3*S*,4*R*,5*R*,6*R*)-6-fluoro-4,5-dihydroxy-2-(4-(trifluoromethyl)benzyl)tetrahydro-2*H*-pyran-3-yl benzoate (5m): Following general procedure D, using **3m** (54.1 mg, 0.2 mmol, 1.0 equivalent) and 1-bromo-4-(trifluoromethyl)benzene (**4a**, 56 µL, 0.4 mmol, 2.0 equivalents) and benzene:*tert*-amyl alcohol (1:1, 4.0 mL, 0.05 M), the crude was purified by column chromatography (hexane:EtOAc, 1:1) to afford **5m** as a white solid (24.6 mg, 32% yield). In an independent experiment, 24.7 mg (32% yield) were obtained, giving an average yield of 32%. **^{1}H NMR (400 MHz, $CDCl_3$)** ^{1}H NMR (400 MHz, $CDCl_3$) δ 8.04 – 7.94 (m, 2H), 7.65 – 7.56 (m, 1H), 7.51 – 7.40 (m, 4H), 7.28 (d, J = 7.9 Hz, 2H), 5.61 (dd, J = 53.2, 2.8 Hz, 1H), 5.04 (app t, J = 9.7 Hz, 1H), 4.35 (ddd, J = 10.0, 7.6, 4.1 Hz, 1H), 4.02 (app t, J = 9.5 Hz, 1H), 3.69 (ddd, J = 24.8, 9.7, 2.7 Hz, 1H), 3.18 (s, 1H), 3.00 (dd, J = 14.7, 4.2 Hz, 1H), 2.93 (dd, J = 14.7, 7.5 Hz, 1H) ppm. **^{13}C NMR (101 MHz, $CDCl_3$)** δ 166.5, 140.7 (q, J = 1.5 Hz), 140.7, 133.9, 129.9, 129.8, 129.1 (q, J = 32.3 Hz), 129.0, 128.7, 125.4 (q, J = 3.8 Hz), 124.3 (q, J = 271.8 Hz), 107.5, 105.3, 73.9, 72.8, 72.5, 72.4, 71.9, 37.6 ppm. **^{19}F NMR (376 MHz, $CDCl_3$)** δ -62.6, -151.4 ppm. **HRMS (ESI) m/z [M+Na]$^+$:** ($C_{20}H_{18}F_4NaO_5$) calcd. 437.0983, found. 437.0972. **IR (neat)** 3450, 3260, 1727, 1328, 1259, 1162, **1111**, 1067, 1019, 987, 879, 808, 752, 709 cm^{-1}. **MP** 126.7-127.4 °C.

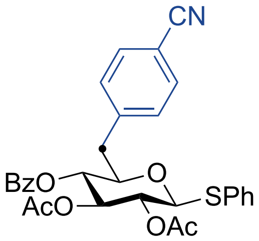

(2*S*,3*R*,4*S*,5*R*,6*R*)-5-(benzoyloxy)-6-(4-cyanobenzyl)-2-(phenylthio)tetrahydro-2*H*-pyran-3,4-diyl diacetate (5n): Following general procedure D, using **3n** (88.9 mg, 0.2 mmol, 1.0 equivalent), methyl 4-bromobenzonitrile (**4b**, 72.8 mg, 0.4 mmol, 2.0 equivalents) and benzene:*tert*-amyl alcohol (1:1, 4.0 mL, 0.05 M), the crude was purified by column chromatography (hexane:EtOAc, 5:1 to 4:1) to afford **5n** as a white solid (65.6 mg, 60% yield). In an independent experiment, 64.6 mg (59% yield) were obtained, giving an average yield of

60%. **^{1}H NMR (400 MHz, CDCl$_3$)** δ 8.00 – 7.93 (m, 2H), 7.64 – 7.58 (m, 1H), 7.52 – 7.43 (m, 4H), 7.30 – 7.24 (m, 3H), 7.20 – 7.12 (m, 4H), 5.43 (app t, *J* = 9.4 Hz, 1H), 5.21 (app t, *J* = 9.6 Hz, 1H), 5.06 (dd, *J* = 10.1, 9.3 Hz, 1H), 4.69 (d, *J* = 10.1 Hz, 1H), 3.80 (ddd, *J* = 9.8, 7.6, 4.7 Hz, 1H), 2.98 – 2.89 (m, 2H), 2.08 (s, 3H), 1.89 (s, 3H) ppm. **^{13}C NMR (101 MHz, CDCl$_3$)** δ 170.2, 169.5, 165.4, 142.7, 133.9, 132.4, 132.2, 132.1, 130.4, 129.9, 129.0, 128.8, 128.3, 118.9, 110.7, 86.3, 78.5, 73.7, 72.3, 70.3, 38.1, 20.9, 20.6 ppm. **HRMS (ESI) m/z [M+Na]$^+$:** ($C_{30}H_{27}NNaO_7S$) calcd. 568.1400, found. 568.1399. **IR (neat)** 3074, 2947, 2884, 2229, 1744, 1717, 1604, 1373, 1266, 1218, 1046, 896, 830, 745, 710, 600 cm^{-1}. **MP** 149.0-153.1 °C.

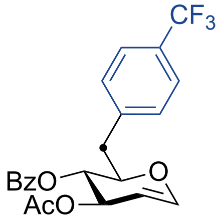

(2*R*,3*R*,4*R*)-4-acetoxy-2-(4-(trifluoromethyl)benzyl)-3,4-dihydro-2*H*-pyran-3-yl benzoate (5o): Following general procedure D, using **3o** (66.3 mg, 0.24 mmol, 1.2 equivalents), 1-bromo-4-(trifluoromethyl)benzene (**4a**, 28 μL, 0.2 mmol, 1 equivalent) and benzene:*tert*-amyl alcohol (1:1, 4.0 mL, 0.05 M), the crude was purified by column chromatography (hexane:DCM, 2:1 to 1:1) to afford **5o** as a colourless oil (32.5 mg, 39% yield). In an independent experiment, 31.9 mg (38% yield) were obtained, giving an average yield of 39%. **^{1}H NMR (500 MHz, CDCl$_3$)** δ 8.02 – 7.98 (m, 2H), 7.62 – 7.57 (m, 1H), 7.55 – 7.50 (m, 2H), 7.48 – 7.43 (m, 2H), 7.36 – 7.30 (m, 2H), 6.49 (dd, *J* = 6.2, 1.4 Hz, 1H), 5.53 (dddd, *J* = 5.5, 3.2, 1.4, 0.8 Hz, 1H), 5.38 (dd, *J* = 7.5, 5.6 Hz, 1H), 4.92 (dd, *J* = 6.2, 3.2 Hz, 1H), 4.43 (dddd, *J* = 8.5, 7.4, 4.4, 0.8 Hz, 1H), 3.13 (dd, *J* = 14.5, 8.7 Hz, 1H), 3.06 (dd, *J* = 14.5, 4.4 Hz, 1H), 2.04 (s, 3H) ppm. **^{13}C NMR (126 MHz, CDCl$_3$)** δ 170.5, 165.5, 145.8, 141.2 (q, *J* = 1.2 Hz), 133.7, 129.9, 129.8, 129.3, 129.2 (q, *J* = 32.2 Hz), 128.7, 125.5 (q, *J* = 3.8 Hz), 124.3 (q, *J* = 272.0 Hz), 99.2, 76.4, 70.9, 67.9, 36.6, 21.2 ppm. **^{19}F NMR (471 MHz, CDCl$_3$)** δ -62.5 ppm. **HRMS (ESI) m/z [M+Na]$^+$:** ($C_{22}H_{19}F_3NaO_5$) calcd. 443.1077, found. 443.1082. **IR (film)** 2959, 2931, 1725, 1648, 1619, 1452, 1371, 1324, 1265, 1223, 1162, 1109, 1066, 1018, 962, 849, 709, 643 cm^{-1}.

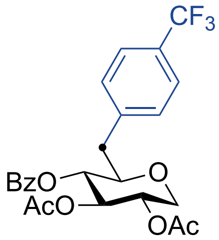

(3*S*,4*R*,5*R*,6*R*)-5-(benzoyloxy)-6-(4-(trifluoromethyl)benzyl)tetrahydro-2*H*-pyran-3,4-diyl diacetate (5p): Following general procedure D, using **3p** (80.7 mg, 0.24 mmol, 1.2 equivalents) and 1-bromo-4-(trifluoromethyl)benzene (**4a**, 28 μL, 0.2 mmol, 1 equivalent), the crude was purified by column chromatography (hexane:EtOAc, 10:1 to 6:1) to afford **5p** as a

white solid (62.1 mg, 65% yield). In an independent experiment, 61.9 mg (64% yield) were obtained, giving an average yield of 65%. **^{1}H NMR (400 MHz, $CDCl_3$)** δ 8.01 – 7.93 (m, 2H), 7.59 (ddt, *J* = 8.8, 7.2, 1.3 Hz, 1H), 7.50 – 7.41 (m, 4H), 7.27 (d, *J* = 8.3 Hz, 2H), 5.38 (app t, *J* = 9.5 Hz, 1H), 5.16 (app t, *J* = 9.6 Hz, 1H), 5.04 (ddd, *J* = 10.5, 9.6, 5.6 Hz, 1H), 4.12 (dd, *J* = 11.2, 5.7 Hz, 1H), 3.73 (ddd, *J* = 9.7, 8.0, 3.9 Hz, 1H), 3.30 – 3.22 (m, 1H), 2.93 (dd, *J* = 14.5, 3.9 Hz, 1H), 2.87 (dd, *J* = 14.5, 8.0 Hz, 1H), 2.02 (s, 3H), 1.90 (s, 3H) ppm. **^{13}C NMR (101 MHz, $CDCl_3$)** δ 170.4, 170.0, 165.6, 141.3 (q, *J* = 1.3 Hz), 133.8, 129.9, 129.9, 129.1, 129.0 (q, *J* = 32.0 Hz), 128.7, 125.3 (q, *J* = 3.8 Hz), 124.3 (q, *J* = 271.8 Hz), 79.1, 73.7, 72.8, 69.4, 67.0, 38.1, 20.8, 20.7 ppm. **^{19}F NMR (376 MHz, $CDCl_3$)** δ -62.5 ppm. **HRMS (ESI) m/z [M+Na]$^{+}$:** ($C_{24}H_{23}F_3NaO_7$) calcd. 503.1288, found. 503.1290. **IR (neat)** 2946, 2863, 1719, 1618, 1451, 1367, 1323, 1235, 1155, 1108, 898, 850, 809, 707, 643, 597 cm^{-1}. **MP** 102.4-106.1 °C.

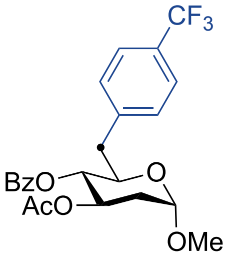

(2*R*,3*R*,4*R*,6*S*)-4-acetoxy-6-methoxy-2-(4-(trifluoromethyl)benzyl)tetrahydro-2*H*-pyran-3-yl benzoate (5q): Following general procedure D, using **3q** (74.0 mg, 0.24 mmol, 1.2 equivalents) and 1-bromo-4-(trifluoromethyl)benzene (**4a**, 28 μL, 0.2 mmol, 1 equivalent), the crude was purified by column chromatography (hexane:EtOAc, 10:1 to 8:1) to afford **5q** as a white solid (51.5 mg, 57% yield). In an independent experiment, 50.8 mg (56% yield) were obtained, giving an average yield of 57%. **^{1}H NMR (400 MHz, $CDCl_3$)** δ 8.04 – 7.97 (m, 2H), 7.59 (ddt, *J* = 8.8, 7.1, 1.3 Hz, 1H), 7.51 – 7.41 (m, 4H), 7.32 (d, *J* = 8.0 Hz, 2H), 5.47 (ddd, *J* = 11.6, 9.4, 5.3 Hz, 1H), 5.14 (app t, *J* = 9.6 Hz, 1H), 4.76 (dd, *J* = 3.8, 1.2 Hz, 1H), 4.09 (td, *J* = 9.4, 3.4 Hz, 1H), 3.02 (s, 3H), 2.95 (dd, *J* = 14.2, 3.3 Hz, 1H), 2.85 (dd, *J* = 14.1, 9.1 Hz, 1H), 2.27 (ddd, *J* = 12.9, 5.3, 1.3 Hz, 1H), 1.90 (s, 3H), 1.89 – 1.81 (m, 1H) ppm. **^{13}C NMR (101 MHz, $CDCl_3$)** δ 170.3, 166.0, 142.1 (q, *J* = 1.3 Hz), 133.6, 129.9, 129.8, 129.5, 128.8 (d, *J* = 33.0 Hz), 128.7, 125.1 (q, *J* = 3.8 Hz), 124.4 (d, *J* = 271.9 Hz), 97.9, 74.0, 70.3, 69.1, 54.7, 38.0, 35.4, 21.0 ppm. **^{19}F NMR (376 MHz, $CDCl_3$)** δ -62.5 ppm. **HRMS (ESI) m/z [M+Na]$^{+}$:** ($C_{23}H_{23}F_3NaO_6$) calcd. 475.1339, found. 475.1354. **IR (neat)** 2954, 2926, 2851, 1716, 1618, 1452, 1325, 1239, 1112, 1066, 1027, 963, 843, 708, 636 cm^{-1}. **MP** 71.9-73.2 °C.

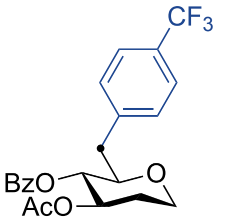

(2*R*,3*R*,4*R*)-4-acetoxy-2-(4-(trifluoromethyl)benzyl)tetrahydro-2*H*-pyran-3-yl benzoate (5r): Following general procedure D, using **3r** (66.8 mg, 0.24 mmol, 1.2 equivalents) and 1-bromo-4-(trifluoromethyl)benzene (**4a**, 28 µL, 0.2 mmol, 1 equivalent), the crude was purified by column chromatography (hexane:EtOAc, 9:1) to afford **5r** as a white solid (48.2 mg, 57% yield). In an independent experiment, 44.6 mg (53% yield) were obtained, giving an average yield of 55%. **^{1}H NMR (400 MHz, CDCl3)** δ 8.03 – 7.97 (m, 2H), 761 – 7.56 (m, 1H), 7.48 – 7.43 (m, 4H), 7.30 – 7.2 (d, *J* = 8.0 Hz, 2H), 5.19 – 5.09 (m, 2H), 3.99 (ddd, *J* = 12.0, 5.1, 1.6 Hz, 1H), 3.69 (td, *J* = 8.5, 3.6 Hz, 1H), 3.46 (td, *J* = 12.4, 2.1 Hz, 1H), 2.94 (dd, *J* = 14.5, 3.7 Hz, 1H), 2.87 (dd, *J* = 14.5, 8.2 Hz, 1H), 2.15 – 2.10 (m, 1H), 1.92 (s, 3H), 1.91 – 1.82 (m, 1H) ppm. **^{13}C NMR (101 MHz, CDCl3)** δ 170.6, 165.9, 141.9, 133.6, 129.8, 129.7, 129.5, 128.8 (q, *J* = 32.5 Hz), 128.7, 125.2 (q, *J* = 3.7 Hz), 124.4 (q, *J* = 272.4 Hz), 79.2, 73.9, 72.5, 65.5, 38.5, 31.5, 21.1 ppm. **^{19}F NMR (376 MHz, CDCl3)** δ -62.5 ppm. **IR (neat)** 2959, 2926, 2855, 1740, 1713, 1601, 1418, 1322, 1235, 1118, 1065, 845 cm^{-1}. **HRMS (ESI) m/z [M+Na]$^{+}$:** ($C_{22}H_{21}F_{3}NaO_{5}$) calcd. 445.1233, found: 445.1243. **MP** 84 °C.

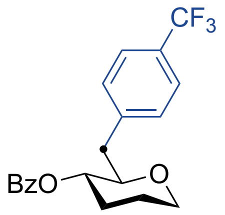

(2*R*,3*S*)-2-(4-(trifluoromethyl)benzyl)tetrahydro-2*H*-pyran-3-yl benzoate (5s): Following general procedure D, using **4a** (28 µL, 0.2 mmol, 1 equivalent), the crude was purified by column chromatography (hexane:EtOAc, 30:1) to afford **5s** as a white solid (31.5 mg, 43% yield). In an independent experiment, 30.6 mg (42% yield) were obtained, giving an average yield of 43%. **^{1}H NMR (400 MHz, $CDCl_3$)** δ 8.00 – 7.93 (m, 2H), 7.58 (ddt, *J* = 8.7, 7.0, 1.3 Hz, 1H), 7.51 – 7.41 (m, 4H), 7.32 (d, *J* = 8.0 Hz, 2H), 4.83 (ddd, *J* = 10.6, 9.3, 4.7 Hz, 1H), 3.99 – 3.91 (m, 1H), 3.68 (ddd, *J* = 9.3, 8.1, 3.6 Hz, 1H), 3.37 (td, *J* = 11.7, 2.6 Hz, 1H), 3.03 (dd, *J* = 14.4, 3.6 Hz, 1H), 2.85 (dd, *J* = 14.4, 8.1 Hz, 1H), 2.39 – 2.30 (m, 1H), 1.86 – 1.76 (m, 1H), 1.76 – 1.70 (m, 1H), 1.65 – 1.57 (m, 1H) ppm. **^{13}C NMR (101 MHz, $CDCl_3$)** δ 165.7, 142.7 (q, *J* = 1.2 Hz), 133.3, 130.2, 129.8, 129.7, 128.6 (q, *J* = 32.2 Hz), 128.6, 125.2 (q, *J* = 3.7 Hz), 124.5 (d, *J* = 271.8 Hz), 80.2, 72.6, 68.1, 38.9, 29.7, 25.3 ppm. **^{19}F NMR (376 MHz, $CDCl_3$)** δ -62.4 ppm. **HRMS (ESI) m/z [M+Na]$^{+}$:** ($C_{20}H_{19}F_{3}NaO_{3}$) calcd. 387.1178, found. 387.1175. **IR (neat)** 2922, 2855, 1715, 1617, 1451, 1327, 1269, 1065, 1096, 1019, 948, 843, 798, 722, 636 cm^{-1}. **MP** 78.8-80.9 °C.

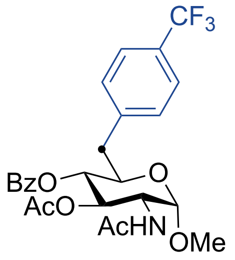

(2*R*,3*R*,4*R*,5*R*,6*S*)-5-acetamido-4-acetoxy-6-methoxy-2-(4-(trifluoromethyl)benzyl)tetrahydro-2*H*-pyran-3-yl benzoate (5t): Following general procedure D, using **3t** (87.7 mg, 0.24 mmol, 1.2 equivalents), 1-bromo-4-(trifluoromethyl)benzene (**4a**, 28 µL, 0.2 mmol, 1 equivalent) and benzene:*tert*-amyl alcohol (1:1, 4.0 mL, 0.05 M), the crude was purified by column chromatography (hexane:acetone, 3:1) to afford **5t** as a white solid (73.4 mg, 72% yield). In an independent experiment, 69.4 mg (68% yield) were obtained, giving an average yield of 70%. **^{1}H NMR (400 MHz, CDCl$_3$)** δ 8.02 – 7.93 (m, 2H), 7.58 (ddt, *J* = 8.8, 7.3, 1.3 Hz, 1H), 7.49 – 7.40 (m, 4H), 7.30 (d, *J* = 8.0 Hz, 2H), 5.77 (d, *J* = 9.5 Hz, 1H), 5.36 (dd, *J* = 10.7, 9.5 Hz, 1H), 5.23 (app t, *J* = 9.7 Hz, 1H), 4.65 (d, *J* = 3.6 Hz, 1H), 4.38 (ddd, *J* = 10.7, 9.5, 3.7 Hz, 1H), 4.04 (td, *J* = 9.3, 3.6 Hz, 1H), 3.05 (s, 3H), 2.93 (dd, *J* = 14.1, 3.6 Hz, 1H), 2.86 (dd, *J* = 14.2, 8.8 Hz, 1H), 1.93 (s, 3H), 1.87 (s, 3H) ppm. **^{13}C NMR (101 MHz, CDCl$_3$)** δ 171.4, 170.0, 165.4, 141.7 (q, *J* = 1.2 Hz), 133.7, 129.8, 129.1, 128.9 (q, *J* = 32.3 Hz), 128.7, 125.1 (q, *J* = 3.8 Hz), 124.3 (q, *J* = 271.9 Hz), 98.1, 72.6, 71.4, 70.1, 55.1, 52.2, 37.7, 23.3, 20.7 ppm. **^{19}F NMR (376 MHz, CDCl$_3$)** δ -62.5 ppm. **HRMS (ESI) m/z [M+Na]$^+$:** ($C_{25}H_{26}F_3NNaO_7$) calcd. 532.1554, found. 532.1554. **IR (neat)** 3293, 2935, 1747, 1656, 1549, 1324, 1266, 1227, 1165, 1112, 1028, 852, 708 cm^{-1}. **MP** 126.8-128.5 °C.

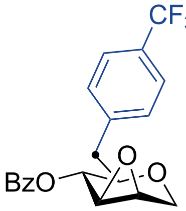

(1*R*,4*R*,5*R*,6*R*)-4-(4-(trifluoromethyl)benzyl)-3,7-dioxabicyclo[4.1.0]heptan-5-yl benzoate (5u): Following general procedure D, using **3u** (56.2 mg, 0.24 mmol, 1.2 equivalents) and 1-bromo-4-(trifluoromethyl)benzene (**4a**, 28 µL, 0.2 mmol, 1 equivalent), the crude was purified by column chromatography (hexane:acetone, 10:1) to afford **5u** as a colourless oil (36.3 mg, 48% yield). In an independent experiment, 33.3 mg (44% yield) were obtained, giving an average yield of 46%. **^{1}H NMR (400 MHz, CDCl$_3$)** δ 8.03 – 7.94 (m, 2H), 7.64 – 7.58 (m, 1H), 7.49 – 7.43 (m, 4H), 7.31 (d, *J* = 8.0 Hz, 2H), 5.04 (d, *J* = 9.3 Hz, 1H), 4.23 (d, *J* = 13.5 Hz, 1H), 3.85 (dd, *J* = 13.5, 0.8 Hz, 1H), 3.52 (ddd, *J* = 9.3, 7.8, 4.4 Hz, 1H), 3.34 (d, *J* = 3.7 Hz, 1H), 3.24 – 3.20 (m, 1H), 2.97 – 2.85 (m, 2H) ppm. **^{13}C NMR (101 MHz, CDCl$_3$)** δ 165.4, 141.7 (q, *J* = 1.2 Hz), 133.8, 129.8, 129.3, 128.9 (q, *J* = 32.4 Hz), 128.7, 125.3 (q, *J* = 3.7 Hz), 124.3 (d, *J* = 271.9 Hz), 76.2, 68.0, 64.9, 53.4, 49.4, 39.4 ppm. **^{19}F NMR (376 MHz, CDCl$_3$)** δ -62.5 ppm. **HRMS (ESI) m/z [M+Na]$^+$:** ($C_{20}H_{17}F_3NaO_4$) calcd. 401.0971, found. 401.0966. **IR (neat)** 3462, 2923, 2857, 1720, 1602, 1452, 1323, 1265, 1160, 1109, 1065, 1018, 960, 840, 709, 634 cm^{-1}.

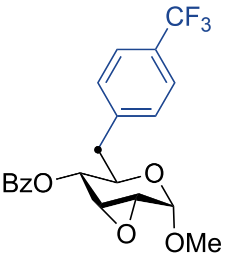

(1*R*,2*S*,4*R*,5*R*,6*R*)-2-methoxy-4-(4-(trifluoromethyl)benzyl)-3,7-dioxabicyclo[4.1.0]heptan-5-yl benzoate (5v): Following general procedure D, using **3v** (63.4 mg, 0.24 mmol, 1.2 equivalents) and 1-bromo-4-(trifluoromethyl)benzene (**4a**, 28 µL, 0.2 mmol, 1 equivalent), the crude was purified by column chromatography (hexane: EtOAc, 10:1 to 6:1) to afford **5v** as a colourless oil (55.5 mg, 68% yield). In an independent experiment, 51.8 mg (63% yield) were obtained, giving an average yield of 66%. **^{1}H NMR (400 MHz, CDCl$_3$)** δ 8.08 – 8.02 (m, 2H), 7.61 (ddt, *J* = 8.8, 7.1, 1.3 Hz, 1H), 7.53 – 7.43 (m, 4H), 7.32 (d, *J* = 8.0 Hz, 2H), 5.20 (dd, *J* = 9.6, 1.7 Hz, 1H), 4.85 (d, *J* = 3.1 Hz, 1H), 4.25 (td, *J* = 9.4, 3.2 Hz, 1H), 3.64 (dd, *J* = 4.2, 1.7 Hz, 1H), 3.56 (dd, *J* = 4.2, 3.1 Hz, 1H), 3.05 (s, 3H), 3.01 (dd, *J* = 13.9, 3.4 Hz, 1H), 2.76 (dd, *J* = 14.1, 9.4 Hz, 1H) ppm. **^{13}C NMR (101 MHz, CDCl$_3$)** δ 166.1, 142.0 (q, *J* = 1.2 Hz), 133.7, 129.9, 129.4, 128.9 (q, *J* = 32.3 Hz), 128.7, 125.2 (q, *J* = 3.8 Hz), 124.3 (q, *J* = 271.8 Hz), 94.6, 71.5, 66.8, 55.5, 55.0, 51.7, 37.8 ppm. **^{19}F NMR (376 MHz, CDCl$_3$)** δ -62.5 ppm. **HRMS (ESI) m/z [M+Na]$^+$:** ($C_{21}H_{19}F_3NaO_5$) calcd. 431.1077, found. 431.1089. **IR (neat)** 2930, 2834, 1720, 1618, 1452, 1323, 1266, 1162, 1108, 1065, 1025, 985, 903, 852, 710, 687 cm^{-1}.

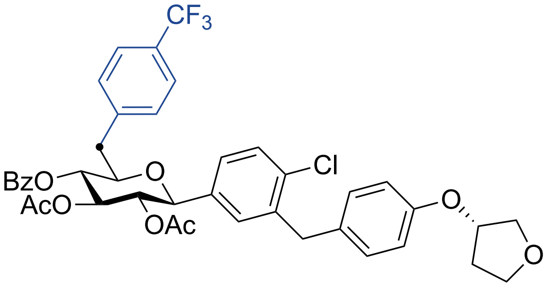

(2*S*,3*S*,4*S*,5*R*,6*R*)-5-(benzoyloxy)-2-(4-chloro-3-(4-(((*S*)-tetrahydrofuran-3-yl)oxy)benzyl)phenyl)-6-(4-(trifluoromethyl)benzyl)tetrahydro-2*H*-pyran-3,4-diyl diacetate (5w): Following general procedure D, using **3w** (149.5 mg, 0.24 mmol, 1.2 equivalents), 1-bromo-4-(trifluoromethyl)benzene (**4a**, 28 µL, 0.2 mmol, 1 equivalent) and benzene:*tert*-amyl alcohol (1:1, 4.0 mL, 0.05 M), the crude was purified by column chromatography (hexane:acetone, 8:1) to afford **5w** as a white solid (72.1 mg, 47% yield). In an independent experiment, 65.9 mg (43% yield) were obtained, giving an average yield of 45%. **^{1}H NMR (400 MHz, CDCl$_3$)** δ 8.01 – 7.94 (m, 2H), 7.61 (ddt, *J* = 8.7, 7.1, 1.3 Hz, 1H), 7.49 – 7.44 (m, 2H), 7.43 – 7.38 (m, 2H), 7.36 (d, *J* = 8.2 Hz, 1H), 7.23 (d, *J* = 8.0 Hz, 2H), 7.15 (dd, *J* = 8.3, 2.2 Hz, 1H), 7.11 – 7.06 (m, 3H), 6.80 (d, *J* = 8.6 Hz, 2H), 5.46 (app t, *J* = 9.5 Hz, 1H), 5.30 (app t, *J* = 9.6 Hz, 1H), 5.04 (app t, *J* = 9.6 Hz, 1H), 4.92 – 4.87 (m, 1H),

4.32 (d, *J* = 9.8 Hz, 1H), 4.09 (d, *J* = 15.5 Hz, 1H), 4.02 – 3.87 (m, 6H), 3.04 – 2.91 (m, 2H), 2.20 – 2.12 (m, 2H), 1.87 (s, 3H), 1.73 (s, 3H) ppm. **^{13}C NMR (101 MHz, $CDCl_3$)** δ 170.4, 168.9, 165.5, 156.1, 141.0 (q, *J* = 1.3 Hz), 139.1, 135.7, 134.5, 133.8, 131.8, 130.1, 130.1, 129.9, 129.8, 129.6, 129.0, 129.0 (q, *J* = 32.4 Hz), 128.7, 126.0, 125.2 (q, *J* = 3.8 Hz), 124.3 (q, *J* = 271.8 Hz), 115.5, 79.3, 78.5, 74.1, 73.2, 73.0, 72.5, 67.3, 38.4, 38.1, 33.1, 20.7, 20.5 ppm. **^{19}F NMR (376 MHz, $CDCl_3$)** δ -62.5 ppm. **HRMS (ESI) m/z [M+Na]$^+$:** ($C_{41}H_{38}ClF_3NaO_9$) calcd. 789.2049, found. 789.2039. **IR (neat)** 2944, 2855, 1751, 1720, 1618, 1582, 1511, 1477, 1368, 1325, 1229, 1162, 1062, 833, 706 cm^{-1}. **MP** 107.9-110.0 °C.

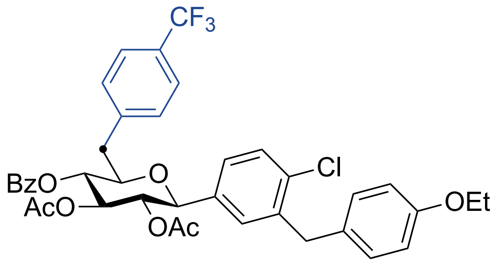

(2*S*,3*S*,4*S*,5*R*,6*R*)-5-(benzoyloxy)-2-(4-chloro-3-(4-ethoxybenzyl)phenyl)-6-(4-(trifluoromethyl)benzyl)tetrahydro-2*H*-pyran-3,4-diyl diacetate (5x): Following general procedure D, using **3x** (139.5 mg, 0.24 mmol, 1.2 equivalents), 1-bromo-4-(trifluoromethyl)benzene (**4a**, 28 μL, 0.2 mmol, 1 equivalent) and benzene:*tert*-amyl alcohol (1:1, 4.0 mL, 0.05 M), the crude was purified by column chromatography (hexane:$CHCl_3$, 1:3) to afford **5x** as a white solid (62.2 mg, 43% yield). In an independent experiment, 61.9 mg (43% yield) were obtained, giving an average yield of 43%. **^{1}H NMR (400 MHz, $CDCl_3$)** δ 8.05 – 7.95 (m, 2H), 7.68 – 7.59 (m, 1H), 7.49 (t, *J* = 7.8 Hz, 2H), 7.43 (d, *J* = 8.0 Hz, 2H), 7.38 (d, *J* = 8.2 Hz, 1H), 7.25 (d, *J* = 8.0 Hz, 2H), 7.17 (dd, *J* = 8.3, 2.2 Hz, 1H), 7.13 – 7.05 (m, 3H), 6.91 – 6.81 (m, 2H), 5.47 (app t, *J* = 9.5 Hz, 1H), 5.32 (app t, *J* = 9.6 Hz, 1H), 5.06 (app t, *J* = 9.6 Hz, 1H), 4.33 (d, *J* = 9.8 Hz, 1H), 4.15 – 3.91 (m, 5H), 3.06 – 2.93 (m, 2H), 1.89 (s, 3H), 1.75 (s, 3H), 1.42 (t, *J* = 7.0 Hz, 3H) ppm. **^{13}C NMR (101 MHz, $CDCl_3$)** δ 170.4, 169.0, 165.5, 157.7, 141.0 (q, *J* = 1.3 Hz), 139.3, 135.6, 134.5, 133.8, 131.3, 130.1, 130.0, 129.9, 129.8, 129.6, 129.0, 129.0 (q, *J* = 32.2 Hz), 128.8, 125.9, 125.2 (q, *J* = 3.8 Hz), 124.3 (q, *J* = 272.1 Hz), 114.7, 79.4, 78.5, 74.2, 73.0, 72.5, 63.5, 38.4, 38.1, 20.7, 20.5, 15.0 ppm. **^{19}F NMR (376 MHz, $CDCl_3$)** δ -62.5 ppm. **HRMS (ESI) m/z [M+Na]$^+$:** ($C_{39}H_{36}ClF_3NaO_8$) calcd. 747.1943, found. 747.1942. **IR (neat)** 2926, 2859, 1747, 1719, 1614, 1510, 1477, 1326, 1230, 1111, 826, 708 cm^{-1}. **MP** 135.6-138.5 °C.

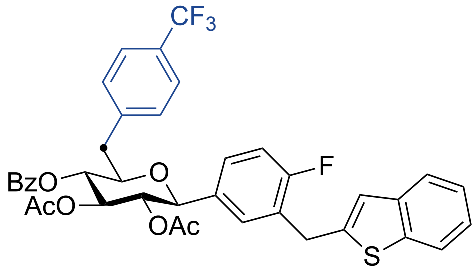

(2*S*,3*S*,4*S*,5*R*,6*R*)-2-(3-(benzo[*b*]thiophen-2-ylmethyl)-4-fluorophenyl)-5-(benzoyloxy)-6-(4-(trifluoromethyl)benzyl)tetrahydro-2*H*-pyran-3,4-diyl diacetate (5y): Following general procedure D, using **3y** (138.4 mg, 0.24 mmol, 1.2 equivalents), 1-bromo-4-(trifluoromethyl)benzene (**4a**, 28 µL, 0.2 mmol, 1 equivalent) and benzene:*tert*-amyl alcohol (1:1, 4.0 mL, 0.05 M), the crude was purified by column chromatography (hexane:$CHCl_3$, 1:1) to afford **5y** as a white solid (73.5 mg, 51% yield). In an independent experiment, 70.6 mg (49% yield) were obtained, giving an average yield of 50%. **^{1}H NMR (400 MHz, $CDCl_3$)** δ 8.01 – 7.94 (m, 2H), 7.78 – 7.72 (m, 1H), 7.70 – 7.66 (m, 1H), 7.60 (ddt, *J* = 8.1, 7.0, 1.3 Hz, 1H), 7.46 (dd, *J* = 8.3, 7.2 Hz, 2H), 7.40 – 7.35 (m, 2H), 7.34 – 7.22 (m, 6H), 7.10 – 7.01 (m, 2H), 5.47 (app t, *J* = 9.5 Hz, 1H), 5.32 (app t, *J* = 9.6 Hz, 1H), 5.07 (app t, *J* = 9.6 Hz, 1H), 4.38 – 4.28 (m, 2H), 4.18 (d, *J* = 16.0 Hz, 1H), 3.95 (ddd, *J* = 9.7, 6.2, 5.1 Hz, 1H), 3.05 – 2.92 (m, 2H), 1.87 (s, 3H), 1.73 (s, 3H) ppm. **^{13}C NMR (101 MHz, $CDCl_3$)** δ 170.4, 169.0, 165.5, 160.8 (d, *J* = 247.7 Hz), 143.2, 140.9 (q, *J* = 1.1 Hz), 140.1, 139.9, 133.8, 132.9 (d, *J* = 3.5 Hz), 130.1, 129.9, 129.7 (d, *J* = 4.5 Hz), 129.0, 129.0 (q, *J* = 32.3 Hz), 128.7, 127.2 (d, *J* = 8.4 Hz), 126.8 (d, *J* = 16.1 Hz), 125.2 (q, *J* = 3.6 Hz), 124.4, 124.3 (d, *J* = 272.1 Hz), 124.0, 123.2, 122.3, 122.2, 115.8 (d, *J* = 22.5 Hz), 79.3, 78.5, 74.2, 73.1, 72.5, 38.1, 29.8 (d, *J* = 3.5 Hz), 20.7, 20.4 ppm. **^{19}F NMR (376 MHz, $CDCl_3$)** δ -62.5, -118.0 ppm. **HRMS (ESI) m/z $[M+Na]^+$:** ($C_{39}H_{32}F_4NaO_7S$) calcd. 743.1697, found. 743.1712. **IR (neat)** 3064, 2946, 2865, 1752, 1719, 1619, 1496, 1327, 1269, 1227, 1160, 1066, 889, 828, 757, 707, 603 cm^{-1}. **MP** 164.5-168.0 °C.

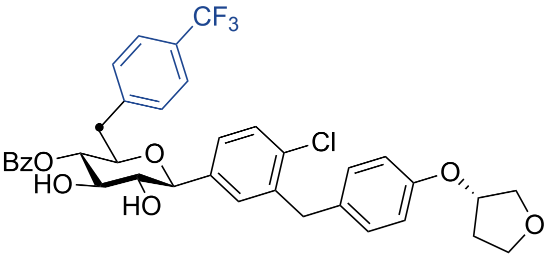

(2*R*,3*S*,4*R*,5*R*,6*S*)-6-(4-chloro-3-(4-(((*S*)-tetrahydrofuran-3-yl)oxy)benzyl)phenyl)-4,5-dihydroxy-2-(4-(trifluoromethyl)benzyl)tetrahydro-2*H*-pyran-3-yl benzoate (5z): Following general procedure D, using **4a** (56 µL, 0.4 mmol, 2.0 equivalents) and benzene:*tert*-amyl alcohol (1:1, 4.0 mL, 0.05 M), the crude was purified by column chromatography (hexane:EtOAc, 1:1) to afford **5z** as a white solid (40.8 mg, 30% yield). In an independent experiment, 41.2 mg (30% yield) were obtained, giving an average yield of 30%. **^{1}H NMR (400 MHz, $CDCl_3$)** δ 8.07 – 7.99 (m, 2H), 7.65 – 7.59 (m, 1H), 7.51 – 7.45 (m, 2H), 7.43 – 7.35 (m, 3H), 7.25 – 7.17 (m, 4H), 7.16 – 7.08 (m, 2H), 6.84 – 6.76 (m, 2H), 5.04 (app t, *J* = 9.5 Hz, 1H), 4.88 (ddd, *J* = 5.5, 4.3, 2.4 Hz, 1H), 4.16 – 4.11 (m, 1H), 4.05 (s, 2H), 3.99 – 3.81 (m, 6H), 3.48 (app t, *J* = 9.1 Hz, 1H), 3.02 (dd, *J* = 14.4, 3.9 Hz, 1H), 2.95 (dd, *J* = 14.4, 7.0 Hz, 1H), 2.90 (s, 1H), 2.35 (s, 1H), 2.20 – 2.10 (m, 2H) ppm. **^{13}C NMR (101 MHz, $CDCl_3$)** δ 166.8, 156.1, 141.3 (q, *J* = 1.4 Hz), 139.3, 137.1, 134.3, 133.9, 131.9, 130.1, 130.1, 130.0, 129.8, 129.3, 128.9 (q, *J* = 32.3 Hz), 128.7, 126.4, 125.2 (q, *J* = 3.7 Hz), 124.3 (q, *J* = 272.0 Hz), 115.5, 80.7, 78.0, 77.4, 76.9, 76.1, 75.0, 73.2, 67.3, 38.5, 38.2, 33.1 ppm. **^{19}F NMR (376 MHz, $CDCl_3$)** δ -62.5 ppm. **HRMS (ESI) m/z $[M+H]^+$:** ($C_{37}H_{35}ClF_3O_7$) calcd. 683.2018,

found. 683.2002. **IR (neat)** 3523, 3456, 2931, 2858, 1700, 1506, 1330, 1268, 1241, 1068, 1016, 807, 708, 564 cm^{-1}. **MP** 137.6-138.8 °C.

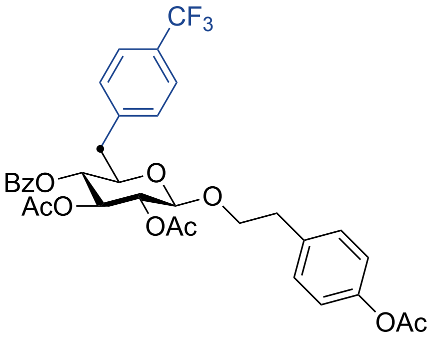

(2*R*,3*R*,4*S*,5*R*,6*R*)-2-(4-acetoxyphenethoxy)-5-(benzoyloxy)-6-(4-(trifluoromethyl)benzyl)tetrahydro-2*H*-pyran-3,4-diyl diacetate (5za): Following general procedure D, using **3za** (123.5 mg, 0.24 mmol, 1.2 equivalents) and 1-bromo-4-(trifluoromethyl)benzene (**4a**, 28 µL, 0.2 mmol, 1 equivalent), the crude was purified by column chromatography (hexane:EtOAc, 10:1 to 4:1) to afford **5za** as a white solid (86.9 mg, 66% yield). In an independent experiment, 79.0 mg (60% yield) were obtained, giving an average yield of 63%. **^{1}H NMR (400 MHz, $CDCl_3$)** δ 8.02 – 7.95 (m, 2H), 7.62 (ddt, *J* = 8.0, 7.0, 1.3 Hz, 1H), 7.52 – 7.45 (m, 4H), 7.31 (d, *J* = 8.0 Hz, 2H), 7.14 (d, *J* = 8.5 Hz, 2H), 7.00 (d, *J* = 8.5 Hz, 2H), 5.39 (app t, *J* = 9.6 Hz, 1H), 5.24 (app t, *J* = 9.6 Hz, 1H), 5.07 (dd, *J* = 9.8, 8.0 Hz, 1H), 4.44 (d, *J* = 8.0 Hz, 1H), 3.96 – 3.86 (m, 1H), 3.80 (dt, *J* = 9.7, 6.1 Hz, 1H), 3.57 (ddd, *J* = 9.6, 7.7, 6.5 Hz, 1H), 2.97 (d, *J* = 6.1 Hz, 2H), 2.89 – 2.75 (m, 2H), 2.30 (s, 3H), 1.95 (s, 3H), 1.91 (s, 3H) ppm. **^{13}C NMR (126 MHz, $CDCl_3$)** δ 170.3, 169.6, 169.5, 165.5, 149.3, 141.3 (q, *J* = 1.1 Hz), 136.2, 133.8, 130.0, 129.9, 129.9, 129.1 (q, *J* = 32.6 Hz), 129.0, 128.7, 125.3 (q, *J* = 3.8 Hz), 124.3 (q, *J* = 272.0 Hz), 121.5, 100.8, 74.4, 72.7, 72.7, 71.5, 70.4, 37.8, 35.4, 21.2, 20.7, 20.6 ppm. **^{19}F NMR (376 MHz, $CDCl_3$)** δ -62.5 ppm. **HRMS (ESI) m/z [M+Na]$^+$:** ($C_{34}H_{33}F_3NaO_{10}$) calcd. 681.1918, found. 681.1913. **IR (neat)** 2957, 2921, 2876, 1749, 1713, 1616, 1508, 1372, 1327, 1274, 1212, 1164, 1122, 1017, 899, 823, 711, 650 cm^{-1}. **MP** 118.8-120.0 °C.

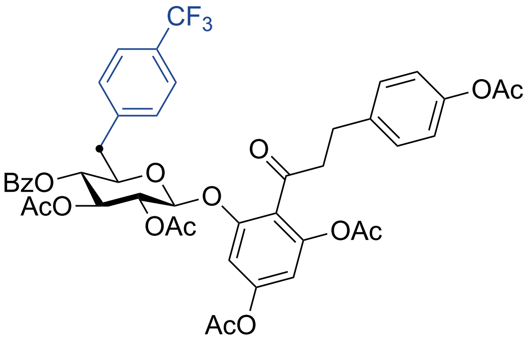

4-(3-(4-acetoxyphenyl)propanoyl)-5-(((2*S*,3*R*,4*S*,5*R*,6*R*)-3,4-diacetoxy-5-(benzoyloxy)-6-(4-(trifluoromethyl)benzyl)tetrahydro-2*H*-pyran-2-yl)oxy)-1,3-phenylene diacetate (5zb): Following general procedure D, using **3zb** (147.0 mg, 0.2 mmol, 1.0 equivalent), 1-bromo-4-

(trifluoromethyl)benzene (**4a**, 56 μL, 0.4 mmol, 2.0 equivalents) and benzene:*tert*-amyl alcohol (1:1, 4.0 mL, 0.05 M), the crude was purified by column chromatography (hexane:EtOAc, 5:1 to 3:1) to afford **5zb** as a white solid (72.1 mg, 41% yield). In an independent experiment, 65.1 mg (37% yield) were obtained, giving an average yield of 39%. **^{1}H NMR (400 MHz, CDCl$_3$)** δ 8.03 – 7.98 (m, 2H), 7.63 (ddt, *J* = 8.1, 7.0, 1.3 Hz, 1H), 7.53 – 7.45 (m, 4H), 7.28 (dd, *J* = 8.5, 6.9 Hz, 4H), 7.04 – 6.99 (m, 2H), 6.63 (d, *J* = 1.9 Hz, 1H), 6.17 (d, *J* = 2.0 Hz, 1H), 5.44 (app t, *J* = 9.6 Hz, 1H), 5.36 – 5.28 (m, 2H), 4.96 (d, *J* = 8.0 Hz, 1H), 3.93 (ddd, *J* = 9.7, 6.7, 5.4 Hz, 1H), 3.21 – 3.11 (m, 1H), 3.06 – 2.91 (m, 5H), 2.28 (s, 3H), 2.18 (s, 3H), 2.08 (s, 3H), 1.97 (s, 3H), 1.91 (s, 3H) ppm. **^{13}C NMR (101 MHz, CDCl$_3$)** δ 200.6, 170.1, 169.7, 169.5, 168.8, 168.6, 165.4, 154.4, 152.0, 149.1, 148.0, 140.9 (q, *J* = 1.1 Hz), 138.8, 134.0, 130.1, 130.0, 129.7, 129.2 (d, *J* = 32.3 Hz), 128.8, 128.8, 125.4 (q, *J* = 3.8 Hz), 124.3 (q, *J* = 271.9 Hz), 123.0, 121.6, 111.7, 106.3, 99.3, 75.2, 72.3, 72.1, 70.7, 46.0, 37.8, 28.9, 21.2, 20.9, 20.7, 20.6, 20.5. **^{19}F NMR (376 MHz, CDCl$_3$)** δ -62.3 ppm. **HRMS (ESI) m/z [M+Na]$^+$:** ($C_{45}H_{41}F_3NaO_{15}$) calcd. 901.2290, found. 901.2289. **IR (neat)** 2928, 1753, 1612, 1508, 1428, 1368, 1324, 1268, 1187, 1115, 1064, 899, 823, 712 cm^{-1}. **MP** 74.8-76.0 °C.

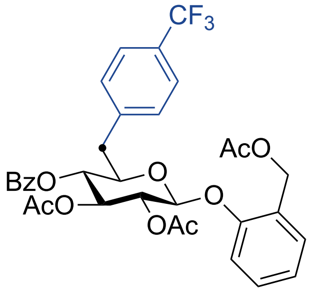

(2*S*,3*R*,4*S*,5*R*,6*R*)-2-(2-(acetoxymethyl)phenoxy)-5-(benzoyloxy)-6-(4-(trifluoromethyl)benzyl)tetrahydro-2*H*-pyran-3,4-diyl diacetate (5zc): Following general procedure D, using **3zc** (101.0 mg, 0.2 mmol, 1.0 equivalent), 1-bromo-4-(trifluoromethyl)benzene (**4a**, 56 μL, 0.4 mmol, 2.0 equivalents) and benzene:*tert*-amyl alcohol (1:1, 4.0 mL, 0.05 M), the crude was purified by column chromatography (hexane:acetone, 5:1 to 4:1) to afford **5zc** as a white solid (77.4 mg, 60% yield). In an independent experiment, 72.0 mg (56% yield) were obtained, giving an average yield of 58%. 74.8 mg, 58% yield, white solid. **^{1}H NMR (400 MHz, CDCl$_3$)** δ 8.06 – 7.99 (m, 2H), 7.62 (ddt, *J* = 8.0, 6.9, 1.3 Hz, 1H), 7.55 – 7.44 (m, 4H), 7.32 (d, *J* = 7.9 Hz, 2H), 7.28 (dd, *J* = 7.6, 1.7 Hz, 1H), 7.00 (td, *J* = 7.5, 1.1 Hz, 1H), 6.87 (td, *J* = 7.8, 1.7 Hz, 1H), 6.25 (dd, *J* = 8.3, 1.1 Hz, 1H), 5.51 (app t, *J* = 9.5 Hz, 1H), 5.43 – 5.31 (m, 2H), 5.11 – 4.99 (m, 2H), 4.91 (d, *J* = 7.9 Hz, 1H), 3.90 (td, *J* = 9.7, 3.2 Hz, 1H), 3.03 (dd, *J* = 14.1, 3.2 Hz, 1H), 2.95 (dd, *J* = 14.1, 9.7 Hz, 1H), 2.09 (s, 6H), 1.94 (s, 3H) ppm. **^{13}C NMR (101 MHz, CDCl$_3$)** δ 170.8, 170.2, 169.4, 165.5, 154.5, 141.2 (q, *J* = 1.2 Hz), 133.9, 130.1, 130.0, 129.3, 129.2, 129.2 (q, *J* = 32.5 Hz), 128.9, 128.8, 125.9, 125.4 (q, *J* = 3.7 Hz), 124.3 (q, *J* = 272.1 Hz), 123.4, 115.6, 99.7, 75.3, 72.6, 72.5, 71.4, 61.1, 37.8, 21.0, 20.7, 20.6 ppm. **^{19}F NMR (376 MHz, CDCl$_3$)** δ -62.5 ppm. **HRMS (ESI) m/z [M+Na]$^+$:**

($C_{33}H_{31}F_3NaO_{10}$) calcd. 667.1762, found. 667.1753. **IR (neat)** 3054, 2960, 2869, 1717, 1603, 1490, 1452, 1378, 1324, 1215, 1173, 899, 822, 764, 710, 655 cm^{-1}. **MP** 125.2-128.0 °C.

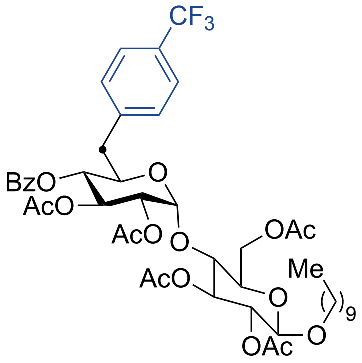

(2*R*,3*R*,4*S*,5*R*,6*R*)-6-(acetoxymethyl)-2-(decyloxy)-5-(((2*R*,3*R*,4*S*,5*R*,6*R*)-3,4-diacetoxy-5-(benzoyloxy)-6-(4-(trifluoromethyl)benzyl)tetrahydro-2*H*-pyran-2-yl)oxy)tetrahydro-2*H*-pyran-3,4-diyl diacetate (5zd): Following general procedure D, using **3zd** (156.2 mg, 0.2 mmol, 1.0 equivalent), 1-bromo-4-(trifluoromethyl)benzene (**4a**, 56 µL, 0.4 mmol, 2.0 equivalents) and benzene:*tert*-amyl alcohol (1:1, 4.0 mL, 0.05 M), the crude was purified by column chromatography (hexane:DCM:acetone, 10:20:1) to afford **5zd** as a white solid (136.7 mg, 74% yield). In an independent experiment, 135.2 mg (73% yield) were obtained, giving an average yield of 74%. **^{1}H NMR (400 MHz, CDCl$_3$)** δ 7.92 (dd, *J* = 8.3, 1.4 Hz, 2H), 7.61 – 7.52 (m, 1H), 7.50 – 7.38 (m, 4H), 7.28 (d, *J* = 8.1 Hz, 2H), 5.49 (dd, *J* = 10.6, 9.5 Hz, 1H), 5.31 (d, *J* = 3.9 Hz, 1H), 5.20 (app t, *J* = 9.2 Hz, 1H), 5.10 (app t, *J* = 9.7 Hz, 1H), 4.83 (dd, *J* = 10.6, 3.9 Hz, 1H), 4.77 (dd, *J* = 9.6, 7.9 Hz, 1H), 4.44 (d, *J* = 7.9 Hz, 1H), 4.26 – 4.14 (m, 2H), 3.85 – 3.73 (m, 2H), 3.69 (t, *J* = 9.2 Hz, 1H), 3.51 – 3.38 (m, 2H), 2.98 – 2.80 (m, 2H), 2.11 (s, 3H), 2.03 (s, 3H), 2.02 (s, 3H), 2.00 (s, 3H), 1.85 (s, 3H), 1.57 – 1.46 (m, 2H), 1.24 (s, 16H), 0.86 (t, *J* = 6.8 Hz, 3H) ppm. **^{13}C NMR (101 MHz, CDCl$_3$)** δ 170.6, 170.5, 170.4, 169.9, 169.7, 165.4, 140.5 (q, *J* = 1.1 Hz), 133.8, 130.2, 129.9, 129.1 (q, *J* = 32.4 Hz), 128.8, 128.7, 125.3 (q, *J* = 3.7 Hz), 124.2 (q, *J* = 272.0 Hz), 100.3, 95.6, 75.1, 73.9, 72.4, 72.2, 72.2, 70.8, 70.7, 70.2, 69.2, 63.3, 37.9, 32.0, 29.7, 29.6, 29.5, 29.4, 25.9, 22.7, 21.0, 20.9, 20.7, 20.6, 14.2 ppm. **^{19}F NMR (376 MHz, CDCl$_3$)** δ -62.5 ppm. **HRMS (ESI) m/z [M+Na]$^+$:** ($C_{46}H_{59}F_3NaO_{16}$) calcd. 947.3647, found. 947.3663. **IR (neat)** 2927, 2856, 1746, 1368, 1325, 1225, 1163, 1026, 902, 711, 600 cm^{-1}. **MP** 48.1-50.3 °C.

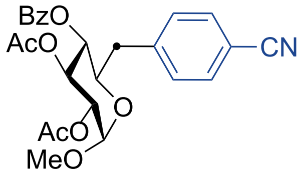

(2*S*,3*R*,4*S*,5*R*,6*R*)-5-(benzoyloxy)-6-(4-cyanobenzyl)-2-methoxytetrahydro-2*H*-pyran-3,4-diyl diacetate (6a): Following general procedure D, using **3a** (87.9 mg, 0.24 mmol, 1.2 equivalents) and methyl 4-bromobenzonitrile (**4b**, 36.4 mg, 0.20 mmol, 1 equivalent), the crude was purified by column chromatography (pentane:EtOAc, 7:1 to 6:1) to afford **6a** as a colourless oil (52.8 mg, 56% yield). In an independent experiment, 59.3 mg (63% yield) were

obtained, giving an average yield of 60%. **^{1}H NMR (400 MHz, CDCl3)** δ 8.00 – 7.97 (m, 2H), 7.62 – 7.58 (m, 1H), 7.54 – 7.51 (m, 2H), 7.48 – 7.43 (m, 2H), 7.32 – 7.29 (m, 2H), 5.64 (dd, *J* = 10.1, 9.4 Hz, 1H), 5.17 (app t, *J* = 9.7 Hz, 1H), 4.93 – 4.87 (m, 2H), 4.07 (td, *J* = 9.4, 3.6 Hz, 1H), 3.06 (s, 3H), 2.93 (dd, *J* = 14.1, 3.6 Hz, 1H), 2.86 (dd, *J* = 14.1, 8.9 Hz, 1H), 2.06 (s, 3H), 1.88 (s, 3H) ppm. **^{13}C NMR (101 MHz, CDCl3)** δ 170.3, 170.0, 165.6, 143.1, 133.8, 132.1, 130.4, 130.0, 129.0, 128.7, 118.9, 110.6, 96.68, 72.9, 71.2, 69.9, 69.5, 55.2, 38.0, 20.8, 20.7 ppm. **IR (neat)** 3062, 2951, 2843, 2228, 2122, 1748, 1729, 1369, 1266, 1219, 1063, 1041, 1026, 711 cm^{-1}. **HRMS (ESI) m/z [M+Na]$^{+}$:** ($C_{25}H_{25}NNaO_8$) calcd. 490.1472, found: 490.1476.

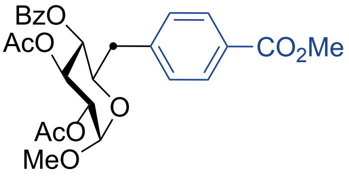

(2*S*,3*R*,4*S*,5*R*,6*R*)-5-(benzoyloxy)-2-methoxy-6-(4-(methoxycarbonyl)benzyl)tetrahydro-2*H*-pyran-3,4-diyl diacetate (6b): Following general procedure D, using **3a** (87.9 mg, 0.24 mmol, 1.2 equivalents) and methyl 4-bromobenzoate (**4c**, 43 mg, 0.20 mmol, 1 equivalent), the crude was purified by column chromatography (pentane:EtOAc, 6:1 to 5:1) to afford **6b** as a colourless oil (68.6 mg, 69% yield). In an independent experiment, 68 mg (68% yield) were obtained, giving an average yield of 69%. **^{1}H NMR (400 MHz, CDCl3)** δ 8.00 (dd, *J* = 8.3, 1.4 Hz, 2H), 7.90 (d, *J* = 8.3 Hz, 2H), 7.60 – 7.56 (m, 1H), 7.47 – 7.42 (m, 2H), 7.28 – 7.25 (m, 2H), 5.64 (dd, *J* = 10.2, 9.4 Hz, 1H), 5.19 (app t, *J* = 9.7 Hz, 1H), 4.93 (dd, *J* = 10.2, 3.7 Hz, 1H), 4.86 (d, *J* = 3.7 Hz, 1H), 4.09 (td, *J* = 9.7, 3.2 Hz, 1H), 3.87 (s, 3H), 3.02 (s, 3H), 2.92 (dd, *J* = 14.1, 3.2 Hz, 1H), 2.84 (dd, *J* = 14.1, 9.4 Hz, 1H), 2.05 (s, 3H), 1.88 (s, 3H) ppm. **^{13}C NMR (101 MHz, CDCl3)** δ 170.3, 170.0, 167.1, 165.7, 143.1, 133.7, 130.0, 129.6, 129.5, 129.1, 128.7, 128.6, 96.6, 73.0, 71.3, 70.0, 69.8, 55.1, 52.08, 37.8, 20.8, 20.7 ppm. **IR (neat)** 2952, 2843, 1748, 1719, 1611, 1268, 1219, 1042, 1025, 709 cm^{-1}. **HRMS (ESI) m/z [M+Na]$^{+}$:** ($C_{26}H_{28}NaO_{10}$) calcd. 523.1575, found: 523.1579.

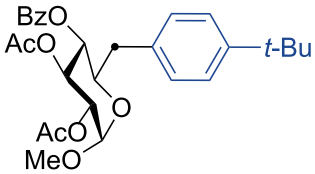

(2*S*,3*R*,4*S*,5*R*,6*R*)-5-(benzoyloxy)-6-(4-(*tert*-butyl)benzyl)-2-methoxytetrahydro-2*H*-pyran-3,4-diyl diacetate (6c): Following general procedure D, using **3a** (87.9 mg, 0.24 mmol, 1.2 equivalents) and 1-bromo-4-(*tert*-butyl)benzene (**4d**, 34.6 µL, 0.20 mmol, 1 equivalent), the crude was purified by column chromatography (pentane:EtOAc, 8:1 to 7:1) to afford **6c** as a colourless oil (49.5 mg, 50% yield). In an independent experiment, 46 mg (46% yield) were obtained, giving an average yield of 48%. **^{1}H NMR (400 MHz, CDCl3)** δ 8.00 – 7.98 (m, 2H), 7.58 (ddt, *J* = 8.7, 7.1, 1.3 Hz, 1H), 7.46 – 7.42 (m, 2H), 7.25 – 7.23 (m, 2H), 7.13 – 7.11 (m,

2H), 5.64 (dd, J = 10.2, 9.4 Hz, 1H), 5.20 (app t, J = 9.7 Hz, 1H), 4.94 (dd, J = 10.2, 3.7 Hz, 1H), 4.90 (d, J = 3.7 Hz, 1H), 4.12 (ddd, J = 9.7, 8.7, 3.5 Hz, 1H), 3.12 (s, 3H), 2.86 (dd, J = 14.3, 3.5 Hz, 1H), 2.78 (dd, J = 14.3, 8.7 Hz, 1H), 2.07 (s, 3H), 1.89 (s, 3H), 1.25 (s, 9H) ppm. **^{13}C NMR (101 MHz, CDCl3)** δ 170.4, 170.1, 165.7, 149.4, 134.4, 133.6, 130.0, 129.3, 129.0, 128.7, 125.2, 96.6, 73.1, 71.4, 70.3, 69.9, 55.1, 37.2, 34.4, 31.4, 20.9, 20.8 ppm. **IR (neat)** 2958, 2869, 1749, 1728, 1367, 1266, 1219, 1043, 1026, 909, 710 cm^{-1}. **HRMS (ESI) m/z [M+Na]$^+$:** ($C_{28}H_{34}NaO_8$) calcd. 521.2146, found: 521.2135.

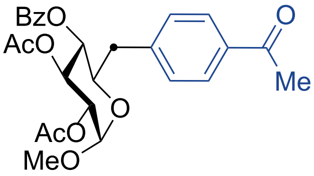

(2*S*,3*R*,4*S*,5*R*,6*R*)-6-(4-acetylbenzyl)-5-(benzoyloxy)-2-methoxytetrahydro-2*H*-pyran-3,4-diyl diacetate (6d): Following general procedure D, using **3a** (87.9 mg, 0.24 mmol, 1.2 equivalents) and 1-(4-bromophenyl)ethan-1-one (**4e**, 39.8 mg, 0.20 mmol, 1 equivalent), the crude was purified by column chromatography (hexane:EtOAc, 6:1 to 5:1) to afford **6d** as a colourless oil (73.1 mg, 75% yield). In an independent experiment, 65.4 mg (68% yield) were obtained, giving an average yield of 72%.**^{1}H NMR (400 MHz, CDCl3)** δ 7.99 – 7.97 (m, 2H), 7.81 (d, J = 8.3 Hz, 2H), 7.57 (ddt, J = 8.8, 7.3, 1.4 Hz, 1H), 7.46 – 7.41 (m, 2H), 7.28 (d, J = 8.2 Hz, 2H), 5.64 (dd, J = 10.2, 9.4 Hz, 1H), 5.18 (app t, J = 9.7 Hz, 1H), 4.92 (dd, J = 10.2, 3.7 Hz, 1H), 4.87 (d, J = 3.7 Hz, 1H), 4.11 (td, J = 9.4, 3.6 Hz, 1H), 3.06 (s, 3H), 2.93 (dd, J = 14.1, 3.6 Hz, 1H), 2.86 (dd, J = 14.1, 9.0 Hz, 1H), 2.52 (s, 3H), 2.05 (s, 3H), 1.88 (s, 3H) ppm. **^{13}C NMR (101 MHz, CDCl3)** δ 197.8, 170.3, 170.0, 165.6, 143.2, 135.6, 133.7, 130.0, 129.7, 129.1, 128.7, 128.4, 96.6, 73.0, 71.3, 70.0, 69.65, 55.2, 37.9, 26.6, 20.8, 20.7 ppm. **IR (neat)** 2953, 2843, 2553, 1747, 1727, 1681, 1607, 1361, 1265, 1219, 1042, 1026, 1042, 1026, 711 cm^{-1}. **HRMS (ESI) m/z [M+Na]$^+$:** ($C_{26}H_{28}NaO_9$) calcd. 507.1626, found: 507.1621.

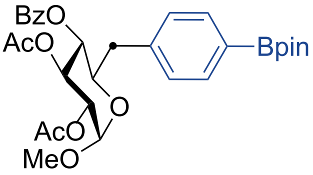

(2*S*,3*R*,4*S*,5*R*,6*R*)-5-(benzoyloxy)-2-methoxy-6-(4-(4,4,5,5-tetramethyl-1,3,2-dioxaborolan-2-yl)benzyl)tetrahydro-2*H*-pyran-3,4-diyl diacetate (6e): Following general procedure D, using **3a** (87.9 mg, 0.24 mmol, 1.2 equivalents) and 2-(4-bromophenyl)-4,4,5,5-tetramethyl-1,3,2-dioxaborolane (**4f**, 56.6 mg, 0.20 mmol, 1 equivalent), the crude was purified by column chromatography (hexane:acetone, 10:1 to 9:1) to afford **6e** as a white solid (64.5 mg, 57% yield). In an independent experiment, 59.5 mg (52% yield) were obtained, giving an average yield of 55%. **^{1}H NMR (400 MHz, CDCl3)** δ 8.02 – 7.99 (m, 2H), 7.67 (d, J = 7.9 Hz, 2H), 7.61 – 7.56 (m, 1H), 7.45 (dd, J = 8.4, 7.2 Hz, 2H), 7.20 (d, J = 8.0 Hz, 2H), 5.65 (dd, J = 10.2, 9.4 Hz, 1H), 5.19 (app t, J = 9.7 Hz, 1H), 4.94 (dd, J = 10.2, 3.7 Hz, 1H), 4.86 (d, J = 3.7 Hz, 1H), 4.10 (td, J = 9.7, 3.0 Hz, 1H), 3.04 (s, 3H), 2.89 (dd, J = 14.1, 3.0 Hz, 1H), 2.79

(dd, $J = 14.1, 9.6$ Hz, 1H), 2.06 (s, 3H), 1.89 (s, 3H), 1.32 (s, 12H) ppm. **^{13}C NMR (101 MHz, CDCl3)** δ 170.3, 170.1, 165.7, 140.9, 134.8, 133.6, 130.0, 129.2, 128.83, 128.7, 96.5, 83.8, 73.1, 71.4, 70.2, 69.9, 55.1, 38.0, 25.0, 20.9, 20.7 ppm. **^{11}B NMR (128 MHz, CDCl3)** δ 30.0 ppm. **IR (neat)** 3540, 2978, 2935, 1749, 1729, 1612, 1358, 1267, 1219, 1143, 1043, 1025, 712, 658 cm^{-1}. **HRMS (ESI) m/z [M+Na]$^+$:** ($C_{30}H_{37}NaO_{10}{}^{10}B$) calcd. 590.2408, found: 590.2388. **MP** 45.3 ºC.

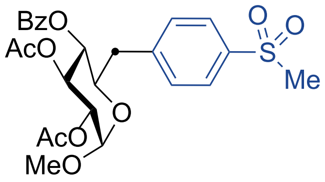

(2*S*,3*R*,4*S*,5*R*,6*R*)-5-(benzoyloxy)-2-methoxy-6-(4-(methylsulfonyl)benzyl)tetrahydro-2*H*-pyran-3,4-diyl diacetate (6f): Following general procedure D, using **3a** (87.9 mg, 0.24 mmol, 1.2 equivalents) and 1-bromo-4-(methylsulfonyl)benzene (**4g**, 47 mg, 0.20 mmol, 1 equivalent), the crude was purified by column chromatography (hexane:acetone, 5:1 to 4:1) to afford **6f** as a white solid (65 mg, 62% yield). In an independent experiment, 64.7 mg (62% yield) were obtained, giving an average yield of 62%.**^{1}H NMR (400 MHz, CDCl3)** δ 8.01 – 7.98 (m, 2H), 7.81 (d, $J = 8.4$ Hz, 2H), 7.61 – 7.57 (m, 1H), 7.48 – 7.39 (m, 4H), 5.64 (app t, $J = 9.8$ Hz, 1H), 5.17 (app t, $J = 9.7$ Hz, 1H), 4.93 – 4.87 (m, 2H), 4.11 (td, $J = 9.3, 3.6$ Hz, 1H), 3.08 (s, 3H), 3.01 – 2.87 (m, 5H), 2.06 (s, 3H), 1.88 (s, 3H) ppm. **^{13}C NMR (101 MHz, CDCl3)** δ 170.3, 170.0, 165.6, 144.0, 138.9, 133.8, 130.6, 130.0, 129.0, 128.8, 127.4, 96.7, 72.8, 71.2, 69.9, 69.4, 55.3, 44.6, 37.7, 20.8, 20.7 ppm. **IR (neat)** 3013, 2929, 2842, 1747, 1726, 1219, 1148, 1026, 909, 711 cm^{-1}. **HRMS (ESI) m/z [M+Na]$^+$:** ($C_{25}H_{28}NaO_{10}S$) calcd. 543.1295, found: 543.1307. **MP** 76 ºC.

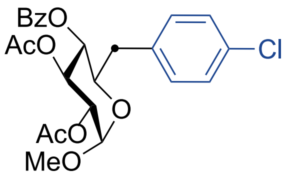

(2*S*,3*R*,4*S*,5*R*,6*R*)-5-(benzoyloxy)-6-(4-chlorobenzyl)-2-methoxytetrahydro-2*H*-pyran-3,4-diyl diacetate (6g): Following general procedure D, using **3a** (87.9 mg, 0.24 mmol, 1.2 equivalents) and 1-bromo-4-chlorobenzene (**4h**, 38.3 mg, 0.20 mmol, 1 equivalent), the crude was purified by column chromatography (hexane:acetone, 10:1) to afford **6g** as a colourless oil (45.5 mg, 48% yield). In an independent experiment, 45.8 mg (48% yield) were obtained, giving an average yield of 48%. **^{1}H NMR (400 MHz, CDCl3)** δ 8.01 – 7.99 (m, 2H), 7.62 – 7.58 (m, 1H), 7.48 – 7.44 (m, 2H), 7.22 – 7.19 (m, 2H), 7.16 – 7.11 (m, 2H), 5.67 – 5.62 (m, 1H), 5.17 (app t, $J = 9.7$ Hz, 1H), 4.92 (dd, $J = 10.2, 3.7$ Hz, 1H), 4.88 (d, $J = 3.7$ Hz, 1H), 4.04 (td, $J = 9.6, 3.2$ Hz, 1H), 3.07 (s, 3H), 2.85 (dd, $J = 14.2, 3.2$ Hz, 1H), 2.76 (dd, $J = 14.2, 9.2$ Hz, 1H), 2.07 (s, 3H), 1.89 (s, 3H) ppm. **^{13}C NMR (101 MHz, CDCl3)** δ 170.4, 170.1, 165.7, 136.0, 133.7, 132.5, 130.9, 130.0, 129.2, 128.7, 128.5, 96.6, 73.0, 71.4, 70.1, 69.9, 55.2, 37.2,

20.9, 20.8 ppm. **IR (neat)** 2936, 2842, 2255, 1748, 1728, 1266, 1219, 1043, 1027, 908, 729, 710 cm^{-1}. **HRMS (ESI) m/z [M+Na]$^{+}$:** ($C_{24}H_{25}ClNaO_8$) calcd. 499.1130, found: 499.1118.

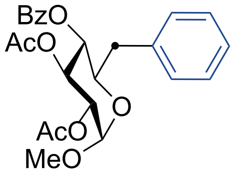

(2*S*,3*R*,4*S*,5*R*,6*R*)-5-(benzoyloxy)-6-benzyl-2-methoxytetrahydro-2*H*-pyran-3,4-diyl diacetate (6h): Following general procedure D, using **3a** (87.9 mg, 0.24 mmol, 1.2 equivalents) and bromobenzene (**4i**, 21 µL, 0.20 mmol, 1 equivalent), the crude was purified by column chromatography (hexane:acetone, 10:1 to 8:1) to afford **6h** as a colourless oil (46.1 mg, 52% yield). In an independent experiment, 48.7 mg (55% yield) were obtained, giving an average yield of 54%. **^{1}H NMR (400 MHz, CDCl3)** δ 8.03 – 8.00 (m, 2H), 7.61 – 7.56 (m, 1H), 7.48 – 7.43 (m, 2H), 7.24 – 7.14 (m, 4H), 5.65 (app t, *J* = 9.8 Hz, 1H), 5.20 (app t, *J* = 9.7 Hz, 1H), 4.94 (dd, *J* = 10.1, 3.8 Hz, 1H), 4.88 (d, *J* = 3.7 Hz, 1H), 4.09 (td, *J* = 9.7, 3.0 Hz, 1H), 3.05 (s, 3H), 2.89 (dd, *J* = 14.2, 3.0 Hz, 1H), 2.78 (dd, *J* = 14.2, 9.4 Hz, 1H), 2.07 (s, 3H), 1.90 (s, 3H) ppm.**^{13}C NMR (101 MHz, CDCl3)** δ 170.4, 170.1, 165.7, 137.6, 133.6, 130.0, 129.5, 129.3, 128.7, 128.3, 126.6, 96.6, 73.1, 71.4, 70.2, 70.1, 55.0, 37.8, 20.9, 20.8 ppm. **IR (neat)** 3030, 2955, 2843, 1746, 1726, 1369, 1265, 1218, 1042, 1026, 710 cm^{-1}. **HRMS (ESI) m/z [M+Na]$^{+}$:** ($C_{24}H_{26}NaO_8$) calcd. 465.1520, found: 465.1510.

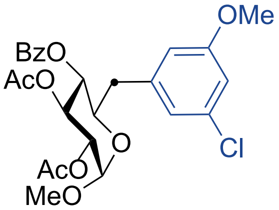

(2*S*,3*R*,4*S*,5*R*,6*R*)-5-(benzoyloxy)-6-(3-chloro-5-methoxybenzyl)-2-methoxytetrahydro-2*H*-pyran-3,4-diyl diacetate (6i): Following general procedure D, using **3a** (87.9 mg, 0.24 mmol, 1.2 equivalents) and 1-bromo-3-chloro-5-methoxybenzene (**4j**, 44.3 mg, 0.20 mmol, 1 equivalent), the crude was purified by column chromatography (hexane:acetone, 10:1) to afford **6i** as a colourless oil (68.3 mg, 67% yield). In an independent experiment, 61.5 mg (61% yield) were obtained, giving an average yield of 64%. **^{1}H NMR (400 MHz, CDCl3)** δ 8.00 – 7.97 (m, 2H), 7.61 –7.56 (m, 1H), 7.47 – 7.42 (m, 2H), 6.79 (t, *J* = 1.6 Hz, 1H), 6.67 (t, *J* = 2.1 Hz, 1H), 6.61 (dd, *J* = 2.4, 1.4 Hz, 1H), 5.64 (app t, *J* = 9.7 Hz, 1H), 5.17 (app t, *J* = 9.7 Hz, 1H), 4.95 – 4.90 (m, 2H), 4.12 – 4.06 (m, 1H), 3.72 (s, 3H), 3.16 (s, 3H), 2.81 (dd, *J* = 14.2, 3.7 Hz, 1H), 2.75 (dd, *J* = 14.2, 8.7 Hz, 1H), 2.07 (s, 3H), 1.89 (s, 3H) ppm. **^{13}C NMR (101 MHz, CDCl3)** δ 170.3, 170.1, 165.6, 160.2, 140.3, 134.6, 133.7, 130.0, 129.1, 128.7, 121.8, 114.0, 112.4, 96.6, 72.9, 71.3, 70.1, 69.6, 55.5, 55.3, 37.7, 20.9, 20.7 ppm. **IR (neat)** 2939, 2839, 1746, 1727, 1576, 1266, 1218, 1043, 1026, 710 cm^{-1}. **HRMS (ESI) m/z [M+Na]$^{+}$:** ($C_{25}H_{27}ClNaO_9$) calcd. 529.1236, found: 529.1216.

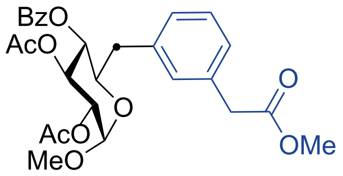

(2*S*,3*R*,4*S*,5*R*,6*R*)-5-(benzoyloxy)-2-methoxy-6-(3-(2-methoxy-2-oxoethyl)benzyl)tetrahydro-2*H*-pyran-3,4-diyl diacetate (6j): Following general procedure D, using **3a** (87.9 mg, 0.24 mmol, 1.2 equivalents) and methyl 2-(3-bromophenyl)acetate (**4k**, 33 µL, 0.20 mmol, 1 equivalent), the crude was purified by column chromatography (hexane:acetone, 9:1 to 7:1) to afford **6j** as a colourless oil (57.6 mg, 56% yield). In an independent experiment, 60.4 mg (59% yield) were obtained, giving an average yield of 58%. **^{1}H NMR (400 MHz, CDCl3)** δ 8.02 – 7.99 (m, 2H), 7.61 – 7.56 (m, 1H), 7.47 – 7.43 (m, 2H), 7.21 – 7.17 (m, 1H), 7.11 – 7.06 (m, 3H), 5.67 – 5.62 (m, 1H), 5.18 (app t, *J* = 9.7 Hz, 1H), 4.93 (dd, *J* = 10.2, 3.7 Hz, 1H), 4.88 (d, *J* = 3.7 Hz, 1H), 4.08 (td, *J* = 9.6, 3.1 Hz, 1H), 3.65 (s, 3H), 3.54 (s, 2H), 3.05 (s, 3H), 2.87 (dd, *J* = 14.2, 3.1 Hz, 1H), 2.77 (dd, *J* = 14.2, 9.3 Hz, 1H), 2.06 (s, 3H), 1.89 (s, 3H) ppm. **^{13}C NMR (101 MHz, CDCl3)** δ 172.0, 170.3, 170.1, 165.7, 138.0, 134.0, 134.0, 130.3, 130.0, 129.2, 128.7, 128.5, 128.3, 127.5, 96.5, 73.0, 71.4, 70.2, 69.9, 55.0, 52.1, 41.2, 37.6, 20.9, 20.7 ppm. **IR (neat)** 2953, 2842, 1727, 1265, 1219, 1042, 1026, 711 cm^{-1}. **HRMS (ESI) m/z [M+Na]$^{+}$:** ($C_{27}H_{30}NaO_{10}$) calcd. 537.1726, found: 537.1726.

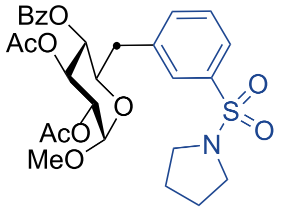

(2*S*,3*R*,4*S*,5*R*,6*R*)-5-(benzoyloxy)-2-methoxy-6-(3-(pyrrolidin-1-ylsulfonyl)benzyl)tetrahydro-2*H*-pyran-3,4-diyl diacetate (6k): Following general procedure D, using **3a** (87.9 mg, 0.24 mmol, 1.2 equivalents) and 1-((3-bromophenyl)sulfonyl)pyrrolidine (**4l**, 58 mg, 0.20 mmol, 1 equivalent), the crude was purified by HPLC (*Column:* SunFire C18 (100x4.6 mm, 5µm), *mobile phase*: H_2O:MeOH, 30:70, *flow*: 1mL/min, *wave length*: 210 nm) to afford **6k** as a colourless oil (56.4 mg, 49% yield). In an independent experiment, 56 mg (49% yield) were obtained, giving an average yield of 49%. **^{1}H NMR (400 MHz, CDCl3)** δ 8.05 – 8.02 (m, 2H), 7.72 – 7.59 (m, 3H), 7.52 – 7.39 (m, 4H), 5.65 (dd, *J* = 10.1, 9.4 Hz, 1H), 5.17 (app t, *J* = 9.7 Hz, 1H), 4.90 (dd, *J* = 10.1, 3.7 Hz, 1H), 4.86 (d, *J* = 3.7 Hz, 1H), 4.08 (td, *J* = 9.6, 3.0 Hz, 1H), 3.22 (m, 4H), 3.05 (s, 3H), 2.96 (dd, *J* = 14.3, 3.1 Hz, 1H), 2.87 (dd, *J* = 14.2, 9.3 Hz, 1H), 2.06 (s, 3H), 1.89 (s, 3H), 1.75 – 1.70 (m, 4H) ppm. **^{13}C NMR (101 MHz, CDCl3)** δ 170.34, 170.01, 165.73, 138.82, 137.00, 134.05, 133.82, 130.06, 129.04, 128.94, 128.79, 128.51, 125.85, 96.69, 72.81, 71.26, 69.95, 69.61, 55.23, 48.04, 37.44, 25.37, 20.87, 20.72 ppm. **IR (neat)** 2955, 1727, 1371, 1344, 1218, 1155, 1026, 712, 606 cm^{-1}. **HRMS (ESI) m/z [M+Na]$^{+}$:** ($C_{28}H_{34}NO_{10}S$) calcd. 576.1898, found: 576.1892.

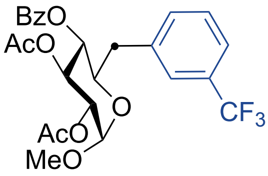

(2*S*,3*R*,4*S*,5*R*,6*R*)-5-(benzoyloxy)-2-methoxy-6-(3-(trifluoromethyl)benzyl)tetrahydro-2*H*-pyran-3,4-diyl diacetate (6l): Following general procedure D, using **3a** (87.9 mg, 0.24 mmol, 1.2 equivalents) and 1-bromo-3-(trifluoromethyl)benzene (**4m**, 27 μL, 0.20 mmol, 1 equivalent), the crude was purified by column chromatography (hexane:EtOAc, 8:1 to 6:1) to afford **6l** as a colourless oil (64 mg, 63% yield). In an independent experiment, 53.8 mg (53% yield) were obtained, giving an average yield of 58%. **^{1}H NMR (400 MHz, CDCl3)** δ 8.03 – 7.99 (m, 2H), 7.62 – 7.57 (m, 1H), 7.48 – 7.33 (m, 6H), 5.66 (dd, *J* = 10.2, 9.4 Hz, 1H), 5.19 (app t, *J* = 9.7 Hz, 1H), 4.94 (dd, *J* = 10.2, 3.7 Hz, 1H), 4.89 (d, *J* = 3.8 Hz, 1H), 4.10 (td, *J* = 9.6, 3.4 Hz, 1H), 3.05 (s, 3H), 2.94 (dd, *J* = 14.3, 3.4 Hz, 1H), 2.87 (dd, *J* = 14.3, 9.2 Hz, 1H), 2.07 (s, 3H), 1.89 (s, 3H) ppm. **^{13}C NMR (101 MHz, CDCl3)** δ 170.3, 170.1, 165.7, 138.6, 133.8, 133.1, 133.0, 130.7 (q, *J* = 32.1 Hz), 130.0, 129.1, 128.7, 126.1 (q, *J* = 3.8 Hz), 124.2 (q, *J* = 272.2 Hz), 123.5 (q, *J* = 3.9 Hz), 96.7, 73.0, 71.3, 70.0, 69.7, 55.1, 37.5, 20.9, 20.7 ppm. **^{19}F NMR (376 MHz, CDCl3)** -62.7 ppm. **IR (neat)** 3021, 2939, 2843, 14746, 1728, 1331, 1220, 1122, 1026, 908, 709 cm^{-1}. **HRMS (ESI) m/z [M+Na]$^{+}$:** ($C_{25}H_{27}F_{3}NaO_{8}$) calcd. 533.1394, found: 533.1386.

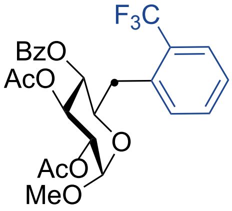

(2*S*,3*R*,4*S*,5*R*,6*R*)-5-(benzoyloxy)-2-methoxy-6-(2-(trifluoromethyl)benzyl)tetrahydro-2*H*-pyran-3,4-diyl diacetate (6m): Following general procedure D, using **3a** (87.9 mg, 0.24 mmol, 1.2 equivalents) and 1-bromo-2-(trifluoromethyl)benzene (**4n**, 27 μL, 0.20 mmol, 1 equivalent), the crude was purified by column chromatography (hexane:EtOAc, 8:1 to 6:1) to afford **6m** as a colourless oil (50.4 mg, 49% yield). In an independent experiment, 42.7 mg (43% yield) were obtained, giving an average yield of 46%. **^{1}H NMR (400 MHz, CDCl3)** δ 8.04 – 8.02 (m, 2H), 7.61 – 7.57 (m, 2H), 7.48 – 7.41 (m, 4H), 7.30 – 7.26 (m, 1H), 5.63 (app t, *J* = 9.8 Hz, 1H), 5.24 (app t, *J* = 9.7 Hz, 1H), 4.96 (dd, *J* = 10.2, 3.7 Hz, 1H), 4.88 (d, *J* = 3.7 Hz, 1H), 4.06 (td, *J* = 10.1, 2.2 Hz, 1H), 3.21 (d, *J* = 15.4 Hz, 1H), 2.93 (s, 3H), 2.86 (dd, *J* = 14.5, 10.3 Hz, 1H), 2.06 (s, 3H), 1.91 (s, 3H) ppm **^{13}C NMR (101 MHz, CDCl3)** δ 170.4, 170.1, 165.9, 136.2, 133.6, 133.3, 131.4, 130.1, 129.1, 128.8 (q, *J* = 29.4 Hz), 128.7, 126.8, 126.1 (q, *J* = 5.6 Hz), 124.6 (q, *J* = 274, 5), 96.5, 73.1, 71.5, 70.2, 69.4, 54.8, 34.6, 20.9, 20.8 ppm. **^{19}F NMR (376 MHz, CDCl3)** -59.0 ppm. **IR (neat)** 2940, 2842, 1747, 1727, 1313, 1266, 1219, 1116, 1037, 1026, 908, 710 cm^{-1}. **HRMS (ESI) m/z [M+Na]$^{+}$:** ($C_{25}H_{27}F_{3}NaO_{8}$) calcd. 533.1394, found: 533.1389.

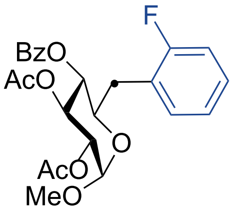

(2*S*,3*R*,4*S*,5*R*,6*R*)-5-(benzoyloxy)-6-(2-fluorobenzyl)-2-methoxytetrahydro-2*H*-pyran-3,4-diyl diacetate (6n): Following general procedure D, using **3a** (87.9 mg, 0.24 mmol, 1.2 equivalents) and 1-bromo-2-fluorobenzene (**4o**, 22 µL, 0.20 mmol, 1 equivalent), the crude was purified by column chromatography (hexane:EtOAc, 8:1 to 6:1) to afford **6n** as a colourless oil (45.2 mg, 49% yield). In an independent experiment, 42 mg (46% yield) were obtained, giving an average yield of 48%. **^{1}H NMR (400 MHz, CDCl3)** δ 8.02 – 7.99 (m, 2H), 7.60 – 7.58 (m, 1H), 7.45 (dd, *J* = 8.3, 7.2 Hz, 2H), 7.22 (td, *J* = 7.5, 1.8 Hz, 1H), 7.14 (tdd, *J* = 7.4, 5.2, 1.8 Hz, 1H), 7.01 – 6.94 (m, 2H), 5.64 (dd, *J* = 10.2, 9.4 Hz, 1H), 5.20 (app t, *J* = 9.7 Hz, 1H), 4.95 (dd, *J* = 10.2, 3.7 Hz, 1H), 4.87 (d, *J* = 3.7 Hz, 1H), 4.15 (td, *J* = 9.7, 3.2 Hz, 1H), 3.05 (s, 3H), 3.02 – 2.97 (m, 1H), 2.81 (dd, *J* = 14.2, 9.5 Hz, 1H), 2.06 (s, 3H), 1.90 (s, 3H) ppm. **^{13}C NMR (101 MHz, CDCl3)** δ 170.4, 170.1, 165.8, 161.2 (d, *J* = 245.5 Hz), 133.6, 132.0 (d, *J* = 4.6 Hz), 130.0, 129.3, 128.7, 128.4 (d, *J* = 8.1 Hz), 124.6 (d, *J* = 15.2 Hz), 123.9 (d, *J* = 3.6 Hz), 115.2 (d, *J* = 22.1 Hz), 96.5, 73.2, 71.4, 70.2, 68.9 (d, *J* = 1.6 Hz), 54.9, 31.3 (d, *J* = 2.1 Hz), 20.9, 20.8 ppm. **^{19}F NMR (376 MHz, CDCl3)** -118.1 ppm. δ **IR (neat)** 3009, 2955, 2844, 1747, 1727, 1266, 1219, 1041, 1026, 756, 710 cm^{-1}. **HRMS (ESI) m/z [M+Na]$^{+}$:** ($C_{24}H_{25}FNaO_8$) calcd. 483.1426, found: 483.1413.

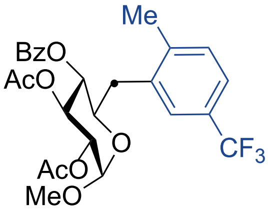

(2*S*,3*R*,4*S*,5*R*,6*R*)-5-(benzoyloxy)-2-methoxy-6-(2-methyl-5-(trifluoromethyl)benzyl)tetrahydro-2*H*-pyran-3,4-diyl diacetate (6o): Following general procedure D, using **3a** (87.9 mg, 0.24 mmol, 1.2 equivalents) and 2-bromo-1-methyl-4-(trifluoromethyl)benzene (**4p**, 47.8 mg, 0.20 mmol, 1 equivalent), the crude was purified by column chromatography (hexane:EtOAc, 8:1 to 7:1) to afford **6o** as a colourless oil (50.6 mg, 48% yield). In an independent experiment, 50 mg (48% yield) were obtained, giving an average yield of 48%. **^{1}H NMR (400 MHz, CDCl3)** δ 8.04 – 8.01 (m, 2H), 7.62 – 7.58 (m, 1H), 7.49 – 7.43 (m, 3H), 7.32 (dd, *J* = 7.8, 1.9 Hz, 1H), 7.20 (d, *J* = 8.0 Hz, 1H), 5.65 (dd, *J* = 10.3, 9.4 Hz, 1H), 5.25 (app t, *J* = 9.7 Hz, 1H), 4.96 (dd, *J* = 10.3, 3.8 Hz, 1H), 4.86 (d, *J* = 3.7 Hz, 1H), 4.04 (td, *J* = 9.6, 3.5 Hz, 1H), 2.96 (s, 3H), 2.93 – 2.80 (m, 2H), 2.31 (s, 3H), 2.07 (s, 3H), 1.91 (s, 3H) ppm **^{13}C NMR (101 MHz, CDCl3)** 170.4, 170.1, 166.8, 140.8, 137.1, 133.8, 130.6, 130.0, 129.0, 128.8, 128.3 (q, J = 32.1 Hz), 126.6 (q, J = 3.7 Hz), 124.4 (q, J = 271.5 Hz), 123.3 (q, J = 3.7 Hz), 96.6, 73.3, 71.3, 70.1, 69.7, 54.9, 34.4, 20.9, 20.8, 19.9 ppm. **^{19}F NMR (376**

MHz, CDCl3) -62.5 ppm. **IR (neat)** 2935, 2845, 1747, 1727, 1332, 1266, 1221, 1118, 1042, 1026, 710, 474 cm^{-1}. **HRMS (ESI) m/z [M+Na]$^+$:** ($C_{26}H_{27}F_3NaO_8$) calcd. 547.1550, found: 547.1540.

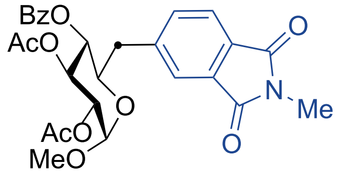

(2*S*,3*R*,4*S*,5*R*,6*R*)-5-(benzoyloxy)-2-methoxy-6-((2-methyl-1,3-dioxoisoindolin-5-yl)methyl)tetrahydro-2*H*-pyran-3,4-diyl diacetate (6p): Following general procedure D, using **3a** (87.9 mg, 0.24 mmol, 1.2 equivalents) and 5-bromo-2-methylisoindoline-1,3-dione (**4q**, 48 mg, 0.20 mmol, 1 equivalent), the crude was purified by column chromatography (hexane:acetone, 6:1 to 5:1) to afford **6p** as a colourless oil (64.9 mg, 62% yield). In an independent experiment, 60.7 mg (58% yield) were obtained, giving an average yield of 60%. **^{1}H NMR (400 MHz, CDCl3)** δ 7.99 – 7.96 (m, 2H), 7.70 – 7.69 (m, 2H), 7.61 – 7.57 (m, 1H), 7.53 (dd, *J* = 7.8, 1.3 Hz, 1H), 7.45 (t, *J* = 7.8 Hz, 2H), 5.65 (app t, *J* = 9.7 Hz, 1H), 5.19 (app t, *J* = 9.7 Hz, 1H), 4.94 – 4.89 (m, 2H), 4.12 (ddd, *J* = 10.0, 8.3, 4.4 Hz, 1H), 3.14 (s, 3H), 3.08 (s, 3H), 3.34 – 2.94 (m, 2H), 2.07 (s, 3H), 1.89 (s, 3H) ppm. **^{13}C NMR (101 MHz, CDCl3)** δ 170.3, 170.0, 168.5, 168.4, 165.6, 144.6, 135.1, 133.8, 132.6, 130.7, 130.0, 128.9, 128.7, 124.2, 123.1, 96.8, 72.9, 71.2, 69.9, 69.6, 55.3, 38.2, 24.0, 20.8, 20.7 ppm. **IR (neat)** 2941, 2256, 1748, 1709, 1450, 1429, 1380, 1220, 1027, 908, 727, 711 cm^{-1}. **HRMS (ESI) m/z [M+Na]$^+$:** ($C_{27}H_{27}NNaO_{10}$) calcd. 548.1527, found: 548.1523.

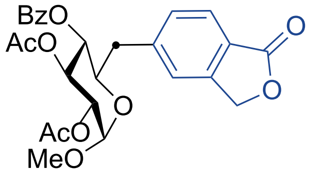

(2*S*,3*R*,4*S*,5*R*,6*R*)-5-(benzoyloxy)-2-methoxy-6-((1-oxo-1,3-dihydroisobenzofuran-5-yl)methyl)tetrahydro-2*H*-pyran-3,4-diyl diacetate 6q: Following general procedure D, using **3a** (87.9 mg, 0.24 mmol, 1.2 equivalents) and 5-bromoisobenzofuran-1(3*H*)-one (**4r**, 42.6 mg, 0.20 mmol, 1 equivalent), the crude was purified by column chromatography (DCM:MeOH, 120:1) to afford **6q** as a white solid (64.8 mg, 65% yield). In an independent experiment, 65.2 mg (65% yield) were obtained, giving an average yield of 65%. **^{1}H NMR (400 MHz, $CDCl_3$)** δ 7.98 – 7.92 (m, 2H), 7.77 (d, *J* = 7.9 Hz, 1H), 7.62 – 7.56 (m, 1H), 7.43 (t, *J* = 7.8 Hz, 2H), 7.38 (dd, *J* = 7.9, 1.3 Hz, 1H), 7.29 (s, 1H), 5.64 (app t, *J* = 9.7 Hz, 1H), 5.25 – 5.12 (m, 3H), 4.94 – 4.88 (m, 2H), 4.14 (ddd, *J* = 9.9, 8.1, 4.4 Hz, 1H), 3.09 (s, 3H), 3.04 – 2.92 (m, 2H), 2.06 (s, 3H), 1.88 (s, 3H) ppm. **^{13}C NMR (101 MHz, $CDCl_3$)** δ 170.9, 170.3, 170.0, 165.6, 147.0, 144.5, 133.9, 130.5, 129.9, 128.9, 128.7, 125.6, 124.3, 123.2, 96.7, 72.9, 71.2, 69.9, 69.7, 69.5, 55.3, 38.4, 20.8, 20.7 ppm. **HRMS (ESI) m/z [M+Na]$^+$:**

($C_{26}H_{26}NaO_{10}$) calcd. 521.1418, found. 521.1414. **IR (neat)** 3065, 2939, 1746, 1620, 1602, 1451, 1366, 1317, 1219, 1122, 1040, 907, 846, 711, 686 cm^{-1}. **MP** 72.2-75.0 °C.

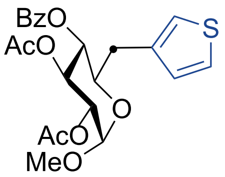

(2*S*,3*R*,4*S*,5*R*,6*R*)-5-(benzoyloxy)-2-methoxy-6-(thiophen-3-ylmethyl)tetrahydro-2*H*-pyran-3,4-diyl diacetate (6r): Following general procedure D, using **3a** (87.9 mg, 0.24 mmol, 1.2 equivalents) and 3-bromothiophene (**4s**, 32.6 mg, 0.20 mmol, 1 equivalent), the crude was purified by column chromatography (hexane:acetone, 10:1 to 8:1) to afford **6r** as a colourless oil (43.1 mg, 48% yield). In an independent experiment, 39.5 mg (44% yield) were obtained, giving an average yield of 46%. **^{1}H NMR (400 MHz, CDCl$_3$)** δ 8.06 – 7.95 (m, 2H), 7.63 – 7.53 (m, 1H), 7.49 – 7.41 (m, 2H), 7.20 (dd, *J* = 4.9, 3.0 Hz, 1H), 7.01 (dd, *J* = 3.0, 1.2 Hz, 1H), 6.97 (dd, *J* = 4.9, 1.3 Hz, 1H), 5.65 (ddd, *J* = 11.3, 9.4, 1.6 Hz, 1H), 5.16 (app t, *J* = 9.7 Hz, 1H), 4.95 – 4.90 (m, 2H), 4.08 (ddd, *J* = 9.9, 8.5, 3.4 Hz, 1H), 3.19 (s, 3H), 2.91 (dd, *J* = 14.7, 3.4 Hz, 1H), 2.85 (dd, *J* = 14.7, 8.5 Hz, 1H), 2.07 (s, 3H), 1.89 (s, 3H) ppm. **^{13}C NMR (101 MHz, CDCl$_3$)** δ 170.4, 170.1, 165.7, 137.5, 133.6, 130.0, 129.2, 128.9, 128.7, 125.3, 122.5, 96.7, 72.8, 71.4, 70.1, 69.5, 55.3, 32.1, 20.9, 20.7 ppm. **HRMS (ESI) m/z [M+Na]$^+$:** ($C_{22}H_{24}NaO_8S$) calcd. 471.1084, found. 471.1074. **IR (neat)** 2929, 2844, 1725, 1602, 1451, 1369, 1217, 1123, 1026, 906, 782, 711, 603 cm^{-1}.

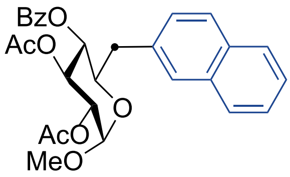

(2*S*,3*R*,4*S*,5*R*,6*R*)-5-(benzoyloxy)-2-methoxy-6-(naphthalen-2-ylmethyl)tetrahydro-2*H*-pyran-3,4-diyl diacetate (6s): Following general procedure D, using **3a** (87.9 mg, 0.24 mmol, 1.2 equivalents) and 2-bromonaphthalene (**4t**, 41.4 mg, 0.20 mmol, 1 equivalent), the crude was purified by column chromatography (hexane:EtOAc, 8:1 to 6:1) to afford **6s** as a white solid (55.5 mg, 56% yield). In an independent experiment, 59.3 mg (60% yield) were obtained, giving an average yield of 58%. **^{1}H NMR (400 MHz, CDCl3)** δ 8.03 – 8.00 (m, 2H), 7.77 – 7.72 (m, 3H), 7.64 – 7.63 (m, 1H), 7.60 – 7.55 (m, 1H), 7.47 – 7.34 (m, 5H), 5.69 (dd, *J* = 10.2, 9.4 Hz, 1H), 5.26 (app t, *J* = 9.7 Hz, 1H), 4.97 (dd, *J* = 10.2, 3.7 Hz, 1H), 4.89 (d, *J* = 3.7 Hz, 1H), 4.22 (td, *J* = 9.6, 3.2 Hz, 1H), 3.06 (dd, *J* = 14.2, 3.2 Hz, 1H), 3.02 (s, 3H), 2.97 (dd, *J* = 14.2, 9.2 Hz, 1H), 2.07 (s, 3H), 1.91 (s, 3H) ppm. **^{13}C NMR (101 MHz, CDCl3)** δ 170.3, 170.1, 165.7, 135.1, 133.6, 133.5, 132.3, 130.0, 129.2, 128.7, 128.0, 127.9, 127.7, 127.7, 127.7, 126.0, 125.5, 96.6, 73.2, 71.4, 70.2, 70.0, 55.1, 38.0, 20.9, 20.8 ppm. **IR (neat)** 3058, 2953, 2843, 1747, 1724, 1368, 1266, 1230, 1026, 859, 749, 711 cm^{-1}. **HRMS (ESI) m/z [M+Na]$^+$:** ($C_{28}H_{28}NaO_8$) calcd. 515.1676, found: 515.1670. **MP** 50.8 ºC.

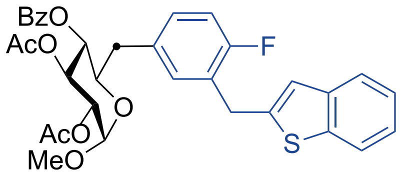

(2*S*,3*R*,4*S*,5*R*,6*R*)-6-(3-(benzo[*b*]thiophen-2-ylmethyl)-4-fluorobenzyl)-5-(benzoyloxy)-2-methoxytetrahydro-2*H*-pyran-3,4-diyl diacetate (6t): Following general procedure D, using **3a** (87.9 mg, 0.24 mmol, 1.2 equivalents) and 2-(5-bromo-2-fluorobenzyl)benzo[*b*]thiophene (**4u**, 64.2 mg, 0.20 mmol, 1 equivalent), the crude was purified by column chromatography (hexane:acetone, 10:1) to afford **6t** as a white solid (54.5 mg, 45% yield). In an independent experiment, 49.8 mg (41% yield) were obtained, giving an average yield of 43%. **^{1}H NMR (400 MHz, CDCl$_3$)** δ 8.04 – 7.95 (m, 2H), 7.75 – 7.68 (m, 1H), 7.67 – 7.60 (m, 1H), 7.63 – 7.54 (m, 2H), 7.44 (t, *J* = 7.8 Hz, 2H), 7.33 – 7.25 (m, 2H), 7.28 – 7.20 (m, 2H), 7.12 (dd, *J* = 7.2, 2.3 Hz, 1H), 7.11 – 7.02 (m, 1H), 7.00 – 6.96 (m, 1H), 6.95 (dd, *J* = 9.6, 8.4 Hz, 2H), 5.63 (dd, *J* = 9.5 Hz, 1H), 5.16 (app t, *J* = 9.7 Hz, 1H), 4.92 (dd, *J* = 10.2, 3.7 Hz, 1H), 4.84 (d, *J* = 3.7 Hz, 1H), 4.17 (s, 2H), 4.01 (td, *J* = 9.6, 3.1 Hz, 1H), 2.97 (s, 3H), 2.83 (dd, *J* = 14.3, 3.2 Hz, 1H), 2.74 (dd, *J* = 14.3, 9.3 Hz, 1H), 2.06 (s, 3H), 1.89 (s, 3H) ppm. **^{13}C NMR (101 MHz, CDCl$_3$)** δ 170.3, 170.1, 165.7, 159.7 (d, *J* = 245.1 Hz), 143.7, 140.1, 139.8, 133.7, 133.5 (d, *J* = 3.7 Hz), 131.9 (d, *J* = 4.1 Hz), 130.0, 129.7 (d, *J* = 8.0 Hz), 129.2, 128.7, 126.4 (d, *J* = 15.9 Hz), 124.3, 123.8, 123.1, 122.2, 121.8, 115.3 (d, *J* = 22.1 Hz), 96.5, 73.0, 71.4, 70.1, 69.9, 55.0, 37.0, 30.0 (d, *J* = 3.4 Hz), 20.9, 20.7 ppm. **^{19}F NMR (376 MHz, CDCl$_3$)** δ -121.7 ppm. **HRMS (ESI) m/z [M+Na]$^+$:** ($C_{33}H_{31}FNaO_8S$) calcd. 629.1616, found. 629.1613. **IR (neat)** 3060, 2929, 2849, 1747, 1601, 1501, 1452, 1435, 1369, 1218, 1122, 1026, 907, 828, 747, 710, 602 cm^{-1}. **MP** 56.1-58.8 °C.

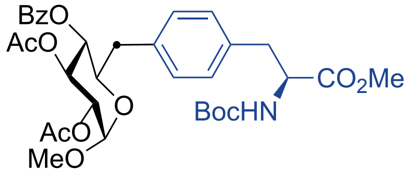

(2*S*,3*R*,4*S*,5*R*,6*R*)-5-(benzoyloxy)-6-(4-((*S*)-2-((*tert*-butoxycarbonyl)amino)-3-methoxy-3-oxopropyl)benzyl)-2-methoxytetrahydro-2*H*-pyran-3,4-diyl diacetate (6u): Following general procedure D, using **3a** (87.9 mg, 0.24 mmol, 1.2 equivalents) and methyl (*S*)-3-(4-bromophenyl)-2-((*tert*-butoxycarbonyl)amino)propanoate (**4v**, 71.6 mg, 0.20 mmol, 1 equivalent), the crude was purified by column chromatography (hexane:EtOAc, 3:1) to afford **6u** as a colourless oil (71.7 mg, 55% yield). In an independent experiment, 60.8 mg (47% yield) were obtained, giving an average yield of 51%.**^{1}H NMR (400 MHz, CDCl3)** δ 8.02 – 8.00 (m, 2H), 7.61 – 7.57 (m, 1H), 7.46 (t, *J* = 7.7 Hz, 2H), 7.12 (d, *J* = 8.0 Hz, 2H), 6.99 (d, *J* = 7.8 Hz, 2H), 5.64 (app t, *J* = 9.8 Hz, 1H), 5.18 (app t, *J* = 9.7 Hz, 1H), 4.93 (d, *J* = 14.0 Hz, 1H), 4.90 – 4.86 (m, 2H), 4.53 – 4.51 (m, 1H), 4.04 (td, *J* = 9.7, 2.9 Hz, 1H), 3.67 (s, 3H), 3.06 – 2.94 (m, 5H), 2.84 (dd, *J* = 14.2, 3.0 Hz, 1H), 2.75 (dd, *J* = 14.2, 9.5 Hz, 1H), 2.06 (s, 3H), 1.89 (s, 3H), 1.41 (s, 9H) ppm. **^{13}C NMR (101 MHz, CDCl3)** δ 172.4, 170.4, 170.1, 165.7, 155.2,

136.5, 134.2, 133.7, 130.0, 129.7, 129.3, 129.2, 128.7, 96.5, 80.1, 73.1, 71.4, 70.2, 70.1, 55.0, 54.5, 52.3, 38.0, 37.3, 28.4, 20.9, 20.8 ppm. **IR (neat)** 3382, 2977, 1727, 1500, 1451, 1367, 1267, 1220, 1043, 1026, 712, 476 cm^{-1}. **HRMS (ESI) m/z $[M+Na]^+$:** ($C_{33}H_{41}NNaO_{12}$) calcd. 666.2521, found: 666.2510. The diastereomeric ratio of **6u** was determined by SFC analysis on a Chiralpak IG-3 column (CO_2/MeOH with a gradient from 100% to 70% CO_2 in 11 min, then maintained at 70% CO_2, column temperature 35 °C, flow rate 1.2 mL/min) with retention time 4.68 min (major) and 4.85 min (minor).

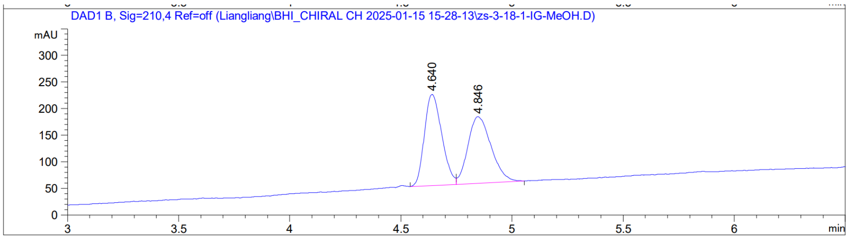

Signal 2: DAD1 B, Sig=210,4 Ref=off

Peak #	RetTime [min]	Type	Width [min]	Area [mAU*s]	Height [mAU]	Area %
1	4.640	BV	0.0852	934.99133	171.37520	50.5670
2	4.846	VV R	0.1119	914.02515	125.61971	49.4330

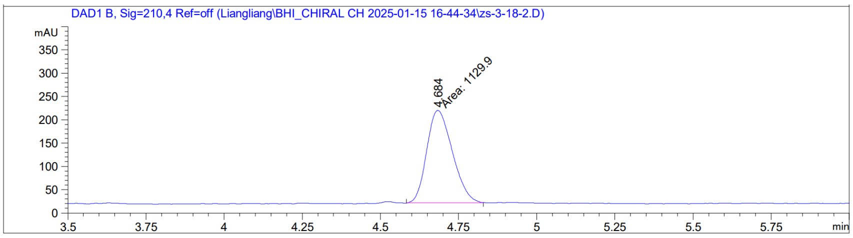

Signal 2: DAD1 B, Sig=210,4 Ref=off

Peak #	RetTime [min]	Type	Width [min]	Area [mAU*s]	Height [mAU]	Area %
1	4.684	MM	0.0950	1129.90112	198.24254	100.0000

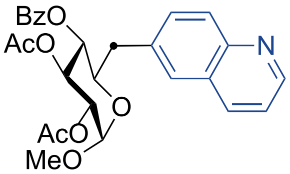

(2*S*,3*R*,4*S*,5*R*,6*R*)-5-(benzoyloxy)-2-methoxy-6-(quinolin-6-ylmethyl)tetrahydro-2*H*-pyran-3,4-diyl diacetate 6v: Following general procedure D, using **3a** (87.9 mg, 0.24 mmol, 1.2 equivalents) and 6-bromoquinoline (**4w**, 41.6 mg, 0.20 mmol, 1 equivalent), the crude was purified by column chromatography (hexane:EtOAc, 2:1 to 1:1) to afford **6v** as a white solid (68.2 mg, 69% yield). In an independent experiment, 66.2 mg (67% yield) were obtained, giving an average yield of 68%. **^{1}H NMR (500 MHz, $CDCl_3$)** δ 8.83 (dd, *J* = 4.2, 1.7 Hz, 1H), 8.06 – 8.01 (m, 1H), 8.00 – 7.94 (m, 3H), 7.62 – 7.58 (m, 2H), 7.58 – 7.53 (m, 1H), 7.41 (t, *J* = 7.8 Hz, 2H), 7.33 (dd, *J* = 8.3, 4.2 Hz, 1H), 5.67 (app t, *J* = 9.8 Hz, 1H), 5.24 (app t, *J* = 9.7

Hz, 1H), 4.95 (dd, J = 10.2, 3.7 Hz, 1H), 4.88 (d, J = 3.7 Hz, 1H), 4.20 (td, J = 9.5, 3.5 Hz, 1H), 3.07 (dd, J = 14.2, 3.5 Hz, 1H), 3.01 (s, 3H), 2.99 (dd, J = 14.2, 9.1 Hz, 1H), 2.05 (s, 3H), 1.89 (s, 3H) ppm. **^{13}C NMR (126 MHz, $CDCl_3$)** δ 170.3, 170.1, 165.7, 150.1, 147.4, 136.0, 135.8, 133.7, 131.3, 129.9, 129.3, 129.0, 128.6, 128.2, 127.9, 121.3, 96.6, 73.1, 71.3, 70.1, 69.8, 55.2, 37.9, 20.9, 20.7 ppm. **HRMS (ESI) m/z [M+Na]$^+$:** calcd. 494.1809, found. 494.1817. **IR (neat)** 2959, 2934, 1750, 1721, 1598, 1504, 1449, 1376, 1265, 1213, 1127, 1069, 1023, 907, 890, 854, 779, 751, 713, 672 cm^{-1}. **MP** 140.8-142.3 °C.

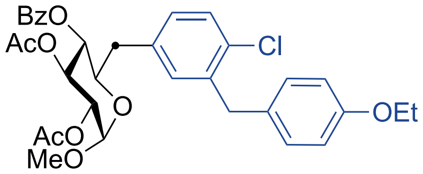

(2*S*,3*R*,4*S*,5*R*,6*R*)-5-(benzoyloxy)-6-(4-chloro-3-(4-ethoxybenzyl)benzyl)-2-methoxytetrahydro-2*H*-pyran-3,4-diyl diacetate (6w): Following general procedure D, using **3a** (87.9 mg, 0.24 mmol, 1.2 equivalents) and 4-bromo-1-chloro-2-(4-ethoxybenzyl)benzene (**4x**, 65.1 mg, 0.20 mmol, 1 equivalent), the crude was purified by column chromatography (hexane:EtOAc, 6:1) to afford **6w** as a white solid (52.6 mg, 43% yield). In an independent experiment, 47.7 mg (39% yield) were obtained, giving an average yield of 41%. **^{1}H NMR (400 MHz, $CDCl_3$)** δ 8.05 – 7.93 (m, 2H), 7.59 (ddt, J = 8.0, 7.0, 1.4 Hz, 1H), 7.50 – 7.42 (m, 2H), 7.22 (d, J = 8.0 Hz, 1H), 7.09 – 6.94 (m, 4H), 6.83 – 6.73 (m, 2H), 5.63 (dd, J = 10.3, 9.4 Hz, 1H), 5.15 (app t, J = 9.7 Hz, 1H), 4.91 (dd, J = 10.2, 3.7 Hz, 1H), 4.84 (d, J = 3.7 Hz, 1H), 4.05 – 3.92 (m, 5H), 2.99 (s, 3H), 2.80 (dd, J = 14.2, 3.3 Hz, 1H), 2.72 (dd, J = 14.2, 9.2 Hz, 1H), 2.07 (s, 3H), 1.89 (s, 3H), 1.39 (t, J = 7.0 Hz, 3H) ppm. **^{13}C NMR (101 MHz, $CDCl_3$)** δ 170.3, 170.1, 165.7, 157.5, 138.9, 136.4, 133.7, 132.4, 132.1, 131.6, 130.0, 129.8, 129.4, 129.2, 128.7, 128.7, 114.5, 96.5, 73.0, 71.3, 70.1, 69.8, 63.5, 55.1, 38.3, 37.2, 20.9, 20.7, 15.0 ppm. **HRMS (ESI) m/z [M+Na]$^+$:** ($C_{33}H_{35}ClNaO_9$) calcd. 633.1862, found. 633.1869. **IR (neat)** 3058, 2927, 2851, 1747, 1611, 1510, 1477, 1451, 1369, 1218, 1117, 1039, 906, 816, 710, 601 cm^{-1}. **MP** 50.0-52.1 °C.

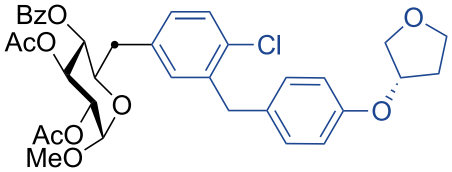

(2*S*,3*R*,4*S*,5*R*,6*R*)-5-(benzoyloxy)-6-(4-chloro-3-(4-(((*S*)-tetrahydrofuran-3-yl)oxy)benzyl)benzyl)-2-methoxytetrahydro-2*H*-pyran-3,4-diyl diacetate (6x): Following general procedure D, using **3a** (87.9 mg, 0.24 mmol, 1.2 equivalents) and (*S*)-3-(4-(5-bromo-2-chlorobenzyl)phenoxy)tetrahydrofuran (**4y**, 73.6 mg, 0.20 mmol, 1 equivalent), the crude was purified by column chromatography (hexane:acetone, 8:1 to 6:1) to afford **6x** as a white solid (57.5 mg, 44% yield). In an independent experiment, 57.7 mg (44% yield) were obtained, giving an average yield of 44%. **^{1}H NMR (400 MHz, $CDCl_3$)** δ 8.02 – 7.93 (m, 2H), 7.59 (ddt,

J = 7.8, 7.0, 1.3 Hz, 1H), 7.48 – 7.43 (m, 2H), 7.23 (d, *J* = 7.9 Hz, 1H), 7.05 (d, *J* = 8.6 Hz, 2H), 7.02 – 6.97 (m, 2H), 6.78 – 6.72 (m, 2H), 5.63 (dd, *J* = 10.2, 9.4 Hz, 1H), 5.14 (app t, *J* = 9.7 Hz, 1H), 4.94 – 4.83 (m, 3H), 4.04 – 3.92 (m, 6H), 3.87 (dd, *J* = 8.3, 4.5 Hz, 1H), 3.00 (s, 3H), 2.81 (dd, *J* = 14.2, 3.3 Hz, 1H), 2.72 (dd, *J* = 14.2, 9.2 Hz, 1H), 2.21 – 2.16 (m, 1H), 2.16 – 2.12 (m, 1H), 2.07 (s, 3H), 1.89 (s, 3H) ppm. **^{13}C NMR (101 MHz, CDCl$_3$)** δ 170.3, 170.1, 165.6, 156.0, 138.7, 136.4, 133.7, 132.4, 132.2, 132.1, 130.0, 129.9, 129.4, 129.1, 128.8, 128.7, 115.4, 96.5, 77.4, 73.3, 73.0, 71.3, 70.0, 69.8, 67.3, 55.1, 38.3, 37.2, 33.1, 20.9, 20.7 ppm. **HRMS (ESI) m/z [M+Na]$^+$:** ($C_{35}H_{37}ClNaO_{10}$) calcd. 675.1967, found. 675.1979. **IR (neat)** 3064, 2929, 2857, 1746, 1610, 1508 1451, 1369, 1231, 1118, 1039, 907, 831, 711, 601 cm^{-1}. **MP** 59.1-61.2 °C.

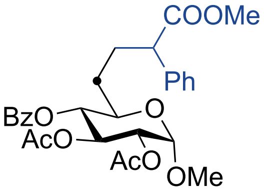

(2*S*,3*R*,4*S*,5*R*,6*R*)-5-(benzoyloxy)-2-methoxy-6-(4-methoxy-4-oxo-3-phenylbutyl)tetrahydro-2*H*-pyran-3,4-diyl diacetate (6y): Following general procedure D, using **3a** (87.9 mg, 0.24 mmol, 1.2 equivalents) and methyl 2-phenylacrylate (**4z**, 32.4 mg, 0.2 mmol, 1 equivalent), the crude was purified by column chromatography (hexane:EtOAc, 8:1 to 6:1) to afford **6y** as a white solid (52.7 mg, 50% yield, d.r. 2/1). In an independent experiment, 53.1 mg (50% yield, d.r. 2/1) were obtained, giving an average yield of 50% and d.r. 2/1. **^{1}H NMR (400 MHz, CDCl$_3$)** δ 7.49 – 7.42 (m, 2H), 7.37 – 7.26 (m, 4H), 7.26 – 7.04 (m, 4H), 5.62 – 5.50 (m, 1H), 4.96 – 4.85 (m, 2H), 4.03 – 3.74 (m, 4H), 3.61 – 3.52 (m, 2H), 3.35 (s, 2H), 3.34 – 3.27 (m, 3H), 3.22 – 3.02 (m, 1H), 2.38 – 2.20 (m, 1H), 2.20 – 2.16 (m, 3H), 2.10 (m, 3H) ppm. **^{13}C NMR (101 MHz, CDCl$_3$)** δ 173.9, 173.4, 170.5, 170.5, 170.1, 170.0, 141.1, 140.8, 139.7, 139.7, 128.9, 128.8, 128.5, 128.4, 128.3, 128.1, 127.6, 127.5, 127.3, 126.1, 126.1, 101.5, 101.1, 97.8, 97.8, 71.7, 71.6, 71.6, 69.8, 69.7, 63.2, 63.1, 63.0, 62.8, 55.5, 55.4, 52.2, 51.9, 46.8, 46.6, 40.9, 39.8, 21.0, 20.9 ppm. **HRMS (ESI) m/z [M+H]$^+$:** ($C_{28}H_{33}O_{10}$) calcd. 529.2068, found. 529.2053. **IR (neat)** 2951, 2926, 1738, 1450, 1435, 1368, 1216, 1155, 1105, 1031, 989, 921, 732, 699 cm^{-1}. **MP** 63.5-64.7 ºC.

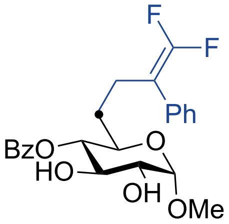

(2*R*,3*S*,4*R*,5*R*,6*S*)-2-(4,4-difluoro-3-phenylbut-3-en-1-yl)-4,5-dihydroxy-6-methoxytetrahydro-2*H*-pyran-3-yl benzoate (6z): Following general procedure D, using **3a** (87.9 mg, 0.24 mmol, 1.2 equivalents) and (3,3,3-trifluoroprop-1-en-2-yl)benzene (**4z'**, 34.4 mg, 0.2 mmol, 1 equivalent). After quenching the reaction, remove the solvent and treat with NaOMe (0.5 equivalents), MeOH (0.1 M), 4 h, the crude was purified by column

chromatography (hexane:EtOAc, 1:1) to afford **6z** as a white solid (43.7 mg, 50% yield). In an independent experiment, 43.9 mg (50% yield) were obtained, giving an average yield of 50%. **^{1}H NMR (400 MHz, $CDCl_3$)** δ 7.46 – 7.40 (m, 2H), 7.35 – 7.27 (m, 5H), 7.26 – 7.21 (m, 1H), 7.18 (m, 2H), 4.69 (d, J = 3.9 Hz, 1H), 3.80 (dd, J = 11.1, 6.1 Hz, 1H), 3.74 – 3.63 (m, 2H), 3.60 – 3.48 (m, 3H), 3.31 (s, 3H), 3.21 – 3.13 (m, 1H), 3.09 (ddd, J = 14.4, 2.3, 1.1 Hz, 1H), 2.14 (d, J = 9.0 Hz, 1H), 1.59 (d, J = 2.2 Hz, 1H) ppm. **^{13}C NMR (101 MHz, $CDCl_3$)** δ 154.8 (t, J = 290.1 Hz), 140.8, 134.5 (dd, J = 4.5, 2.7 Hz), 128.6 (t, J = 2.7 Hz), 128.4, 128.4, 128.0, 127.5, 126.1, 102.0 (t , J = 3.4 Hz), 100.0, 88.0 (dd, J = 20.4, 17.4 Hz), 72.8, 72.6, 72.3, 63.5, 62.9, 55.6, 36.2 ppm. **^{19}F NMR (376 MHz, $CDCl_3$)** δ -89.4 (d, J = 35.2 Hz), -90.4 (d, J = 35.1 Hz) ppm. **HRMS (ESI) m/z [M+Na]$^+$:** ($C_{23}H_{24}F_2NaO_6$) calcd. 457.1433, found. 457.1443. **IR (neat)** 3404, 2925, 2851, 1730, 1448, 1240, 1124, 1056, 1022, 984, 935, 746, 764, 696 cm^{-1}. **MP** 47.2-48.3 °C.

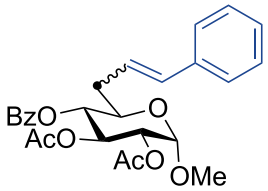

(2*S*,3*R*,4*S*,5*R*,6*R*)-5-(benzoyloxy)-2-methoxy-6-(3-phenylallyl)tetrahydro-2*H*-pyran-3,4-diyl diacetate (6za): Following general procedure D, using **3a** (87.9 mg, 0.24 mmol, 1.2 equivalents) and (2-bromovinyl)benzene (**4za**, 27 μL, 0.2 mmol, 1 equivalent), the crude was purified by column chromatography (hexane:EtOAc, 8:1 to 6:1) to afford **6za** as a white solid (35.7 mg, 38% yield). In an independent experiment, 34.9 mg (38% yield) were obtained, giving an average yield of 38%. **^{1}H NMR (400 MHz, $CDCl_3$)** δ 8.04 – 7.91 (m, 2H), 7.60 – 7.53 (m, 1H), 7.46 – 7.39 (m, 2H), 7.29 – 7.08 (m, 5H), 6.56 – 6.37 (m, 1H), 6.27 – 5.71 (m, 1H), 5.66 (td, J = 9.6, 7.5 Hz, 1H), 5.25 – 5.12 (m, 1H), 5.01 – 4.91 (m, 2H), 4.07 – 3.96 (m, 1H), 3.47 – 3.40 (m, 3H), 2.70 – 2.40 (m, 2H), 2.09 (s, 3H), 1.90 (s, 3H) ppm. **^{13}C NMR (101 MHz, $CDCl_3$)** δ 170.4, 170.4, 170.1, 165.6, 165.5, 137.4, 137.0, 133.6, 133.5, 133.3, 131.7, 130.0, 130.0, 129.3, 128.7, 128.7, 128.6, 128.6, 128.2, 127.3, 126.9, 126.8, 126.2, 124.8, 96.8, 96.8, 72.7, 71.4, 71.4, 70.1, 69.2, 68.9, 55.5, 55.4, 35.0, 30.2, 29.8, 20.9, 20.8 ppm. **HRMS (ESI) m/z [M+Na]$^+$:** ($C_{26}H_{28}NaO_8$) calcd. 491.1676, found. 491.1669. **IR (neat)** 2922, 2852, 1746, 1726, 1451, 1369, 1267, 1219, 1126, 1094, 1026, 908, 745, 711, 603 cm^{-1}. **MP** 48.3-49.2 °C.

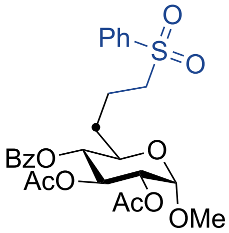

(2*S*,3*R*,4*S*,5*R*,6*R*)-5-(benzoyloxy)-2-methoxy-6-(3-(phenylsulfonyl)propyl)tetrahydro-2*H*-pyran-3,4-diyl diacetate (6zb): Following general procedure D, using **3a** (87.9 mg, 0.24 mmol, 1.2 equivalents) and (vinylsulfonyl)benzene (**4zb**, 33.6 mg, 0.2 mmol, 1 equivalent), the

crude was purified by column chromatography (hexane:EtOAc, 8:1 to 6:1) to afford **6zb** as a white solid (37.7 mg, 35% yield). In an independent experiment, 37.9 mg (35% yield) were obtained, giving an average yield of 35%. **^{1}H NMR (500 MHz, CDCl$_3$)** δ 7.84 – 7.79 (m, 2H), 7.69 – 7.64 (m, 1H), 7.58 – 7.54 (m, 2H), 7.31 (s, 5H), 5.49 (app t, *J* = 9.7 Hz, 1H), 4.88 (d, *J* = 3.8 Hz, 1H), 4.84 (dd, *J* = 9.9, 3.8 Hz, 1H), 3.96 – 3.91 (m, 1H), 3.79 – 3.72 (m, 2H), 3.65 (app t, *J* = 9.4 Hz, 1H), 3.30 (s, 3H), 2.94 (ddd, *J* = 14.0, 12.5, 4.0 Hz, 1H), 2.81 (ddd, *J* = 13.9, 12.4, 4.4 Hz, 1H), 2.65 – 2.56 (m, 1H), 2.26 – 2.21 (m, 1H), 2.10 (s, 3H), 2.09 (s, 3H) ppm. **^{13}C NMR (126 MHz, CDCl$_3$)** δ 170.4, 169.9, 140.0, 138.9, 134.0, 129.6, 128.9, 128.6, 128.0, 125.5, 100.4, 97.8, 71.6, 71.4, 69.5, 63.1, 63.0, 55.5, 29.2, 20.9, 20.9 ppm. **HRMS (ESI) m/z [M+Na]$^+$:** ($C_{26}H_{30}NaO_{10}S$) calcd. 557.1452, found. 557.1442. **IR (neat)** 2925, 1743, 1448, 1370, 1306, 1218, 1151, 1031, 919, 730, 547 cm^{-1}. **MP** 61.3-63.2 °C.

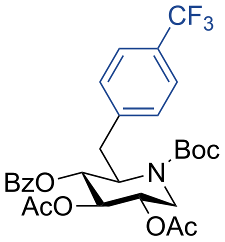

(3*S*,4*R*,5*R*,6*R*)-5-(benzoyloxy)-1-(*tert*-butoxycarbonyl)-6-(4-(trifluoromethyl)benzyl)piperidine-3,4-diyl diacetate (7a): Following general procedure D, using **3ad** (104.5 mg, 0.24 mmol, 1.2 equivalents), 1-bromo-4-(trifluoromethyl)benzene (**4a**, 28 µL, 0.2 mmol, 1 equivalent) and benzene:*tert*-amyl alcohol (1:1, 4.0 mL, 0.05 M), the crude was purified by column chromatography (hexane: EtOAc, 5:1 to 3:1) to afford **7a** as a white solid (51.0 mg, 44% yield). In an independent experiment, 48.8 mg (42% yield) were obtained, giving an average yield of 43%. **^{1}H NMR (400 MHz, MeOD)** δ 8.08 (d, *J* = 7.7 Hz, 2H), 7.63 (t, *J* = 7.5 Hz, 1H), 7.58 (d, *J* = 8.0 Hz, 2H), 7.50 – 7.41 (m, 4H), 5.15 (dt, *J* = 2.9, 1.4 Hz, 1H), 5.08 (app t, *J* = 2.5 Hz, 1H), 4.87 (s, 1H), 4.80 (s, 1H), 4.35 (d, *J* = 15.4 Hz, 1H), 3.56 (dd, *J* = 15.3, 1.9 Hz, 1H), 3.40 – 3.32 (m, 1H), 3.12 (s, 1H), 2.21 (s, 3H), 2.03 (s, 3H), 0.93 (s, 9H) ppm. **^{13}C NMR (101 MHz, MeOD)** δ 171.3, 170.5, 166.4, 156.8, 144.4, 134.8, 131.0, 130.9, 130.9, 129.9 (q, *J* = 32.4 Hz), 129.6, 126.2 (q, *J* = 3.9 Hz), 125.8 (q, *J* = 270.9 Hz), 81.3, 71.2, 68.8, 68.5, 57.2, 37.8, 35.3, 28.0, 21.0, 20.8 ppm. **^{19}F NMR (376 MHz, MeOD)** δ -63.9 ppm. **HRMS (ESI) m/z [M+Na]$^+$:** ($C_{29}H_{32}F_3NNaO_8$) calcd. 602.1972, found. 602.1958. **IR (neat):** ν_{max} (cm^{-1}) 2976, 2932, 1721, 1693, 1618, 1416, 1367, 1216, 1160, 1110, 1066, 820, 712. **MP** 58.6-60.2 °C.

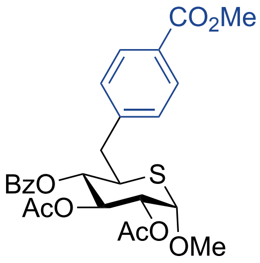

(2*S*,3*R*,4*S*,5*S*,6*R*)-5-(benzoyloxy)-2-methoxy-6-(4-(methoxycarbonyl)benzyl)tetrahydro-2*H*-thiopyran-3,4-diyl diacetate (7b): Following general procedure D, using **3ae** (91.8 mg, 0.24 mmol, 1.2 equivalents), methyl 4-bromobenzoate (**4c**, 43 mg, 0.20 mmol, 1.0 equivalent), the crude was purified by column chromatography (DCM:MeOH, 100:1) to afford **7b** as a white solid (48.9 mg, 46% yield). In an independent experiment, 44.7 mg (42% yield) were obtained, giving an average yield of 44%. **^{1}H NMR (400 MHz, $CDCl_3$)** δ 7.96 (dd, *J* = 8.4, 1.3 Hz, 2H), 7.92 – 7.86 (m, 2H), 7.59 – 7.54 (m, 1H), 7.45 – 7.40 (m, 2H), 7.22 (d, *J* = 8.3 Hz, 2H), 5.69 (app t, *J* = 9.9 Hz, 1H), 5.49 (dd, *J* = 10.6, 9.5 Hz, 1H), 5.24 (dd, *J* = 10.3, 2.9 Hz, 1H), 4.60 (d, *J* = 2.9 Hz, 1H), 3.87 (s, 3H), 3.54 (td, *J* = 10.4, 4.6 Hz, 1H), 3.29 (s, 3H), 3.12 (dd, *J* = 14.4, 4.6 Hz, 1H), 2.72 (dd, *J* = 14.4, 10.2 Hz, 1H), 2.06 (s, 3H), 1.82 (s, 3H) ppm. **^{13}C NMR (101 MHz, $CDCl_3$)** δ 170.3, 169.8, 167.0, 165.8, 143.0, 133.6, 130.0, 129.8, 129.0, 128.9, 128.8, 128.7, 81.2, 76.4, 75.1, 70.6, 56.5, 52.1, 40.8, 36.2, 21.0, 20.6 ppm. **HRMS (ESI) m/z [M+Na]$^+$:** ($C_{26}H_{28}NaO_9S$) calcd. 539.1346, found. 539.1343. **IR (neat)** 2952, 2854, 1718, 1748, 1609, 1434, 1370, 1229, 1179, 1159, 1102, 1082, 1042, 1023, 961, 904, 766, 708, 601 cm^{-1}. **MP** 132.8-135.0 °C.

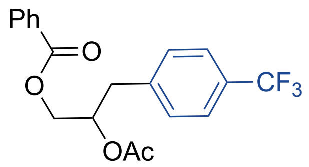

2-acetoxy-3-(4-(trifluoromethyl)phenyl)propyl benzoate (8a): Following general procedure D, using **3aa** (133.3 mg, 0.6 mmol, 3.0 equivalents), 1-bromo-4-(trifluoromethyl)benzene (**4a**, 28 μL, 0.2 mmol, 1 equivalent) and benzene:*tert*-amyl alcohol (1:1, 4.0 mL, 0.05 M), the crude was purified by column chromatography (hexane: EtOAc, 10:1) to afford **8a** as a colourless oil (32.9 mg, 45% yield). In an independent experiment, 25.7 mg (35% yield) were obtained, giving an average yield of 40%. **^{1}H NMR (400 MHz, $CDCl_3$)** ^{1}H NMR (400 MHz, $CDCl_3$) δ 8.08 – 7.96 (m, 2H), 7.64 – 7.53 (m, 3H), 7.50 – 7.42 (m, 2H), 7.37 (d, *J* = 7.9 Hz, 2H), 5.51 – 5.40 (m, 1H), 4.47 (dd, *J* = 11.9, 3.7 Hz, 1H), 4.30 (dd, *J* = 11.9, 5.9 Hz, 1H), 3.08 (d, *J* = 3.0 Hz, 1H), 3.07 (d, *J* = 2.5 Hz, 1H), 2.04 (s, 3H) ppm. **^{13}C NMR (101 MHz, $CDCl_3$)** δ 170.4, 166.2, 140.6 (q, *J* = 1.2 Hz), 129.8, 129.8, 129.8, 129.4 (q, *J* = 32.4 Hz), 128.6, 125.7 (q, *J* = 3.7 Hz), 124.3 (d, *J* = 271.6 Hz), 71.7, 64.7, 37.2, 21.1 ppm. **^{19}F NMR (376 MHz, $CDCl_3$)** δ -62.6 ppm. **HRMS (ESI) m/z [M+Na]$^+$:** ($C_{19}H_{17}F_3NaO_4$) calcd. 389.0971, found. 389.0975. **IR (neat)** 3064, 2957, 1721, 1619, 1452, 1374, 1323, 1271, 1228, 1162, 1112, 1065, 1018, 955, 847, 710, 636, 605 cm^{-1}.

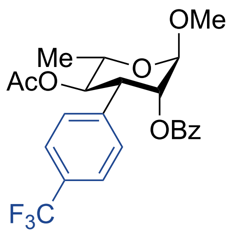

(2*R*,3*R*,4*R*,5*R*,6*S*)-5-acetoxy-2-methoxy-6-methyl-4-(4-(trifluoromethyl)phenyl)tetrahydro-2*H*-pyran-3-yl benzoate (9a): Following general procedure D, using **3ab** (74.0 mg, 0.24 mmol, 1.2 equivalents) and 1-bromo-4-(trifluoromethyl)benzene (**4a**, 28 µL, 0.2 mmol, 1 equivalent), the crude was purified by column chromatography (hexane: acetone, 10:1) to afford **9a** as a white solid (38.9 mg, 43% yield). In an independent experiment, 32.6 mg (36% yield) were obtained, giving an average yield of 39%. **^{1}H NMR (400 MHz, CDCl$_3$)** δ 8.00 (dd, *J* = 8.3, 1.4 Hz, 2H), 7.65 – 7.55 (m, 1H), 7.53 – 7.34 (m, 6H), 5.64 (dd, *J* = 11.5, 9.4 Hz, 1H), 5.21 (dd, *J* = 2.9, 1.6 Hz, 1H), 4.76 (d, *J* = 1.6 Hz, 1H), 4.00 (dq, *J* = 9.4, 6.3 Hz, 1H), 3.67 (dd, *J* = 11.5, 2.9 Hz, 1H), 3.49 (s, 3H), 1.82 (s, 3H), 1.30 (d, *J* = 6.3 Hz, 3H) ppm. **^{13}C NMR (101 MHz, CDCl$_3$)** δ 170.4, 165.5, 140.9 (q, *J* = 1.2 Hz), 133.6, 129.8, 129.7 (q, *J* = 32.6 Hz), 129.4, 129.3, 128.8, 125.5 (q, *J* = 3.9 Hz), 124.2 (q, *J* = 272.1 Hz), 97.5, 73.9, 70.7, 67.4, 55.3, 45.7, 20.8, 17.9 ppm. **^{19}F NMR (376 MHz, CDCl$_3$)** δ -62.7 ppm. **HRMS (ESI) m/z [M+Na]$^+$:** ($C_{23}H_{23}F_3NaO_6$) calcd. 475.1339, found. 475.1330. **IR (neat)** 3356, 2960, 2920, 2851, 1732, 1619, 1423, 1452, 1374, 1322, 1232, 1066, 978, 831, 705 cm^{-1}. **MP** 129.8-132.2 °C.

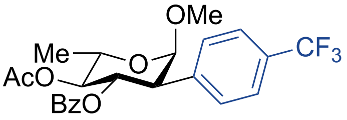

(2*S*,3*S*,4*S*,5*S*,6*R*)-3-acetoxy-6-methoxy-2-methyl-5-(4-(trifluoromethyl)phenyl)tetrahydro-2*H*-pyran-4-yl benzoate (9a′): Following general procedure D, using **3ab** (74.0 mg, 0.24 mmol, 1.2 equivalents) and 1-bromo-4-(trifluoromethyl)benzene (**4a**, 28 µL, 0.2 mmol, 1 equivalent), the crude was purified by column chromatography (hexane: acetone, 10:1) to afford **9a′** (4.7 mg, 5% yield). In an independent experiment, 4.6 mg (5% yield) were obtained, giving an average yield of 5%. **^{1}H NMR (500 MHz, CDCl$_3$)** δ 7.83 – 7.77 (m, 2H), 7.54 – 7.46 (m, 5H), 7.39 – 7.32 (m, 2H), 6.11 (dd, *J* = 11.7, 9.1 Hz, 1H), 5.07 (dd, *J* = 9.8, 9.1 Hz, 1H), 4.76 (d, *J* = 3.1 Hz, 1H), 4.12 (dq, *J* = 9.8, 6.2 Hz, 1H), 3.43 (dd, *J* = 11.7, 3.2 Hz, 1H), 3.34 (s, 3H), 1.94 (s, 3H), 1.31 (d, *J* = 6.3 Hz, 3H) ppm. **^{13}C NMR (126 MHz, CDCl$_3$)** δ 170.3, 165.9, 139.5 (q, *J* = 1,4 Hz), 133.2, 129.9, 129.6, 129.5, 128.5, 125.2 (q, *J* = 3.9 Hz), 124.3 (q, *J* = 271.8 Hz), 100.9, 75.7, 71.1, 65.9, 55.4, 52.5, 20.8, 17.8 ppm. **^{19}F NMR (376 MHz, CDCl$_3$)** δ -62.7 ppm. **HRMS (ESI) m/z [M+Na]$^+$:** ($C_{23}H_{23}F_3NaO_6$) calcd. 475.1339, found. 475.1336. **IR (neat)** 3360, 2966, 2920, 2851, 1731, 1620, 1423, 1455, 1374, 1322, 1232, 1066, 978, 831, 705 cm^{-1}.

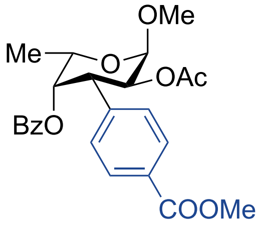

Methyl 4-((2*R*,3*S*,4*R*,5*S*,6*S*)-3-acetoxy-5-(benzoyloxy)-2-methoxy-6-methyltetrahydro-2*H*-pyran-4-yl)benzoate (9b): Following general procedure D, using **3ac** (74.0 mg, 0.24

mmol, 1.2 equivalents) and methyl 4-bromobenzoate (**4c**, 43 mg, 0.20 mmol, 1 equivalent), the crude was purified by column chromatography (hexane:EtOAc, 8:1) to afford **9b** (17.7 mg, 20% yield, d.r. 6/1) as a white solid. In an independent experiment, 18.2 mg (20% yield, d.r. 6/1) were obtained, giving an average yield of 20% and d.r. 6/1. **^{1}H NMR (400 MHz, CDCl$_3$)** δ 8.02 – 7.78 (m, 4H), 7.62 – 7.38 (m, 3H), 7.24 (d, *J* = 8.4 Hz, 2H), 5.79 (dd, *J* = 12.2, 3.5 Hz, 1H), 5.47 – 5.39 (m, 1H), 5.06 (d, *J* = 3.5 Hz, 1H), 4.40 – 4.23 (m, 1H), 3.83 (s, 3H), 3.84 – 3.74 (m, 1H), 3.52 (s, 3H), 1.85 (s, 3H), 1.17 (d, *J* = 6.5 Hz, 3H) ppm. **^{13}C NMR (101 MHz, CDCl$_3$)** δ 170.7, 166.9, 165.5, 142.0, 133.5, 129.9, 129.3, 129.3, 129.2, 128.7, 128.6, 128.4, 97.0, 74.4, 67.9, 66.0, 55.6, 52.1, 44.6, 20.8, 16.5 ppm. **HRMS (ESI) m/z [M+H]$^+$:** ($C_{24}H_{27}O_8$) calcd. 443.1700, found. 443.1689. **IR (neat)** 2952, 2926, 2849, 1719, 1613, 1451, 1436, 1367, 1266, 1230, 1190, 1107, 1051, 1024, 951, 710 cm^{-1}. **MP** 52.5-53.6 °C.

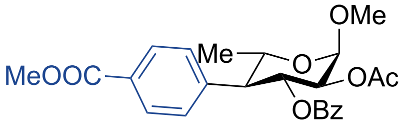

Methyl 4-((2*S*,3*S*,4*R*,5*S*,6*R*)-5-acetoxy-4-(benzoyloxy)-6-methoxy-2-methyltetrahydro-2*H*-pyran-3-yl)benzoate (9b′): Following general procedure D, using **3ac** (74.0 mg, 0.24 mmol, 1.2 equivalents) and methyl 4-bromobenzoate (**4c**, 43 mg, 0.20 mmol, 1 equivalent), the crude was purified by column chromatography (hexane:EtOAc, 8:1) to afford **9b′** (8.7 mg, 10% yield) as a white solid. In an independent experiment, 8.5 mg (10% yield) were obtained, giving an average yield of 10%. **^{1}H NMR (400 MHz, CDCl$_3$)** δ 7.96 – 7.89 (m, 2H), 7.81 – 7.74 (m, 2H), 7.50 – 7.44 (m, 1H), 7.36 – 7.30 (m, 4H), 6.01 (dd, *J* = 11.0, 9.9 Hz, 1H), 5.15 (dd, *J* = 9.9, 3.6 Hz, 1H), 5.00 (d, *J* = 3.5 Hz, 1H), 4.19 (dq, *J* = 10.3, 6.2 Hz, 1H), 3.85 (s, 3H), 3.50 (s, 3H), 2.93 (app t, *J* = 10.7 Hz, 1H), 1.95 (s, 3H), 1.04 (d, *J* = 6.2 Hz, 3H) ppm. **^{13}C NMR (101 MHz, CDCl$_3$)** δ 170.7, 166.9, 165.2, 142.4, 133.1, 130.1, 129.6, 129.6, 128.7, 128.4, 97.7, 72.8, 71.1, 68.0, 55.8, 55.6, 52.2, 21.0, 19.0 ppm. **HRMS (ESI) m/z [M+H]$^+$:** ($C_{24}H_{27}O_8$) calcd. 443.1700, found. 443.1695. **IR (neat)** 2924, 2850, 1720, 1611, 1436, 1369, 1268, 1229, 1192, 1095, 1044, 924, 848, 770, 707 cm^{-1}. **MP** 63.6-64.5 °C.

Unsuccessful substrates

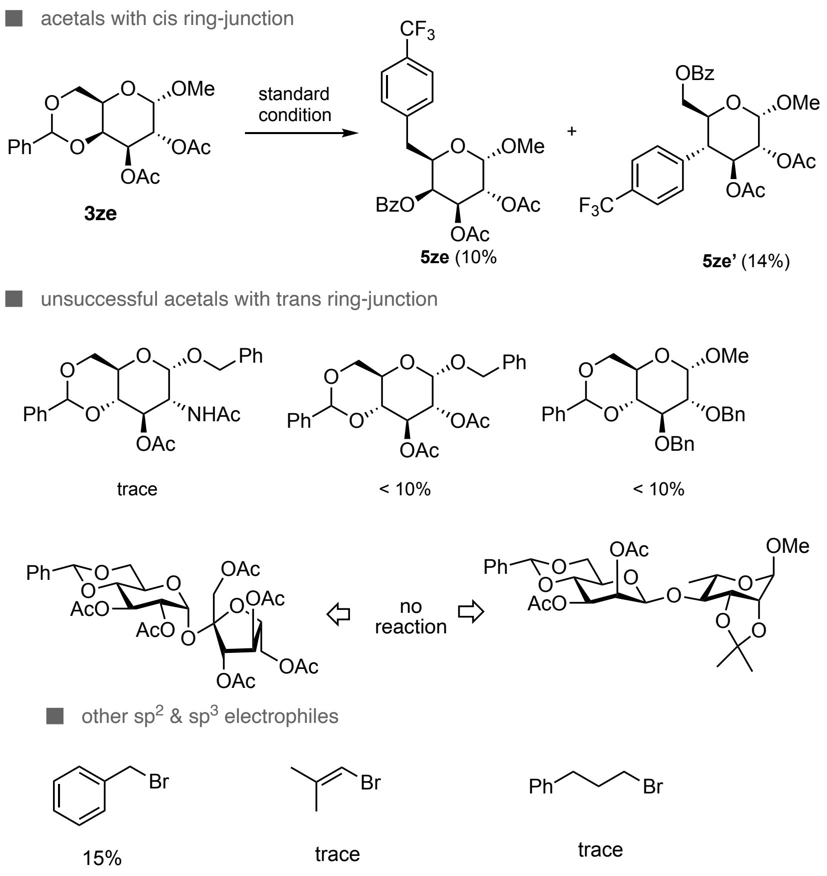

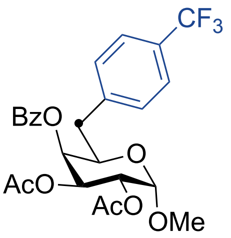

(2*S*,3*R*,4*S*,5*S*,6*R*)-5-(benzoyloxy)-2-methoxy-6-(4-(trifluoromethyl)benzyl)tetrahydro-2*H*-pyran-3,4-diyl diacetate (5ze): Following general procedure D, using **3ze** (87.9 mg, 0.24 mmol, 1.2 equivalents) and 1-bromo-4-(trifluoromethyl)benzene (**4a**, 28 μL, 0.2 mmol, 1 equivalent), the crude was purified by column chromatography (hexane:EtOAc, 8:1 to 6:1) to afford **5ze** as a white solid (9.7 mg, 10% yield). In an independent experiment, 9.9 mg (10% yield) were obtained, giving an average yield of 10%. **^{1}H NMR (400 MHz, CDCl$_3$)** δ 8.21 – 8.12 (m, 2H), 7.69 – 7.60 (m, 1H), 7.56 – 7.47 (m, 4H), 7.31 (d, *J* = 7.9 Hz, 2H), 5.66 (dd, *J* = 3.4, 1.2 Hz, 1H), 5.43 (dd, *J* = 10.9, 3.4 Hz, 1H), 5.26 (dd, *J* = 10.9, 3.6 Hz, 1H), 5.02 (d, *J* = 3.6 Hz, 1H), 4.27 – 4.17 (m, 1H), 3.10 (s, 3H), 2.94 (dd, *J* = 13.9, 9.1 Hz, 1H), 2.82 (dd, *J* = 14.0, 4.3 Hz, 1H), 2.07 (s, 3H), 1.94 (s, 3H) ppm. **^{13}C NMR (101 MHz, CDCl$_3$)** δ 170.6, 170.3, 166.1, 141.8 (q, *J* = 1,4 Hz), 133.8, 130.1, 129.8, 129.4, 129.2 (q, *J* = 32.5 Hz), 128.8, 125.4 (q, *J* = 3.7 Hz), 124.3 (q, *J* = 271.8 Hz), 97.3, 70.9, 69.5, 68.6, 68.2, 55.4, 37.1, 21.0, 20.8 ppm. **^{19}F NMR (376 MHz, CDCl$_3$)** δ -62.5 ppm. **HRMS (ESI) m/z [M+Na]$^{+}$:** ($C_{25}H_{25}F_3NaO_8$) calcd.

533.1394, found. 533.1398. **IR (neat)** 2950, 2845, 1745, 1620, 1455, 1370, 1325, 1220, 1162, 1115, 1026, 907, 851, 819, 710, 639, 601 cm^{-1}.

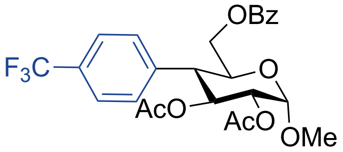

(2*S*,3*R*,4*S*,5*R*,6*S*)-6-((benzoyloxy)methyl)-2-methoxy-5-(4-(trifluoromethyl)phenyl)tetrahydro-2*H*-pyran-3,4-diyl diacetate (5ze′): Following general procedure D, using **3ze** (87.9 mg, 0.24 mmol, 1.2 equivalents) and 1-bromo-4-(trifluoromethyl)benzene (**4a**, 28 μL, 0.2 mmol, 1 equivalent), the crude was purified by column chromatography (hexane:EtOAc, 8:1 to 6:1) to afford **5ze′** as a white solid (14.3 mg, 14% yield). In an independent experiment, 13.9 mg (14% yield) were obtained, giving an average yield of 14%. **^{1}H NMR (400 MHz, $CDCl_3$)** δ 8.03 – 7.91 (m, 2H), 7.61 – 7.52 (m, 3H), 7.49 – 7.42 (m, 2H), 7.37 (d, *J* = 7.9 Hz, 2H), 5.82 (dd, *J* = 11.0, 9.6 Hz, 1H), 5.05 (d, *J* = 3.6 Hz, 1H), 5.02 (dd, *J* = 9.6, 3.6 Hz, 1H), 4.34 (ddd, *J* = 11.0, 5.2, 2.9 Hz, 1H), 4.24 (dd, *J* = 12.0, 2.9 Hz, 1H), 4.09 (dd, *J* = 12.0, 5.2 Hz, 1H), 3.50 (s, 3H), 3.19 (t, *J* = 11.0 Hz, 1H), 2.09 (s, 3H), 1.75 (s, 3H) ppm. **^{13}C NMR (75 MHz, $CDCl_3$)** δ 170.6, 169.5, 166.1, 139.9 (q, *J* = 1.3 Hz), 133.4, 130.4 (q, *J* = 32.8 Hz), 129.7, 129.0, 128.6, 126.0 (q, *J* = 3.7 Hz), 124.0 (q, *J* = 272.3 Hz), 97.7, 72.4, 70.3, 69.8, 64.4, 55.7, 50.2, 21.0, 20.5 ppm. **^{19}F NMR (376 MHz, $CDCl_3$)** δ -62.5 ppm. **HRMS (ESI) m/z $[M+Na]^+$:** ($C_{25}H_{25}F_3NaO_8$) calcd. 533.1394, found. 533.1394. **IR (neat)** 2948, 2850, 1755, 1620, 1455, 1360, 1325, 1222, 1162, 1111, 1026, 910, 852, 819, 710, 639, 598 cm^{-1}.

Control experiments

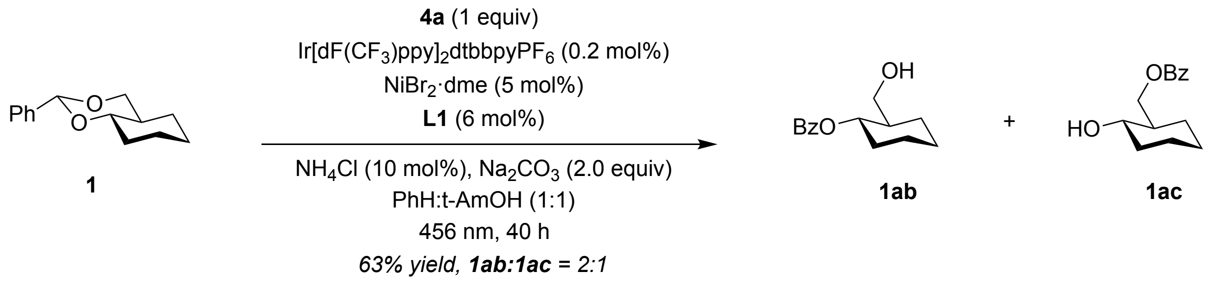

Reaction of 1 with 4a: To an oven dried 12 mL reaction vial equipped with a Teflon-coated stirring bar, {Ir[dF(CF_3)ppy]$_2$(dtbbpy)}PF_6 (0.4 mg, 0.4 μmol, 0.2 mol%) was added as a 2 mg/mL solution in dichloromethane. Solvent was evaporated under vacuum for 30 minutes and, then 4,7-dimethyl-1,10-phenanthroline (**L1,** 2.5 mg, 0.12 mmol, 6 mol%) and (2*R*,4a*R*,8a*S*)-2-phenylhexahydro-4*H*-benzo[*d*][1,3]dioxine (**1**, 52.4 mg, 0.24 mmol, 1.2 equivalents) were added. Subsequently, the reaction vial was brought inside a nitrogen-filled glovebox and $NiBr_2$·dme (3.1, 0.01 mmol, 5 mol%), NH_4Cl (1.1, 0.02 mmol, 10 mol%) and Na_2CO_3 (42.4 mg, 0.4 mmol, 2 equivalents) were added. Then, the vial was closed and taken outside the glovebox where benzene:*tert*-amyl alcohol (1:1, 2.0 mL, 0.1 M) and 1-bromo-4-(trifluoromethyl)benzene (**4a**, 28 μL, 0.2 mmol, 1 equivalent) were added sequentially via syringe. The reaction was stirred at 650 rpm under 456 nm light irradiation for 40 h using the UFO photorreactor. After the reaction was completed, the crude was filtered through a short celite pad eluting with ethyl acetate and concentrated under vacuum. The crude was analyzed by ^{1}H NMR using 1,3,5-trimethoxybenzene as internal standard. By crude ^{1}H-NMR analysis, no arylation took place but rather resulted in the formation of **1ab** and **1ac** in a combined 63% yield, the formation of which might be rationalized via oxidation events.

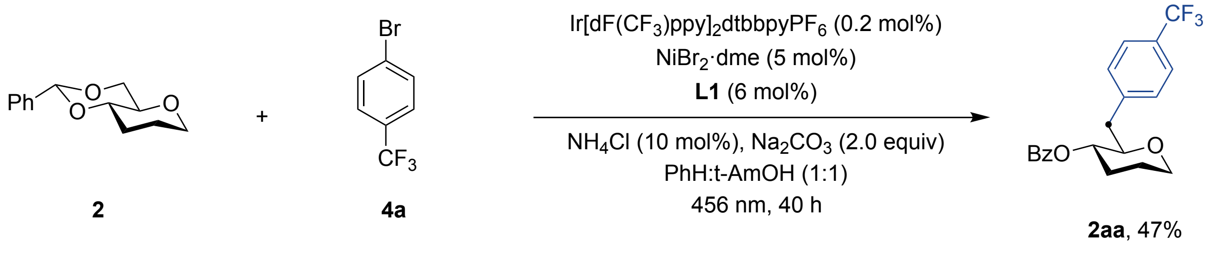

Reaction of 2 with 4a: To an oven dried 12 mL reaction vial equipped with a Teflon-coated stirring bar, {Ir[dF(CF_3)ppy]$_2$(dtbbpy)}PF_6 (0.4 mg, 0.4 μmol, 0.2 mol%) was added as a 2 mg/mL solution in dichloromethane. Solvent was evaporated under vacuum for 30 minutes and, then 4,7-dimethyl-1,10-phenanthroline (**L1,** 2.5 mg, 0.12 mmol, 6 mol%) and (2*R*,4a*R*,8a*S*)-2-phenylhexahydropyrano[3,2-*d*][1,3]dioxine (**2**, 52.9 mg, 0.24 mmol, 1.2 equivalents) were added. Subsequently, the reaction vial was brought inside a nitrogen-filled glovebox and $NiBr_2$·dme (3.1, 0.01 mmol, 5 mol%), NH_4Cl (1.1, 0.02 mmol, 10 mol%) and Na_2CO_3 (42.4 mg, 0.4 mmol, 2 equivalents) were added. Then, the vial was closed and taken outside the glovebox where benzene:*tert*-amyl alcohol (1:1, 2.0 mL, 0.1 M) and 1-bromo-4-

(trifluoromethyl)benzene (**4a**, 28 μL, 0.2 mmol, 1 equivalent) were added sequentially via syringe. The reaction was stirred at 650 rpm under 456 nm light irradiation for 40 h using the UFO photorreactor. After the reaction was completed, the crude was filtered through a short celite pad eluting with ethyl acetate and concentrated under vacuum. The crude was analyzed by ^{1}H NMR using 1,3,5-trimethoxybenzene as internal standard, giving rise to **2aa** in 47% yield, with traces, if any of oxidation byproducts.

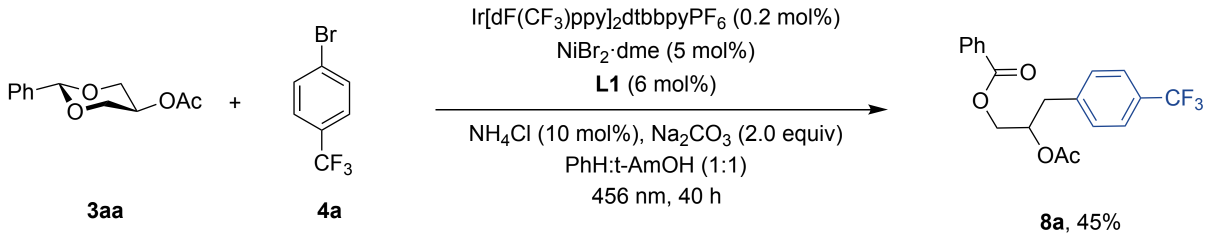

Reaction of 3aa with 4a: To an oven dried 12 mL reaction vial equipped with a Teflon-coated stirring bar, {Ir[dF(CF_3)ppy]$_2$(dtbbpy)}PF_6 (0.4 mg, 0.4 μmol, 0.2 mol%) was added as a 2 mg/mL solution in dichloromethane. Solvent was evaporated under vacuum for 30 minutes and, then 4,7-dimethyl-1,10-phenanthroline (2.5 mg, 0.12 mmol, 6 mol%) and (2*R*,5*R*)-2-phenyl-1,3-dioxan-5-yl acetate (**3aa**, 133.3 mg, 0.6 mmol, 3.0 equivalents) were added. Subsequently, the reaction vial was brought inside a nitrogen-filled glovebox and $NiBr_2$·dme (3.1, 0.01 mmol, 5 mol%), NH_4Cl (1.1, 0.02 mmol, 10 mol%) and Na_2CO_3 (42.4 mg, 0.4 mmol, 2 equivalents) were added. Then, the vial was closed and taken outside the glovebox where benzene:*tert*-amyl alcohol (1:1, 4.0 mL, 0.05 M) and 1-bromo-4-(trifluoromethyl)benzene (**4a**, 28 μL, 0.2 mmol, 1 equivalent) were added sequentially via syringe. The reaction was stirred at 650 rpm under 456 nm light irradiation for 40 h using the UFO photorreactor. After the reaction was completed, the crude was filtered through a short celite pad eluting with ethyl acetate and concentrated under vacuum. The crude was analyzed by ^{1}H NMR using 1,3,5-trimethoxybenzene as internal standard. By crude ^{1}H-NMR analysis, the product was obtained with a 45% NMR yield.

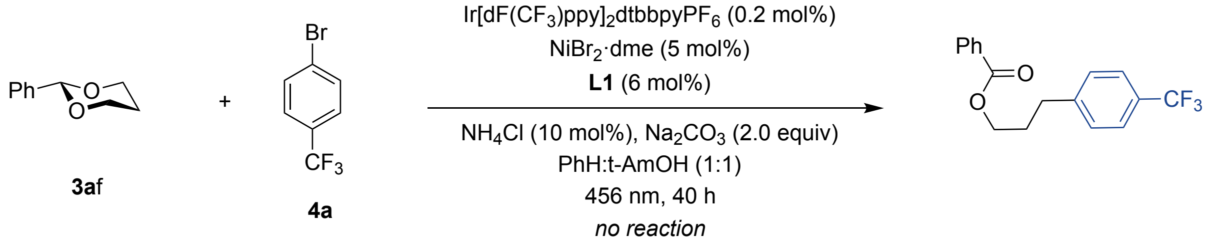

Reaction of 3af with 4a: To an oven dried 12 mL reaction vial equipped with a Teflon-coated stirring bar, {Ir[dF(CF_3)ppy]$_2$(dtbbpy)}PF_6 (0.4 mg, 0.4 μmol, 0.2 mol%) was added as a 2 mg/mL solution in dichloromethane. Solvent was evaporated under vacuum for 30 minutes and, then 4,7-dimethyl-1,10-phenanthroline (2.5 mg, 0.12 mmol, 6 mol%) and 2-phenyl-1,3-dioxane (**3af**, 39.4 mg, 0.24 mmol, 1.2 equivalents) were added. Subsequently, the reaction vial was brought inside a nitrogen-filled glovebox and $NiBr_2$·dme (3.1, 0.01 mmol, 5 mol%),

NH_4Cl (1.1, 0.02 mmol, 10 mol%) and Na_2CO_3 (42.4 mg, 0.4 mmol, 2 equivalents) were added. Then, the vial was closed and taken outside the glovebox where benzene:*tert*-amyl alcohol (1:1, 2.0 mL, 0.1 M) and 1-bromo-4-(trifluoromethyl)benzene (**4a**, 28 μL, 0.2 mmol, 1 equivalent) were added sequentially via syringe. The reaction was stirred at 650 rpm under 456 nm light irradiation for 40 h using the UFO photorreactor. After the reaction was completed, the crude was filtered through a short celite pad eluting with ethyl acetate and concentrated under vacuum. The crude was analyzed by ^{1}H NMR using 1,3,5-trimethoxybenzene as internal standard. By crude ^{1}H-NMR analysis, only trace product was obtained.

Influence of time under the optimized reaction conditions

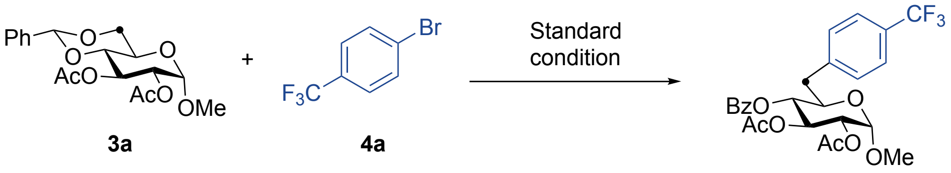

Time (h)	conversion of 3a (%)	P (%)
12	67	50
24	90	73
36	95	78
40	96	79

Nature of the aryl halide in the reaction conditions

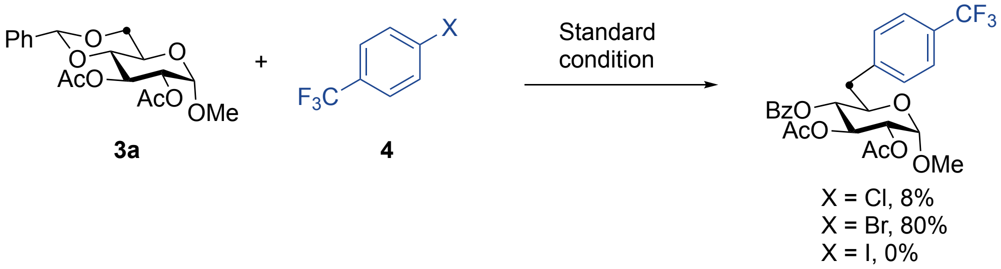

Mechanistic Experiments

Synthesis of an Oxidative Addition Ni Complex Ni-1. In a nitrogen-filled glovebox, a 20 mL vial was charged with $Ni(cod)_2$ (138 mg, 0.500 mmol, 1.00 equiv), 4,4'-di-*tert*-butyl-2,2'-bipyridine (134 mg, 0.500 mmol 1.00 equiv), and Et_2O (10 mL) and stirred at room temperature for 30 min. To the resulting dark purple a solution was added 2-bromobenzotrifluoride (272 mL, 1.00 mmol, 4.00 equiv), followed by stirring for 30 min. The red solution was then concentrated under vacuum (ca. 2 mL), precipitated with pentane (10 mL), and stored in the freezer for 2 h. A yellow-orange solid was filtered was washed with pentane (4´3 mL), and dried under vacuum. Yield of bright-yellow **Ni-1**: 215 mg, 0.389 mmol (78%). The complex was stored at –35 ºC in the glovebox freezer for further use. Crystals suitable for X-ray diffraction were obtained by liquid diffusion of pentane into a solution of **Ni-1** in Et_2O. **^{19}F{^{1}H} NMR** (376 MHz, THF-d_8) δ –58.41. **^{1}H NMR** (300 MHz, THF-d_8) δ 9.36 (brs, 1H, *t*Bubipy CH), 8.11 (s, 2H, *t*Bubipy CH), 8.03 (d, $^2J_{HH}$ = 7.0 Hz, 1H, Ar CH), 7.61 (br. s, 1H, *t*Bubipy CH), 7.27–7.21 (overlapping br. s + d, $^2J_{HH}$ = 7.0 Hz, 2H, *t*Bubipy CH + Ar CH), 6.98 (dd, $^2J_{HH}$ = 7.8 Hz, 1H, Ar CH), 6.92 (br. s, 1H, *t*Bubipy CH), 6.85 (dd, $^2J_{HH}$ = 7.8 Hz, 1H, Ar CH), 1.35 (s, 18H, *t*Bubipy CH_3).**^{13}C{^{1}H} NMR** (100 MHz, THF-d_8): δ 30.2, 36.0, 54.8, 118.5, 119.2, 122.4, 123.9, 124.8, 125.3, 125.3, 127.5, 127.6, 136.3, 136.6, 139.4, 151.0, 151.5, 152.0, 164.1.

UV–Vis Spectroscopic Analysis

To evaluate the photochemical properties of **Ni-1**, UV–vis absorption spectra were recorded across a range of concentrations in anhydrous THF. As shown in Figure S3a, **Ni-1** displays an absorption band in the visible region (467 nm). The linear dependence of absorbance of this band on concentration was confirmed by Beer–Lambert plots at selected wavelengths. At 467 nm, a calibration curve shows a high linear correlation with concentration (R^2 = 0.999, Figure S3b), yielding a molar extinction coefficient (ε) of 1699 M^{-1} cm^{-1}. This value confirms that **Ni-3** effectively absorbs light at the 456 nm excitation wavelength used for reactivity and quantum yield studies.

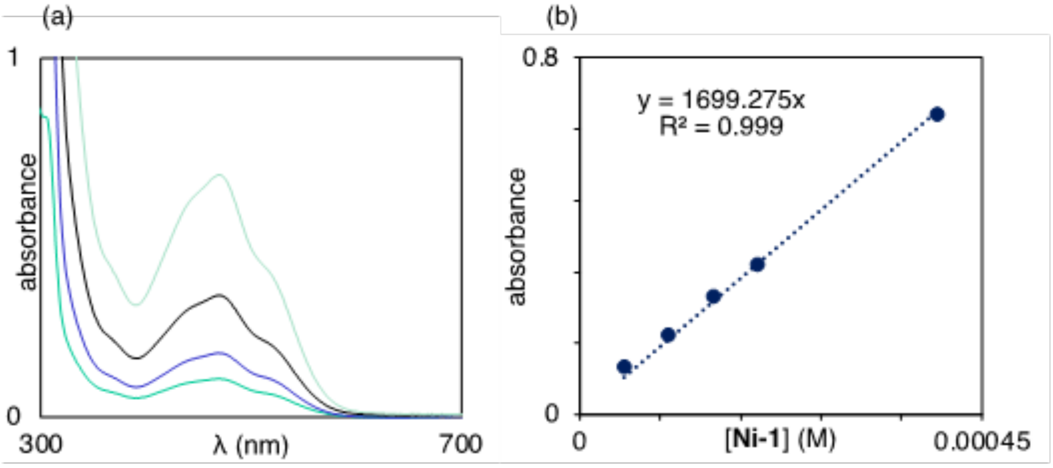

Figure S3. (a) UV–vis plots in THF of **Ni-1** at different concentrations, and (b) Beer–Lambert plot used to determine molar extinction coefficients at 467 nm.

- **Photostability and Kinetic Analysis of Ni-1 under 451 nm Irradiation**

The photodegradation of **Ni-1** was investigated under the same photochemical conditions used for catalytic reactions, employing a 456 nm Kessil lamp (PR160L-456 nm, 40 W) used on full intensity. The photon flux and output power of the LED setup was independently characterized and used to standardize rate and quantum yield calculations. In a N_2-filled glovebox, 2.0 mL of a 500 mM solution of **Ni-1** in anhydrous benzene was transferred to a screw-cap quartz cuvette and irradiated under constant stirring at 30 ºC. The absorbance at 451 nm was monitored at regular time intervals to track the degradation process (Figure S4a). The decay profile showed linearity between concentration and time, meaning good fitting to zeroth order kinetics (Figure S4b). The slope for this rate constant of k = 10.7 mM·min^{-1}, corresponding to a half-life $t_{1/2}$ = 23.4 min, with a fit quality of R^2 = 0.995. Decomposition proceeded cleanly over one full half-life at 30 ºC. Attempts to linearize the data using integrated forms of first-, and second-order kinetics did not produce satisfactory linear plots (Figures S4c and S4d).

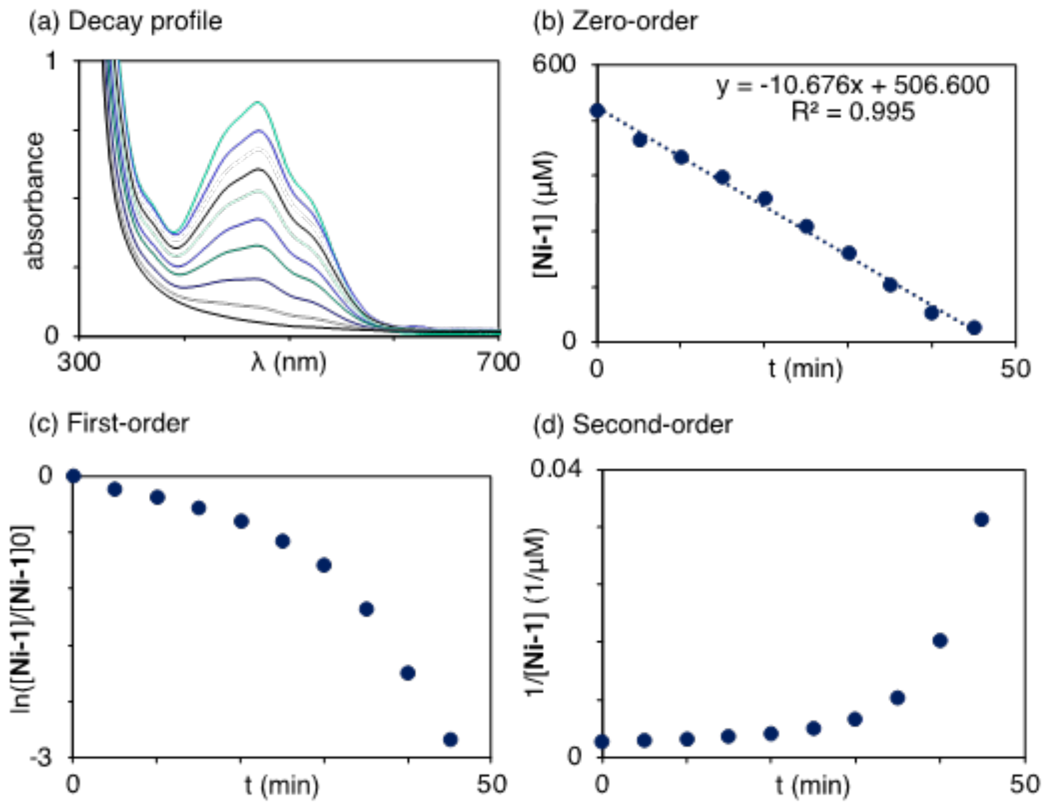

Figure S4. Kinetic analysis of photolysis of oxidative addition complex **Ni-1** under 456 nm irradiation.

- **Quantum yield**

Quantum yield (F) was calculated using the rate of photodegradation of **Ni-1** and the total photon flux delivered by the light source, following Equation S2. The calculation incorporates Planck's constant (h), the speed of light (c), Avogadro's constant (N_A), the volume of the irradiated solution ($V_{cuvette}$), the rate of degradation of the complex ($k_{complex}$), the initial concentration of **Ni-1**, the power of the LED (P_{LED}), the wavelength of the LED (λ_{LED}), and the absorbance of **Ni-1** at the irradiation wavelength ($A_{\lambda LED}$).

$\Phi = h * c * NA * Vcuvette * kdegradation * [Ni-1]0PLED * \lambda LED * (1-10-(A\lambda LED$
$(Ni-1)))\Phi = h * c * NA * Vcuvette * kdegradation * [Ni-1]0PLED * \lambda LED * (1-10-A\lambda LED\ Ni-1)$

In this study, quartz cuvettes with 1 cm path lengths were filled them with 2.0 mL solution, which results in having a side rectangular surface area of about 200 mm^2. The surface exposed to LED irradiation was that of the bottom of the cuvette (100 mm^2). As such, the measured P_{LED} values reported in mW/cm^2 are a close approximation of the power experienced by the cuvette and by extension to that of the reaction tubes in the catalytic reactions.

Supplementary Table S2. Values for calculating quantum yield using Equation S2 for compound **Ni-3**.

**Compound**	**λ_{LED} (10^{-9} m)**	**$k_{degradation}$ (μM•min^{-1})**	$A_{\lambda LED}^{Ni(II)}$	***[Ni-1] (μM)***	***P_{LED}* (W)**	**Φ (10-4)**
**Ni-1**	456	10.7	0.85	3.6E-4	1.6E-1	6.70

This low value suggests that only ~0.07% of absorbed photons lead to productive decomposition of **Ni-1** under these conditions.

EPR characterization of photogenerated Ni(I) species from Ni-1

To investigate the photogeneration of Ni(I) species from irradiation of Ni(II) complex **Ni-1**, time-resolved X-band CW-EPR spectroscopy was employed using a Bruker EMXmicro spectrometer (9.387 GHz, modulation amplitude: 10 G, microwave power: 0.5375 mW, time constant: 20.48 ms, 148 scans per spectrum). A capped reaction tube charged with **Ni-1** (5 mg, 0.0091 mmol) and benzene (1.5 mL, 6 mM **Ni-1**) was subjected to continuous irradiation with a 456 nm Kessil lamp. After 1 h, the resulting dark green solution was analysed by EPR at room temperature, revealing full conversion to an EPR-active species (Figure S5a), a monomeric Ni(I) complex with spin ½, showing a rhombic signal characterized by g_x=2.108, g_y=2.194, and g_z=2.232, that can be assigned to **Ni-1'**, consistent with previously reported studies by Doyle et al. These data support a mechanistic scenario in which, under 456 nm irradiation, the *ortho* trifluoromethyl phenyl ligand in the **Ni-1** complex undergoes homolytic cleavage to generate **Ni-1'** and a free *ortho* trifluoromethyl phenyl radical. The presence of aryl radical was confirmed by performing an identical experiment in the presence of PBN (4.8 mg, 0.027 mmol, 3 equiv), an observing the formation of the corresponding spin adduct shown in Figure S5b.

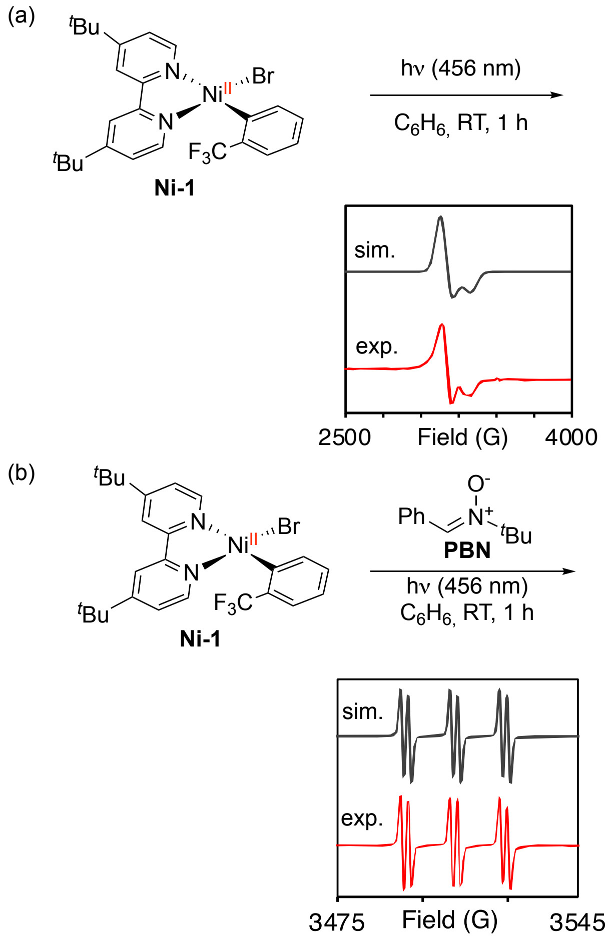

Figure S5. Irradiation of **Ni-1** generates (a) Ni(I) species and (b) an aryl radical.

Spin trapping experiments.

Trapping β-scission radical. Following General Procedure A, with addition of DMPO (23 mg, 0.200 mmol, 1 equiv) or PBN (45 mg, 0.200 mmol, 1 equiv) from the onset. Direct EPR analysis (RT) of this reaction crude mixture after 1 h revealed formation of carbon radicals (Figure S6). HR ESI MS analysis of this mixture with DMPO showed the mass of the corresponding adduct of DMPO with the carbohydrate radical posterior to β scission.

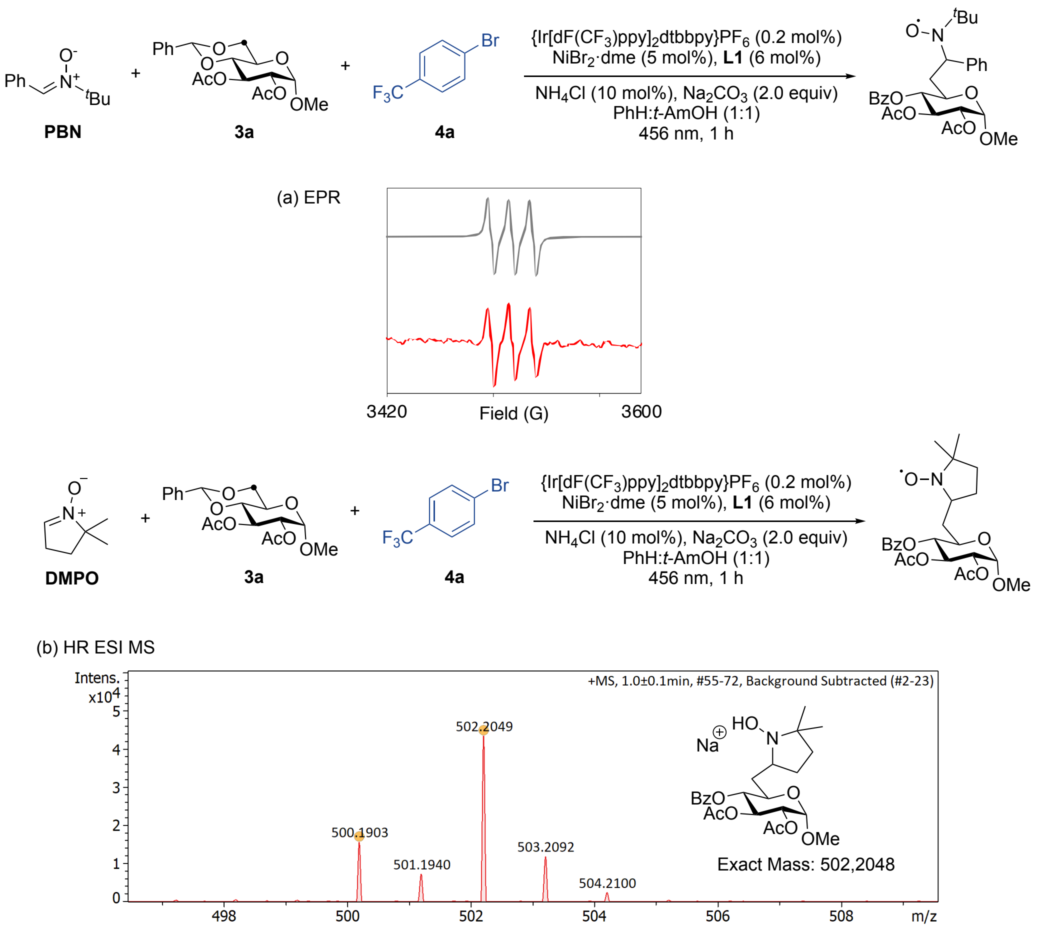

Figure S6. Observing β-scission radicals via spin trapping.

Trapping bromine radicals generated by the Ir photocatalyst. A reaction tube was charged with {Ir[dF(CF$_3$)ppy]$_2$(dtbbpy)}PF$_6$ (4.6 mg, 0.0041 mmol), NMe$_4$Br, (16 mg, 0.10 mmol, 24 equiv), DMPO (5.8 mg, 0.051 mmol, 12 equiv), and THF (1.5 mL). The resulting solution was stirred under irradiation with a 456 nm Kessil for 1 h, then transferred into a quartz capillary, and immediately analysed by EPR (RT). A A mixture of bromine radical and THF-derived radical adducts was observed, the latter arising from HAT by bromine radicals on the solvent (Figure S7).

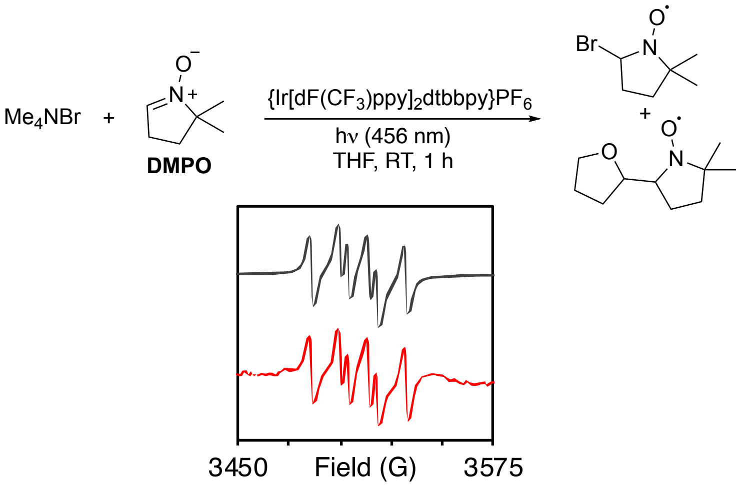

Figure S7. Spin trapping of bromine and THF-derived radicals generated by the photocatalyst.

Trapping bromine radicals generated by $NiBr_2$dtbbpy. A reaction tube was charged with solid green $NiBr_2$dtbbpy (5.0 mg, 0.010 mmol), DMPO (5.8 mg, 0.051 mmol, 12 equiv), and THF (1.5 mL). The resulting yellow suspension was stirred under irradiation with a 456 nm Kessil for 1 h, then transferred into a quartz capillary, and immediately analysed by EPR (RT). A mixture of bromine radical and THF-derived radical adducts was observed, the latter arising from HAT by bromine radicals on the solvent (Figure S8a). In the absence of light, no radicals were detected (Figure S8b).

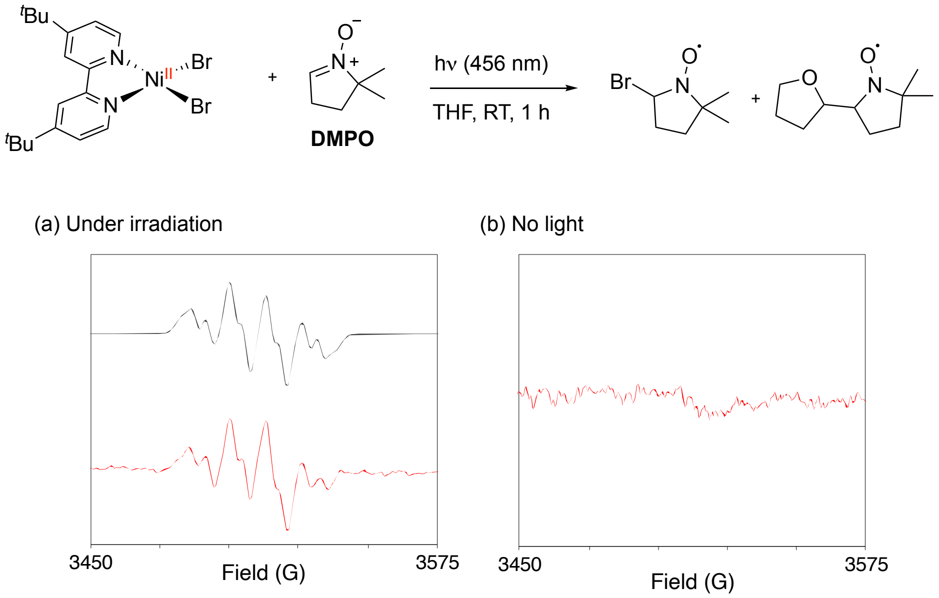

Figure S8. Spin trapping of bromine and THF-derived radicals generated by $NiBr_2$dtbbpy.

Stern-Volmer fluorescence-quenching experiments.

A 37×10^{-6} M solution of the photocatalyst (Ir[dF(CF_3)ppy]$_2$dtbbpyPF_6) in anhydrous and degassed acetone was prepared in a nitrogen-filled glovebox and transferred to a quartz cuvette (path length: l = 1.0 cm) with a septum cap. Upon irradiation at 456 nm, emission with a

maximum at 473 nm was observed. Aliquots of quencher solutions were injected through the septum under a nitrogen atmosphere (balloon), and emission spectra were recorded after each addition. Figure S10 shows the emission intensity ratio (I/I_0) as a function of quencher concentration, where I and I_0 denote the emission intensities in the presence and absence of quencher, respectively

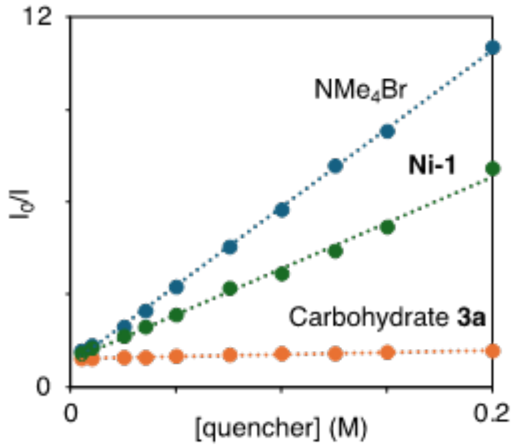

Figure S9. Fluorescence quenching of {Ir[dF(CF_3)ppy]$_2$(dtbbpy)}PF_6.

X-Ray Crystallographic Data

X-ray structure of 3r

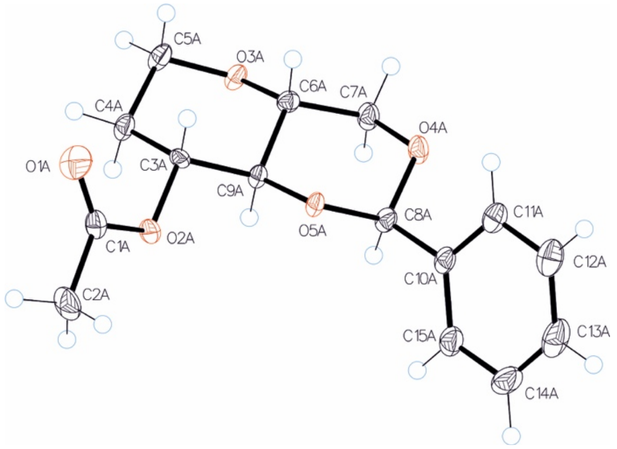

Figure S10. Crystal structure of compound **3r.**

Measurements were made in a Bruker APEX-II CCD diffractometer. The crystal was kept at 100.15 K during data collection.

Supplementary Table 3. Crystal data and structure refinement for 3r.

Identification code	mo_JO512_0m_a
Empirical formula	$C_{15}H_{18}O_5$
Formula weight	278.29
Temperature/K	100.15
Crystal system	monoclinic
Space group	$P2_1$
a/Å	8.5215(6)
b/Å	17.2007(14)
c/Å	10.0618(8)
α/°	90
β/°	109.429(3)
γ/°	90
Volume/Å^3	1390.83(19)
Z	4
ρ_{calc}g/cm^3	1.329
μ/mm^{-1}	0.100
F(000)	592.0
Crystal size/mm^3	0.1 × 0.1 × 0.1
Radiation	MoKα (λ = 0.71073)

2Θ range for data collection/°	4.292 to 62.914
Index ranges	$-12 \le h \le 12$, $-25 \le k \le 24$, $-14 \le l \le 10$
Reflections collected	16952
Independent reflections	8616 [R_{int} = 0.0534, R_{sigma} = 0.0929]
Data/restraints/parameters	8616/1/363
Goodness-of-fit on F^2	1.013
Final R indexes [I>=2σ (I)]	R_1 = 0.0552, wR_2 = 0.1043
Final R indexes [all data]	R_1 = 0.0935, wR_2 = 0.1200
Largest diff. peak/hole / e Å^{-3}	0.26/-0.28
Flack parameter	-0.3(7)

X-Ray structure of 5e

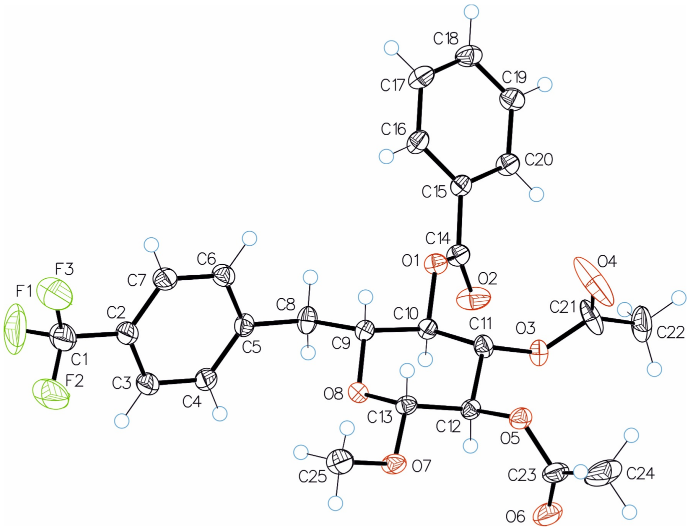

Figure S11. Crystal structure of compound **5e** (CCDC-2478708)

Measurements were made in a **XtaLAB AFC11 (RCD3): quarter-chi single** diffractometer. The crystal was kept at 100(2) K during data collection.

Supplementary Table 4. Crystal data and structure refinement for 5e.

Identification code	SZ-2-33-3
Empirical formula	$C_{25}H_{24}F_3O_8$
Formula weight	509.44
Temperature/K	100(2)
Crystal system	orthorhombic
Space group	$P2_12_12_1$
a/Å	7.02850(10)
b/Å	13.5872(2)

c/Å	25.8551(5)
α/°	90
β/°	90
γ/°	90
Volume/$Å^3$	2469.10(7)
Z	4
ρ_{calc}g/cm^3	1.370
μ/mm^{-1}	0.116
F(000)	1060.0
Crystal size/mm^3	0.5 × 0.5 × 0.5
Radiation	Mo Kα (λ = 0.71073)
2Θ range for data collection/°	5.598 to 72.636
Index ranges	-11 ≤ h ≤ 11, -22 ≤ k ≤ 22, -42 ≤ l ≤ 43
Reflections collected	44978
Independent reflections	11954 [R_{int} = 0.0290, R_{sigma} = 0.0233]
Data/restraints/parameters	11954/135/390
Goodness-of-fit on F^2	1.045
Final R indexes [I>=2σ (I)]	R_1 = 0.0426, wR_2 = 0.1206
Final R indexes [all data]	R_1 = 0.0437, wR_2 = 0.1217
Largest diff. peak/hole / e $Å^{-3}$	0.82/-0.42
Flack parameter	0.01(8)

X-Ray structure of 5n

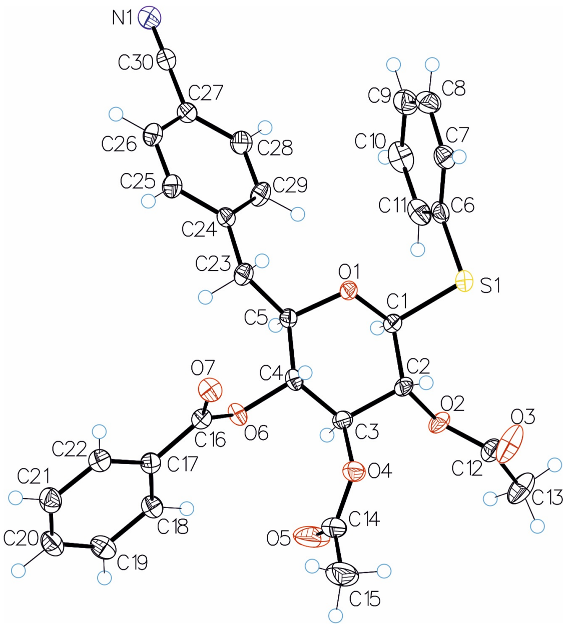

Figure S12. Crystal structure of compound **5n** (CCDC-2478709)

Measurements were made in a Bruker APEX-II CCD diffractometer. The crystal was kept at 99.93 K during data collection.

Supplementary Table 4. Crystal data and structure refinement for 5n.

Identification code	mo_s211811_0m_a
Empirical formula	$C_{30}H_{27}NO_7S$
Formula weight	545.58
Temperature/K	99.93
Crystal system	monoclinic
Space group	$P2_1$
a/Å	5.4969(11)
b/Å	12.948(3)
c/Å	19.686(4)
α/°	90
β/°	95.397(7)
γ/°	90
Volume/Å^3	1395.0(5)
Z	2
ρ_{calc}g/cm^3	1.299
μ/mm^{-1}	0.164
F(000)	572.0
Crystal size/mm^3	0.2 × 0.2 × 0.05
Radiation	MoKα (λ = 0.71073)
2Θ range for data collection/°	3.77 to 55.106
Index ranges	$-6 \leq h \leq 7, -16 \leq k \leq 12, -25 \leq l \leq 25$
Reflections collected	19105
Independent reflections	5495 [R_{int} = 0.0467, R_{sigma} = 0.0458]
Data/restraints/parameters	5495/1/354
Goodness-of-fit on F^2	1.039
Final R indexes [I>=2σ (I)]	R_1 = 0.0384, wR_2 = 0.0924
Final R indexes [all data]	R_1 = 0.0447, wR_2 = 0.0957
Largest diff. peak/hole / e Å^{-3}	0.24/-0.23
Flack parameter	0.01(7)

X-Ray structure of 5r

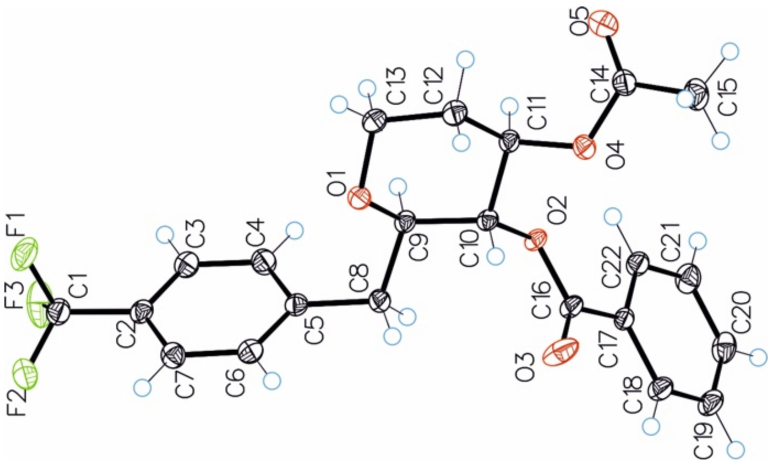

Figure S13. Crystal structure of compound **5r** (CCDC-2478710)

Measurements were made on a Bruker APEX-II CCD diffractometer. The crystal was kept at 99.73 K during data collection.

Supplementary Table 6. Crystal data and structure refinement for 5r.

Identification code	mo_JO5182C_0m_a
Empirical formula	$C_{22}H_{21}F_3O_5$
Formula weight	422.39
Temperature/K	99.73
Crystal system	monoclinic
Space group	$P2_1$
a/Å	8.9236(14)
b/Å	10.0145(14)
c/Å	11.454(2)
α/°	90
β/°	95.031(6)
γ/°	90
Volume/Å^3	1019.6(3)
Z	2
ρ_{calc}g/cm^3	1.376
μ/mm^{-1}	0.114
F(000)	440.0
Crystal size/mm^3	0.4 × 0.4 × 0.4
Radiation	MoKα (λ = 0.71073)
2Θ range for data collection/°	3.57 to 61.212
Index ranges	$-12 \le h \le 12$, $-14 \le k \le 9$, $-16 \le l \le 14$

Reflections collected	14648
Independent reflections	5028 [R_{int} = 0.0483, R_{sigma} = 0.0512]
Data/restraints/parameters	5028/1/272
Goodness-of-fit on F^2	1.038
Final R indexes [I>=2σ (I)]	R_1 = 0.0423, wR_2 = 0.1094
Final R indexes [all data]	R_1 = 0.0439, wR_2 = 0.1112
Largest diff. peak/hole / e Å^{-3}	0.40/-0.33

X-Ray structure of 5za

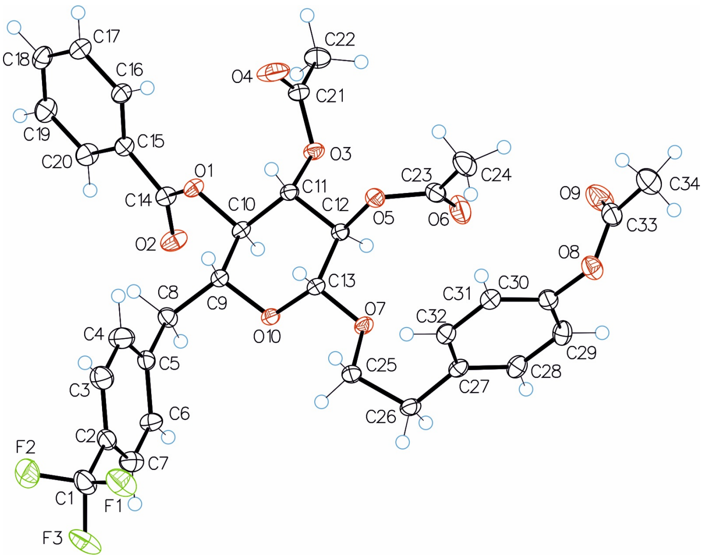

Figure S14. Crystal structure of compound **5za** (CCDC-2478711)

Measurements were made in a **XtaLAB AFC11 (RCD3): quarter-chi single** diffractometer. The crystal was kept at 103(5) K during data collection.

Supplementary Table 7. Crystal data and structure refinement for 5za.

Identification code	SZ-2-34-2_c
Empirical formula	$C_{34}H_{33}F_3O_{10}$
Formula weight	658.60
Temperature/K	103(5)
Crystal system	orthorhombic
Space group	$P2_12_12_1$
a/Å	5.83116(7)
b/Å	11.37248(13)
c/Å	48.3843(7)
α/°	90

β/°	90
γ/°	90
Volume/Å^3	3208.59(7)
Z	4
ρ_{calc}g/cm^3	1.363
μ/mm^{-1}	0.111
F(000)	1376.0
Crystal size/mm^3	0.4 × 0.2 × 0.2
Radiation	Mo Kα (λ = 0.71073)
2Θ range for data collection/°	6.194 to 69.992
Index ranges	$-9 \leq h \leq 6$, $-18 \leq k \leq 17$, $-56 \leq l \leq 77$
Reflections collected	45707
Independent reflections	13492 [R_{int} = 0.0248, R_{sigma} = 0.0268]
Data/restraints/parameters	13492/207/499
Goodness-of-fit on F^2	1.045
Final R indexes [I>=2σ (I)]	R_1 = 0.0375, wR_2 = 0.0969
Final R indexes [all data]	R_1 = 0.0417, wR_2 = 0.0990
Largest diff. peak/hole / e Å^{-3}	0.40/-0.57
Flack parameter	0.07(12)

X-Ray structure of 9a

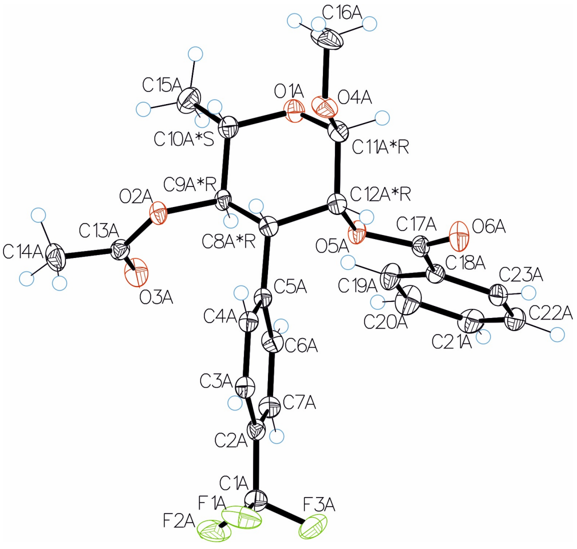

Figure S15. Crystal structure of compound **9a** (CCDC-2478712)

Measurements were made in a Bruker APEX-II CCD diffractometer. The crystal was kept at 99.80 K during data collection.

Supplementary Table 8. Crystal data and structure refinement for 9a.

Identification code	mo_SZ2413_0m_a
Empirical formula	$C_{23}H_{23}F_3O_6$
Formula weight	452.41
Temperature/K	99.80
Crystal system	monoclinic
Space group	$P2_1$
a/Å	12.233(3)
b/Å	10.783(3)
c/Å	17.482(4)
α/°	90
β/°	106.175(6)
γ/°	90
Volume/Å^3	2214.8(8)
Z	4
ρ_{calc}g/cm^3	1.357
μ/mm^{-1}	0.113
F(000)	944.0
Crystal size/mm^3	0.3 × 0.2 × 0.2
Radiation	MoKα (λ = 0.71073)
2Θ range for data collection/°	3.636 to 63.168
Index ranges	$-17 \leq h \leq 9$, $-15 \leq k \leq 15$, $-25 \leq l \leq 25$
Reflections collected	31518
Independent reflections	14357 [R_{int} = 0.0560, R_{sigma} = 0.0645]
Data/restraints/parameters	14357/1/583
Goodness-of-fit on F^2	1.025
Final R indexes [I>=2σ (I)]	R_1 = 0.0467, wR_2 = 0.1199
Final R indexes [all data]	R_1 = 0.0553, wR_2 = 0.1259
Largest diff. peak/hole / e Å^{-3}	0.36/-0.31
Flack parameter	-0.3(3)

X-Ray structure of Ni-1

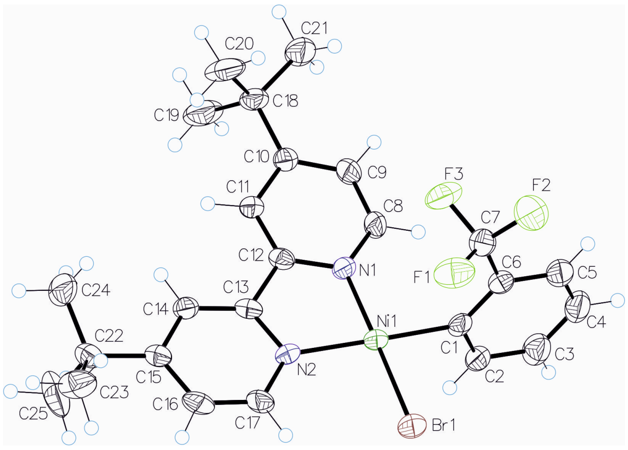

Figure S16. Crystal structure of compound **Ni-1** (CCDC-2547123)

Supplementary Table 9. Crystal data and structure refinement for Ni-1.

Identification code	COBIV027bLT
Empirical formula	$C_{25}H_{28}BrF_3N_2Ni$
Formula weight	552.11
Temperature (K)	100(2)
Crystal system	monoclinic
Space group	$P2_1/c$
a (Å)	16.5463(17)
b (Å)	10.4481(8)
c (Å)	13.9059(15)
α (°)	90
β (°)	95.136(9)
γ (°)	90
Volume ($Å^3$)	2394.4(4)
Z	4
ρcalc (g/cm^3)	1.532
μ (mm^{-1})	2.517
F(000)	1128.0
Crystal size (mm^3)	0.2 × 0.15 × 0.1
Radiation	Mo Kα (λ = 0.71073 Å)
2θ range for data collection (°)	4.884 to 51.362
Index ranges	$-20 \leq h \leq 18$, $-12 \leq k \leq 11$, $-15 \leq l \leq 16$
Reflections collected	15438
Independent reflections	4498 [R_{int} = 0.1110, R_{sigma} = 0.1175]
Data/restraints/parameters	4498/264/361
Goodness-of-fit on F^2	1.025
Final R indexes [I ≥ 2σ(I)]	R_1 = 0.0612, wR_2 = 0.1435

Final R indexes [all data]	R_1 = 0.1069, wR_2 = 0.1642
Largest diff. peak/hole (e Å^{-3})	0.96 / −0.79

Computational Studies

All structures were optimized using density functional theory (DFT) as implemented in Gaussian 16,[13] with M06-2X[14] as functional and 6-311++G(d,p) as basis set, introducing solvation factors with the IEF-PCM[15] method (2-propanol as solvent). The stationary points were characterized by frequency calculations in order to verify that they have the right number of imaginary frequencies.

Supplementary Table 10. Energies of the structures studied in the main manuscript and figure above. Energies are given in Hartress. ΔG in kcal/mol.

Compound	G (M06-2X)	ΔG	Negative Frequency
**1**	-694.420649		
**III-rad**	-693.790342	0	
**III-TS**	-693.756562	21.2	-1166.7
**III-cat**	-693.653971		
**2**	-730.346553		
**IV -rad**	-729.714873	0	
**IV-TS-prim**	-729.682805	20.1	-1146.8
**IV-TS-sec**	-729.677066	23.7	-862.9
**IV-cat**	-729.572799		
**3ab-rad**	-1072.046262	0	
**TS3ab-$_{C3}$**	-1072.009389	23.1	-1134.4
**TS3ab-$_{C4}$**	-1072.014799	19.7	-1009.5
**3ab-int$_{C3}$**	-1072.057190	-6.9	
**3ab-int$_{C4}$**	-1072.057323	-6.9	

Supplementary Table 11. Cartesian coordinates of the optimized structures are shown below:

1

Standard orientation:

Center Number	Atomic Number	Atomic Type	Coordinates (Angstroms) X	Y	Z
1	6	0	-2.365383	1.676797	-0.211039
2	6	0	-3.845301	1.450986	0.119724
3	6	0	-4.348348	0.111031	-0.428304
4	6	0	-2.019294	-0.813957	-0.292542
5	6	0	-1.546150	0.509859	0.303473
6	1	0	-5.390794	-0.044052	-0.139800
7	1	0	-3.974975	1.465270	1.208210
8	1	0	-2.227841	1.749894	-1.295607
9	1	0	-1.921600	-0.734967	-1.382901
10	1	0	-1.641695	0.454879	1.401185
11	8	0	-0.167339	0.713371	-0.010931
12	6	0	0.623480	-0.342008	0.474443
13	8	0	0.268714	-1.567448	-0.111714
14	6	0	-1.083473	-1.914347	0.184235
15	1	0	-1.192214	-2.063097	1.268448
16	1	0	-1.278735	-2.861613	-0.318887
17	6	0	2.076452	-0.049620	0.186047
18	6	0	2.536548	1.266812	0.194720
19	6	0	2.974379	-1.091250	-0.041286
20	6	0	3.883177	1.538538	-0.027120
21	1	0	1.836466	2.074841	0.367053
22	6	0	4.321215	-0.817131	-0.263360
23	1	0	2.614421	-2.112252	-0.050750
24	6	0	4.778812	0.497367	-0.256804
25	1	0	4.232526	2.564404	-0.022832
26	1	0	5.012607	-1.631944	-0.443738
27	1	0	5.827083	0.709746	-0.430937
28	1	0	-4.443960	2.271989	-0.281114
29	1	0	-4.319403	0.136383	-1.523704
30	1	0	-2.002630	2.606823	0.233750
31	6	0	-3.484522	-1.054078	0.065246
32	1	0	-3.825616	-1.998178	-0.369105
33	1	0	-3.581483	-1.147781	1.154278
34	1	0	0.457297	-0.421718	1.563012

III-rad

Standard orientation:

Center Number	Atomic Number	Atomic Type	Coordinates (Angstroms) X	Y	Z
1	6	0	2.404444	-1.654185	-0.261718
2	6	0	3.810567	-1.452850	0.315307
3	6	0	4.385923	-0.085547	-0.069297
4	6	0	2.059620	0.838535	-0.249750
5	6	0	1.513113	-0.513865	0.182553
6	1	0	5.368084	0.050301	0.389227
7	1	0	3.765013	-1.530183	1.407678
8	1	0	2.442441	-1.662551	-1.356485
9	1	0	2.132829	0.835679	-1.344779
10	1	0	1.407480	-0.525642	1.277579
11	8	0	0.196975	-0.703191	-0.374936
12	6	0	-0.667700	0.312129	-0.162617
13	8	0	-0.245252	1.579133	-0.362998
14	6	0	1.055872	1.896757	0.162347
15	1	0	0.983154	1.956744	1.254982
16	1	0	1.302034	2.879993	-0.235516
17	6	0	-2.049145	0.046048	-0.057603
18	6	0	-2.531123	-1.288078	-0.010280
19	6	0	-2.992075	1.104957	0.004961
20	6	0	-3.886836	-1.537353	0.107637
21	1	0	-1.824626	-2.106505	-0.063572
22	6	0	-4.343131	0.831284	0.122817
23	1	0	-2.640420	2.127892	-0.036766
24	6	0	-4.807189	-0.486755	0.177249
25	1	0	-4.236656	-2.562942	0.145194
26	1	0	-5.048813	1.652990	0.171948
27	1	0	-5.866549	-0.691492	0.269985
28	1	0	4.468708	-2.252020	-0.031938
29	1	0	4.530216	-0.047136	-1.154957
30	1	0	1.978468	-2.605530	0.065473
31	6	0	3.448930	1.051448	0.350053
32	1	0	3.846784	2.018684	0.031554
33	1	0	3.370452	1.079044	1.443907

III-TS

Standard orientation:

Center Number	Atomic Number	Atomic Type	Coordinates (Angstroms) X	Y	Z
1	6	0	-2.389588	-1.595040	-0.305031
2	6	0	-3.888902	-1.294820	-0.297799
3	6	0	-4.222991	-0.245860	0.763381
4	6	0	-1.893290	0.749282	0.519438
5	6	0	-1.583726	-0.328339	-0.531645
6	1	0	-5.290151	-0.011514	0.745372
7	1	0	-4.192365	-0.923387	-1.283433
8	1	0	-2.092260	-2.023250	0.658792
9	1	0	-1.600581	0.327050	1.488598
10	1	0	-1.773420	0.072853	-1.534683
11	8	0	-0.194733	-0.715908	-0.449613
12	6	0	0.685749	0.300666	-0.591777
13	8	0	0.259624	1.434144	-0.914897
14	6	0	-1.118922	2.018360	0.267715
15	1	0	-1.566875	2.736751	-0.409626
16	1	0	-0.542123	2.444881	1.079339
17	6	0	2.055526	0.015877	-0.203360
18	6	0	2.467634	-1.287257	0.123891
19	6	0	2.995651	1.060626	-0.178882
20	6	0	3.791669	-1.532650	0.462443
21	1	0	1.745948	-2.094067	0.110071
22	6	0	4.312190	0.805415	0.172144
23	1	0	2.675726	2.062570	-0.438113
24	6	0	4.718959	-0.491757	0.491007
25	1	0	4.102397	-2.540728	0.711175
26	1	0	5.028596	1.618285	0.195016
27	1	0	5.750077	-0.688461	0.758769
28	1	0	-2.135726	-2.322209	-1.080071
29	1	0	-4.447755	-2.215865	-0.118444
30	1	0	-3.999223	-0.650715	1.757049
31	6	0	-3.408047	1.027910	0.539094
32	1	0	-3.689828	1.478405	-0.420686
33	1	0	-3.623027	1.769036	1.313235

III-cat

Standard orientation:

Center Number	Atomic Number	Atomic Type	Coordinates (Angstroms) X	Y	Z
1	6	0	2.322972	-1.675881	-0.056678
2	6	0	3.832711	-1.446858	0.101567
3	6	0	4.283677	-0.156327	-0.589957
4	6	0	1.993895	0.811261	-0.272363
5	6	0	1.604302	-0.453307	0.463121
6	1	0	5.349946	0.004539	-0.420938
7	1	0	4.079008	-1.394644	1.167624
8	1	0	2.067330	-1.814575	-1.111799
9	1	0	1.782051	0.668419	-1.339001
10	1	0	1.750656	-0.338419	1.541205
11	8	0	0.152649	-0.667867	0.298543
12	6	0	-0.648793	0.322719	0.188496
13	8	0	-0.277915	1.547432	0.192484
14	6	0	1.137451	1.936135	0.252345
15	1	0	1.339583	2.168357	1.298323
16	1	0	1.189408	2.839095	-0.349770
17	6	0	-2.071826	0.043264	0.049708
18	6	0	-2.517224	-1.283504	0.066031
19	6	0	-2.972047	1.105077	-0.096210
20	6	0	-3.871888	-1.543350	-0.067313
21	1	0	-1.807950	-2.092753	0.181747
22	6	0	-4.324076	0.831600	-0.228343
23	1	0	-2.610753	2.125177	-0.106211
24	6	0	-4.771259	-0.488576	-0.214012
25	1	0	-4.228451	-2.565196	-0.056520
26	1	0	-5.029726	1.644283	-0.342647
27	1	0	-5.829375	-0.696929	-0.317580
28	1	0	1.998762	-2.560870	0.493343
29	1	0	4.369621	-2.304162	-0.307068
30	1	0	4.140428	-0.253520	-1.671389
31	6	0	3.494172	1.056011	-0.086441
32	1	0	3.785139	1.961297	-0.623264
33	1	0	3.704050	1.223808	0.976030

2

Standard orientation:

Center Number	Atomic Number	Atomic Type	Coordinates (Angstroms) X	Y	Z
1	6	0	-2.404874	1.688255	-0.193498
2	6	0	-3.872352	1.353105	0.102589
3	6	0	-4.232883	-0.031263	-0.428019
4	6	0	-2.019956	-0.773481	-0.284279
5	6	0	-1.553256	0.548393	0.323748
6	1	0	-5.237302	-0.322849	-0.122698
7	1	0	-4.044346	1.374920	1.182995
8	1	0	-2.246784	1.785180	-1.273040
9	1	0	-1.931128	-0.701719	-1.378982
10	1	0	-1.654733	0.475424	1.418036
11	8	0	-3.355958	-1.036844	0.079799
12	8	0	-0.181678	0.742180	-0.002334
13	6	0	0.602453	-0.318579	0.490149
14	8	0	0.230365	-1.544908	-0.088034
15	6	0	-1.117685	-1.889013	0.213721
16	1	0	-1.245648	-2.015908	1.296810
17	1	0	-1.328323	-2.833802	-0.285307
18	6	0	2.055602	-0.044377	0.189801
19	6	0	2.529252	1.267239	0.187924
20	6	0	2.940221	-1.097557	-0.035925
21	6	0	3.877255	1.522719	-0.043901
22	1	0	1.839452	2.084076	0.360177
23	6	0	4.288391	-0.839204	-0.267983
24	1	0	2.569747	-2.114789	-0.035986
25	6	0	4.759945	0.470315	-0.272297
26	1	0	4.237808	2.544642	-0.047740
27	1	0	4.969855	-1.662542	-0.447051
28	1	0	5.809385	0.670246	-0.453818
29	1	0	-2.111670	2.629312	0.276419
30	1	0	-4.534653	2.093662	-0.350944
31	1	0	-4.184331	-0.040330	-1.525281
32	1	0	0.438155	-0.388250	1.578775

IV-rad

Standard orientation:

Center Number	Atomic Number	Atomic Type	Coordinates (Angstroms) X	Y	Z
1	6	0	-2.441065	-1.669137	0.228211
2	6	0	-3.836893	-1.356499	-0.327058
3	6	0	-4.273399	0.052305	0.061509
4	6	0	-2.063944	0.795866	0.242830
5	6	0	-1.514379	-0.554062	-0.201780
6	1	0	-5.210656	0.324667	-0.421962
7	1	0	-3.826619	-1.437365	-1.418071
8	1	0	-2.464803	-1.705198	1.322526
9	1	0	-2.151553	0.795654	1.340062
10	1	0	-1.405471	-0.545366	-1.294812
11	8	0	-3.317536	1.031326	-0.350587
12	8	0	-0.212924	-0.730156	0.377579
13	6	0	0.643331	0.292862	0.146474
14	8	0	0.205563	1.557714	0.347637
15	6	0	-1.088448	1.872389	-0.185565
16	1	0	-1.032369	1.916963	-1.278360
17	1	0	-1.353866	2.850241	0.210378
18	6	0	2.029324	0.041648	0.053911
19	6	0	2.524936	-1.286584	0.021346
20	6	0	2.958385	1.110766	-0.013798
21	6	0	3.884190	-1.521677	-0.087670
22	1	0	1.827347	-2.112345	0.078657
23	6	0	4.313368	0.851911	-0.122469
24	1	0	2.594431	2.129752	0.016537
25	6	0	4.791791	-0.461009	-0.162346
26	1	0	4.246109	-2.543289	-0.113835
27	1	0	5.009835	1.681043	-0.175624
28	1	0	5.853890	-0.654338	-0.247979
29	1	0	-2.076944	-2.632050	-0.134940
30	1	0	-4.567289	-2.075371	0.049642
31	1	0	-4.406044	0.123258	1.149173

IV-TS-prim

Standard orientation:

Center Number	Atomic Number	Atomic Type	Coordinates (Angstroms) X	Y	Z
1	6	0	-2.431940	-1.605248	-0.297387
2	6	0	-3.910400	-1.212331	-0.264377
3	6	0	-4.127883	-0.093300	0.744303
4	6	0	-1.916874	0.714689	0.513456
5	6	0	-1.591760	-0.365394	-0.531823
6	1	0	-5.153523	0.273912	0.721025
7	1	0	-4.229400	-0.868279	-1.252736
8	1	0	-2.139637	-2.045813	0.662031
9	1	0	-1.683211	0.290171	1.501580
10	1	0	-1.764201	0.041672	-1.533956
11	8	0	-0.210232	-0.742406	-0.404610
12	6	0	0.662429	0.278289	-0.575780
13	8	0	0.213100	1.407588	-0.892425
14	6	0	-1.135380	1.980186	0.288940
15	1	0	-1.606754	2.704690	-0.363417
16	1	0	-0.561473	2.383925	1.113074
17	6	0	2.038901	0.011637	-0.202043
18	6	0	2.468592	-1.285605	0.123892
19	6	0	2.965402	1.068018	-0.191518
20	6	0	3.798828	-1.514124	0.449253
21	1	0	1.756664	-2.101139	0.119126
22	6	0	4.288770	0.829198	0.145364
23	1	0	2.630637	2.065354	-0.449594
24	6	0	4.713495	-0.461980	0.463748
25	1	0	4.124343	-2.517489	0.697684
26	1	0	4.995918	1.650227	0.157582
27	1	0	5.749761	-0.645748	0.720557
28	1	0	-2.231553	-2.341852	-1.077995
29	1	0	-4.527946	-2.072537	0.002468
30	1	0	-3.905988	-0.447584	1.760206
31	8	0	-3.301208	1.030604	0.450310

IV-TS-sec

Standard orientation:

Center Number	Atomic Number	Atomic Type	Coordinates (Angstroms) X	Y	Z
1	6	0	-2.634788	1.678103	-0.100615
2	6	0	-3.977204	1.229337	0.514476
3	6	0	-2.038629	-0.740840	-0.208538
4	6	0	-1.633637	0.627953	0.226450
5	1	0	-3.906441	1.249692	1.609716
6	1	0	-2.753815	1.753570	-1.186737
7	1	0	-2.209604	-0.719236	-1.292140
8	1	0	-1.106098	0.711659	1.174200
9	8	0	-0.069015	0.693355	-0.955674
10	6	0	0.711368	-0.228640	-0.640459
11	8	0	0.269635	-1.519280	-0.618721
12	6	0	-0.956142	-1.764826	0.096208
13	1	0	-0.731172	-1.768594	1.169481
14	1	0	-1.262312	-2.766448	-0.203301
15	6	0	2.093394	-0.019320	-0.230750
16	6	0	2.610803	1.287038	-0.198868
17	6	0	2.926810	-1.096249	0.112411
18	6	0	3.926407	1.507115	0.178794
19	1	0	1.967180	2.114231	-0.472152
20	6	0	4.246177	-0.865757	0.479312
21	1	0	2.530061	-2.102964	0.088790
22	6	0	4.751380	0.432948	0.517012
23	1	0	4.315491	2.518139	0.207037
24	1	0	4.883393	-1.702200	0.741458
25	1	0	5.780589	0.608119	0.806422
26	1	0	-4.786672	1.890042	0.203225
27	1	0	-2.359668	2.657389	0.294044
28	6	0	-3.390136	-1.065032	0.445371
29	1	0	-3.782433	-2.018402	0.086983
30	1	0	-3.288074	-1.111246	1.538553
31	8	0	-4.338776	-0.074669	0.085595

IV-cat

Standard orientation:

Center Number	Atomic Number	Atomic Type	Coordinates (Angstroms) X	Y	Z
1	6	0	-2.378555	1.686717	-0.034442
2	6	0	-3.868548	1.337998	0.105866
3	6	0	-4.184954	0.006916	-0.565870
4	6	0	-1.999732	-0.768369	-0.284293
5	6	0	-1.602650	0.493098	0.465491
6	1	0	-5.207725	-0.306373	-0.366523
7	1	0	-4.136437	1.278575	1.164278
8	1	0	-2.123908	1.863136	-1.083768
9	1	0	-1.802675	-0.623525	-1.357421
10	1	0	-1.730658	0.349605	1.541549
11	8	0	-3.351014	-1.043894	-0.056580
12	8	0	-0.168223	0.709235	0.252356
13	6	0	0.627184	-0.293819	0.185784
14	8	0	0.239812	-1.514473	0.226645
15	6	0	-1.168259	-1.912470	0.245115
16	1	0	-1.417673	-2.146757	1.279242
17	1	0	-1.211986	-2.803525	-0.373938
18	6	0	2.050991	-0.035465	0.052318
19	6	0	2.512338	1.286792	0.046905
20	6	0	2.938610	-1.111621	-0.070802
21	6	0	3.870253	1.526941	-0.085220
22	1	0	1.813016	2.106873	0.145297
23	6	0	4.293863	-0.856866	-0.201849
24	1	0	2.564693	-2.127159	-0.065418
25	6	0	4.756675	0.458202	-0.209375
26	1	0	4.239791	2.544138	-0.091385
27	1	0	4.990144	-1.679708	-0.298977
28	1	0	5.817608	0.651748	-0.312645
29	1	0	-2.124956	2.578992	0.538644
30	1	0	-4.477131	2.123039	-0.345302
31	1	0	-4.039997	0.075674	-1.650770

3ab-rad

Standard orientation:

Center Number	Atomic Number	Atomic Type	Coordinates (Angstroms) X	Y	Z
1	6	0	-1.733377	-2.131306	0.093868
2	6	0	-0.588114	-1.625856	-0.772471
3	6	0	-0.663726	-0.146916	-1.150155
4	6	0	-2.498156	0.071907	0.554885
5	1	0	-1.182118	0.032785	-2.089943
6	1	0	-3.241285	-0.064663	-0.239500
7	1	0	-1.425384	-3.024278	0.648116
8	1	0	-0.496997	-2.255142	-1.659961
9	6	0	-1.240411	0.711101	-0.032621
10	1	0	-0.501877	0.828924	0.764847
11	8	0	-1.543438	1.985028	-0.602750
12	6	0	-1.208173	3.097652	0.087579
13	8	0	-0.676171	3.069633	1.165529
14	8	0	0.641940	-1.669273	-0.038912
15	6	0	1.412230	-0.652431	-0.496109
16	8	0	0.725922	0.166464	-1.330416
17	8	0	-2.112426	-1.202680	1.075087
18	6	0	-3.089471	0.869409	1.696977
19	1	0	-2.348395	1.006777	2.486475
20	1	0	-3.419857	1.848967	1.347687
21	1	0	-3.952315	0.339913	2.102870
22	8	0	-2.769102	-2.420084	-0.794483
23	6	0	-3.913150	-2.980788	-0.162099
24	1	0	-4.598818	-3.270354	-0.954960
25	1	0	-3.631494	-3.862488	0.421954
26	1	0	-4.395128	-2.252134	0.493997
27	6	0	2.749218	-0.476116	-0.153023
28	6	0	3.418921	-1.421980	0.674508
29	6	0	3.477950	0.653251	-0.623552
30	6	0	4.746525	-1.233425	1.005713
31	1	0	2.877180	-2.286157	1.038509
32	6	0	4.805056	0.813746	-0.276172
33	1	0	2.980513	1.381220	-1.252449
34	6	0	5.456464	-0.120383	0.538845
35	1	0	5.242922	-1.961612	1.637246
36	1	0	5.346847	1.678903	-0.641477
37	1	0	6.496944	0.016474	0.804999

38	6	0	-1.596934	4.329902	-0.671398
39	1	0	-1.298212	5.211995	-0.112115
40	1	0	-1.118489	4.321082	-1.651232
41	1	0	-2.676958	4.330483	-0.826167

3ab-TS$_{C3}$

Standard orientation:

Center Number	Atomic Number	Atomic Type	Coordinates (Angstroms) X	Y	Z
1	6	0	-1.403744	-2.257343	0.037854
2	6	0	-0.321023	-1.479574	-0.653372
3	6	0	-0.464459	-0.010722	-0.894960
4	6	0	-2.705711	-0.277712	0.194989
5	1	0	-0.687157	0.212414	-1.939349
6	1	0	-3.118073	-0.548735	-0.784795
7	1	0	-0.970268	-2.996975	0.721920
8	1	0	0.243942	-2.040869	-1.389102
9	6	0	-1.498974	0.641168	0.010567
10	1	0	-1.062655	0.846708	0.989599
11	8	0	-1.885336	1.867509	-0.615564
12	6	0	-2.022574	2.969935	0.151118
13	8	0	-1.836235	2.977013	1.339506
14	8	0	0.936950	-1.162238	0.670479
15	6	0	1.572765	-0.212632	0.131105
16	8	0	0.835753	0.603749	-0.647114
17	8	0	-2.216076	-1.448884	0.848369
18	6	0	-3.782750	0.322112	1.072619
19	1	0	-3.371389	0.585345	2.048868
20	1	0	-4.198172	1.219110	0.610268
21	1	0	-4.588500	-0.400670	1.206381
22	8	0	-2.142727	-2.907123	-0.963031
23	6	0	-3.121309	-3.798216	-0.441735
24	1	0	-3.556242	-4.319727	-1.291575
25	1	0	-2.657192	-4.523465	0.234787
26	1	0	-3.901427	-3.252590	0.093932
27	6	0	3.013216	-0.081051	0.089003
28	6	0	3.811772	-1.003614	0.785913
29	6	0	3.621903	0.979358	-0.602919
30	6	0	5.191932	-0.875759	0.769944
31	1	0	3.335890	-1.810436	1.329858

32	6	0	5.004226	1.100676	-0.603796
33	1	0	3.006129	1.694658	-1.133526
34	6	0	5.794750	0.176346	0.078548
35	1	0	5.803356	-1.595063	1.301778
36	1	0	5.469470	1.918839	-1.140715
37	1	0	6.873361	0.276652	0.075052
38	6	0	-2.442077	4.145312	-0.679281
39	1	0	-2.482223	5.035573	-0.057938
40	1	0	-1.739963	4.284674	-1.501504
41	1	0	-3.425178	3.944630	-1.108674

3ab-TS$_{C4}$

Standard orientation:

Center Number	Atomic Number	Atomic Type	Coordinates (Angstroms) X	Y	Z
1	6	0	-2.048768	-1.829243	0.018550
2	6	0	-1.125155	-1.458518	-1.141669
3	6	0	-1.061057	0.018573	-1.364021
4	6	0	-2.034963	0.389372	0.902091
5	1	0	-1.570803	0.445515	-2.216568
6	1	0	-3.032400	0.553584	0.479615
7	1	0	-1.834719	-2.844161	0.369734
8	1	0	-1.422716	-1.993985	-2.042338
9	6	0	-0.978979	0.839296	-0.122193
10	1	0	0.005214	0.719023	0.350915
11	8	0	-1.188642	2.210278	-0.460101
12	6	0	-0.308883	3.128036	-0.004062
13	8	0	0.643013	2.851450	0.677276
14	8	0	0.211754	-1.899755	-0.762773
15	6	0	1.112427	-0.965647	-1.129204
16	8	0	0.699216	-0.128272	-1.977895
17	8	0	-1.834174	-1.005357	1.136833
18	6	0	-1.893698	1.097849	2.231873
19	1	0	-0.897788	0.928661	2.646466
20	1	0	-2.049464	2.171049	2.108813
21	1	0	-2.640115	0.719989	2.931870
22	8	0	-3.348111	-1.732891	-0.476320
23	6	0	-4.336720	-2.148002	0.457785
24	1	0	-5.290523	-2.127843	-0.064422
25	1	0	-4.129881	-3.164656	0.806645

26	1	0	-4.372881	-1.472323	1.315824
27	6	0	2.350601	-0.876845	-0.384612
28	6	0	2.688629	-1.842644	0.578603
29	6	0	3.247112	0.170012	-0.661268
30	6	0	3.894728	-1.747734	1.258180
31	1	0	2.001227	-2.652968	0.786723
32	6	0	4.444595	0.257228	0.030435
33	1	0	2.985745	0.904799	-1.413144
34	6	0	4.775949	-0.700306	0.990965
35	1	0	4.149648	-2.492915	2.002448
36	1	0	5.127137	1.072191	-0.179500
37	1	0	5.716103	-0.631640	1.524675
38	6	0	-0.694410	4.503169	-0.458820
39	1	0	0.033438	5.224460	-0.098087
40	1	0	-0.743794	4.525948	-1.547928
41	1	0	-1.686330	4.744076	-0.073697

3ab-int$_{C3}$

Standard orientation:

Center Number	Atomic Number	Atomic Type	Coordinates (Angstroms) X	Y	Z
1	6	0	2.900047	-1.588126	-0.728978
2	6	0	1.409151	-1.601853	-0.652860
3	6	0	0.763456	-0.501401	0.112660
4	6	0	2.942812	0.714179	-0.102128
5	1	0	3.197507	0.417540	0.922751
6	1	0	3.268839	-2.209766	-1.551143
7	1	0	0.857902	-2.498585	-0.898320
8	6	0	1.422241	0.829425	-0.223322
9	1	0	1.154433	1.128832	-1.238642
10	8	0	0.967749	1.809287	0.709999
11	6	0	0.030096	2.696778	0.314089
12	8	0	-0.414845	2.740900	-0.802672
13	8	0	3.386452	-0.291017	-1.012243
14	6	0	3.646788	2.002667	-0.469775
15	1	0	3.399815	2.285581	-1.495519
16	1	0	3.343574	2.804975	0.203565
17	1	0	4.726216	1.868765	-0.390830
18	8	0	3.400243	-2.065131	0.495773
19	6	0	4.816108	-2.196528	0.510168

20	1	0	5.079179	-2.674417	1.451486
21	1	0	5.151935	-2.821669	-0.323803
22	1	0	5.301233	-1.219591	0.445732
23	6	0	-0.361800	3.593075	1.450097
24	1	0	0.527233	4.060547	1.874219
25	1	0	-1.056946	4.349238	1.096165
26	1	0	-0.829073	2.992108	2.232044
27	1	0	0.849812	-0.673360	1.194376
28	8	0	-0.625005	-0.349462	-0.208772
29	6	0	-1.491496	-1.186266	0.380553
30	8	0	-1.147493	-2.100837	1.088381
31	6	0	-2.904458	-0.845717	0.058558
32	6	0	-3.232312	0.323021	-0.631882
33	6	0	-3.904879	-1.723057	0.478083
34	6	0	-4.566301	0.607619	-0.901066
35	1	0	-2.447971	1.001206	-0.945979
36	6	0	-5.235371	-1.435528	0.202233
37	1	0	-3.628231	-2.622392	1.014840
38	6	0	-5.565337	-0.270163	-0.487035
39	1	0	-4.827031	1.514339	-1.433209
40	1	0	-6.014050	-2.116551	0.523128
41	1	0	-6.603661	-0.045487	-0.700946

3ab-int$_{C4}$

Standard orientation:

Center Number	Atomic Number	Atomic Type	Coordinates (Angstroms) X	Y	Z
1	6	0	0.581060	1.986526	-0.004765
2	6	0	0.247778	0.874691	-1.006495
3	6	0	1.375993	-0.083948	-1.109752
4	6	0	2.303158	0.709635	1.055927
5	1	0	1.598927	-0.587060	-2.040118
6	1	0	3.058957	1.330365	0.561875
7	1	0	-0.322587	2.529281	0.290307
8	1	0	-0.011582	1.316559	-1.967111
9	6	0	2.029625	-0.505932	0.159534
10	1	0	1.393837	-1.212854	0.710114
11	8	0	3.300632	-1.120085	-0.086714
12	6	0	3.293202	-2.401569	-0.497215
13	8	0	2.277785	-3.025509	-0.672343

14	8	0	-0.898896	0.141671	-0.500046
15	6	0	-2.108938	0.677128	-0.717002
16	8	0	-2.272900	1.731904	-1.281365
17	8	0	1.091283	1.452253	1.194086
18	6	0	2.755449	0.320803	2.445849
19	1	0	1.981707	-0.270122	2.940880
20	1	0	3.671641	-0.268217	2.390265
21	1	0	2.948979	1.215771	3.038467
22	8	0	1.485152	2.832278	-0.642589
23	6	0	1.825130	3.976071	0.130395
24	1	0	2.450832	4.606059	-0.497780
25	1	0	0.922867	4.524827	0.418487
26	1	0	2.376348	3.691378	1.030155
27	6	0	-3.211440	-0.174312	-0.188563
28	6	0	-2.959820	-1.393345	0.443253
29	6	0	-4.521207	0.282367	-0.340730
30	6	0	-4.022264	-2.152710	0.919669
31	1	0	-1.941014	-1.740405	0.557733
32	6	0	-5.579416	-0.479892	0.136877
33	1	0	-4.695348	1.231434	-0.832813
34	6	0	-5.329571	-1.697304	0.766941
35	1	0	-3.830790	-3.099538	1.409597
36	1	0	-6.596677	-0.127056	0.019309
37	1	0	-6.155236	-2.292012	1.139605
38	6	0	4.687215	-2.915339	-0.697817
39	1	0	5.249084	-2.817183	0.231719
40	1	0	4.651534	-3.955638	-1.008905
41	1	0	5.188352	-2.312910	-1.456660

Additional DFT studies. We performed additional DFT calculations to gain insight into the intriguing selectivity between primary and secondary radical formation during acetal cleavage. Two opposing factors primarily govern the observed selectivity. The first one is the stability of the forming radical, which is typically considered the dominant factor and generally favors formation of the intrinsically more stable secondary radical. In the case of methyl-cyclohexane, this effect accounts for a computed energy difference of 2.6 kcal/mol (Figure S17, left, X=CH_2). Notably, the presence of oxygen in 2-methyl-THP weakens this effect, reducing the difference to 1.2 kcal/mol (X=O). However, during cleavage of the acetal radical, an even more influential second factor is revealed, the strength of the C–O bond being broken. The C–O bond at the secondary carbon is stronger than that at the primary carbon and is therefore more difficult to cleave (+3.6 kcal/mol for X = O). As these two effects operate in opposite directions, the greater strength of the secondary C–O bond ultimately predominates, leading to preferential formation

of the primary radical from **IV**. This conclusion is supported by the computed activation energies (20.1 vs 23.4 kcal/mol, Figure S17, right), which favor formation of the primary radical. Moreover, if C–O bond cleavage is reversible, primary and secondary radicals may exist in equilibrium. Under such conditions, subsequent formation of the C–Ni intermediate can occur preferentially at the primary position, resulting in a product distribution governed by the Curtin–Hammett principle.

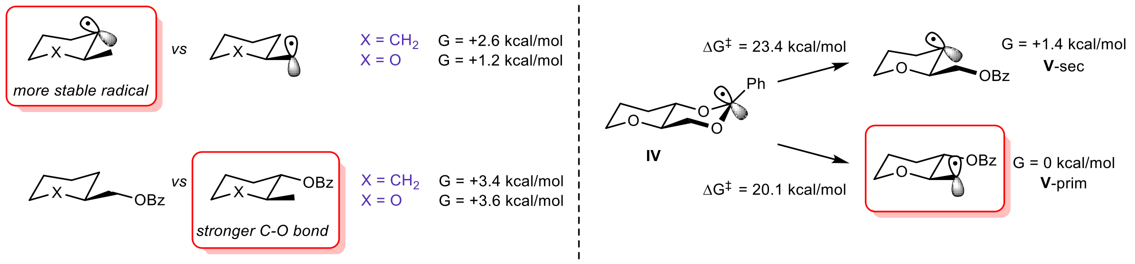

Figure S17. Additional Calculations.

Cartesian coordinates of the optimized structures are shown below:

Methyl-cyclohexane (primary radical)

Standard orientation:

Center Number	Atomic Number	Atomic Type	Coordinates (Angstroms) X	Y	Z
1	6	0	-1.151944	1.246362	-0.231470
2	6	0	-1.842007	-0.015790	0.290250
3	6	0	-1.121346	-1.274105	-0.197745
4	6	0	1.066535	0.016100	-0.331879
5	6	0	0.327707	1.269189	0.156376
6	1	0	-1.602376	-2.169722	0.204907
7	1	0	-1.834942	-0.001550	1.387082
8	1	0	-1.236678	1.275921	-1.324676
9	1	0	0.997603	-0.018093	-1.428846
10	1	0	0.417415	1.319613	1.249685
11	1	0	-1.653728	2.139763	0.149968
12	1	0	-2.889697	-0.032677	-0.022004
13	1	0	-1.198880	-1.333211	-1.290224
14	6	0	2.503910	0.032469	0.060398
15	1	0	3.222426	-0.601462	-0.444405
16	1	0	2.812969	0.522574	0.976743
17	6	0	0.356700	-1.250796	0.198811

18	1	0	0.443692	-1.263135	1.292687
19	1	0	0.870264	-2.141490	-0.174395
20	1	0	0.814603	2.162899	-0.244966

Methyl-cyclohexane (secondary radical)

Standard orientation:

Center Number	Atomic Number	Atomic Type	Coordinates (Angstroms) X	Y	Z
1	6	0	1.202577	-1.289067	-0.144537
2	6	0	1.877399	0.003545	0.339905
3	6	0	1.169800	1.232681	-0.232992
4	6	0	-1.034123	-0.027595	-0.336371
5	6	0	-0.274052	-1.247998	0.078559
6	1	0	1.654171	2.146706	0.120920
7	1	0	1.832560	0.042162	1.434217
8	1	0	1.407983	-1.395024	-1.223668
9	1	0	-1.038023	0.014174	-1.440833
10	1	0	-0.817963	-2.169161	0.261766
11	1	0	1.645180	-2.162580	0.340036
12	1	0	2.933592	0.001914	0.058038
13	1	0	1.261576	1.225384	-1.326359
14	6	0	-2.483129	-0.063420	0.144256
15	1	0	-3.028751	0.822562	-0.189479
16	1	0	-3.002256	-0.947442	-0.234099
17	1	0	-2.516926	-0.092529	1.237477
18	6	0	-0.311362	1.240875	0.148468
19	1	0	-0.403101	1.296019	1.240937
20	1	0	-0.810700	2.123689	-0.262683

Methyl-Tetrahydropyran (primary radical)

Standard orientation:

Center Number	Atomic Number	Atomic Type	Coordinates (Angstroms) X	Y	Z
1	6	0	1.172497	1.225125	0.189550
2	6	0	1.798669	-0.082553	-0.296357
3	6	0	0.984186	-1.265067	0.210303
4	6	0	-1.019648	-0.010125	0.319305
5	6	0	-0.310398	1.258885	-0.185523
6	1	0	1.355783	-2.208977	-0.189613
7	1	0	1.811382	-0.104156	-1.390964
8	1	0	1.270843	1.291255	1.279439
9	1	0	-0.940457	-0.022669	1.420266
10	1	0	-0.424642	1.301200	-1.274265
11	8	0	-0.376311	-1.173737	-0.191674
12	6	0	-2.446220	-0.058729	-0.084398
13	1	0	-2.731876	-0.620568	-0.963622
14	1	0	-3.156775	0.615594	0.373296
15	1	0	1.693453	2.086730	-0.233781
16	1	0	2.830929	-0.178085	0.050025
17	1	0	1.033504	-1.311982	1.308054
18	1	0	-0.806178	2.136338	0.237272

Methyl-Tetrahydropyran (secondary radical)

Standard orientation:

Center Number	Atomic Number	Atomic Type	Coordinates (Angstroms) X	Y	Z
1	6	0	1.257438	1.238755	0.118994
2	6	0	1.828312	-0.105996	-0.350646
3	6	0	0.998625	-1.246530	0.222836
4	6	0	-0.993822	0.031085	0.332923
5	6	0	-0.226463	1.257917	-0.045052
6	1	0	1.352147	-2.214375	-0.134153
7	1	0	1.794801	-0.162649	-1.442536
8	1	0	1.508680	1.369585	1.184927

9	1	0	-1.008869	-0.060196	1.436248
10	1	0	-0.760962	2.197200	-0.126843
11	8	0	-0.360566	-1.146200	-0.176247
12	1	0	1.721962	2.072576	-0.411356
13	1	0	2.870112	-0.215088	-0.039586
14	1	0	1.056996	-1.241287	1.321617
15	6	0	-2.419618	0.039902	-0.184901
16	1	0	-2.937087	-0.873628	0.111192
17	1	0	-2.960806	0.896807	0.220400
18	1	0	-2.419280	0.109861	-1.274864

Cyclohexylmethyl benzoate (primary benzoate)

Standard orientation:

Center Number	Atomic Number	Atomic Type	Coordinates (Angstroms) X	Y	Z
1	6	0	-3.462548	1.758008	0.398498
2	6	0	-4.766346	0.957765	0.372066
3	6	0	-4.688388	-0.180119	-0.647987
4	6	0	-2.179210	-0.290246	-0.346289
5	1	0	-5.610692	-0.766632	-0.636307
6	1	0	-4.948390	0.535195	1.367782
7	1	0	-3.323433	2.251330	-0.571157
8	1	0	-2.019429	0.143212	-1.342672
9	8	0	1.367844	-2.301402	0.382302
10	6	0	1.330432	-1.124771	0.107200
11	8	0	0.198701	-0.466373	-0.139151
12	6	0	-1.022383	-1.224041	-0.052877
13	1	0	-1.100570	-1.647049	0.953144
14	1	0	-0.974767	-2.045756	-0.771479
15	6	0	2.536665	-0.252128	0.007273
16	6	0	3.788125	-0.834679	0.209634
17	6	0	2.434468	1.110694	-0.276590
18	6	0	4.936218	-0.057084	0.126455
19	1	0	3.847040	-1.893581	0.429796
20	6	0	3.585773	1.885819	-0.357850
21	1	0	1.459766	1.555660	-0.430745

22	6	0	4.834868	1.303269	-0.157161
23	1	0	5.908208	-0.508958	0.282434
24	1	0	3.509026	2.943774	-0.577323
25	1	0	5.730409	1.909994	-0.221648
26	1	0	-5.610806	1.613631	0.144220
27	1	0	-4.592217	0.244406	-1.654523
28	1	0	-3.515191	2.547900	1.152313
29	6	0	-3.488007	-1.088027	-0.371973
30	1	0	-3.429070	-1.880128	-1.124642
31	1	0	-3.623019	-1.578482	0.601083
32	6	0	-2.263253	0.848994	0.674645
33	1	0	-1.334861	1.425802	0.667083
34	1	0	-2.362841	0.411155	1.677185

2-Methylcyclohexyl benzoate (secondary benzoate)

Standard orientation:

Center Number	Atomic Number	Atomic Type	Coordinates (Angstroms) X	Y	Z
1	6	0	2.022813	-1.219675	-0.792349
2	6	0	3.514648	-1.487499	-0.582375
3	6	0	4.317851	-0.190954	-0.695768
4	6	0	2.297083	1.142378	0.086709
5	6	0	1.528328	-0.169943	0.194031
6	1	0	5.377502	-0.382495	-0.509030
7	1	0	3.668346	-1.923676	0.411618
8	1	0	1.852420	-0.842639	-1.807266
9	1	0	2.148390	1.525192	-0.931698
10	1	0	1.596543	-0.553145	1.217820
11	8	0	0.138788	0.132643	-0.074539
12	6	0	-0.789393	-0.678655	0.431157
13	8	0	-0.539549	-1.658887	1.093774
14	6	0	-2.175898	-0.237596	0.094600
15	6	0	-2.419706	0.934315	-0.623345
16	6	0	-3.240407	-1.031103	0.523210
17	6	0	-3.727928	1.307978	-0.909753
18	1	0	-1.588737	1.545415	-0.951463
19	6	0	-4.546125	-0.655234	0.234033

20	1	0	-3.031475	-1.936250	1.080205
21	6	0	-4.789876	0.514530	-0.482592
22	1	0	-3.919277	2.217674	-1.465849
23	1	0	-5.372642	-1.271694	0.565967
24	1	0	-5.808422	0.807926	-0.708020
25	1	0	3.863705	-2.223377	-1.310333
26	1	0	4.236537	0.195241	-1.718567
27	1	0	1.440316	-2.135829	-0.673311
28	6	0	3.791972	0.859256	0.283475
29	1	0	4.347577	1.795472	0.179852
30	1	0	3.950540	0.509596	1.312159
31	6	0	1.790274	2.174752	1.090297
32	1	0	0.733336	2.397562	0.934790
33	1	0	1.915267	1.803981	2.112567
34	1	0	2.354337	3.105700	0.998699

(Tetrahydro-pyran-2-yl)methyl benzoate (primary benzoate)

Standard orientation:

Center Number	Atomic Number	Atomic Type	Coordinates (Angstroms) X	Y	Z
1	6	0	-3.530392	1.733187	0.415197
2	6	0	-4.760873	0.827489	0.341476
3	6	0	-4.520117	-0.291623	-0.662034
4	6	0	-2.181218	-0.236763	-0.337202
5	1	0	-5.342524	-1.006978	-0.673572
6	1	0	-4.956903	0.386154	1.324017
7	1	0	-3.411523	2.260876	-0.538297
8	1	0	-2.041361	0.192895	-1.341553
9	8	0	1.324227	-2.296589	0.369601
10	6	0	1.300455	-1.118426	0.105961
11	8	0	0.169936	-0.447782	-0.131868
12	6	0	-1.046173	-1.200208	-0.051153
13	1	0	-1.146067	-1.629082	0.948890
14	1	0	-1.027983	-2.007749	-0.784866
15	6	0	2.511074	-0.254117	0.009573
16	6	0	3.757908	-0.847375	0.209899
17	6	0	2.418835	1.110297	-0.270181

18	6	0	4.911717	-0.078294	0.129091
19	1	0	3.808814	-1.907410	0.426516
20	6	0	3.576076	1.876560	-0.349228
21	1	0	1.447916	1.563768	-0.423123
22	6	0	4.820563	1.283644	-0.150400
23	1	0	5.880206	-0.538040	0.283541
24	1	0	3.507498	2.935649	-0.565718
25	1	0	5.720702	1.883671	-0.213262
26	1	0	-5.649217	1.392108	0.047421
27	1	0	-4.407724	0.125302	-1.673037
28	1	0	-3.653424	2.492919	1.189744
29	6	0	-2.282152	0.890719	0.684893
30	1	0	-1.376547	1.500190	0.642544
31	1	0	-2.340663	0.444769	1.684801
32	8	0	-3.352341	-1.038077	-0.336407

2-Methyltetrahydro-pyran-3-yl benzoate (secondary benzoate)

Standard orientation:

Center Number	Atomic Number	Atomic Type	Coordinates (Angstroms) X	Y	Z
1	6	0	2.040223	-1.238452	-0.785547
2	6	0	3.550910	-1.406563	-0.612551
3	6	0	4.228289	-0.045254	-0.668572
4	6	0	2.307193	1.099727	0.115278
5	6	0	1.529613	-0.213961	0.217769
6	1	0	5.294675	-0.118617	-0.455538
7	1	0	3.768525	-1.873858	0.352636
8	1	0	1.819693	-0.868703	-1.793288
9	1	0	2.158949	1.505462	-0.897590
10	1	0	1.605997	-0.599127	1.238732
11	8	0	0.155018	0.115523	-0.053128
12	6	0	-0.787030	-0.706934	0.418318
13	8	0	-0.544360	-1.710204	1.045537
14	6	0	-2.165187	-0.241078	0.088829
15	6	0	-2.392657	0.950970	-0.600866
16	6	0	-3.239838	-1.032798	0.495151
17	6	0	-3.695542	1.346572	-0.881449

18	1	0	-1.554088	1.560429	-0.912107
19	6	0	-4.540015	-0.634371	0.212115
20	1	0	-3.043296	-1.953883	1.030055
21	6	0	-4.767747	0.555418	-0.476285
22	1	0	-3.874622	2.271727	-1.415605
23	1	0	-5.374680	-1.248788	0.526847
24	1	0	-5.782111	0.866399	-0.696843
25	1	0	3.955310	-2.054136	-1.393576
26	1	0	4.101296	0.401379	-1.664682
27	1	0	1.516372	-2.186299	-0.651515
28	6	0	1.880085	2.114869	1.154749
29	1	0	0.824262	2.360475	1.035266
30	1	0	2.037569	1.710377	2.157512
31	1	0	2.468051	3.027392	1.048704
32	8	0	3.687882	0.833542	0.311260

V-prim

Standard orientation:

Center Number	Atomic Number	Atomic Type	Coordinates (Angstroms) X	Y	Z
1	6	0	2.255838	-1.351350	0.793993
2	6	0	3.762567	-1.108490	0.895409
3	6	0	4.270131	-0.471173	-0.389804
4	6	0	2.179571	0.520949	-0.893859
5	6	0	1.566107	-0.056091	0.401256
6	1	0	5.321594	-0.195481	-0.312135
7	1	0	3.978564	-0.440346	1.734467
8	1	0	2.048013	-2.104261	0.025622
9	1	0	2.038871	-0.233755	-1.683933
10	1	0	1.644932	0.691240	1.192673
11	8	0	3.568137	0.733203	-0.679591
12	8	0	0.181714	-0.347650	0.153504
13	6	0	-0.750153	0.511707	0.587390
14	8	0	-0.505366	1.511343	1.217656
15	6	0	1.561964	1.807840	-1.297670
16	1	0	1.915735	2.724864	-0.845791
17	1	0	0.679946	1.824121	-1.921232

18	6	0	-2.128463	0.086345	0.206309
19	6	0	-2.363328	-1.067123	-0.543896
20	6	0	-3.196466	0.884019	0.619147
21	6	0	-3.666494	-1.419019	-0.876740
22	1	0	-1.530599	-1.680703	-0.861941
23	6	0	-4.496912	0.528754	0.284775
24	1	0	-2.994449	1.775728	1.199763
25	6	0	-4.731840	-0.622878	-0.463496
26	1	0	-3.851039	-2.313562	-1.459006
27	1	0	-5.326086	1.147409	0.605647
28	1	0	-5.746394	-0.899806	-0.724865
29	1	0	1.846190	-1.715801	1.738397
30	1	0	4.290319	-2.047755	1.074251
31	1	0	4.153385	-1.168006	-1.231353

V-sec

Standard orientation:

Center Number	Atomic Number	Atomic Type	Coordinates (Angstroms) X	Y	Z
1	6	0	3.307454	1.710392	0.758010
2	6	0	4.589813	0.928514	0.441908
3	6	0	2.044458	-0.328249	-0.121615
4	6	0	2.158666	1.157609	-0.018299
5	1	0	4.940827	1.181261	-0.562677
6	1	0	3.098650	1.616330	1.836622
7	1	0	1.679019	-0.746430	0.834694
8	1	0	1.278975	1.757240	-0.218990
9	8	0	-0.679833	-1.916023	0.388138
10	6	0	-1.009741	-0.905078	-0.182908
11	8	0	-0.209116	-0.241959	-1.028018
12	6	0	1.105280	-0.771760	-1.236170
13	1	0	1.438306	-0.360651	-2.188258
14	1	0	1.068150	-1.859499	-1.287062
15	6	0	-2.343640	-0.256931	-0.035710
16	6	0	-3.276136	-0.863514	0.806767
17	6	0	-2.667336	0.922262	-0.709028
18	6	0	-4.531293	-0.293282	0.975005

19	1	0	-3.005851	-1.777629	1.321067
20	6	0	-3.924665	1.489841	-0.537269
21	1	0	-1.938910	1.387260	-1.360538
22	6	0	-4.855318	0.883570	0.303006
23	1	0	-5.255976	-0.763509	1.628351
24	1	0	-4.178711	2.404608	-1.058686
25	1	0	-5.834502	1.328701	0.434604
26	1	0	5.382632	1.181967	1.149522
27	1	0	3.435628	2.776374	0.560508
28	6	0	4.311971	-0.566619	0.500591
29	1	0	5.192627	-1.148404	0.228668
30	1	0	4.002452	-0.857648	1.514713
31	8	0	3.293901	-0.937081	-0.422403

References

1. Oderinde, M. S.; Johannes, J. W. Practical syntheses of [2,2′-bipyridine]bis[3,5-difluoro-2-[5-(trifluoromethyl)-2-pyridinyl]phenyl]iridium(III) hexafluorophosphate, [Ir{dF(CF_3)ppy}$_2$(bpy)]PF_6 and [4,4′-bis(tert-butyl)-2,2′-bipyridine]bis[3,5-difluoro-2-[5-(trifluoromethyl)-2-pyridinyl]phenyl]iridium(III) hexafluorophosphate, [Ir{dF(CF_3)ppy}$_2$(dtbbpy)]PF_6. *Org. Synth.* **2017**, *94*, 77-92.
2. Pulcinella, A.; Bonciolini, S.; Lukas, F.; Sorato, A.; Noël, T. Photocatalytic alkylation of C(sp^3)-H bonds using sulfonylhydrazones. *Angew. Chem.Int. Ed.* **2023**, *62*, e202215374.
3. Mong, K.-K. T.; Chao, C.-S.; Chen, M.-C.; Lin, C.-W. Tandem one-pot acetalation-acetylation for direct access to differentially protected thioglycosides and O-glycosides with p-toluenesulfonic acid. *Synlett*, **2009**, *2009*, 603−606.
4. Demchenko, A. V.; Pornsuriyasak, P.; De Meo, C. Acetal protecting groups in the organic laboratory: synthesis of methyl-4, 6-O-benzylidene-α-D-glucopyranoside. *J. Chem. Educ.* **2006**, *83*, 782−784.
5. Roën, A.; Padrón, J. I.; Vázquez, J. T. Hydroxymethyl rotamer populations in disaccharides. *J. Org. Chem.* **2003**, *68*, 4615-4630.
6. Santra, A.; Guchhait, G.; Misra, A. K. Efficient acylation and sulfation of carbohydrates using sulfamic acid, a mild, eco-friendly catalyst under organic solvent-free conditions. *Green Chem.* **2011**, *13*, 1345–1351.
7. Maki, Y.; Nomura, K.; Okamoto, R.; Izumi, M.; Mizutani, Y.; Kajihara, Y. Acceleration and deceleration factors on the hydrolysis reaction of 4,6-O-benzylidene acetal group. *J. Org. Chem.* **2020**, *85*, 15849−15856.
8. Godage, H. Y.; Chambers, D. J.; Evans, G. R.; Fairbanks, A. Stereoselective synthesis of C-glycosides from carboxylic acids: the tandem Tebbe-Claisen approach. *Org. Biomol. Chem.* **2003**, *1*, 3772-3786.
9. Alvarez, E.; Pérez, R.; Rico, M.; Rodríguez, R. M.; Martín, J. D. Simple designs for the construction of complex trans-fused polyether toxin frameworks. A convergent strategy based on hydroxy ketone cyclization of C-linked oxacycles. *J. Org. Chem.* **1996**, *61*, 3003-3016.
10. Danieli, E.; Lalot, J.; Murphy, P. V. Selective protecting group manipulations on the 1-deoxynojirimycin scaffold. *Tetrahedron* **2007**, *63*, 6827–6834.
11. Yuasa, H.; Tamura, J.; Hashimoto, H. Synthesis of per-O-alkylated 5-thio-D-glucono-1,5-lactones and transannular participation of the ring sulphur atom of 5-thio-D-glucose derivatives on solvolysis under acidic conditions. *J. Chem. Soc., Perkin Trans. 1* **1990**, 2763-2769.
12. Tamaruya, Y., Suzuki, M., Kamura, G., Kanai, M., Hama, K., Shimizu, K., Aoki, J., Arai, H. & Shibasaki, M. Identifying specific conformations by using a carbohydrate scaffold: discovery of subtype-selective LPA-receptor agonists and an antagonist. *Angew. Chem., Int. Ed.* **2004**, *43*, 2834-2837.

13.Gaussian 16, Revision A.03, Frisch, M. J.; Trucks, G. W.; Schlegel, H. B.; Scuseria, G. E.; Robb, M. A.; Cheeseman, J. R.; Scalmani, G.; Barone, V.; Petersson, G. A.; Nakatsuji, H.; Li, X.; Caricato, M.; Marenich, A. V.; Bloino, J.; Janesko, B. G.; Gomperts, R.; Mennucci, B.; Hratchian, H. P.; Ortiz, J. V.; Izmaylov, A. F.; Sonnenberg, J. L.; Williams-Young, D.; Ding, F.; Lipparini, F.; Egidi, F.; Goings, J.; Peng, B.; Petrone, A.; Henderson, T.; Ranasinghe, D.; Zakrzewski, V. G.; Gao, J.; Rega, N.; Zheng, G.; Liang, W.; Hada, M.; Ehara, M.; Toyota, K.; Fukuda, R.; Hasegawa, J.; Ishida, M.; Nakajima, T.; Honda, Y.; Kitao, O.; Nakai, H.; Vreven, T.; Throssell, K.; Montgomery, J. A., Jr.; Peralta, J. E.; Ogliaro, F.; Bearpark, M. J.; Heyd, J. J.; Brothers, E. N.; Kudin, K. N.; Staroverov, V. N.; Keith, T. A.; Kobayashi, R.; Normand, J.; Raghavachari, K.; Rendell, A. P.; Burant, J. C.; Iyengar, S. S.; Tomasi, J.; Cossi, M.; Millam, J. M.; Klene, M.; Adamo, C.; Cammi, R.; Ochterski, J. W.; Martin, R. L.; Morokuma, K.; Farkas, O.; Foresman, J. B.; Fox, D. J. Gaussian, Inc., Wallingford CT, **2016**.

14. Zhao, Y.; Truhlar, D. G. The M06 suite of density functionals for main group thermochemistry, thermochemical kinetics, noncovalent interactions, excited states, and transition elements: two new functionals and systematic testing of four M06-class functionals and 12 other functionals. *Theor. Chem. Acc.*, **2008**, *120*, 215-241.

15. (a) Cancés, E.; Mennucci, B.; Tomasi, J. A new integral equation formalism for the polarizable continuum model: Theoretical background and applications to isotropic and anisotropic dielectrics. *J. Chem. Phys.*, **1997**, *107*, 3032-3041; (b) Cossi, M.; Barone, V.; Mennucci, B.; Tomasi, J. Ab initio study of ionic solutions by a polarizable continuum dielectric model. *Chem. Phys. Lett.*, **1998**, *286*, 253-260; (c) Tomasi, J.; Mennucci, B.; Cancès, E. The IEF version of the PCM solvation method: an overview of a new method addressed to study molecular solutes at the QM ab initio level. *J. Mol.Struc.: THEOCHEM*, **1999**, *464*, 211-226.

NMR Spectra

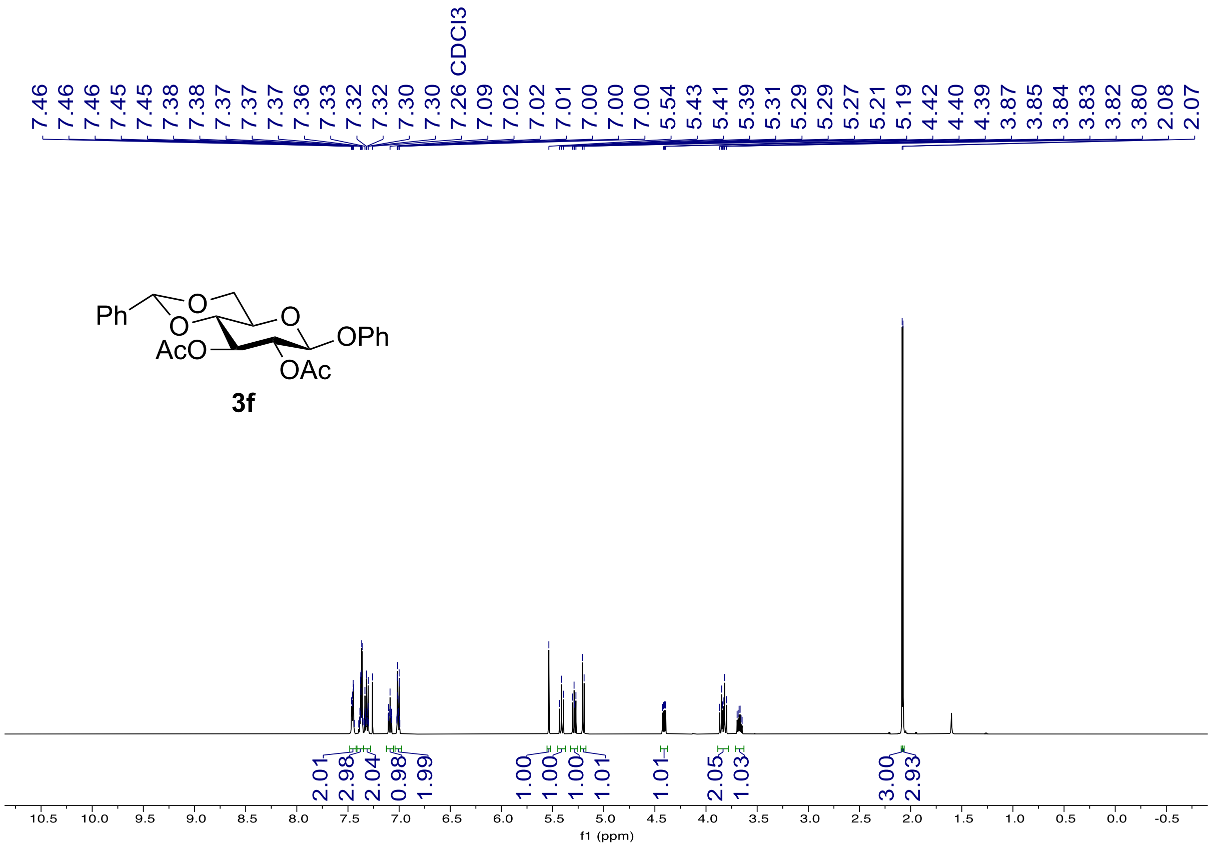

^{1}H NMR (500 MHz, $CDCl_3$) of 3f

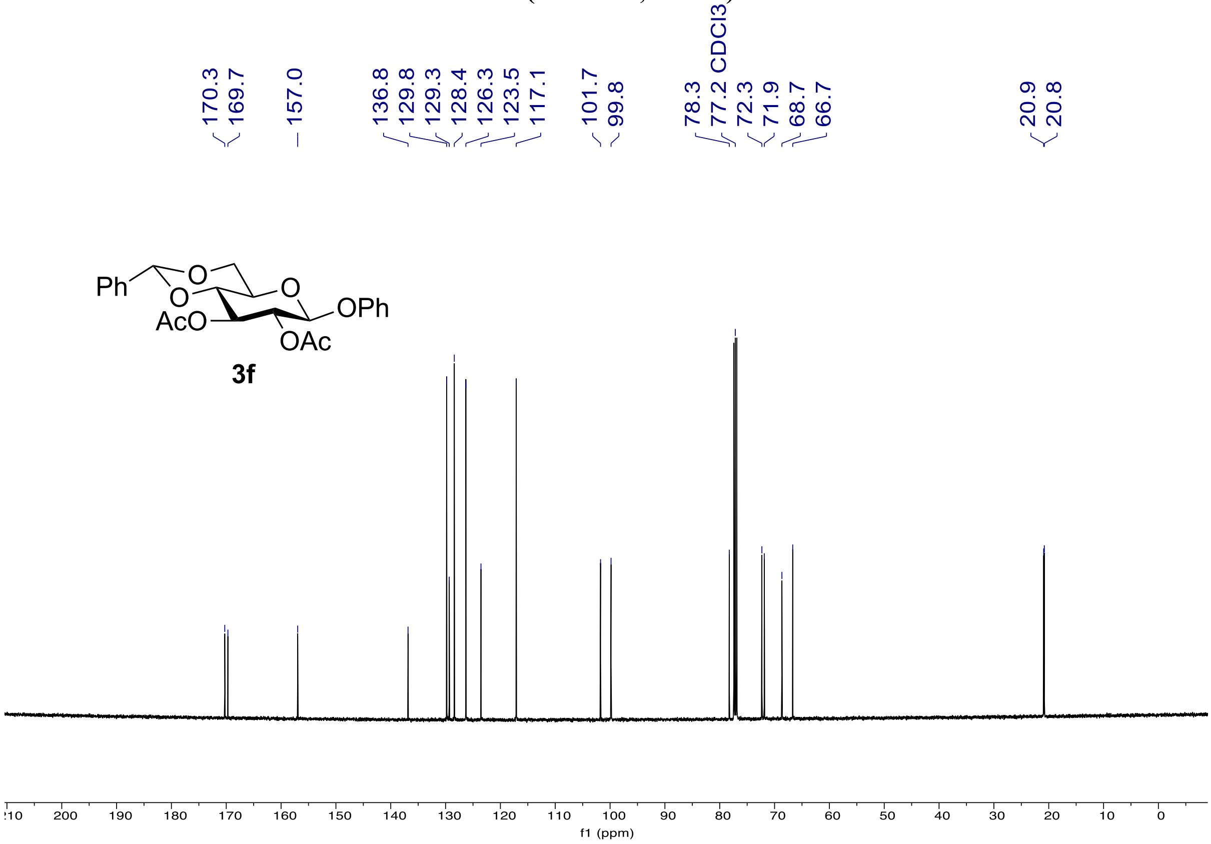

^{13}C NMR (126 MHz, $CDCl_3$) of 3f

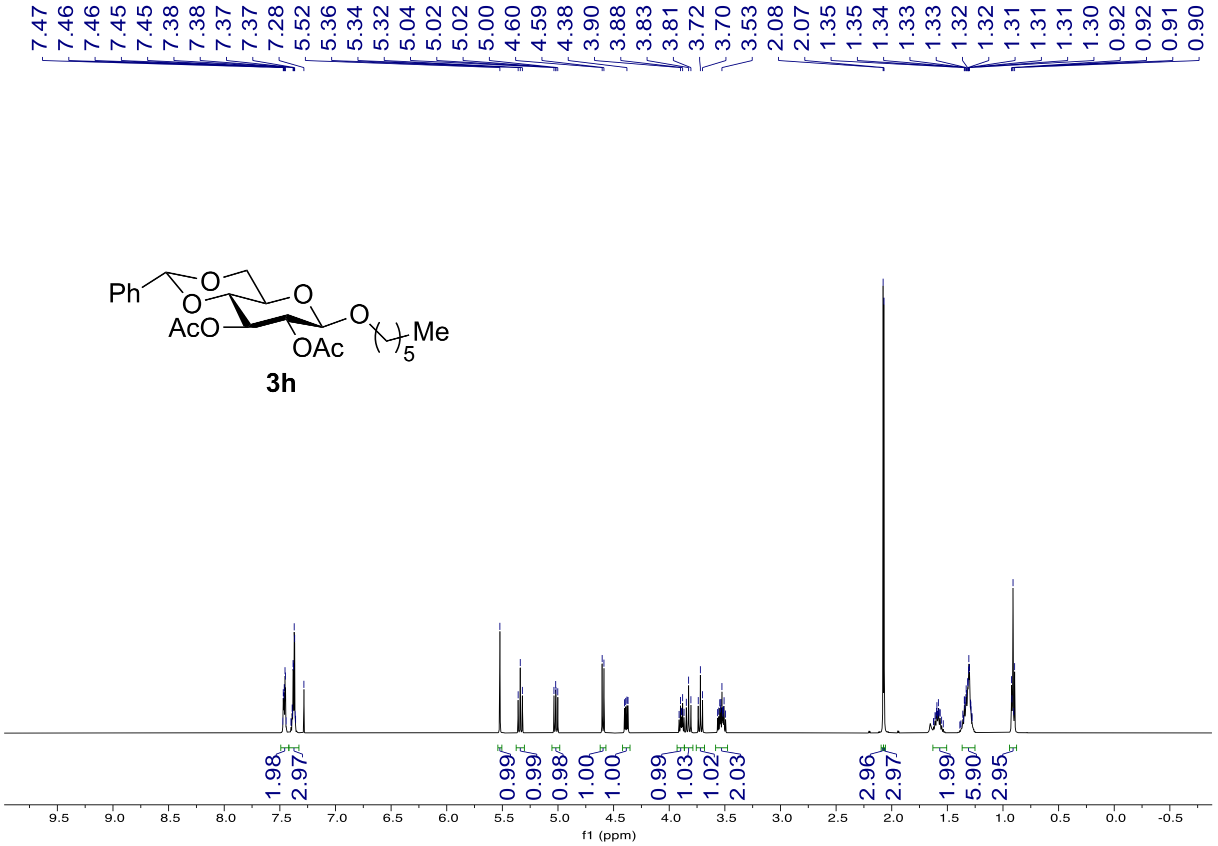

^{1}H NMR (500 MHz, $CDCl_3$) of 3h

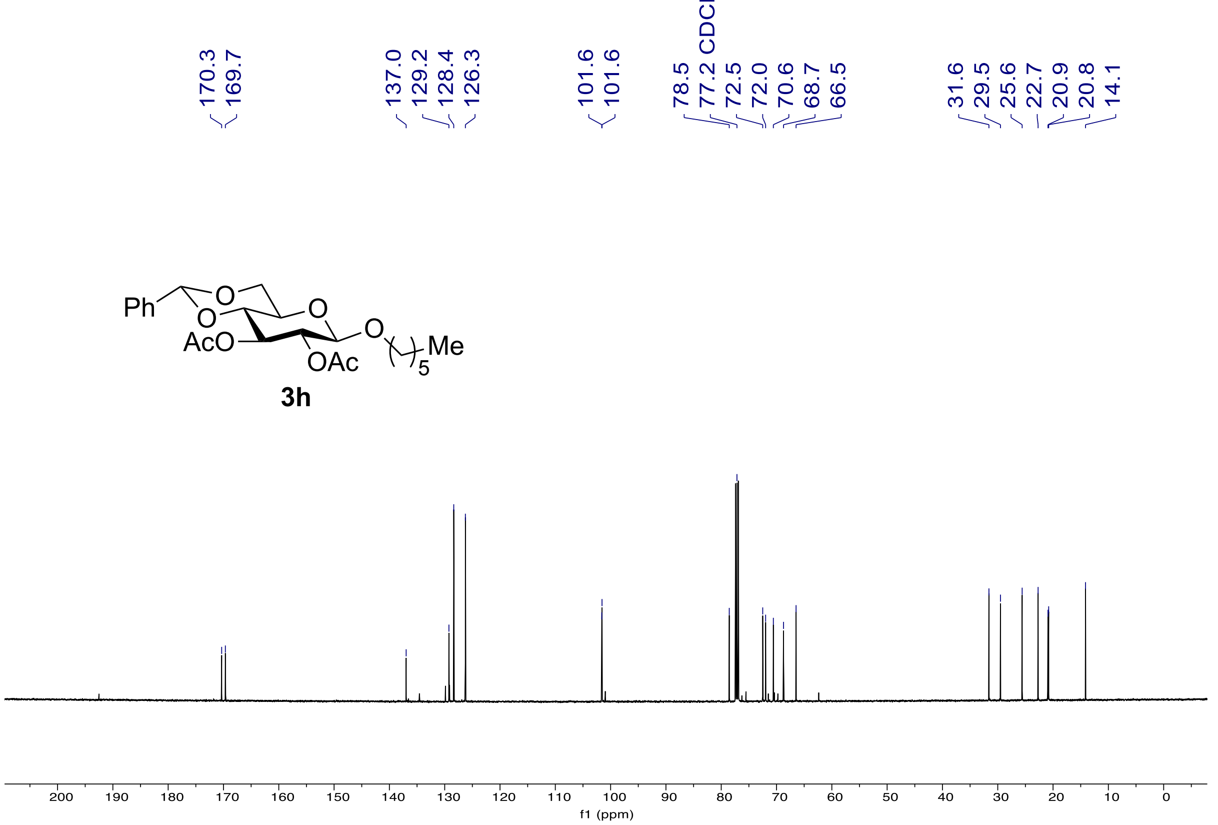

^{13}C NMR (126 MHz, $CDCl_3$) of 3h

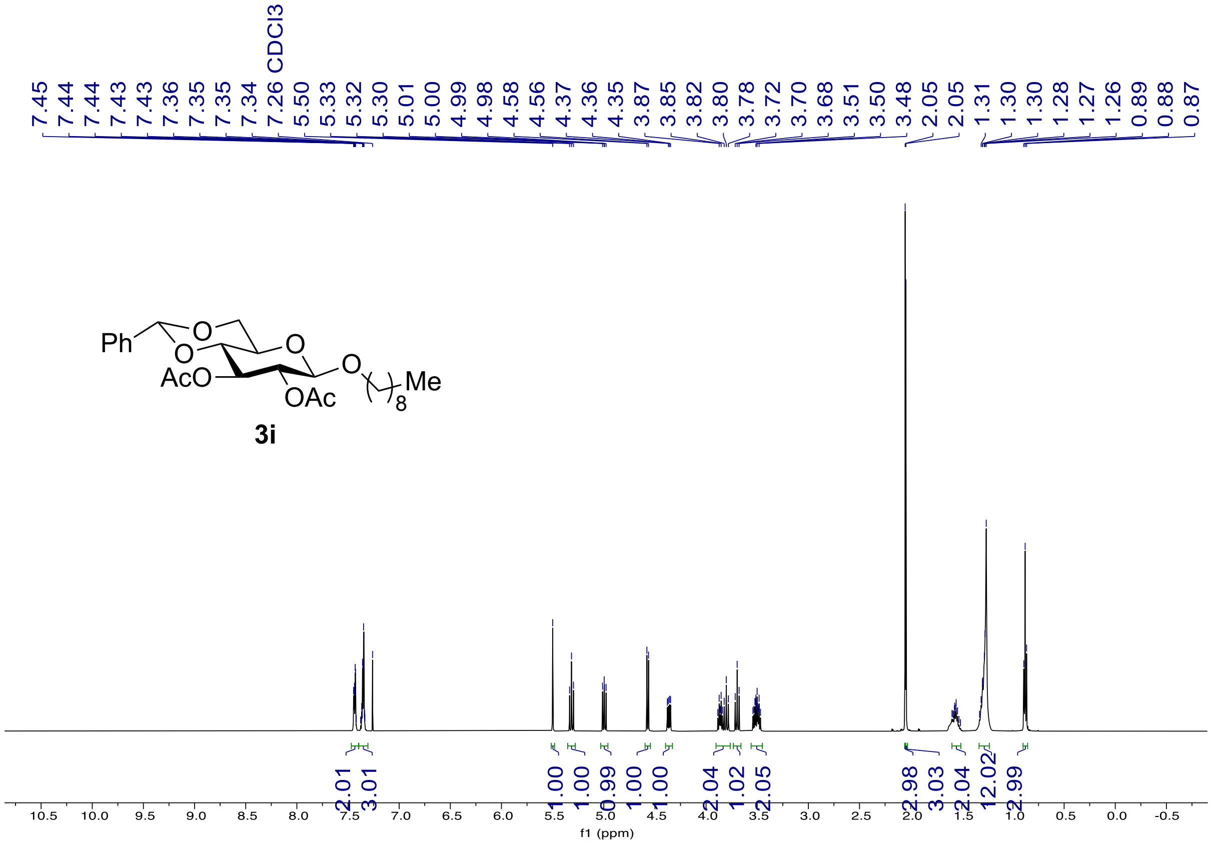

^{1}H NMR (500 MHz, CDCl$_3$) of 3i

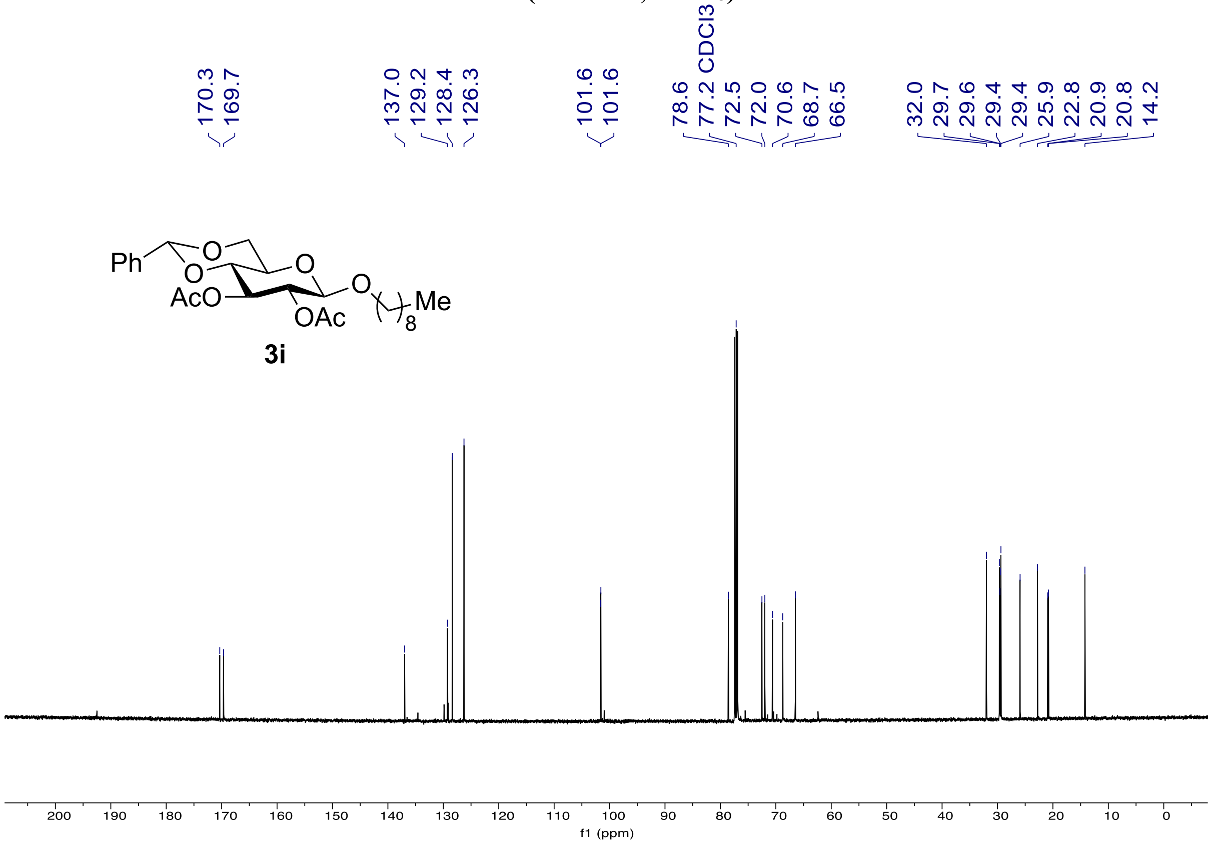

^{13}C NMR (126 MHz, CDCl$_3$) of 3i

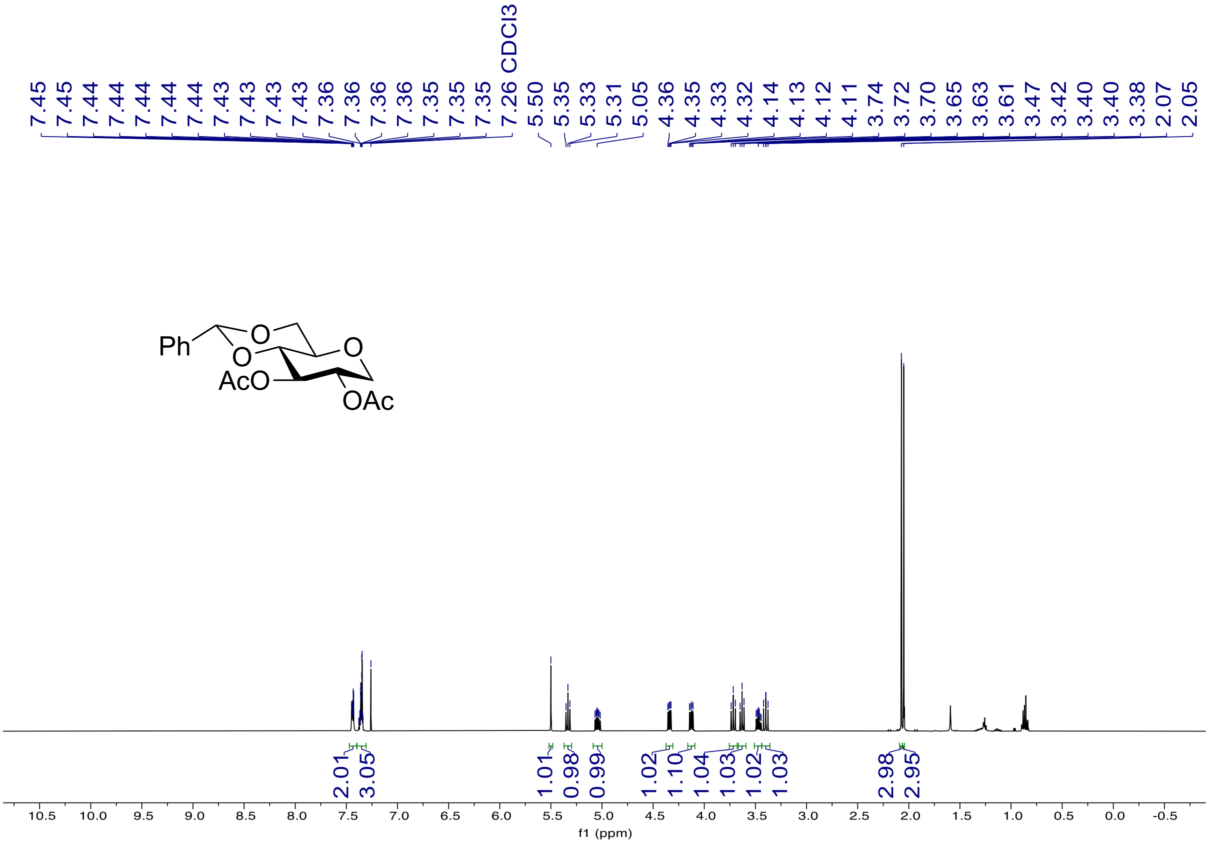

^{1}H NMR (500 MHz, $CDCl_3$) of 3p

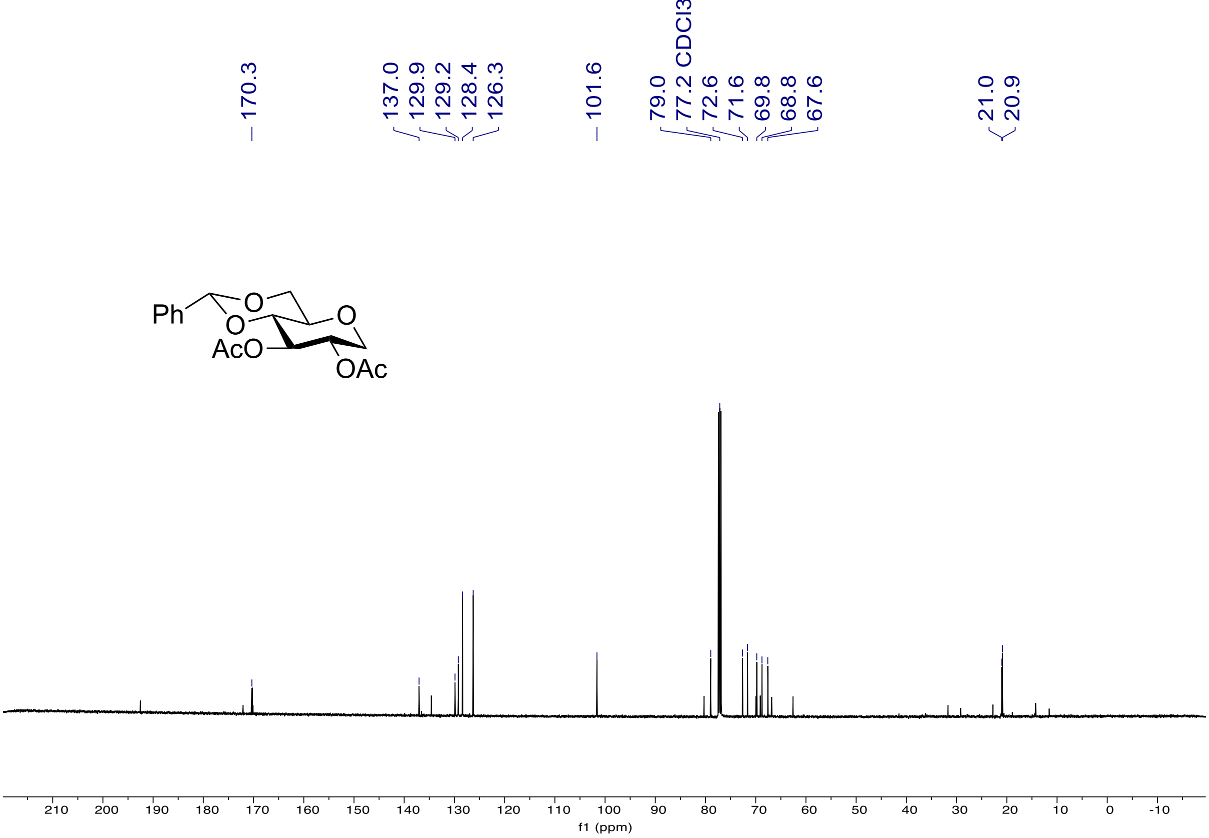

^{13}C NMR (126 MHz, $CDCl_3$) of 3p

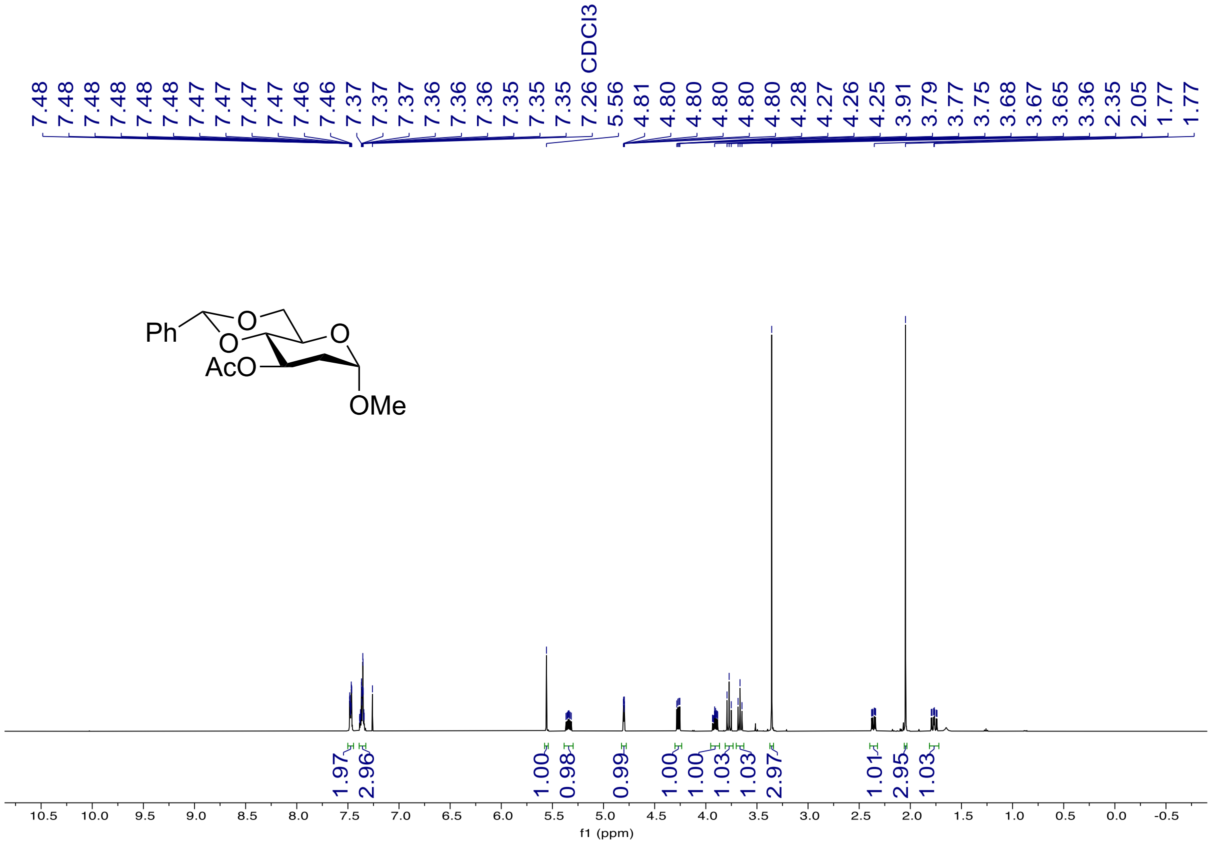

^{1}H NMR (500 MHz, $CDCl_3$) of 3q

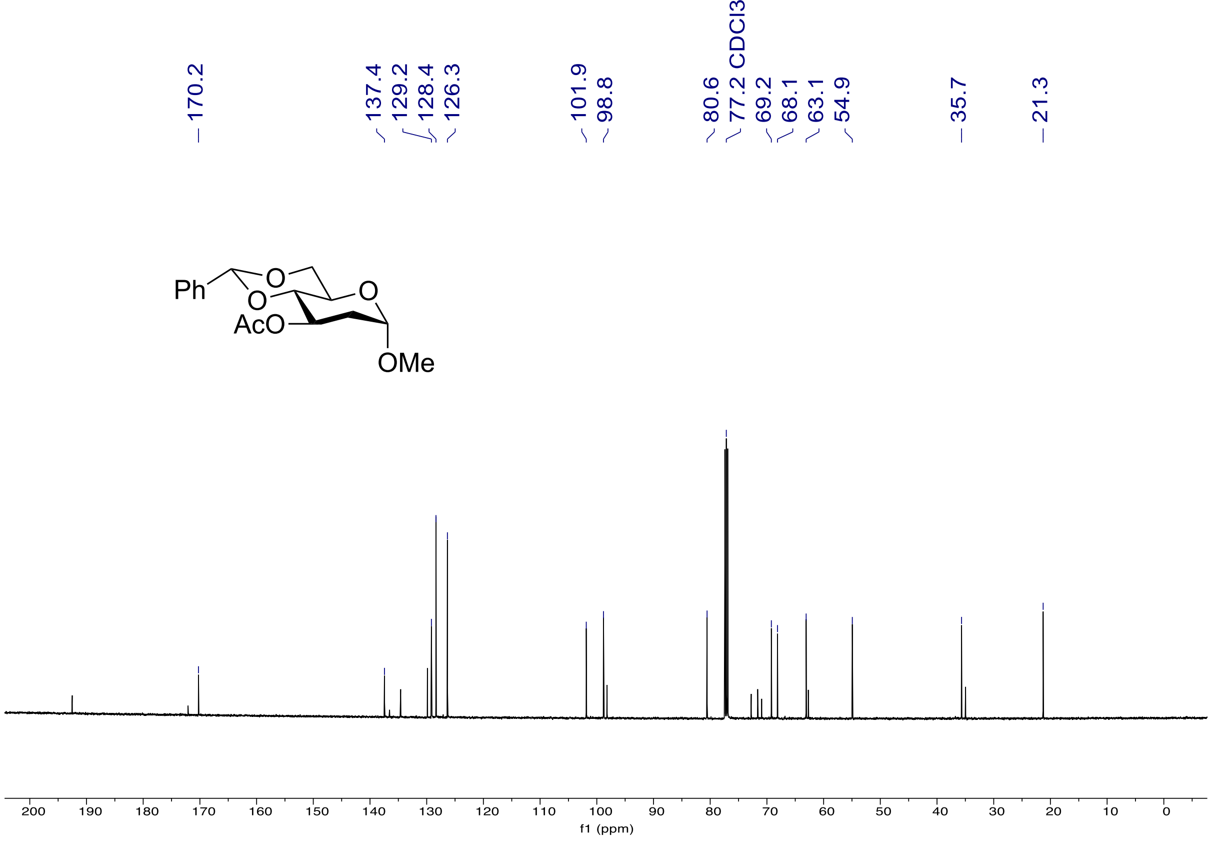

^{13}C NMR (126 MHz, $CDCl_3$) of 3q

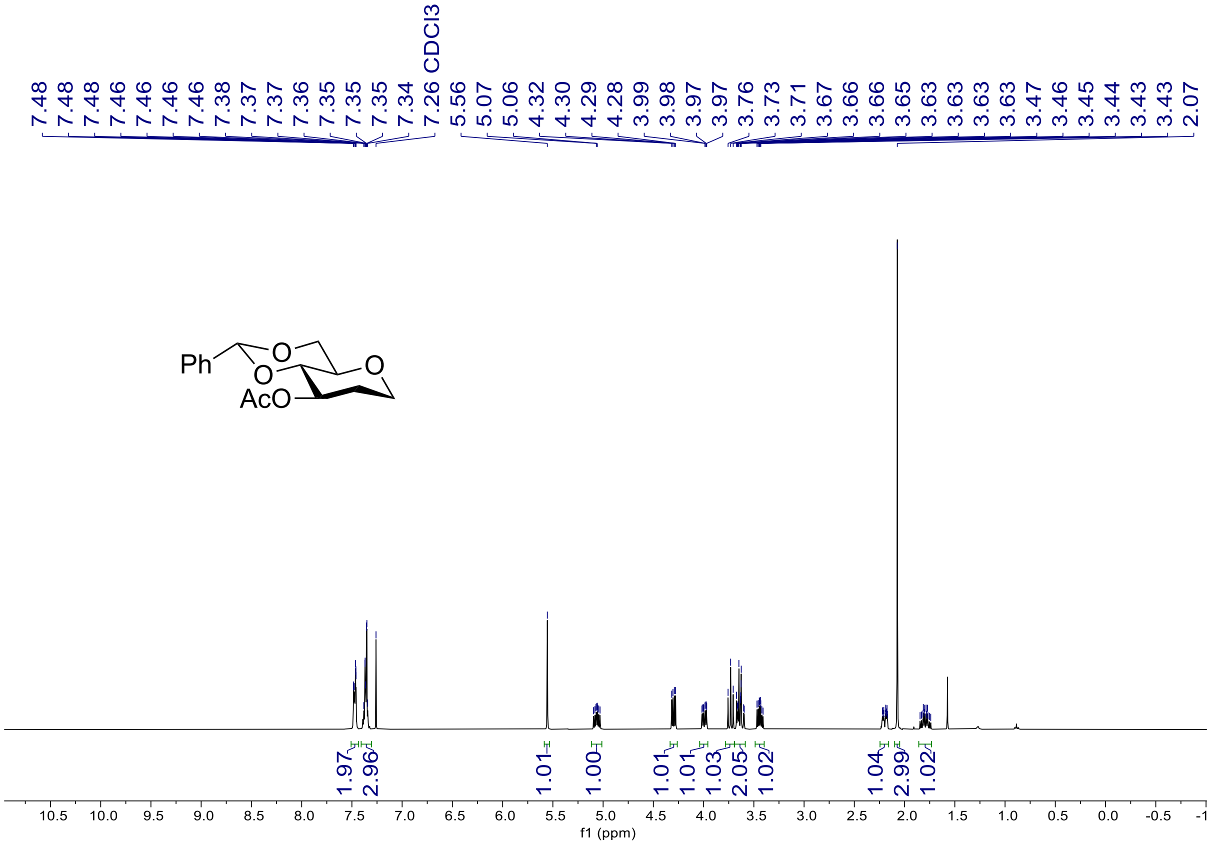

^{1}H NMR (400 MHz, CDCl$_3$) of 3r

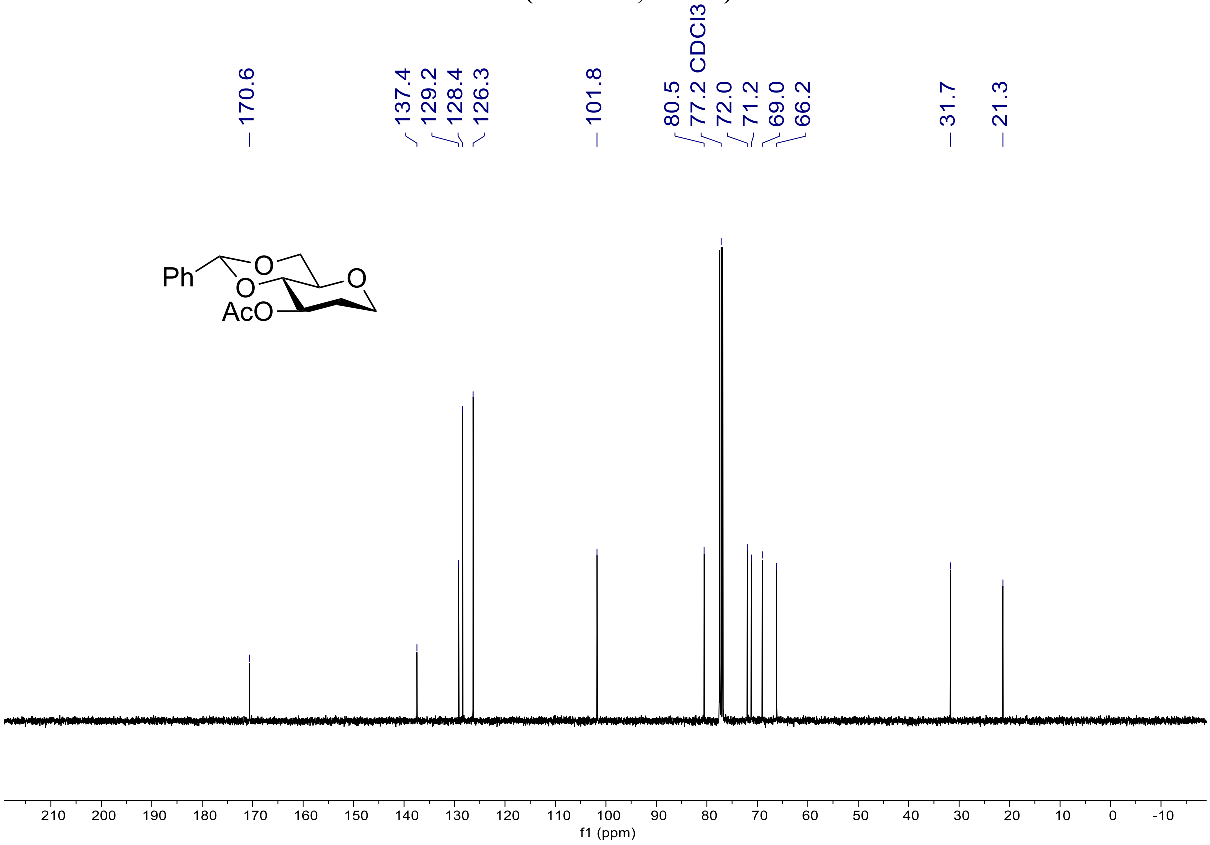

^{13}C NMR (101 MHz, CDCl$_3$) of 3o

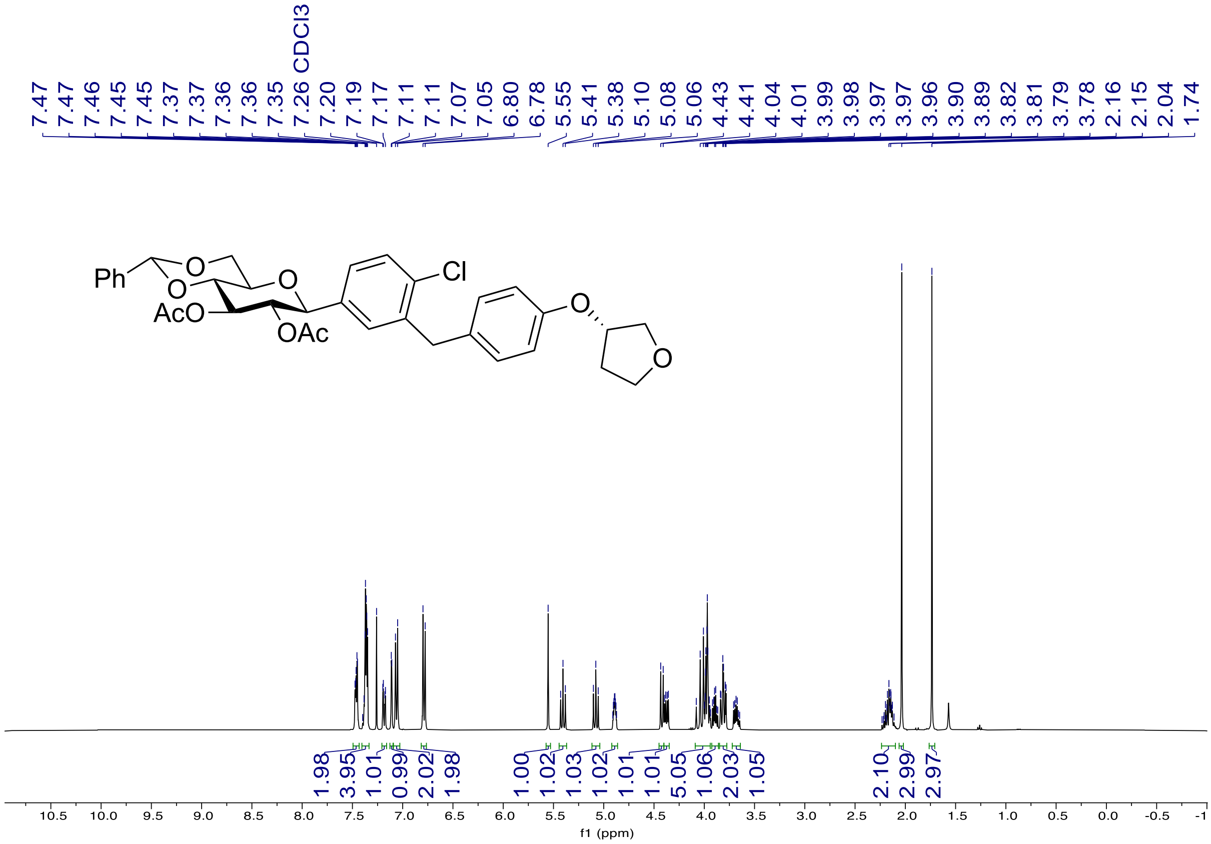

^{1}H NMR (400 MHz, $CDCl_3$) of 3w

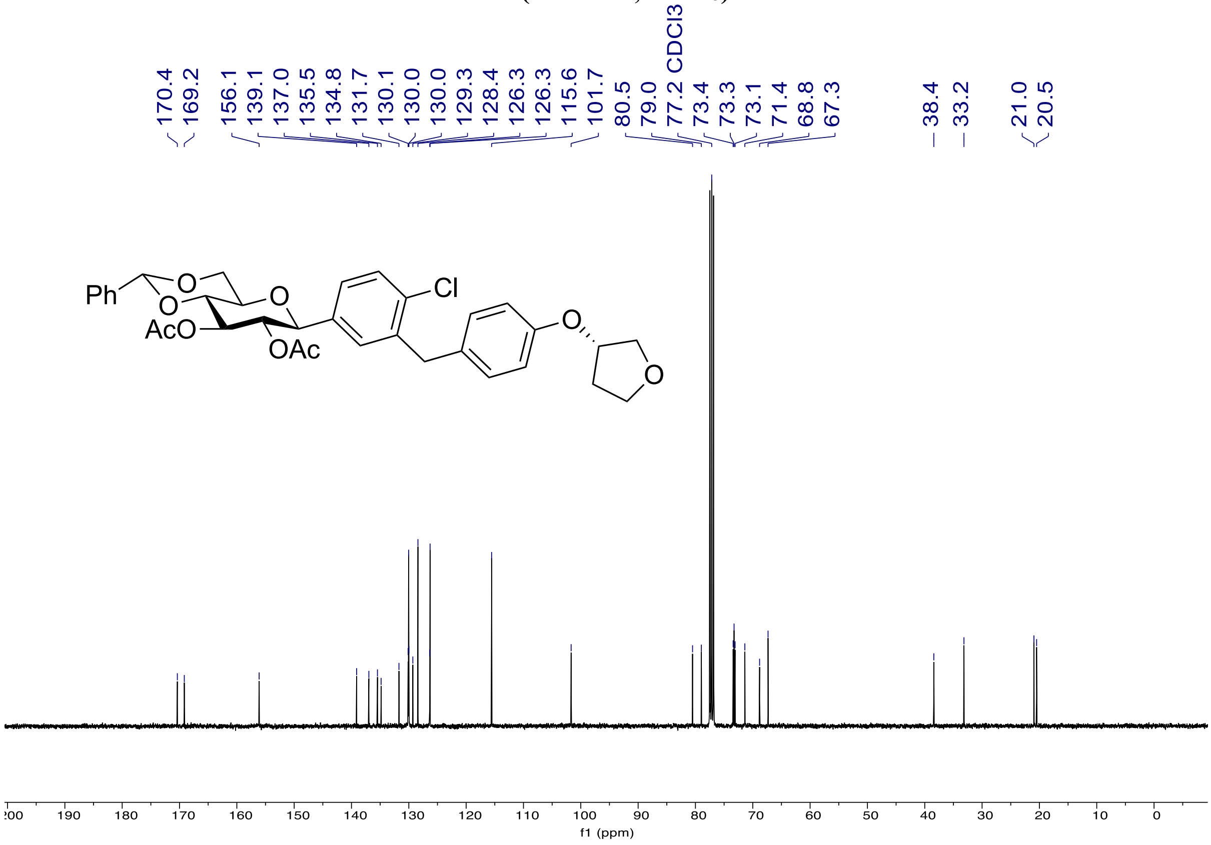

^{13}C NMR (101 MHz, $CDCl_3$) of 3w

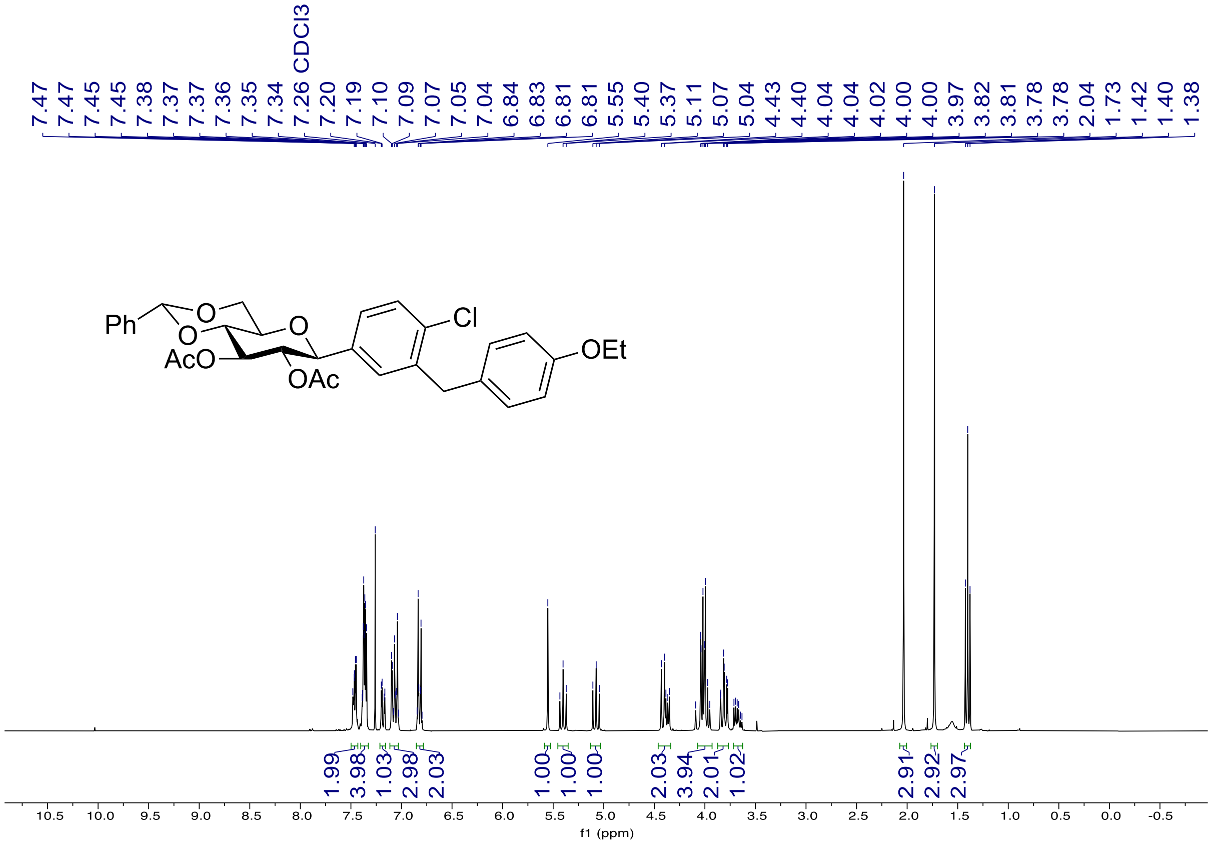

^{1}H NMR (300 MHz, CDCl$_3$) of 3x

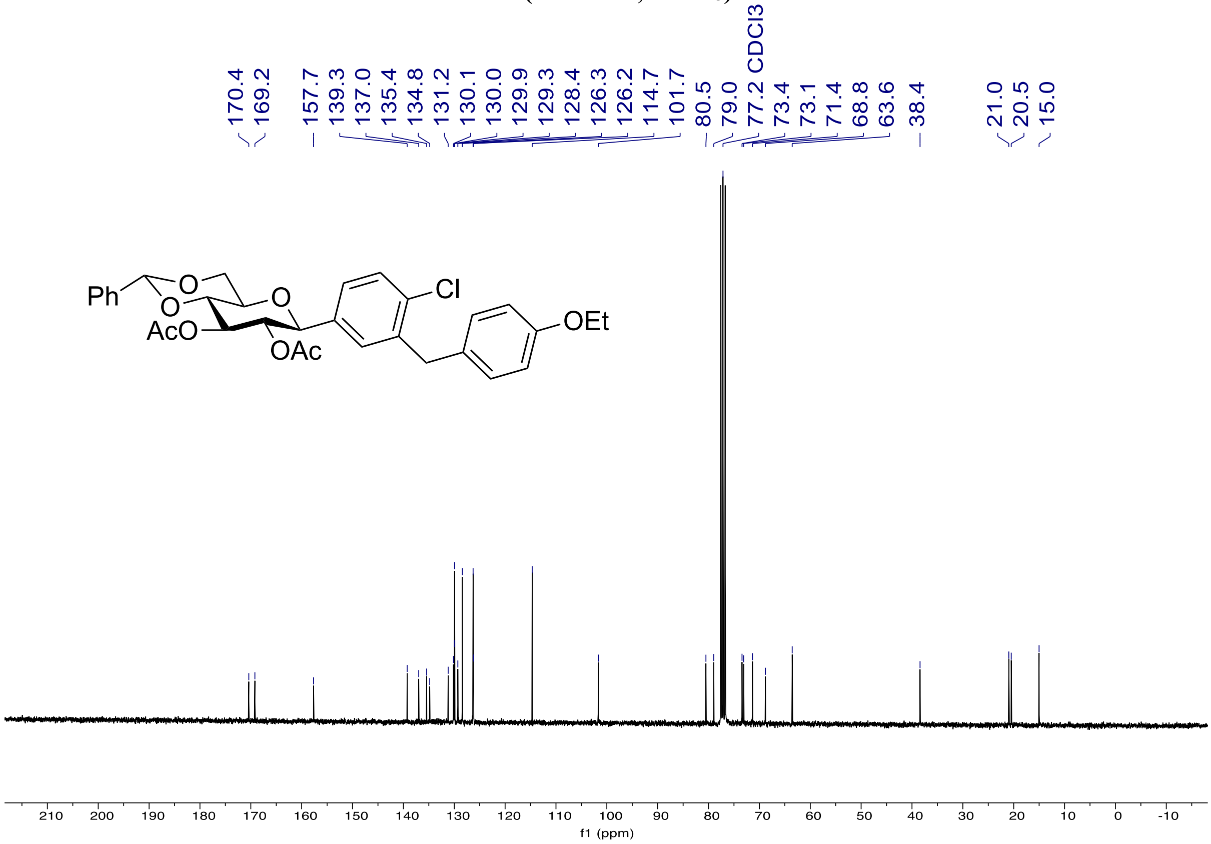

^{13}C NMR (75 MHz, CDCl$_3$) of 3x

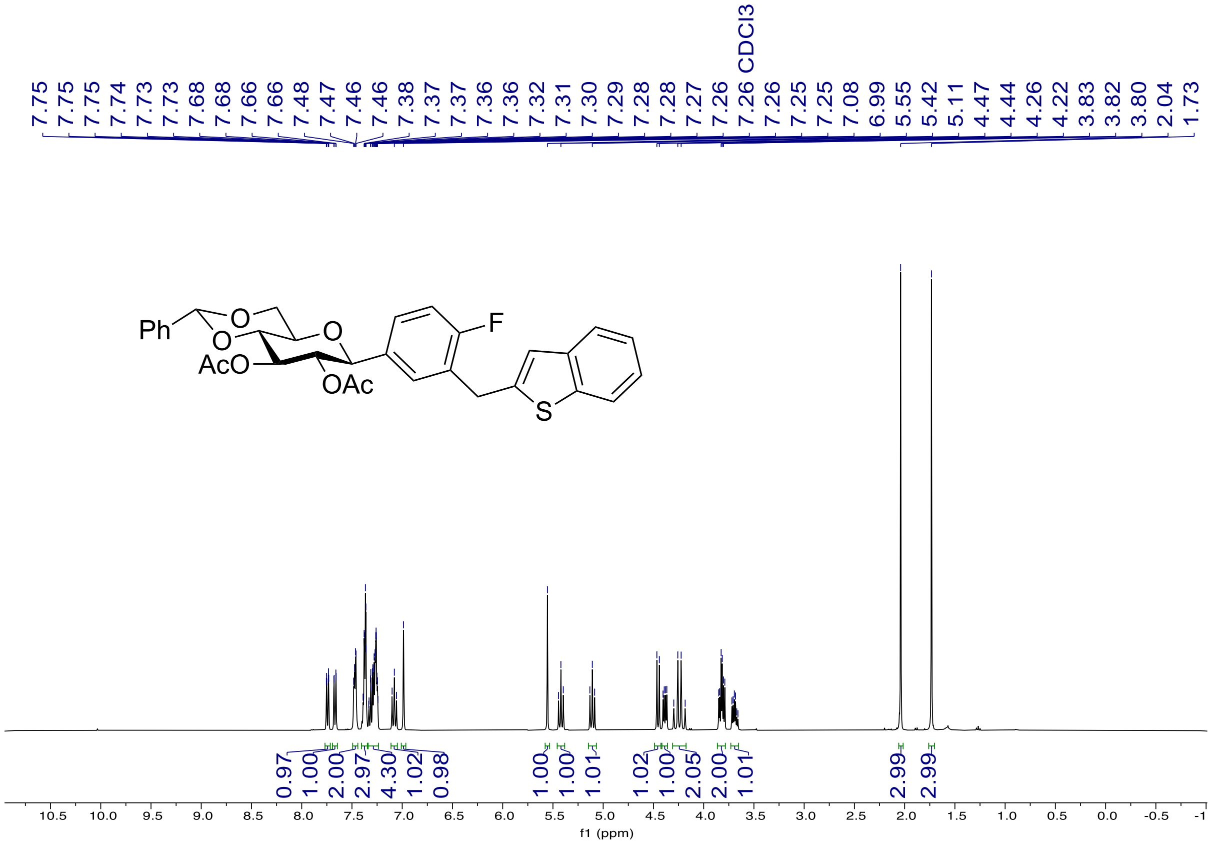

^{1}H NMR (400 MHz, $CDCl_3$) of 3y

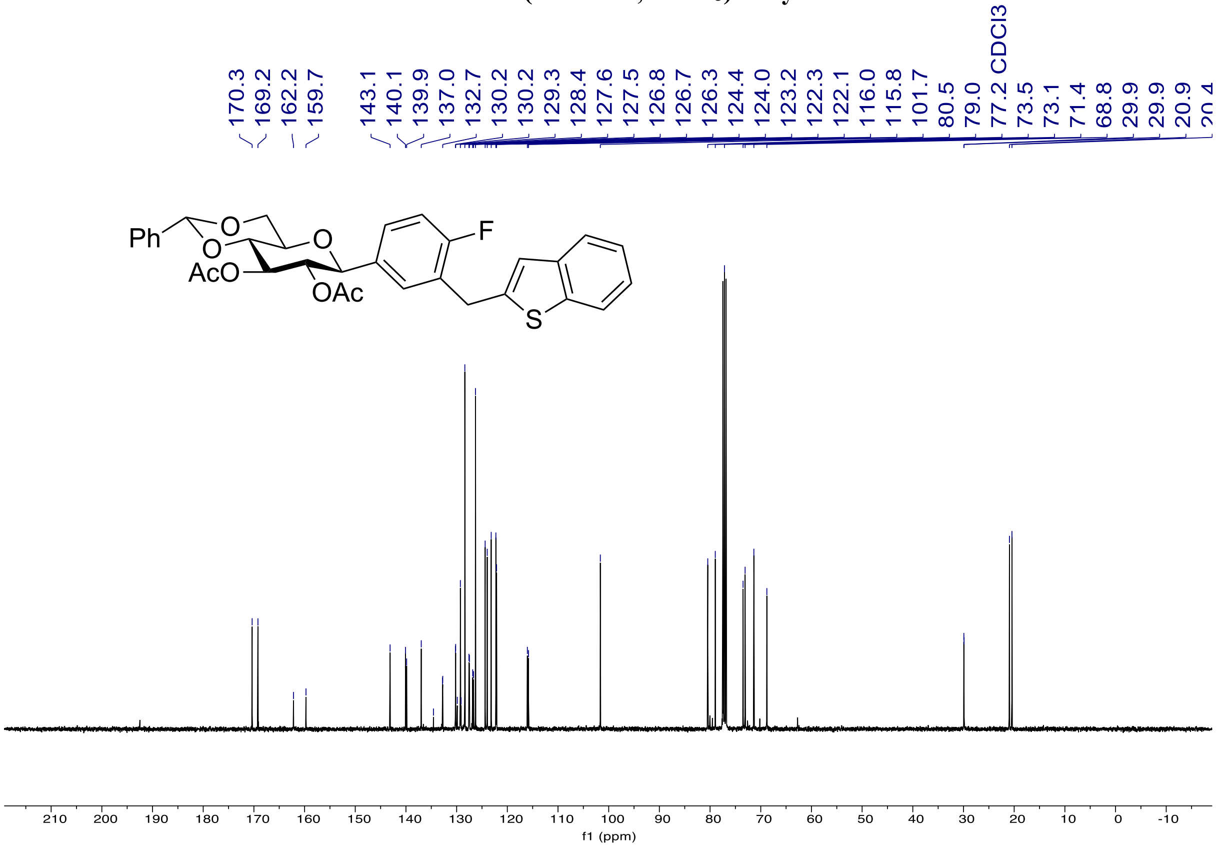

^{13}C NMR (101 MHz, $CDCl_3$) of 3y

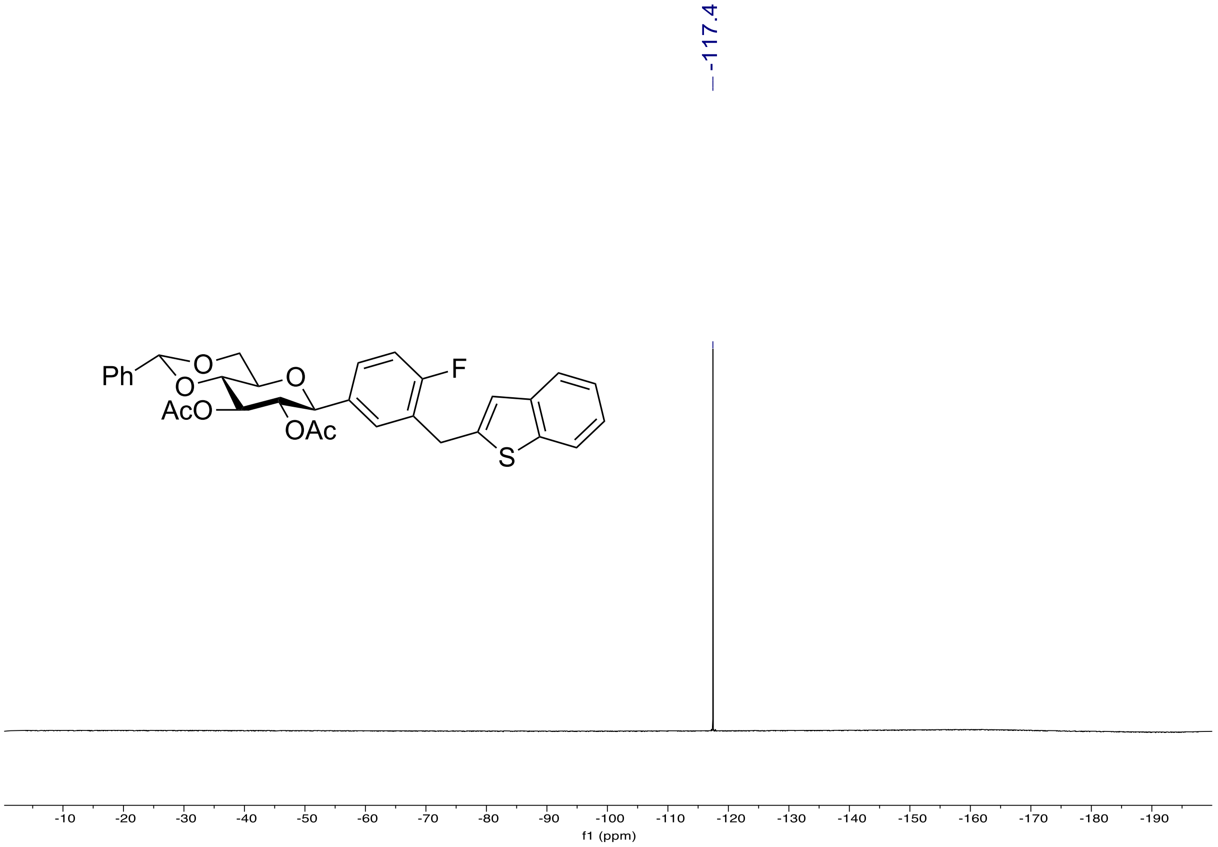

^{19}F NMR (376 MHz, CDCl$_3$) of 3y

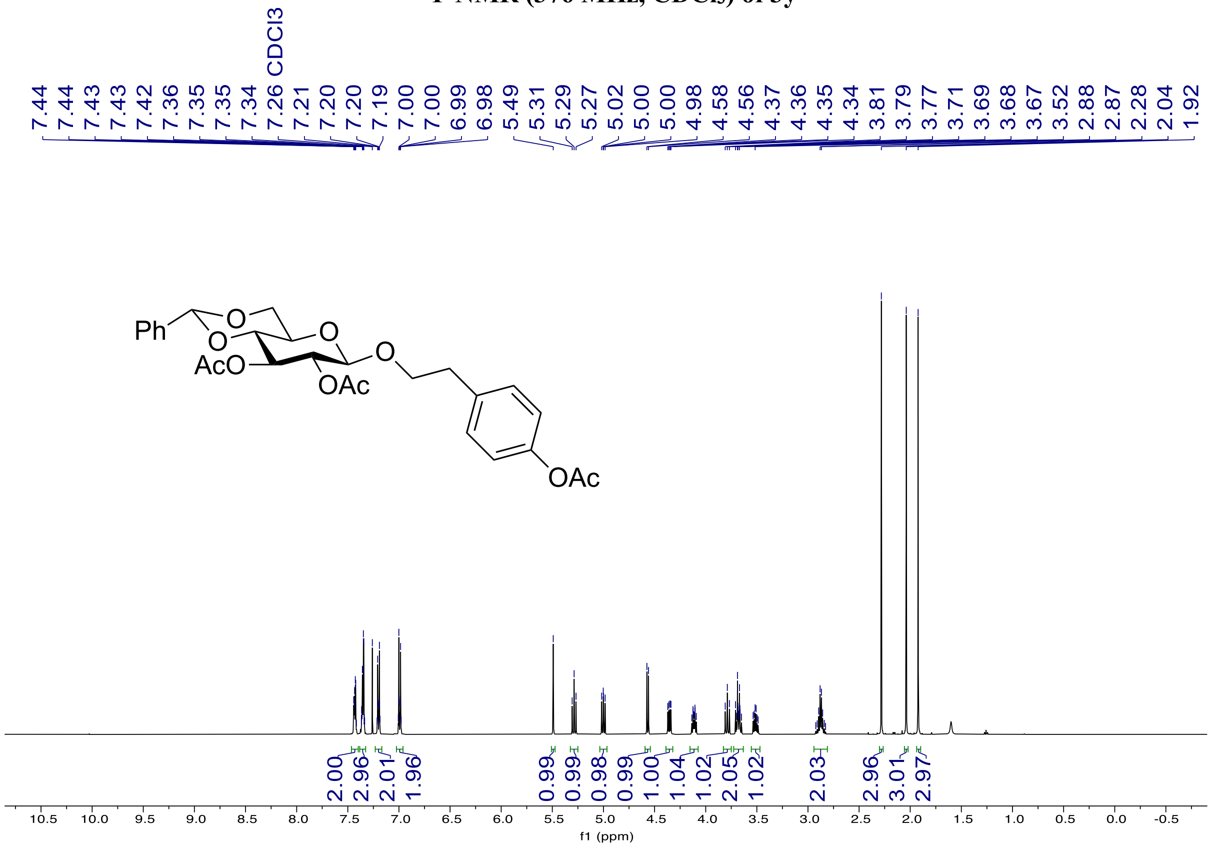

^{1}H NMR (500 MHz, CDCl$_3$) of 3za

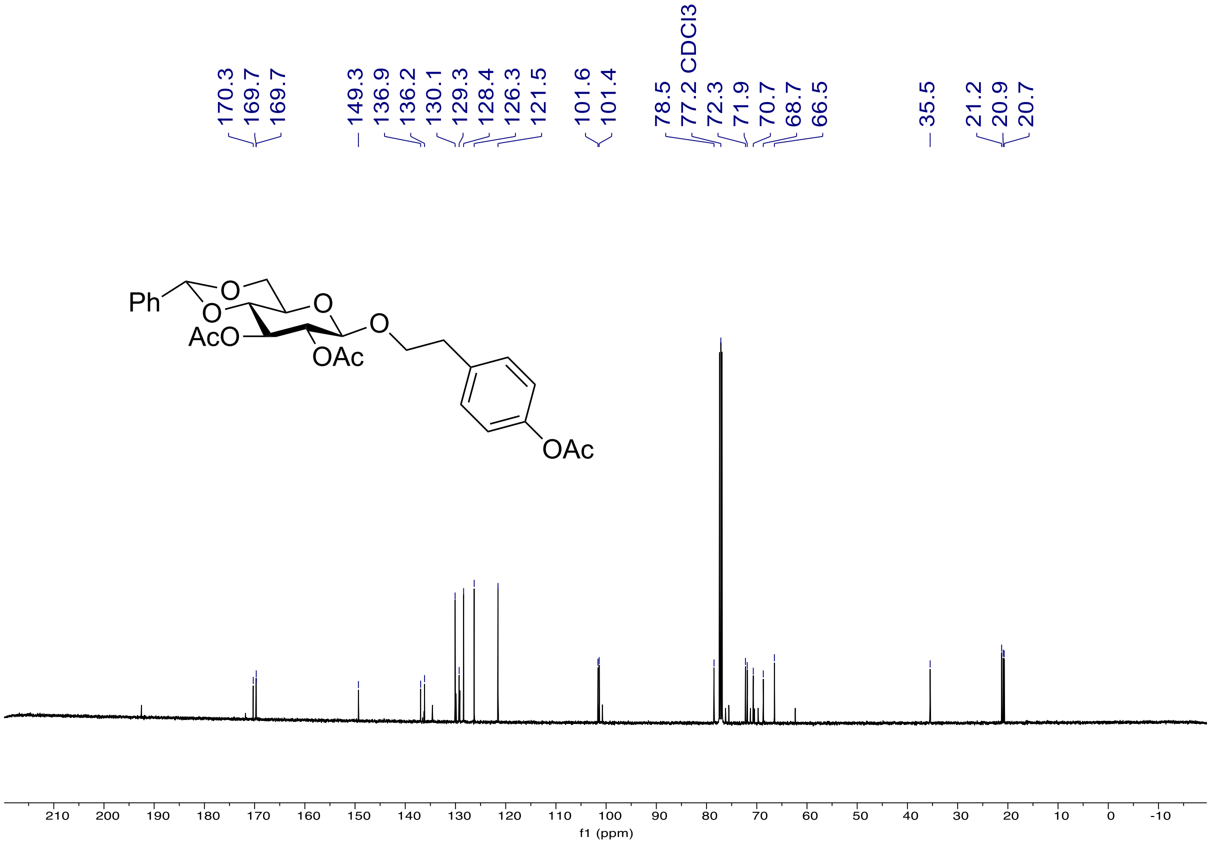

^{13}C NMR (126 MHz, CDCl₃) of 3za

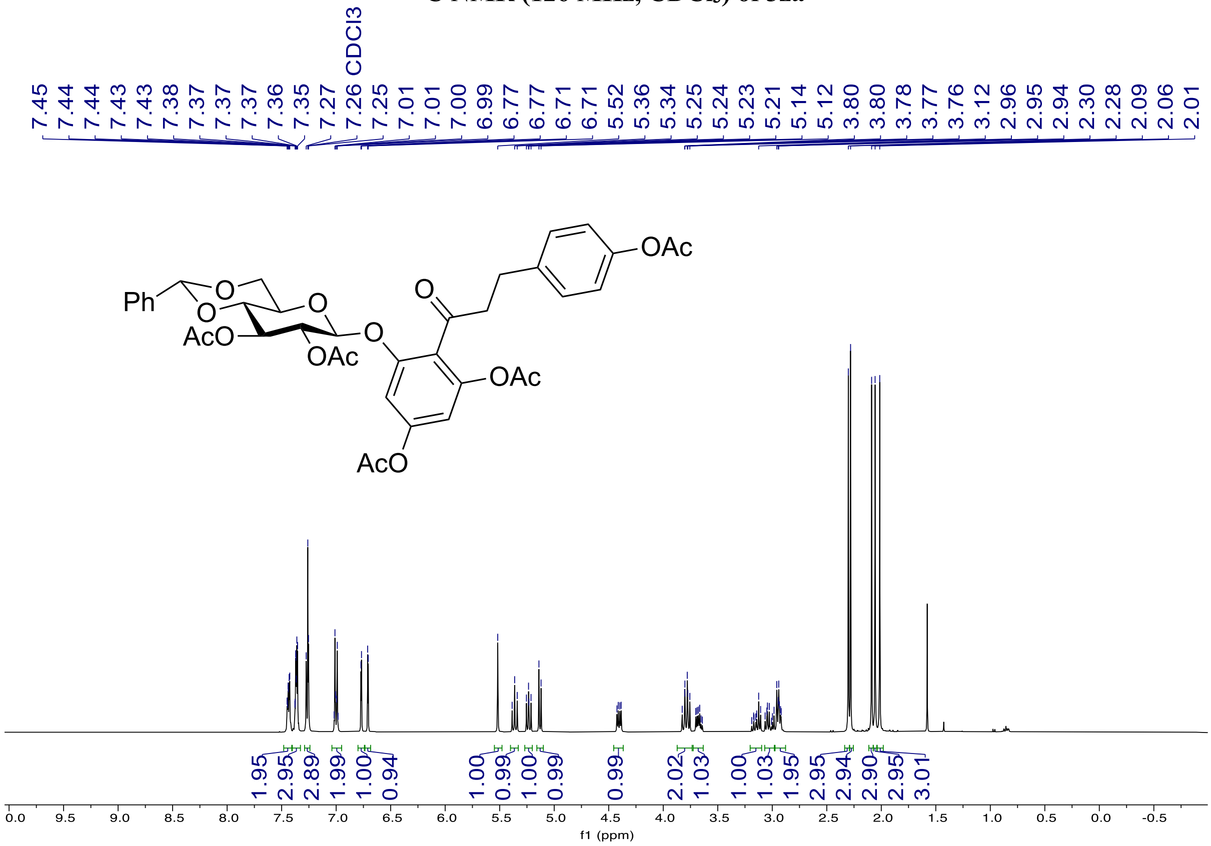

^{1}H NMR (400 MHz, CDCl₃) of 3zb

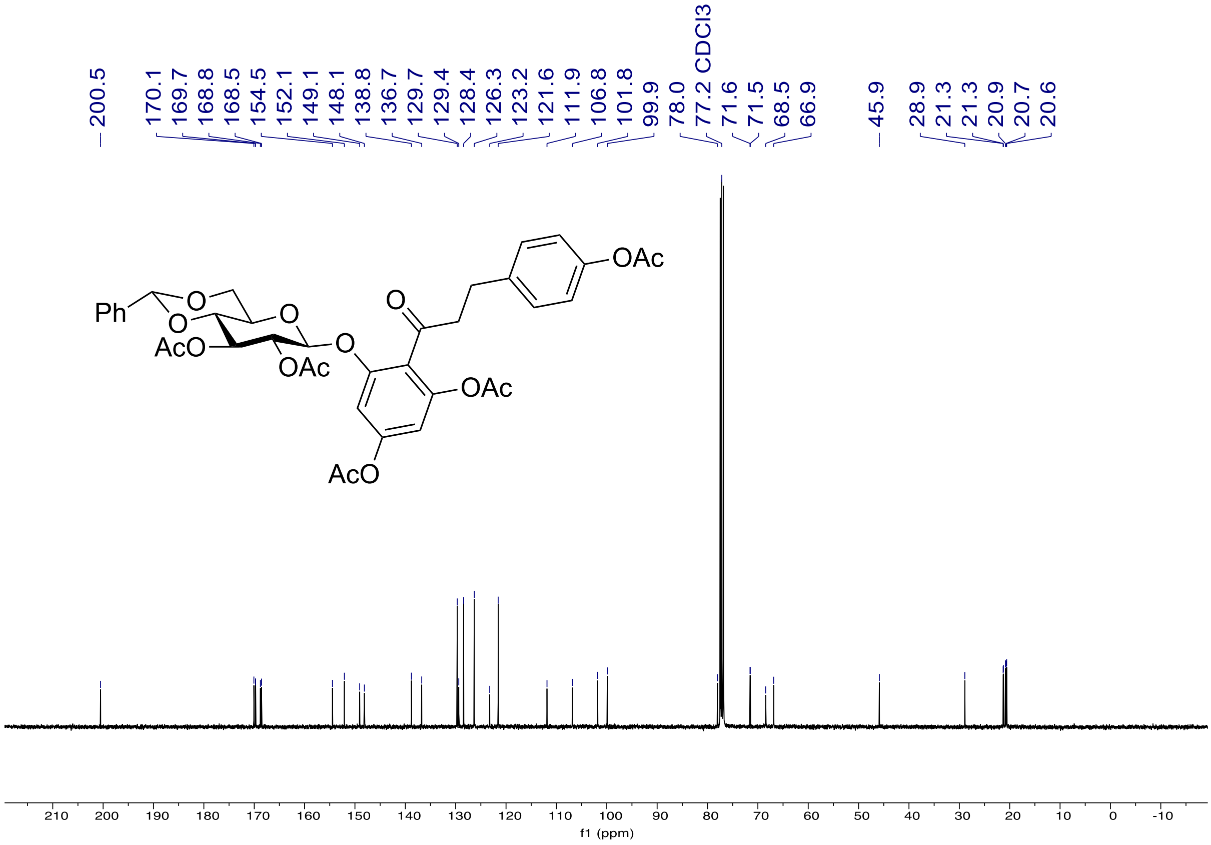

^{13}C NMR (101 MHz, $CDCl_3$) of 3zb

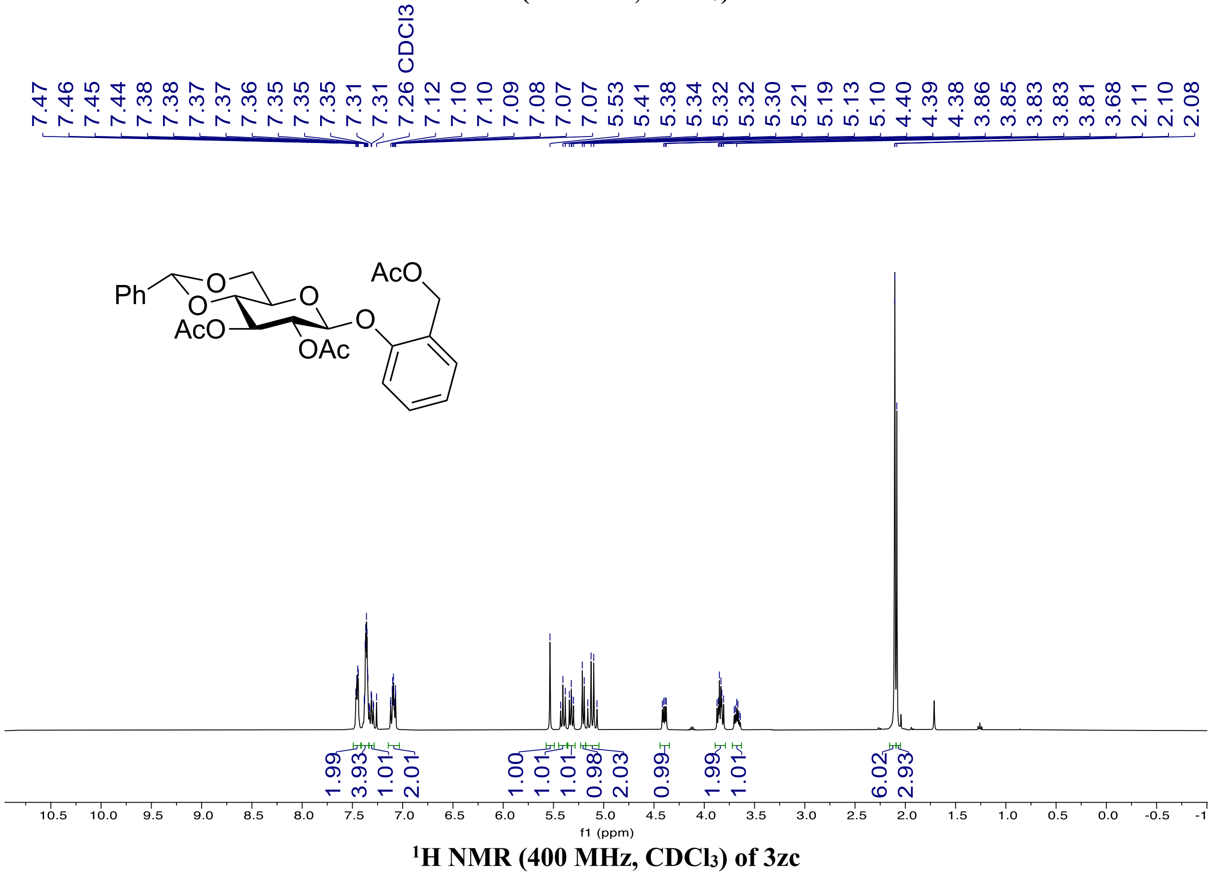

^{1}H NMR (400 MHz, $CDCl_3$) of 3zc

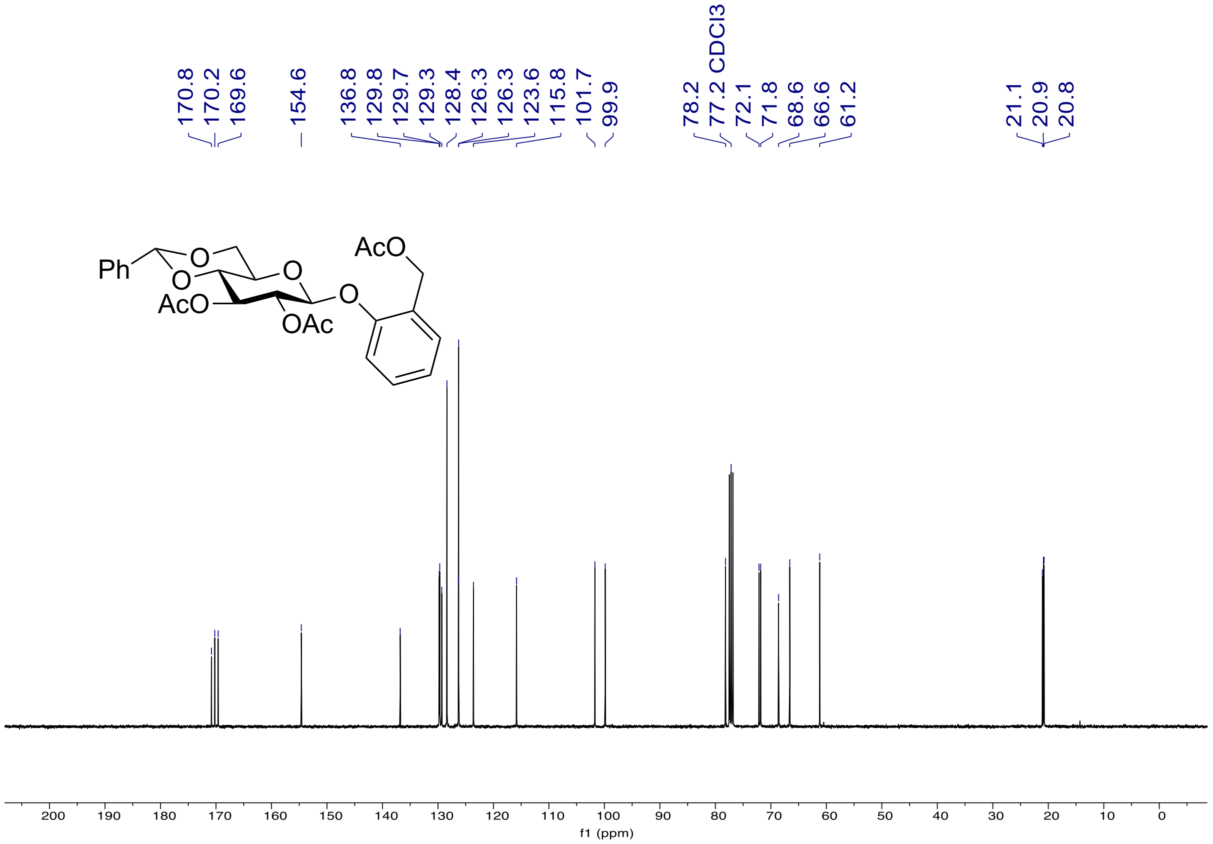

^{13}C NMR (101 MHz, CDCl$_3$) of 3zc

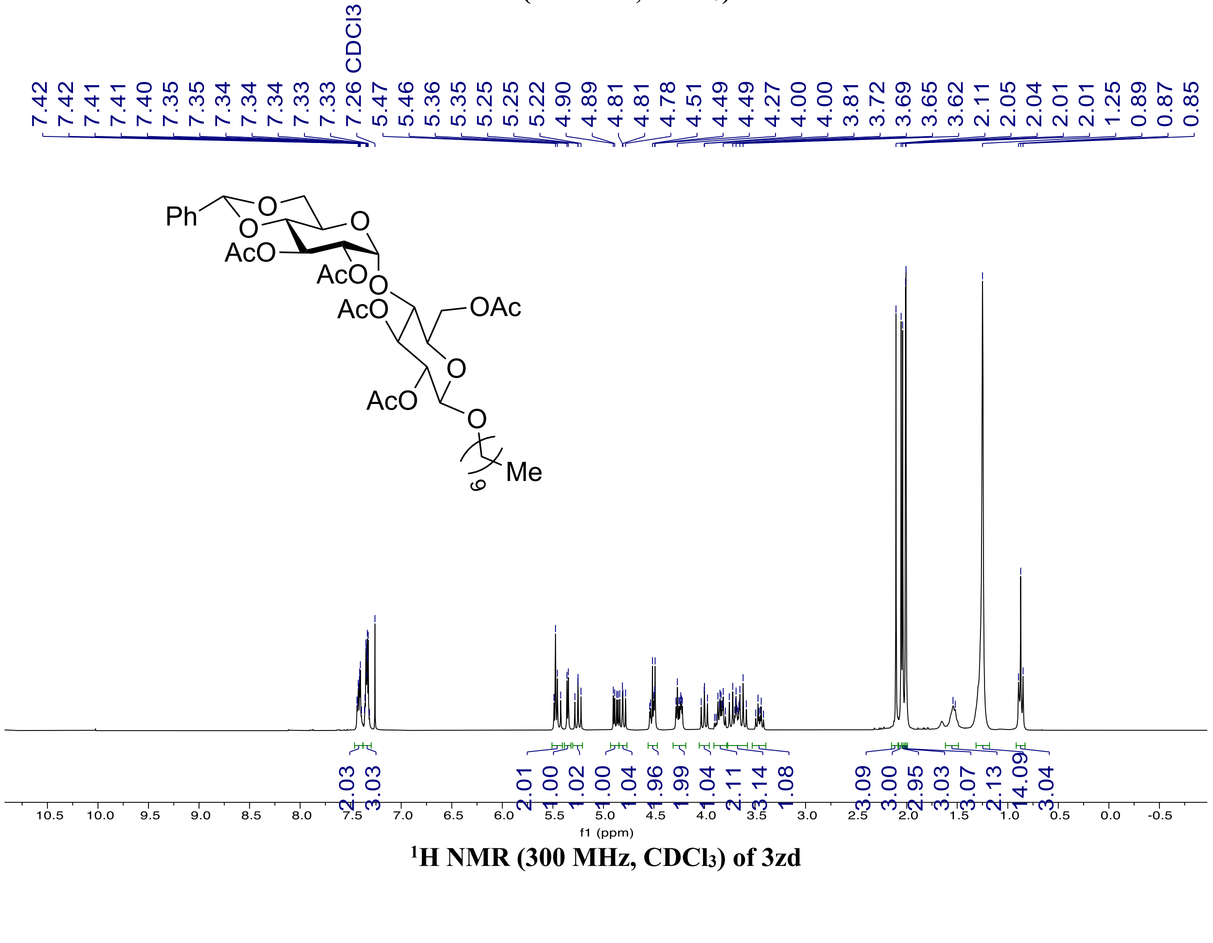

^{1}H NMR (300 MHz, CDCl$_3$) of 3zd

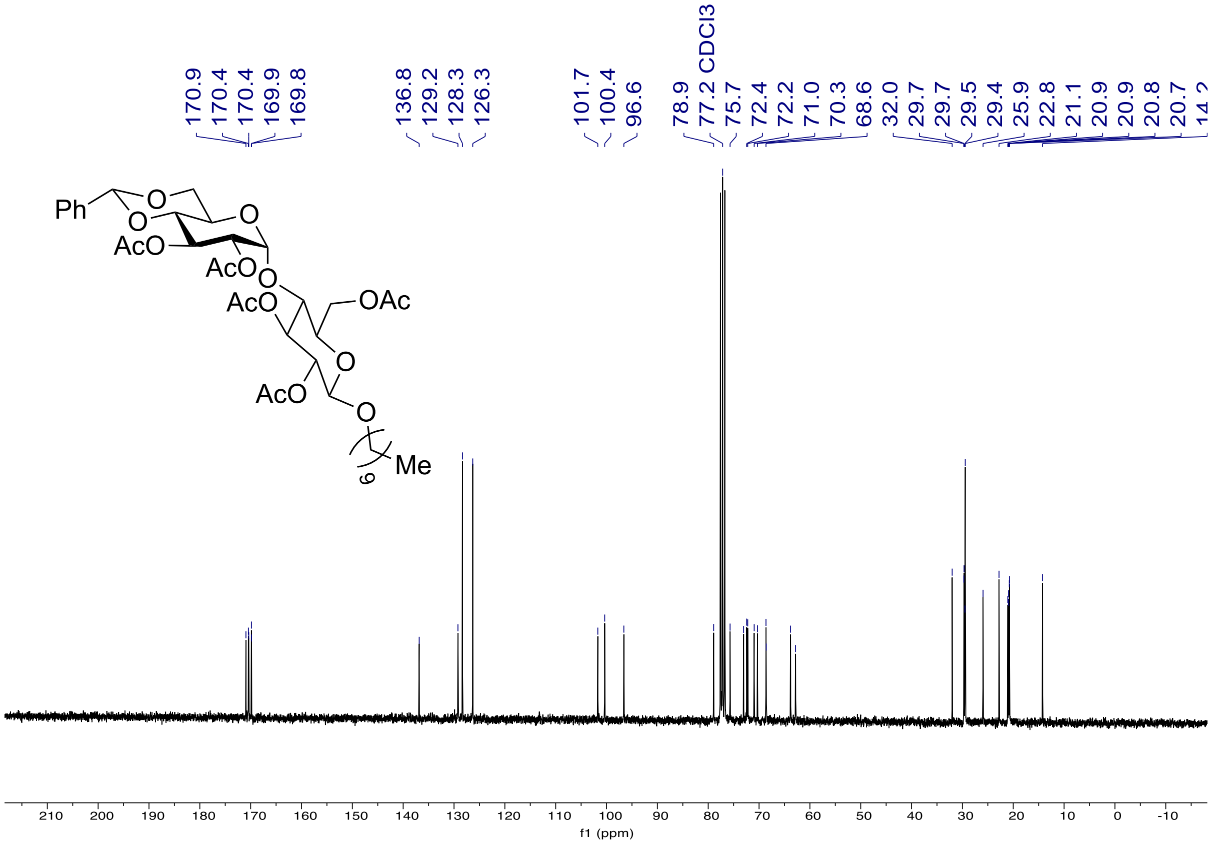

^{13}**C NMR (75 MHz, CDCl$_3$) of 3zd**

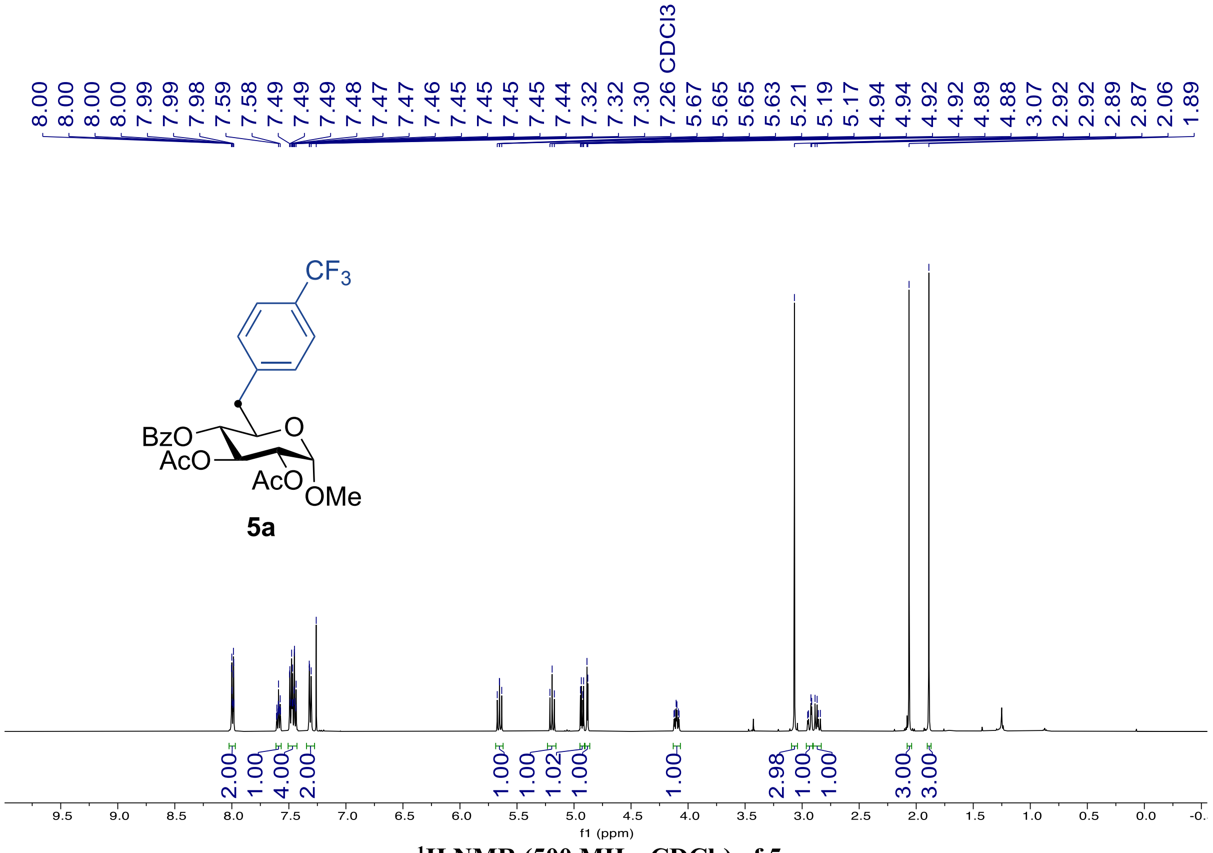

^{1}**H NMR (500 MHz, CDCl$_3$) of 5a**

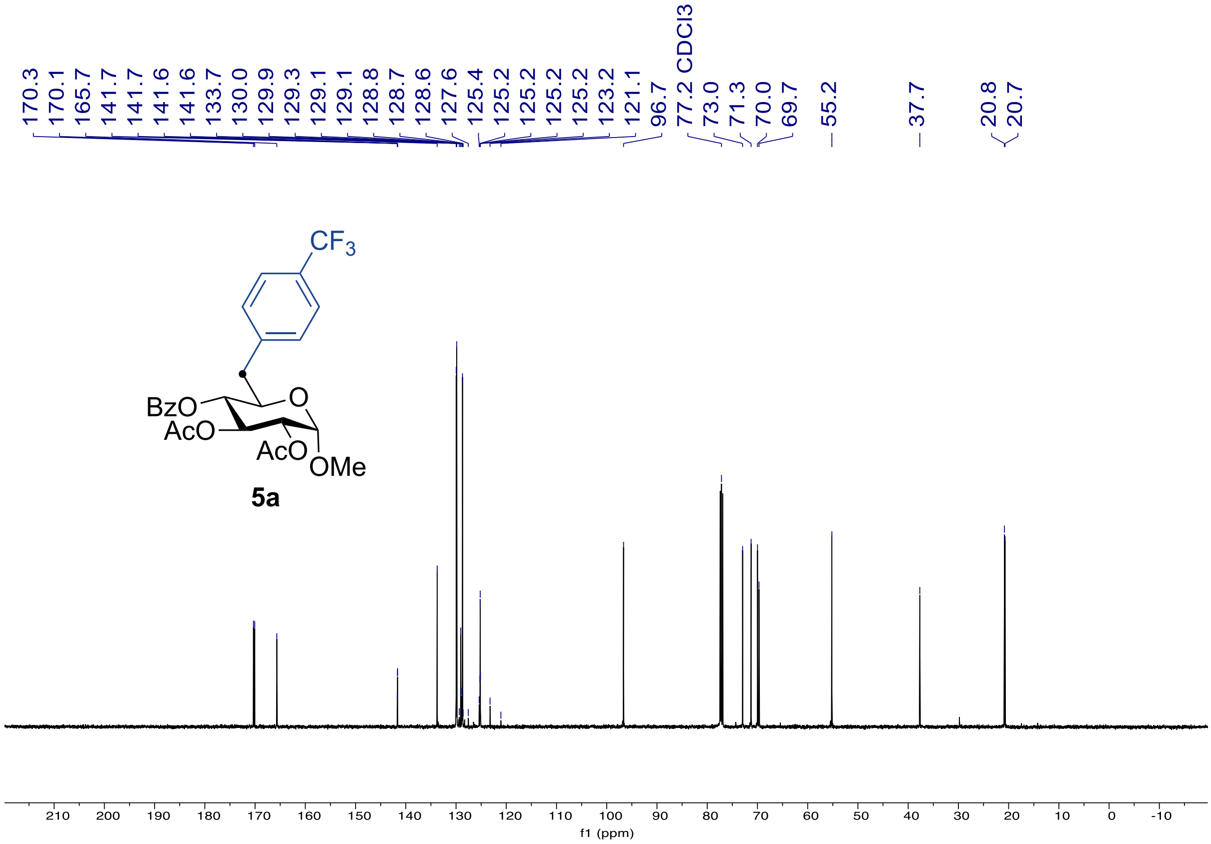

^{13}C NMR (126 MHz, $CDCl_3$) of 5a

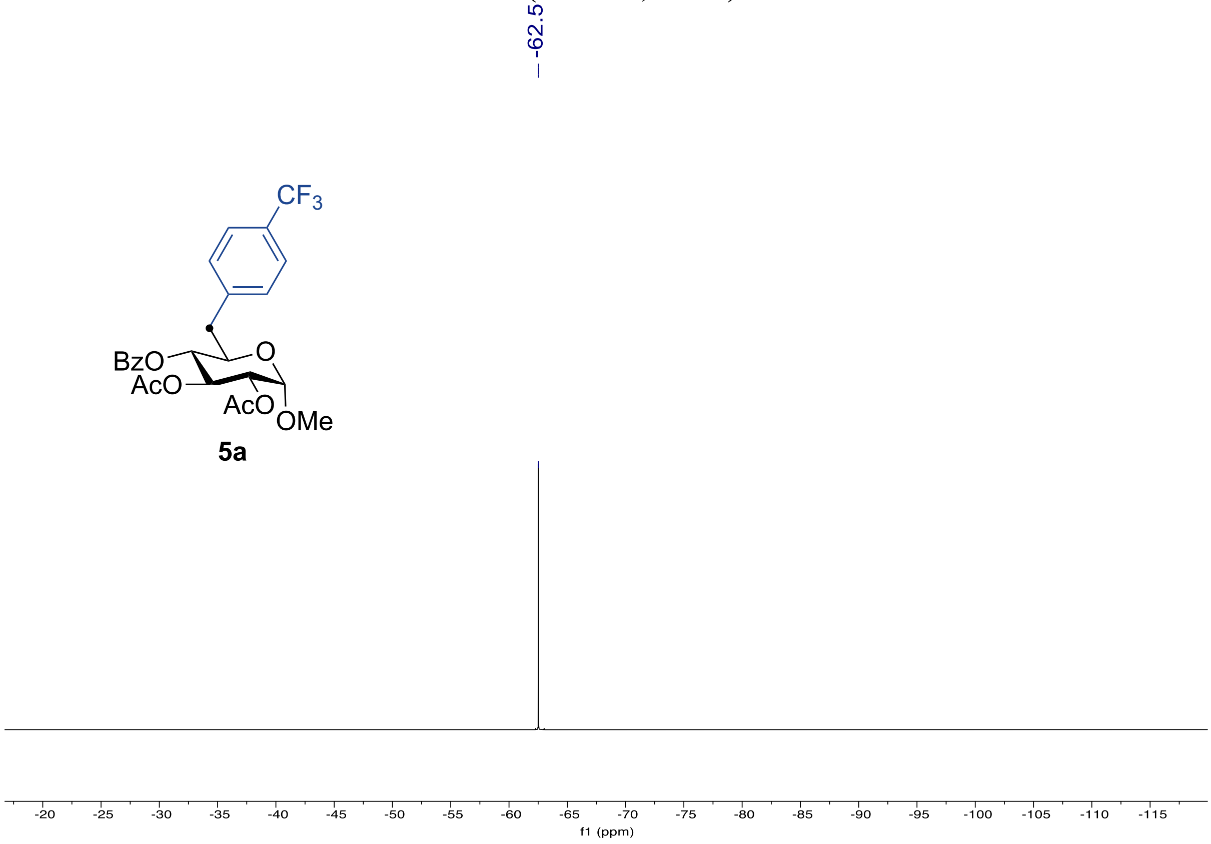

^{19}F NMR (376 MHz, $CDCl_3$) of 5a

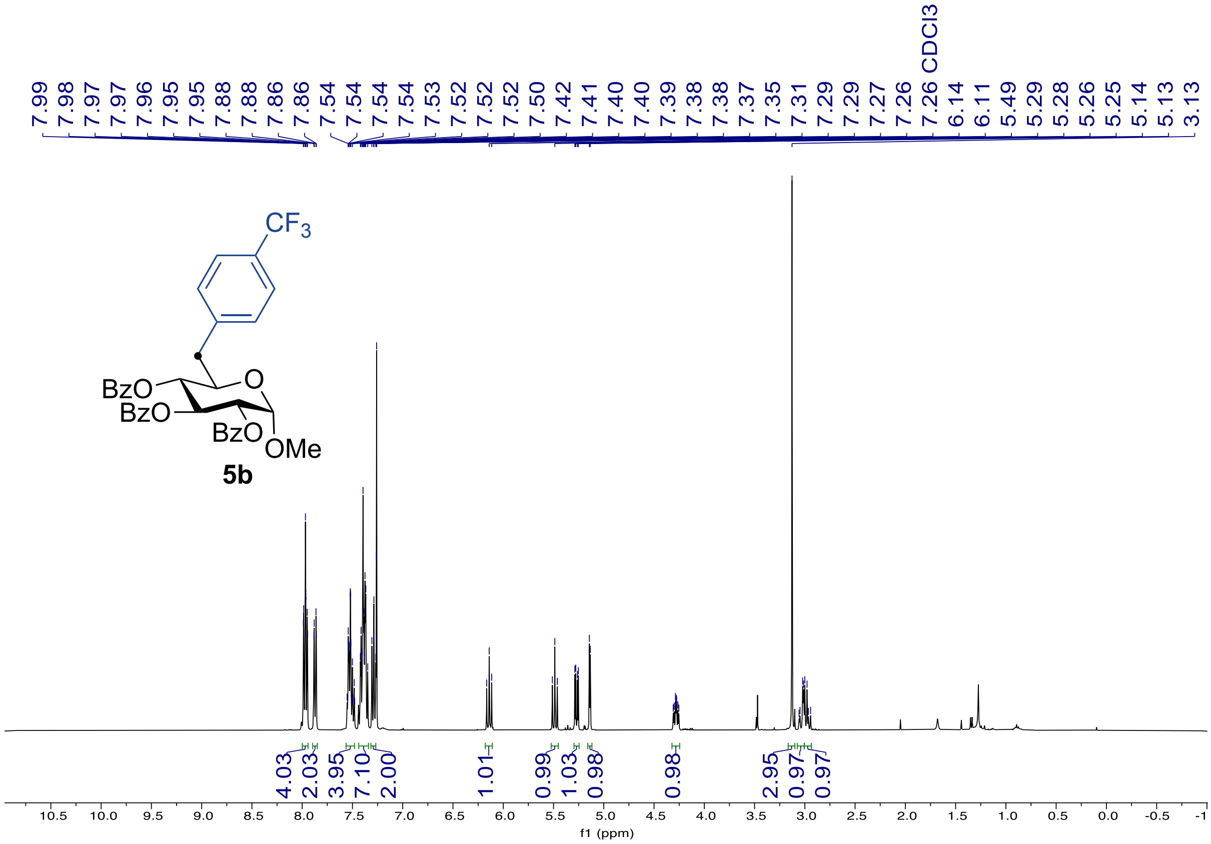

^{1}H NMR (400 MHz, CDCl3) of 5b

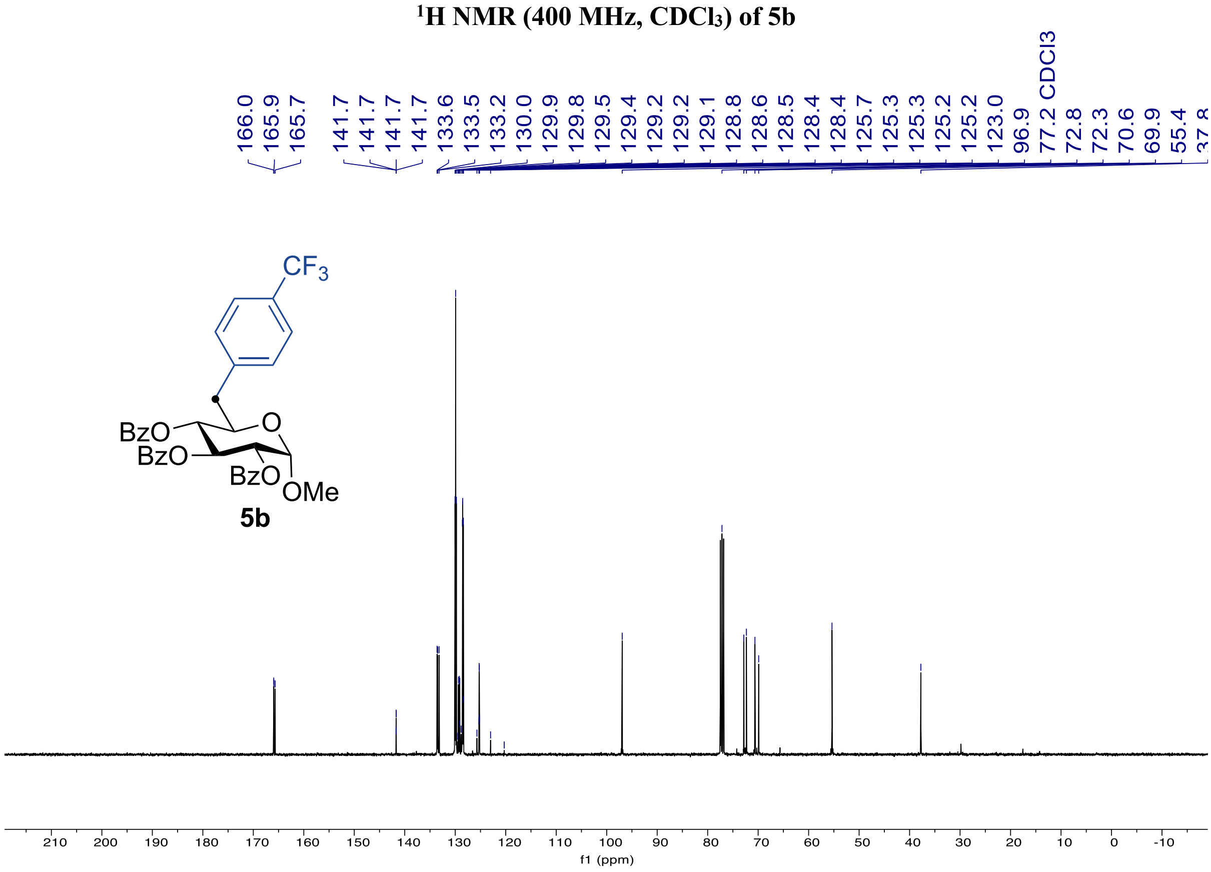

^{13}C NMR (101 MHz, CDCl3) of 5b

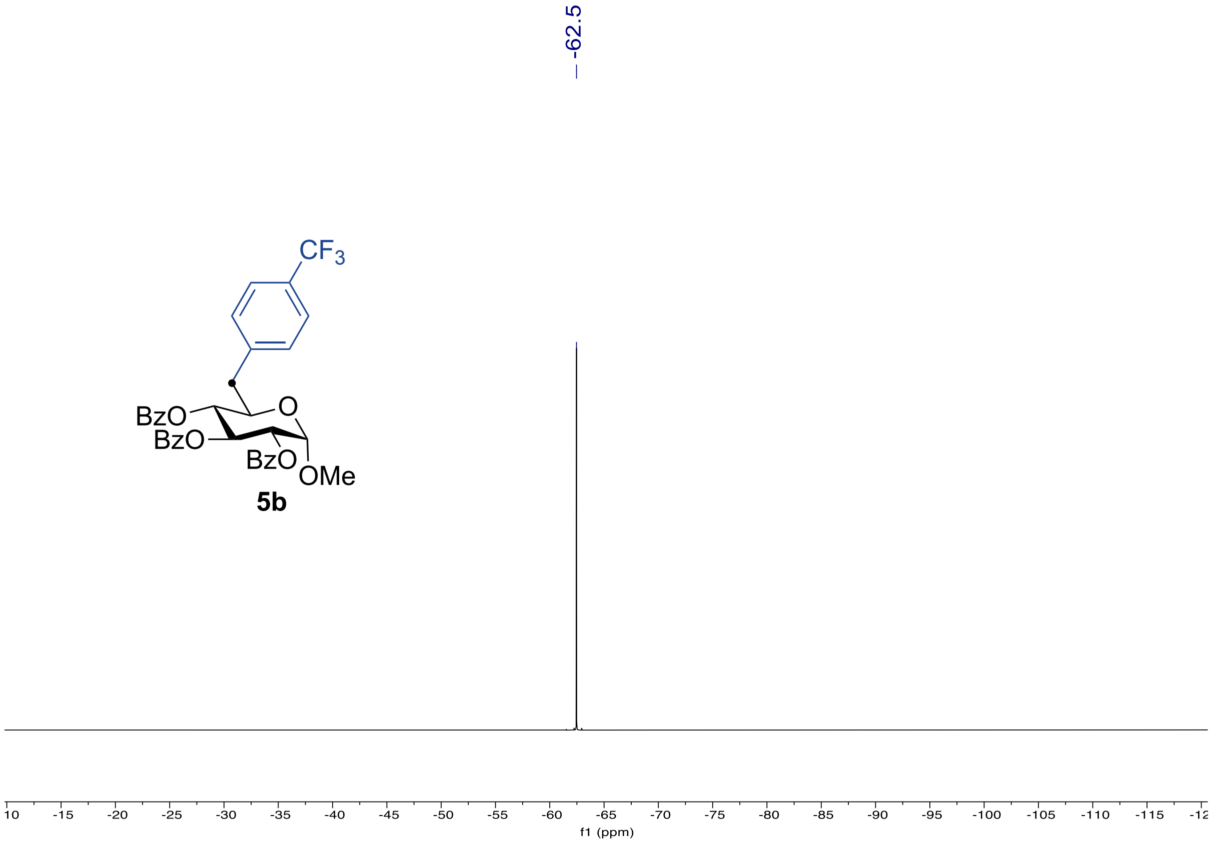

^{19}F NMR (376 MHz, CDCl$_3$) of 5b

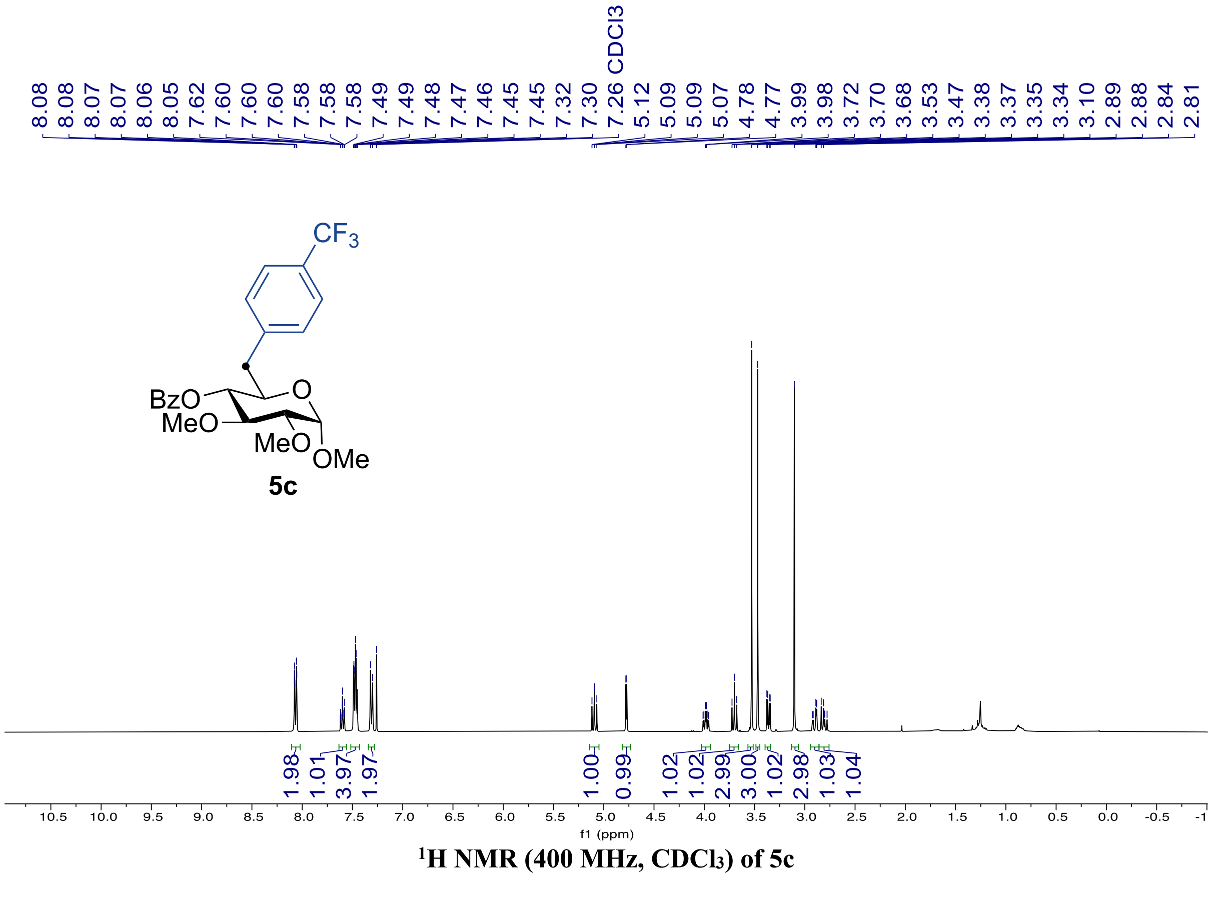

^{1}H NMR (400 MHz, CDCl$_3$) of 5c

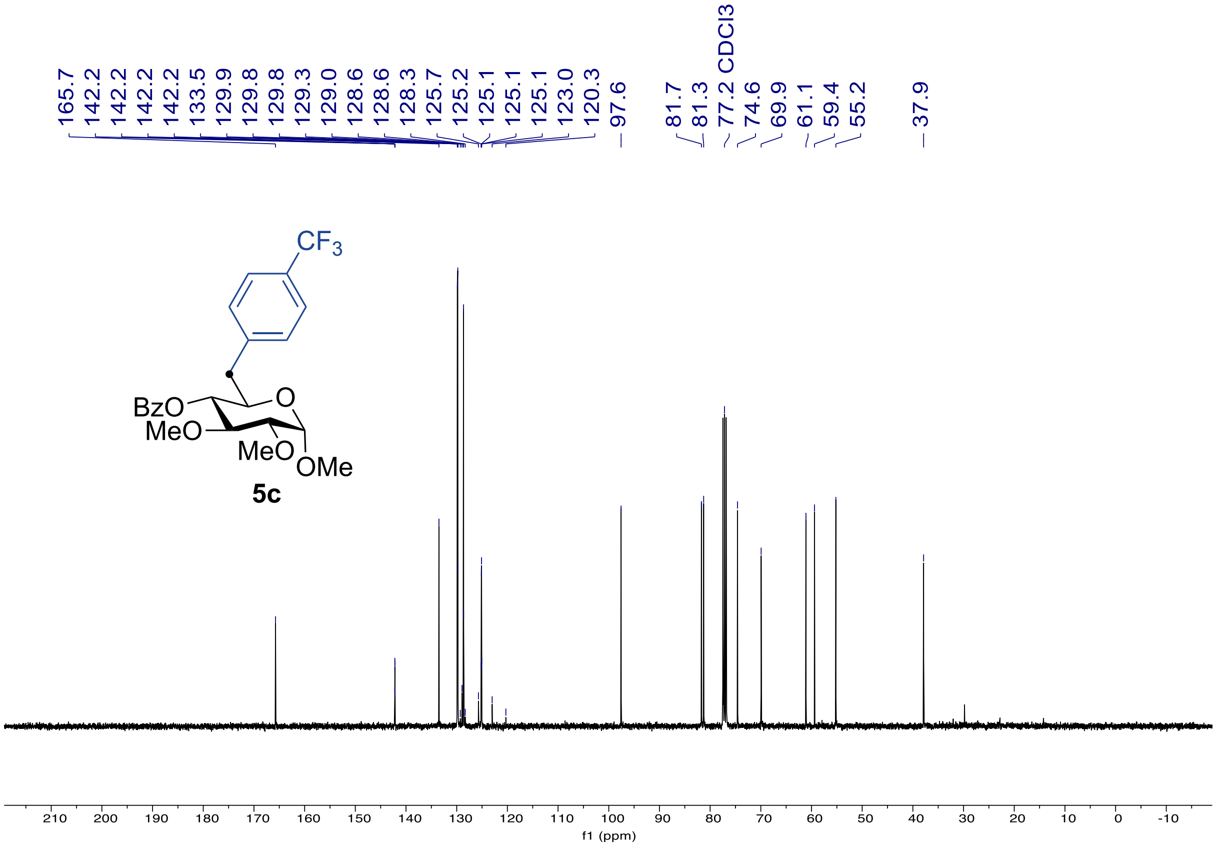

^{13}C NMR (101 MHz, CDCl$_3$) of 5c

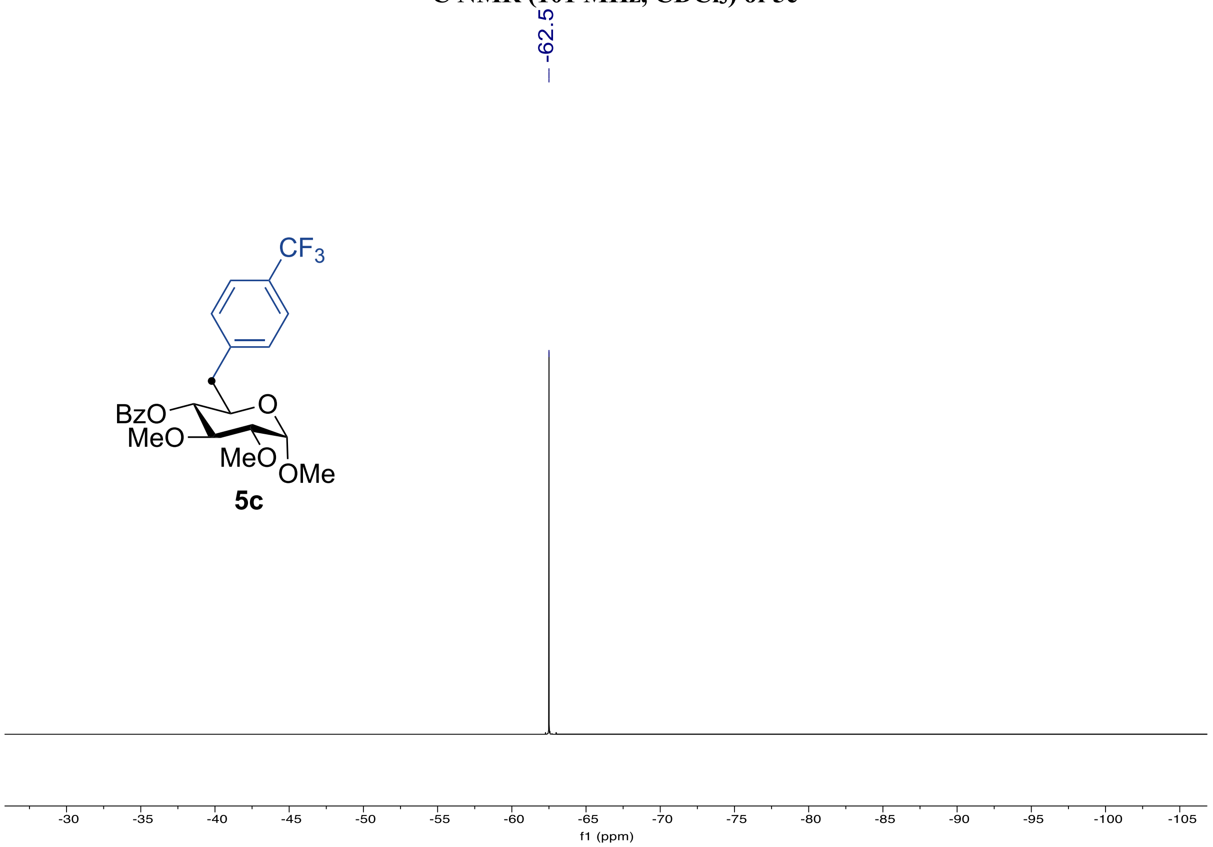

^{19}F NMR (376 MHz, CDCl$_3$) of 5c

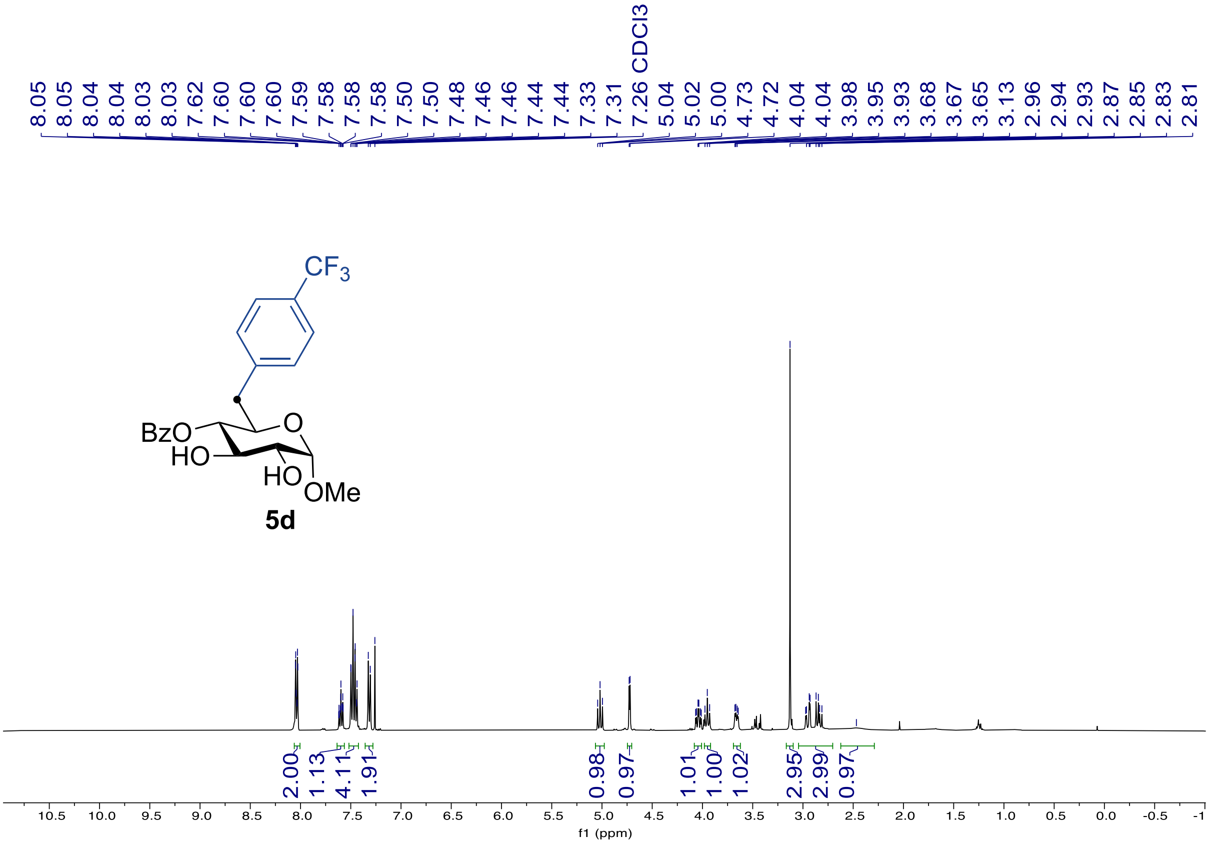

^{1}H NMR (400 MHz, CDCl₃) of 5d

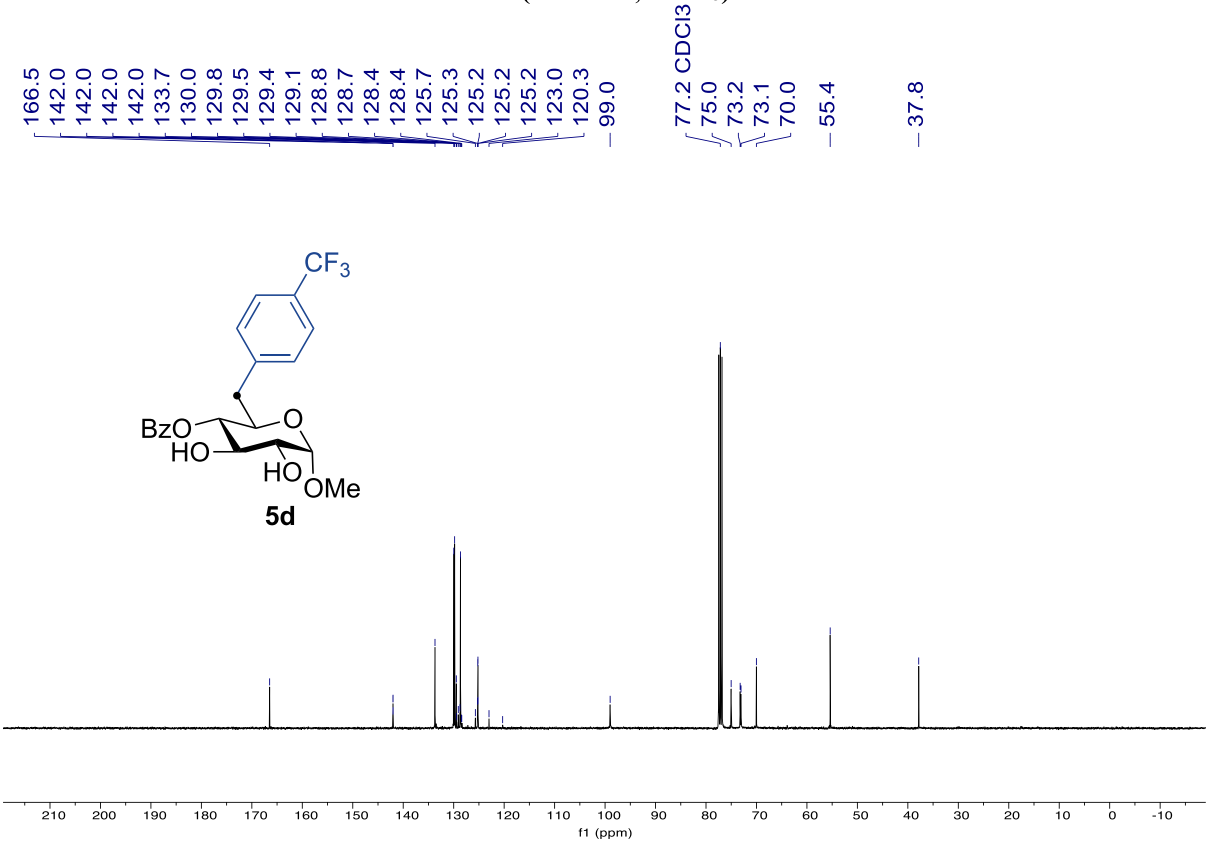

^{13}C NMR (101 MHz, CDCl₃) of 5d

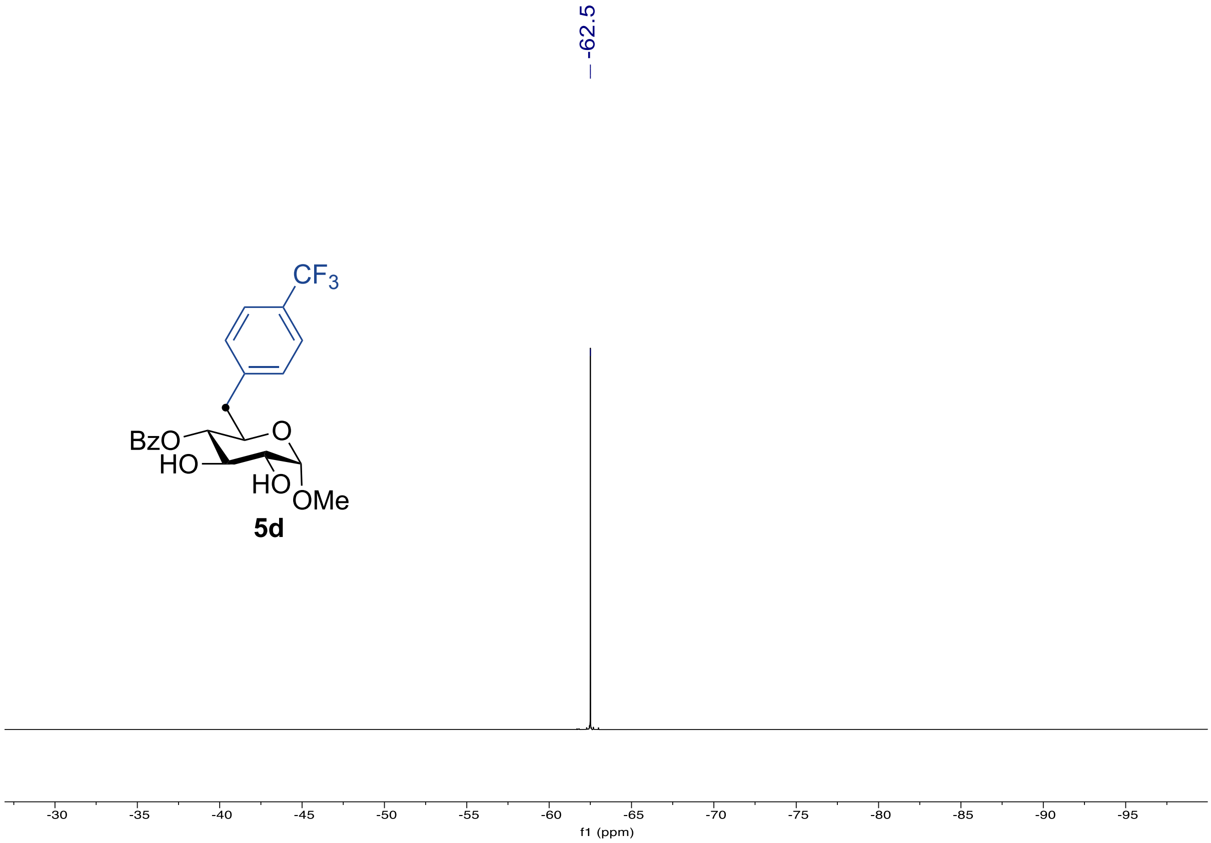

^{19}F NMR (376 MHz, CDCl$_3$) of 5d

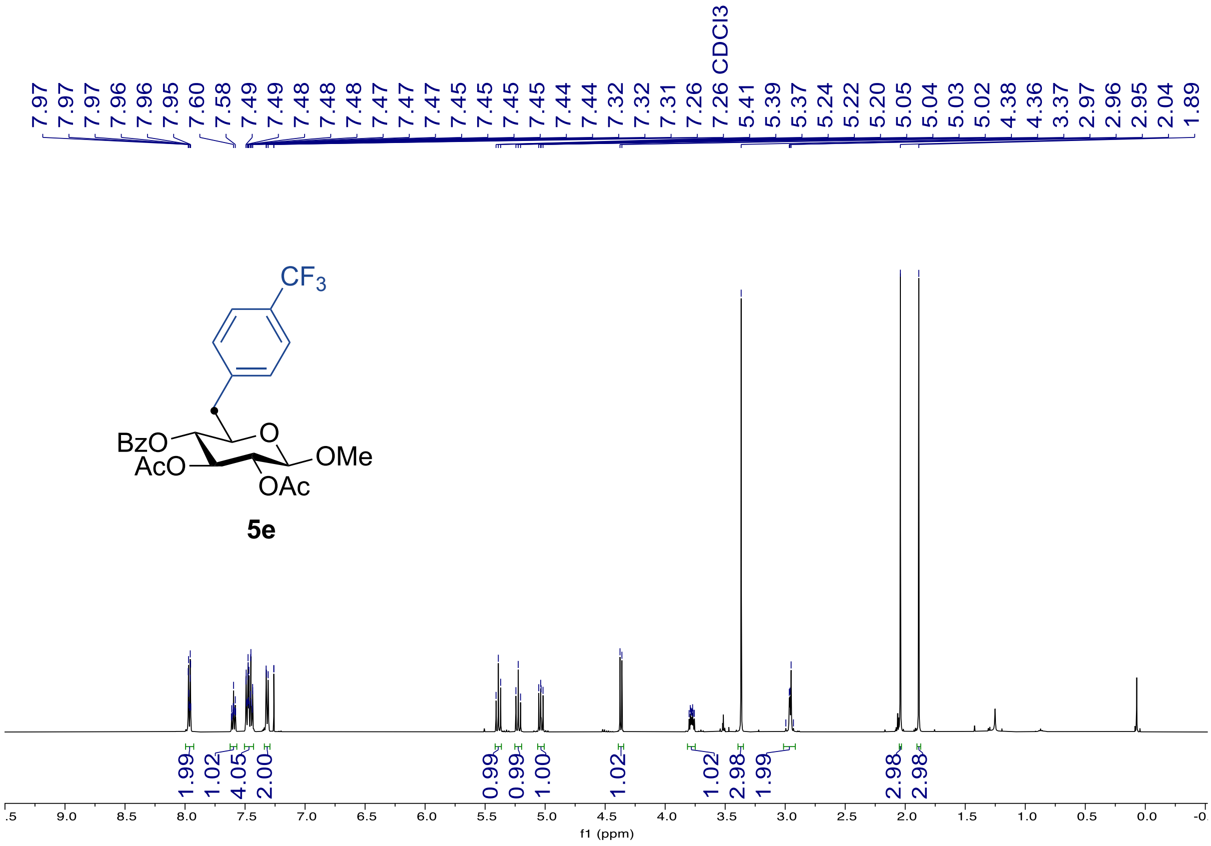

^{1}H NMR (500 MHz, CDCl$_3$) of 5e

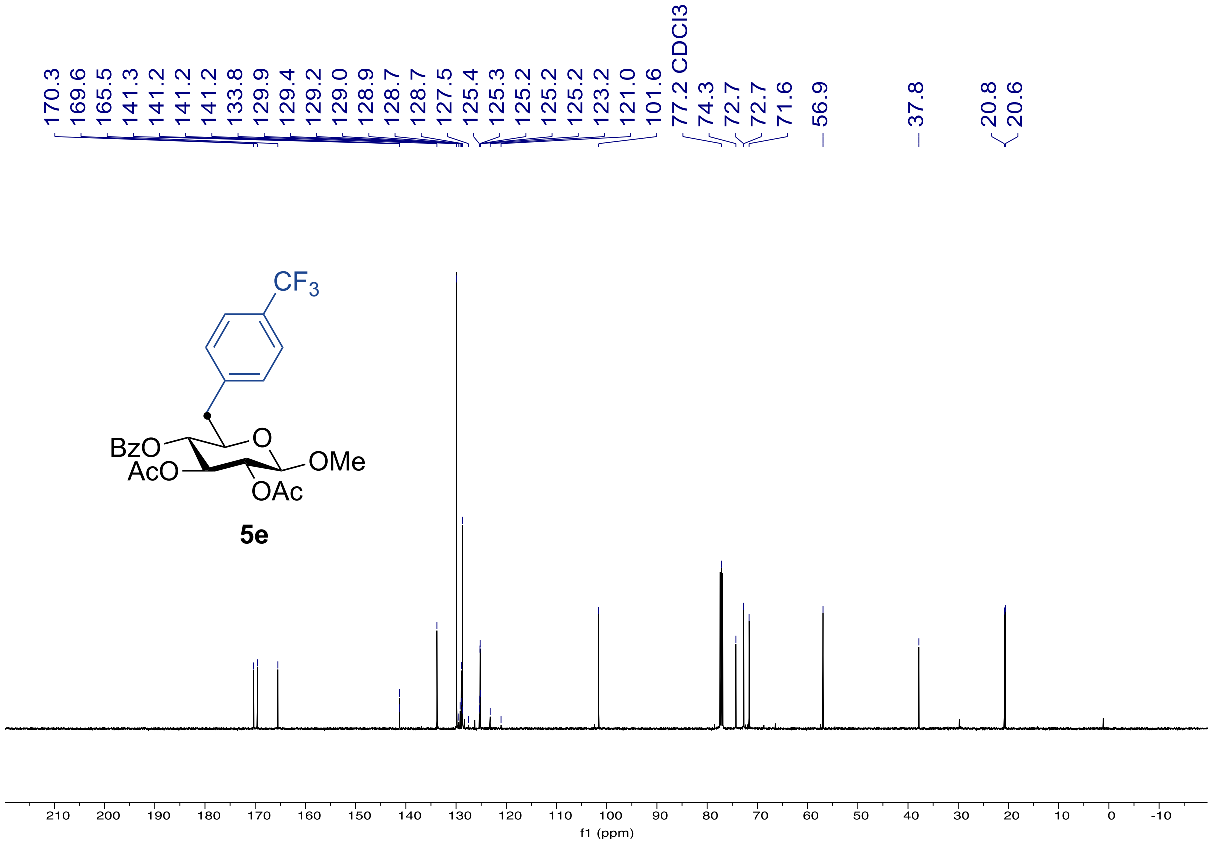

^{13}C NMR (126 MHz, $CDCl_3$) of 5e

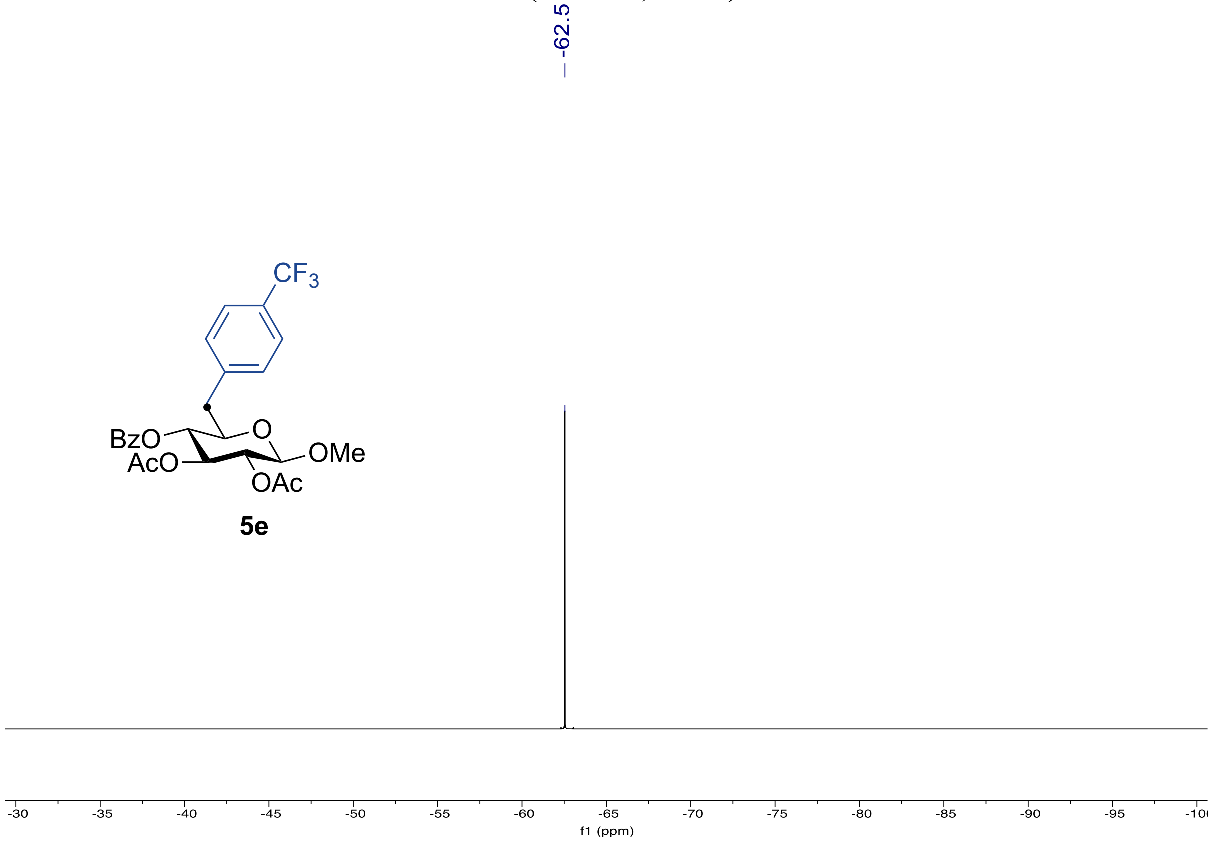

^{19}F NMR (376 MHz, $CDCl_3$) of 5e

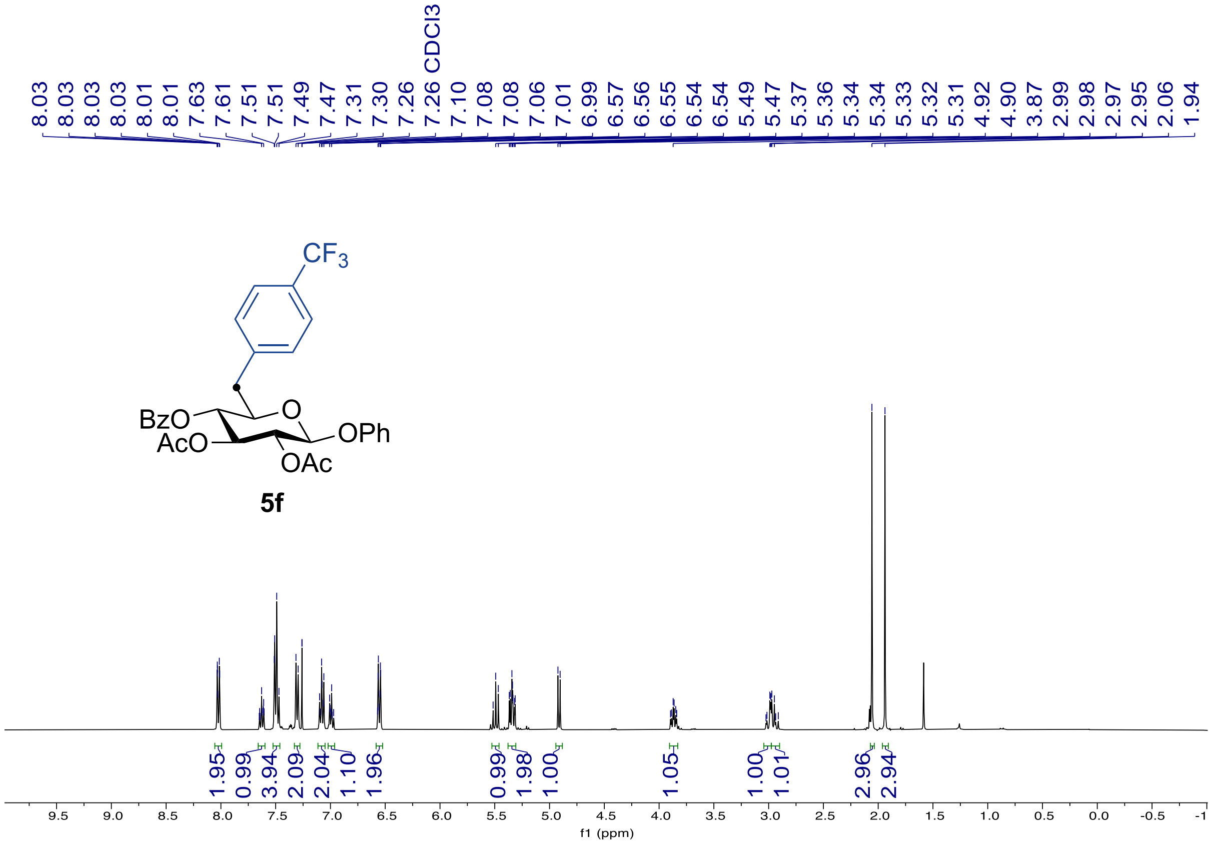

^{1}H NMR (400 MHz, $CDCl_3$) of 5f

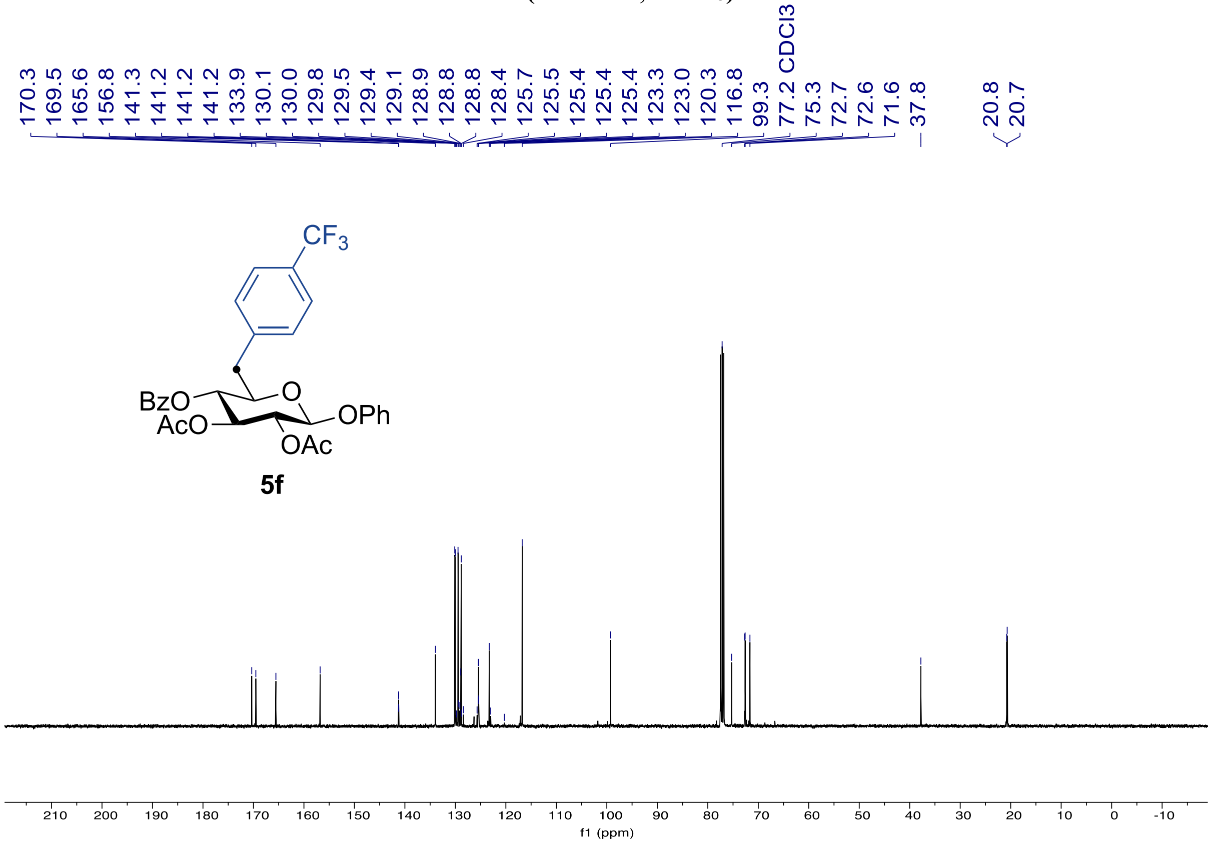

^{13}C NMR (101 MHz, $CDCl_3$) of 5f

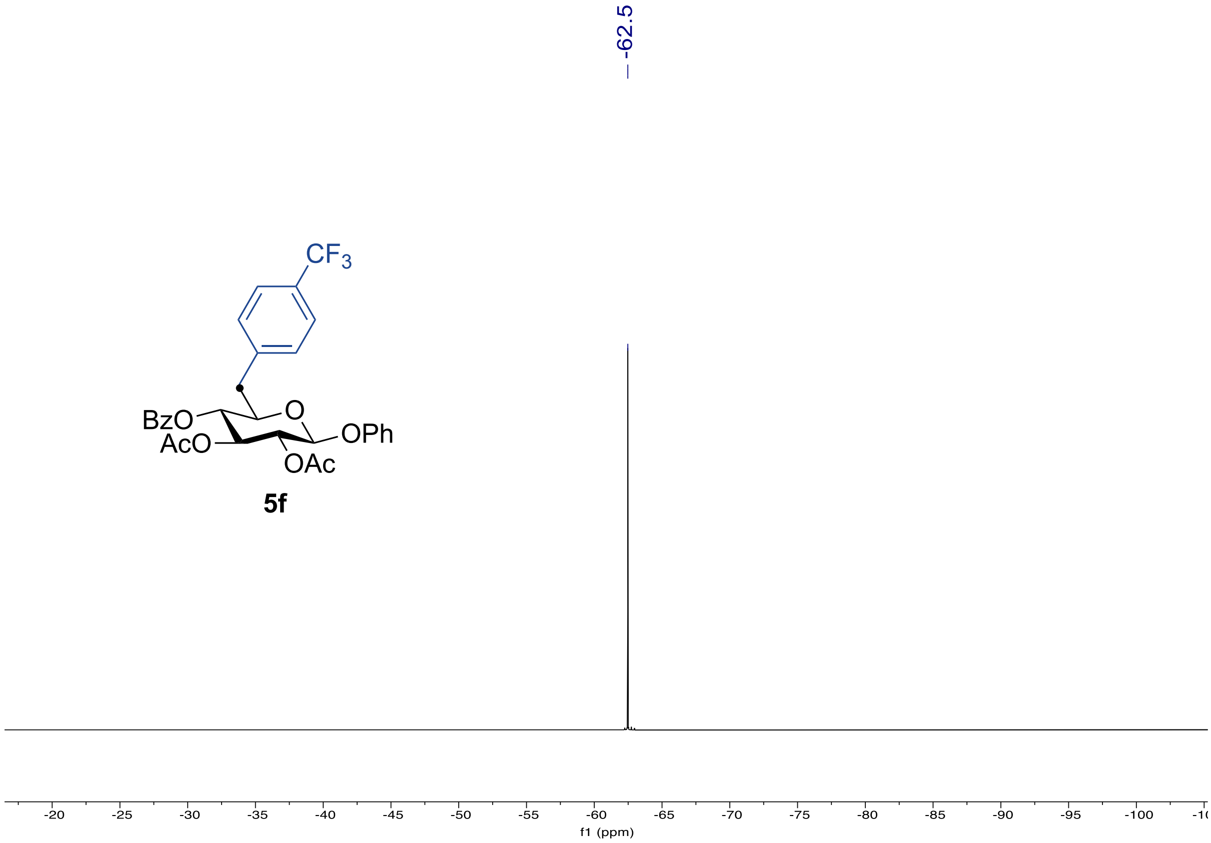

^{19}F NMR (376 MHz, CDCl$_3$) of 5f

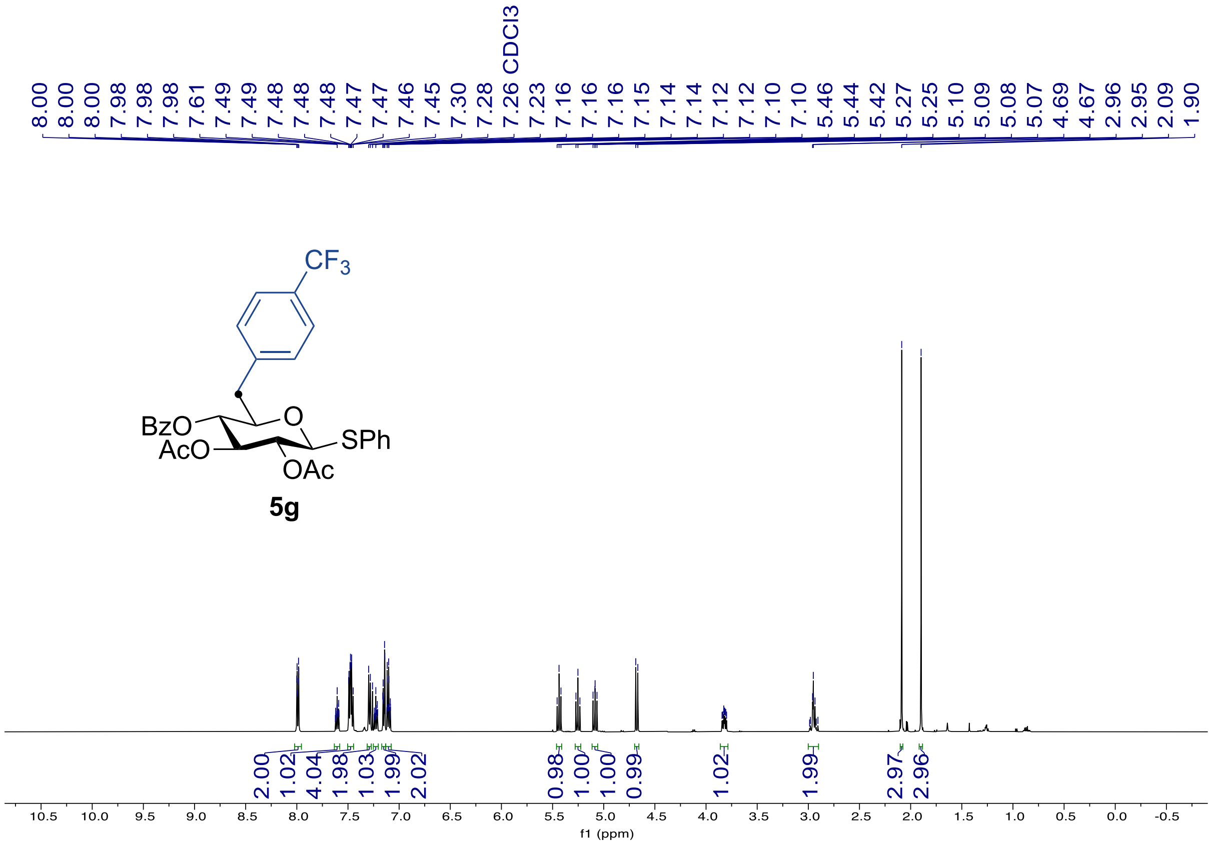

^{1}H NMR (500 MHz, CDCl$_3$) of 5g

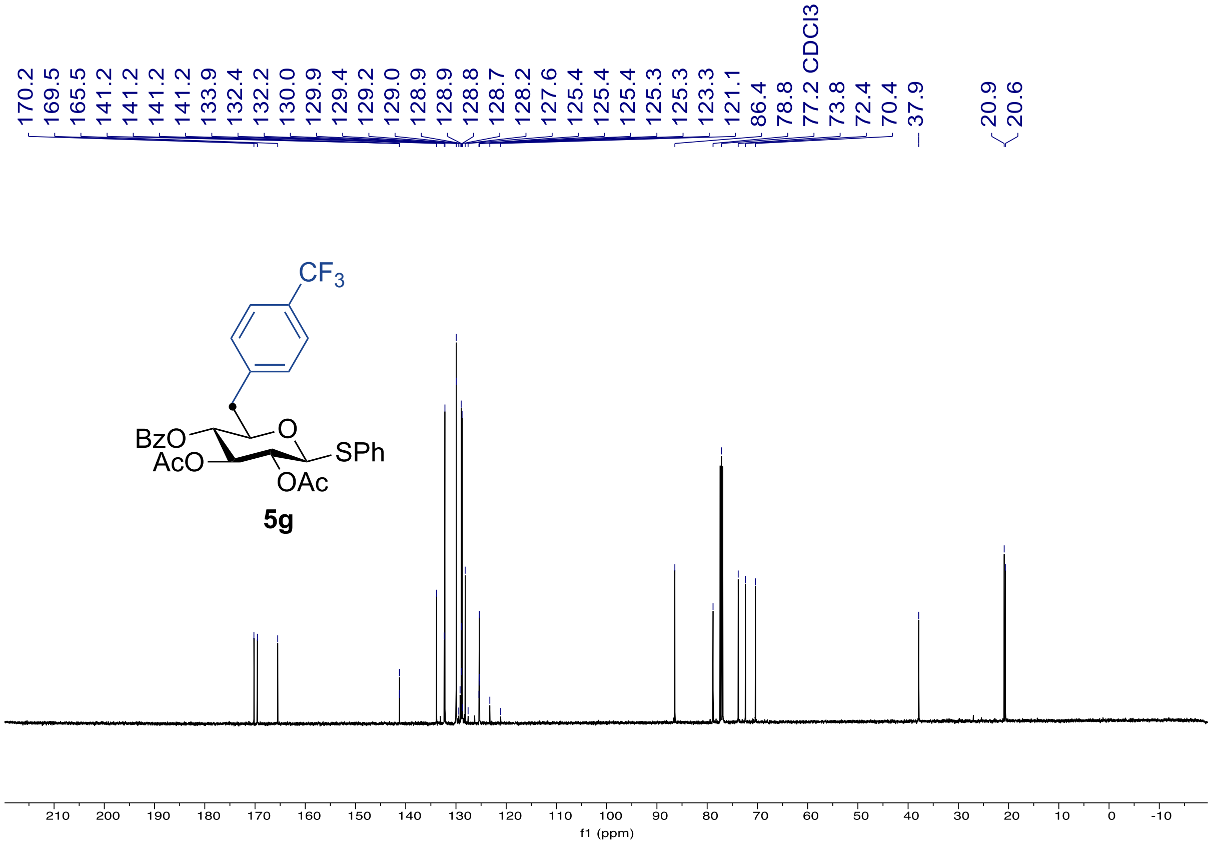

^{13}C NMR (126 MHz, CDCl$_3$) of 5g

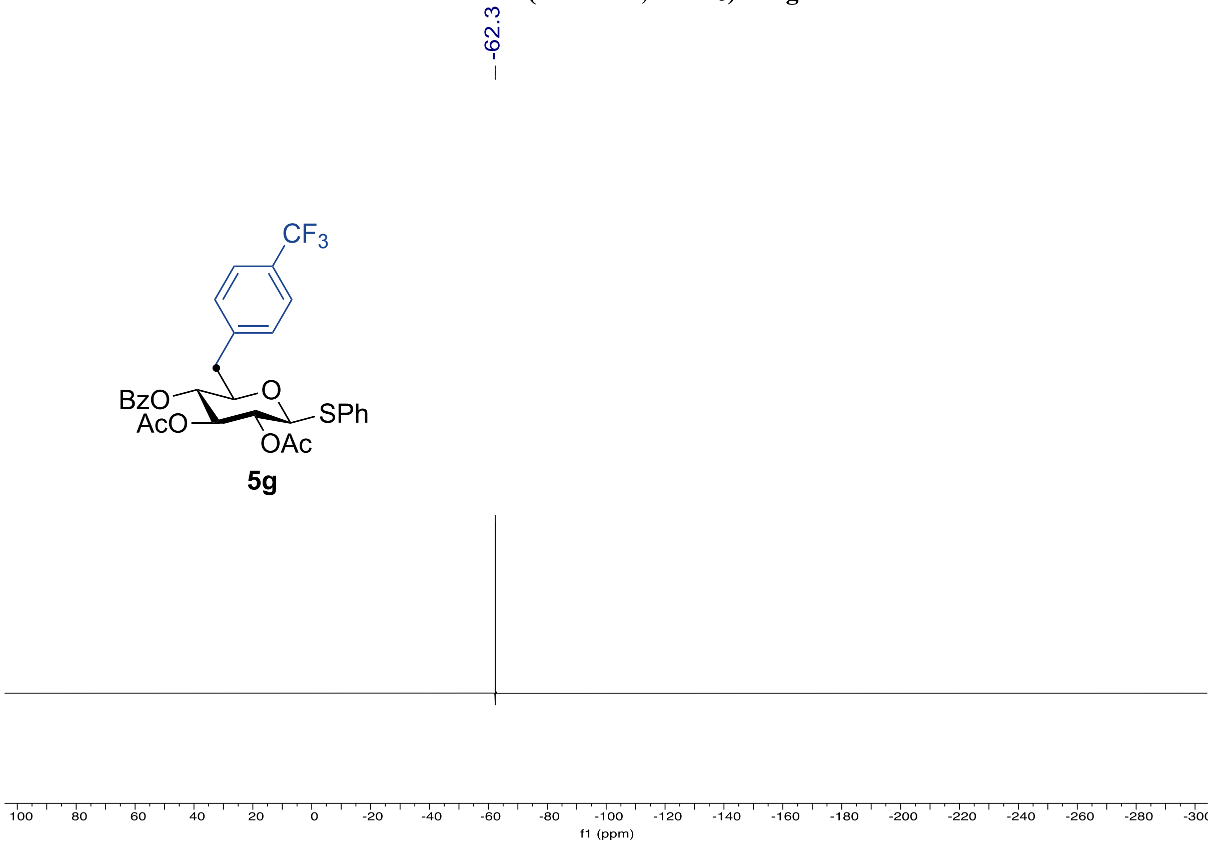

^{19}F NMR (471 MHz, CDCl$_3$) of 5g

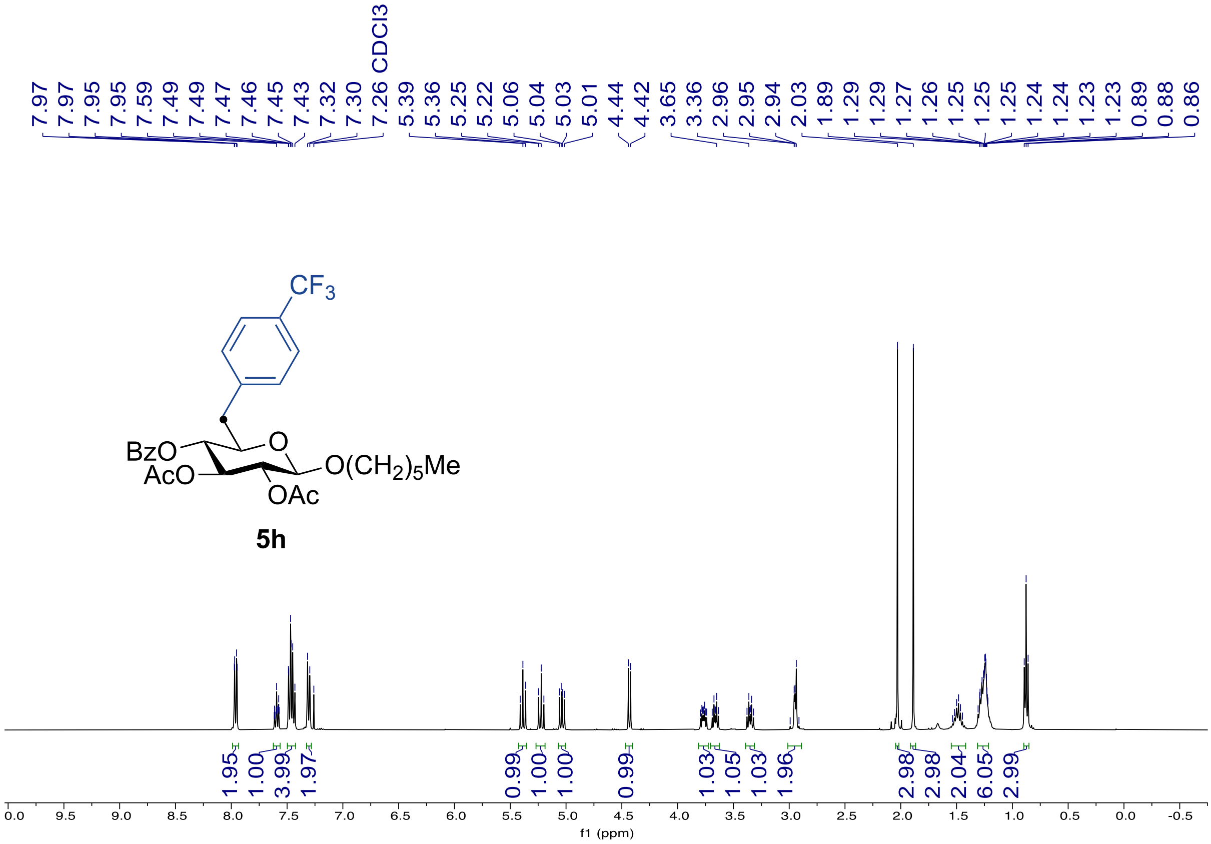

^{1}H NMR (400 MHz, $CDCl_3$) of 5h

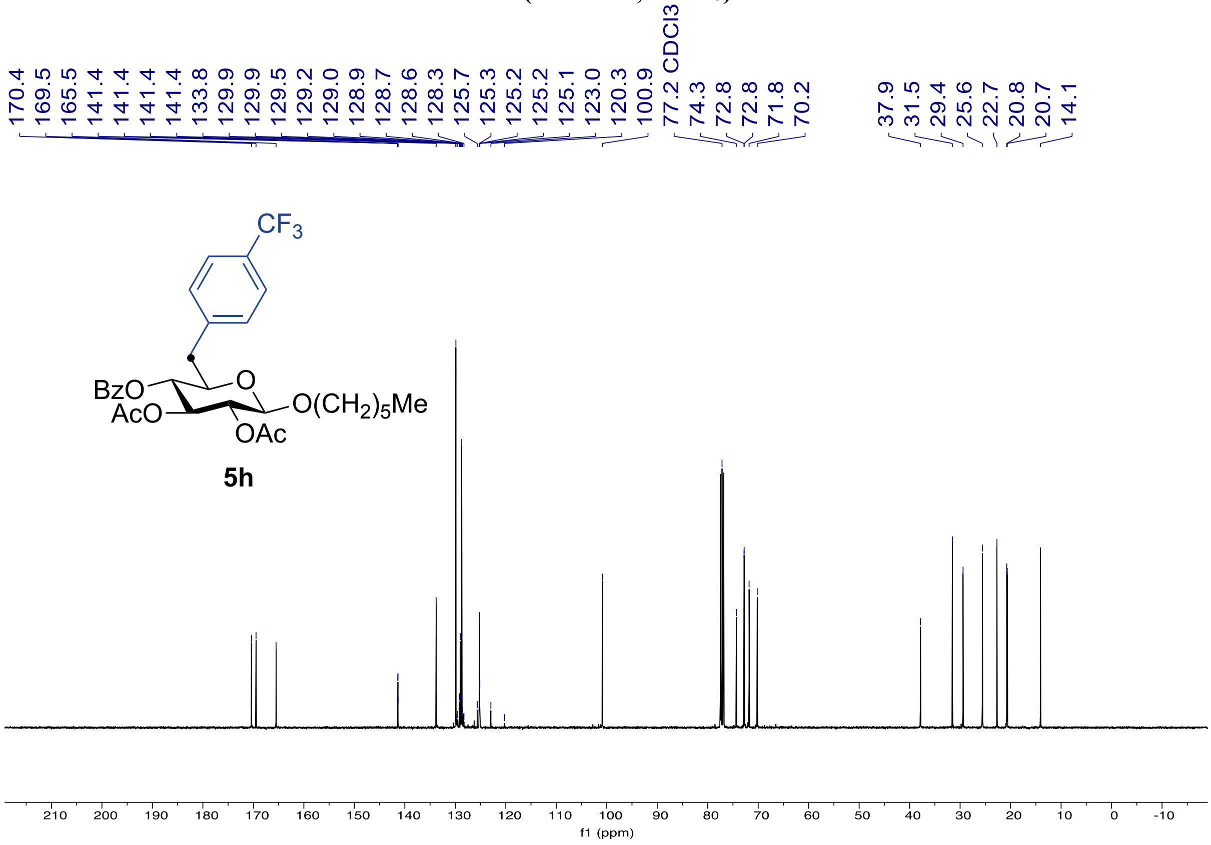

^{13}C NMR (101 MHz, $CDCl_3$) of 5h

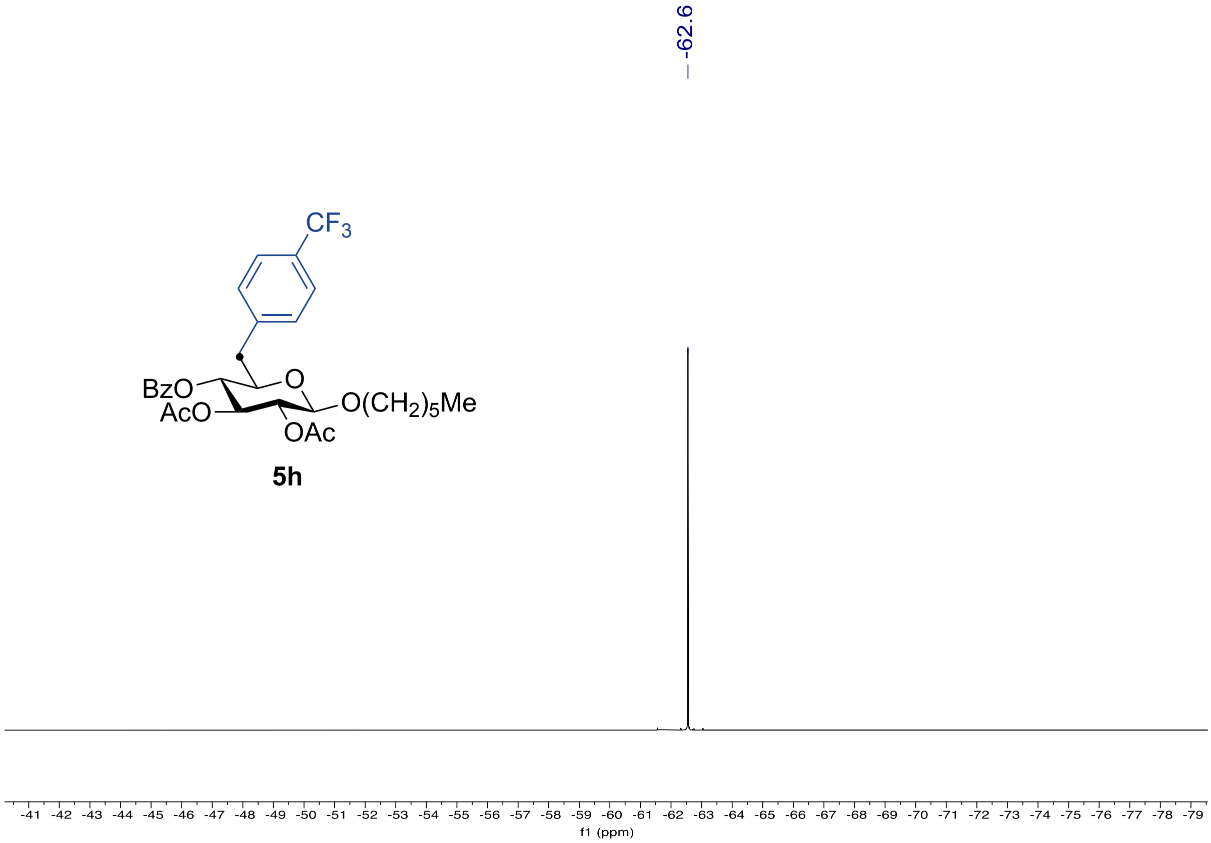

^{19}F NMR (376 MHz, $CDCl_3$) of 5h

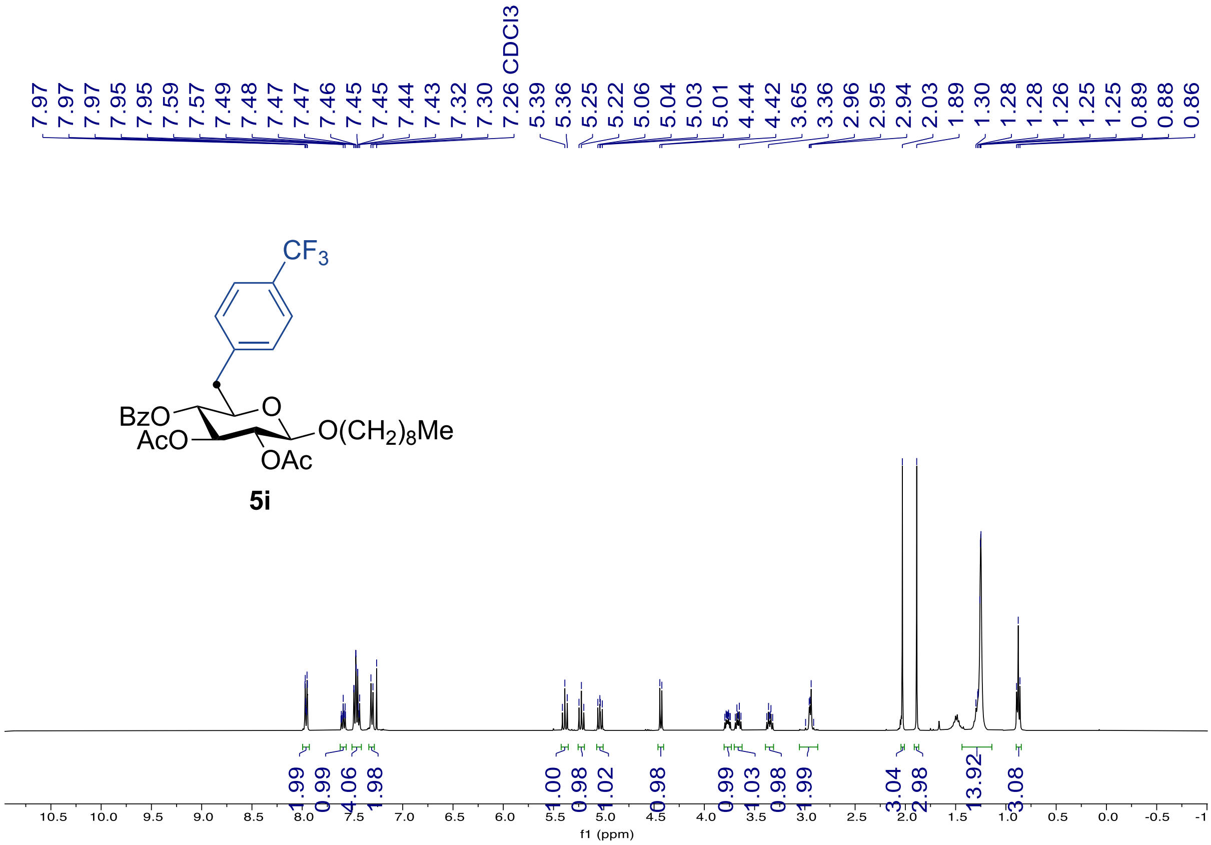

^{1}H NMR (400 MHz, $CDCl_3$) of 5i

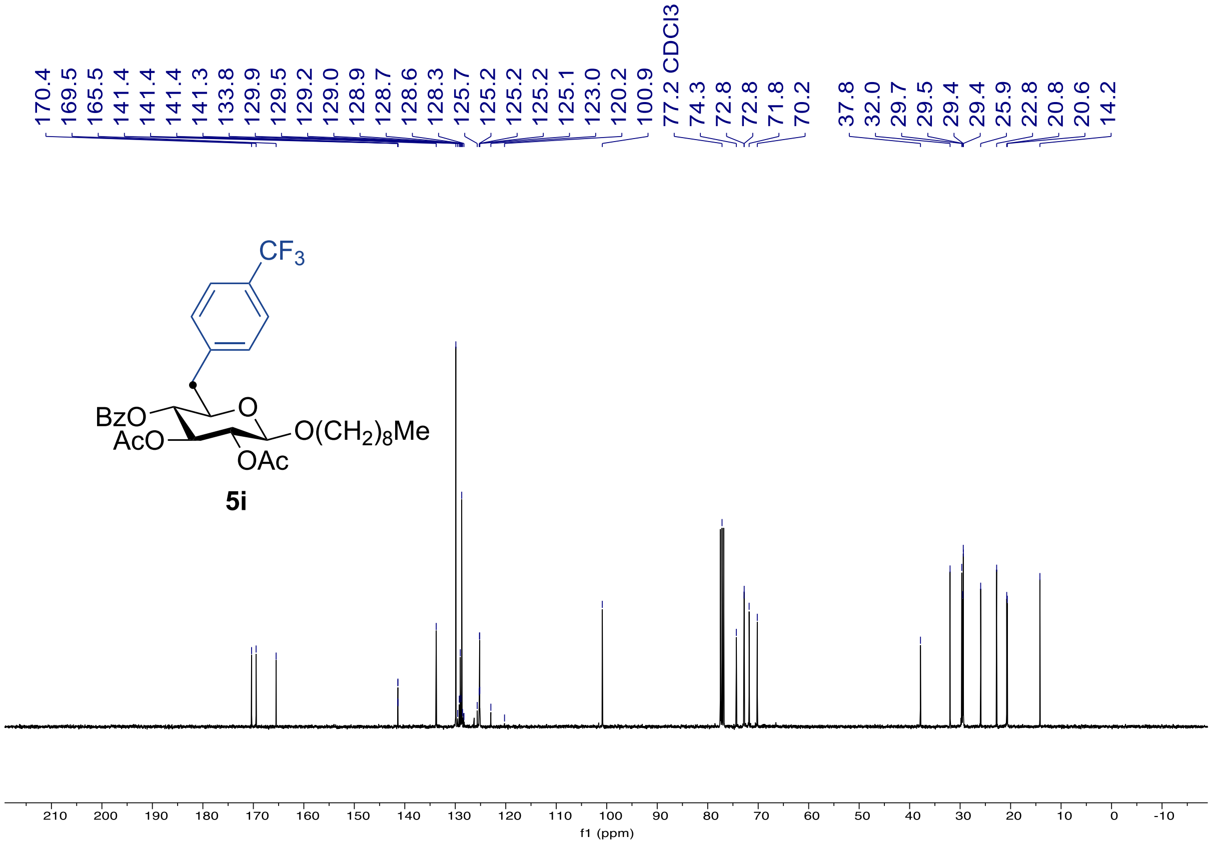

^{13}C NMR (101 MHz, $CDCl_3$) of 5i

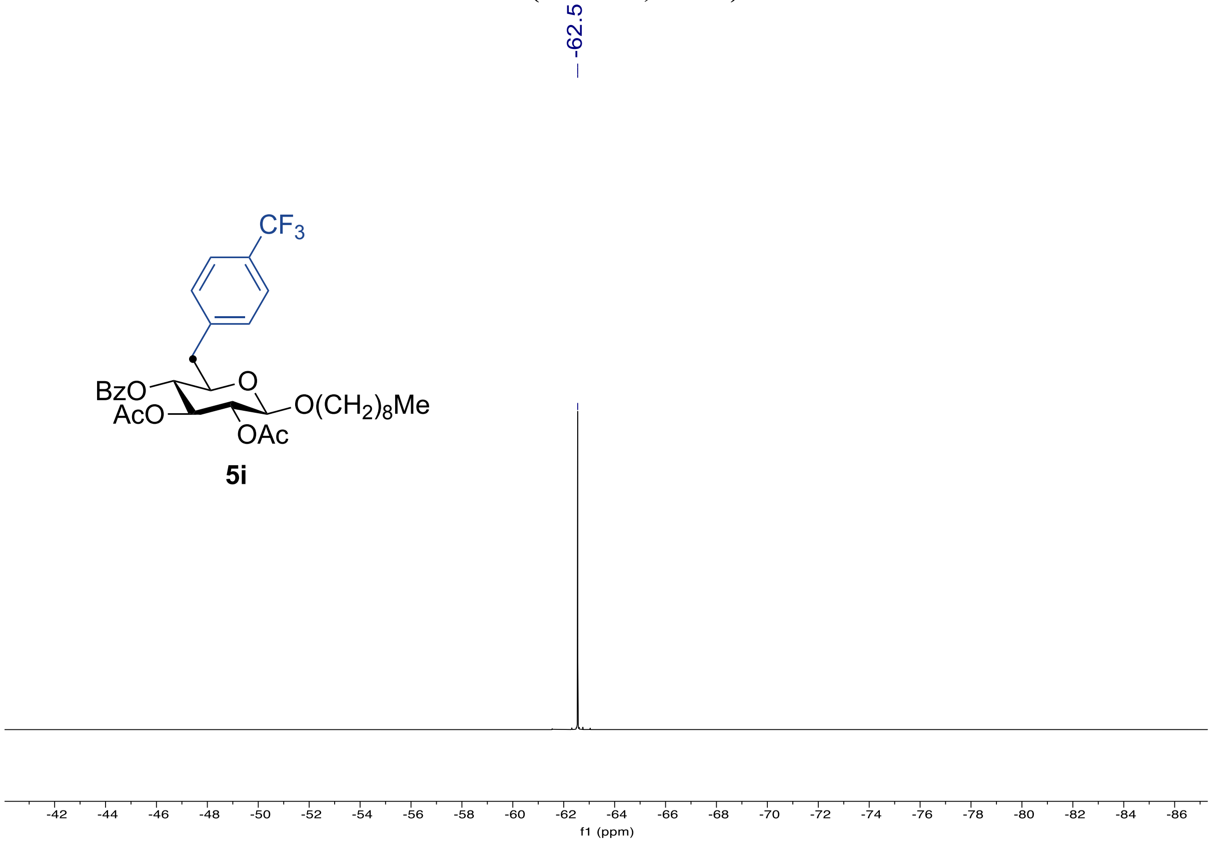

^{19}F NMR (376 MHz, $CDCl_3$) of 5i

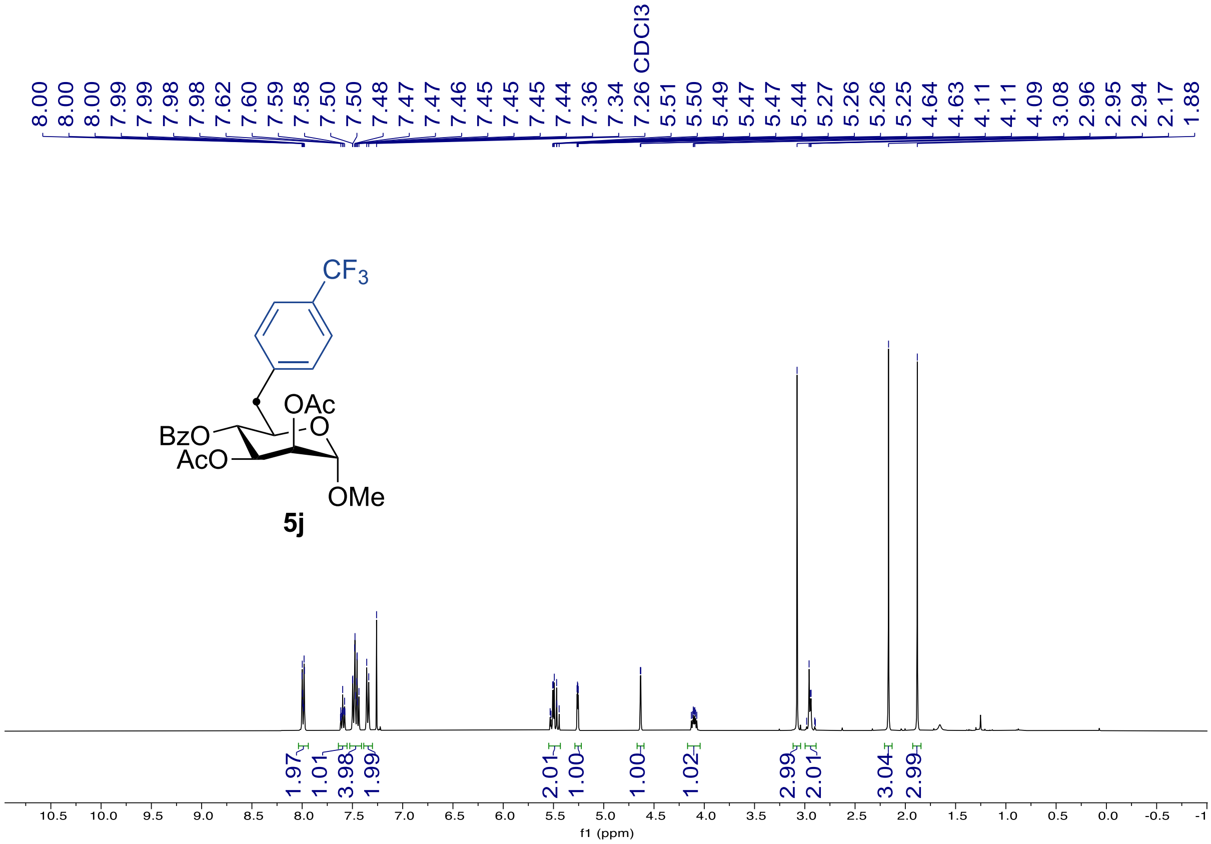

^{1}H NMR (400 MHz, CDCl3) of 5j

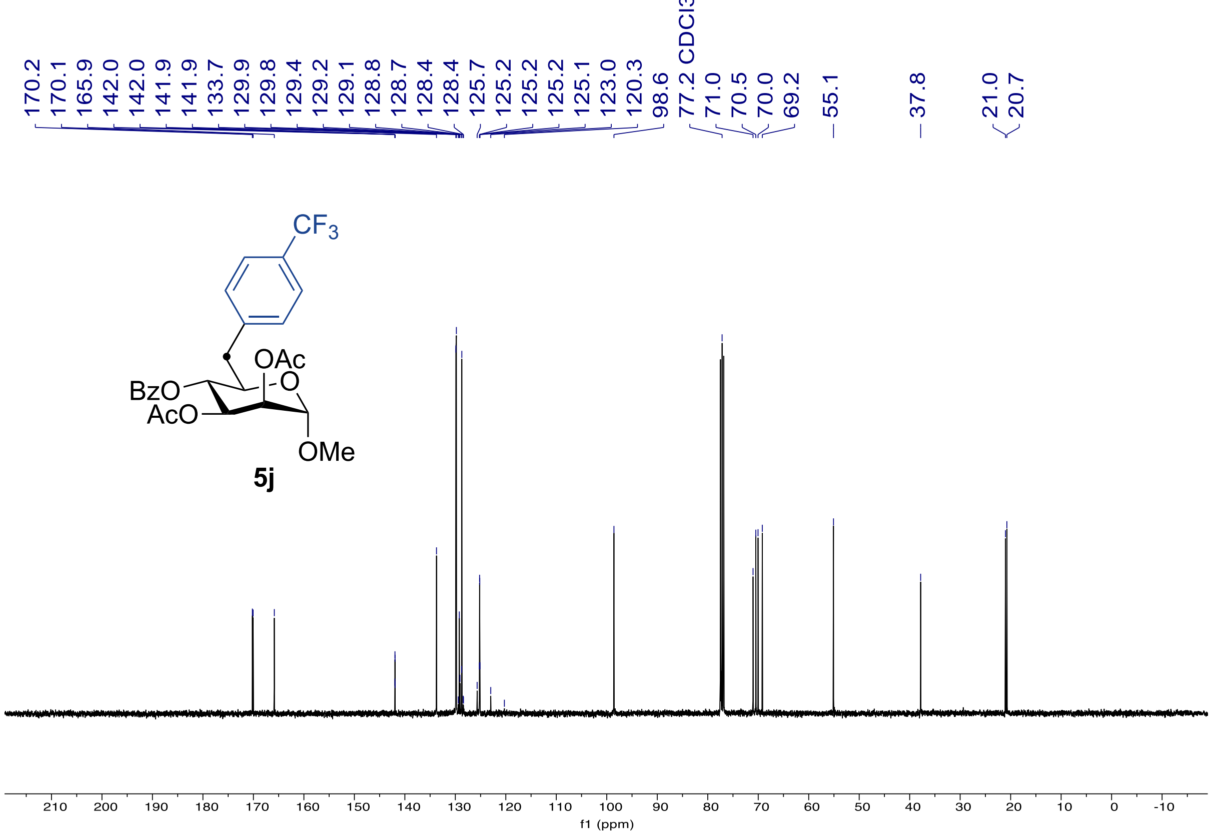

^{13}C NMR (101 MHz, CDCl3) of 5j

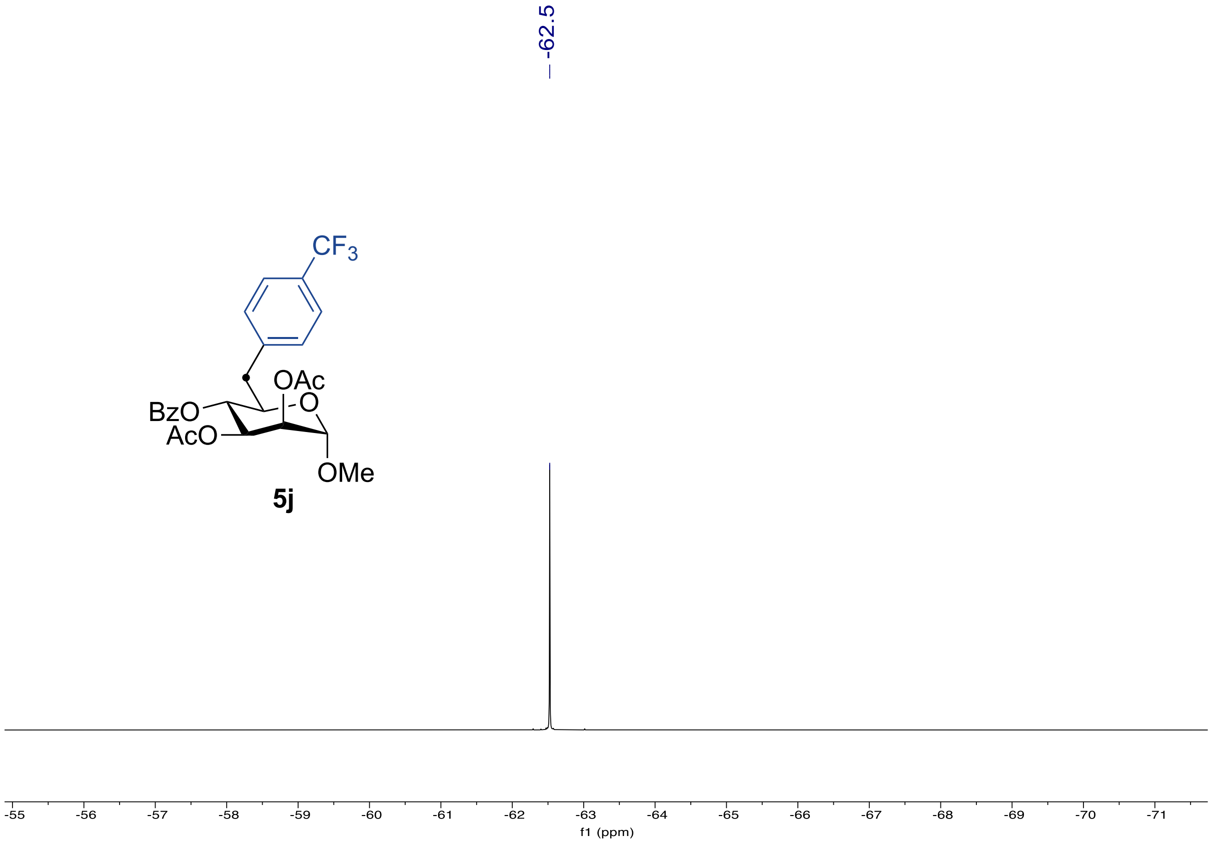

^{19}F NMR (376 MHz, CDCl₃) of 5j

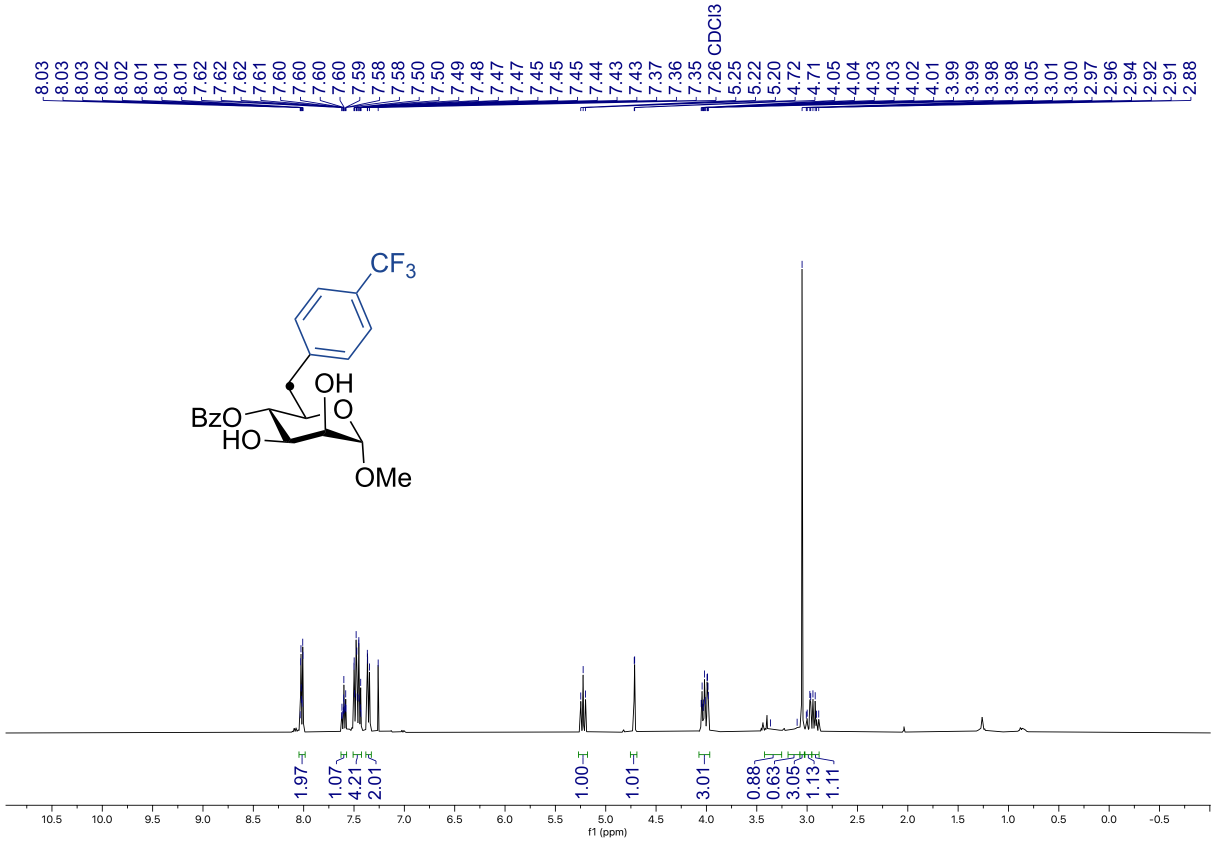

^{1}H NMR (400 MHz, CDCl₃) of 5k

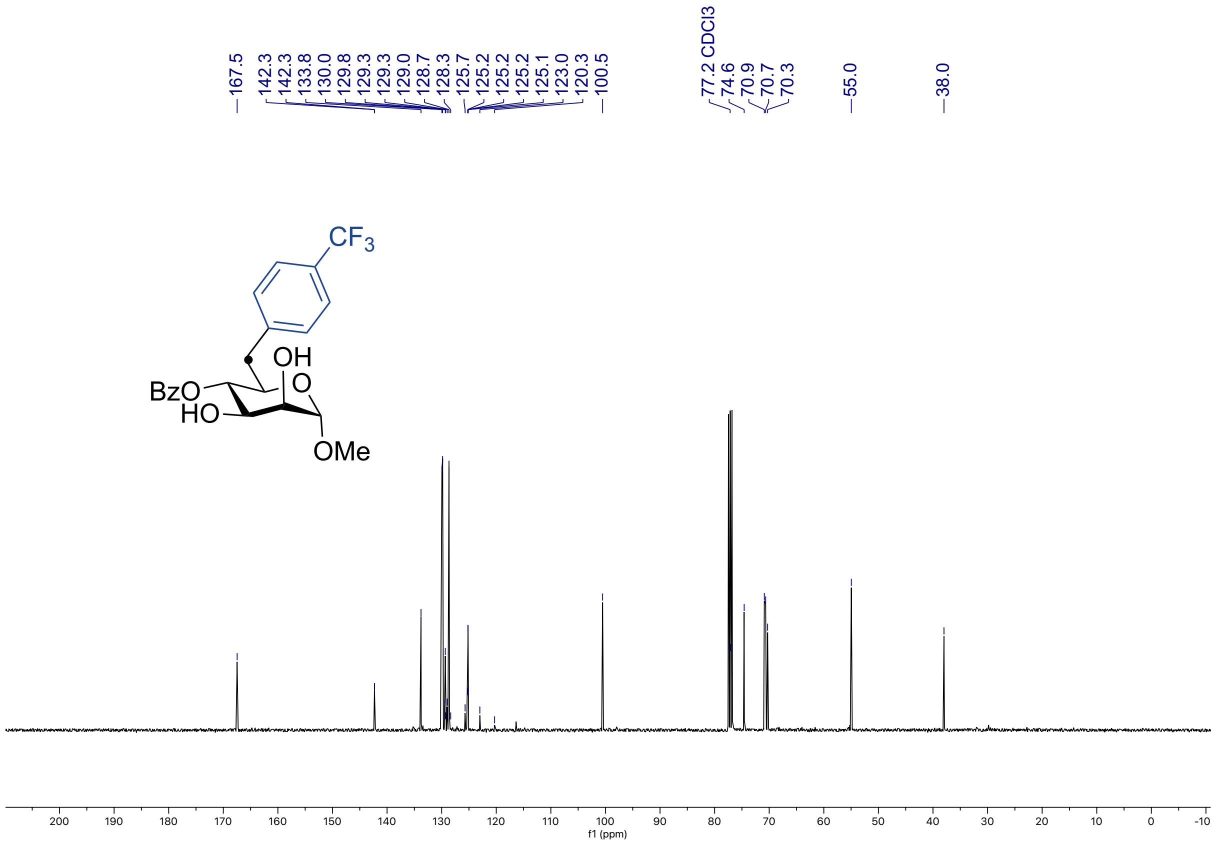

^{13}C NMR (101 MHz, CDCl$_3$) of 5k

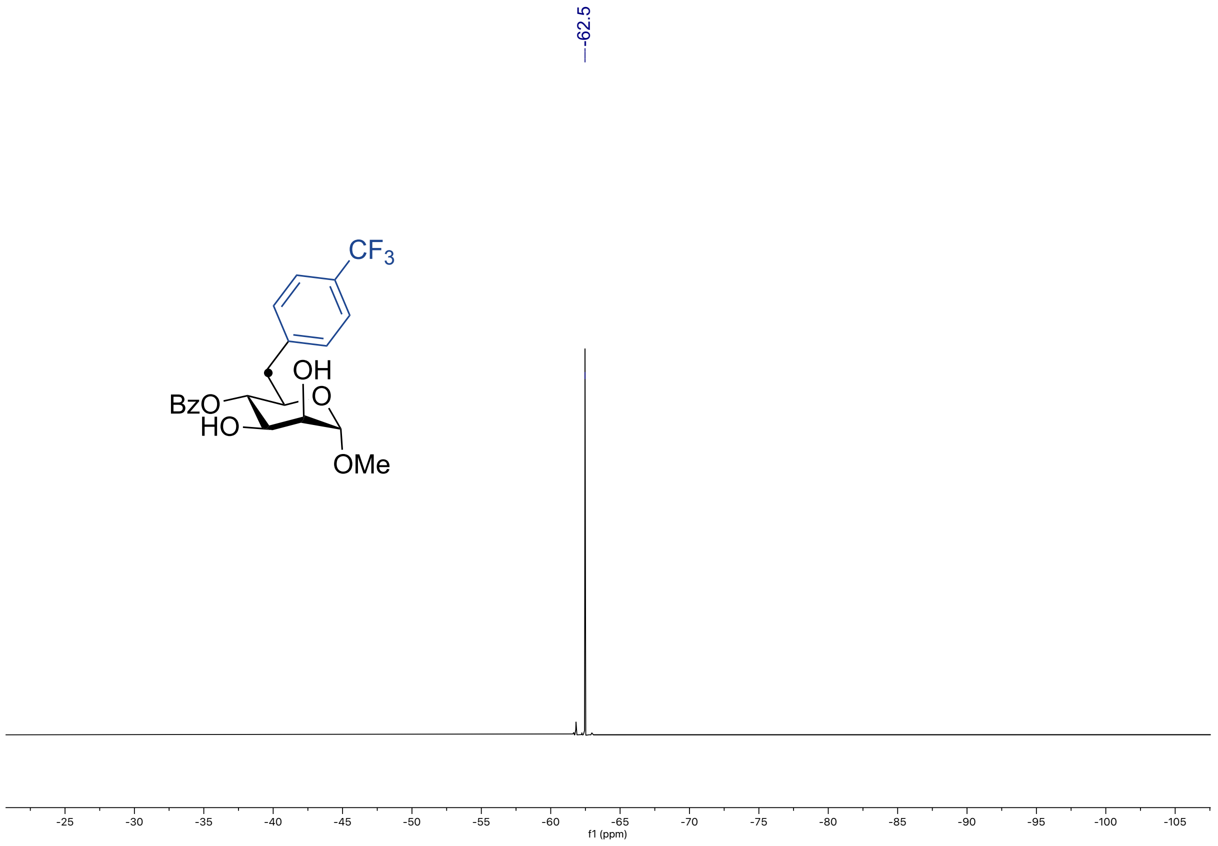

^{19}F NMR (376 MHz, CDCl$_3$) of 5k

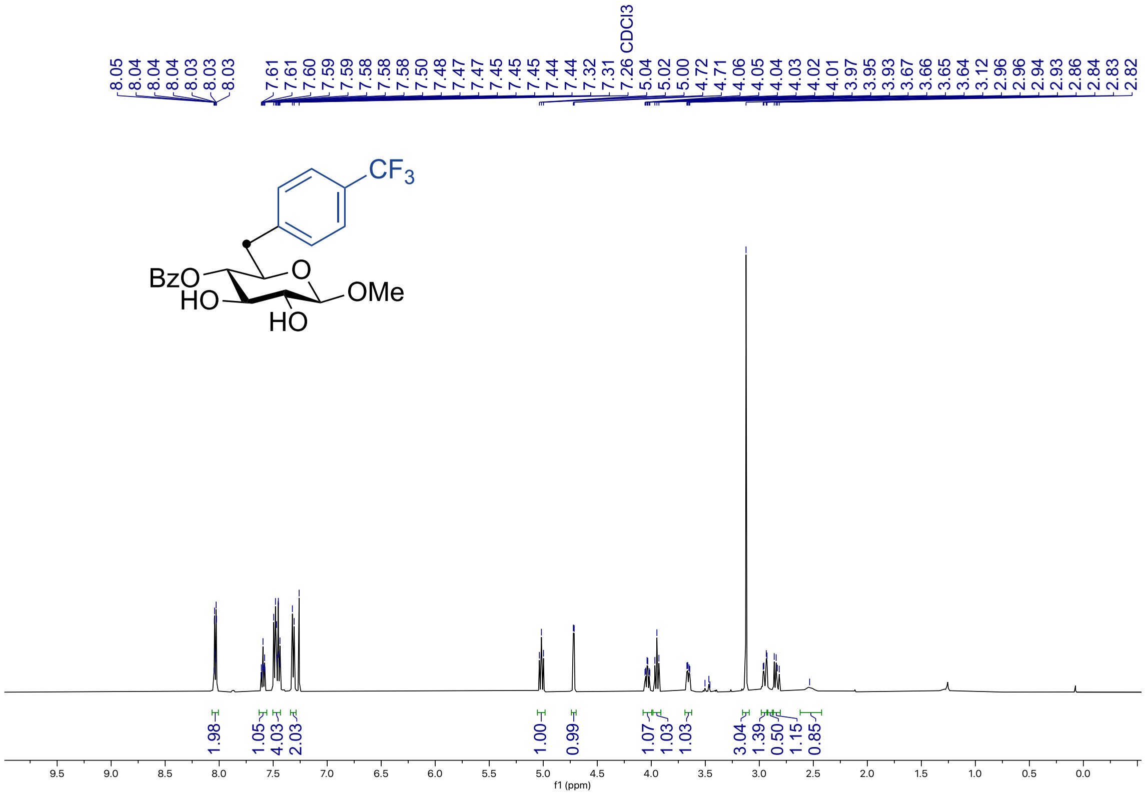

^{1}H NMR (500 MHz, $CDCl_3$) of 5l

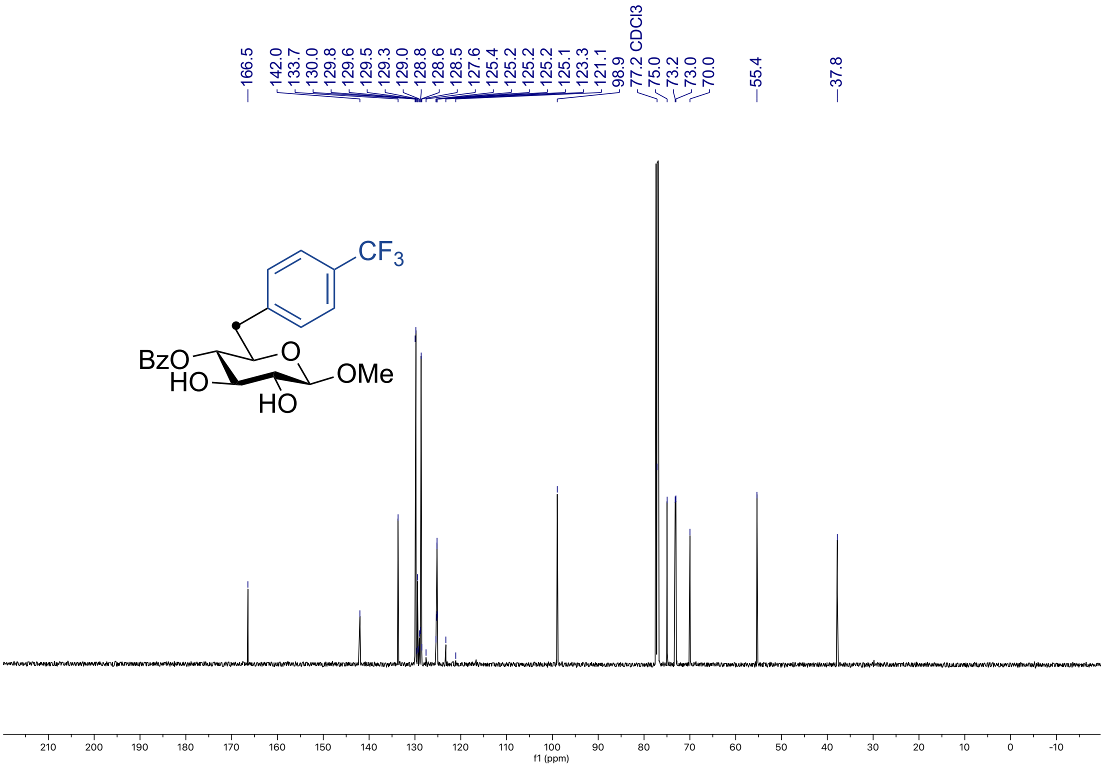

^{13}C NMR (126 MHz, $CDCl_3$) of 5l

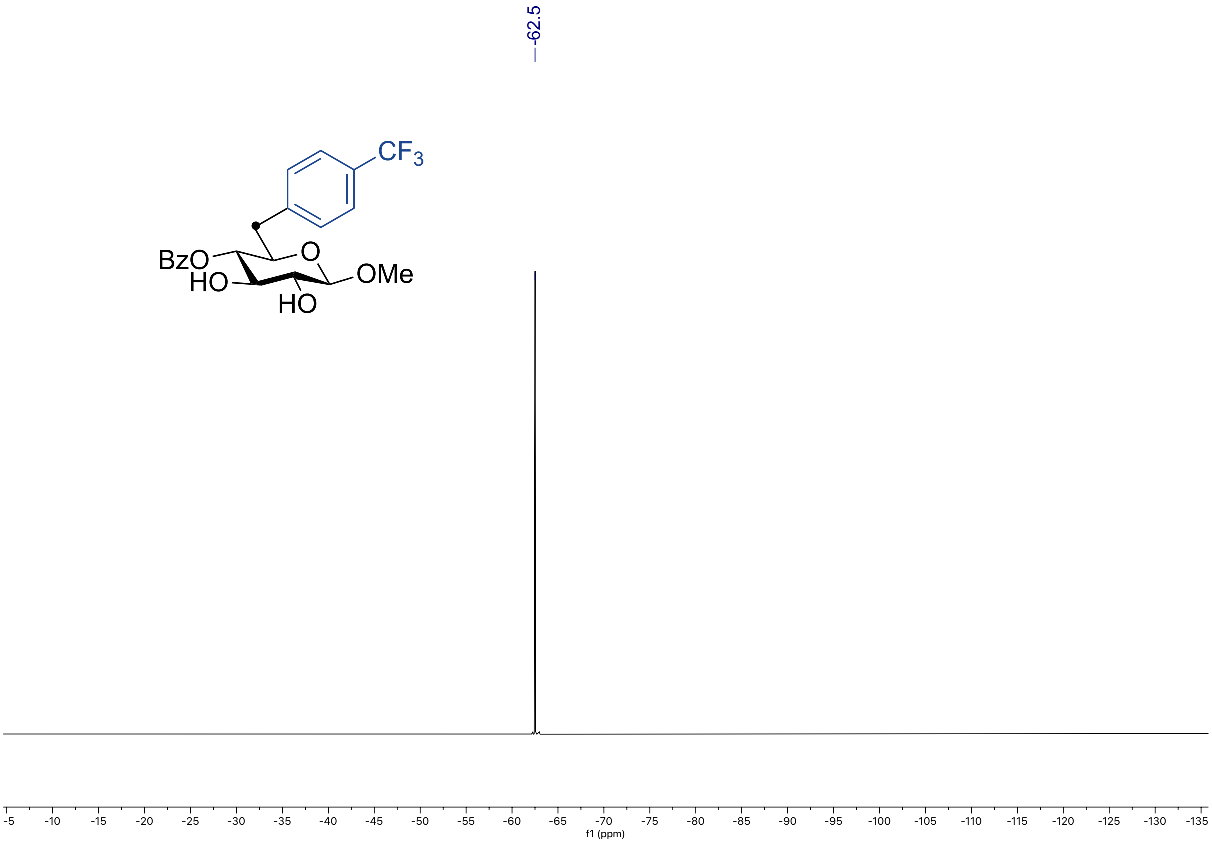

^{19}F NMR (376 MHz, CDCl$_3$) of 5l

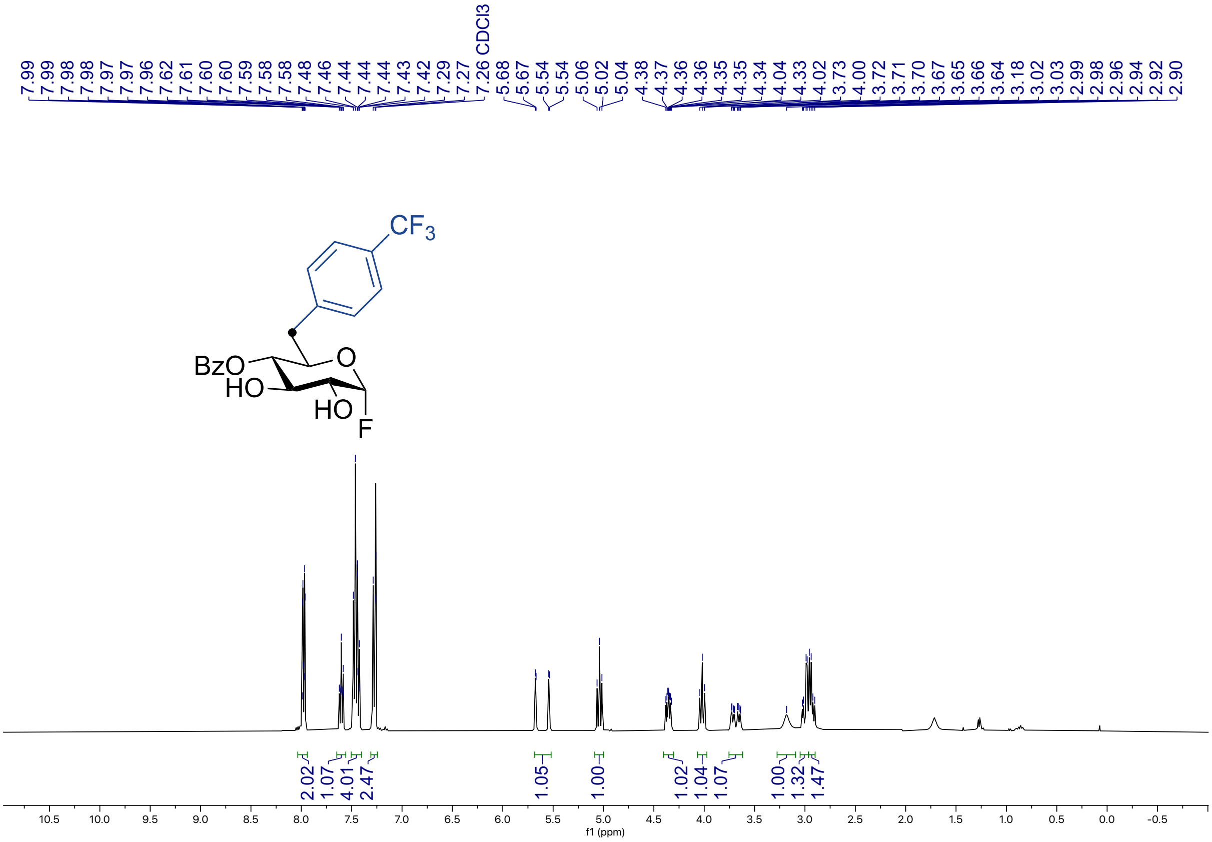

^{1}H NMR (400 MHz, CDCl$_3$) of 5m

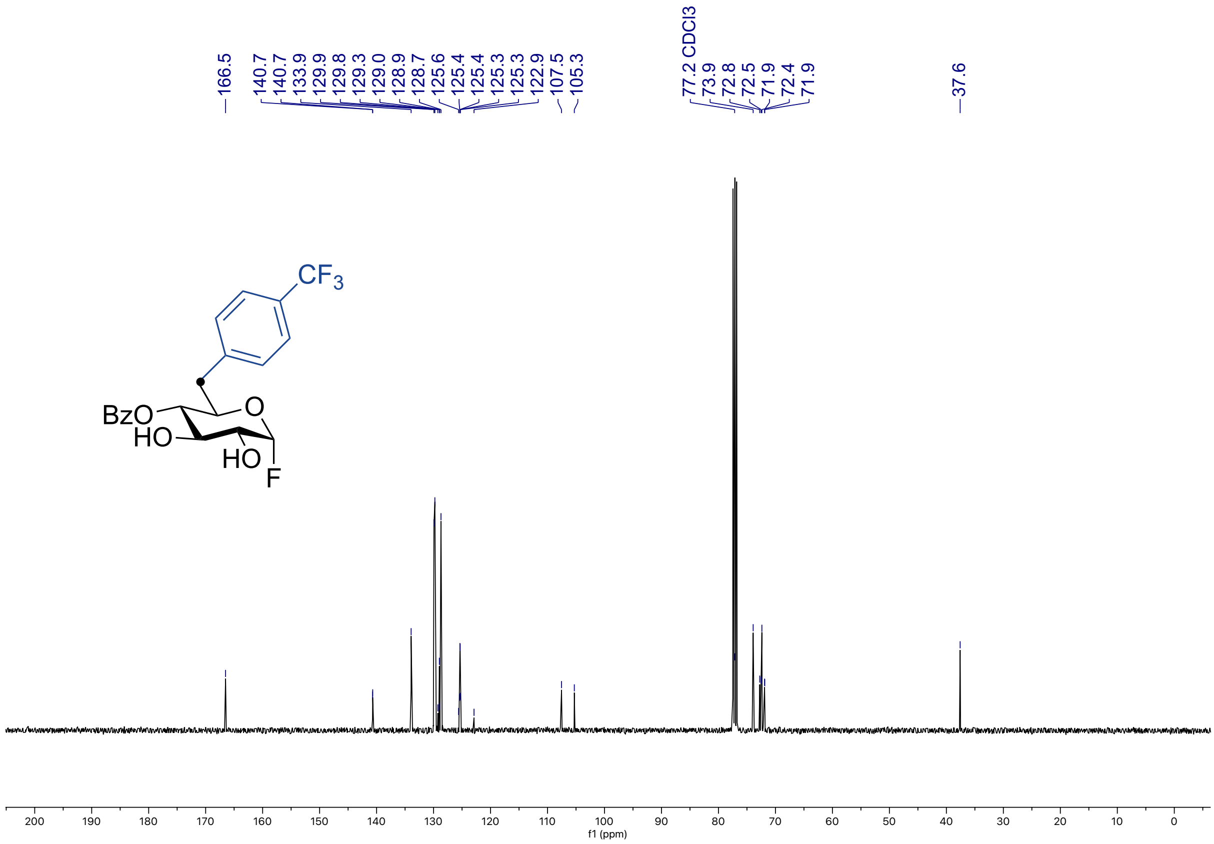

^{13}C NMR (101 MHz, CDCl₃) of 5m

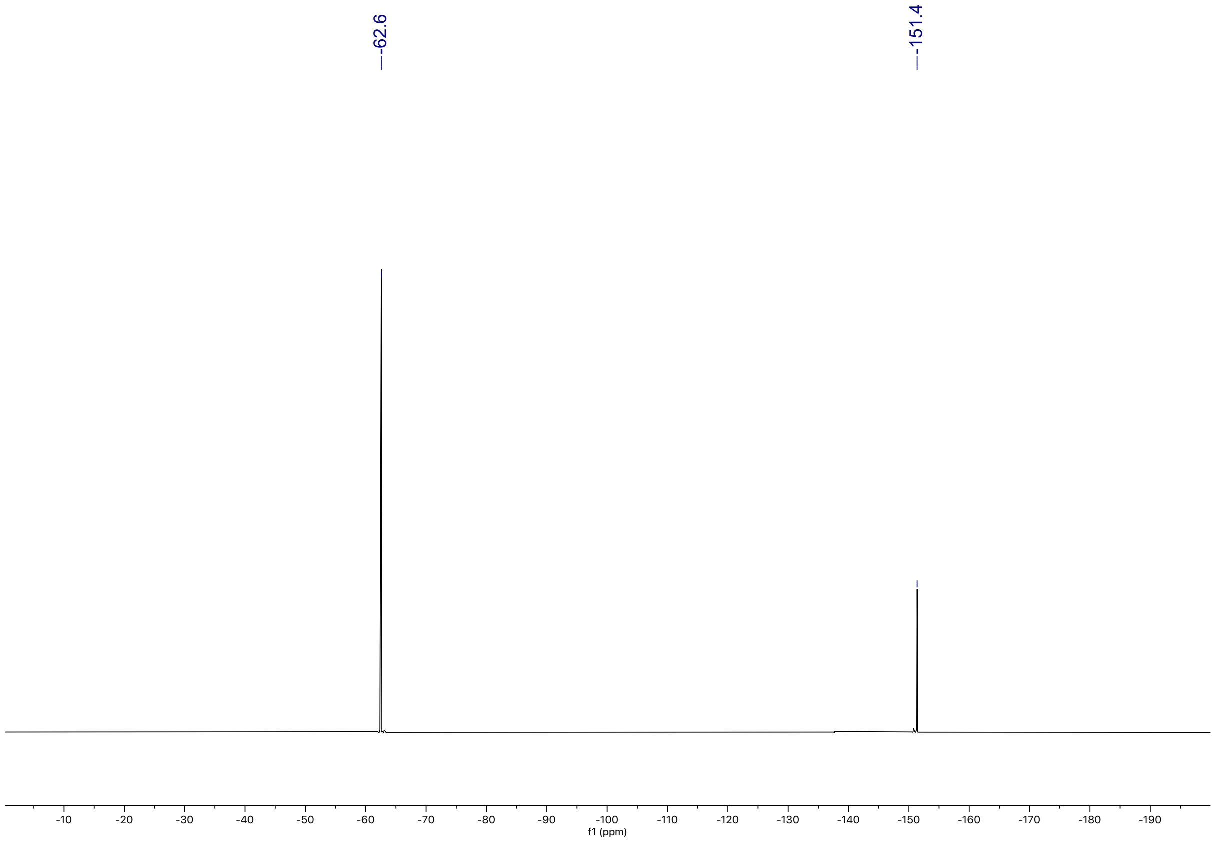

^{19}F NMR (376 MHz, CDCl₃) of 5m

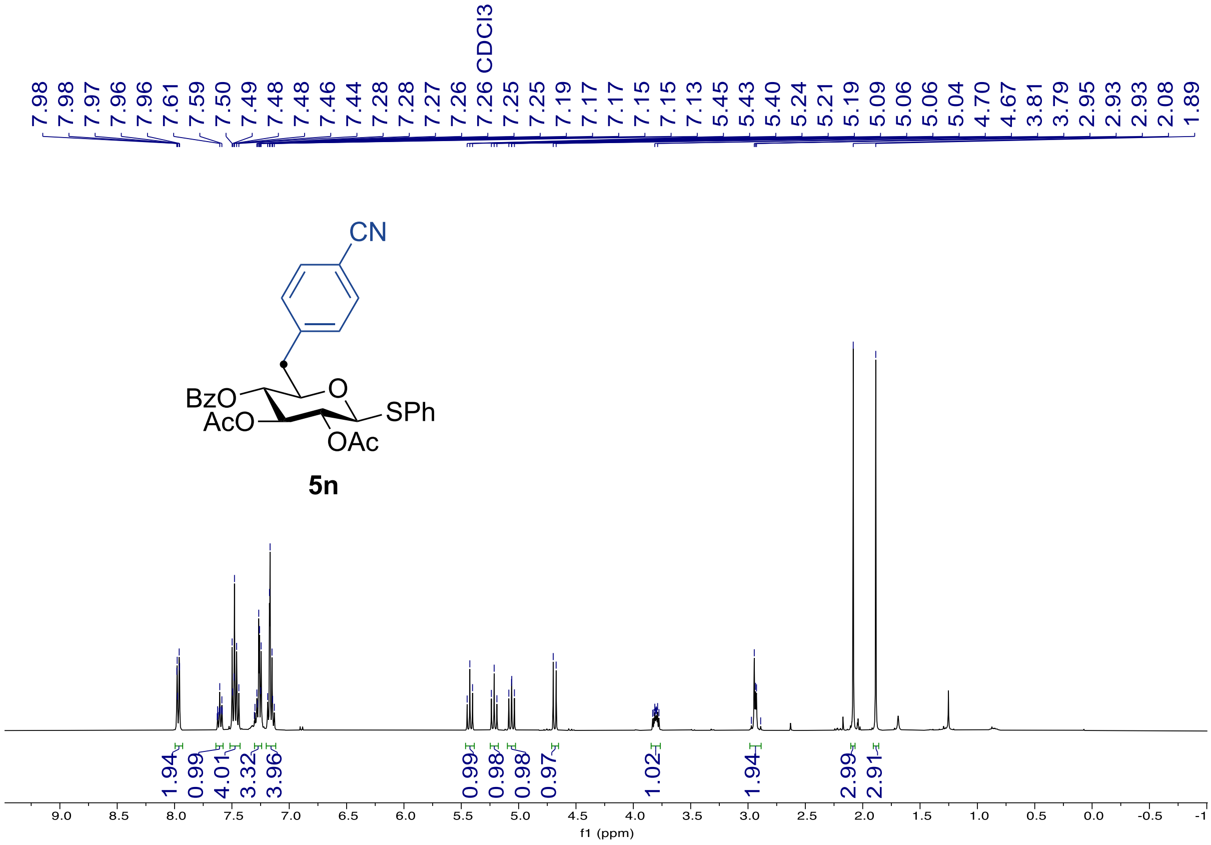

^{1}H NMR (400 MHz, CDCl3) of 5n

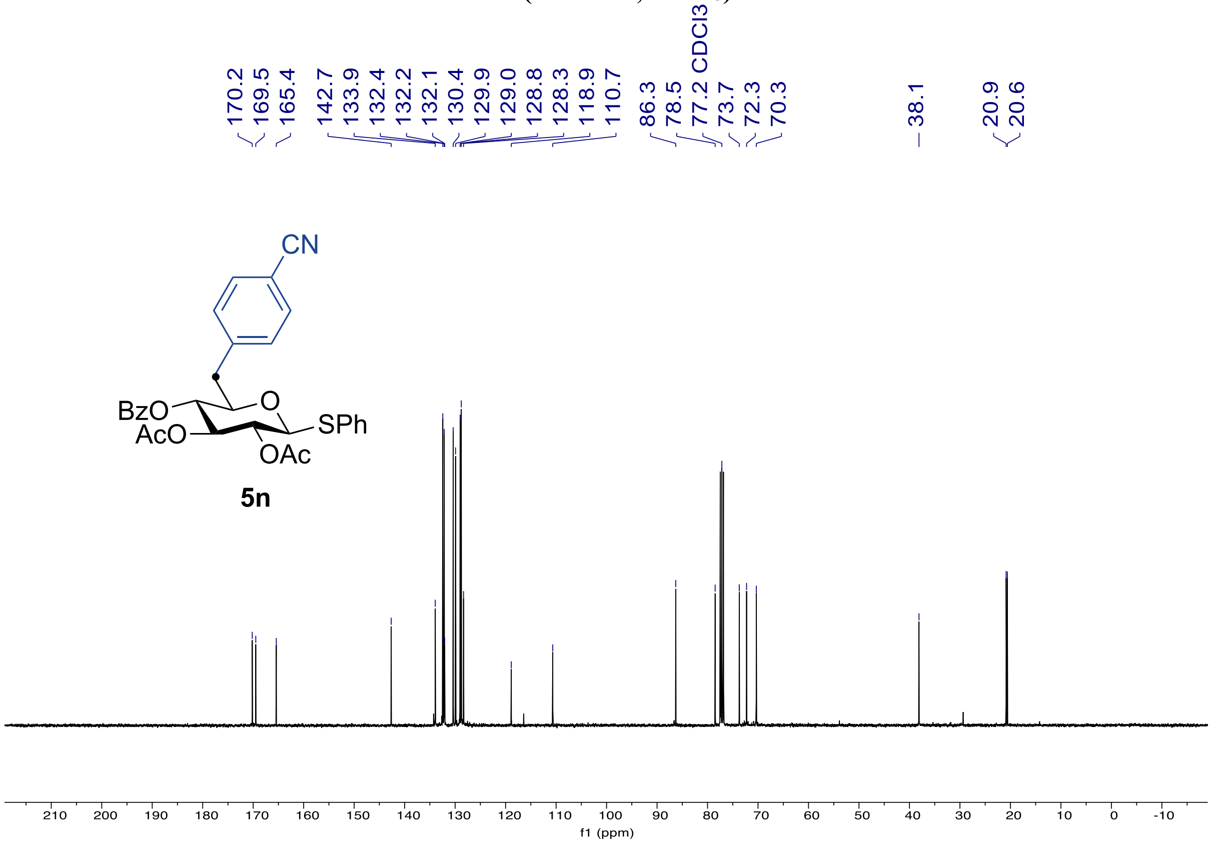

^{13}C NMR (101 MHz, CDCl3) of 5n

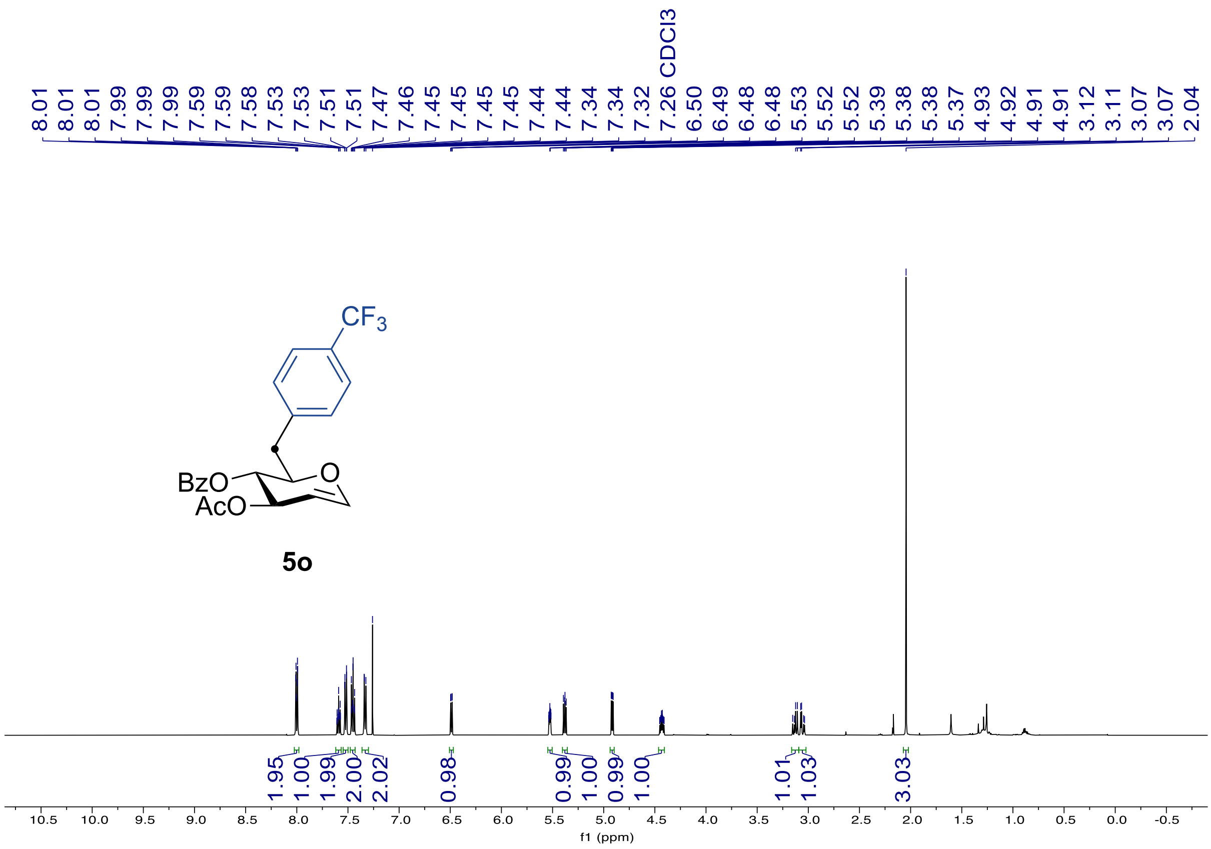

^{1}H NMR (500 MHz, CDCl3) of 5o

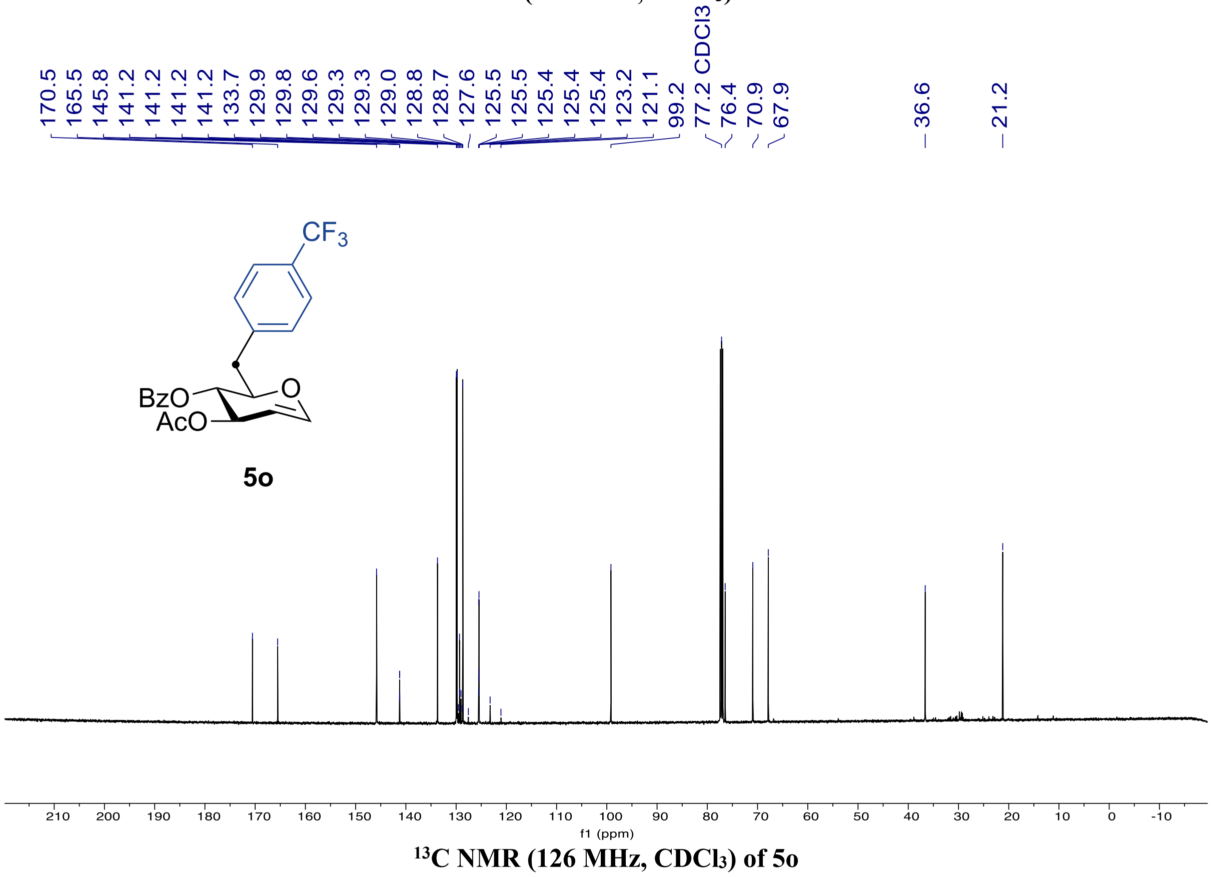

^{13}C NMR (126 MHz, CDCl3) of 5o

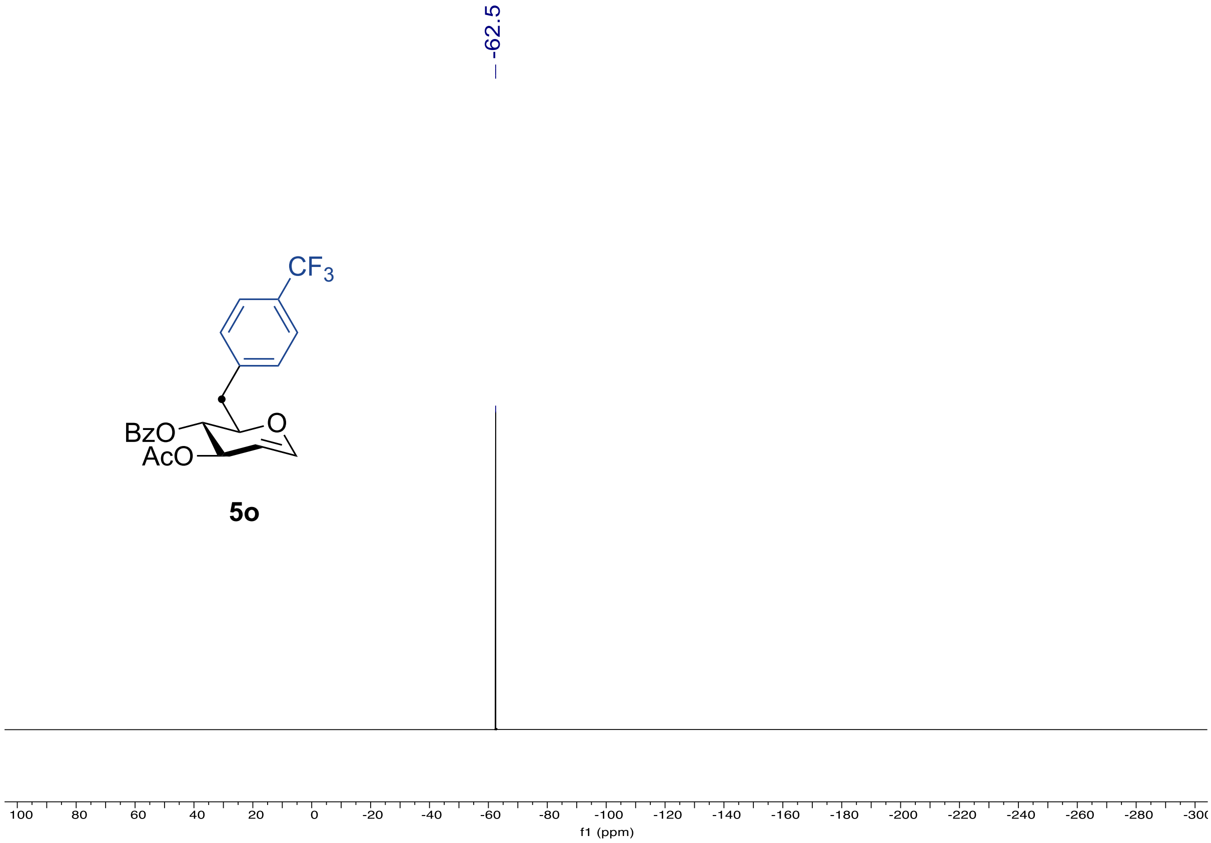

^{19}F NMR (471 MHz, CDCl$_3$) of 5o

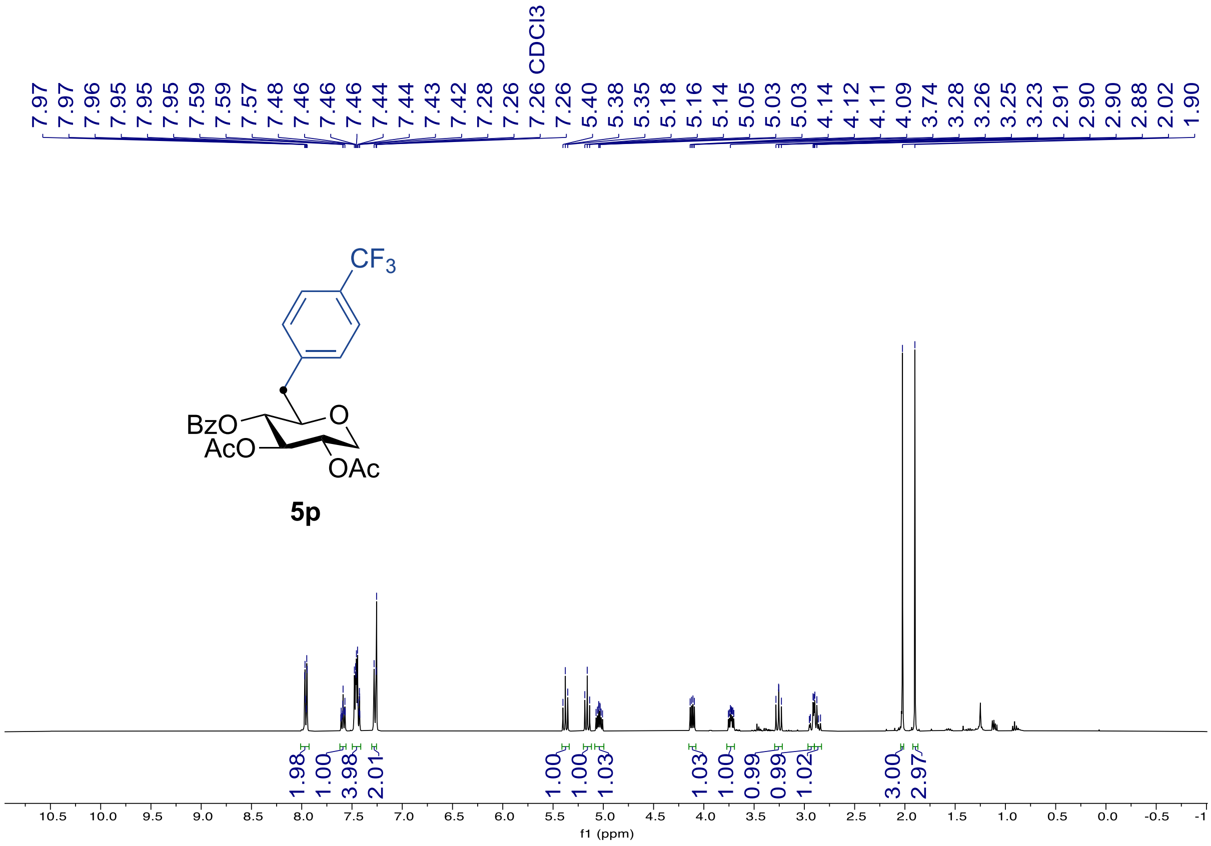

^{1}H NMR (400 MHz, CDCl$_3$) of 5p

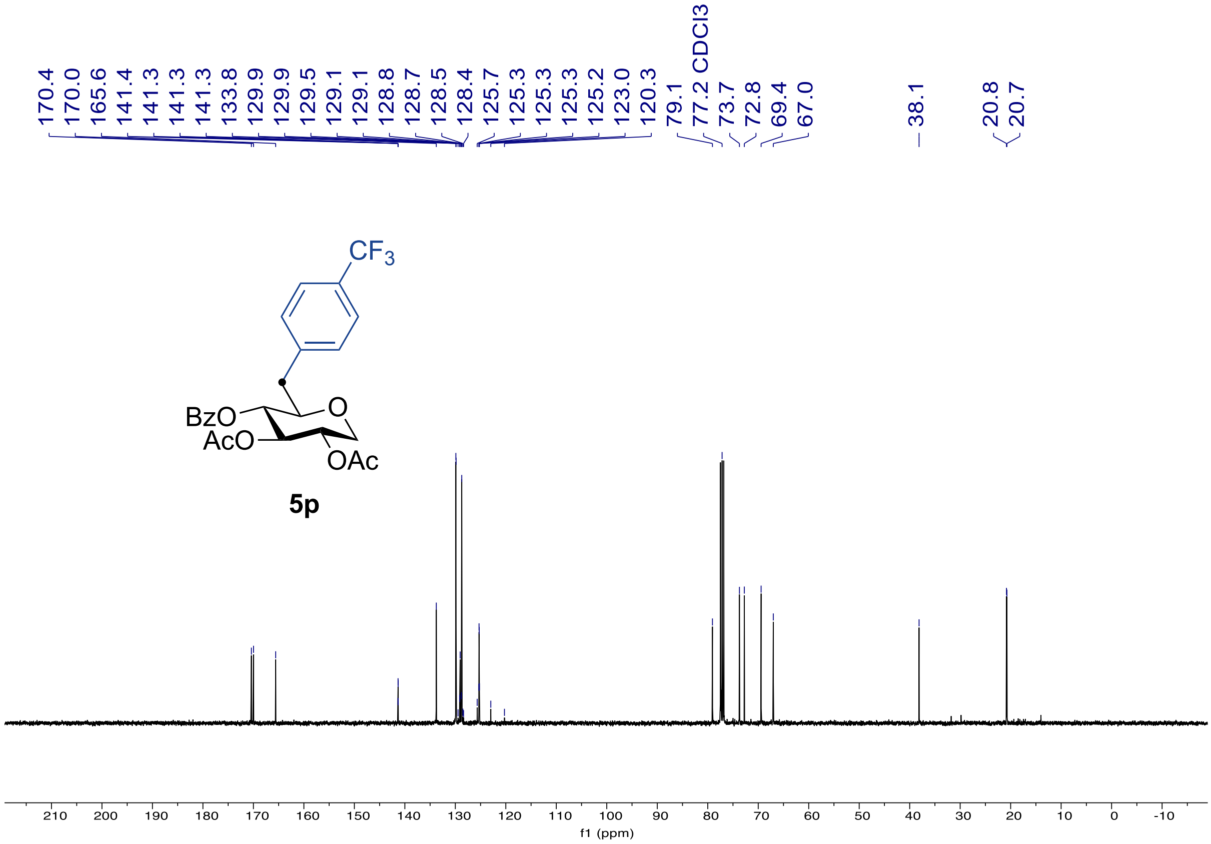

^{13}C NMR (101 MHz, CDCl$_3$) of 5p

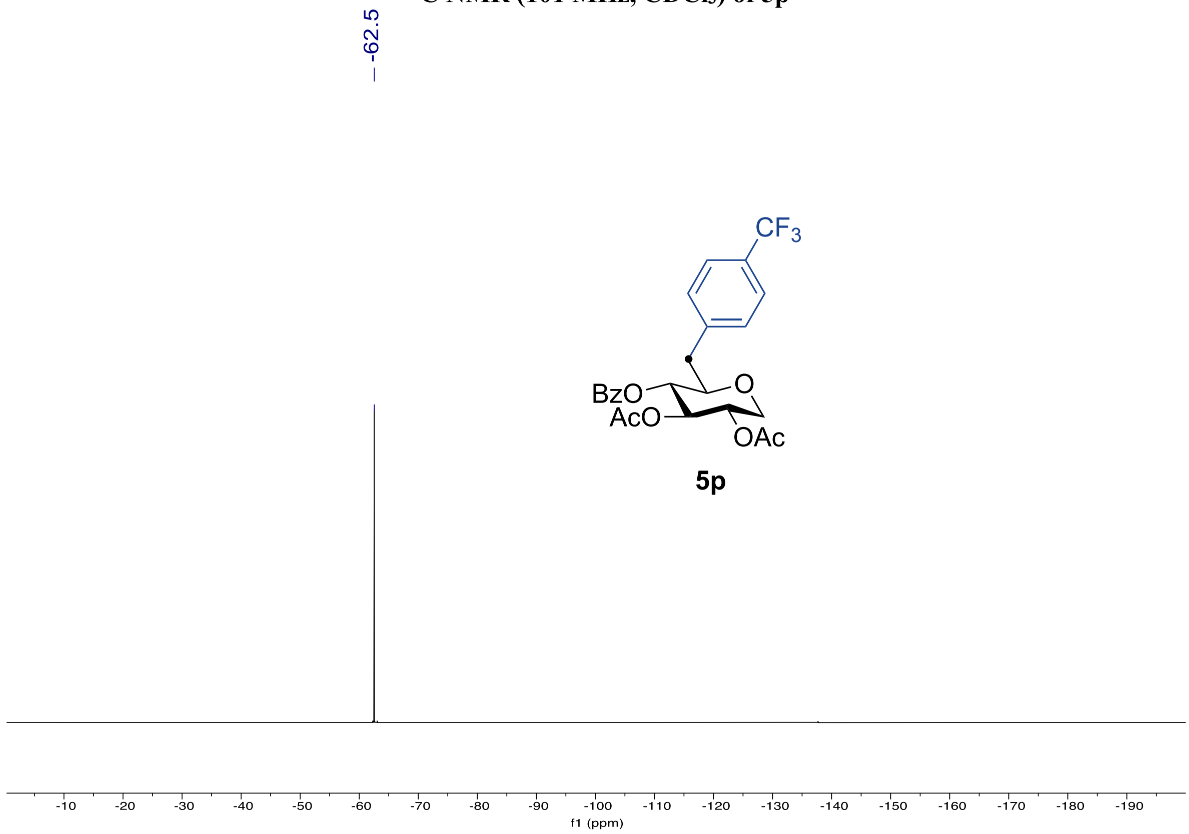

^{19}F NMR (376 MHz, CDCl$_3$) of 5p

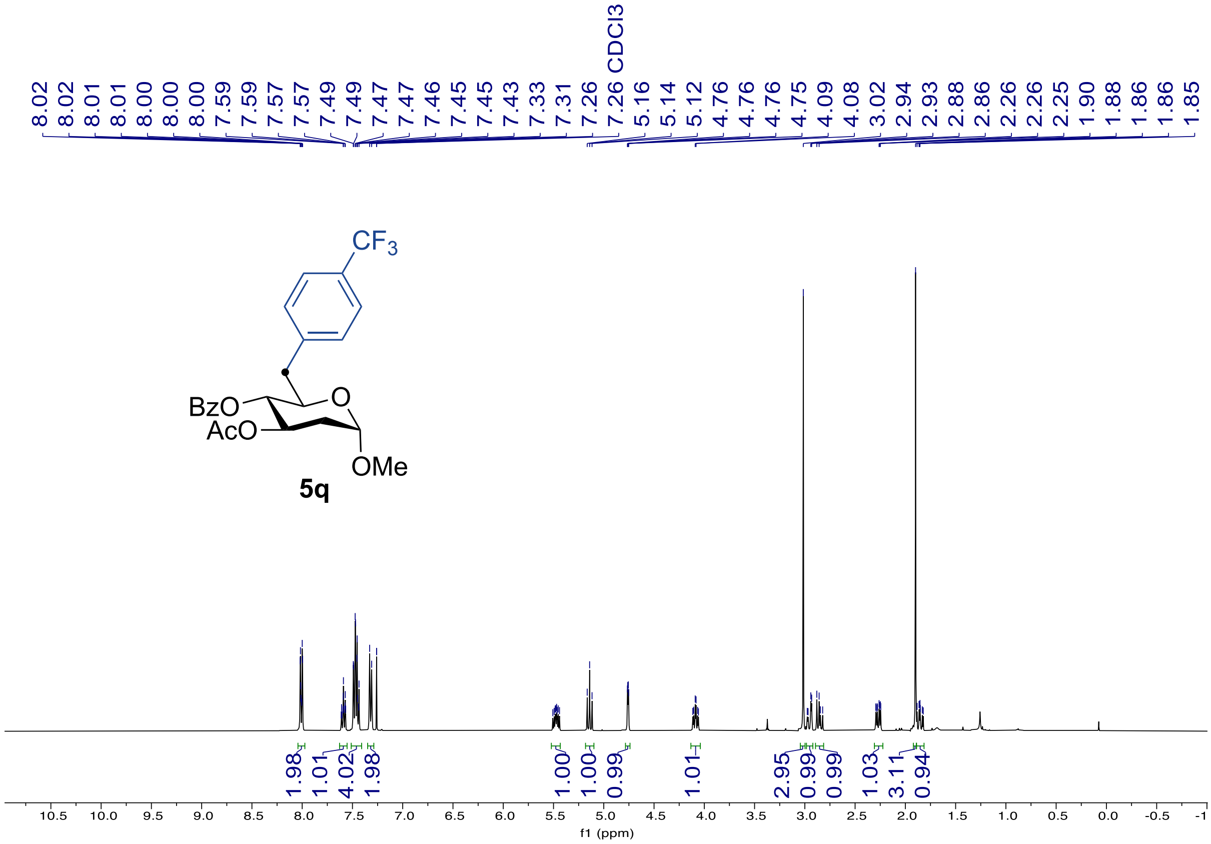

^{1}H NMR (400 MHz, CDCl₃) of 5q

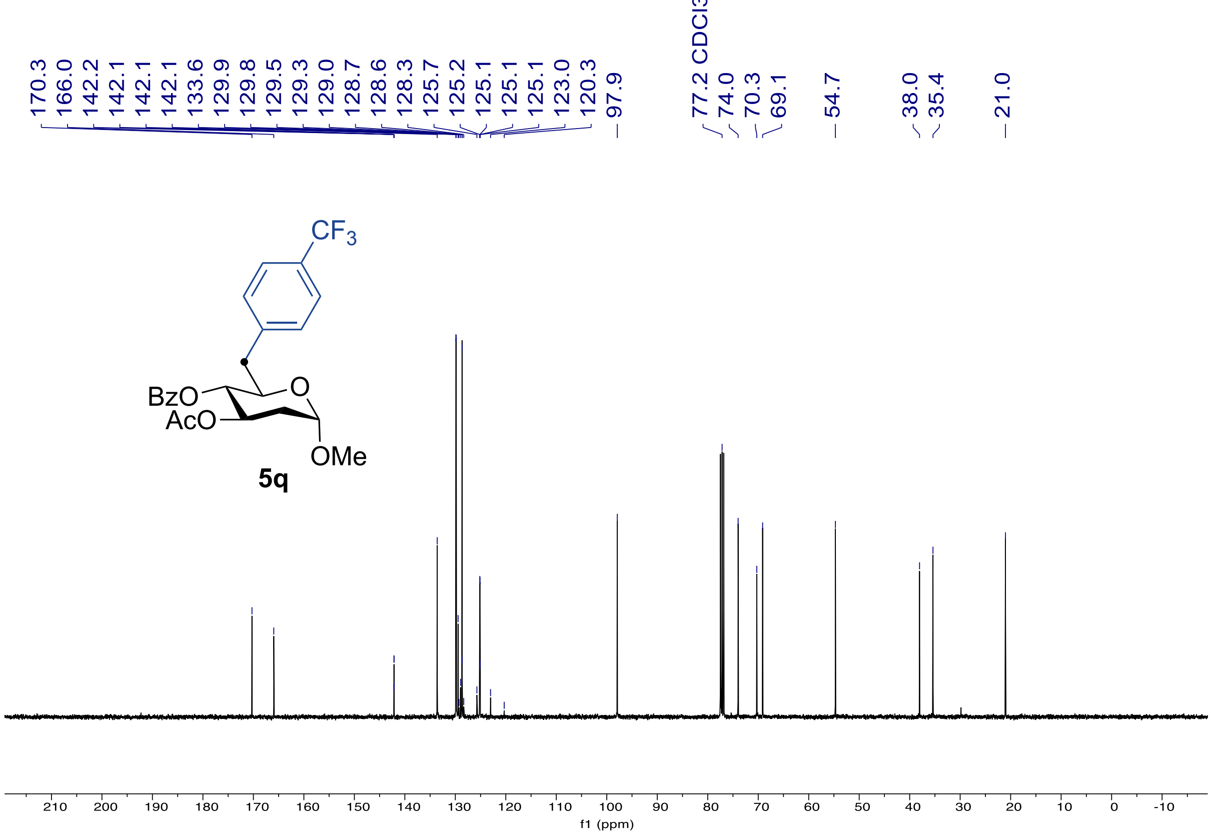

^{13}C NMR (101 MHz, CDCl₃) of 5q

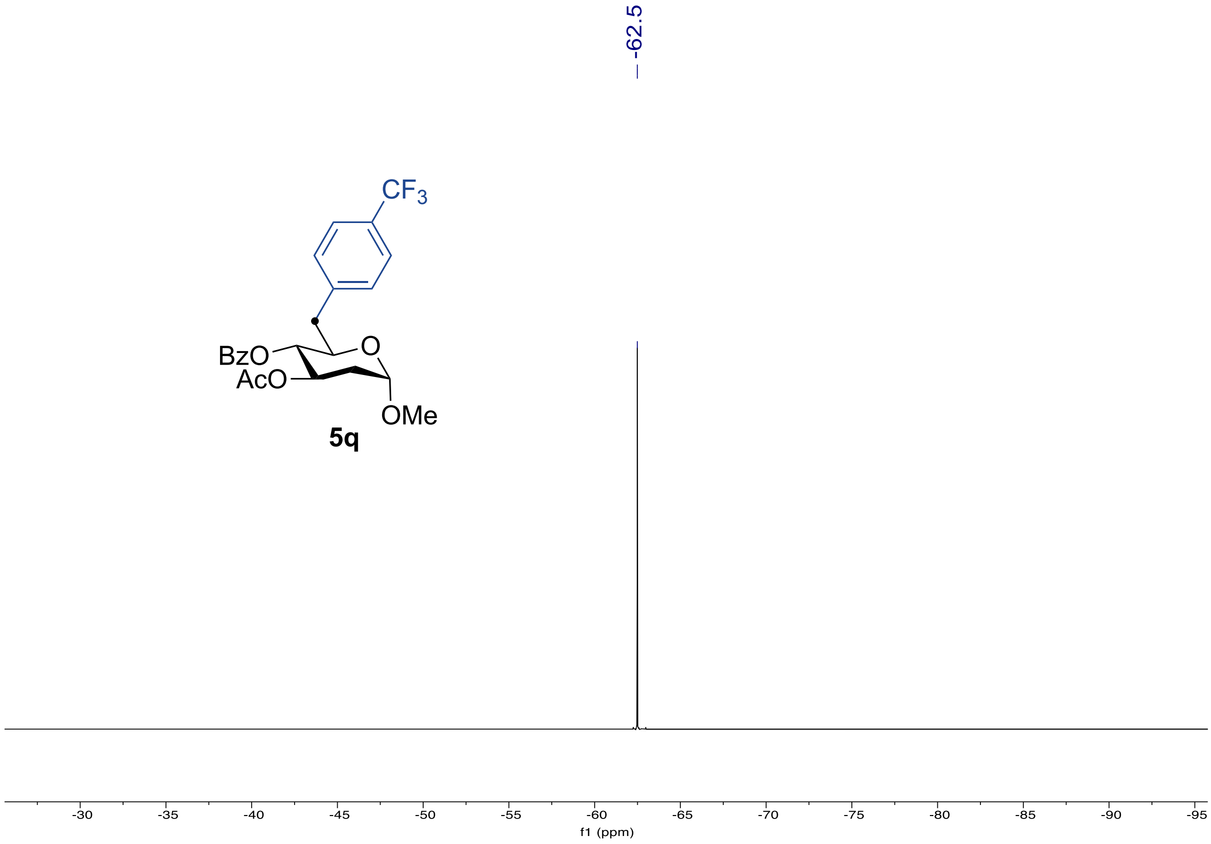

^{19}F NMR (376 MHz, $CDCl_3$) of 5q

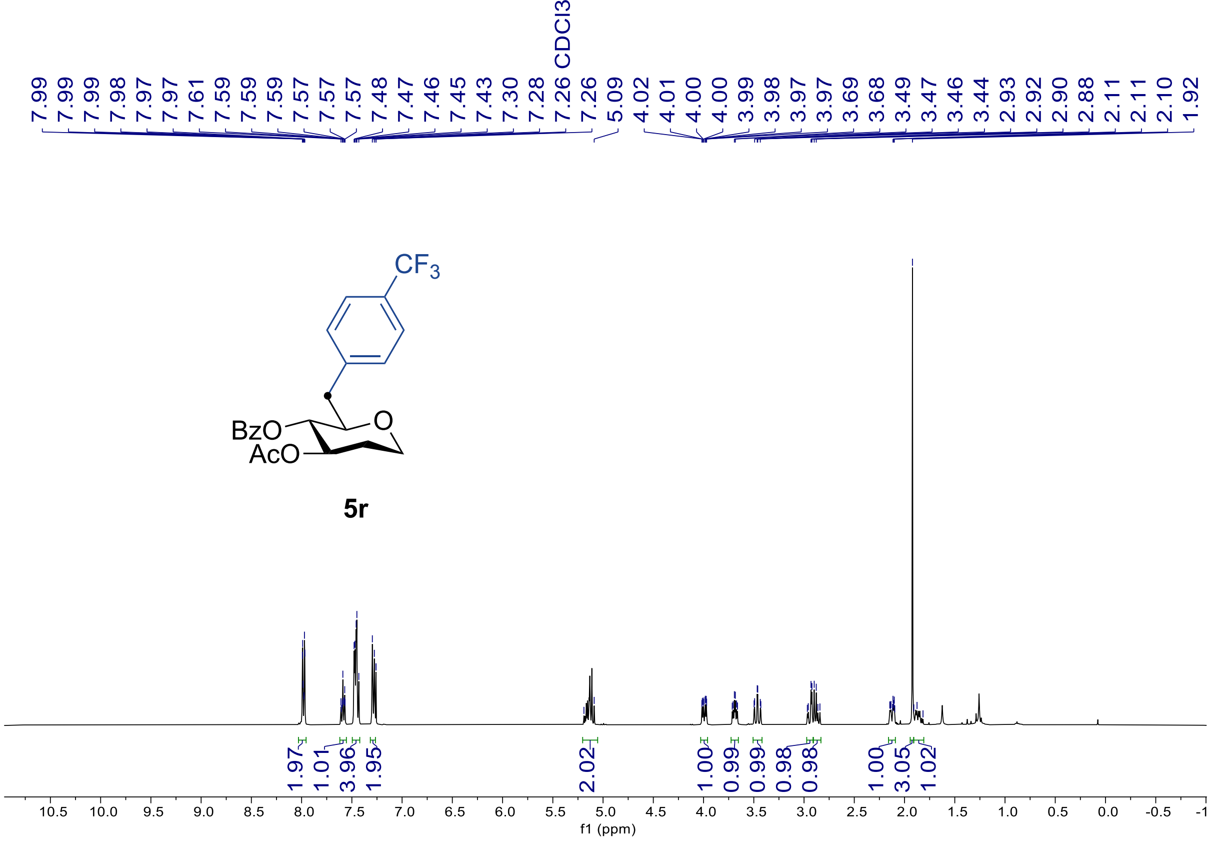

^{1}H NMR (400 MHz, $CDCl_3$) of 5r

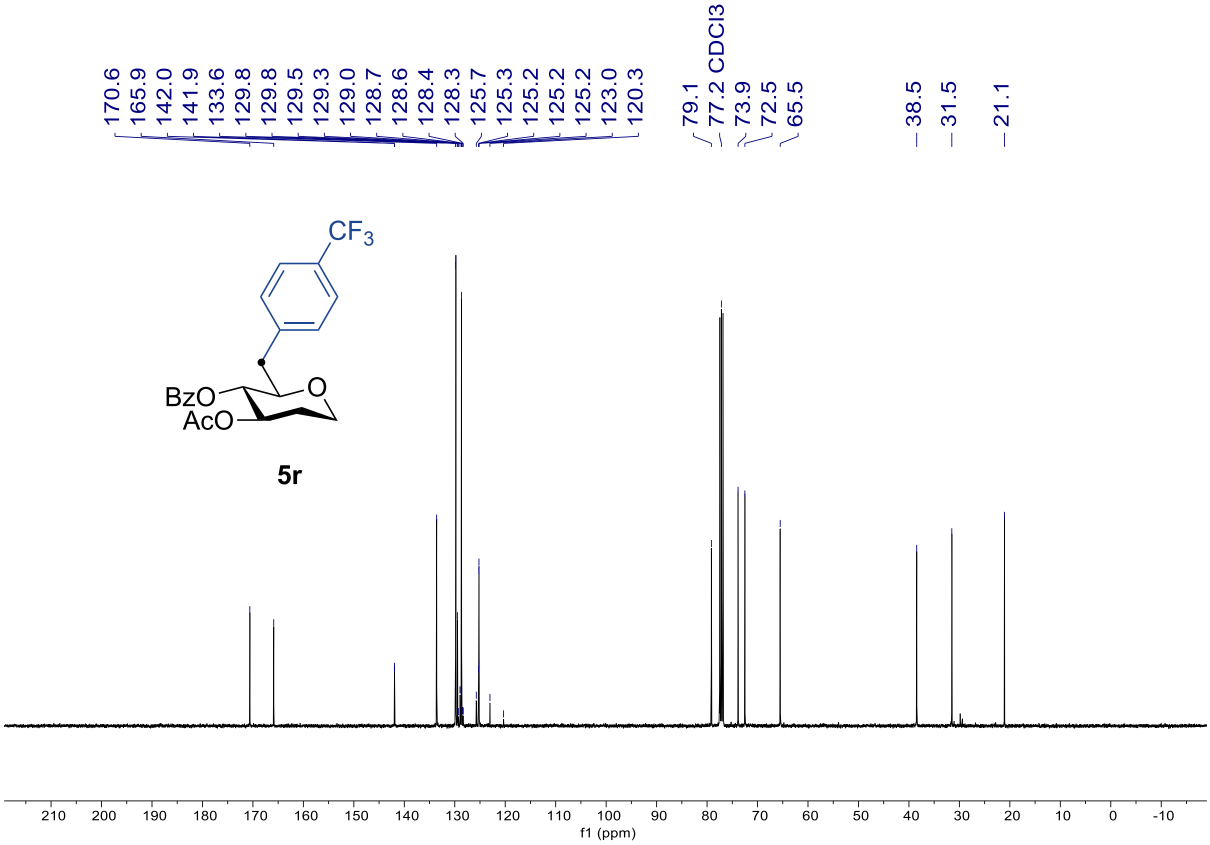

^{13}C NMR (101 MHz, CDCl$_3$) of 5r

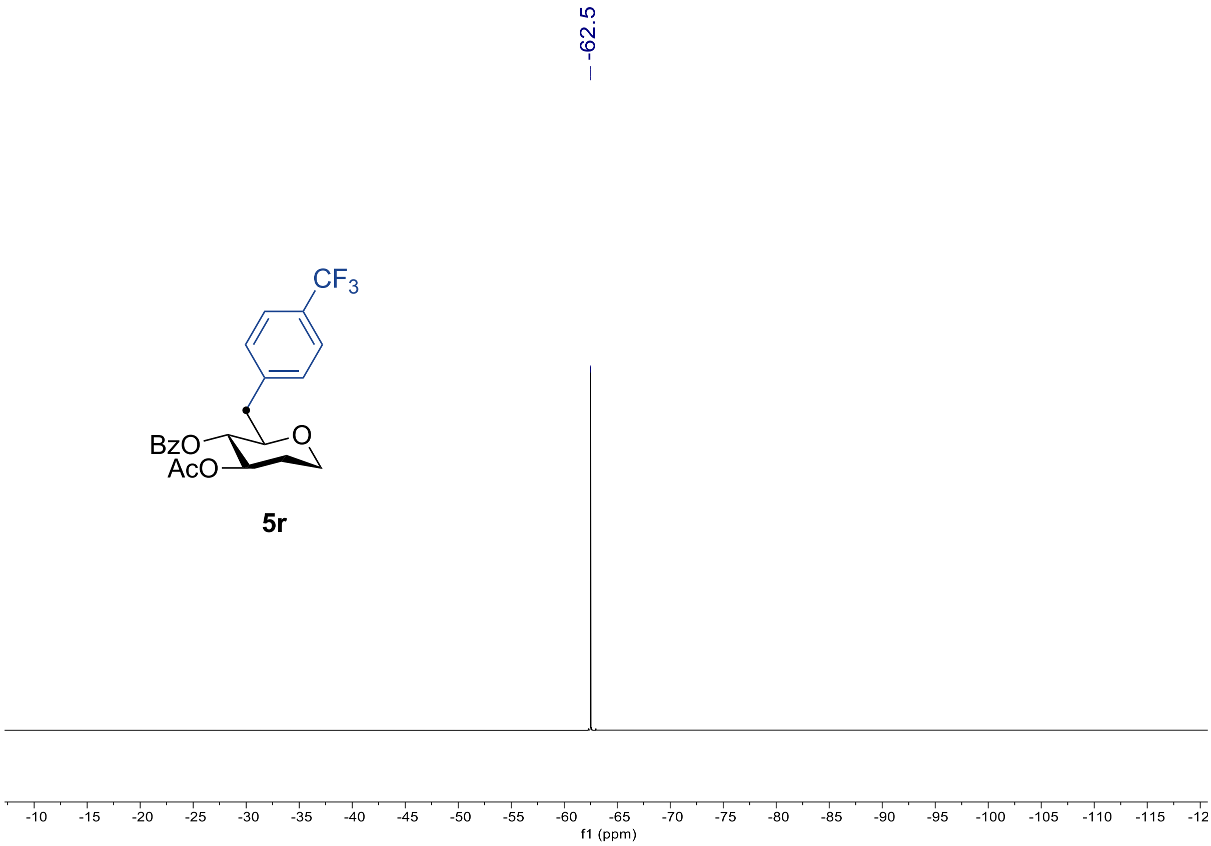

^{19}F NMR (376 MHz, CDCl$_3$) of 5r

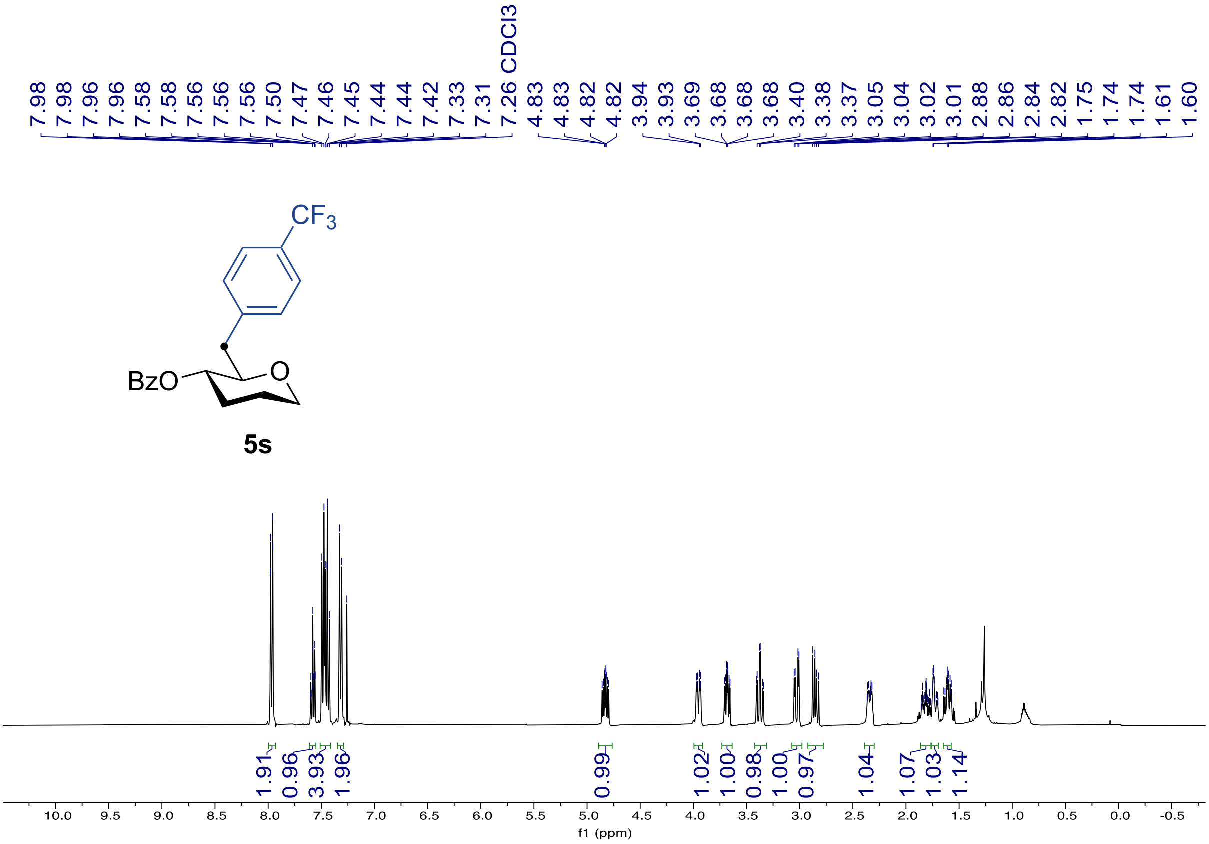

^{1}H NMR (400 MHz, CDCl3) of 5s

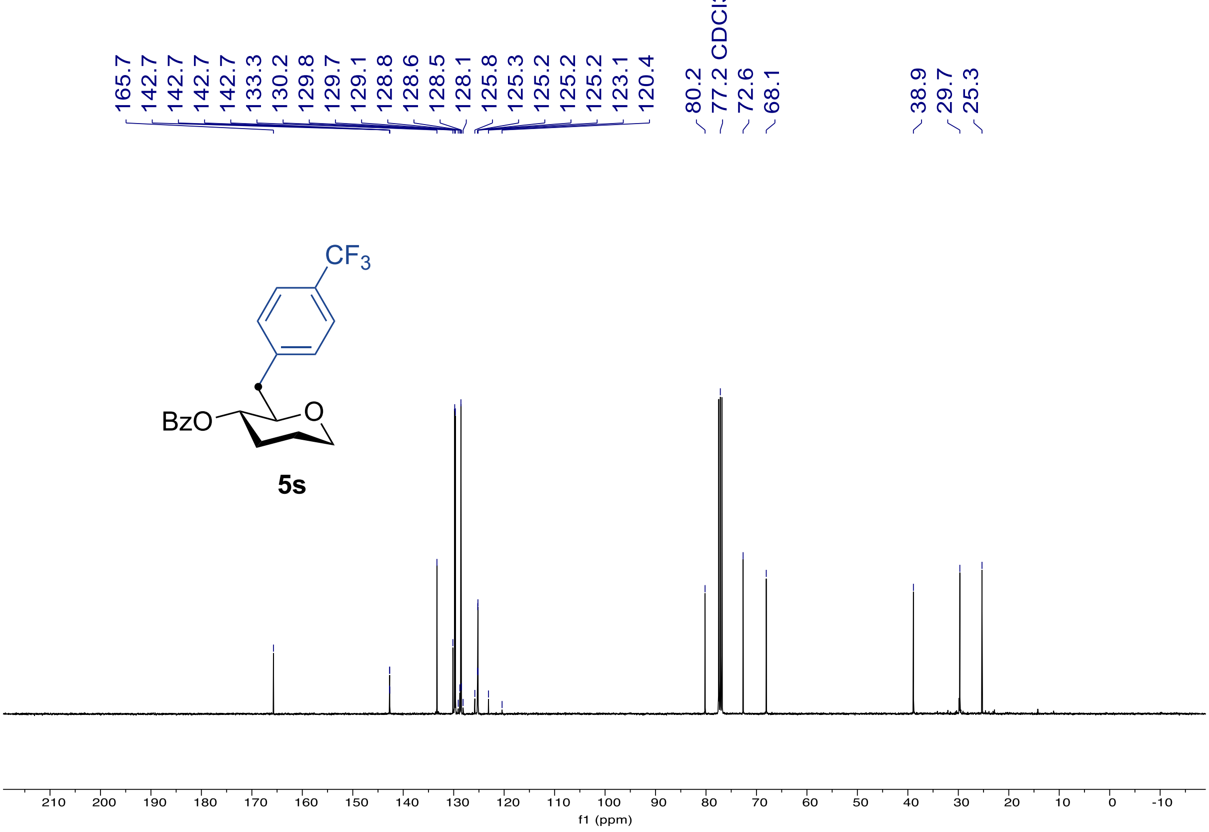

^{13}C NMR (101 MHz, CDCl3) of 5s

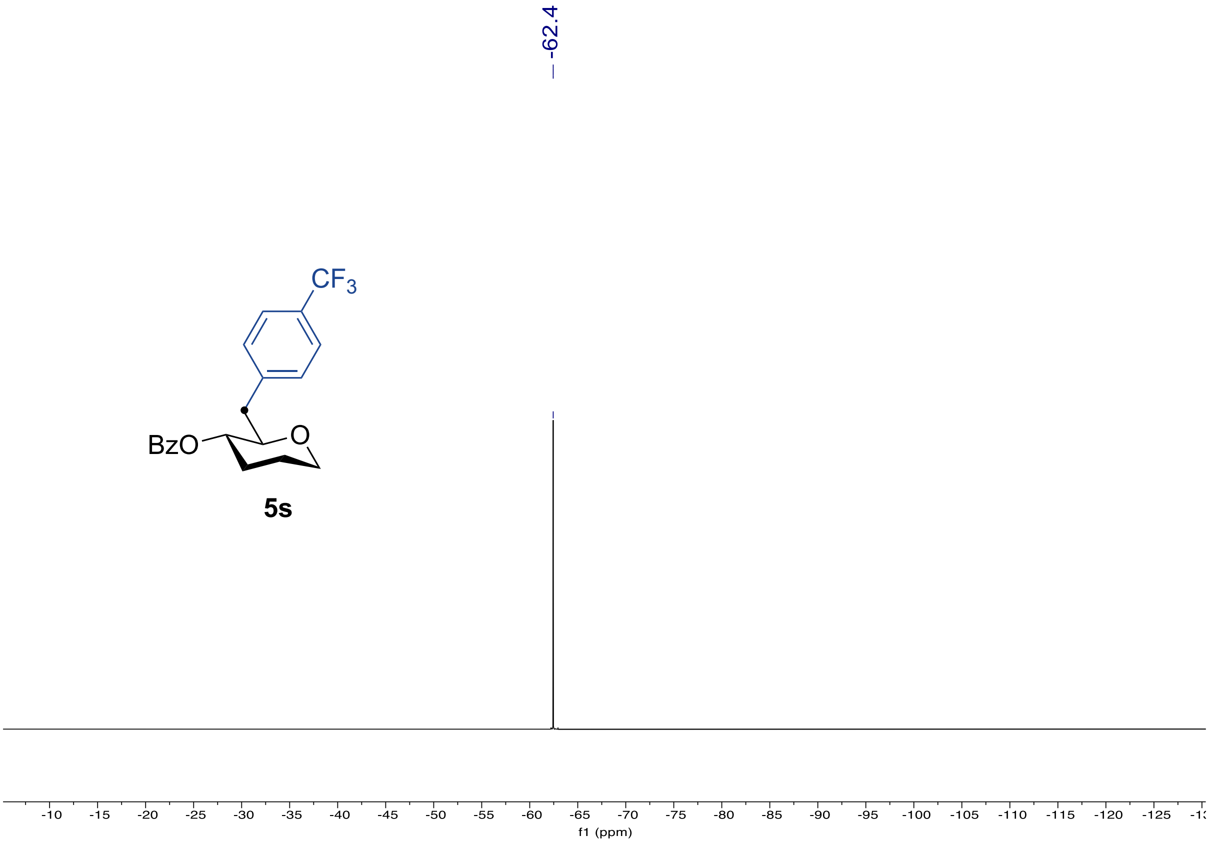

^{19}F NMR (376 MHz, $CDCl_3$) of 5s

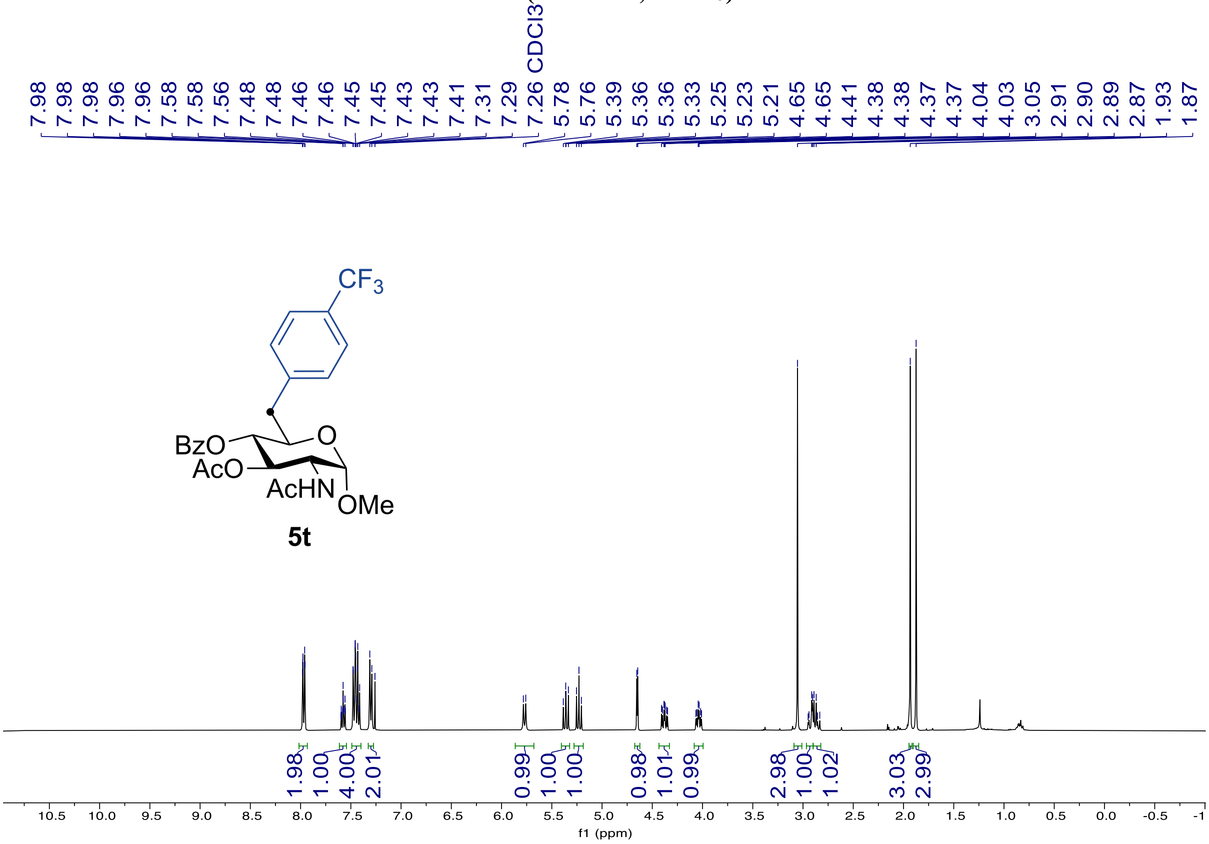

^{1}H NMR (400 MHz, $CDCl_3$) of 5t

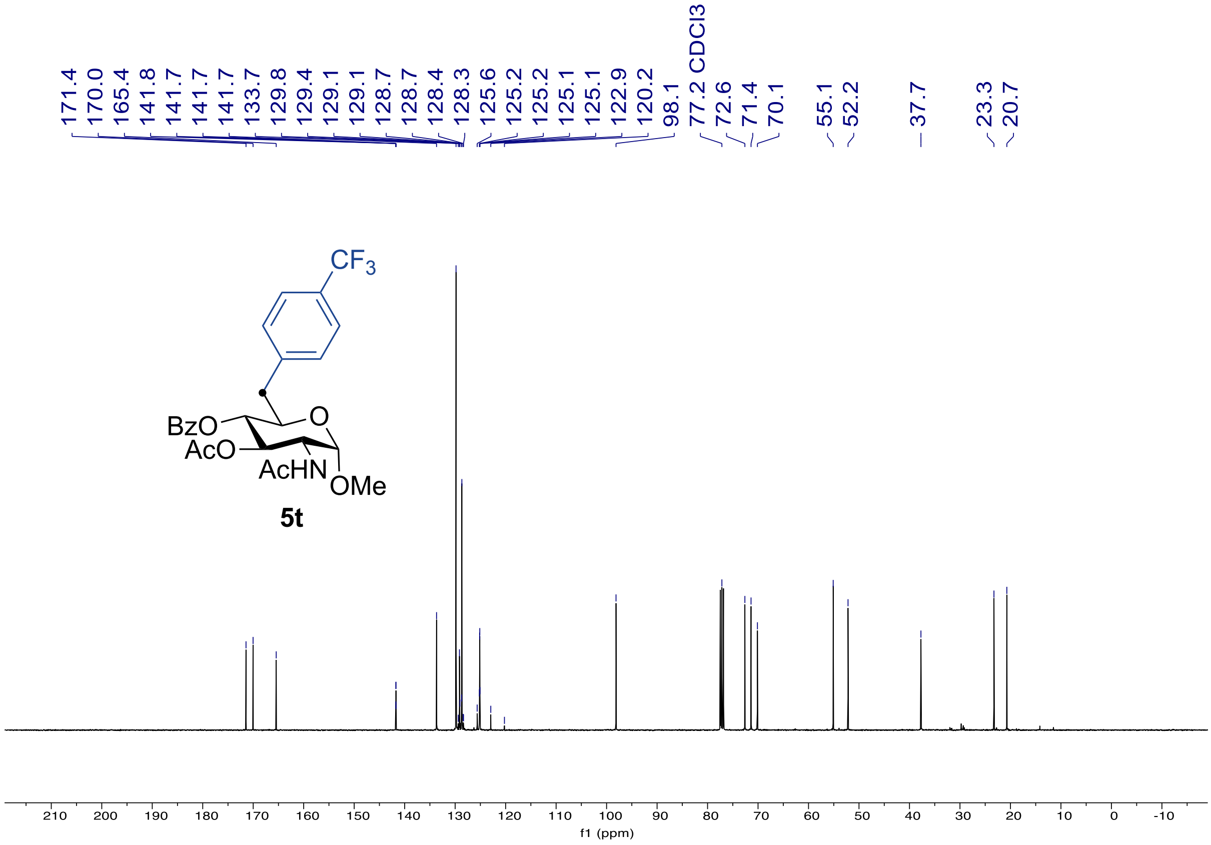

^{13}C NMR (101 MHz, $CDCl_3$) of 5t

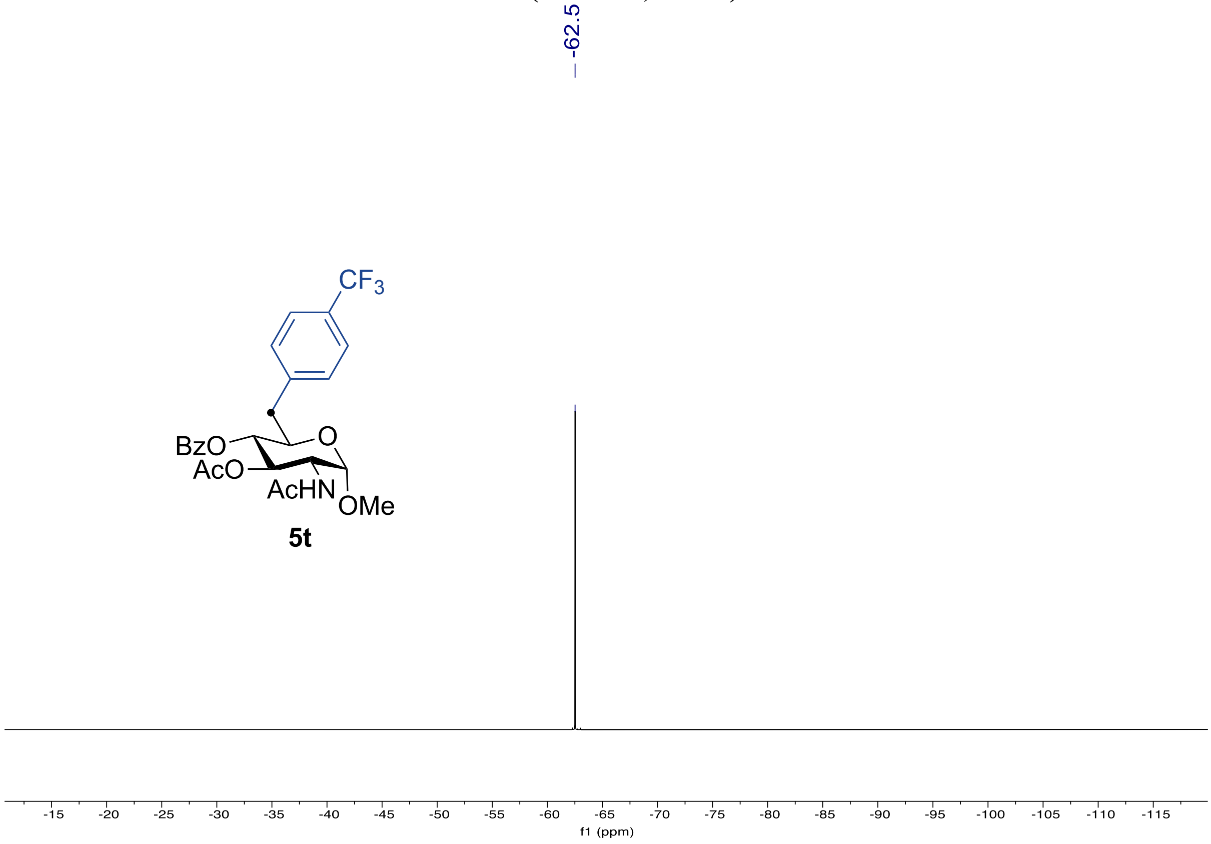

^{19}F NMR (376 MHz, $CDCl_3$) of 5t

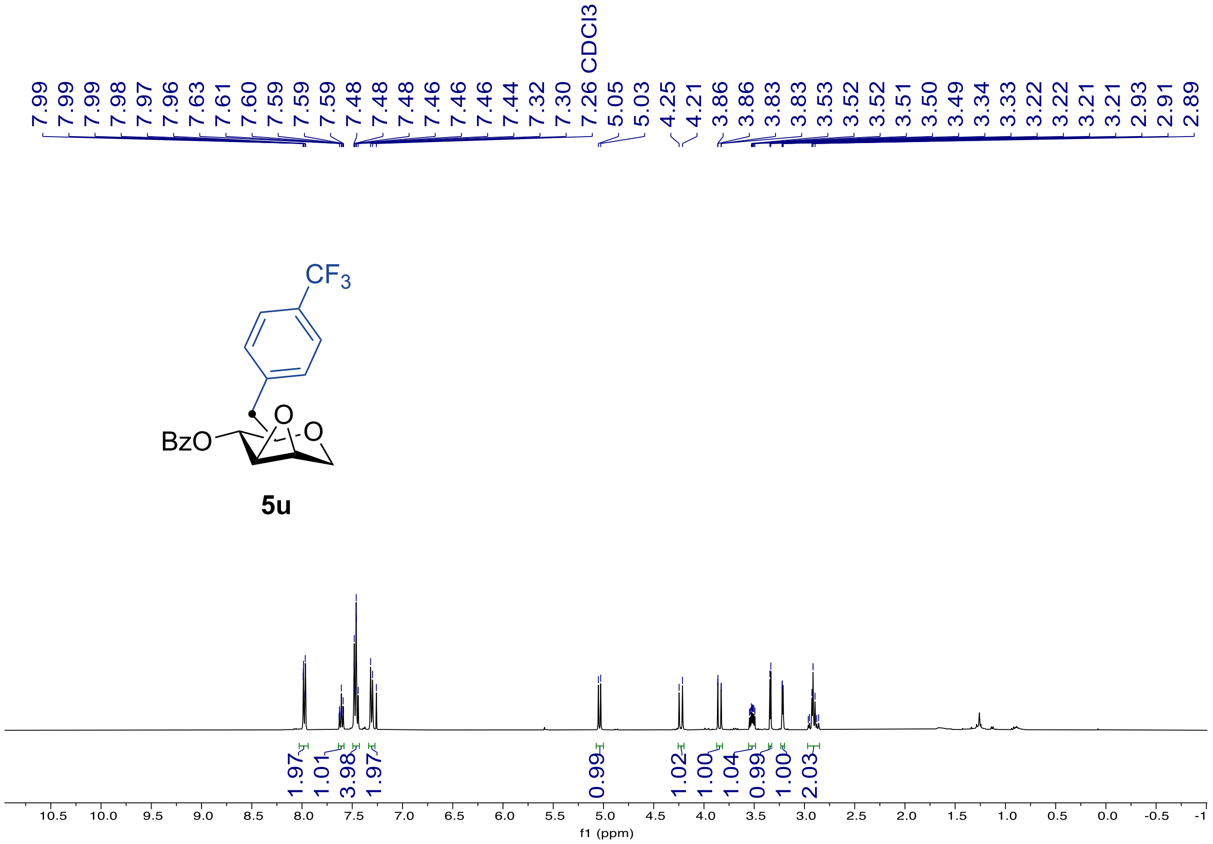

^{1}H NMR (400 MHz, CDCl₃) of 5u

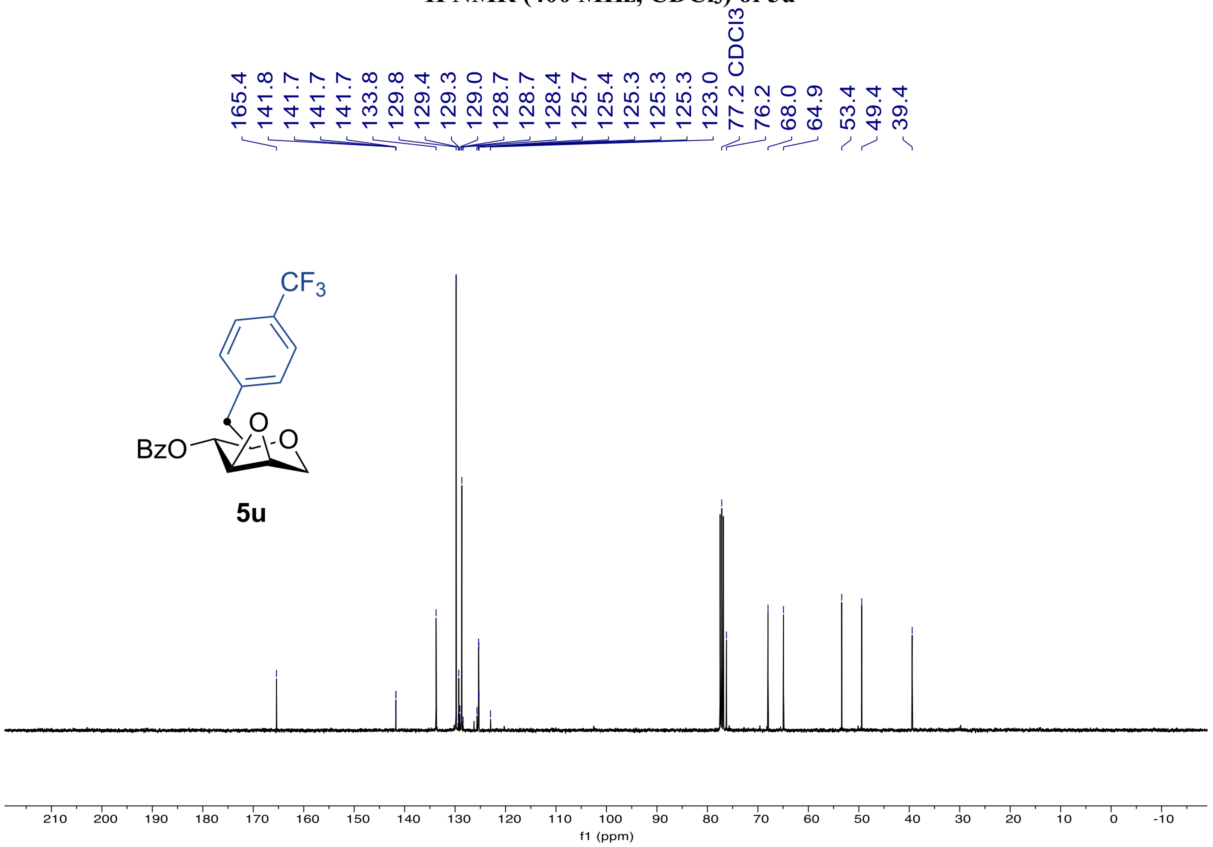

^{13}C NMR (101 MHz, CDCl₃) of 5u

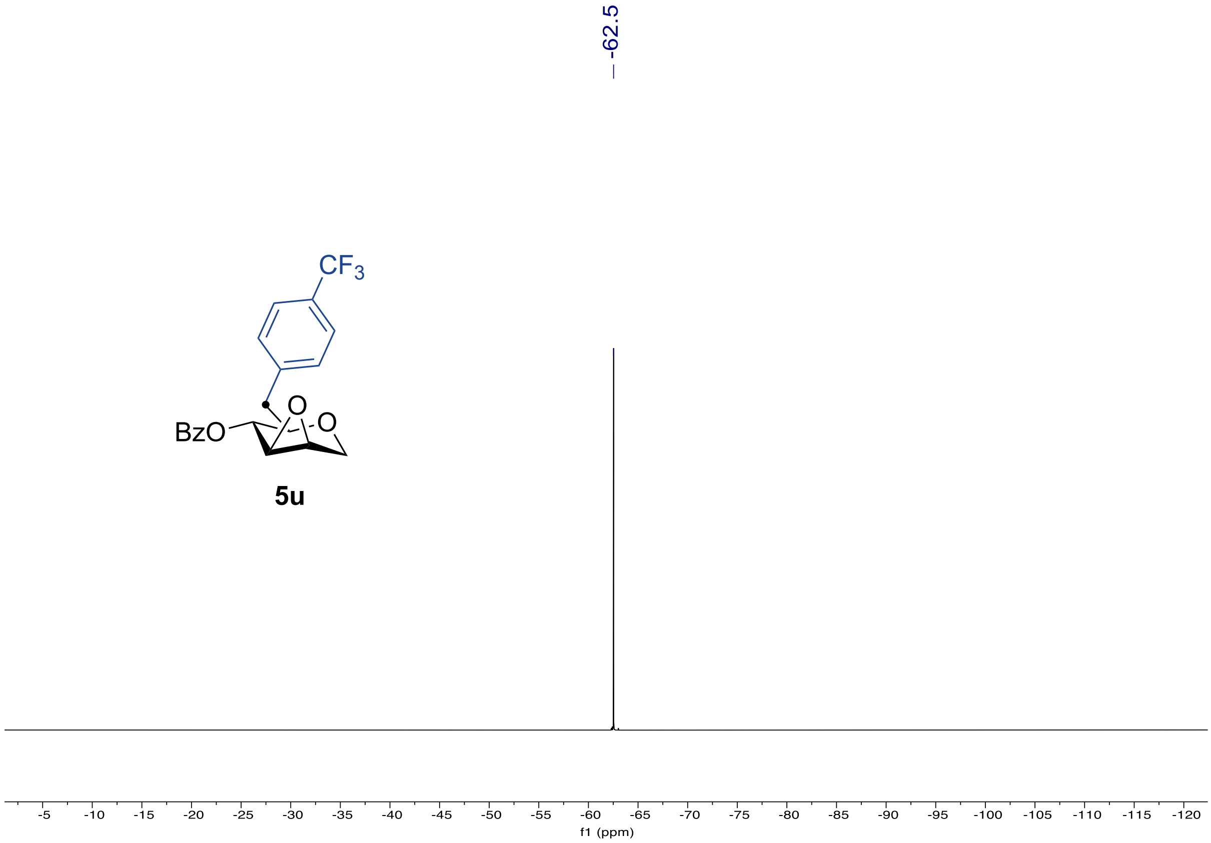

^{19}F NMR (376 MHz, $CDCl_3$) of 5u

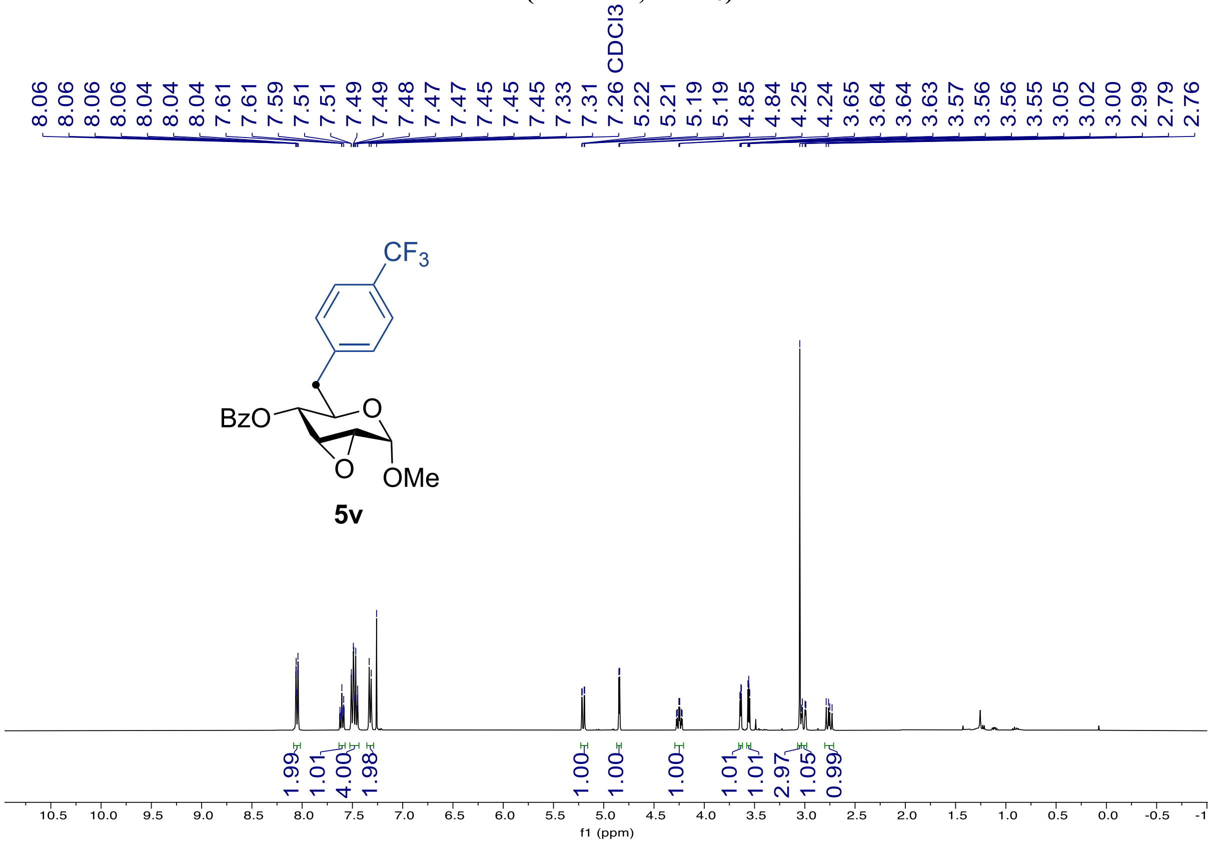

^{1}H NMR (400 MHz, $CDCl_3$) of 5v

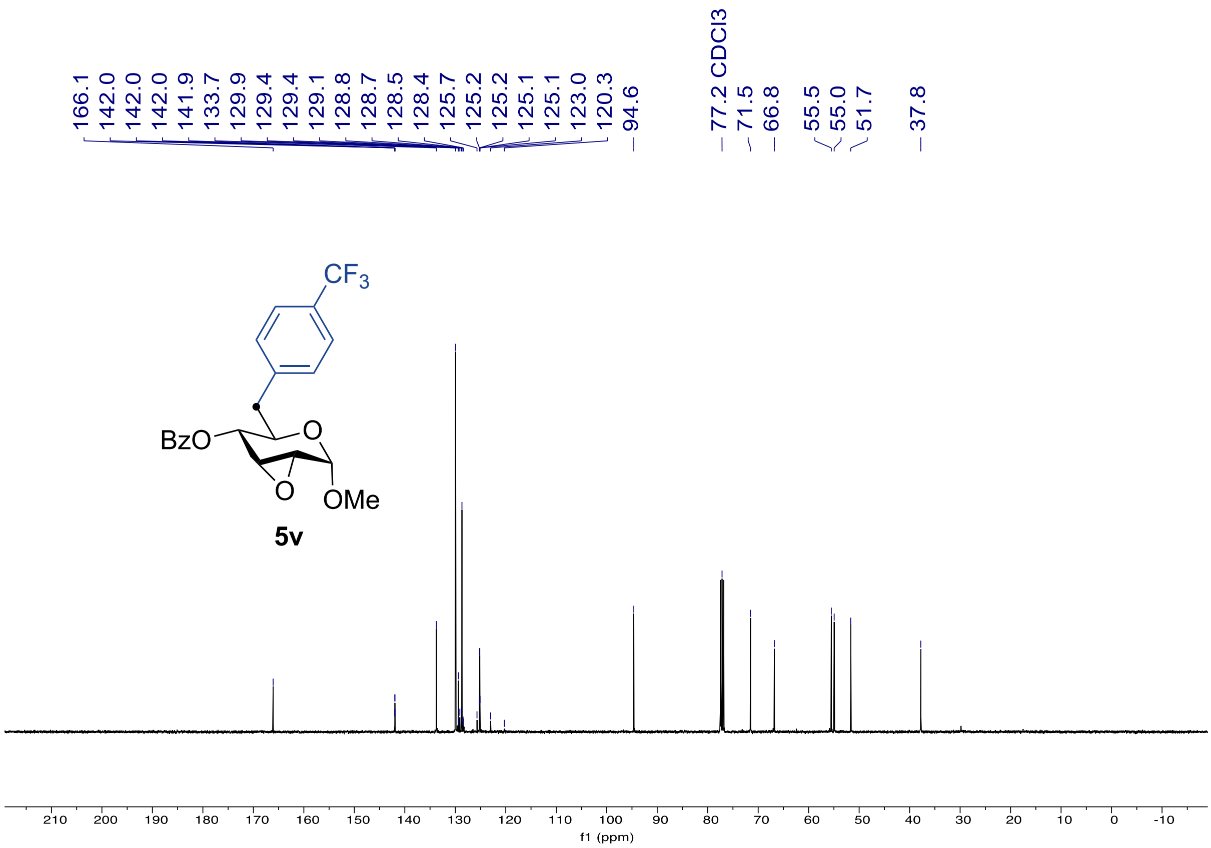

^{13}C NMR (101 MHz, $CDCl_3$) of 5v

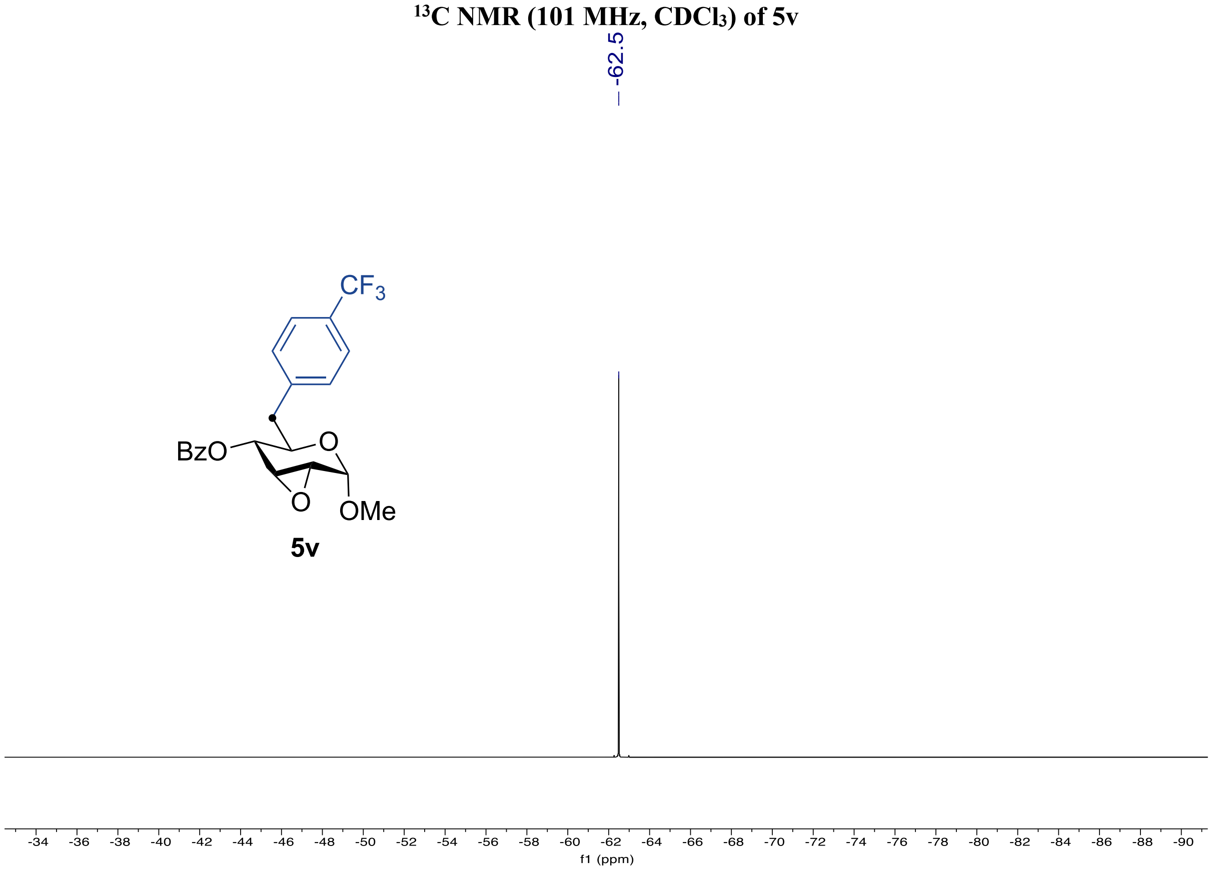

^{19}F NMR (376 MHz, $CDCl_3$) of 5v

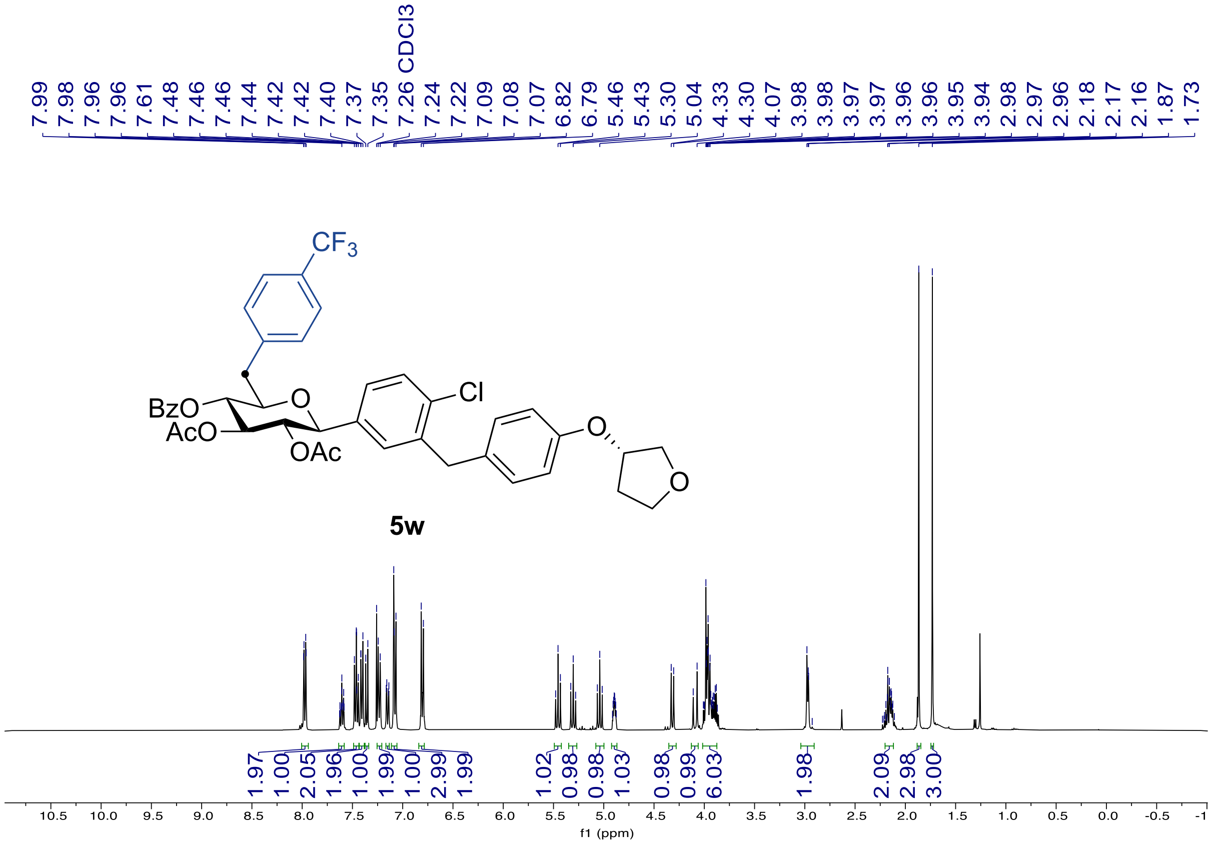

^{1}H NMR (400 MHz, CDCl₃) of 5w

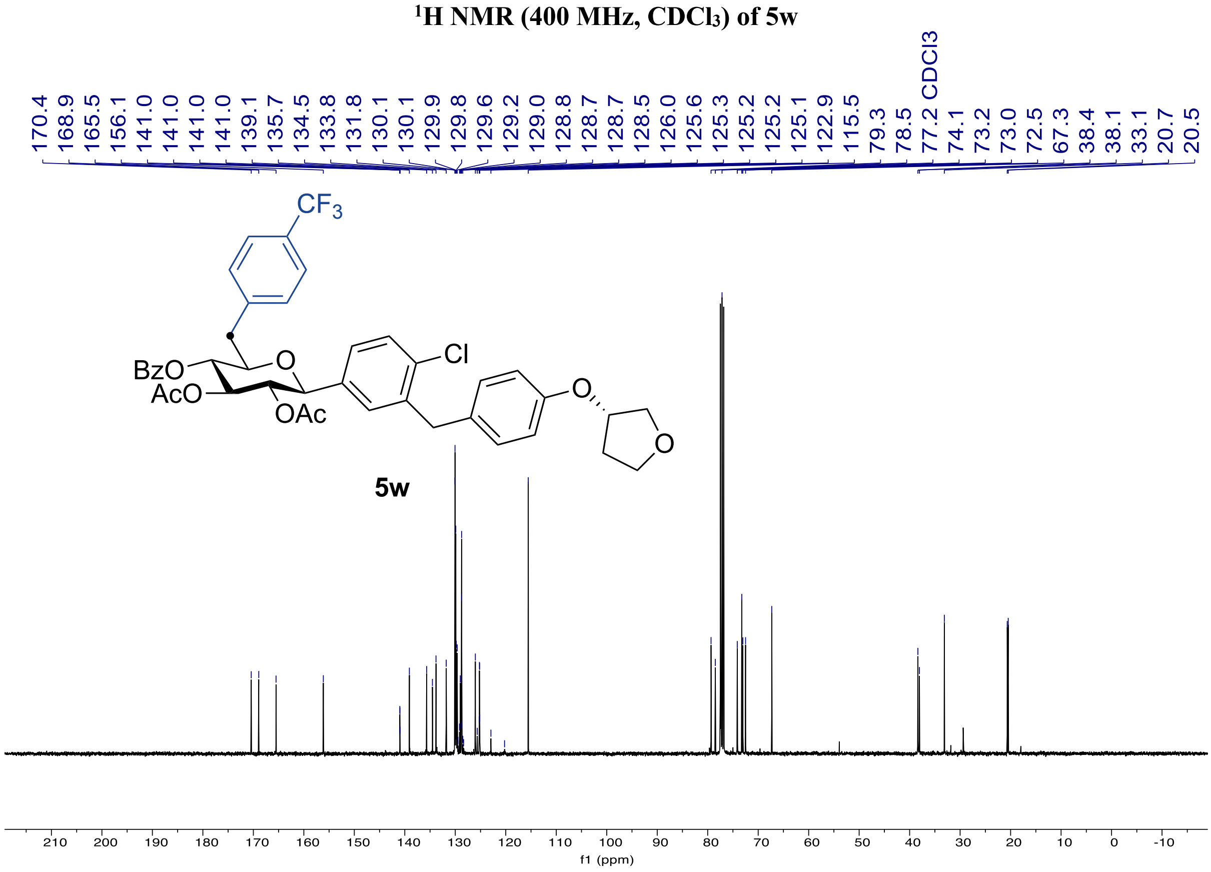

^{13}C NMR (101 MHz, CDCl₃) of 5w

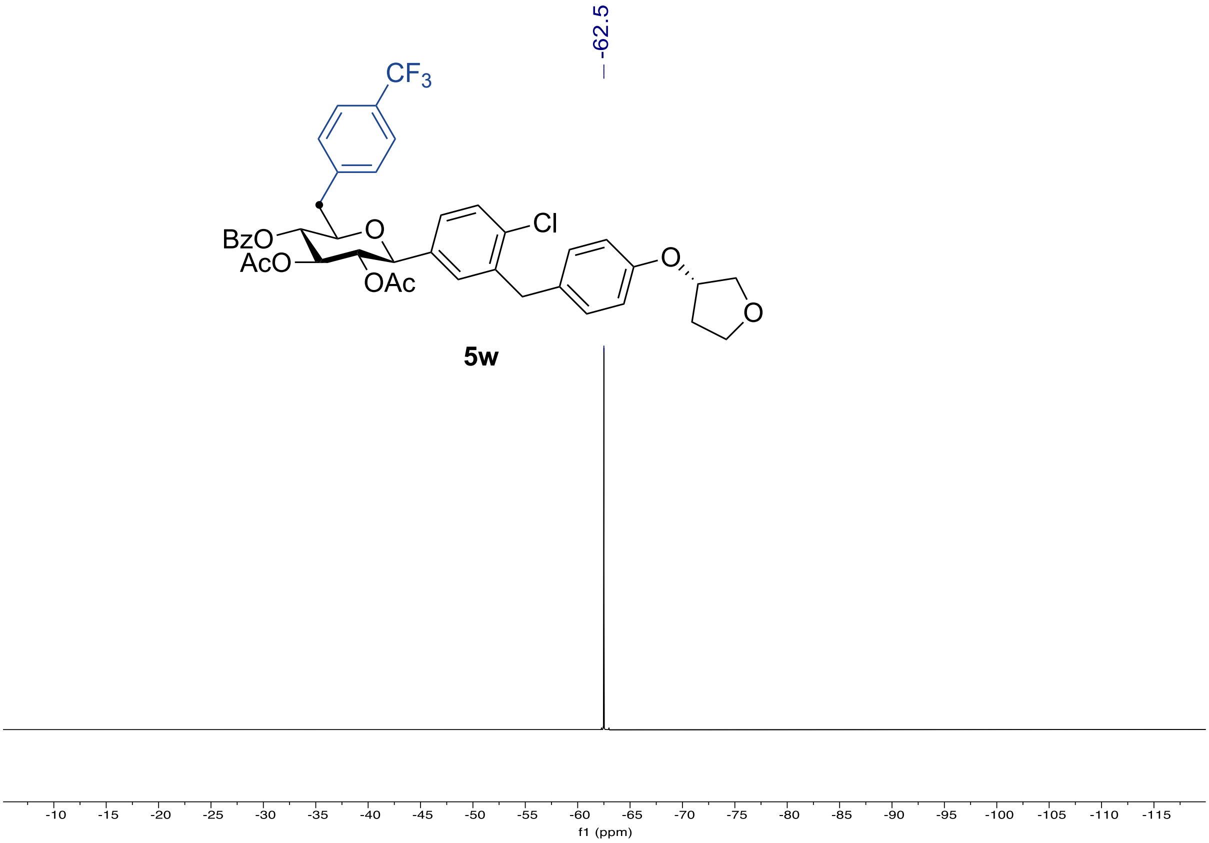

^{19}F NMR (376 MHz, CDCl₃) of 5w

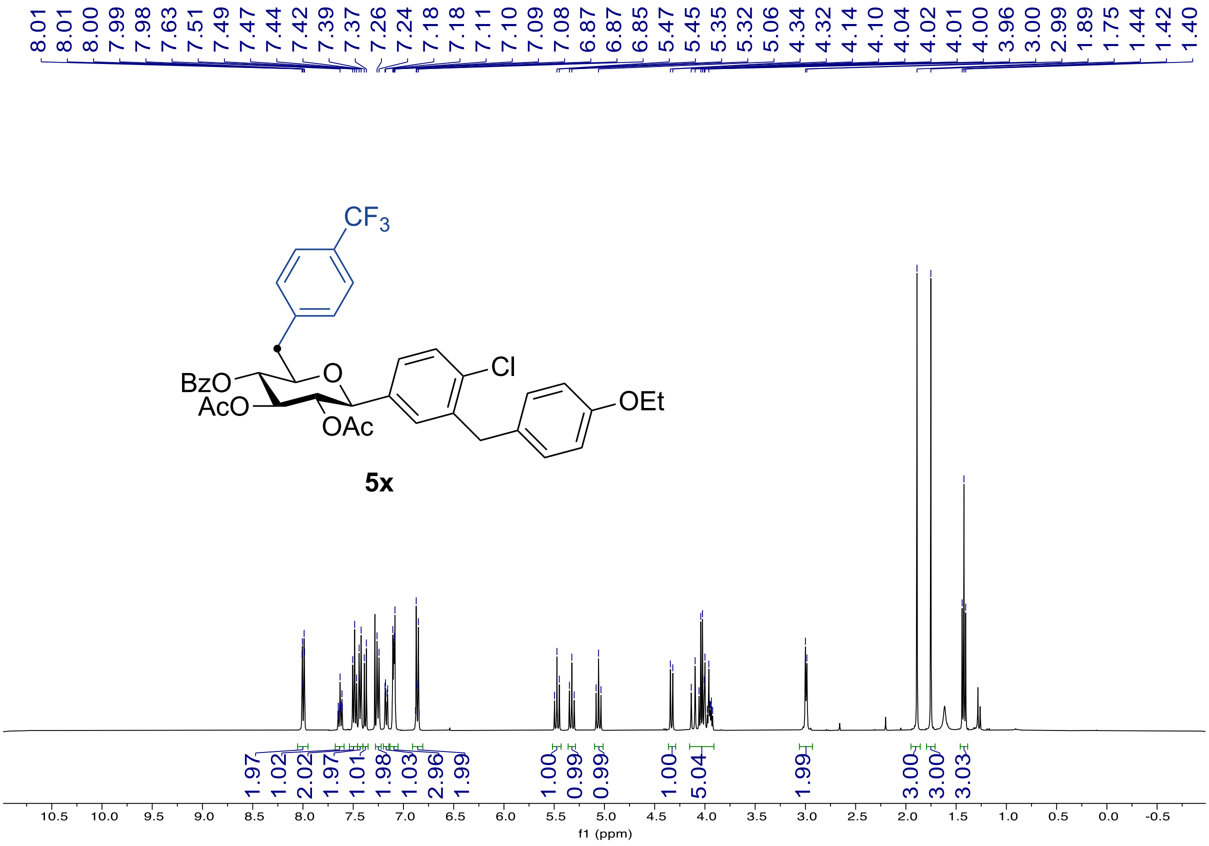

^{1}H NMR (400 MHz, CDCl₃) of 5x

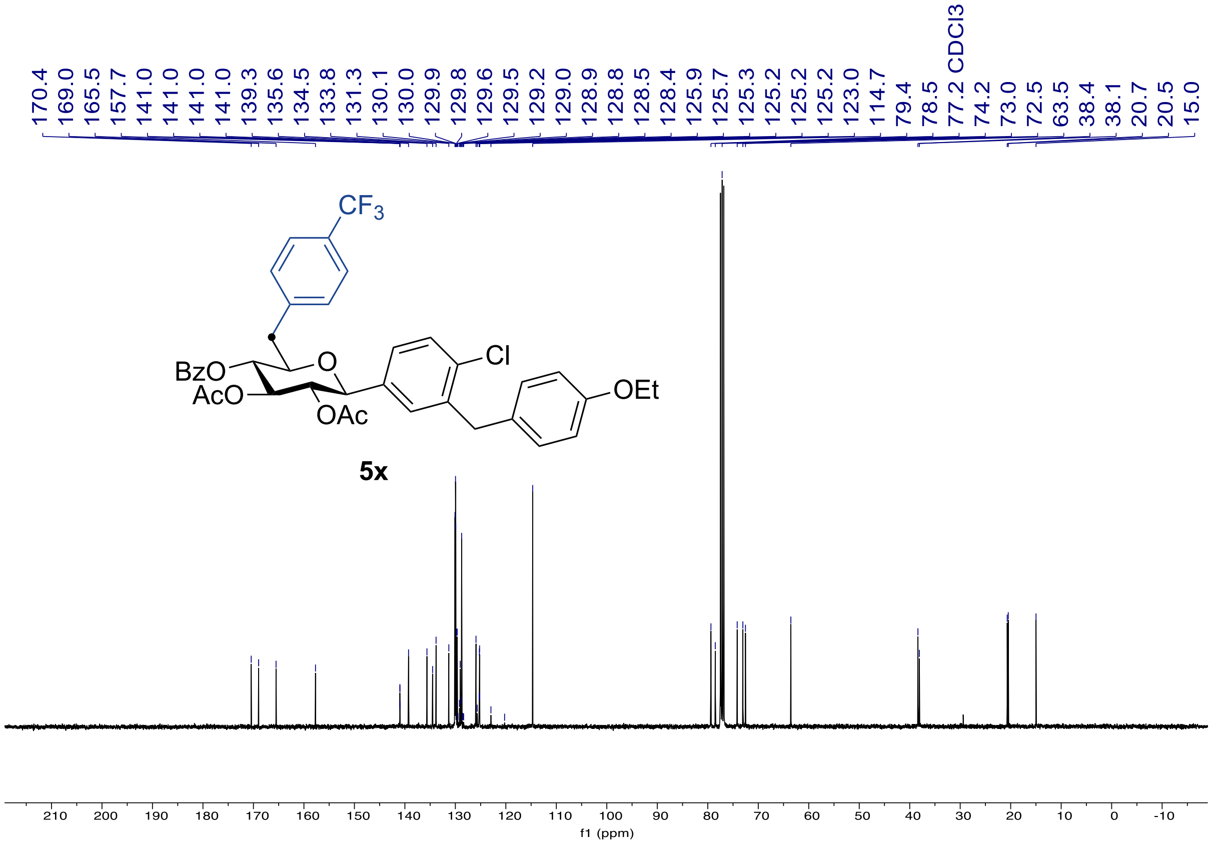

^{13}C NMR (101 MHz, CDCl$_3$) of 5x

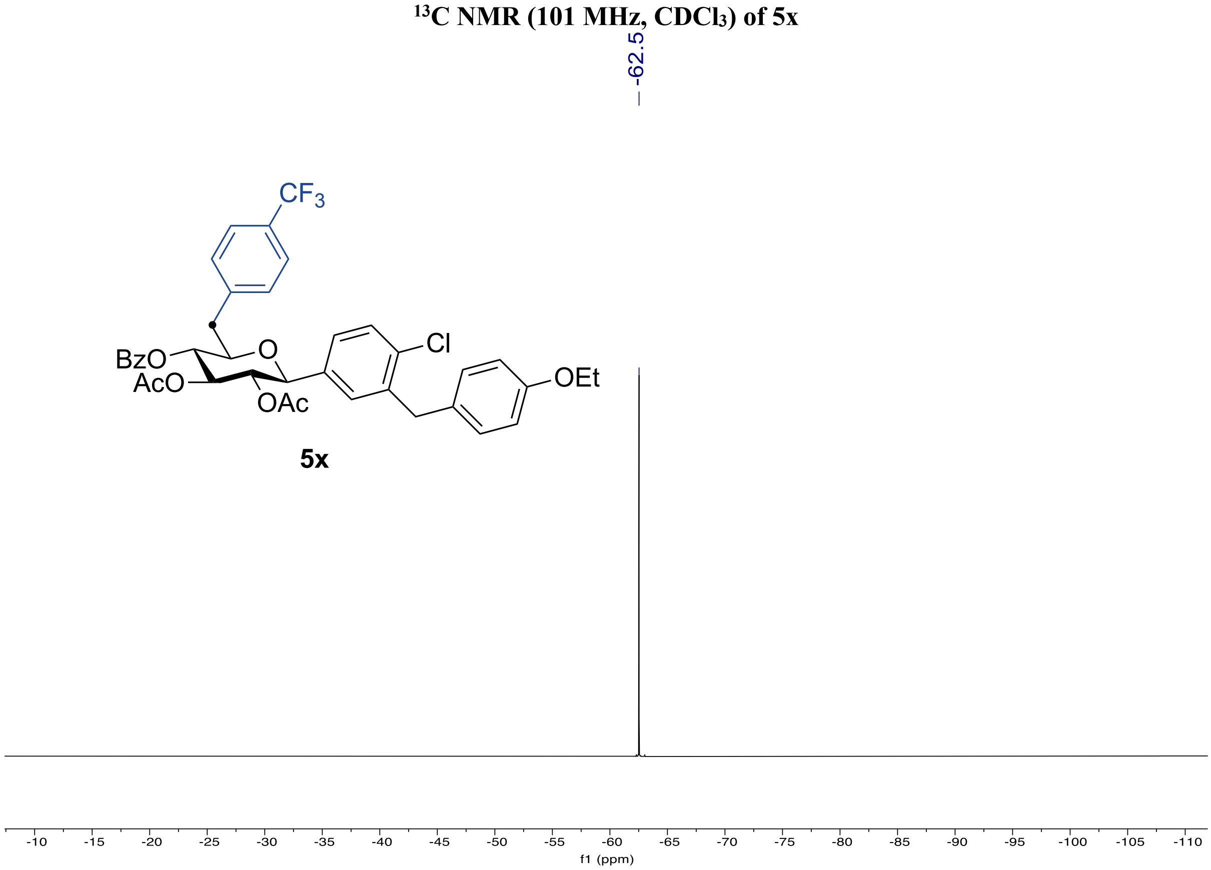

^{19}F NMR (376 MHz, CDCl$_3$) of 5x

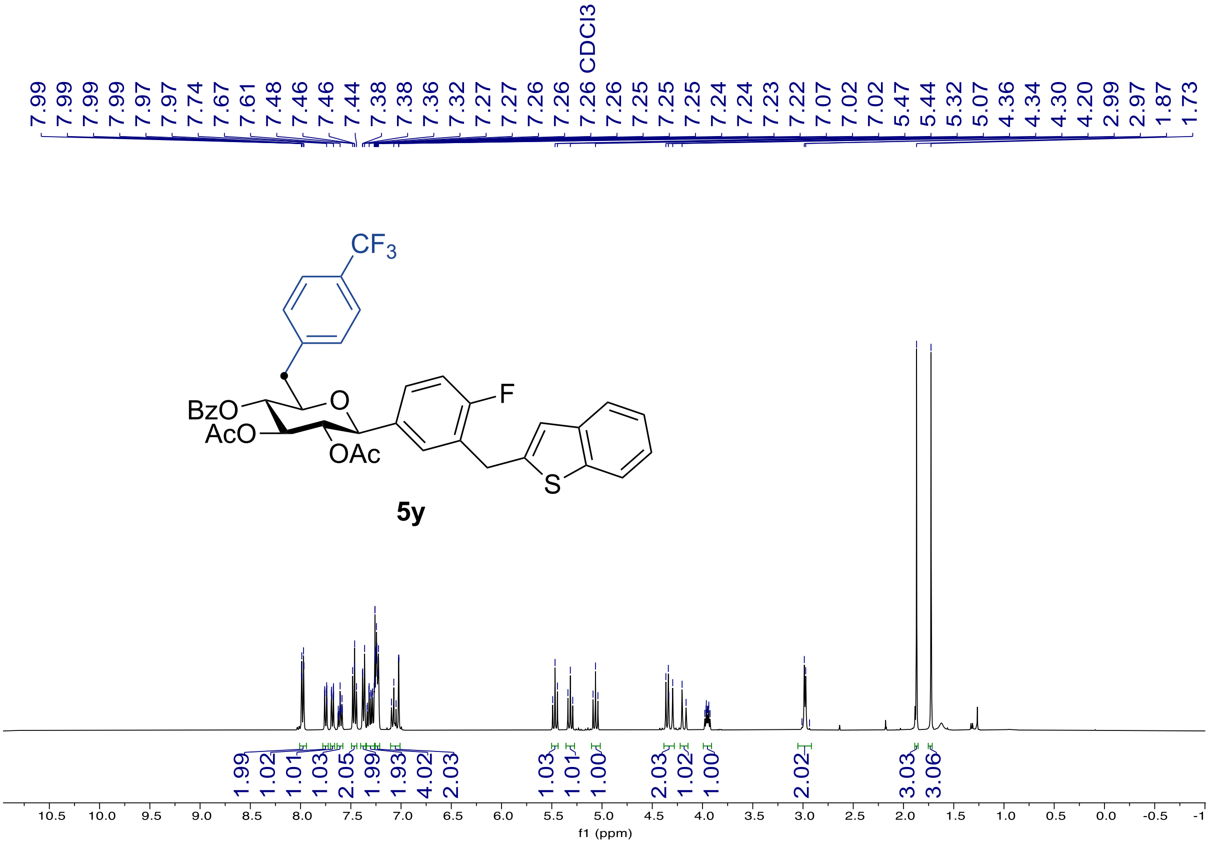

^{1}H NMR (400 MHz, CDCl3) of 5y

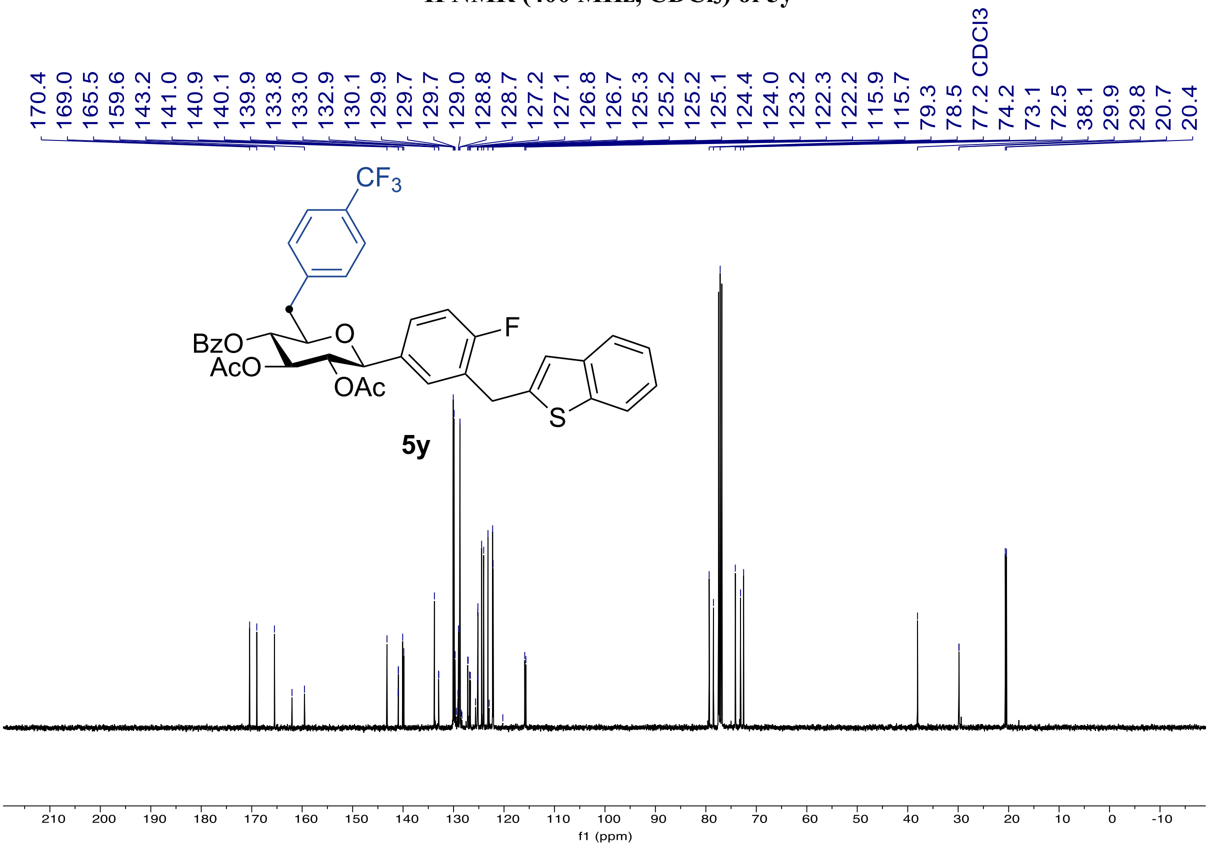

^{13}C NMR (101 MHz, CDCl3) of 5y

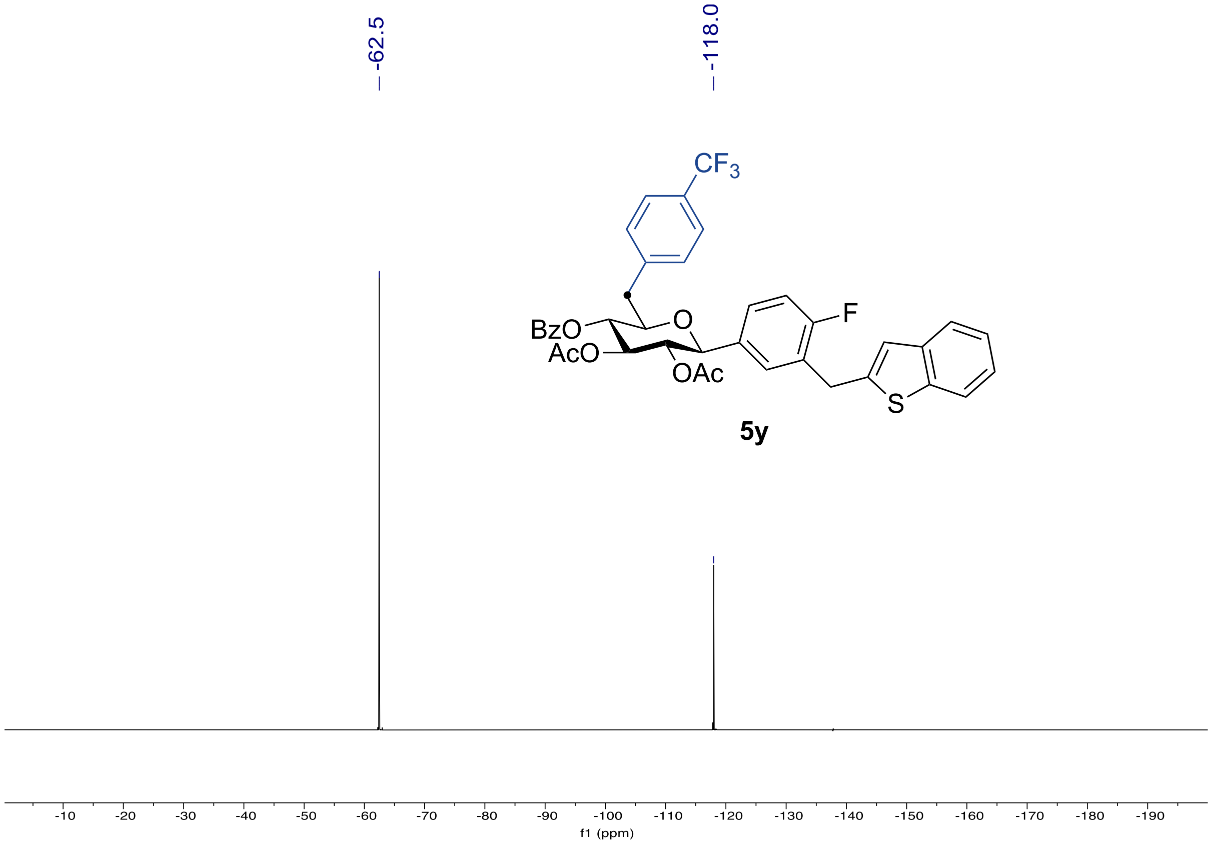

^{19}F NMR (376 MHz, $CDCl_3$) of 5y

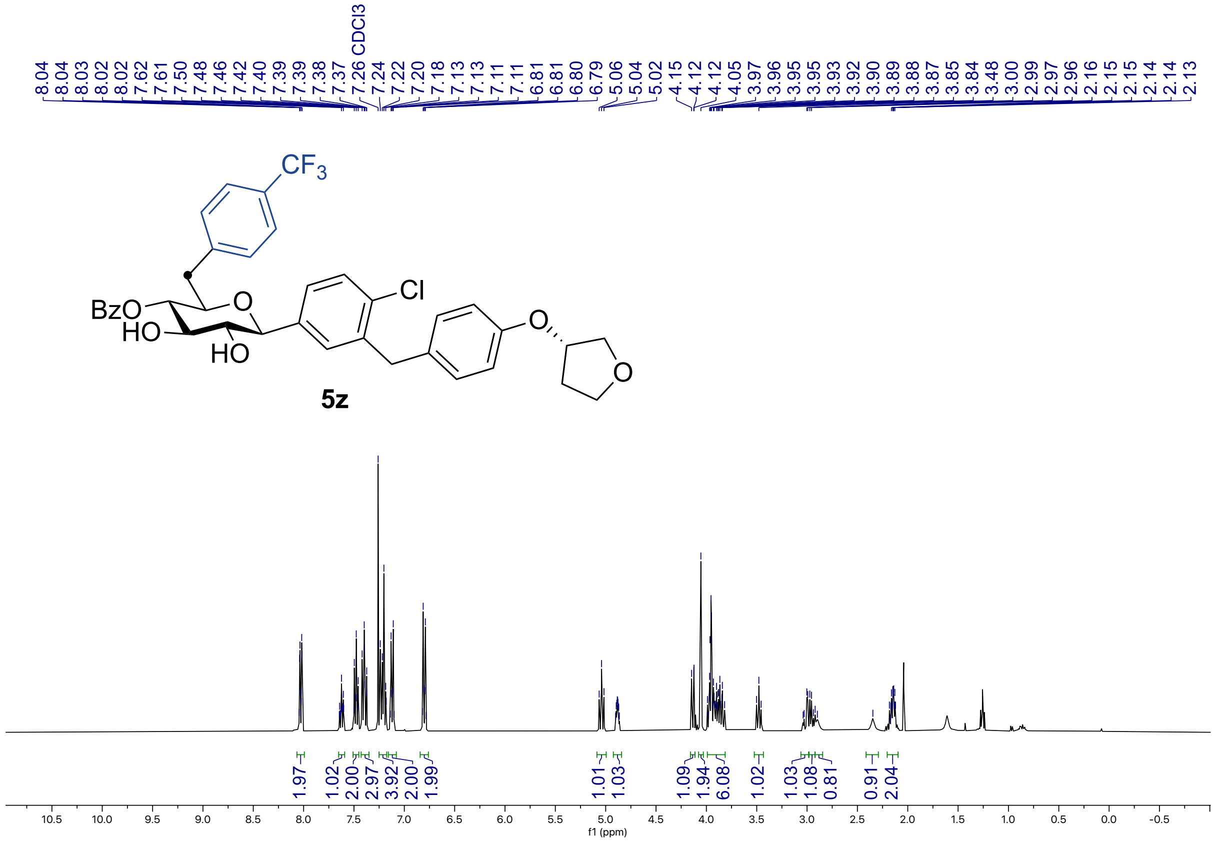

^{1}H NMR (400 MHz, CDCl₃) of 5z

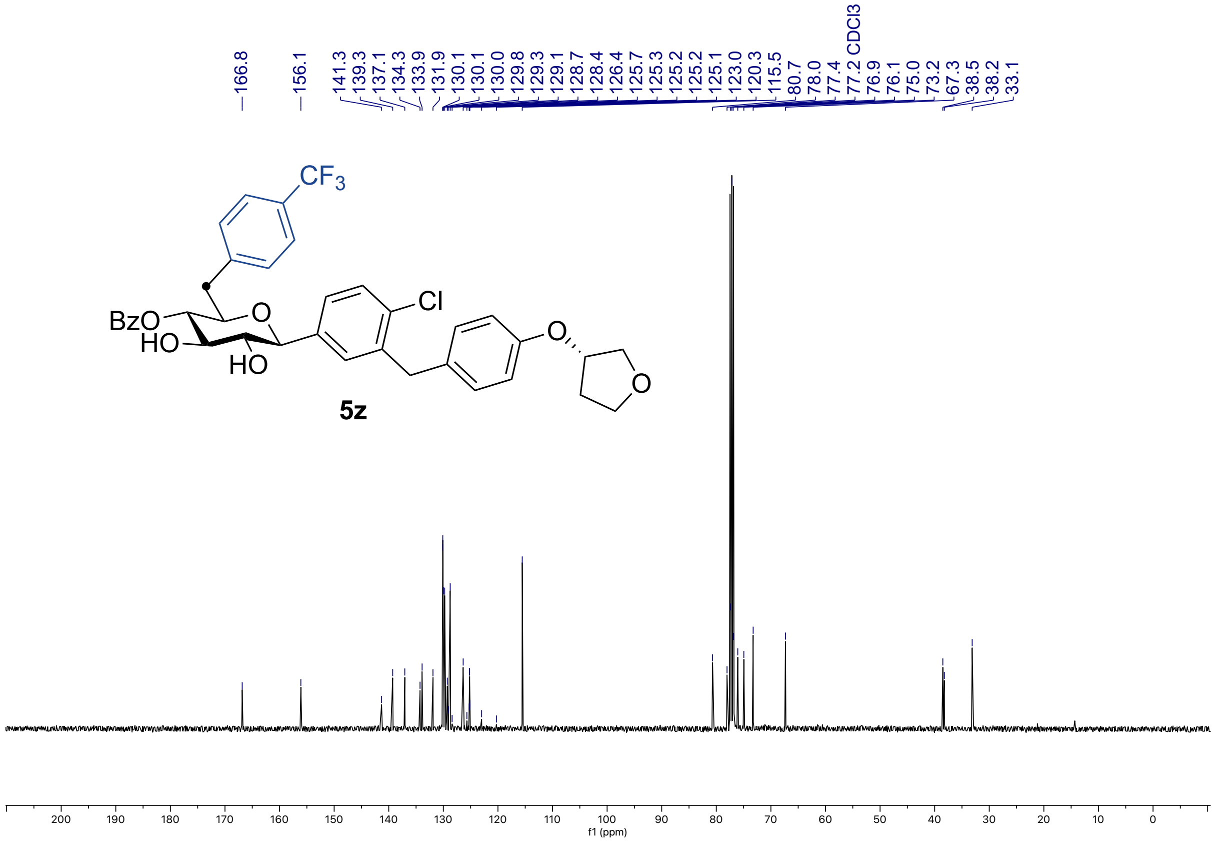

^{13}C NMR (101 MHz, CDCl₃) of 5z

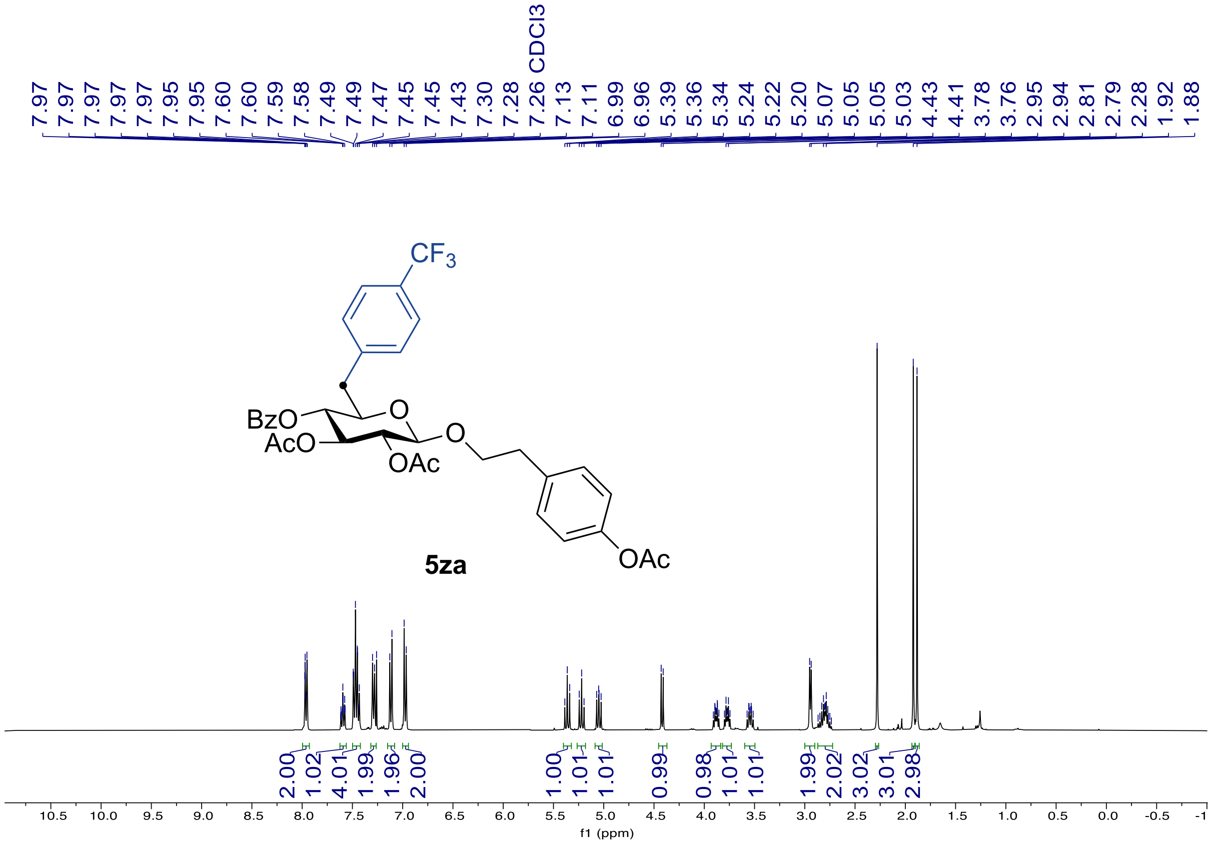

^{1}H NMR (400 MHz, CDCl₃) of 5za

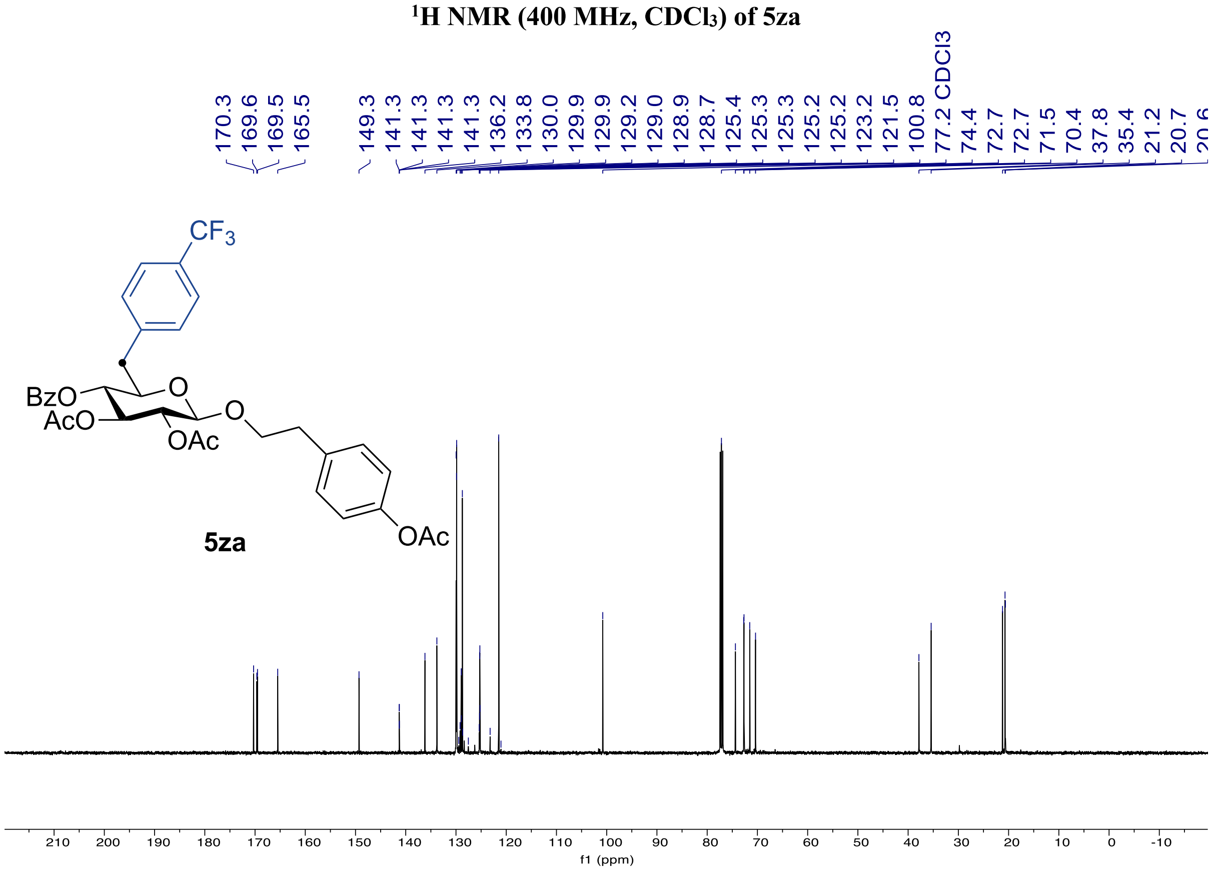

^{13}C NMR (126 MHz, CDCl₃) of 5za

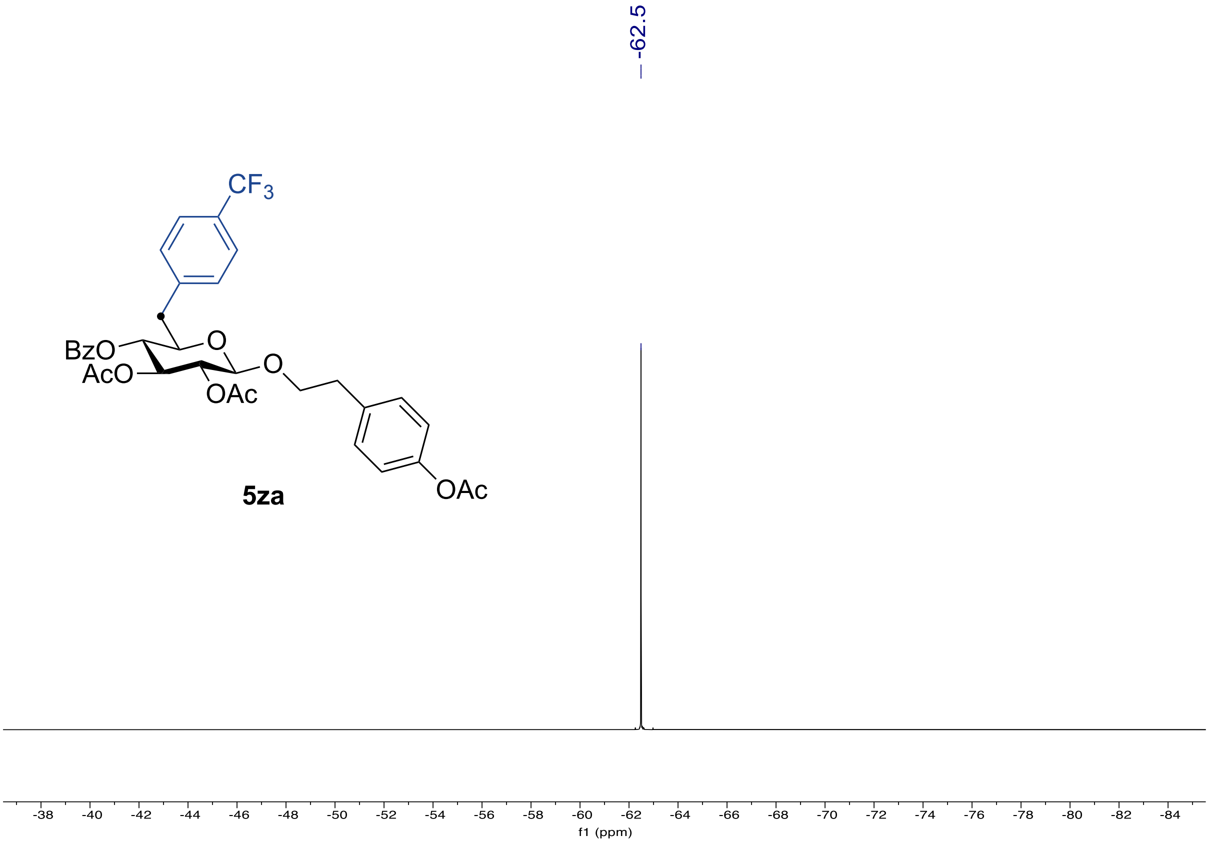

^{19}F NMR (376 MHz, CDCl$_3$) of 5za

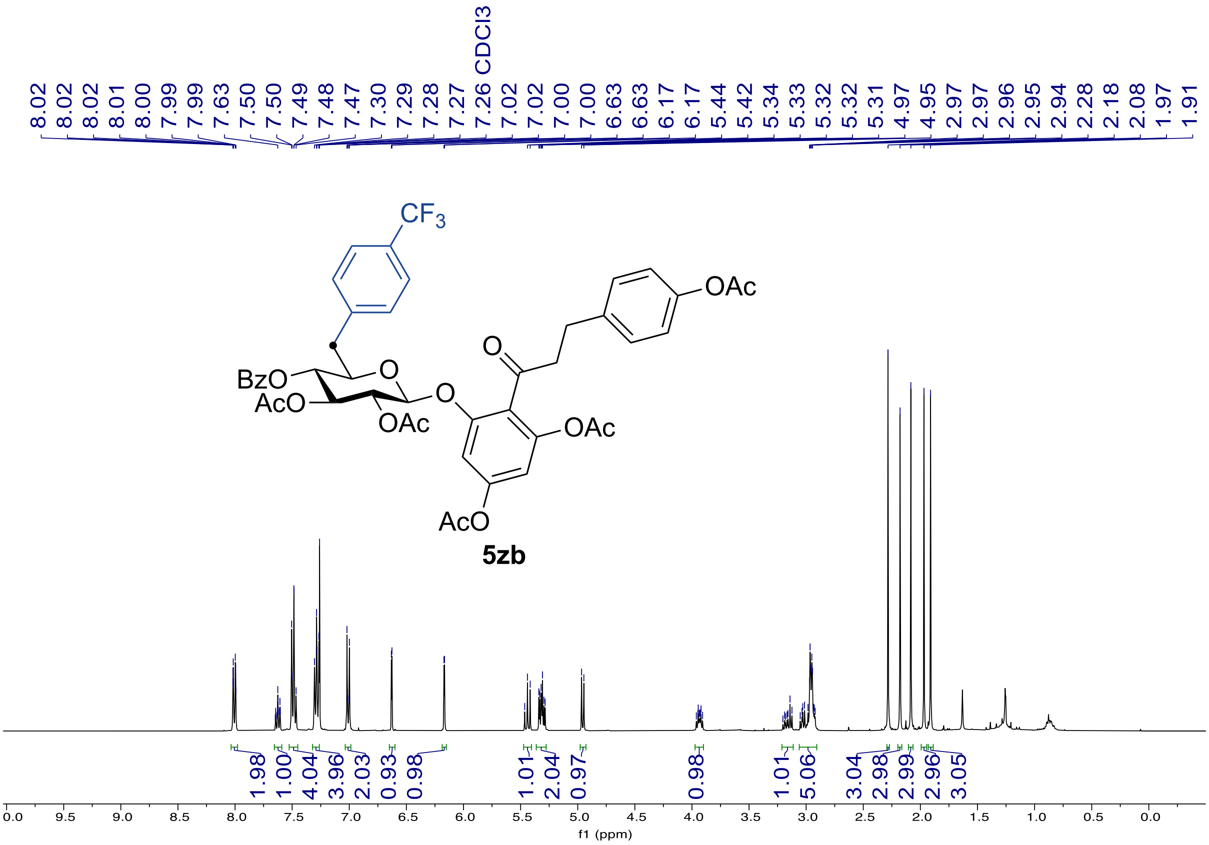

^{1}H NMR (400 MHz, CDCl$_3$) of 5zb

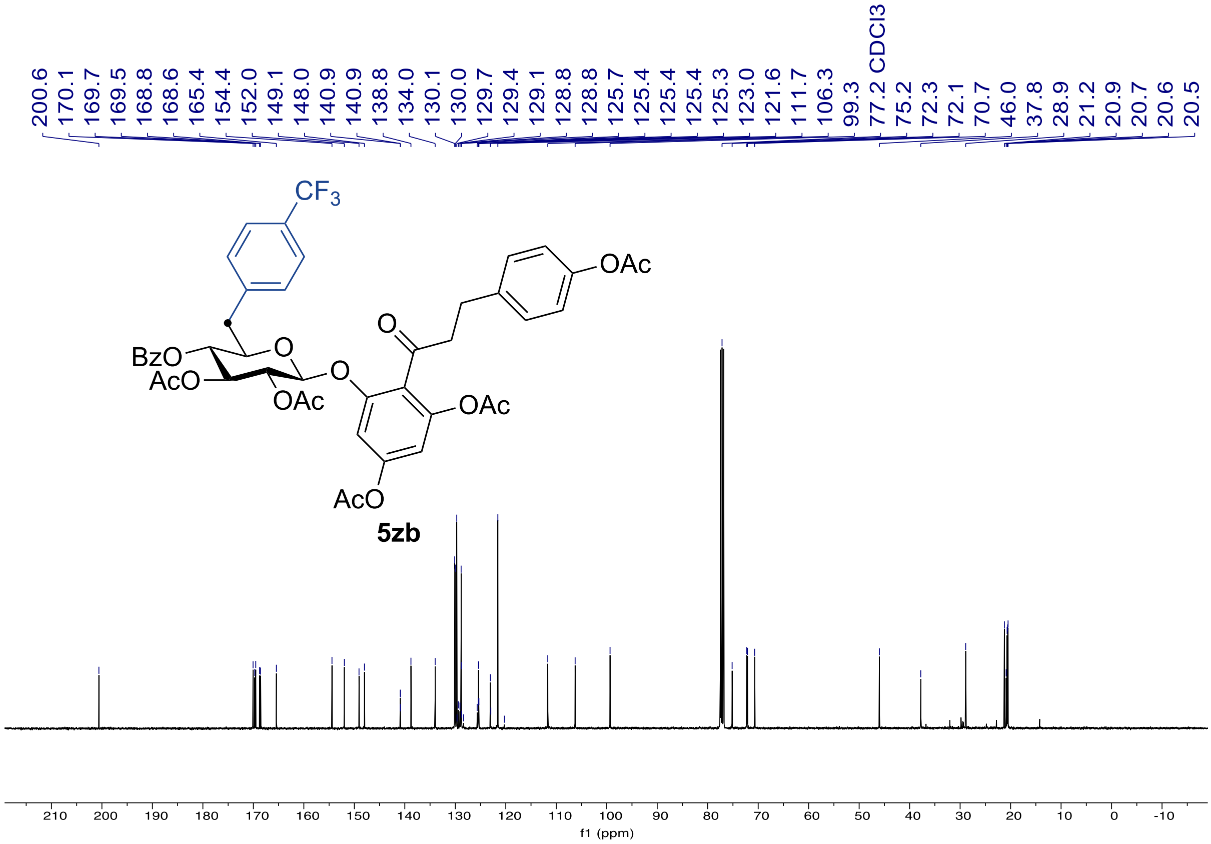

^{13}C NMR (101 MHz, CDCl3) of 5zb

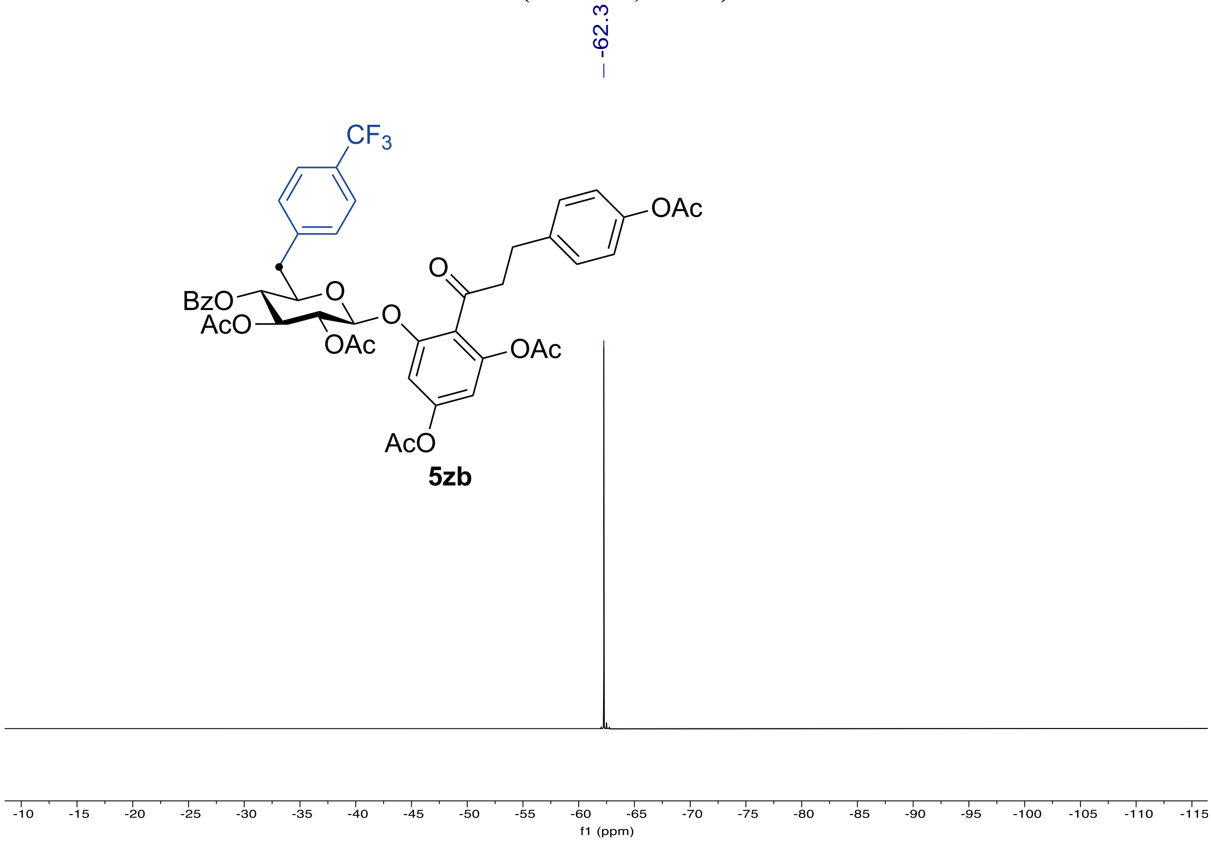

^{19}F NMR (376 MHz, CDCl3) of 5zb

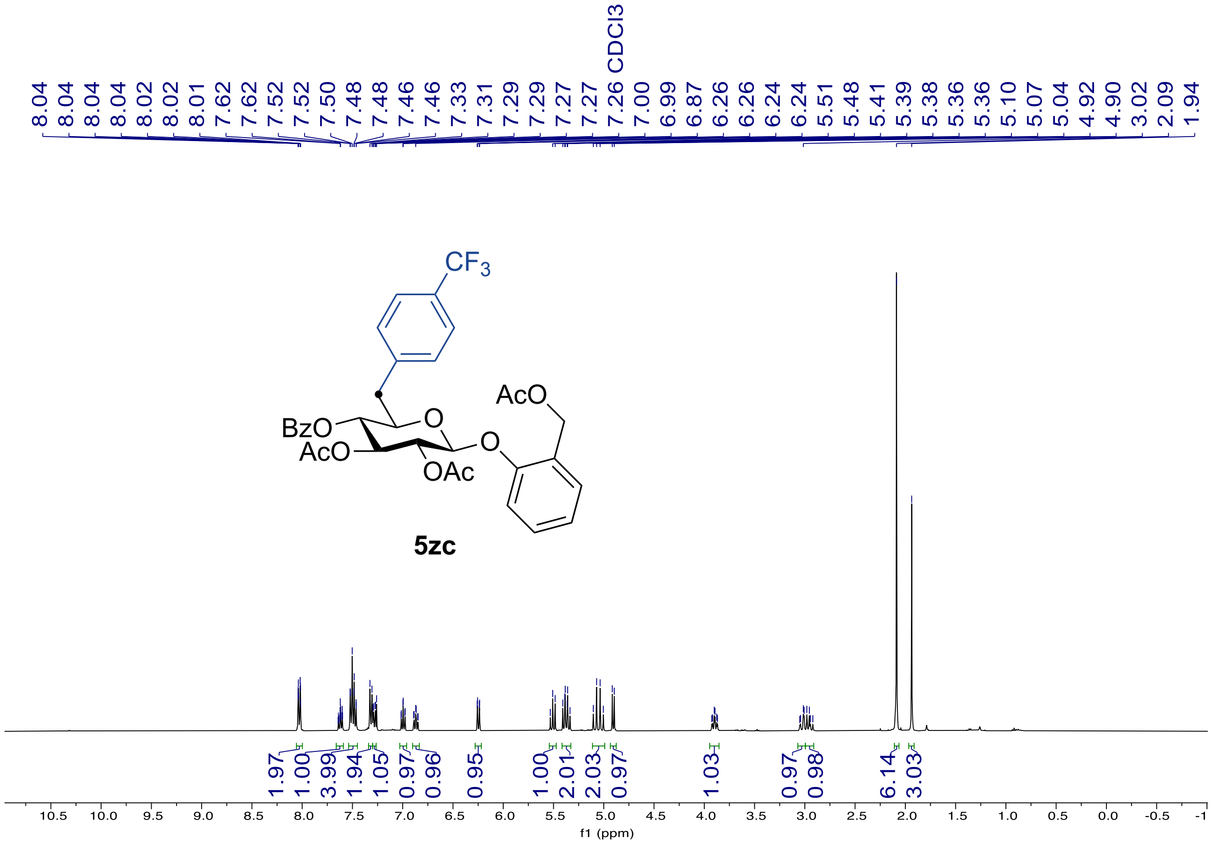

^{1}H NMR (400 MHz, CDCl$_3$) of 5zc

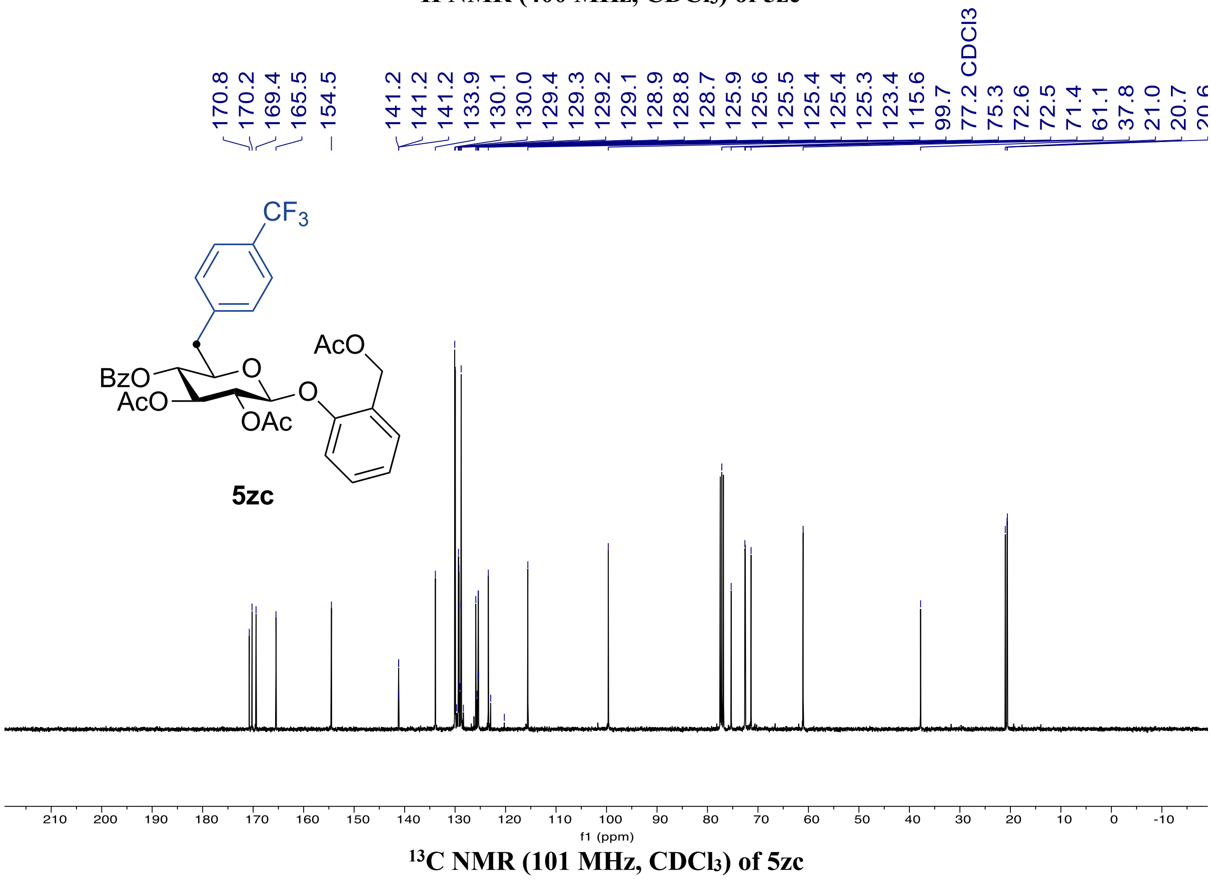

^{13}C NMR (101 MHz, CDCl$_3$) of 5zc

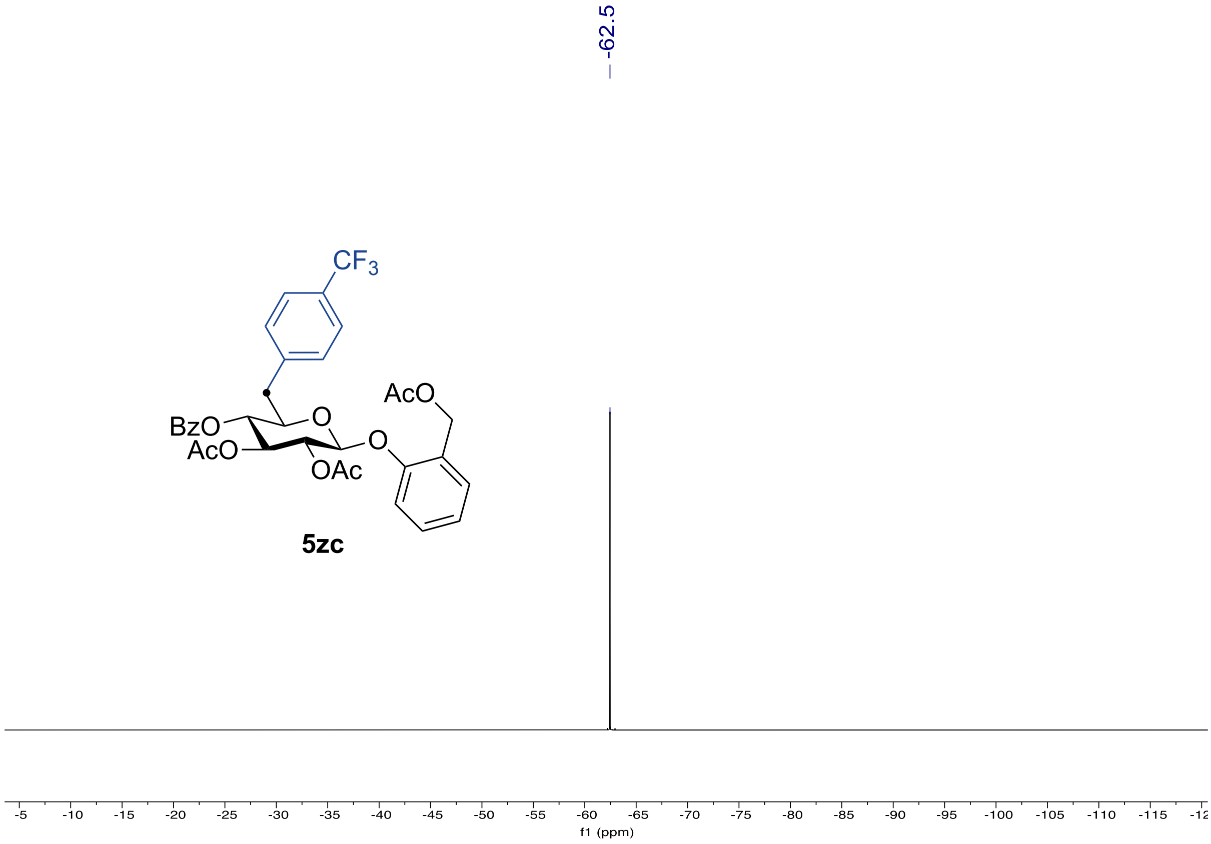

^{19}F NMR (376 MHz, $CDCl_3$) of 5zc

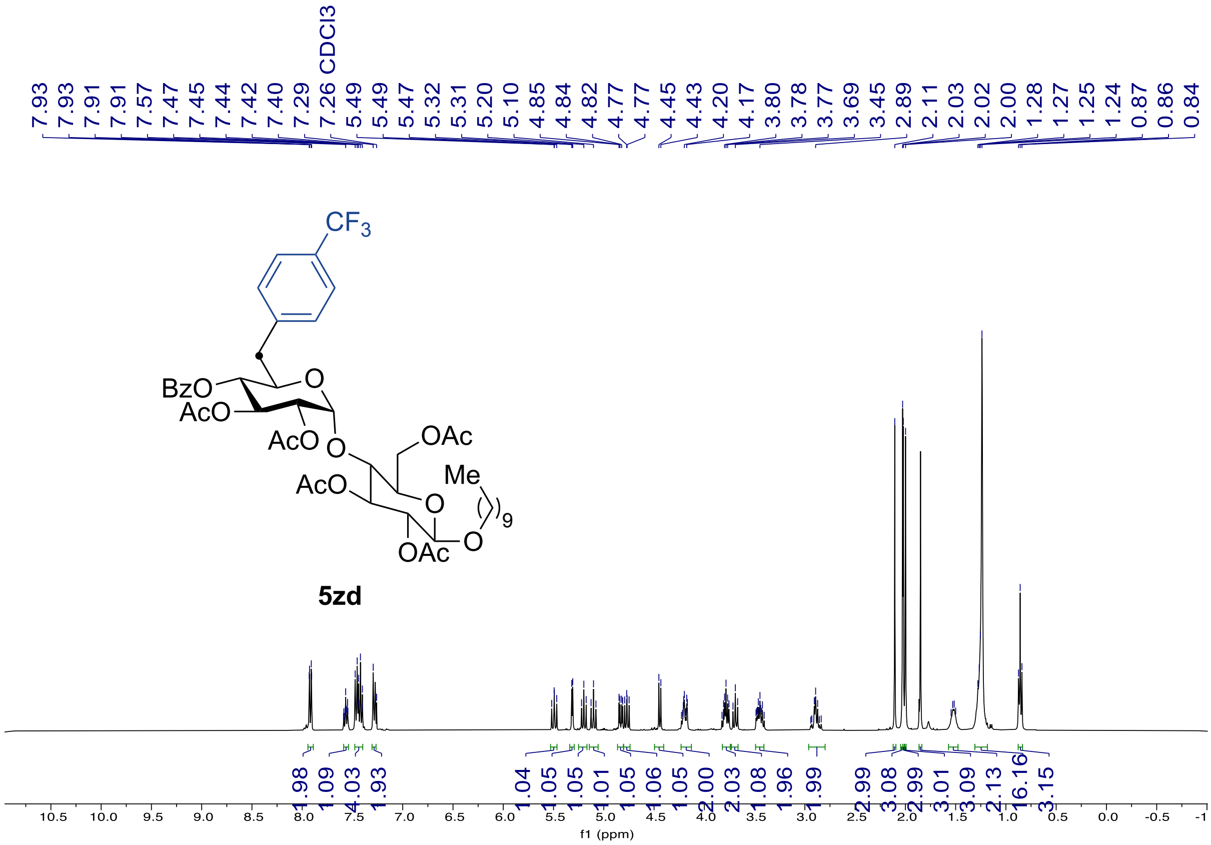

^{1}H NMR (400 MHz, $CDCl_3$) of 5zd

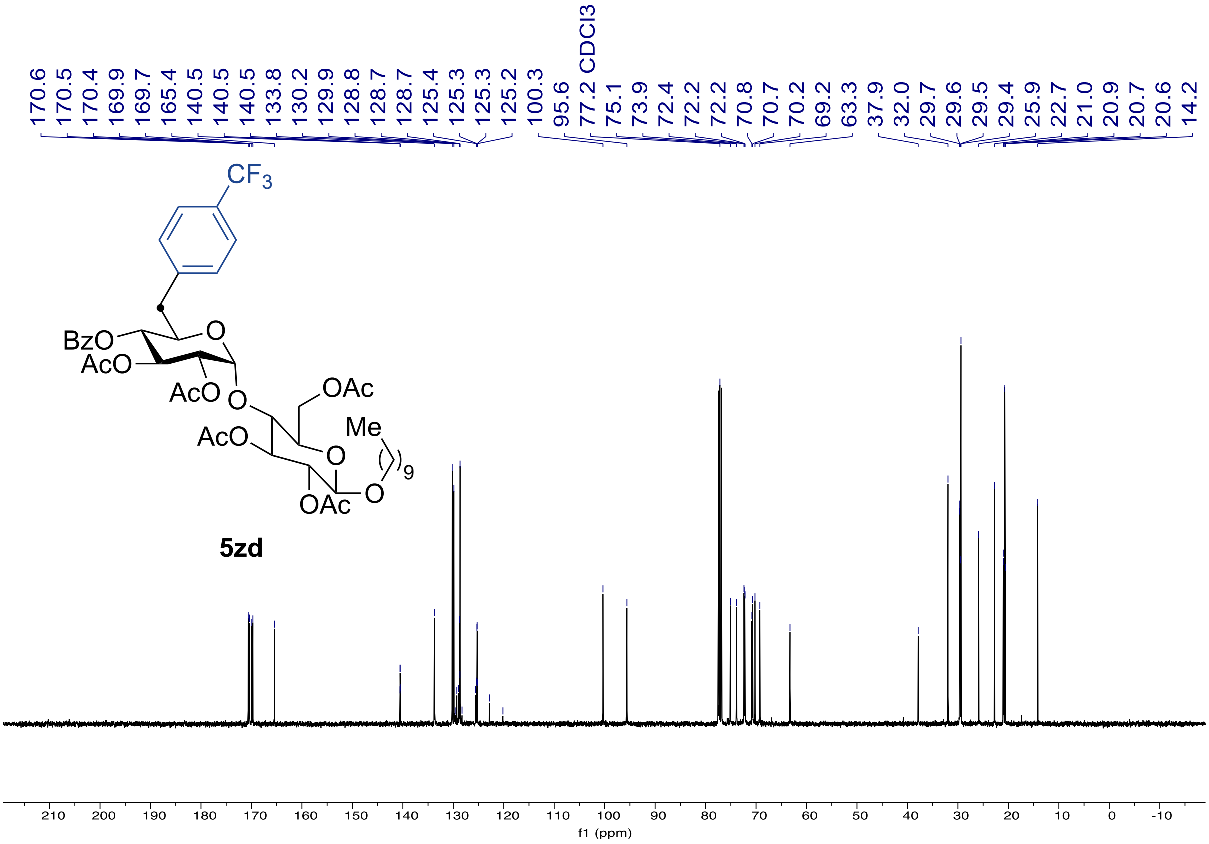

^{13}C NMR (101 MHz, CDCl$_3$) of 5zd

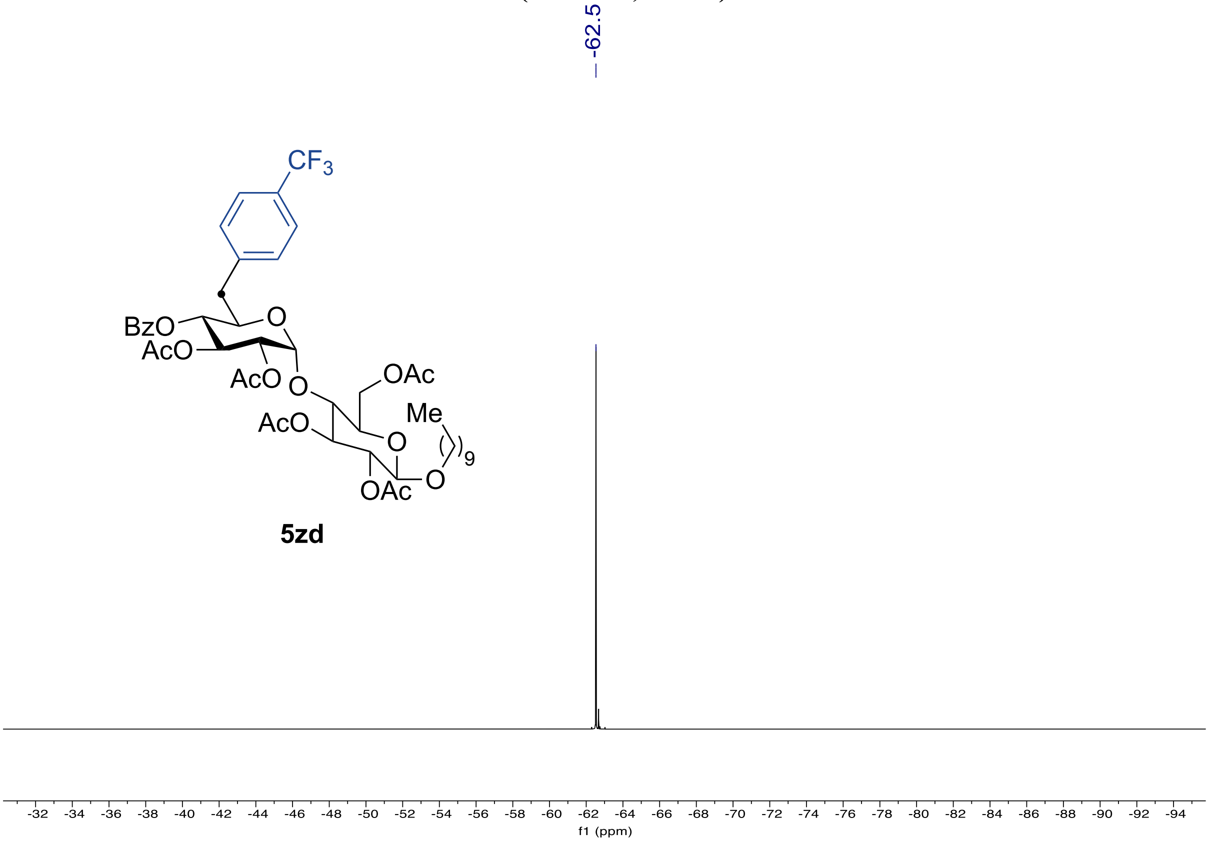

^{19}F NMR (376 MHz, CDCl$_3$) of 5zd

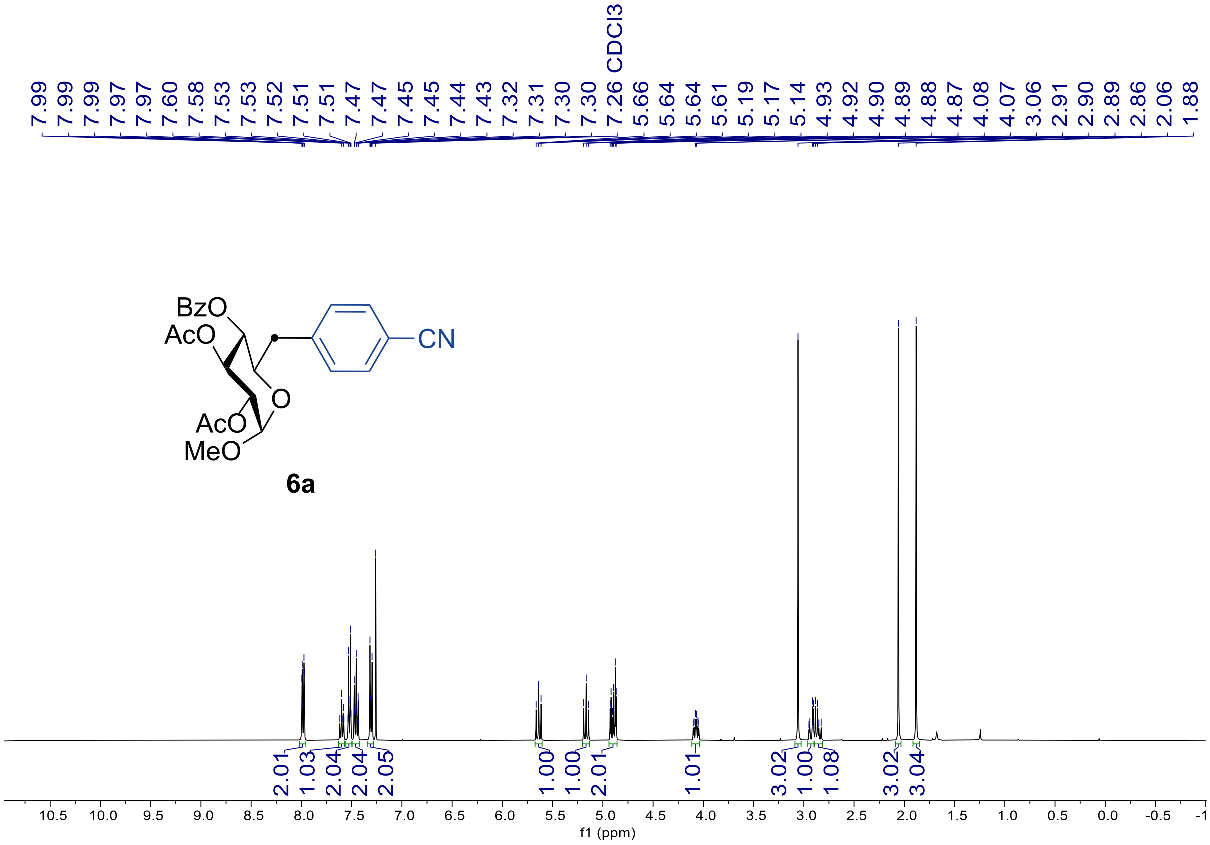

^{1}H NMR (400 MHz, $CDCl_3$) of 6a

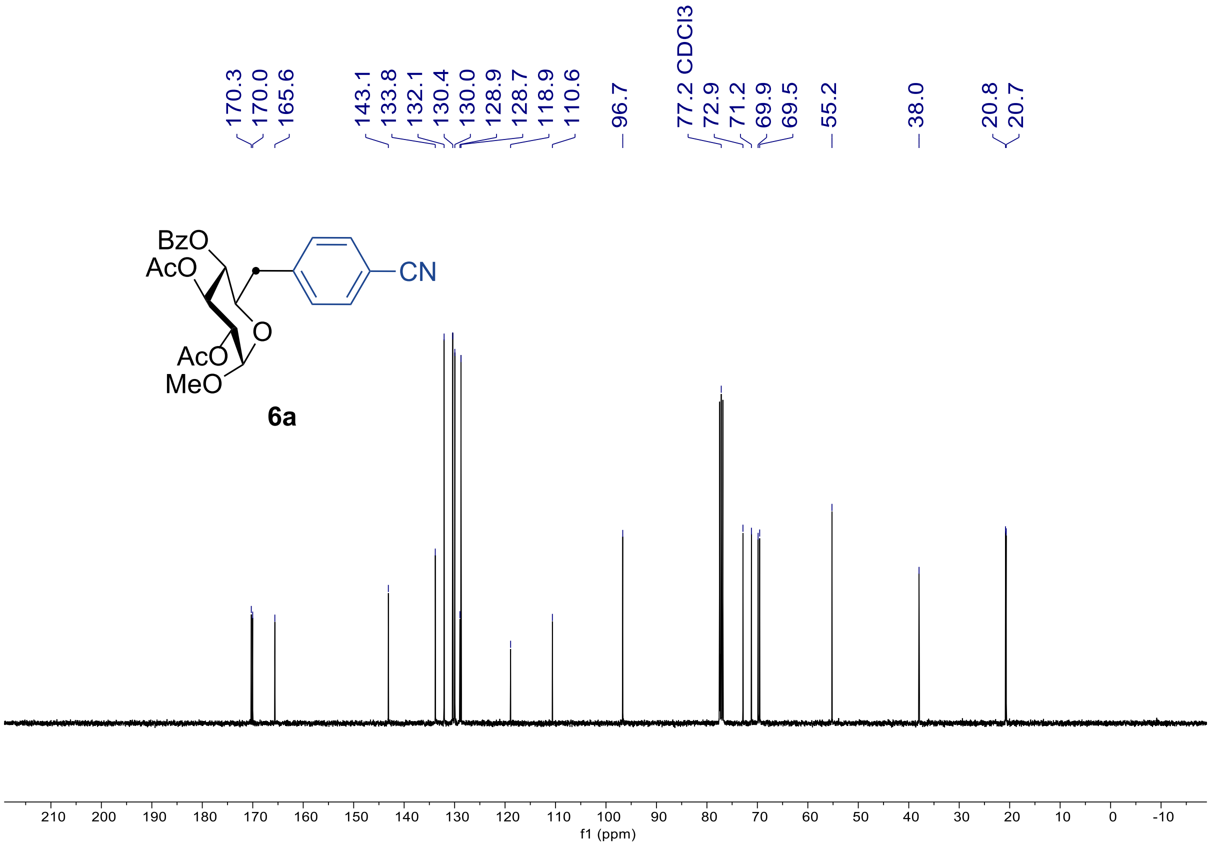

^{13}C NMR (101 MHz, $CDCl_3$) of 6a

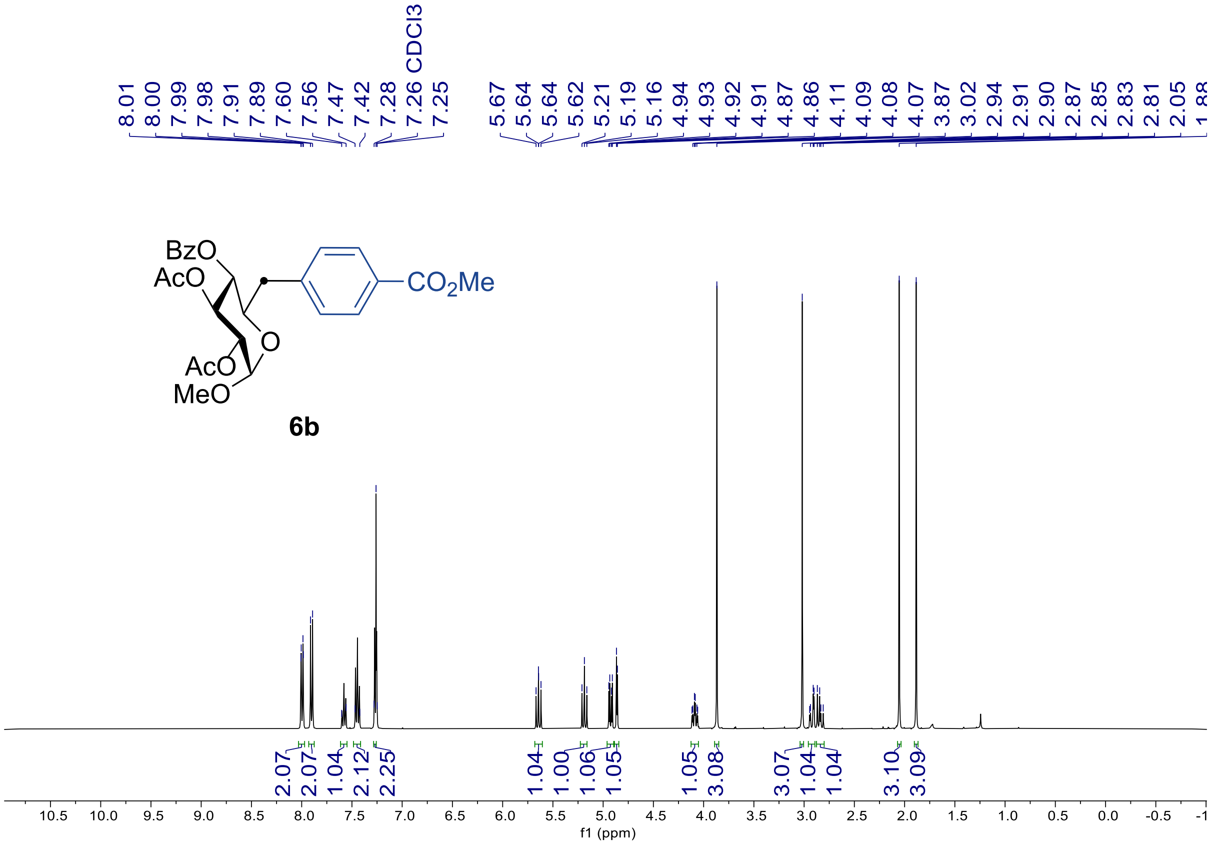

^{1}H NMR (400 MHz, CDCl₃) of 6b

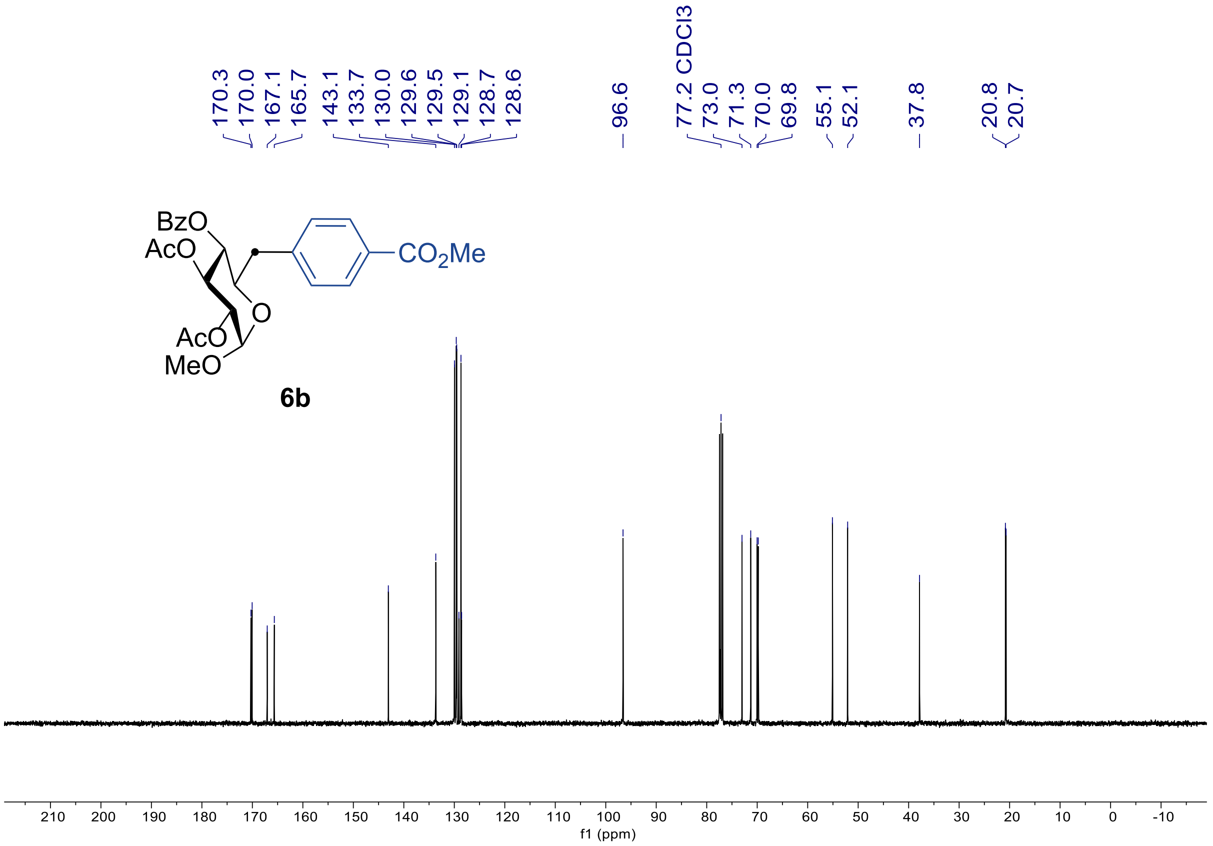

^{13}C NMR (101 MHz, CDCl₃) of 6b

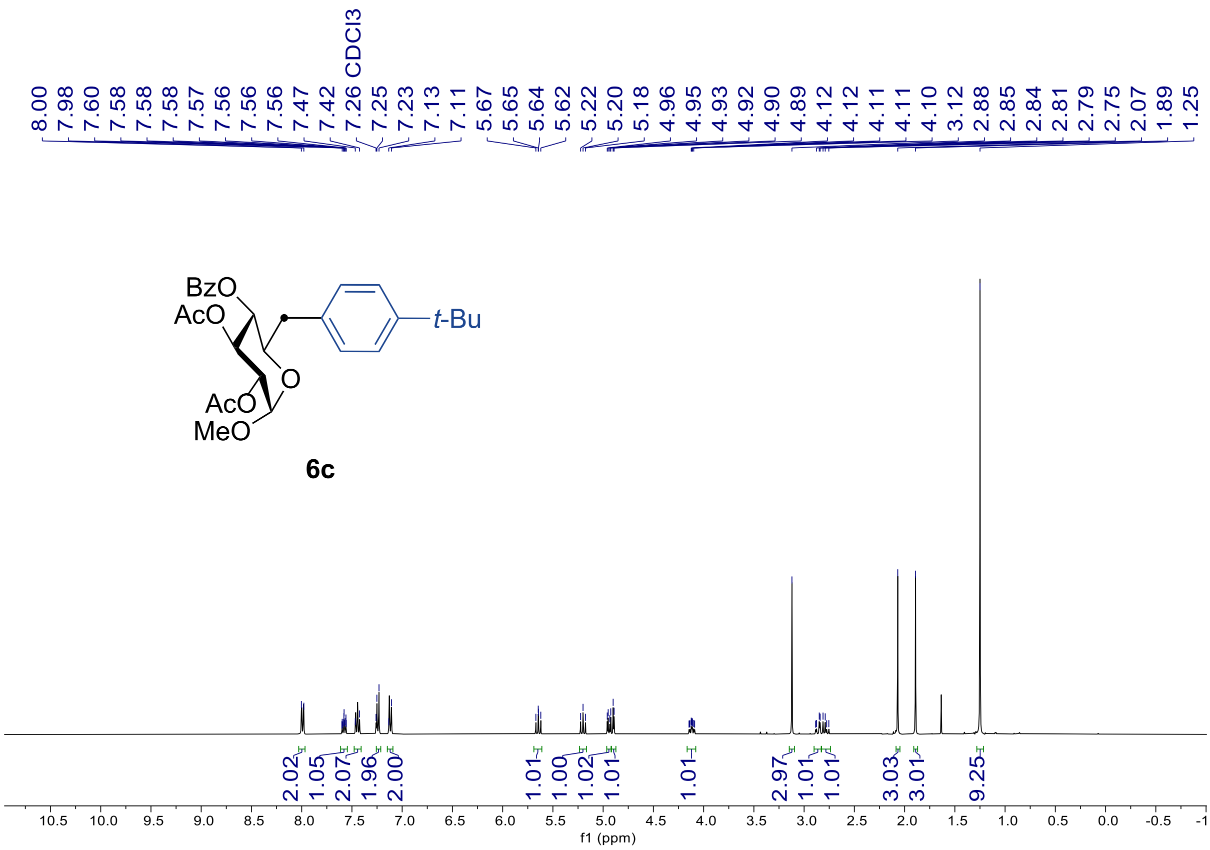

^{1}H NMR (400 MHz, CDCl$_3$) of 6c

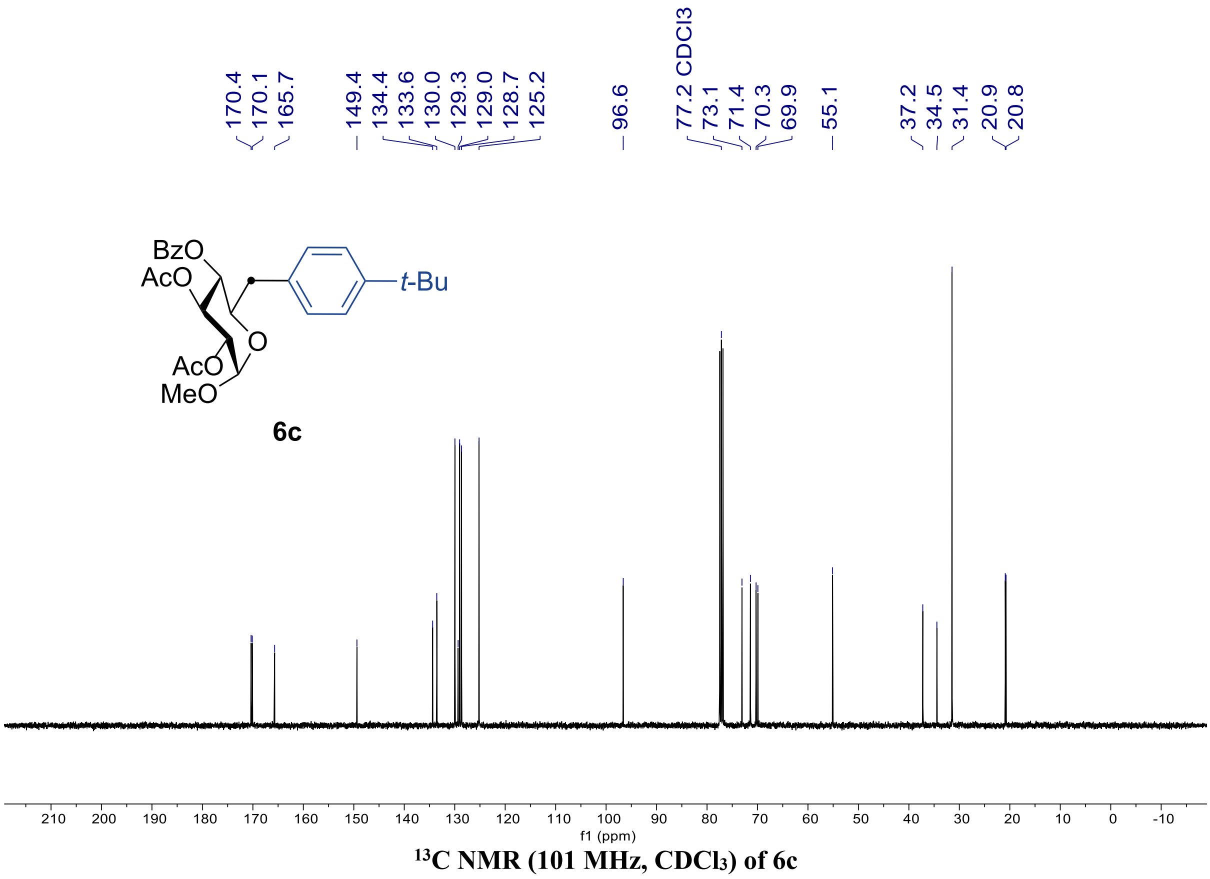

^{13}C NMR (101 MHz, CDCl$_3$) of 6c

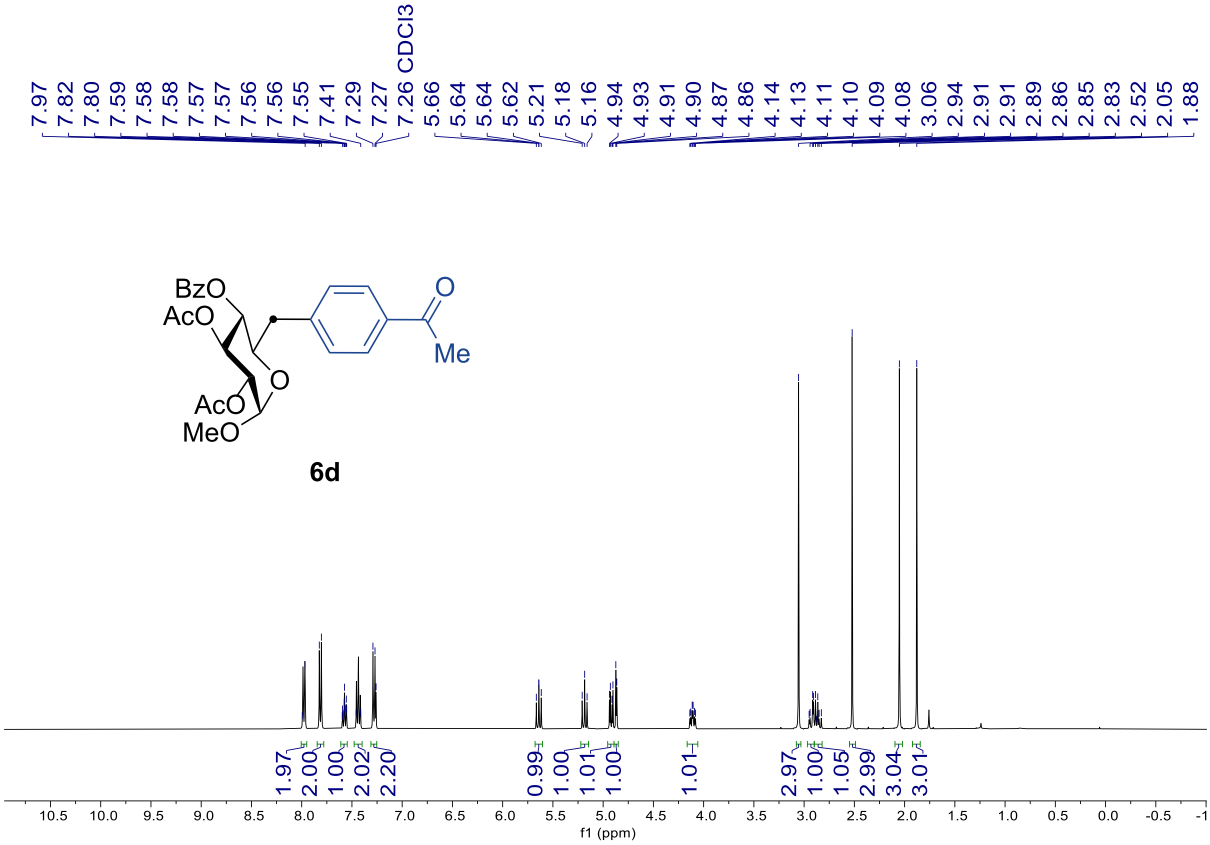

^{1}H NMR (400 MHz, $CDCl_3$) of 6d

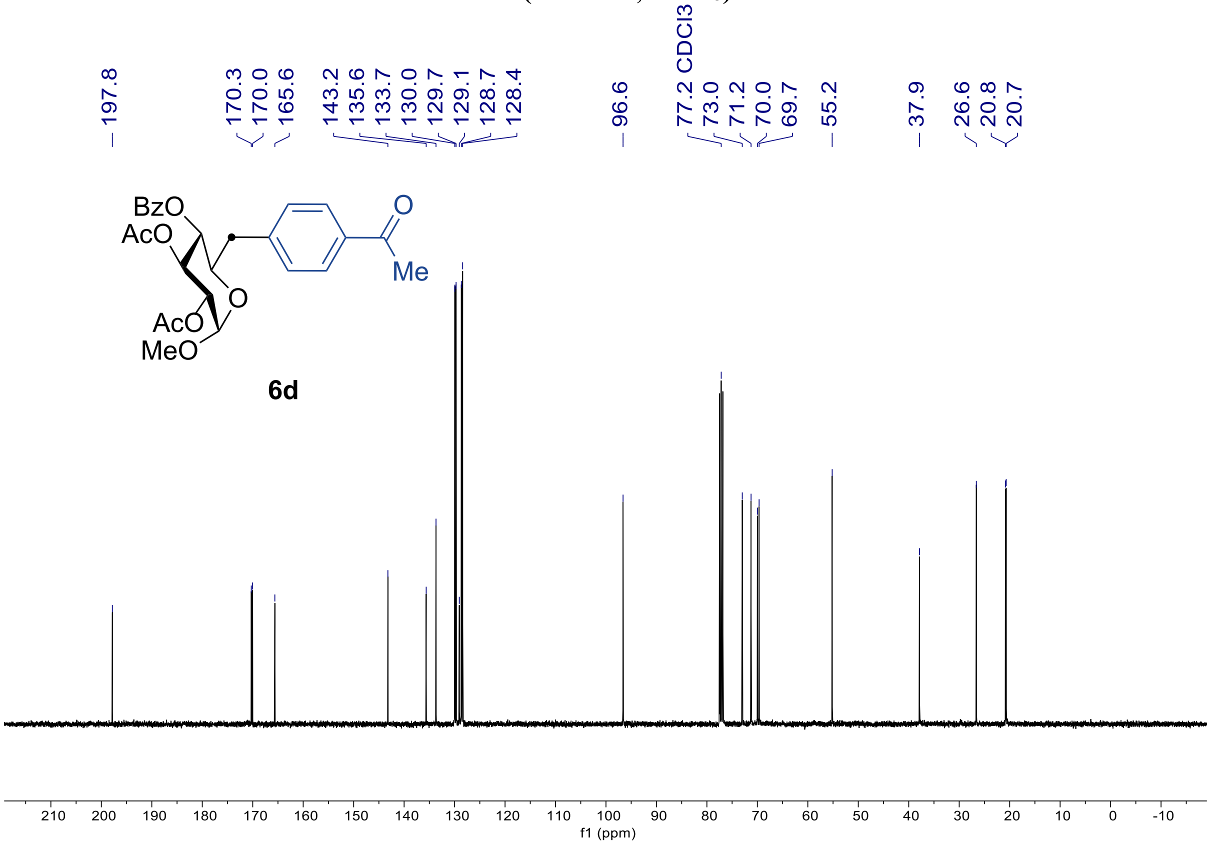

^{13}C NMR (101 MHz, $CDCl_3$) of 6d

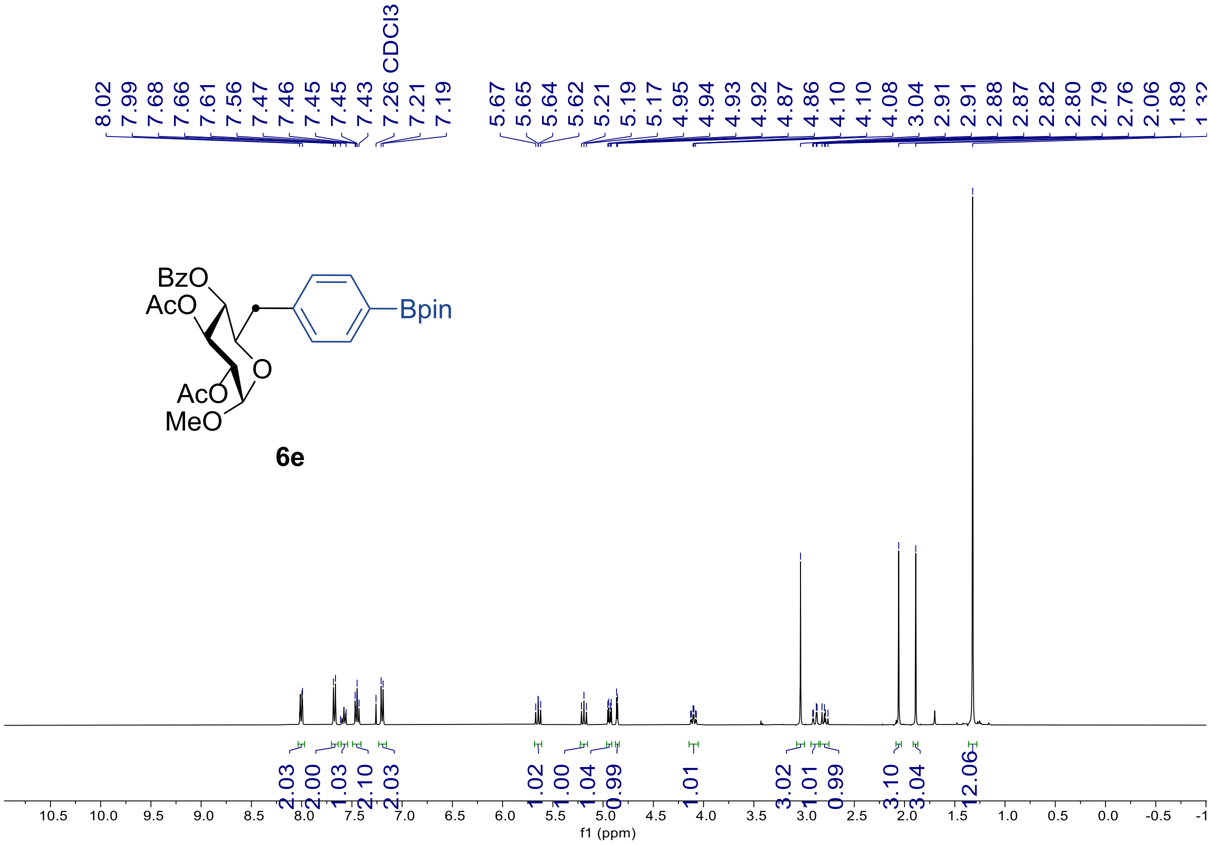

^{1}H NMR (400 MHz, $CDCl_3$) of 6e

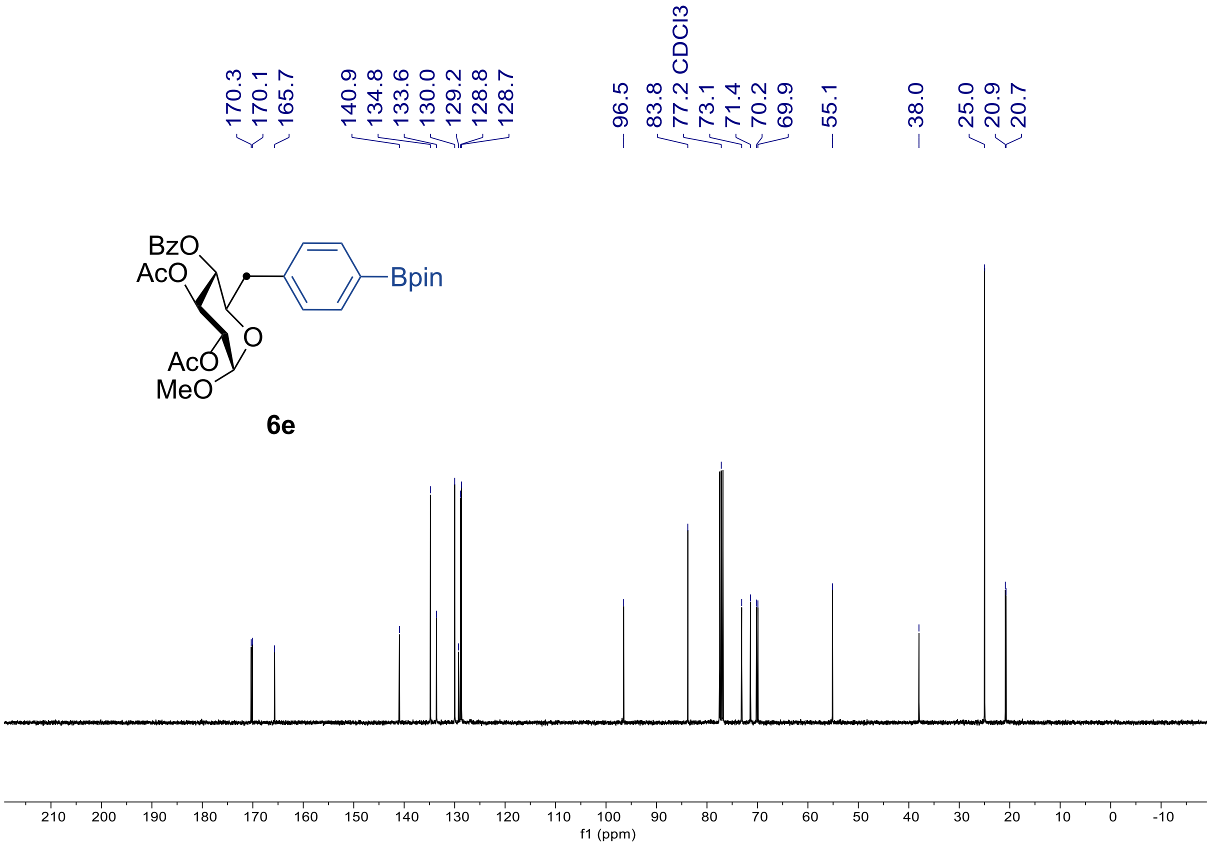

^{13}C NMR (101 MHz, $CDCl_3$) of 6e

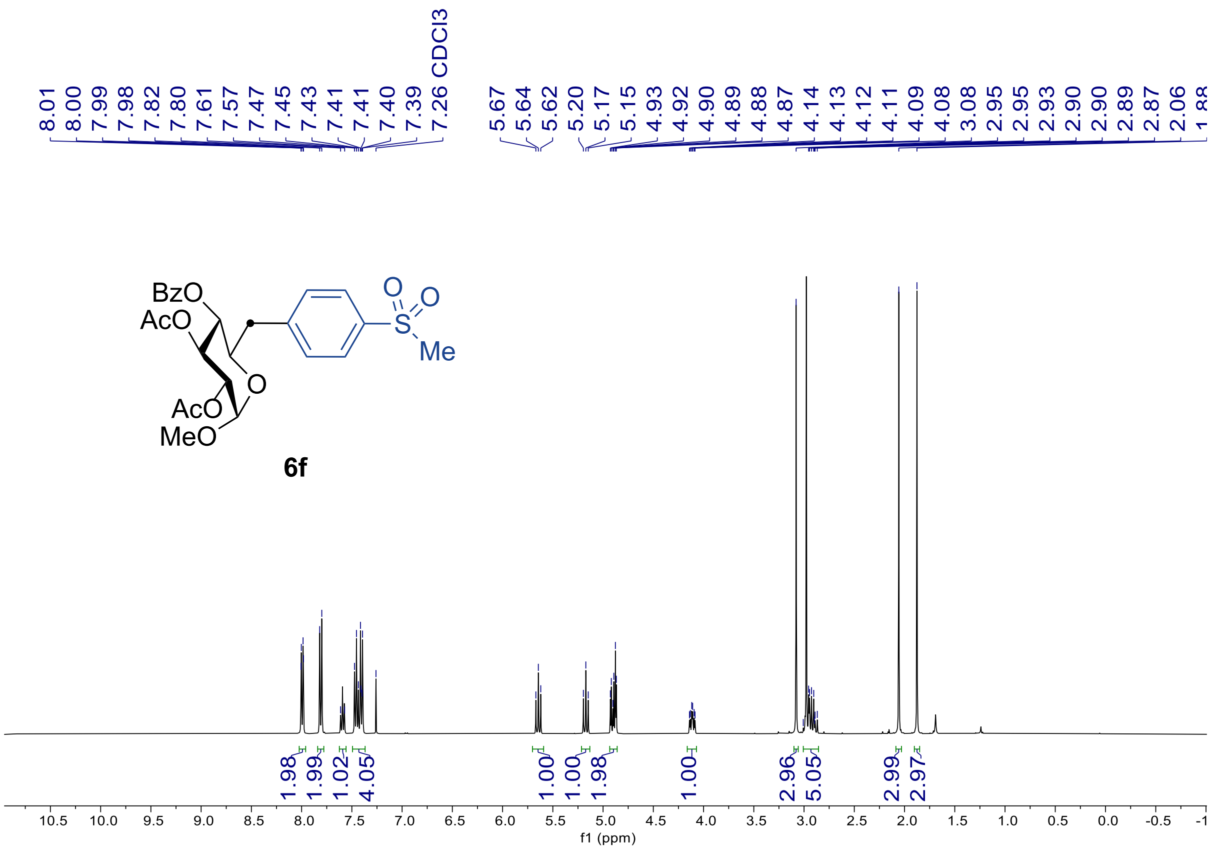

^{1}H NMR (400 MHz, CDCl$_3$) of 6f

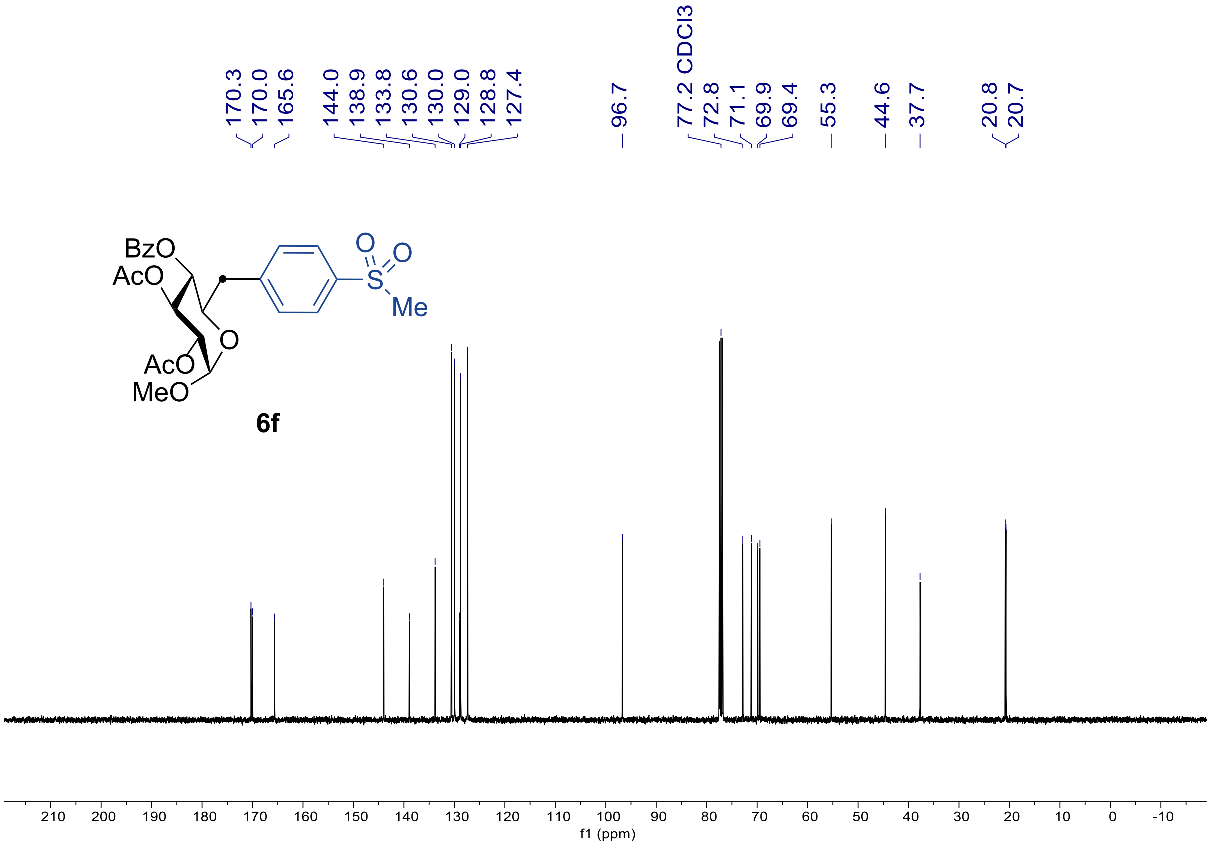

^{13}C NMR (101 MHz, CDCl$_3$) of 6f

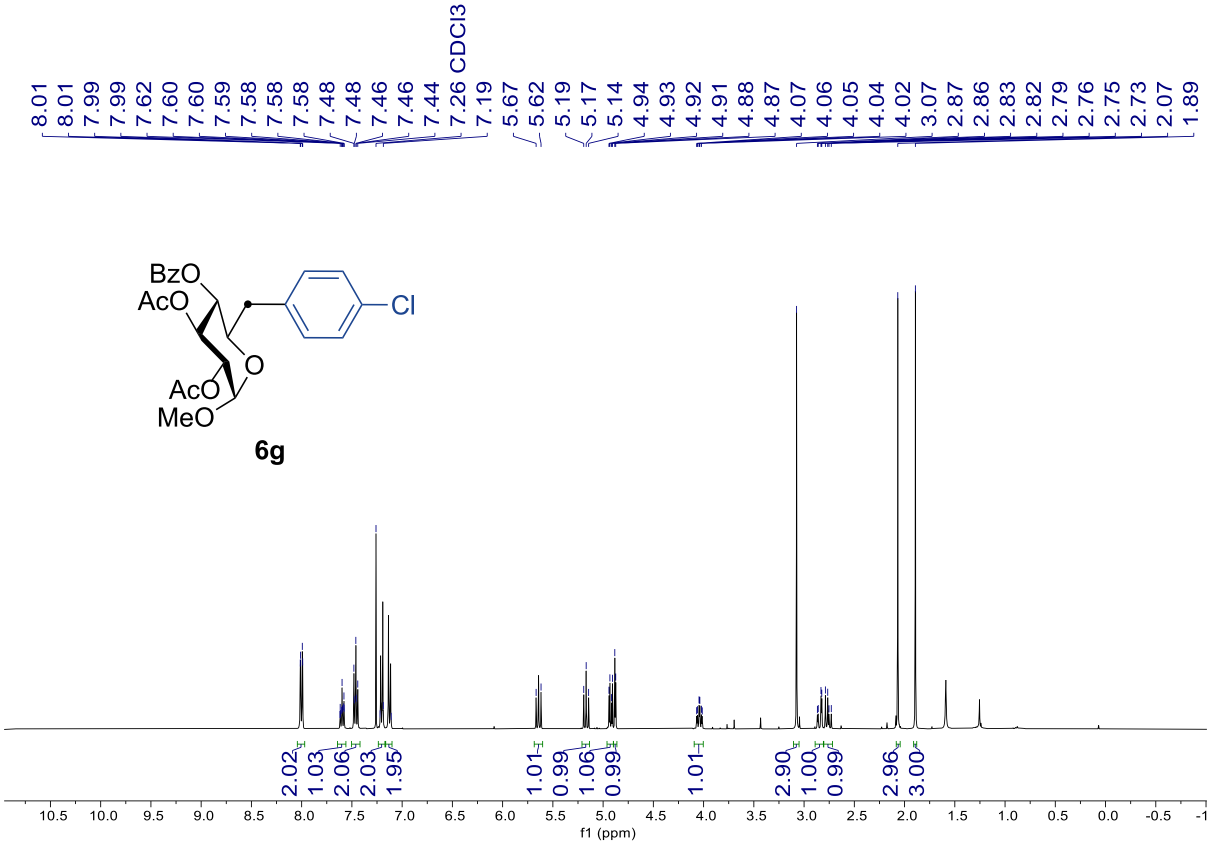

^{13}C NMR (101 MHz, CDCl$_3$) of 6g

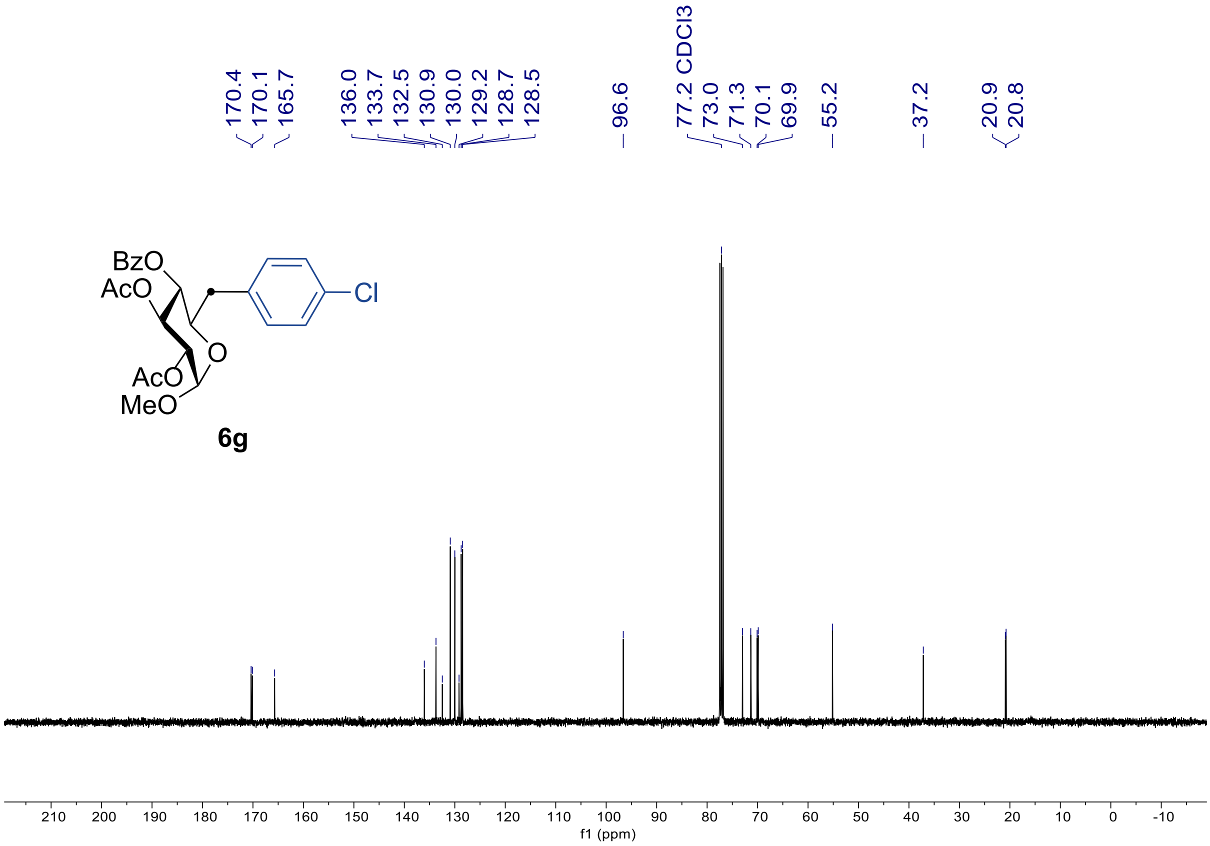

^{13}C NMR (101 MHz, CDCl$_3$) of 6g

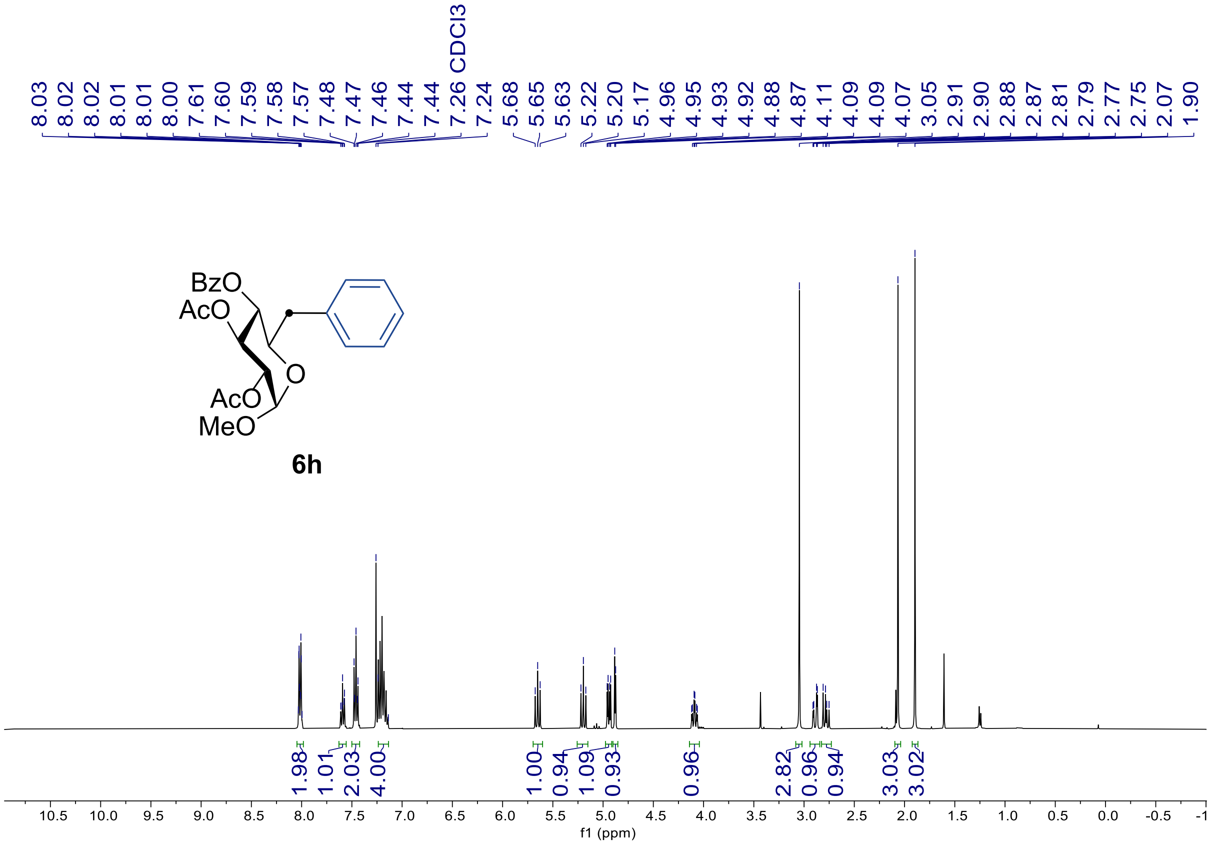

^{1}H NMR (400 MHz, CDCl$_3$) of 6h

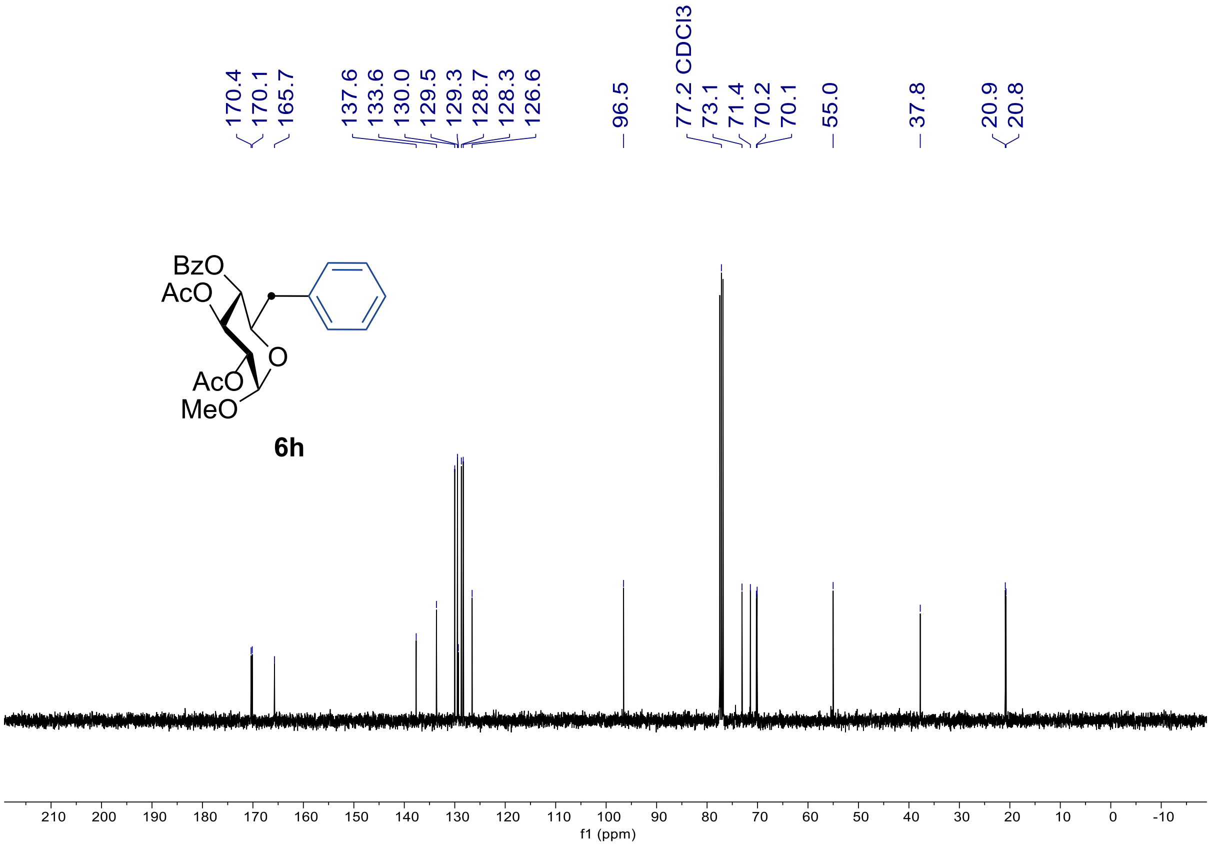

^{13}C NMR (101 MHz, CDCl$_3$) of 6h

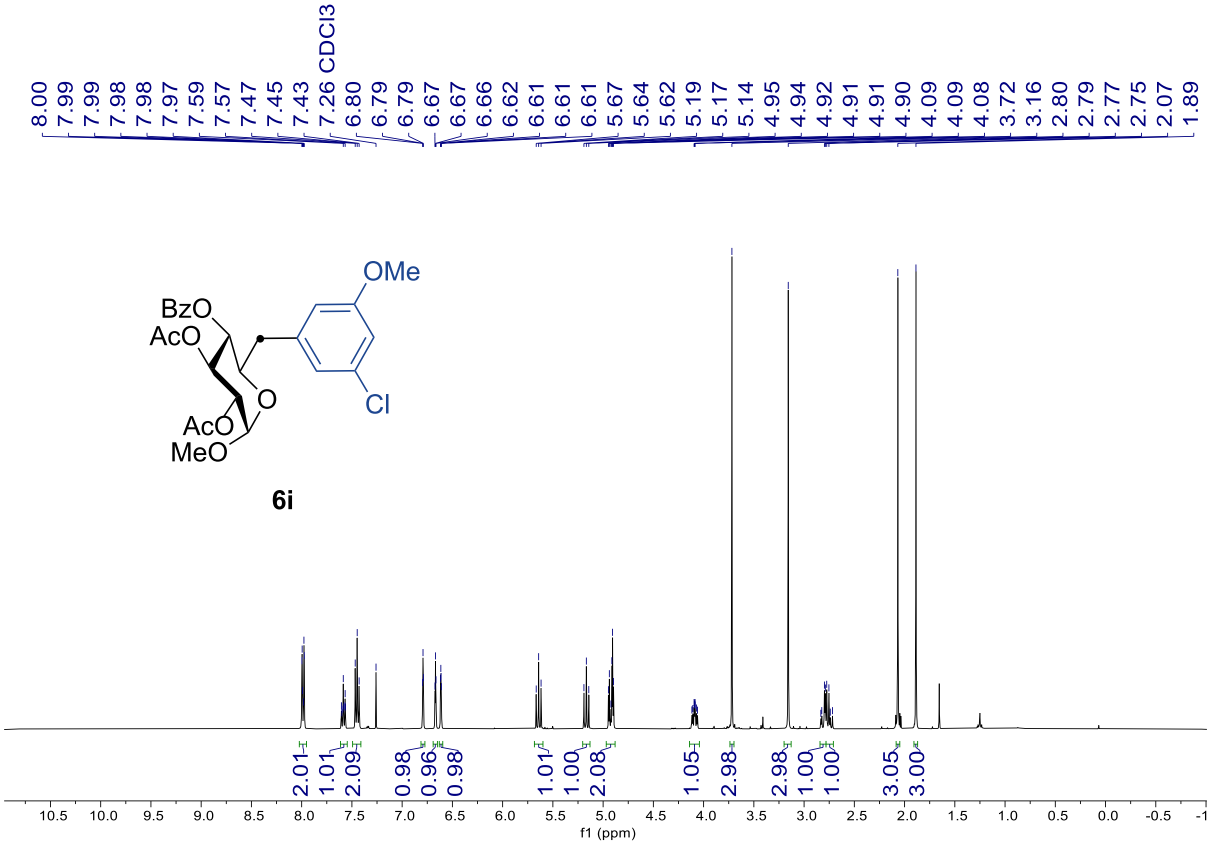

^{1}H NMR (400 MHz, CDCl3) of 6i

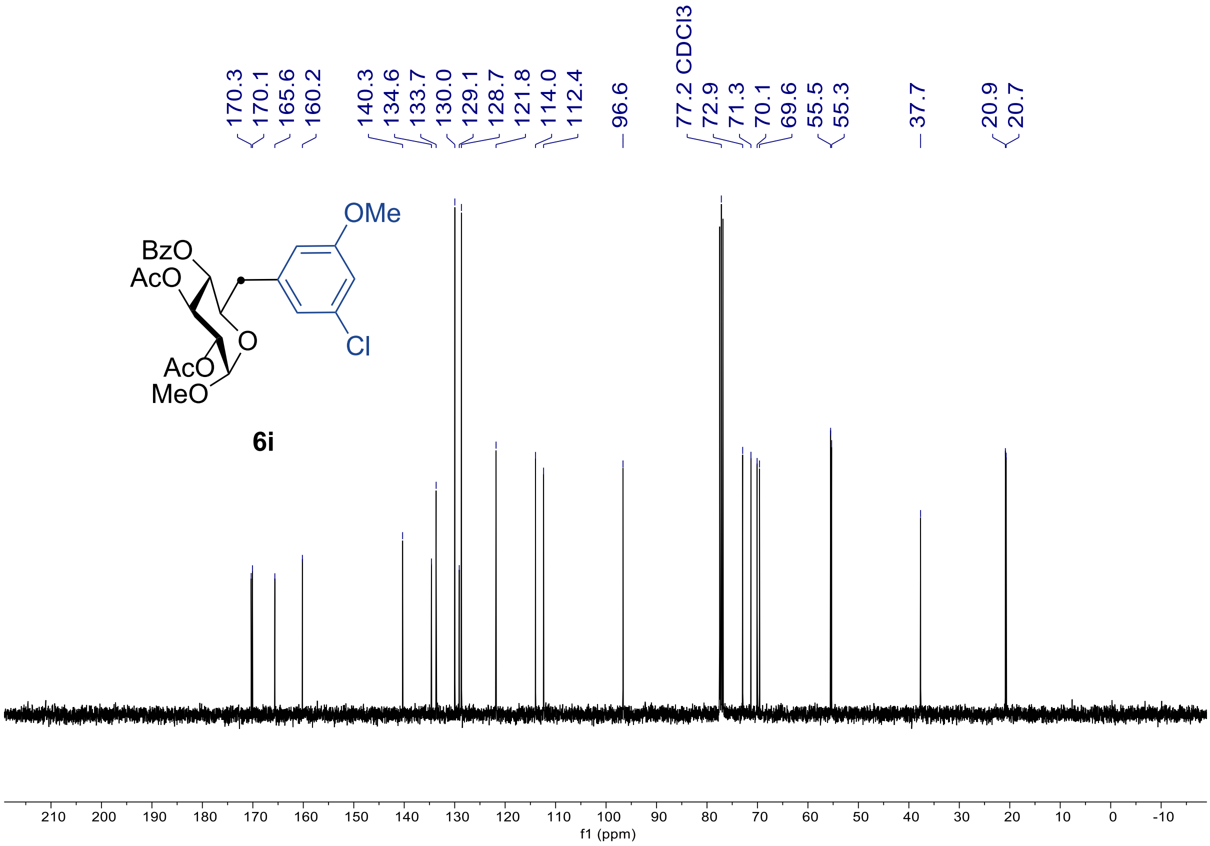

^{13}C NMR (101 MHz, CDCl3) of 6i

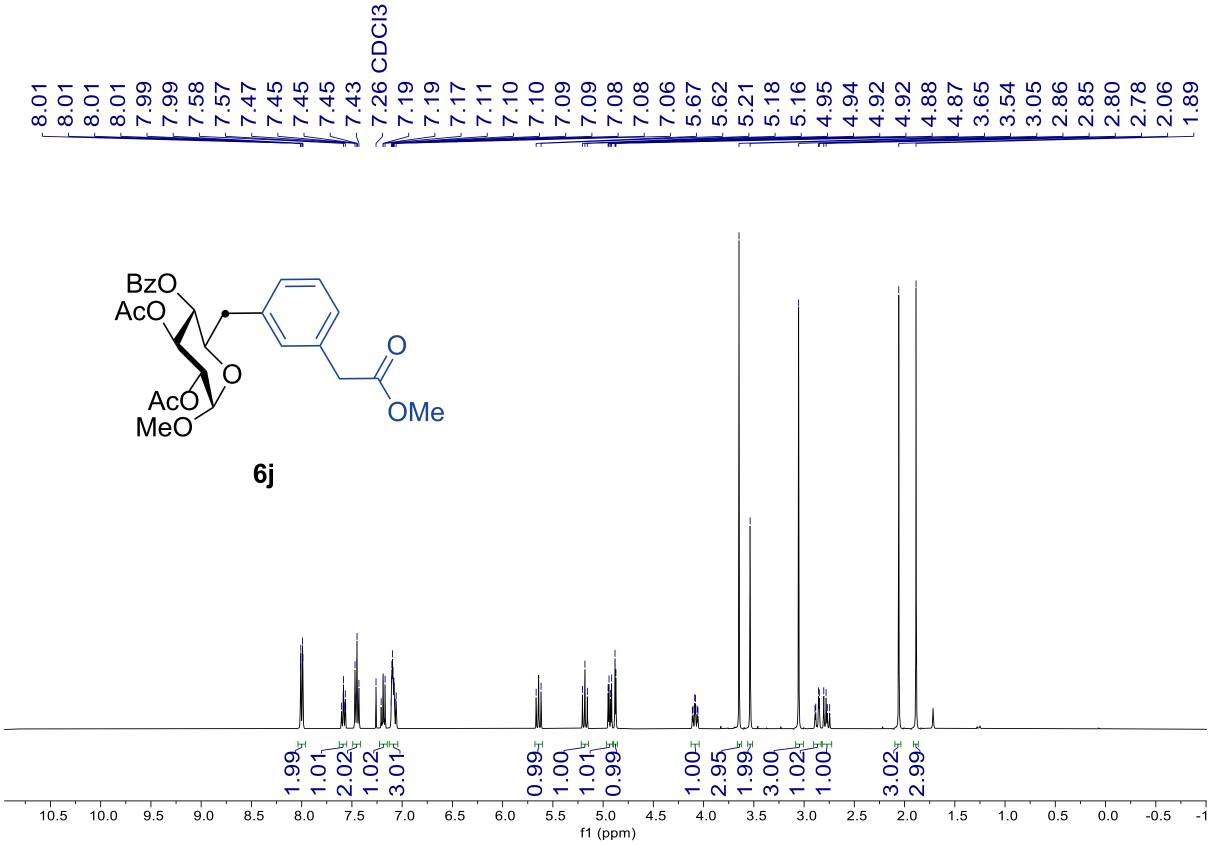

^{1}H NMR (400 MHz, $CDCl_3$) of 6j

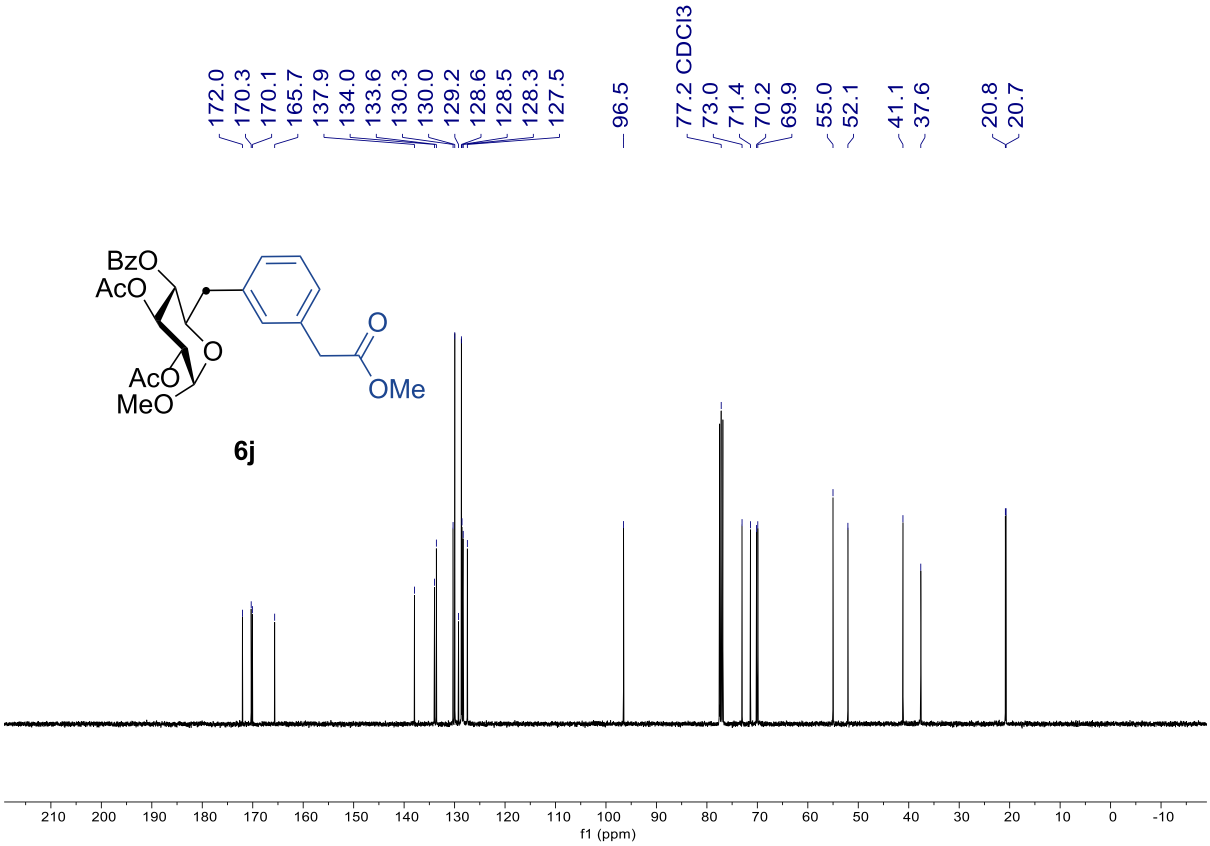

^{13}C NMR (101 MHz, $CDCl_3$) of 6j

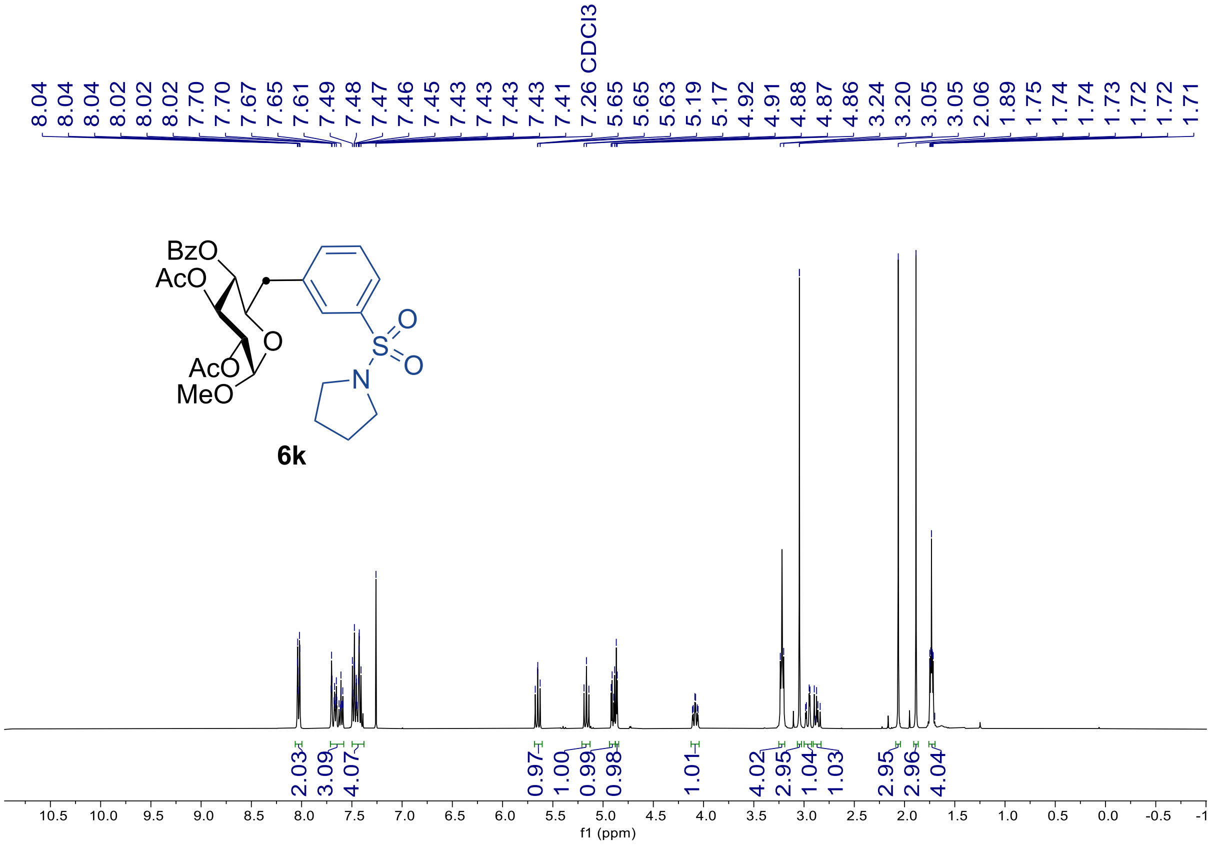

^{1}H NMR (400 MHz, CDCl₃) of 6k

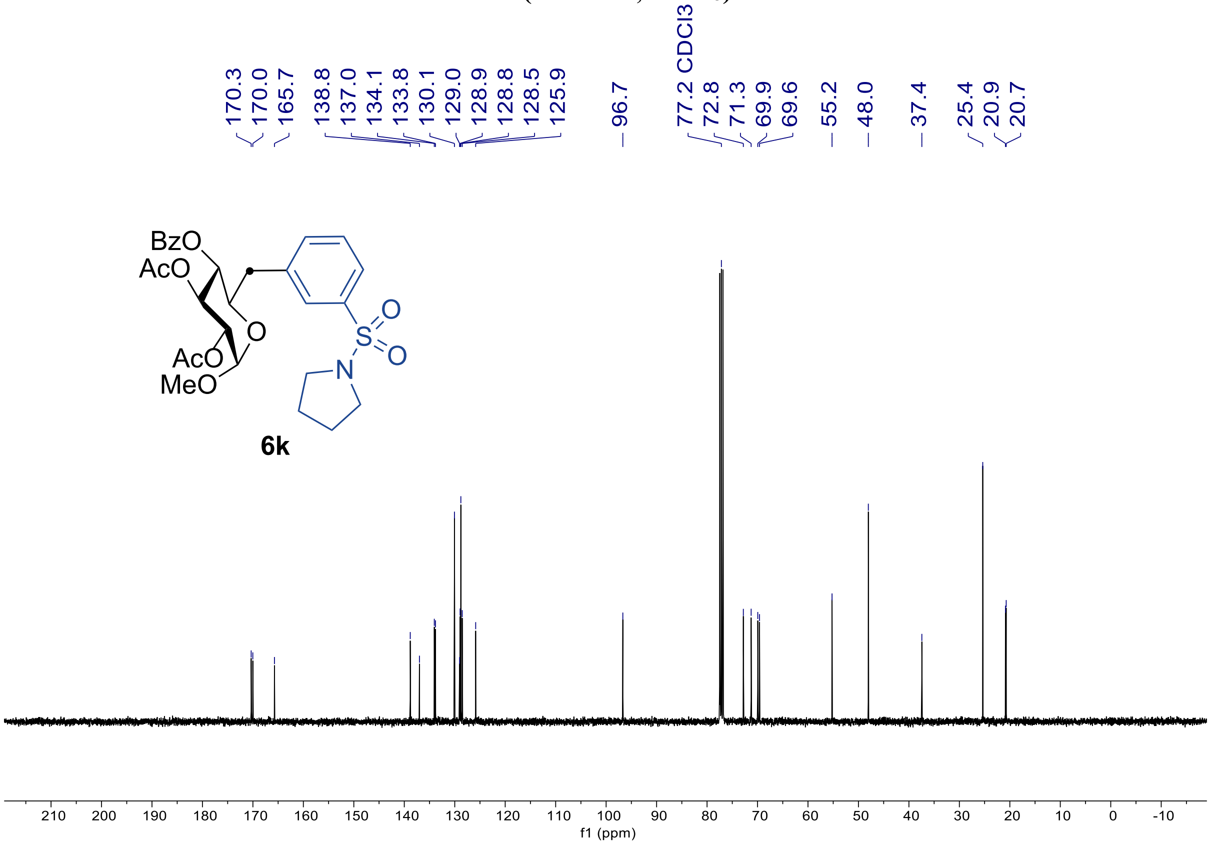

^{13}C NMR (101 MHz, CDCl₃) of 6k

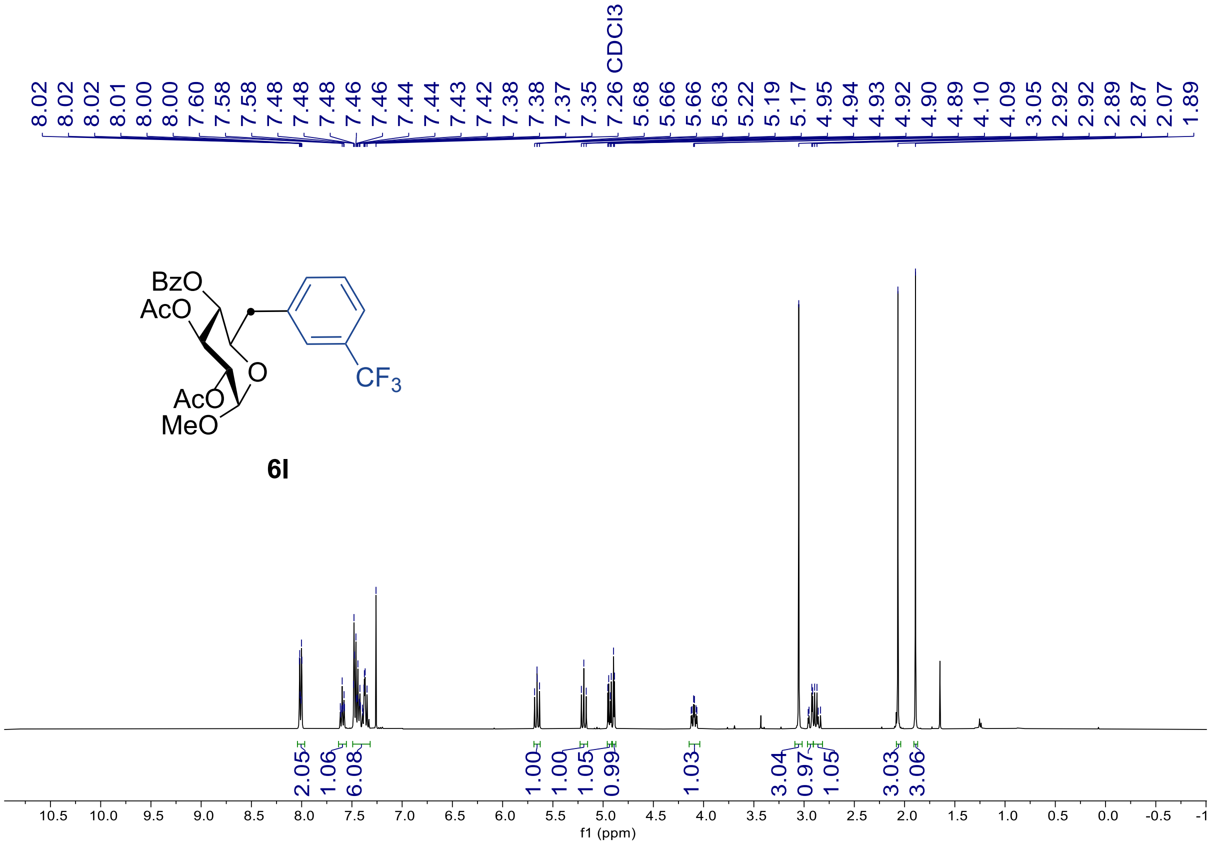

^{1}H NMR (400 MHz, CDCl3) of 6l

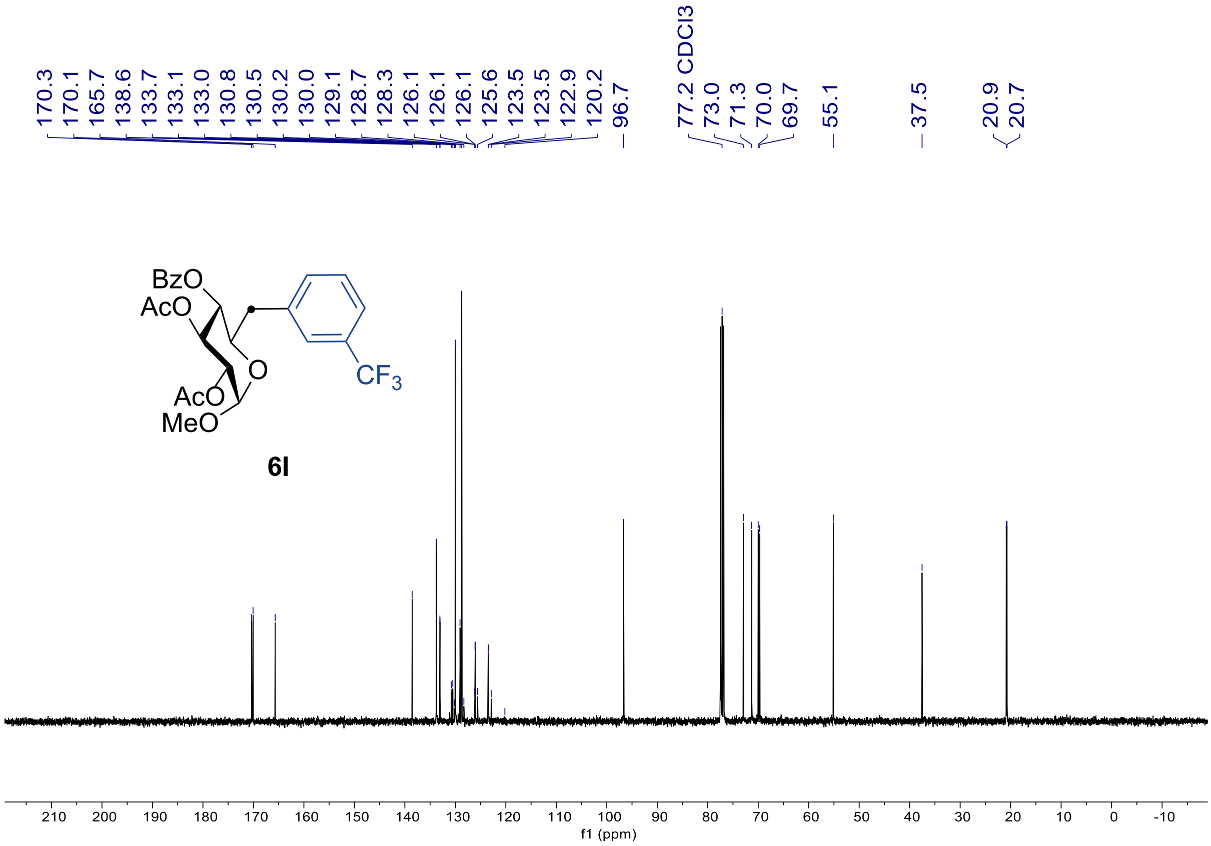

^{13}C NMR (101 MHz, CDCl3) of 6l

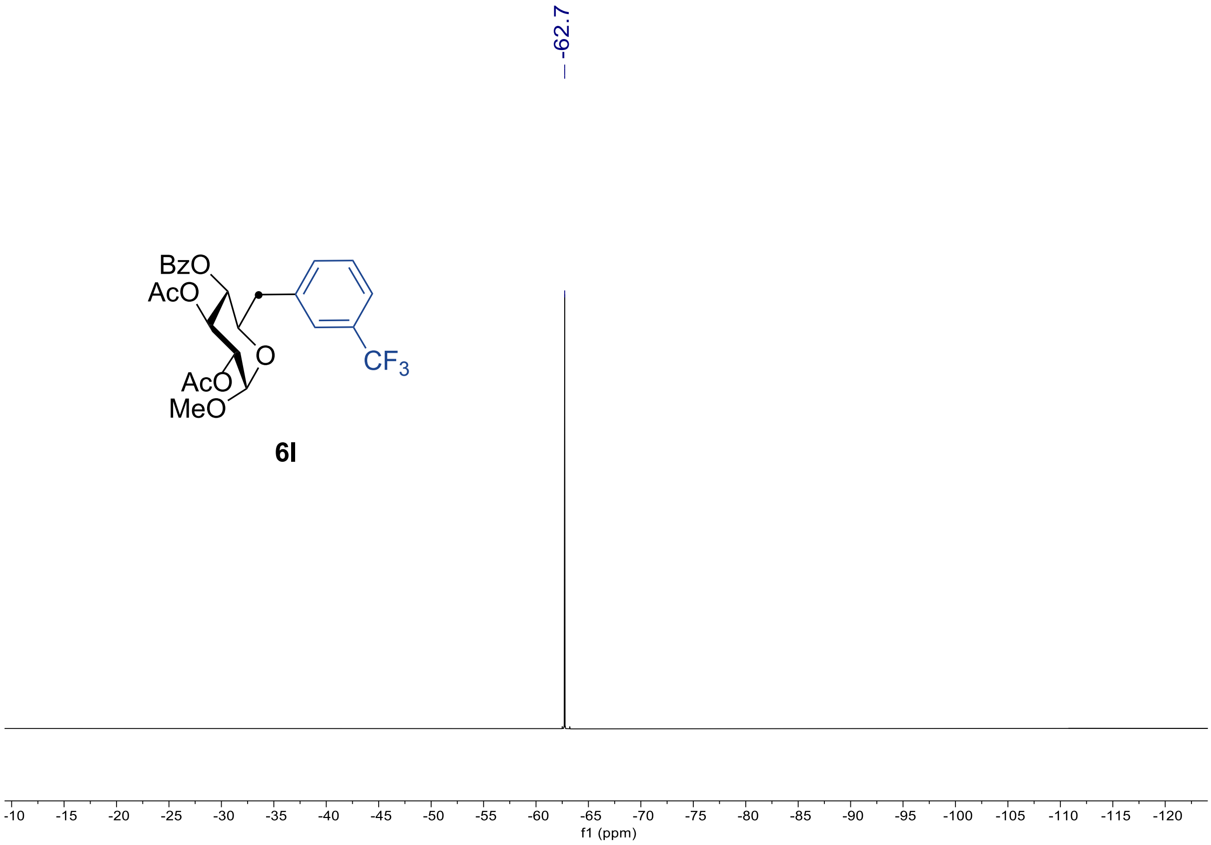

^{19}F NMR (376 MHz, CDCl$_3$) of 6l

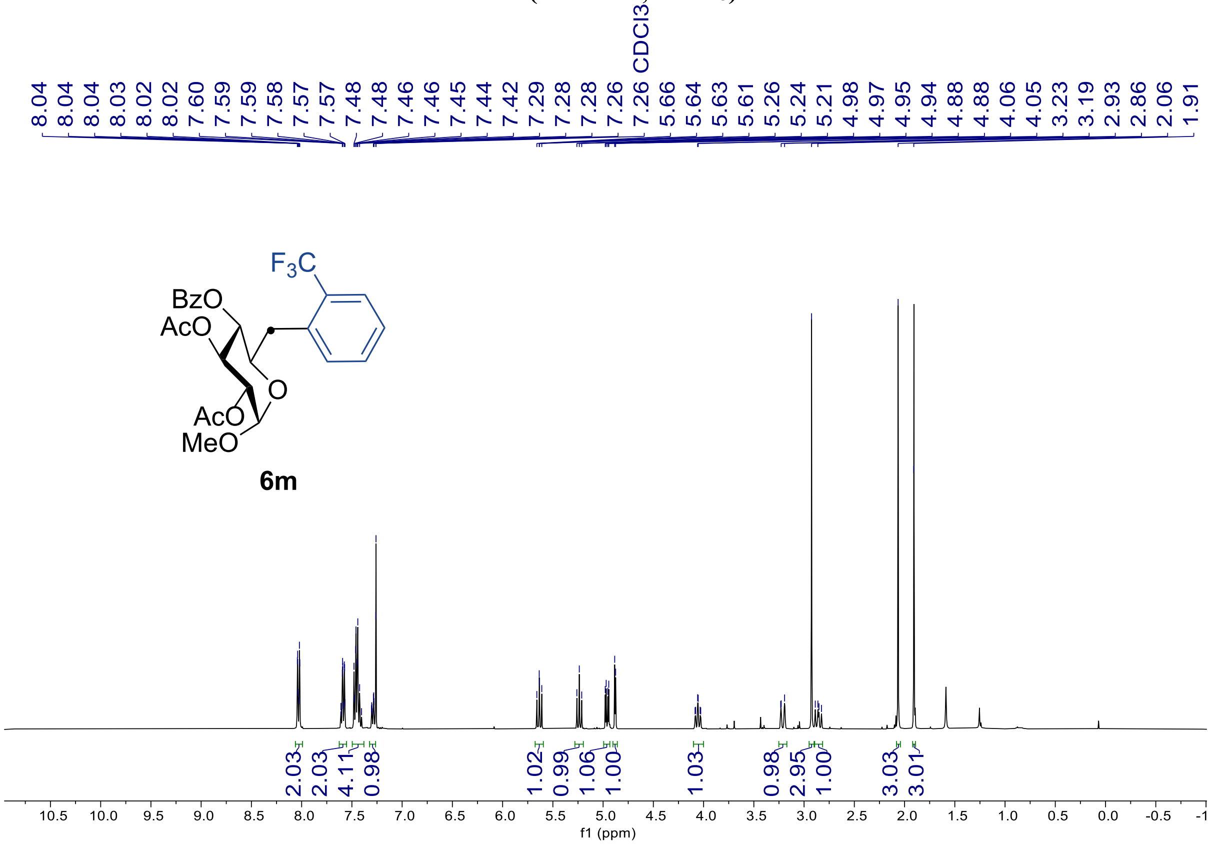

^{1}H NMR (400 MHz, CDCl$_3$) of 6m

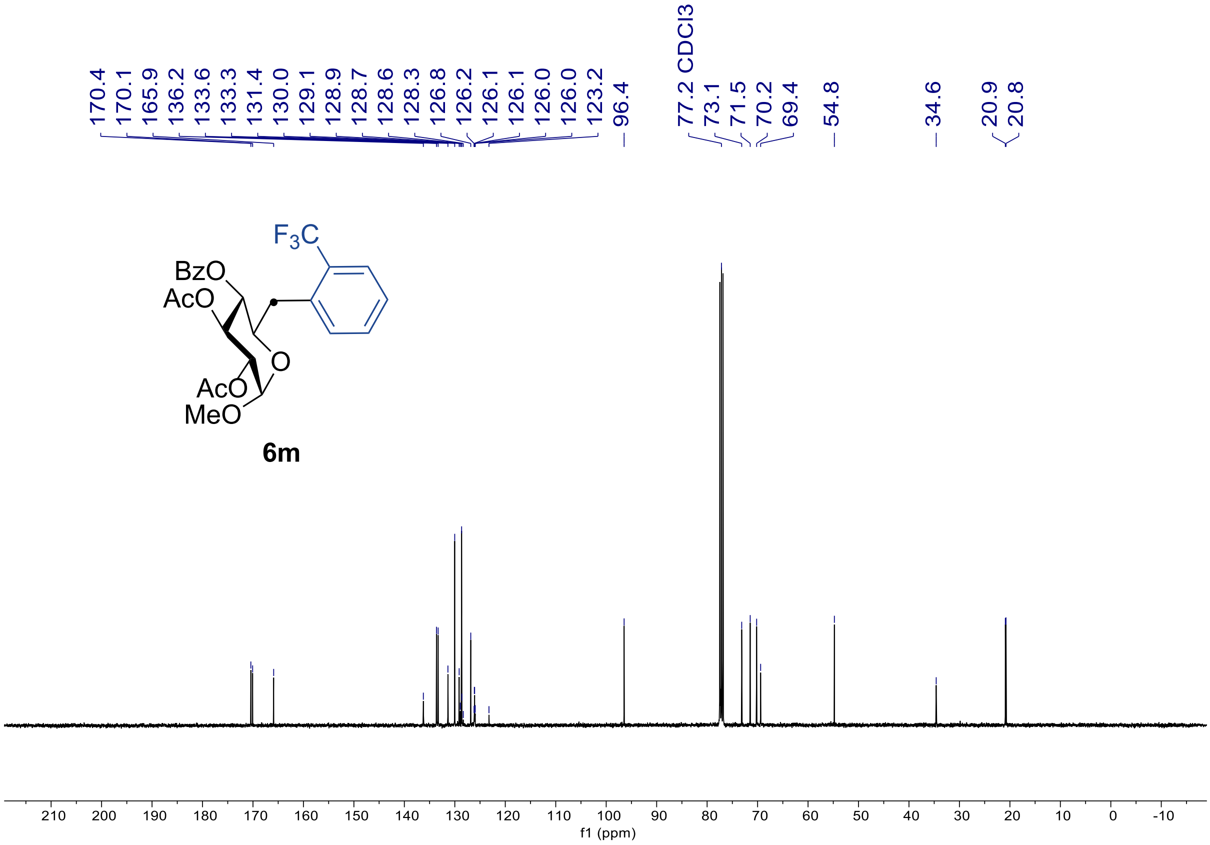

^{13}C NMR (101 MHz, CDCl$_3$) of 6m

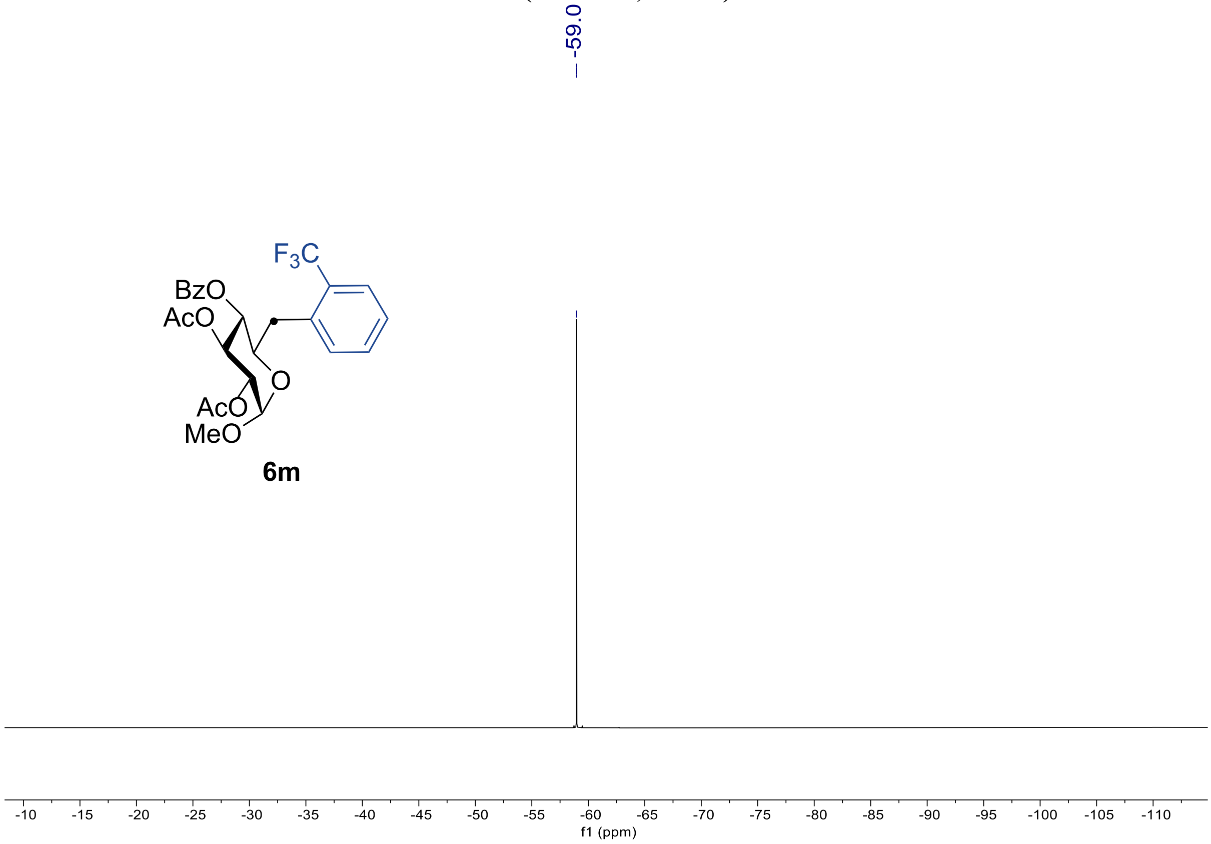

^{19}F NMR (376 MHz, CDCl$_3$) of 6m

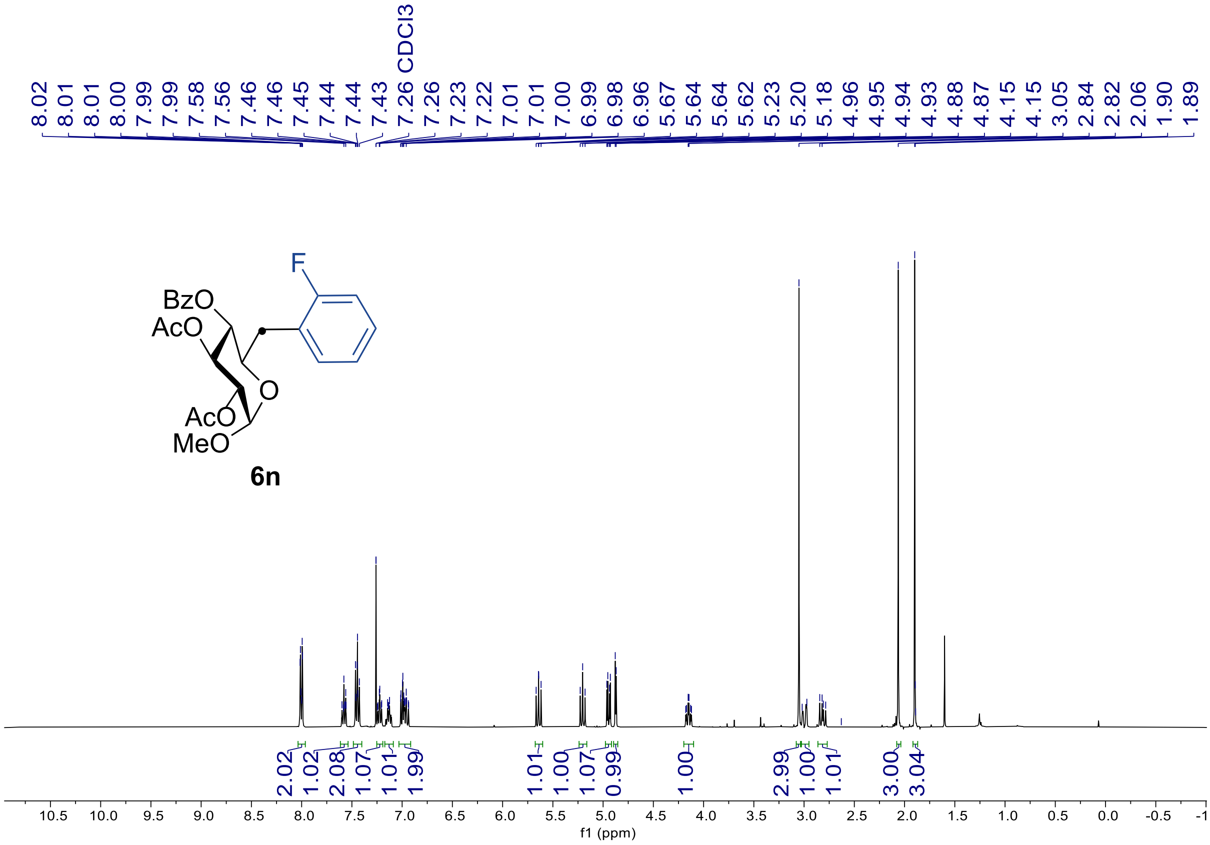

^{1}H NMR (400 MHz, CDCl$_3$) of 6n

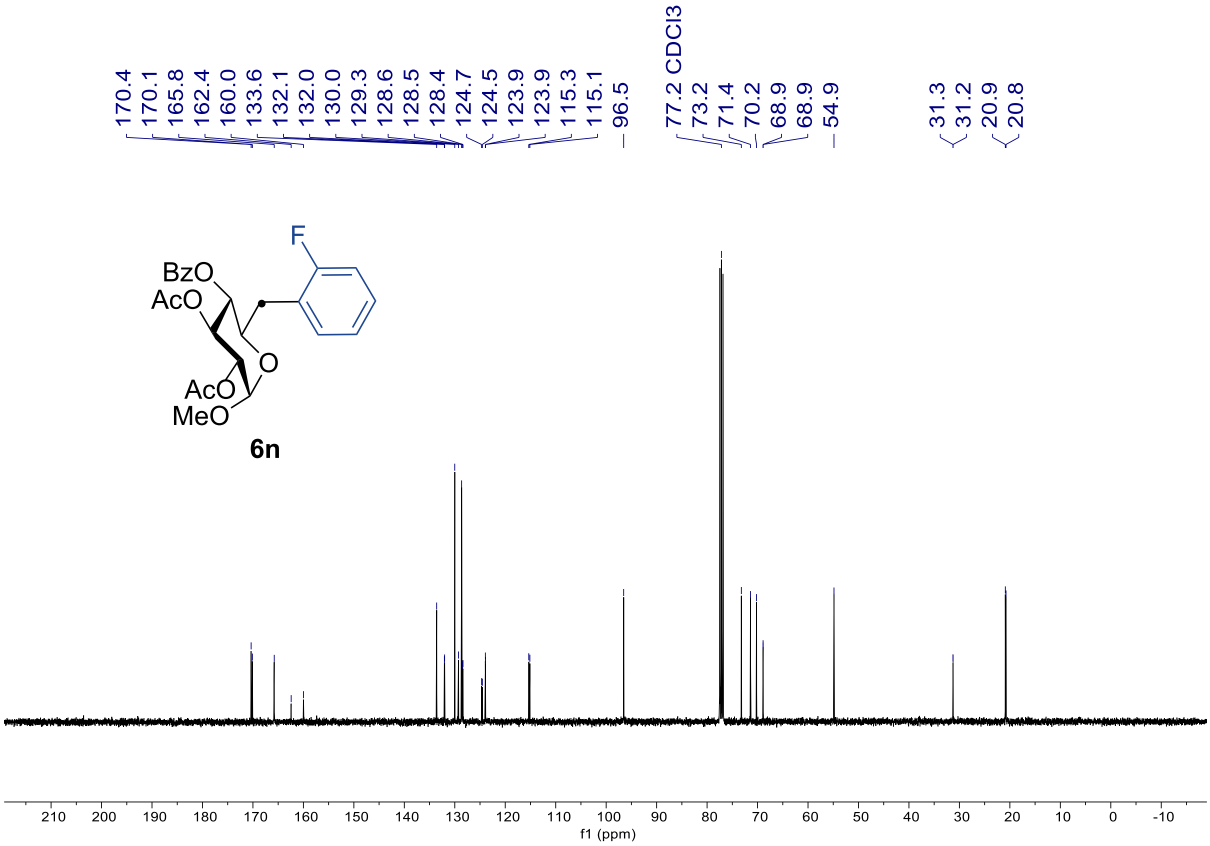

^{13}C NMR (101 MHz, CDCl$_3$) of 6n

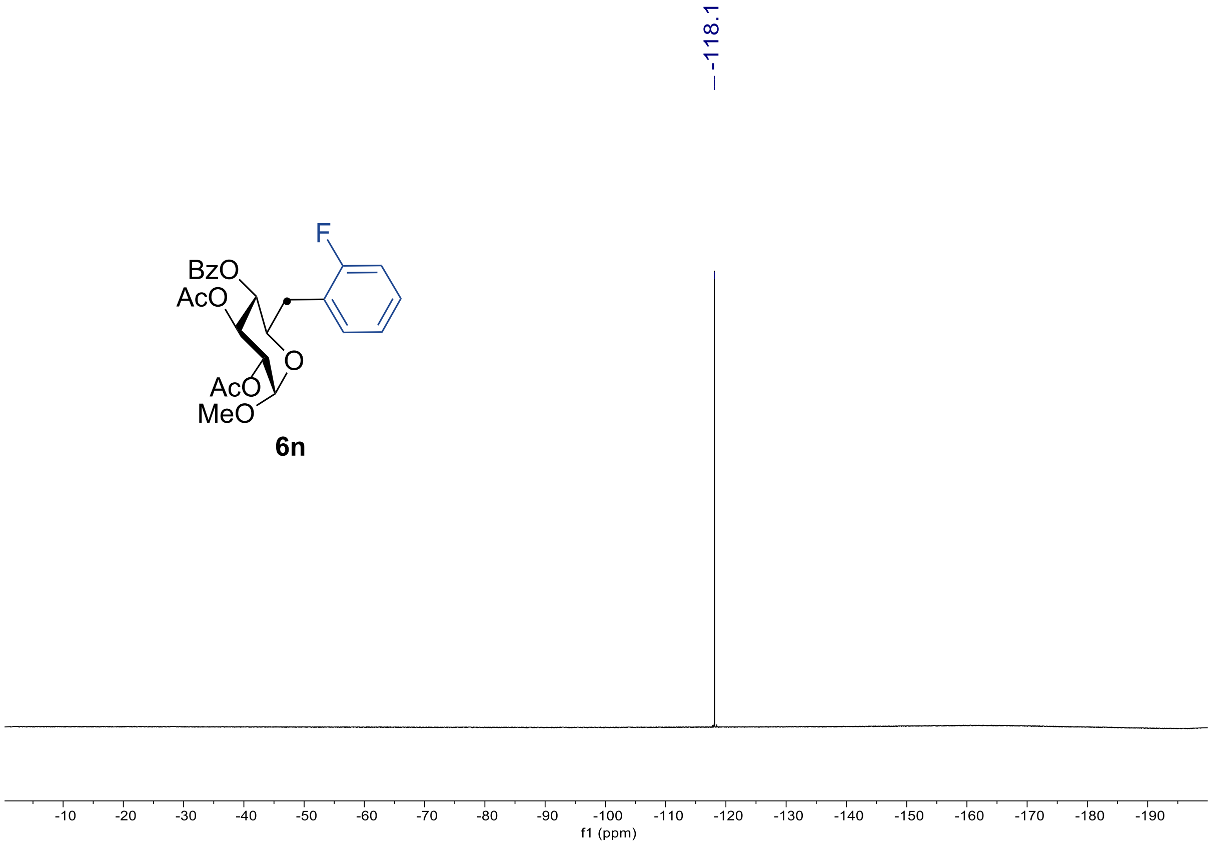

^{19}F NMR (376 MHz, CDCl$_3$) of 6n

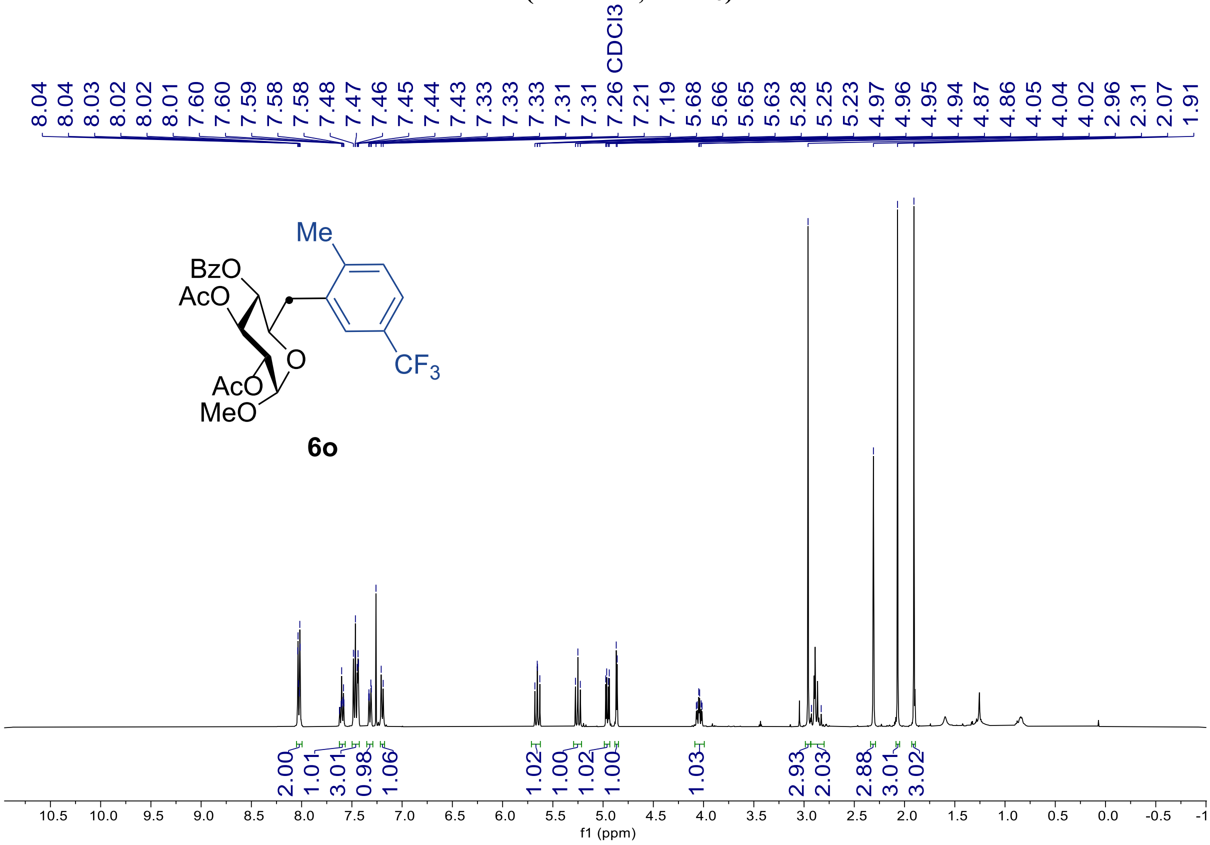

^{1}H NMR (400 MHz, CDCl$_3$) of 6o

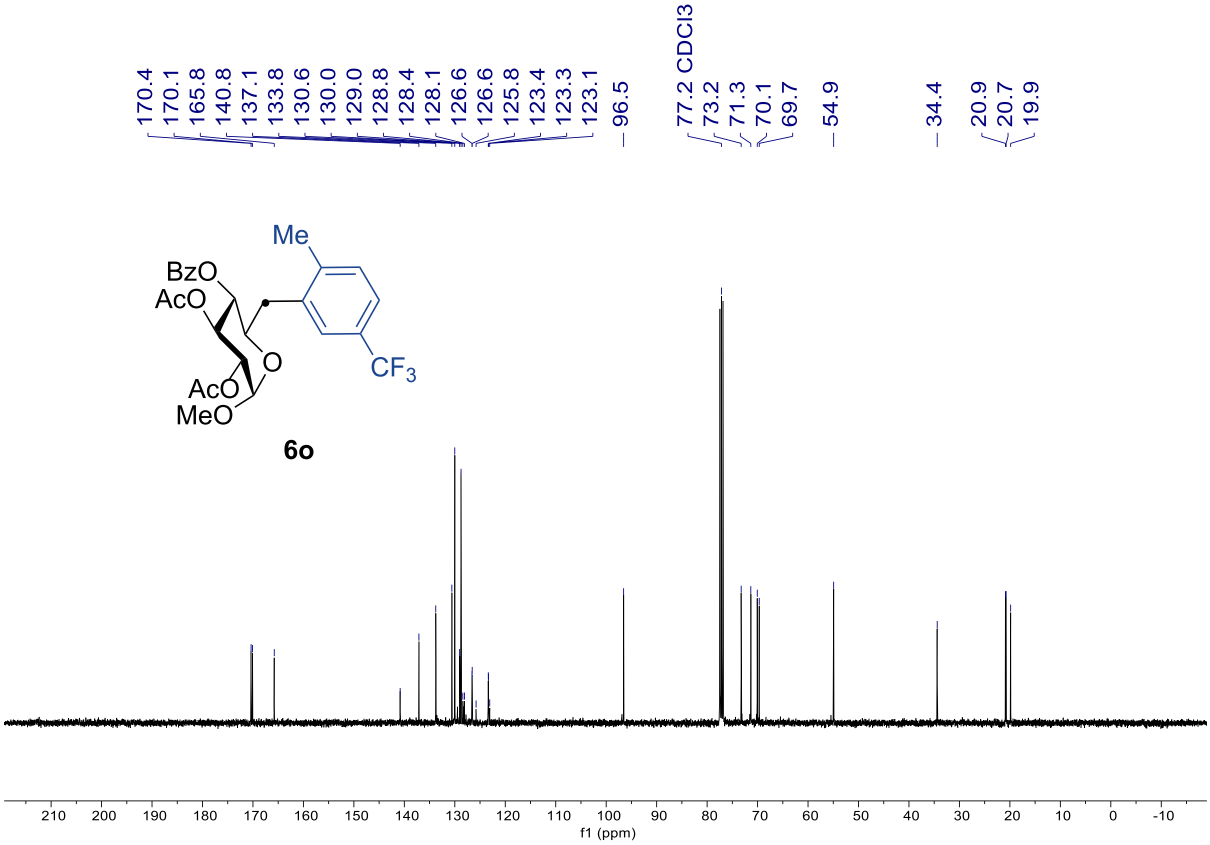

^{13}C NMR (101 MHz, CDCl$_3$) of 6o

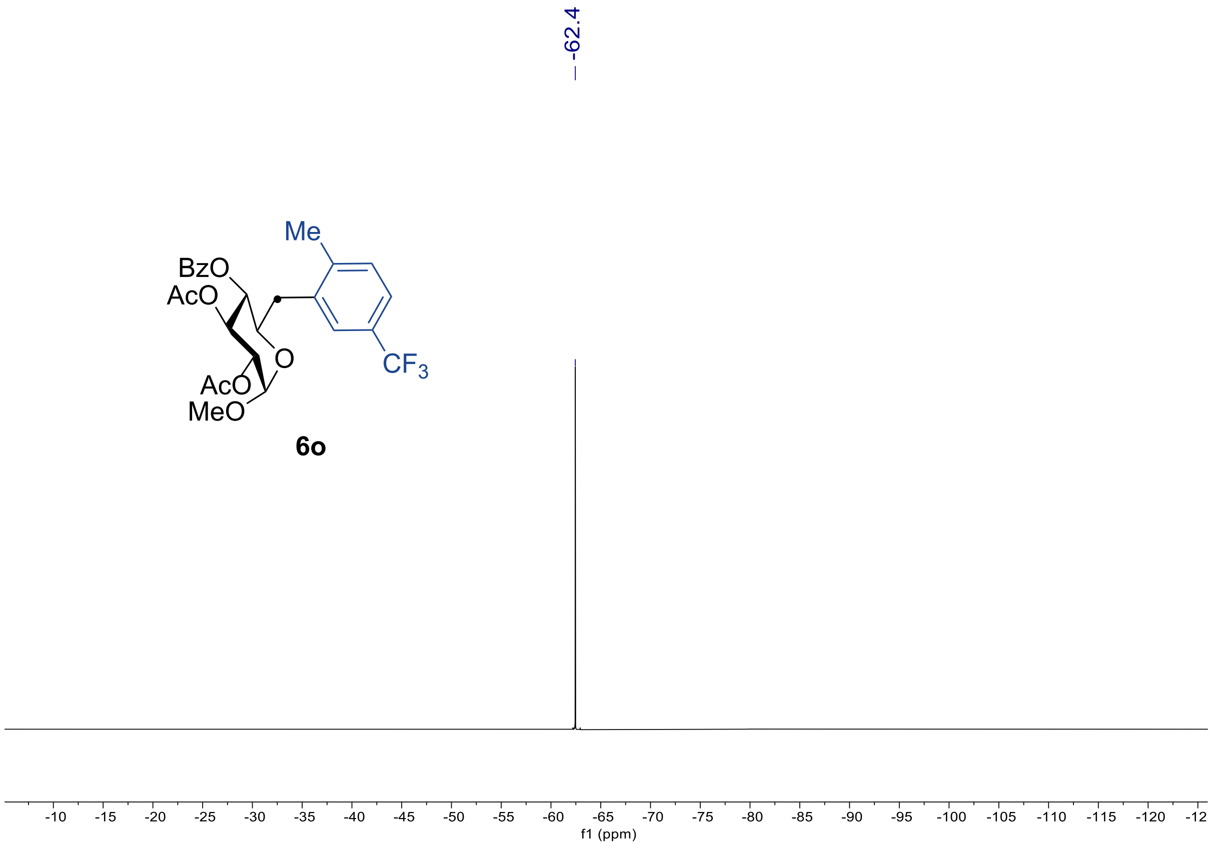

^{19}F NMR (376 MHz, CDCl$_3$) of 6o

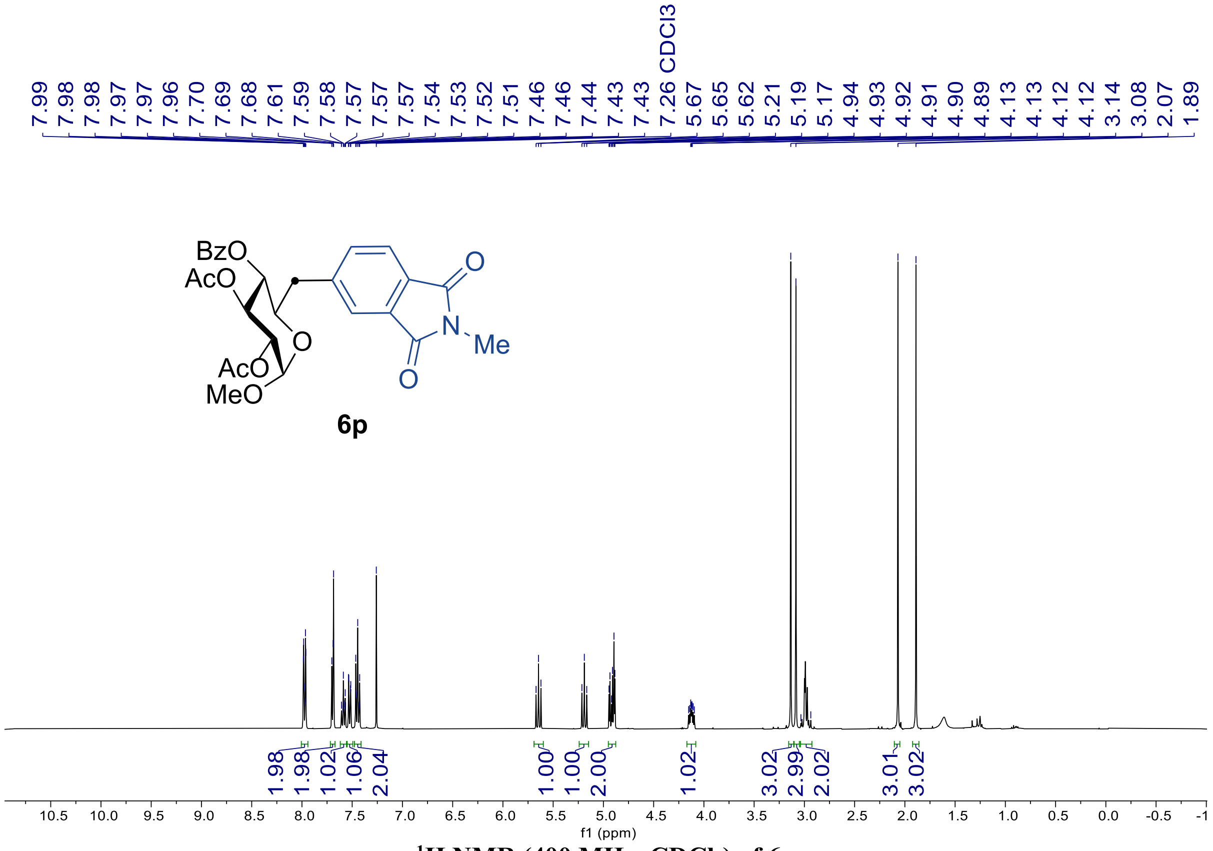

^{1}H NMR (400 MHz, $CDCl_3$) of 6p

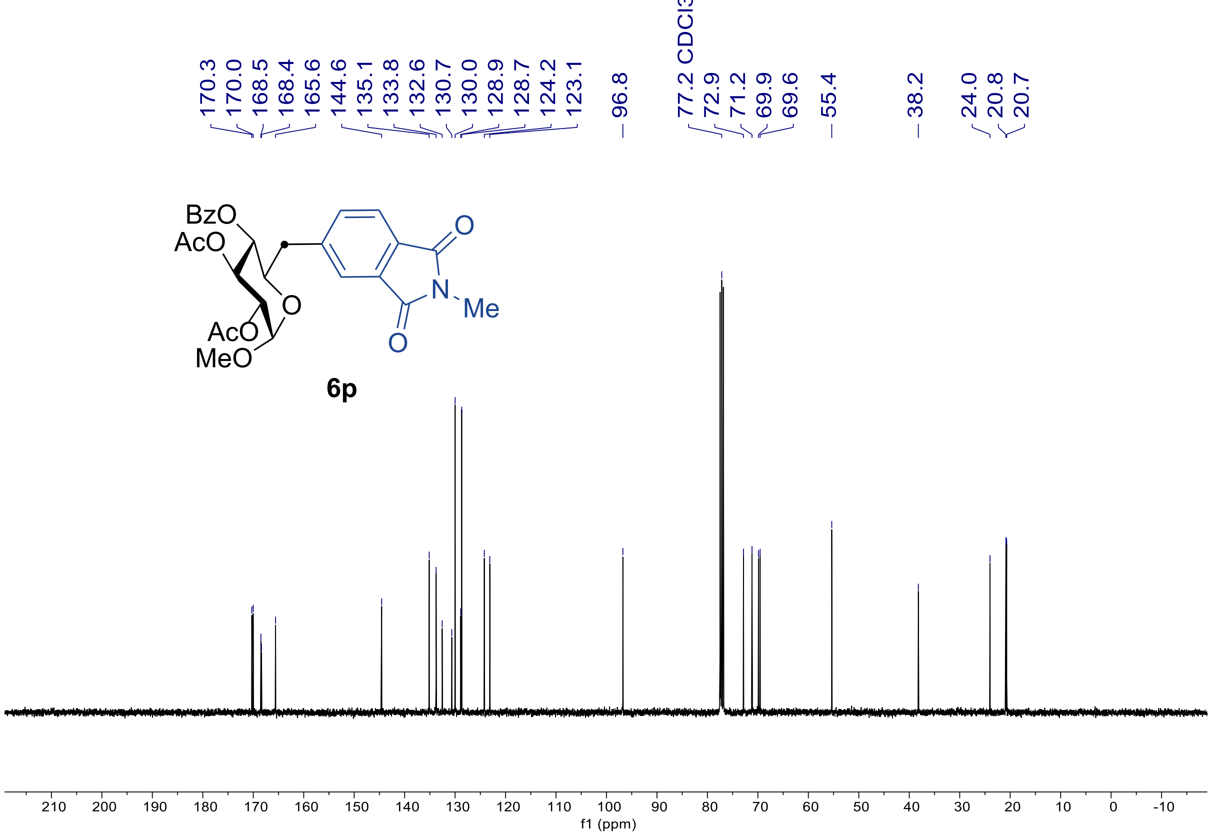

^{13}C NMR (101 MHz, $CDCl_3$) of 6p

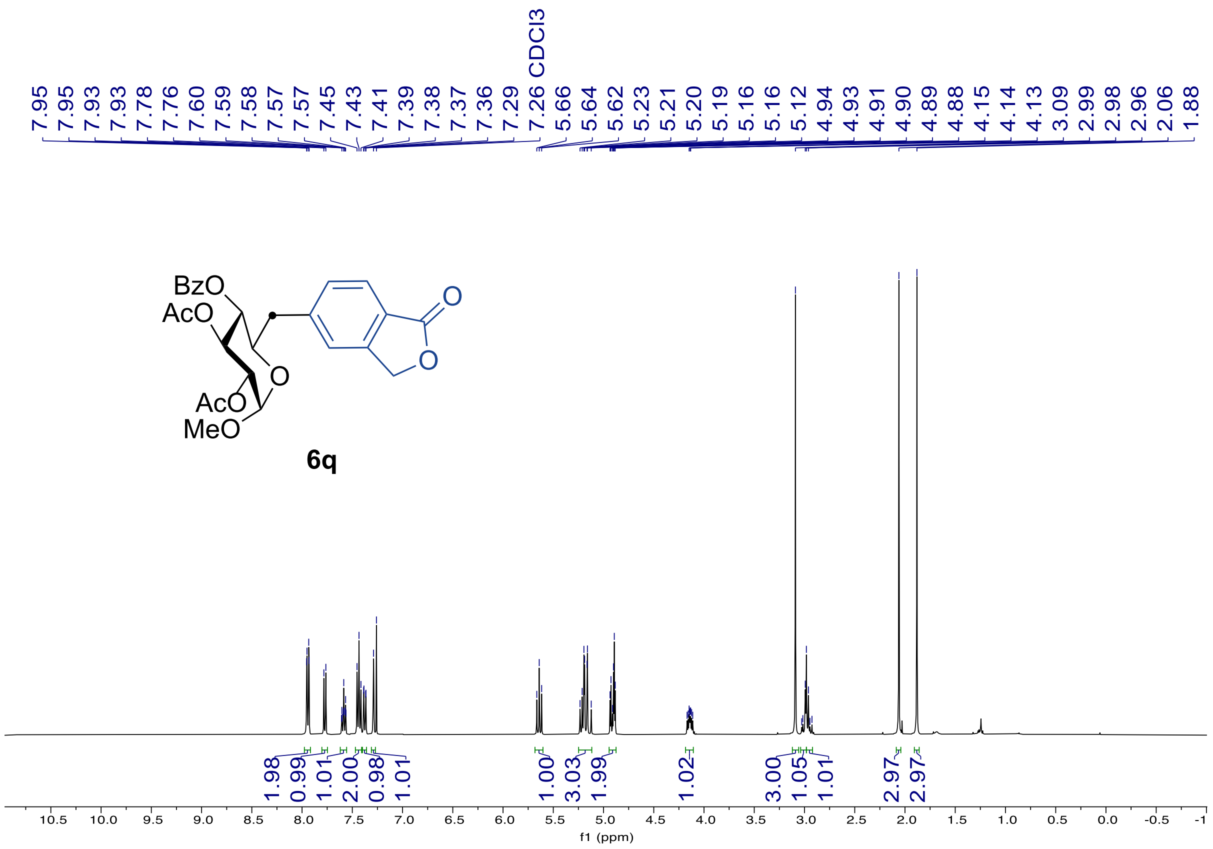

^{1}H NMR (400 MHz, $CDCl_3$) of 6q

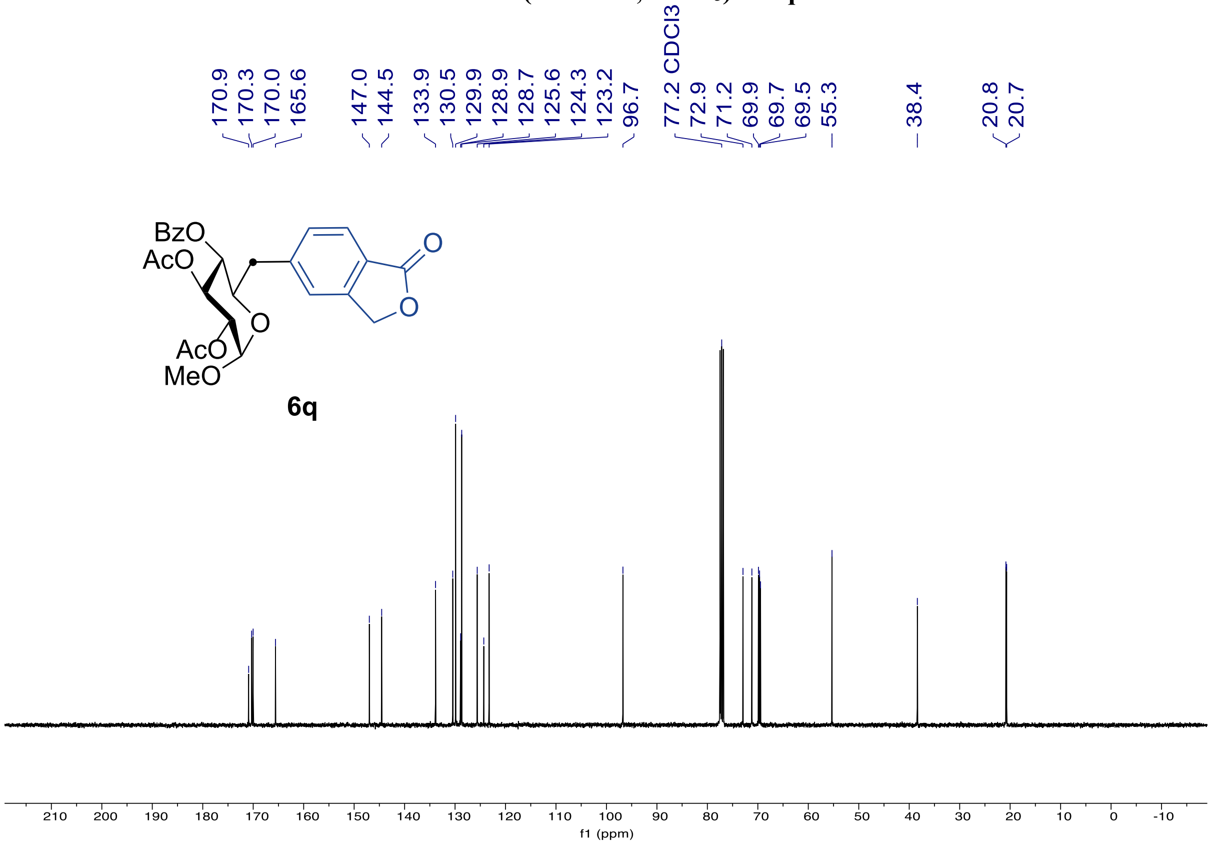

^{13}C NMR (101 MHz, $CDCl_3$) of 6q

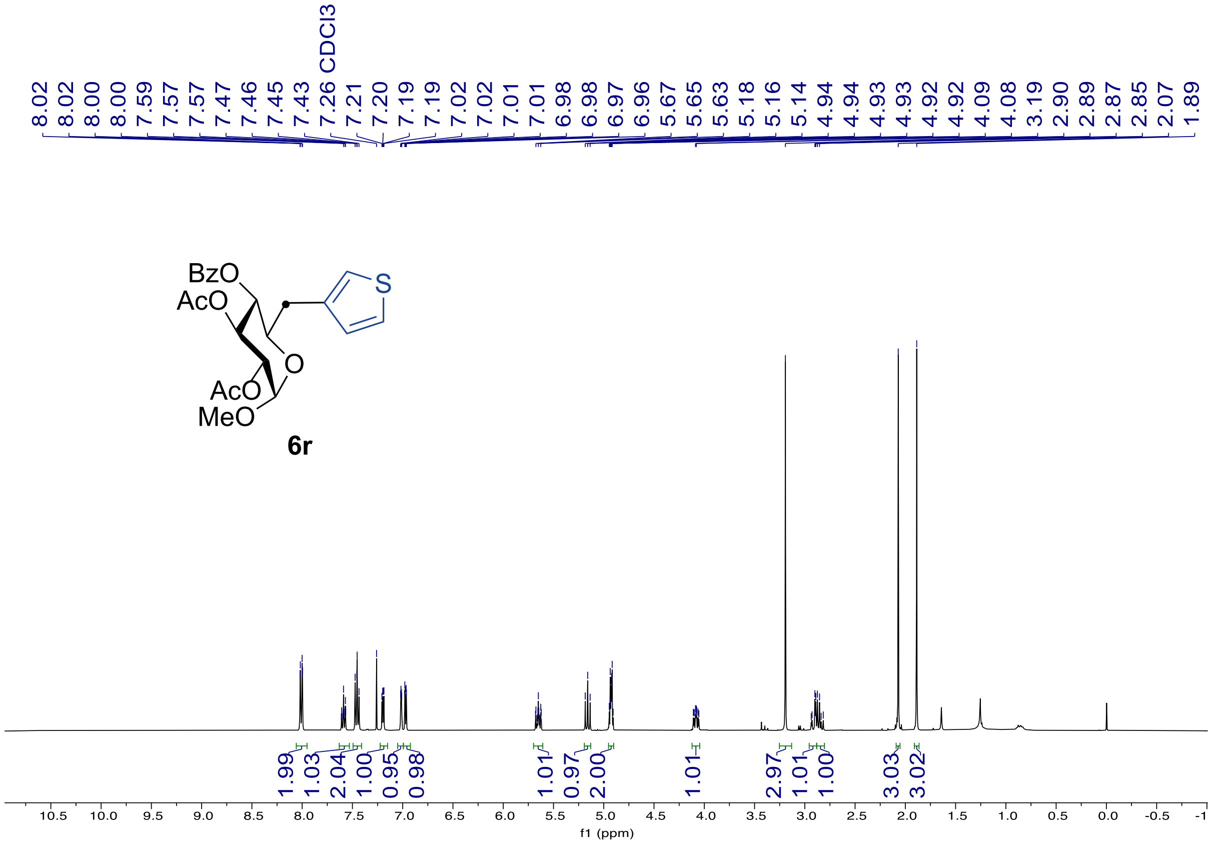

^{1}H NMR (400 MHz, CDCl$_3$) of 6r

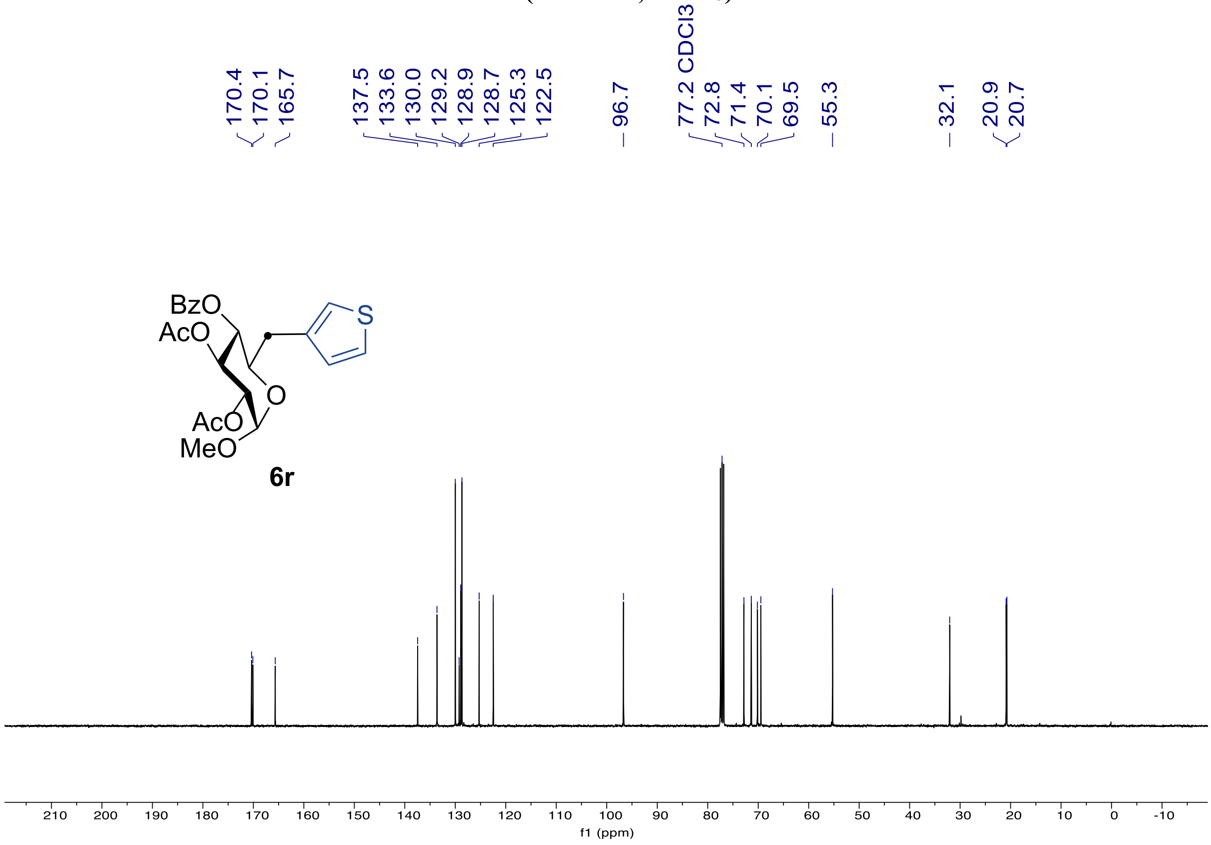

^{13}C NMR (101 MHz, CDCl$_3$) of 6r

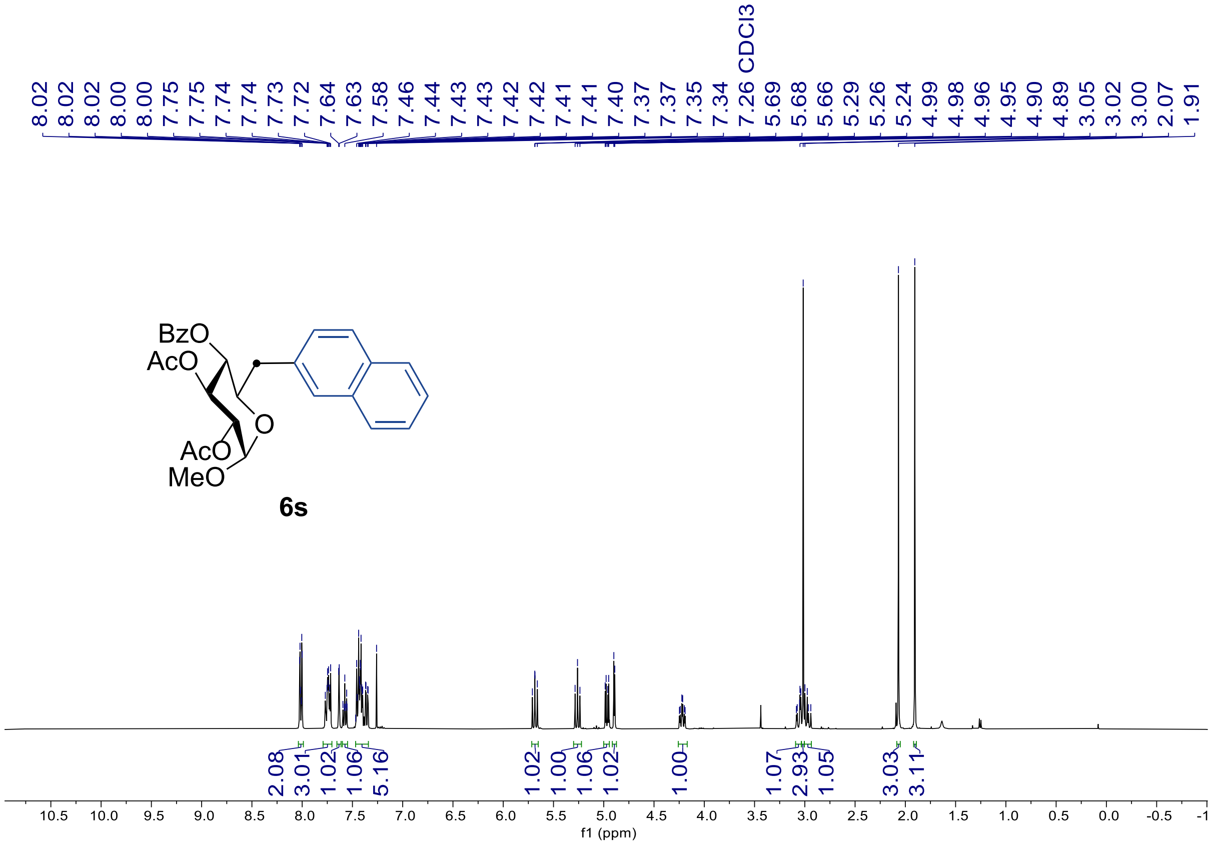

^{1}H NMR (400 MHz, CDCl3) of 6s

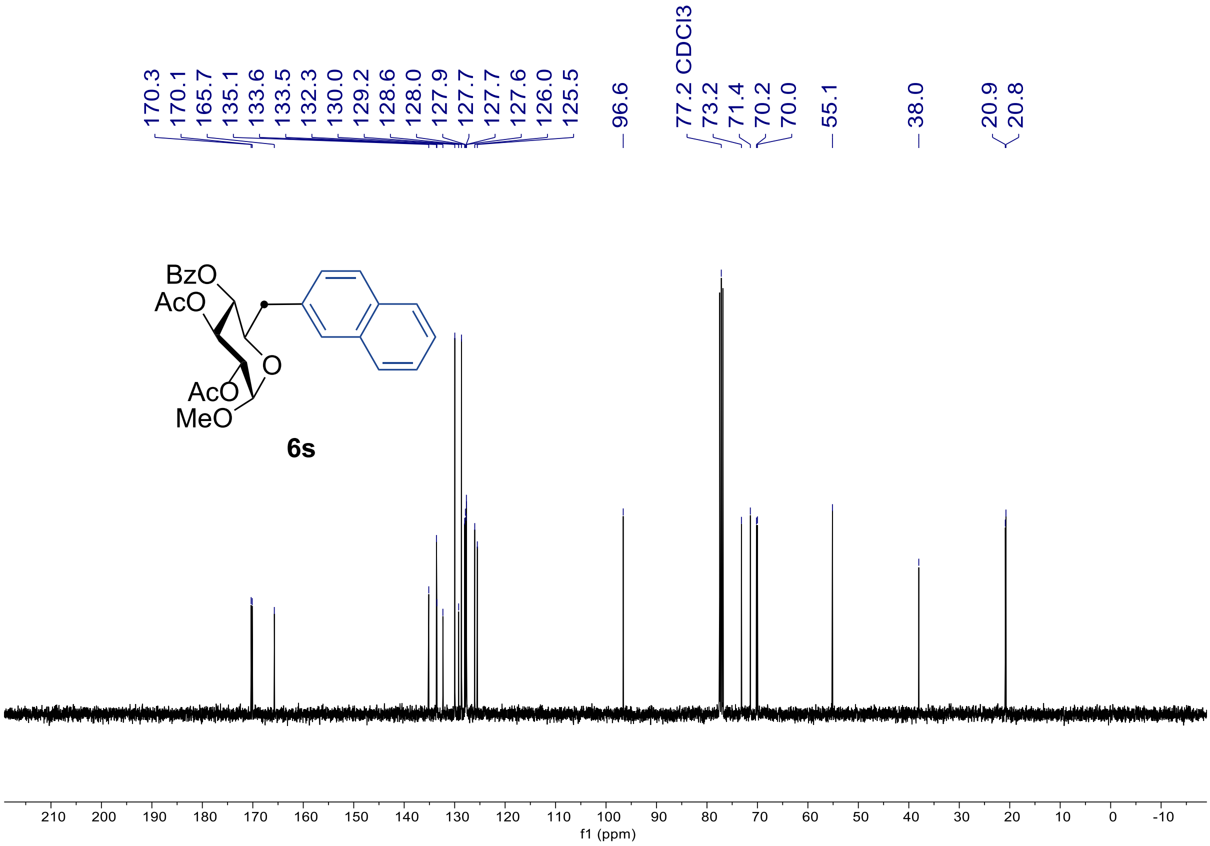

^{13}C NMR (101 MHz, CDCl3) of 6s

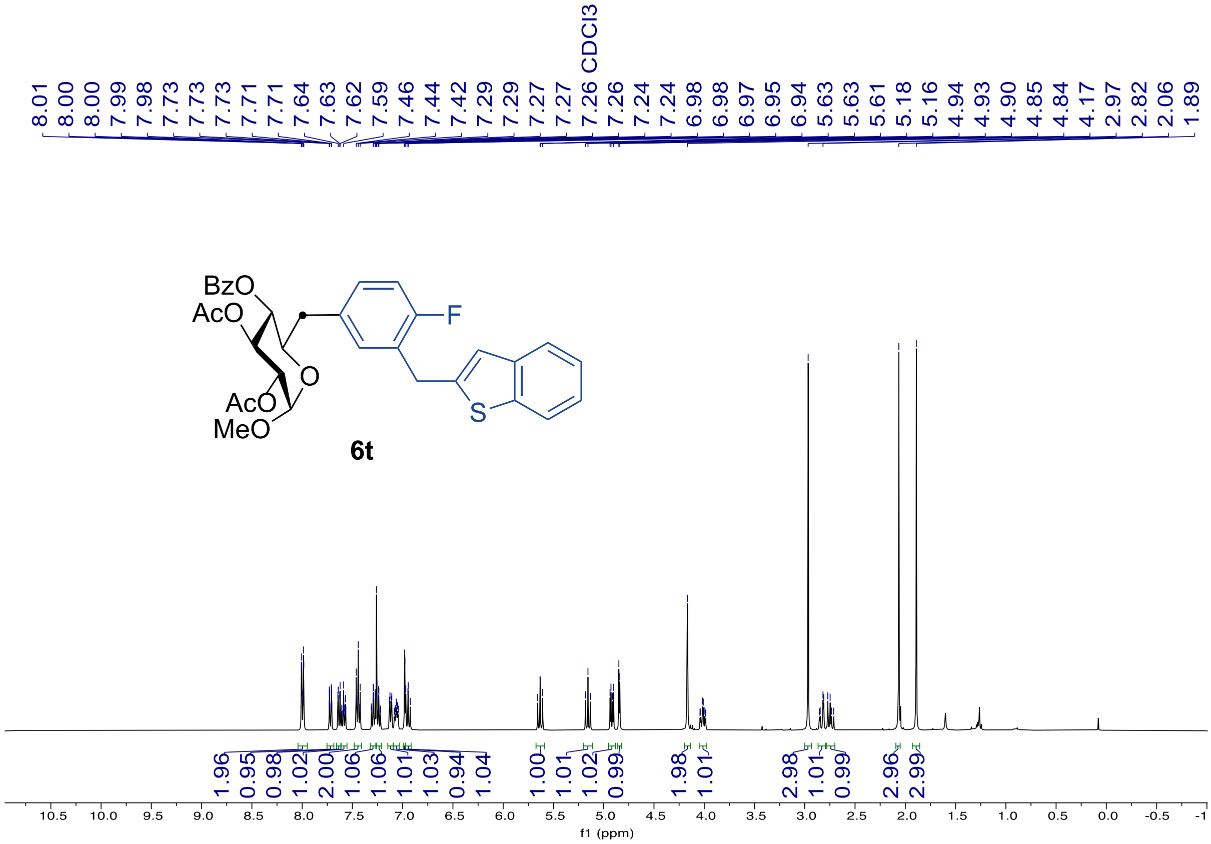

^{1}H NMR (400 MHz, $CDCl_3$) of 6t

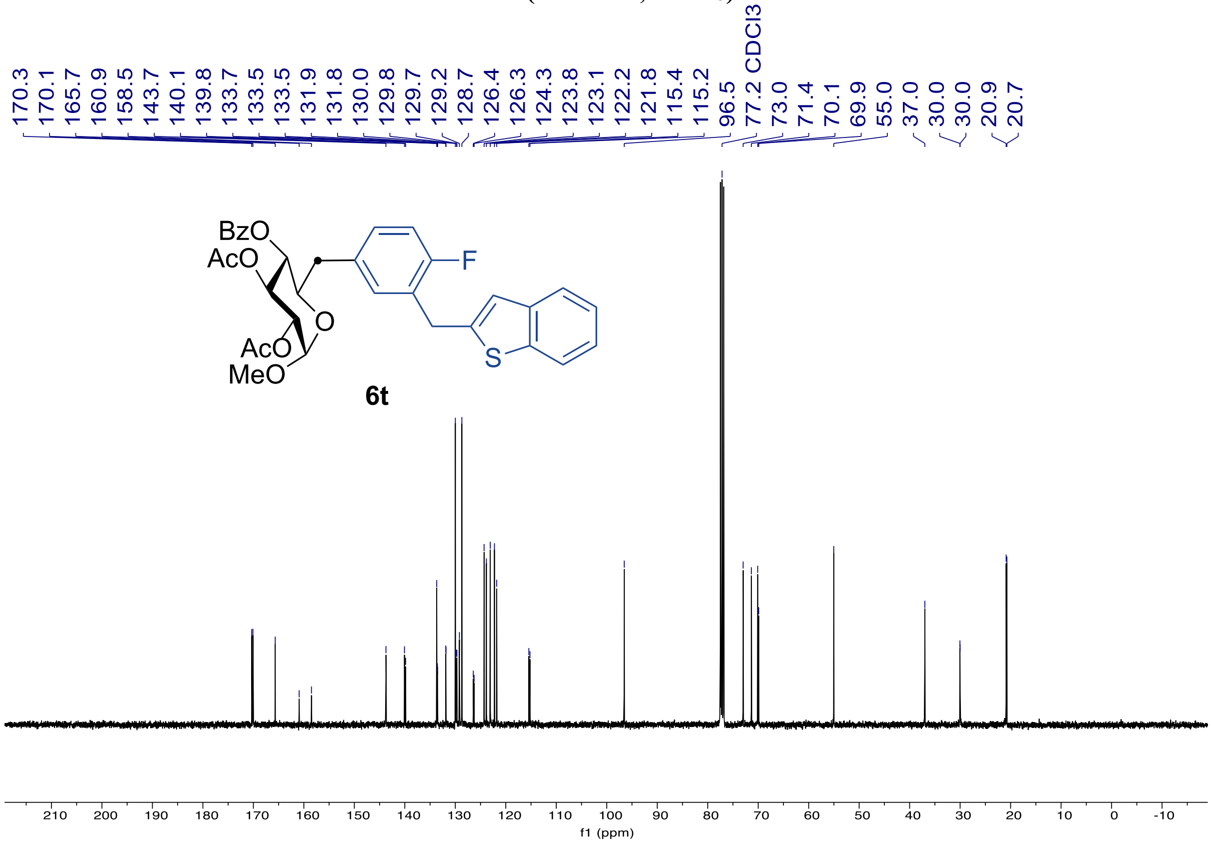

^{13}C NMR (101 MHz, $CDCl_3$) of 6t

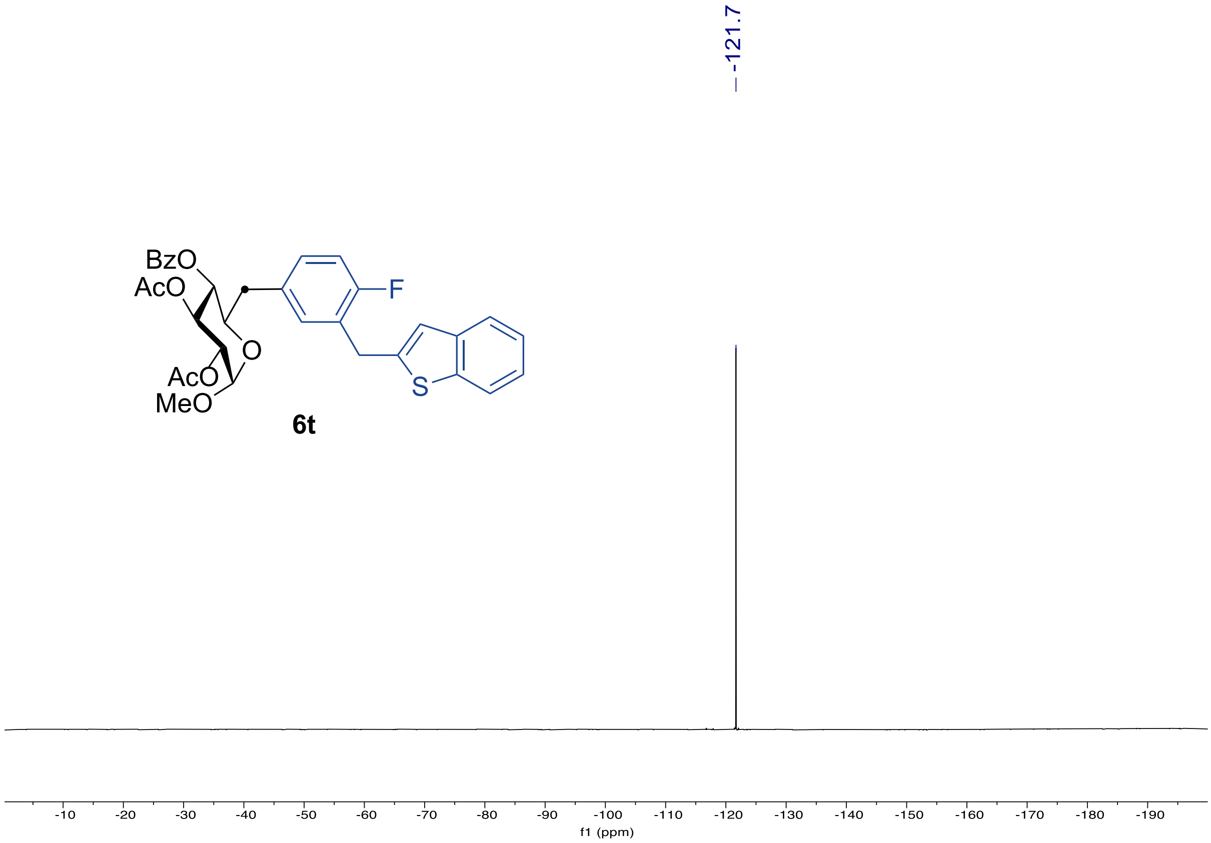

^{19}F NMR (376 MHz, CDCl$_3$) of 6t

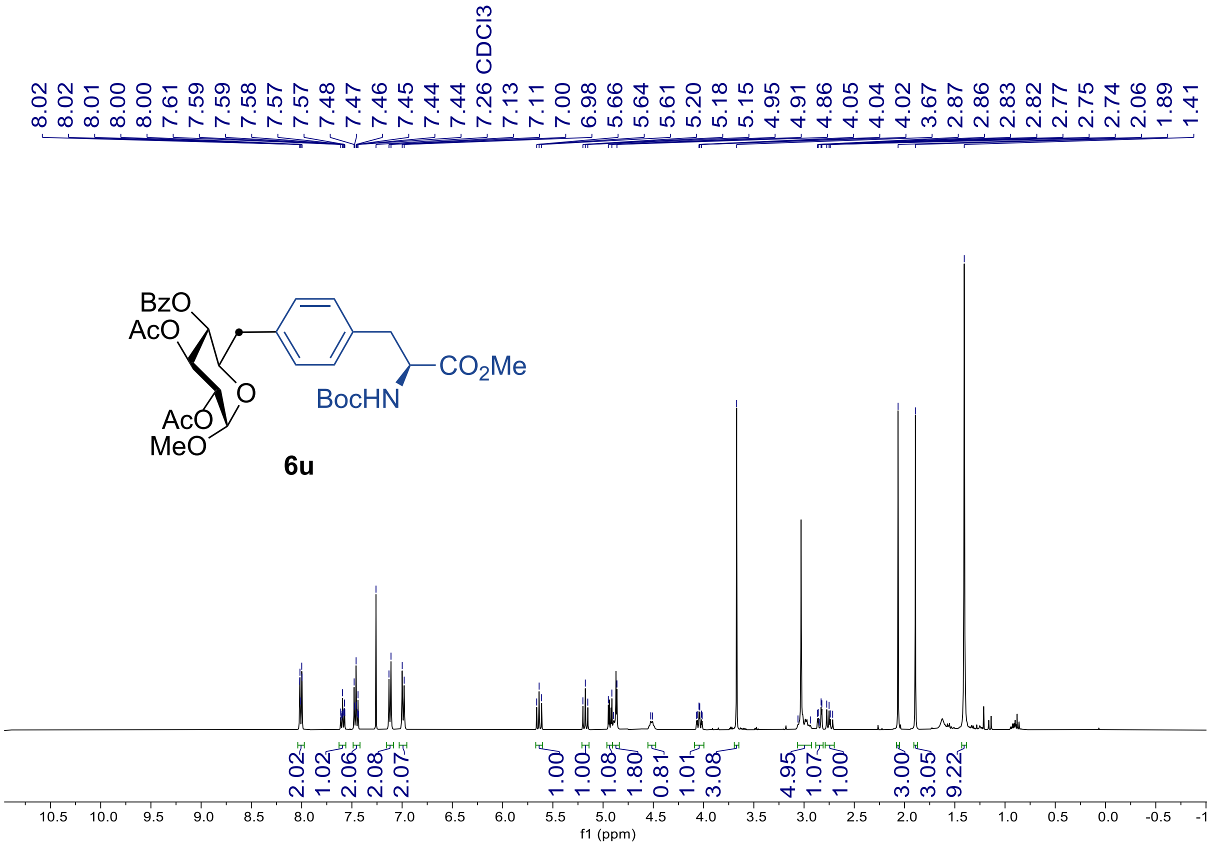

^{1}H NMR (400 MHz, CDCl$_3$) of 6u

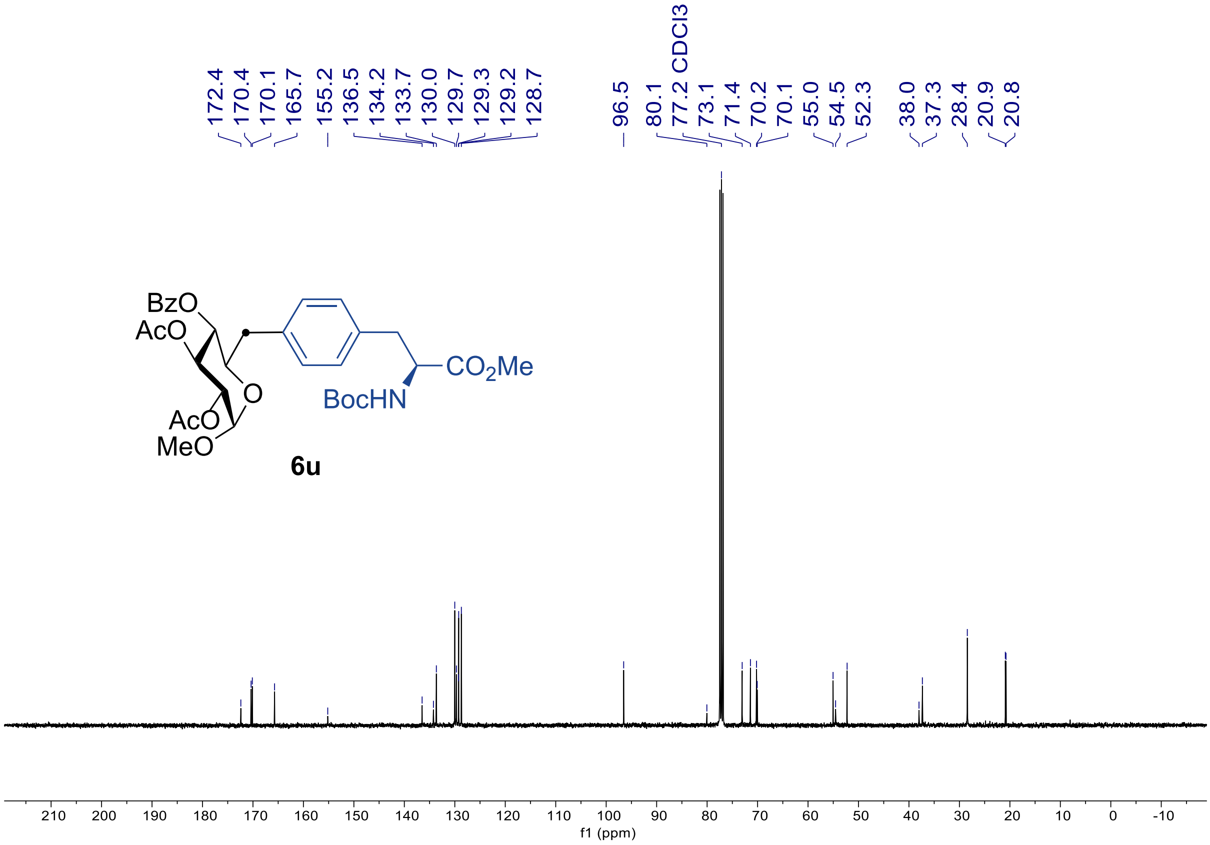

^{13}C NMR (101 MHz, CDCl$_3$) of 6u

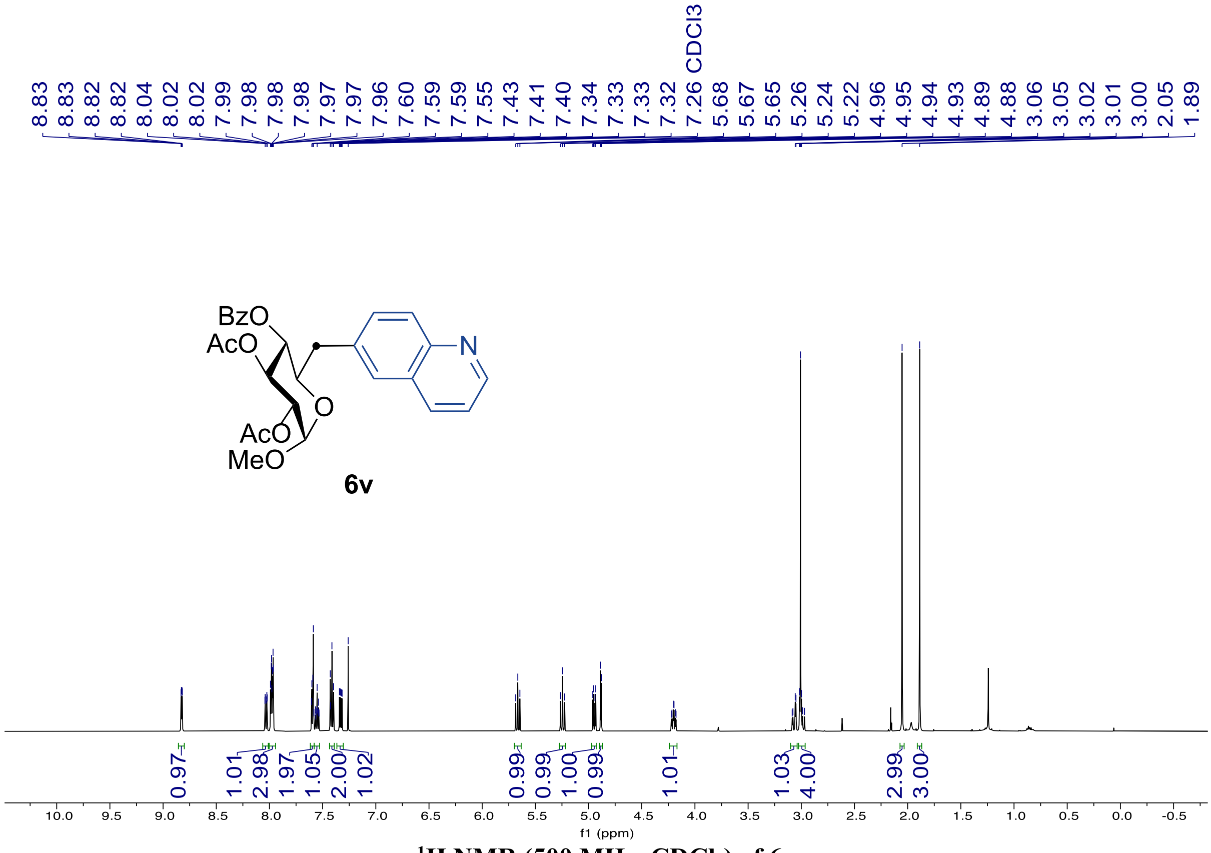

^{1}H NMR (500 MHz, CDCl$_3$) of 6v

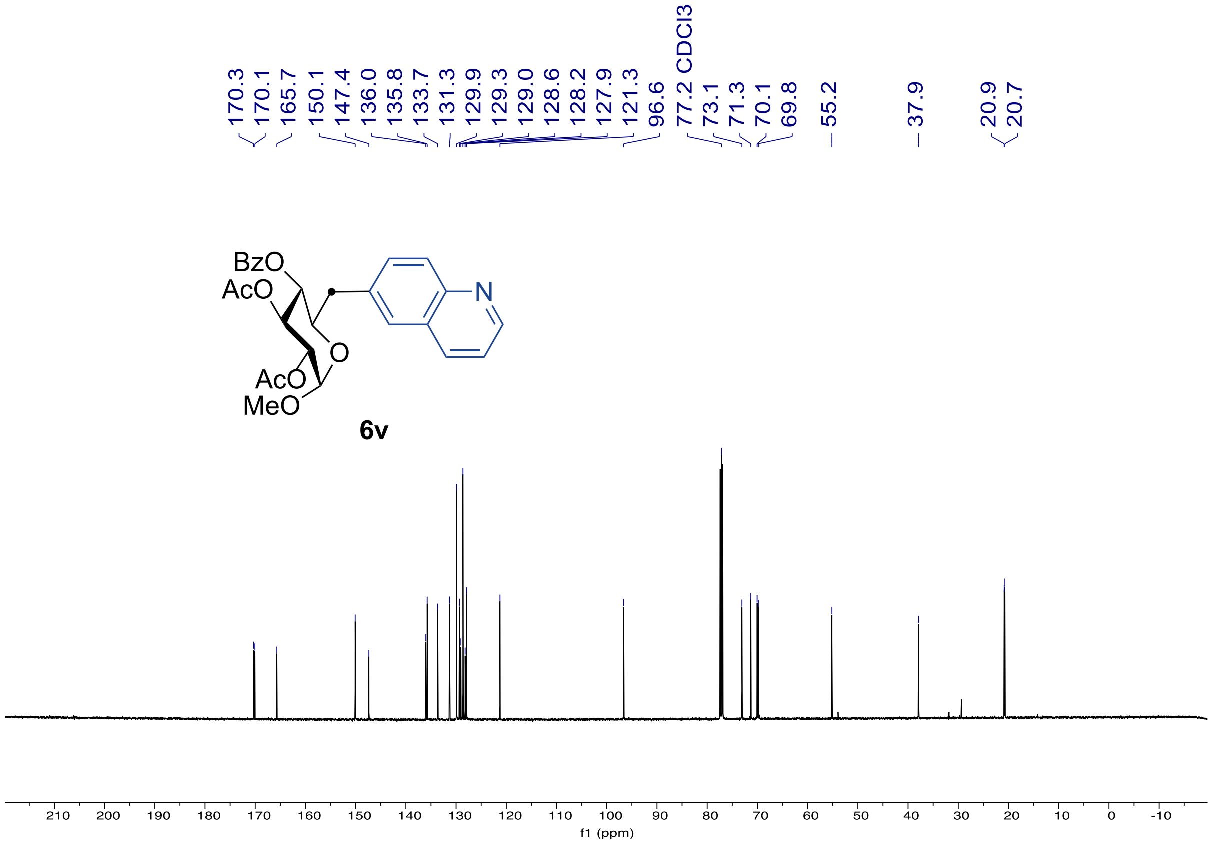

^{13}C NMR (126 MHz, CDCl$_3$) of 6v

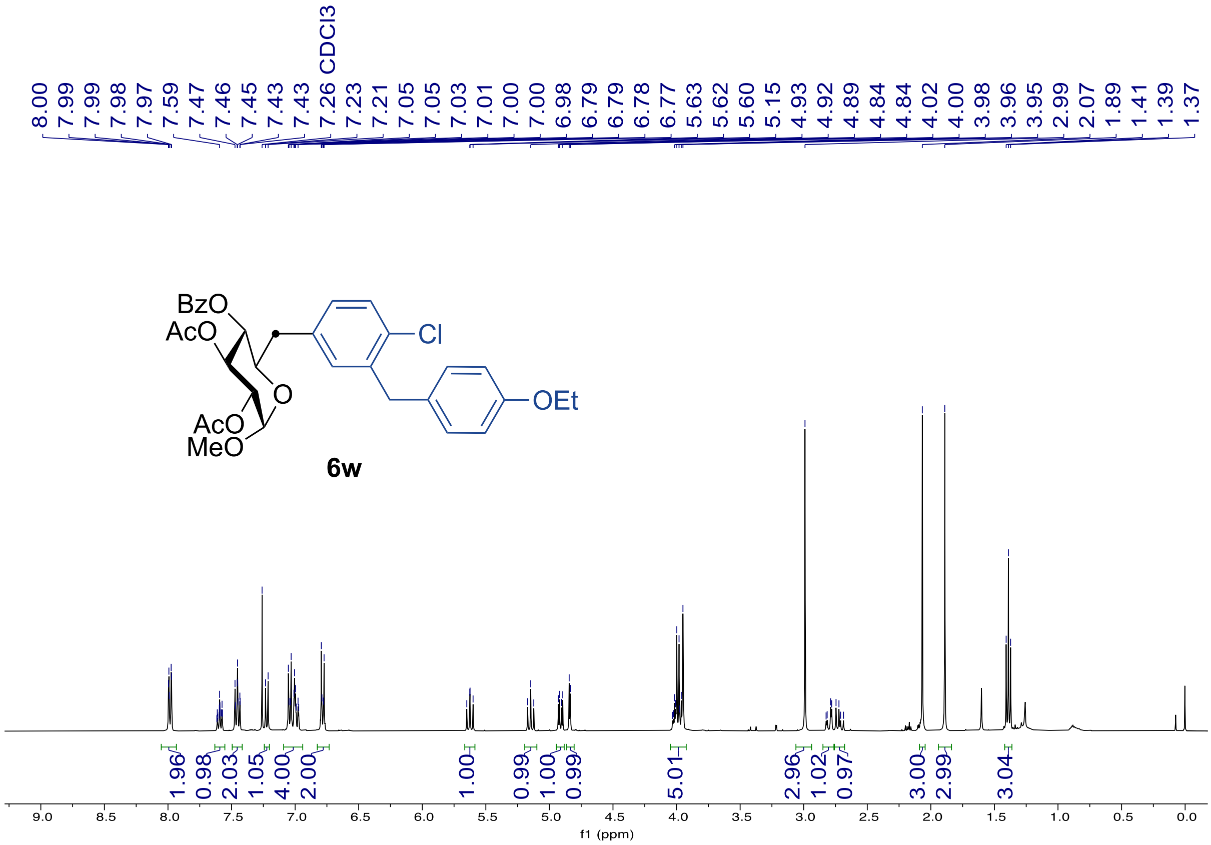

^{1}H NMR (400 MHz, CDCl$_3$) of 6w

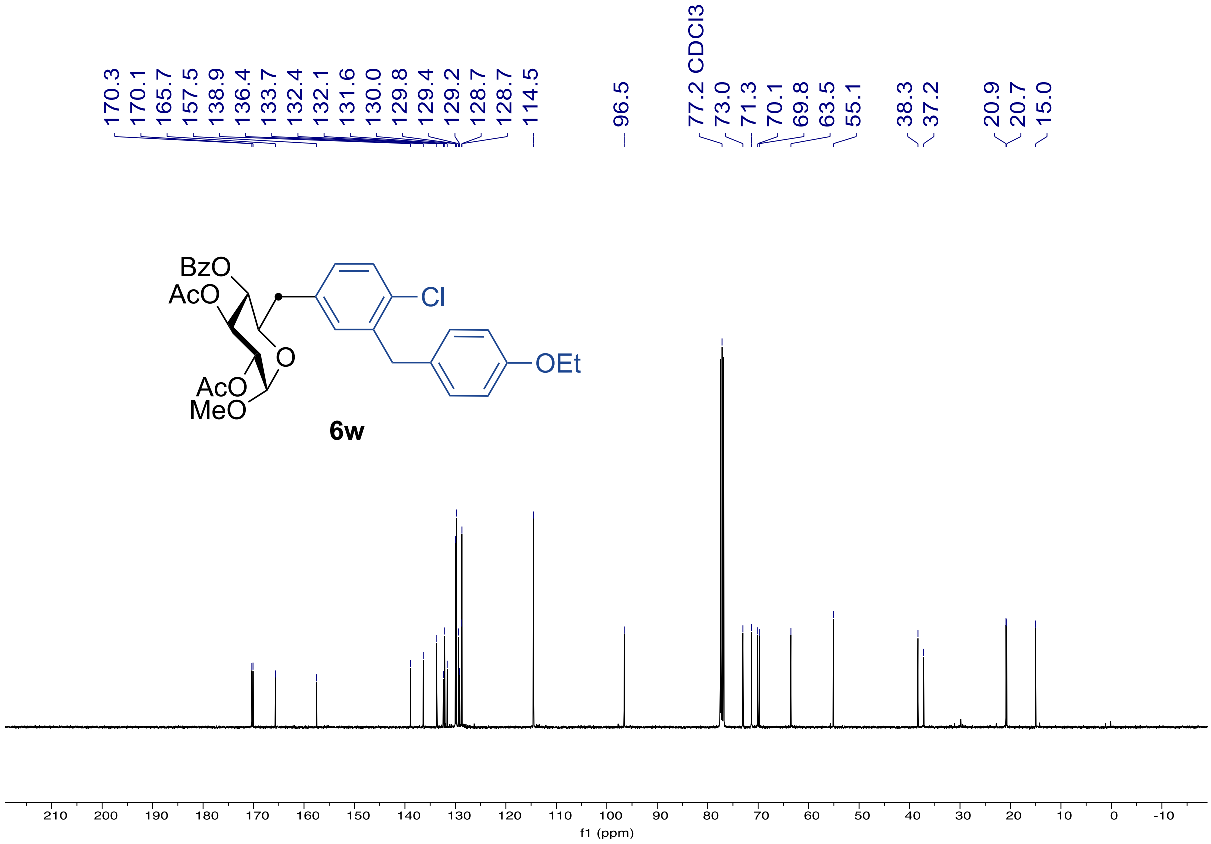

^{13}C NMR (101 MHz, CDCl₃) of 6w

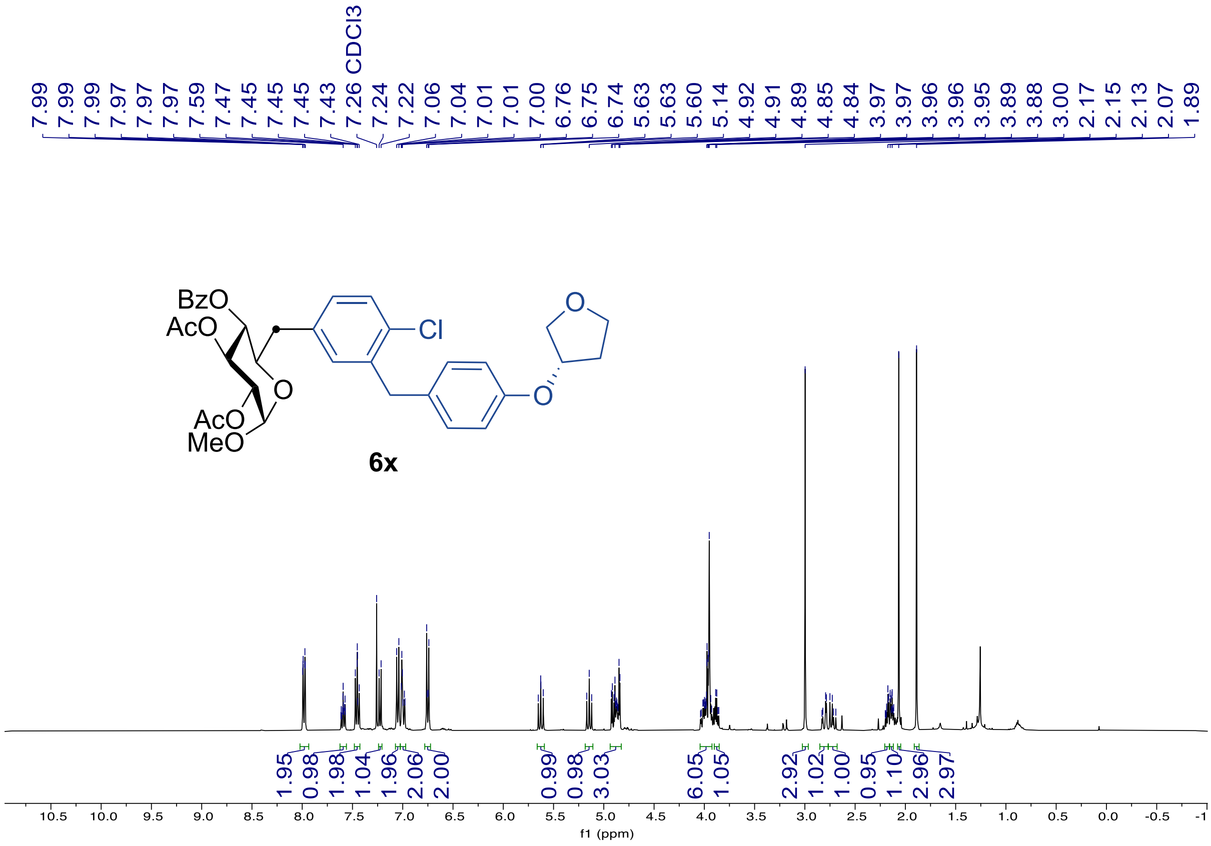

^{1}H NMR (400 MHz, CDCl₃) of 6x

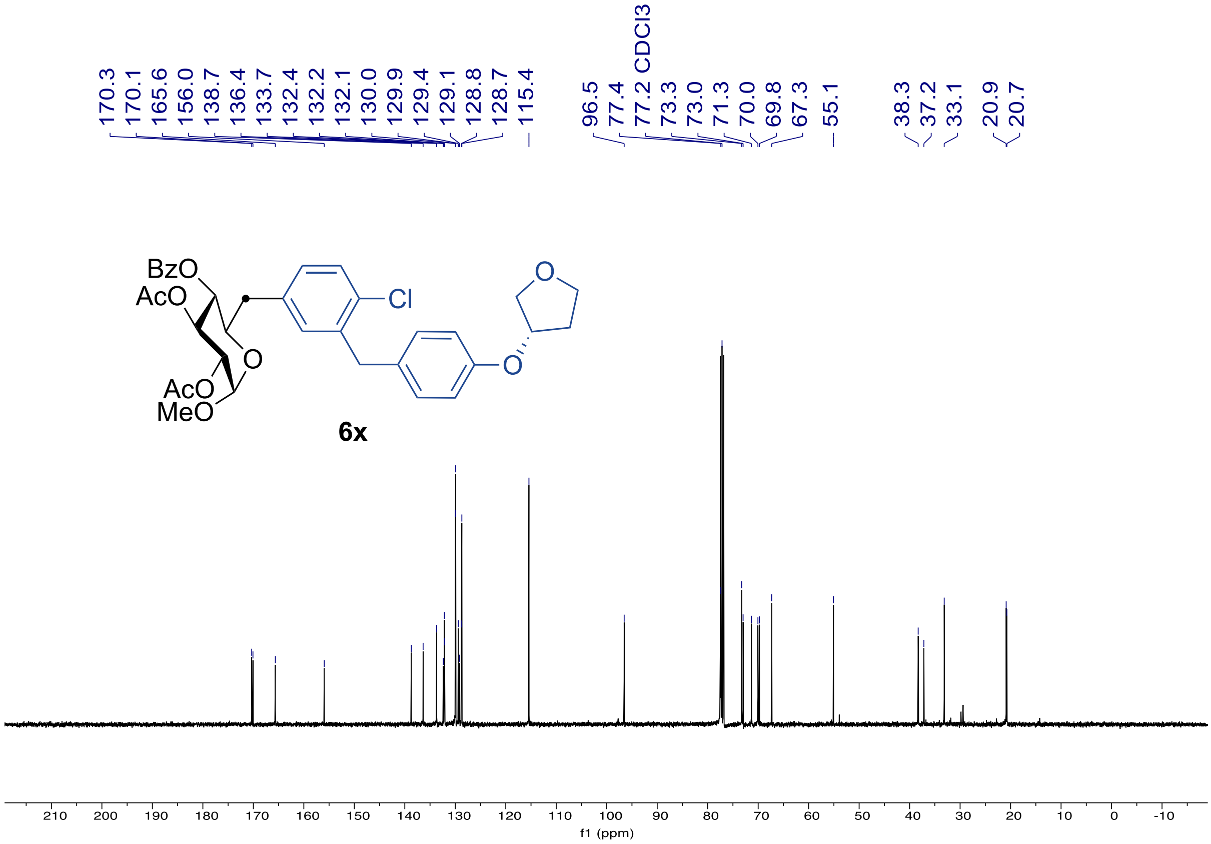

^{13}C NMR (101 MHz, CDCl$_3$) of 6x

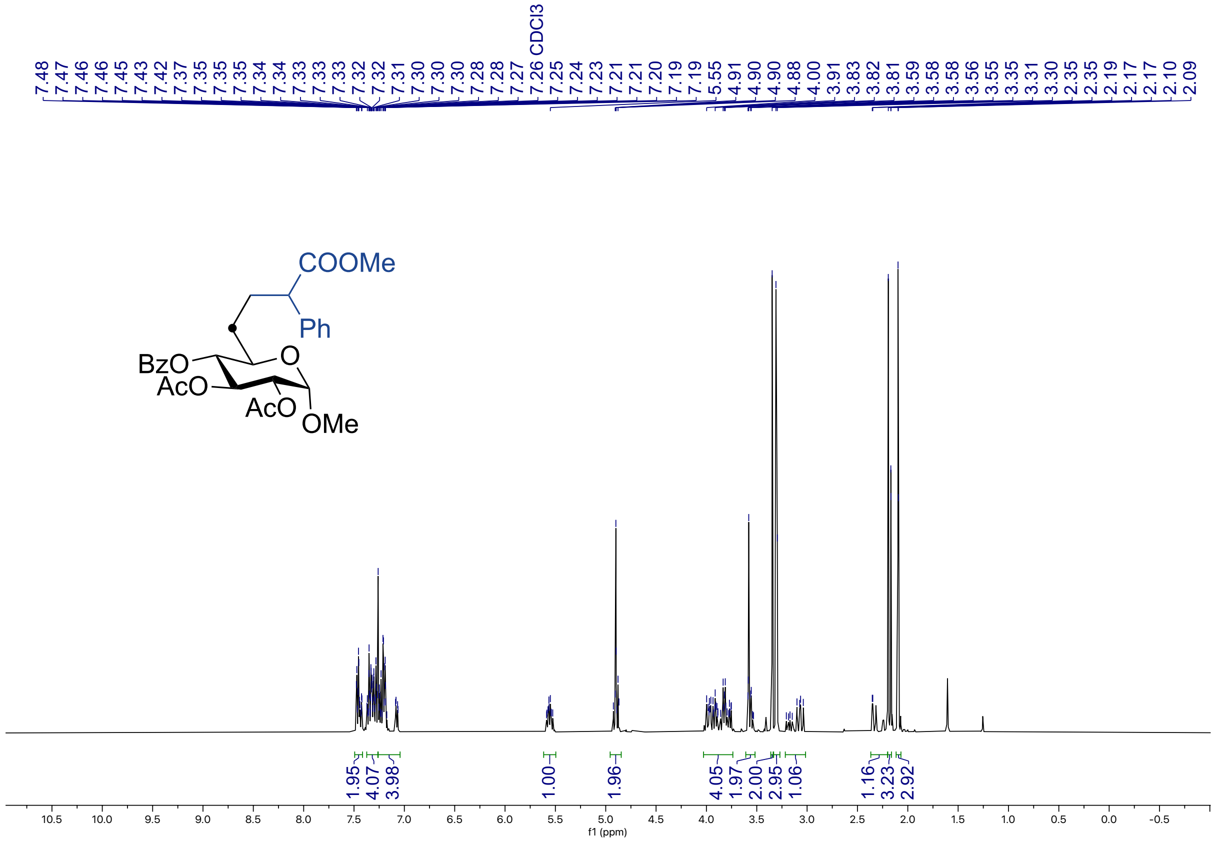

¹H NMR (400 MHz, CDCl₃) of 6y

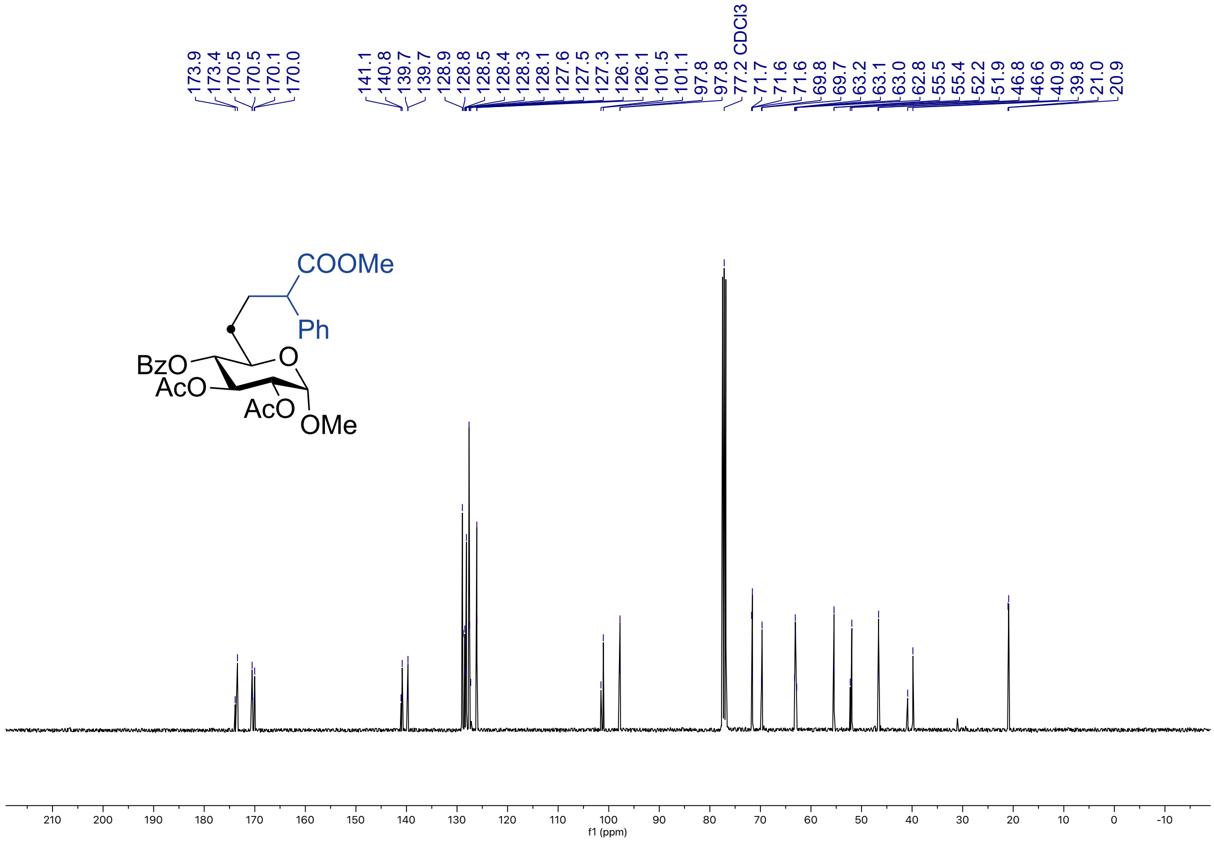

¹³C NMR (126 MHz, CDCl₃) of 6y

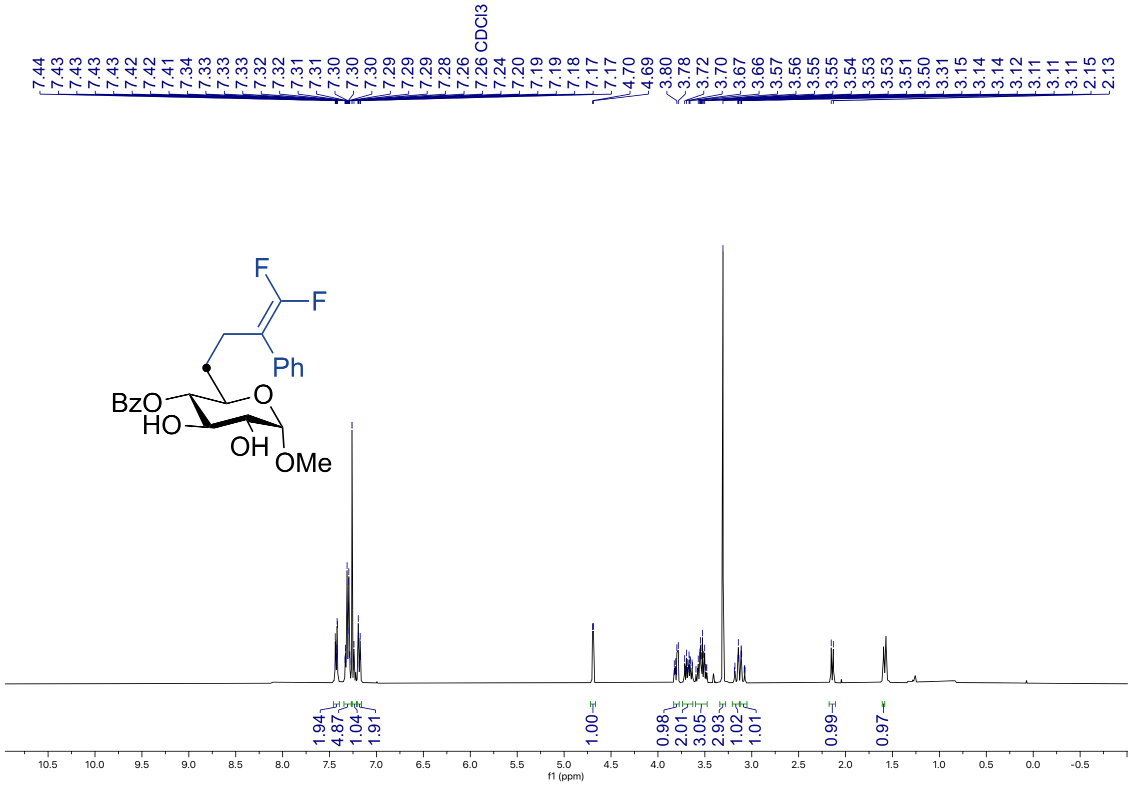

^{1}H NMR (400 MHz, CDCl$_3$) of 6z

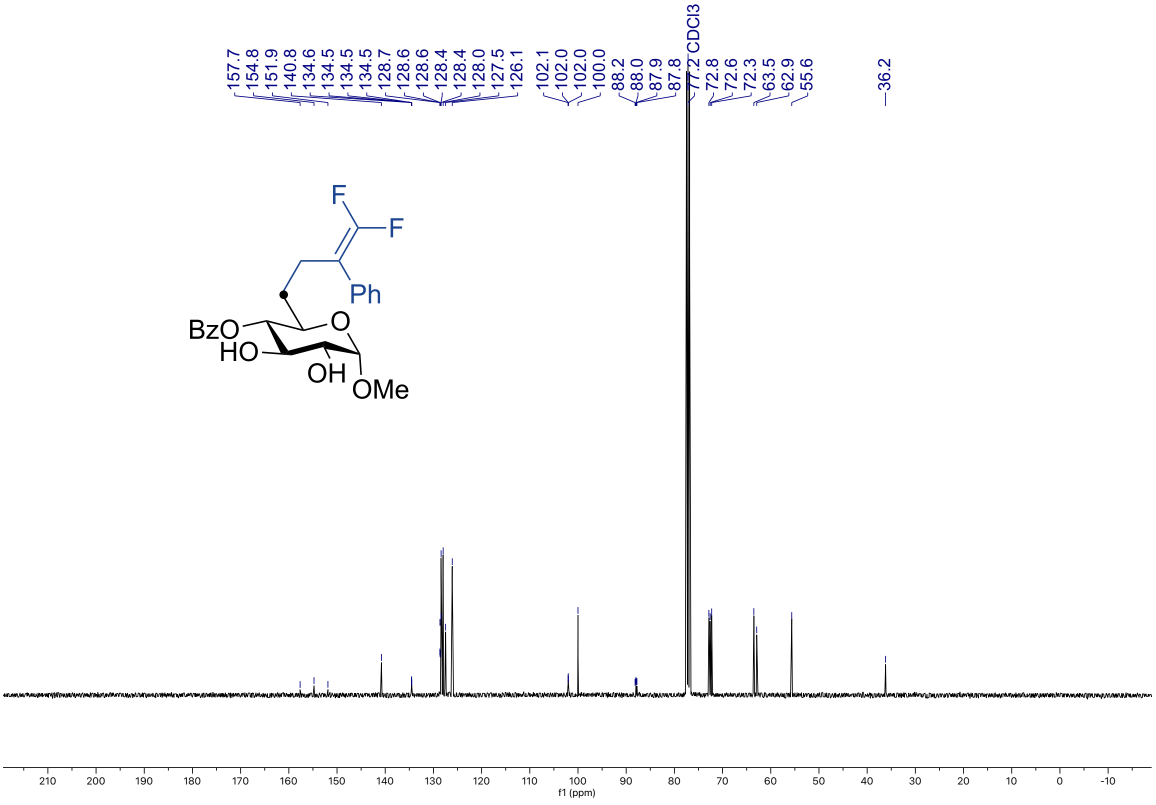

^{13}C NMR (101 MHz, CDCl$_3$) of 6z

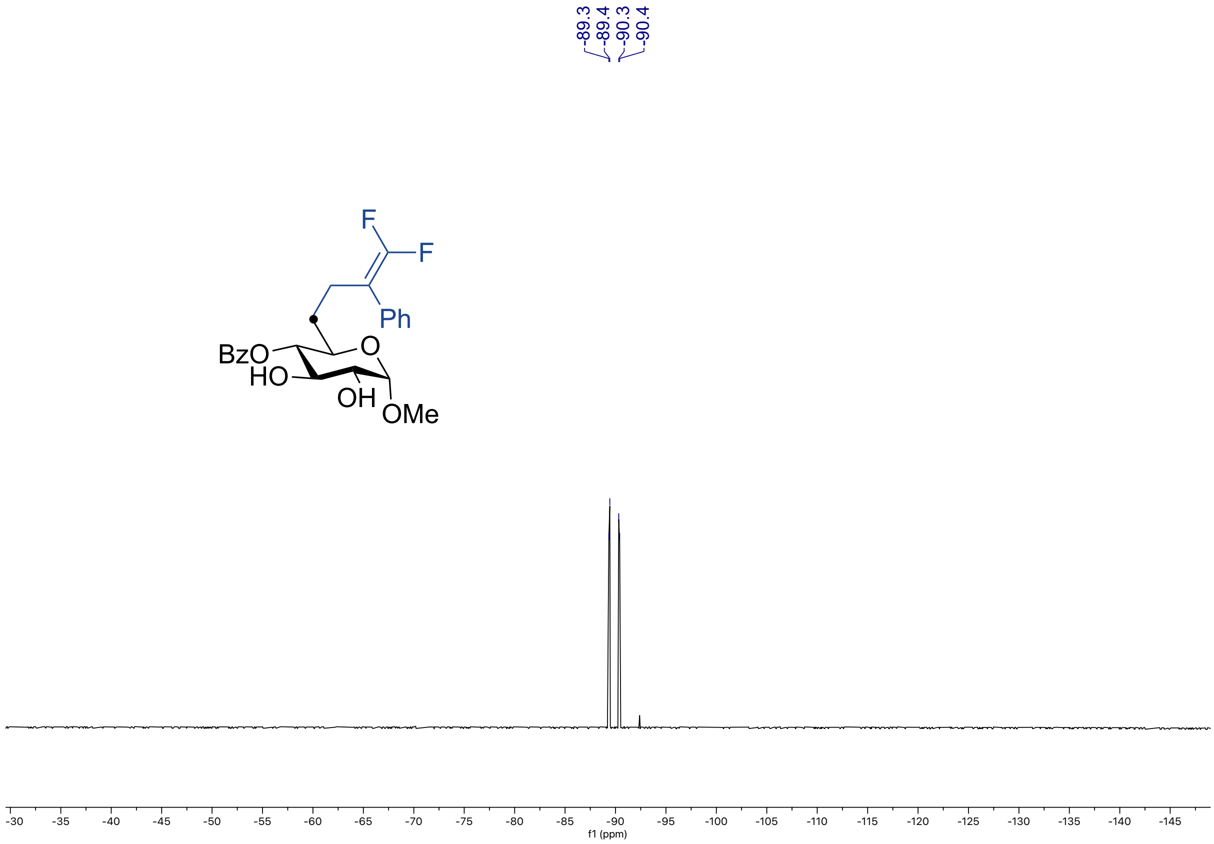

^{19}F NMR (376 MHz, CDCl$_3$) of 6z

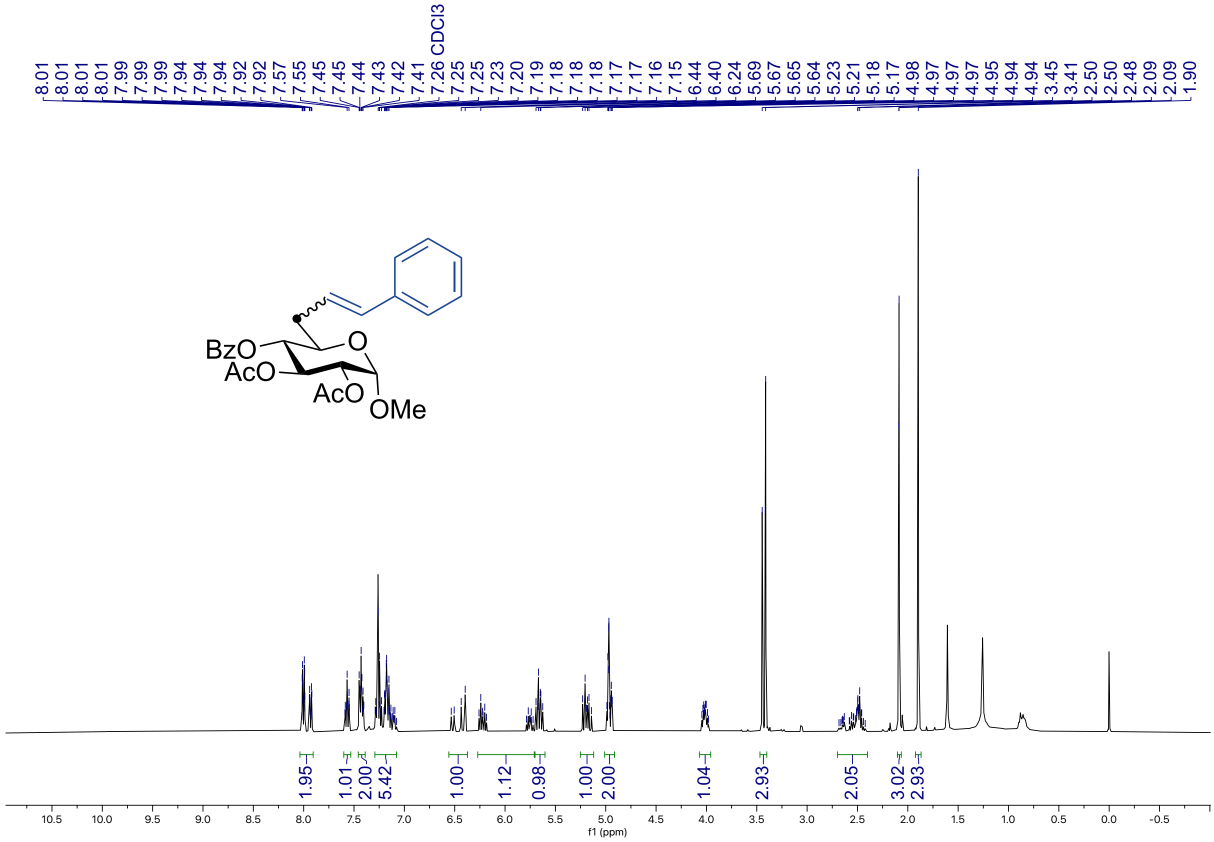

^{1}H NMR (400 MHz, CDCl$_3$) of 6za

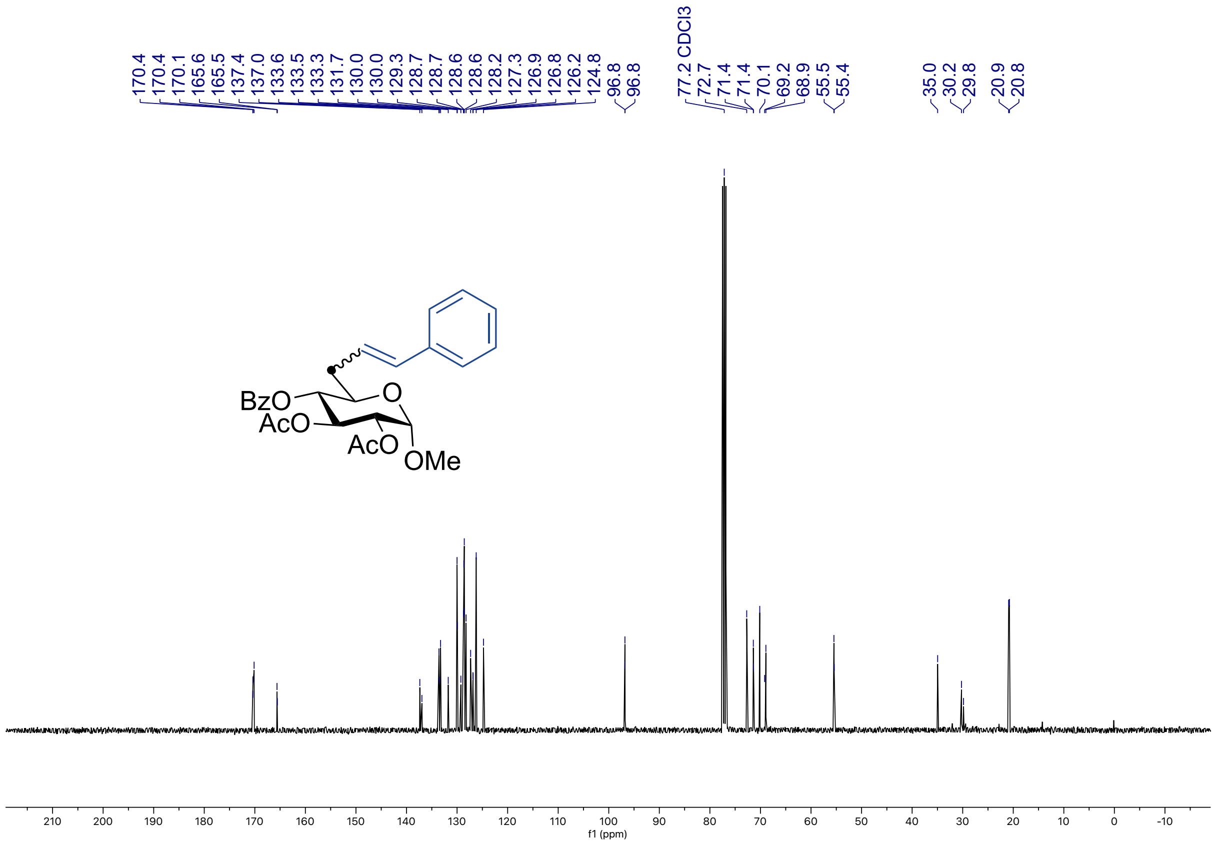

^{13}C NMR (101 MHz, CDCl$_3$) of 6za

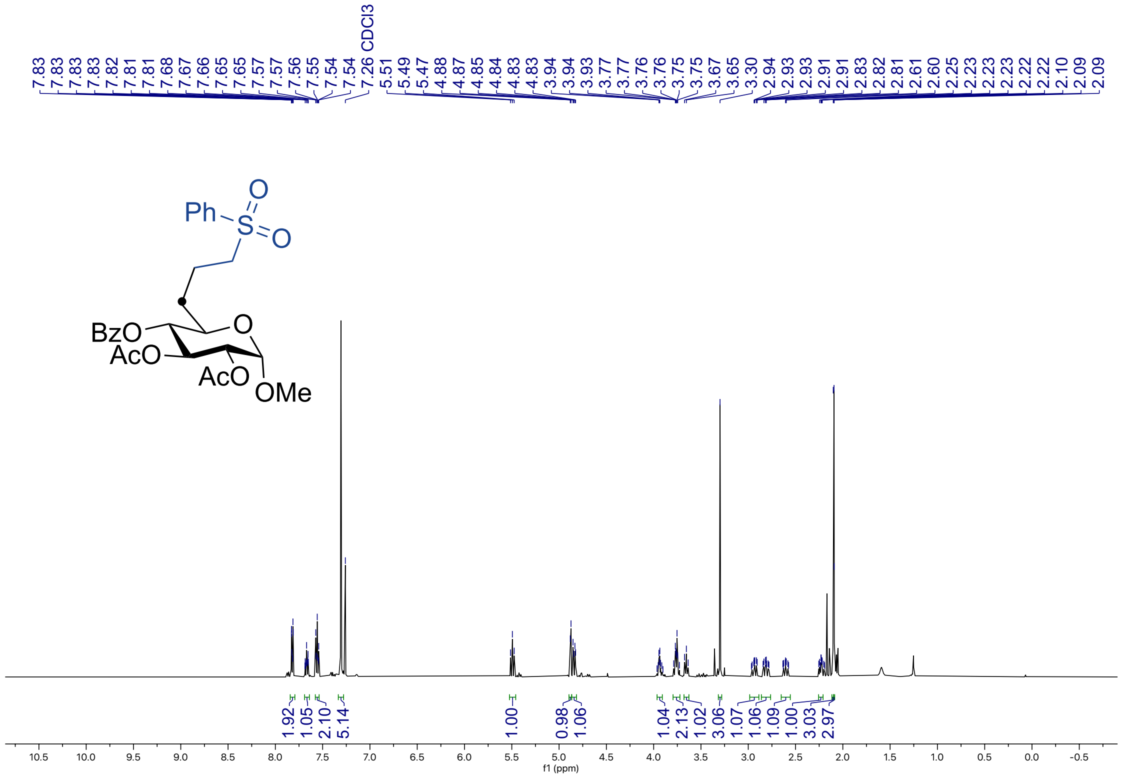

^{1}H NMR (500 MHz, CDCl$_3$) of 6zb

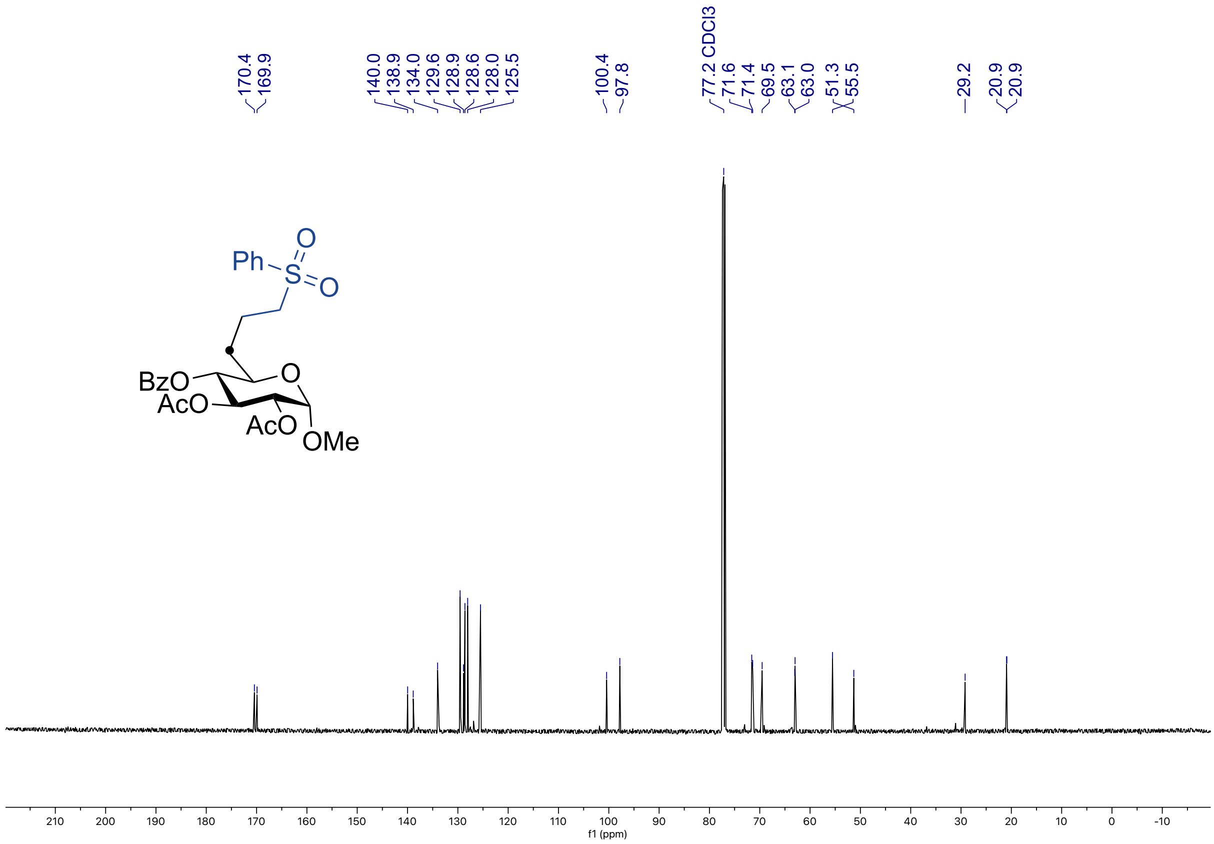

^{13}C NMR (126 MHz, CDCl₃) of 6zb

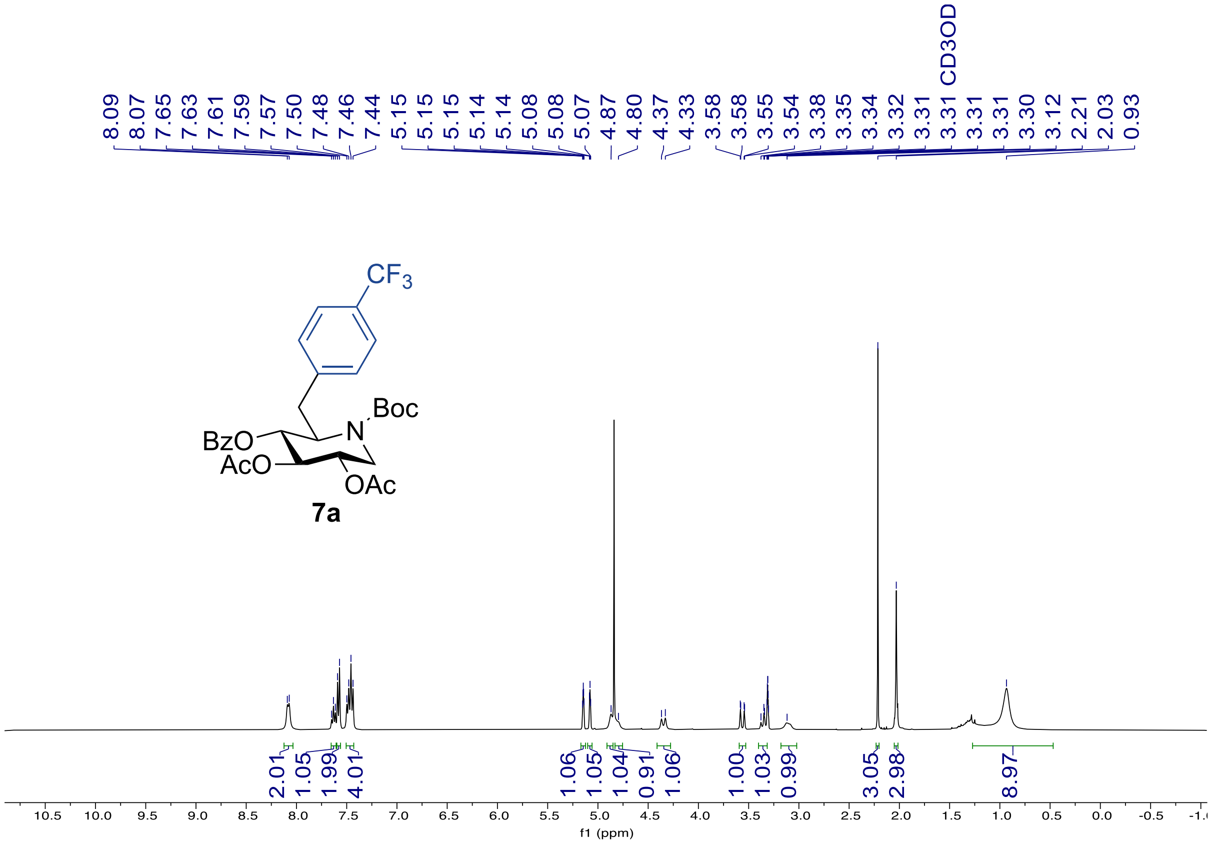

^{1}H NMR (400 MHz, MeOD) of 7a

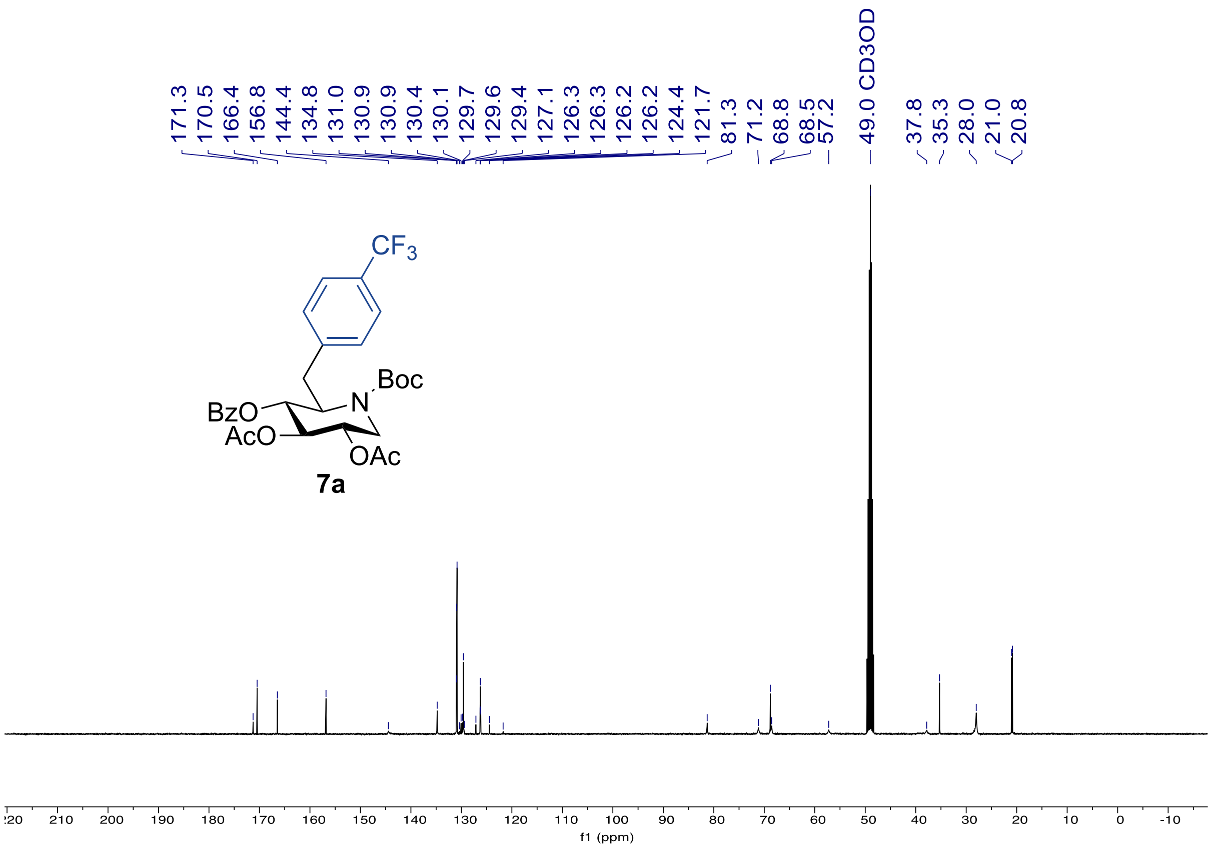

^{13}C NMR (101 MHz, MeOD) of 7a

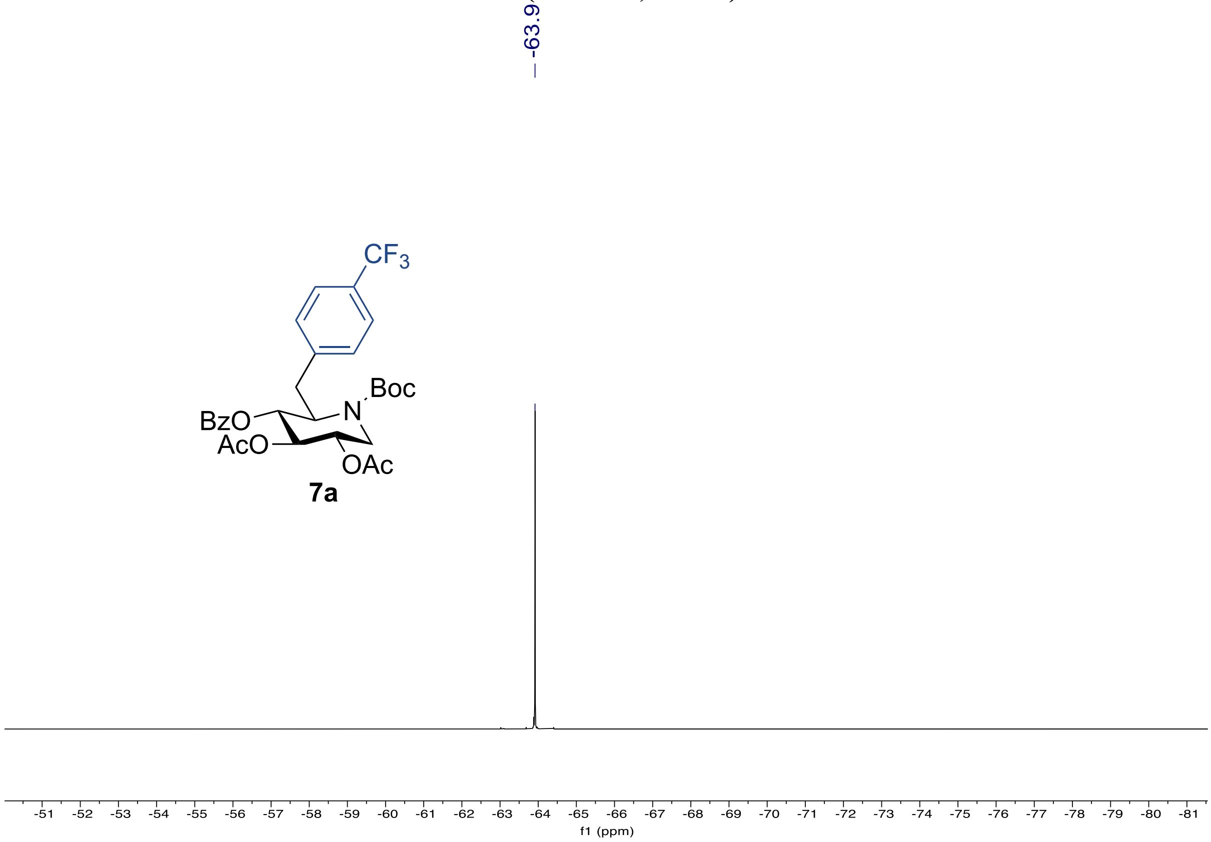

^{19}F NMR (376 MHz, MeOD) of 7a

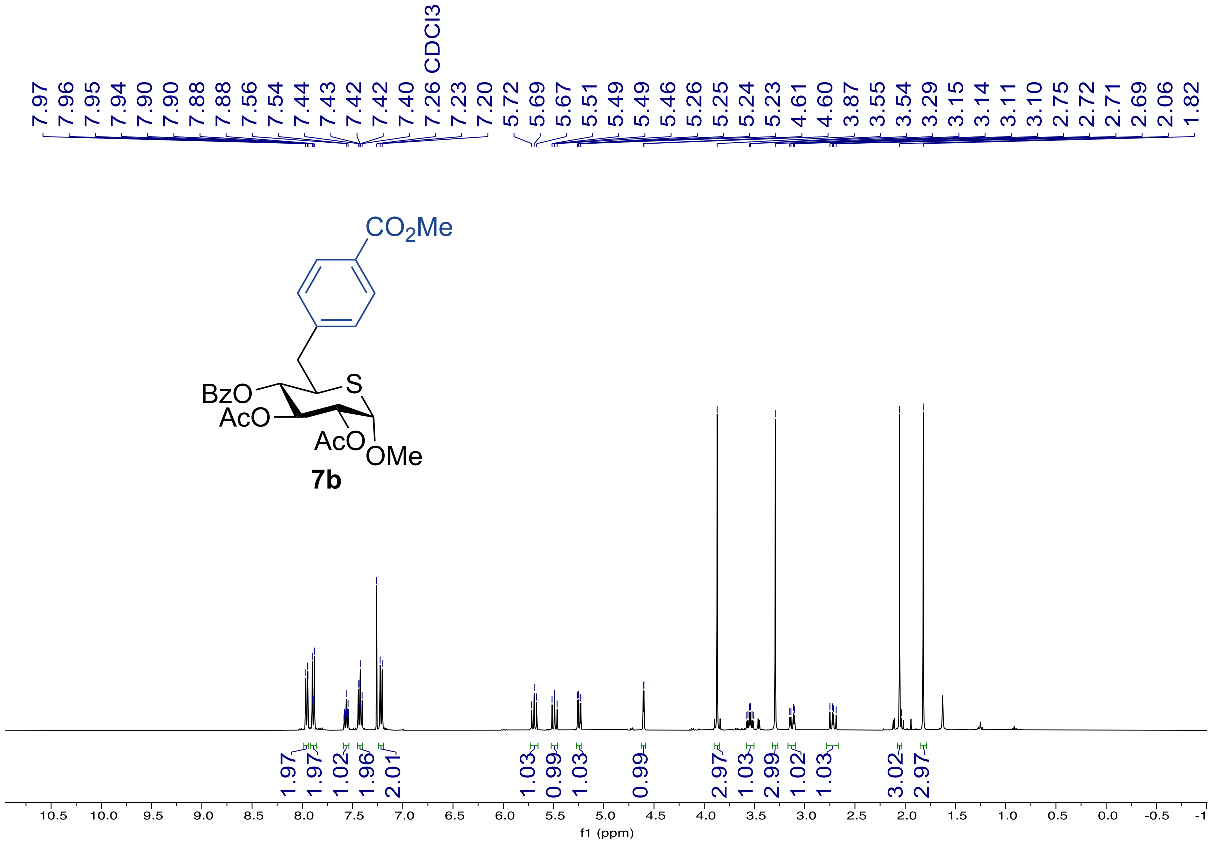

^{1}H NMR (400 MHz, CDCl$_3$) of 7b

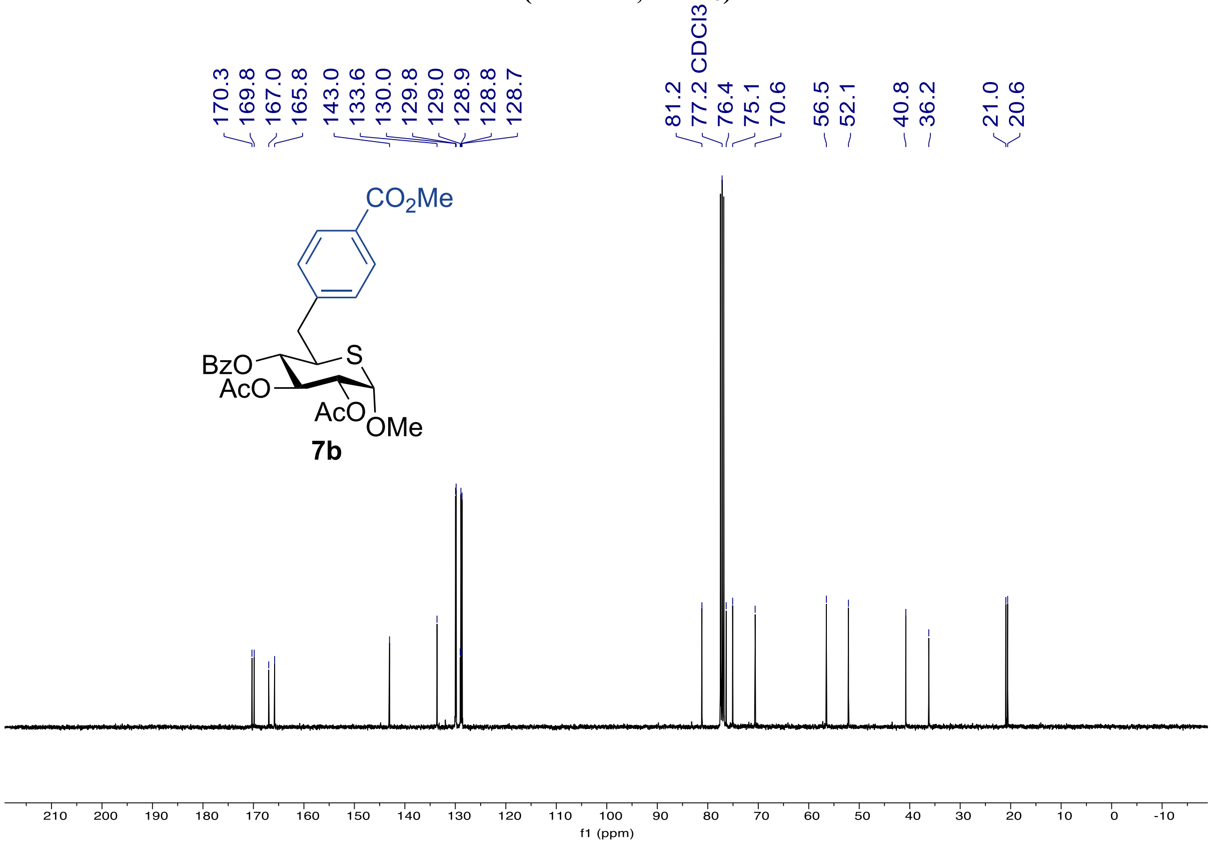

^{13}C NMR (101 MHz, CDCl$_3$) of 7b

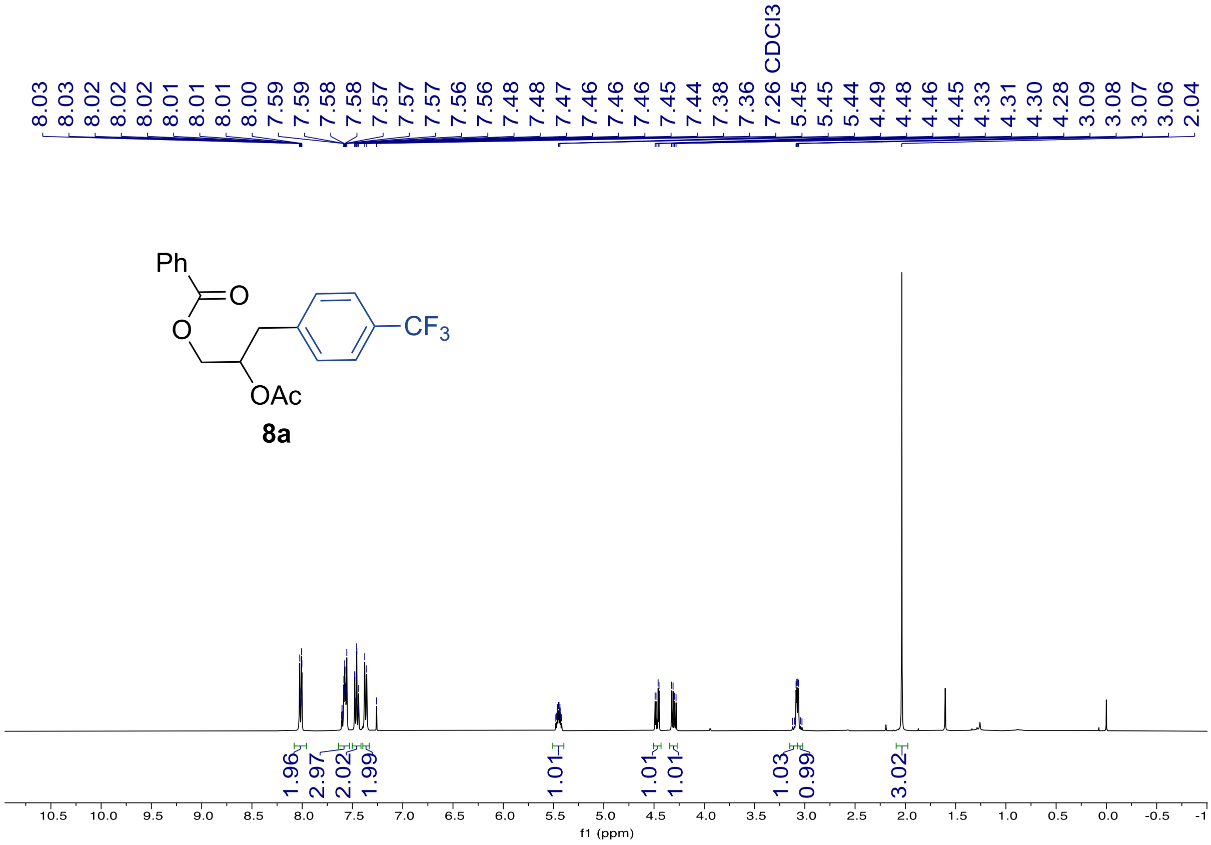

^{1}H NMR (400 MHz, CDCl$_3$) of 8a

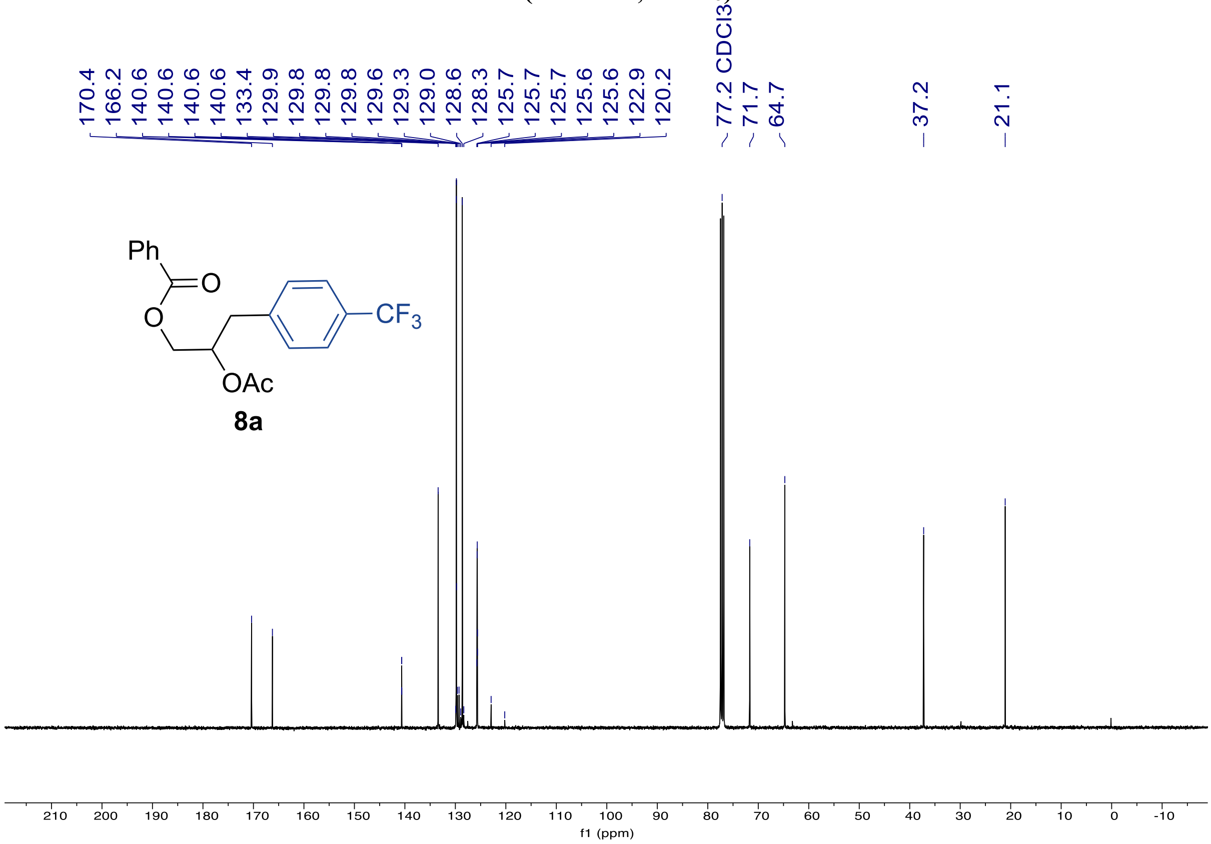

^{13}C NMR (101 MHz, CDCl$_3$) of 8a

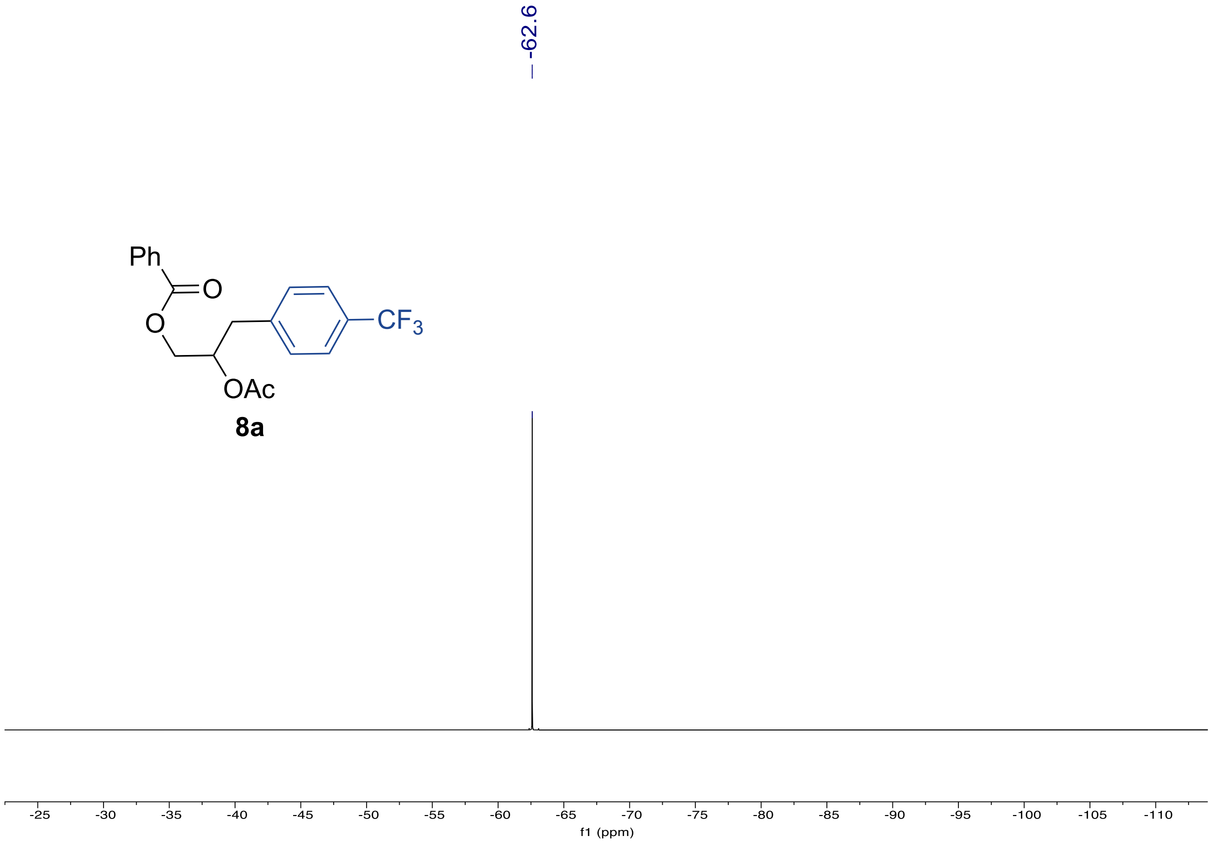

^{19}F NMR (376 MHz, CDCl$_3$) of 8a

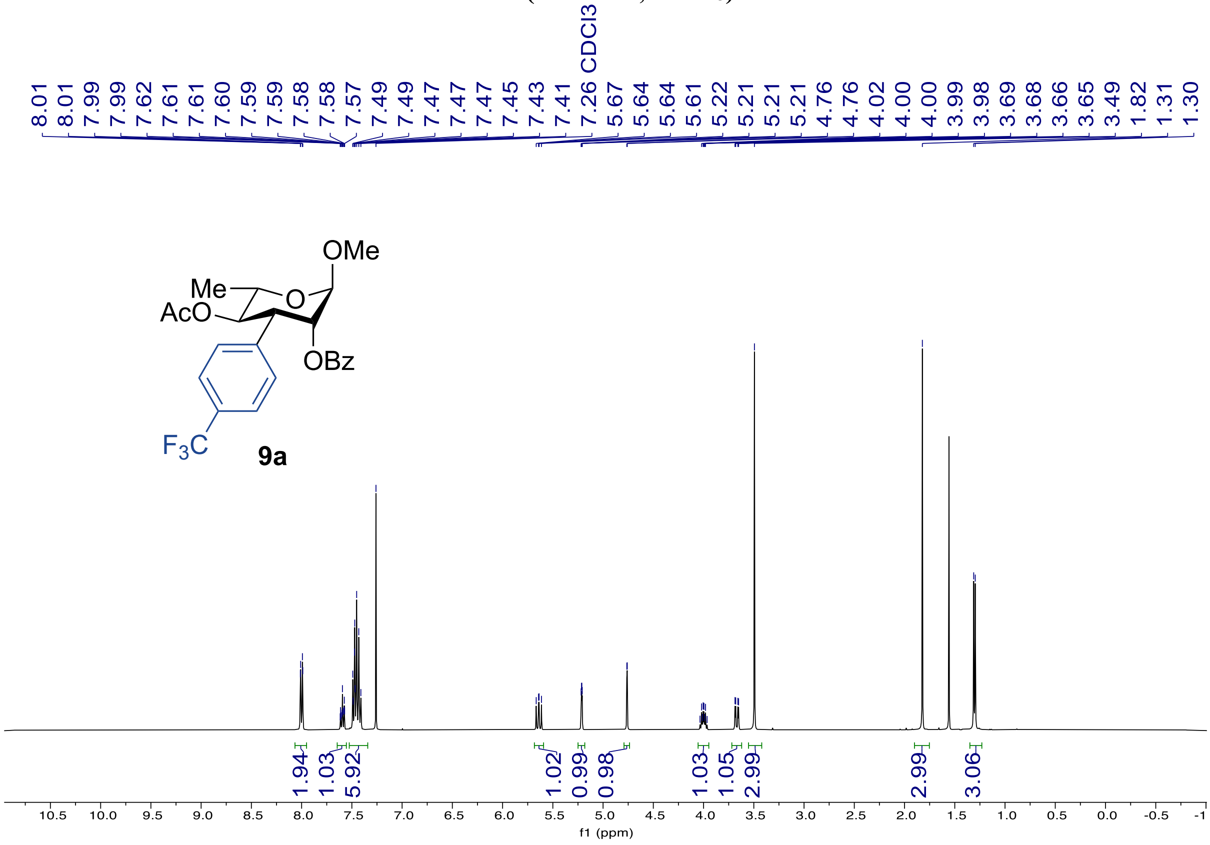

^{1}H NMR (400 MHz, CDCl$_3$) of 9a

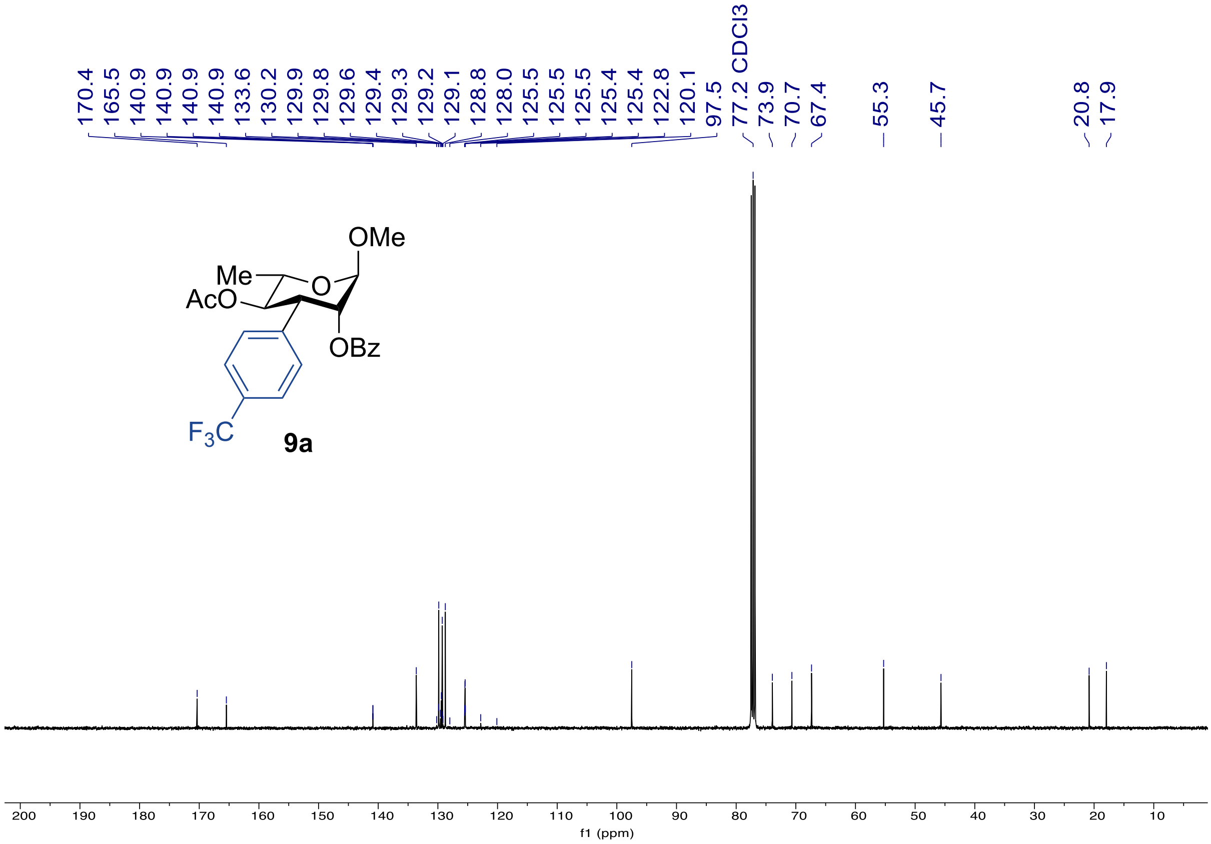

^{13}C NMR (101 MHz, CDCl$_3$) of 9a

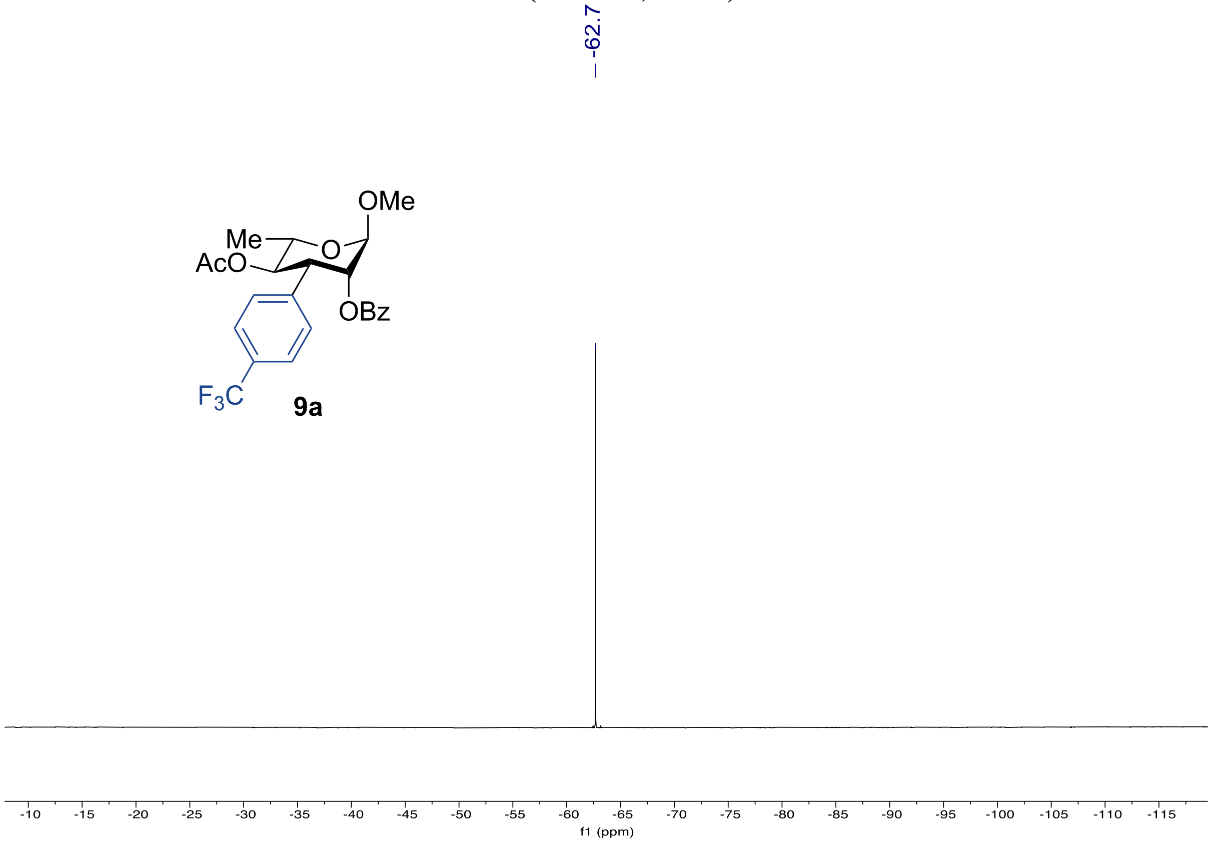

^{19}F NMR (376 MHz, CDCl$_3$) of 9a

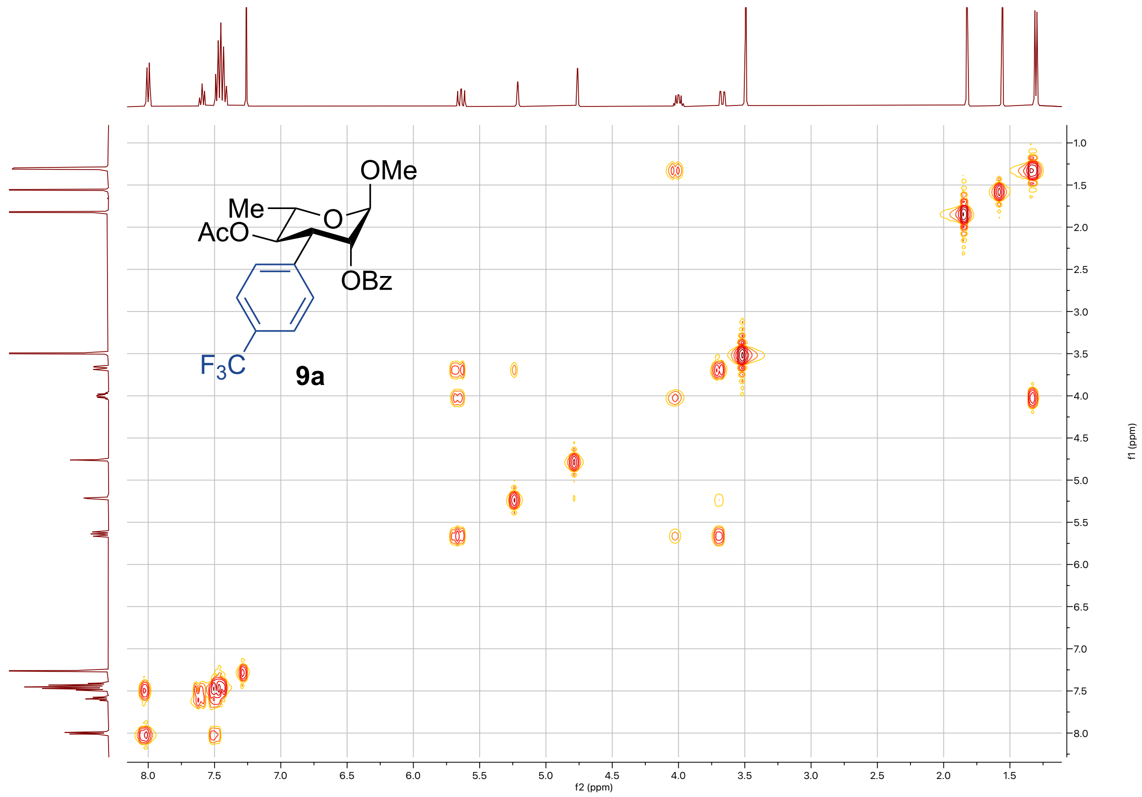

^{1}H-^{1}H COSY of 9a

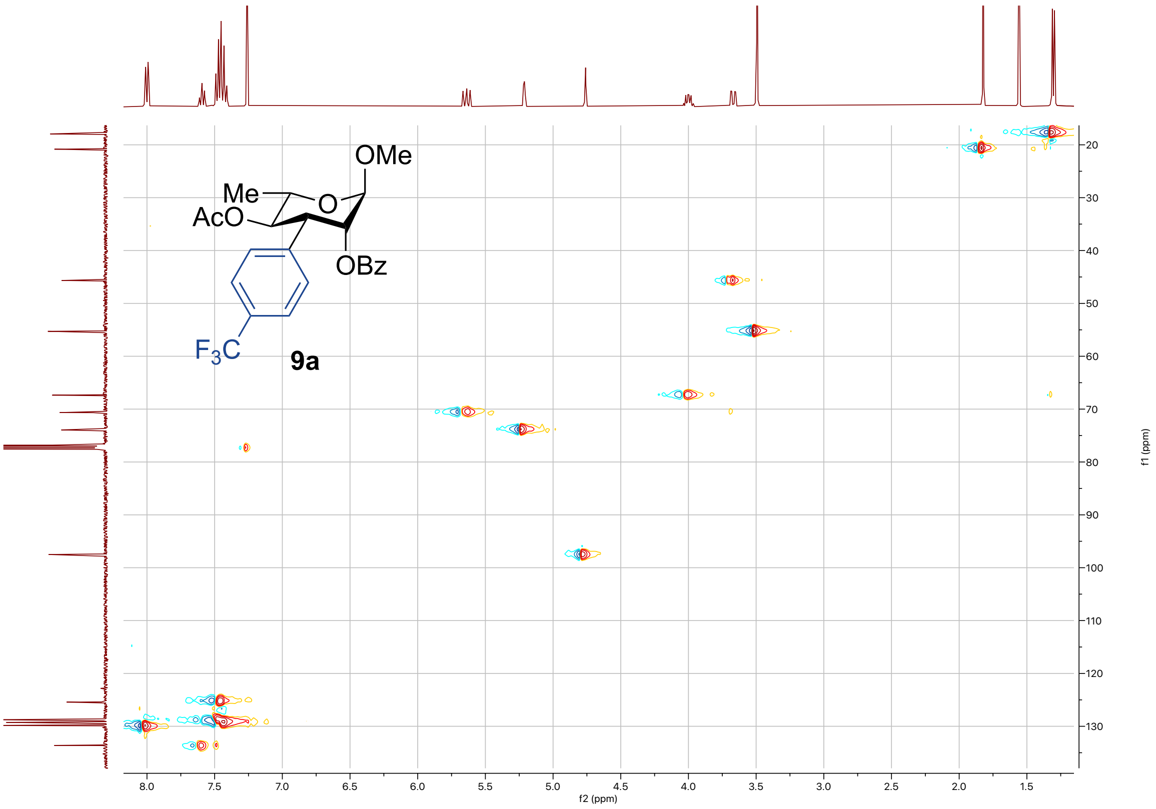

^{1}H-^{13}C HSQC of 9a

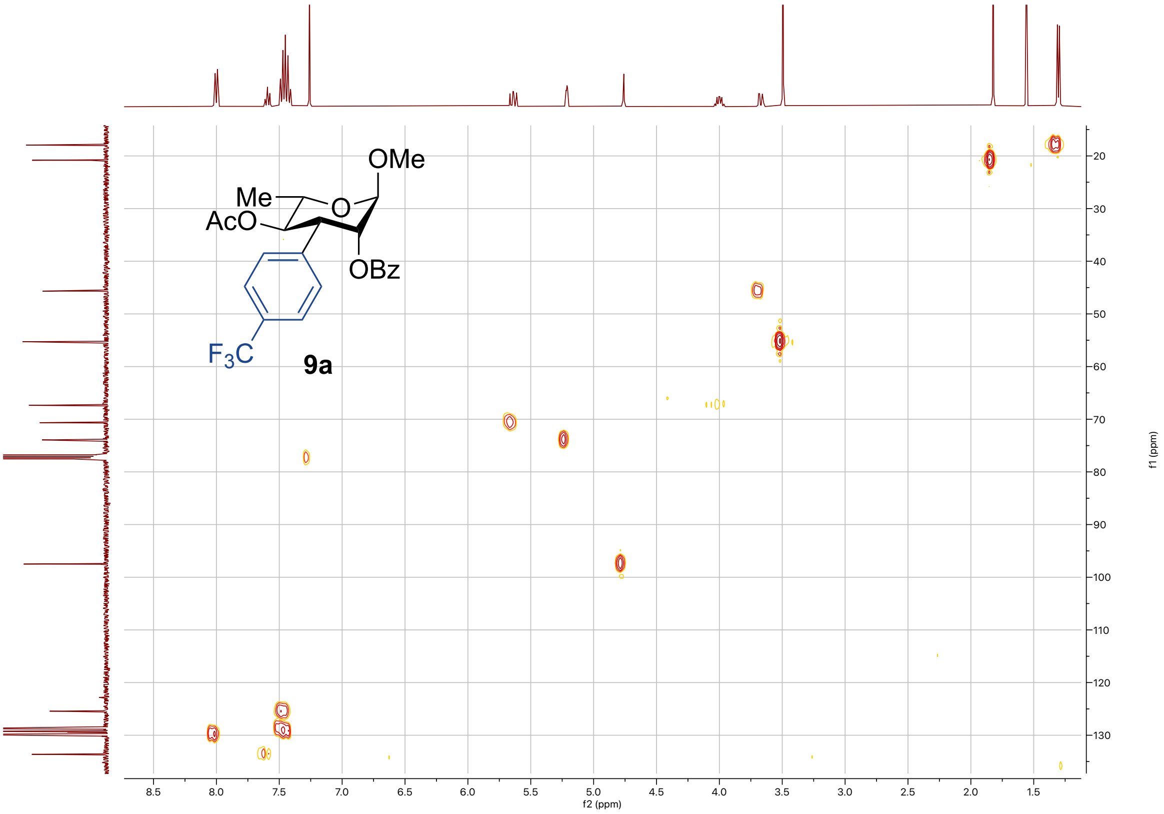

[1]H-^{13}C HMQC of 9a

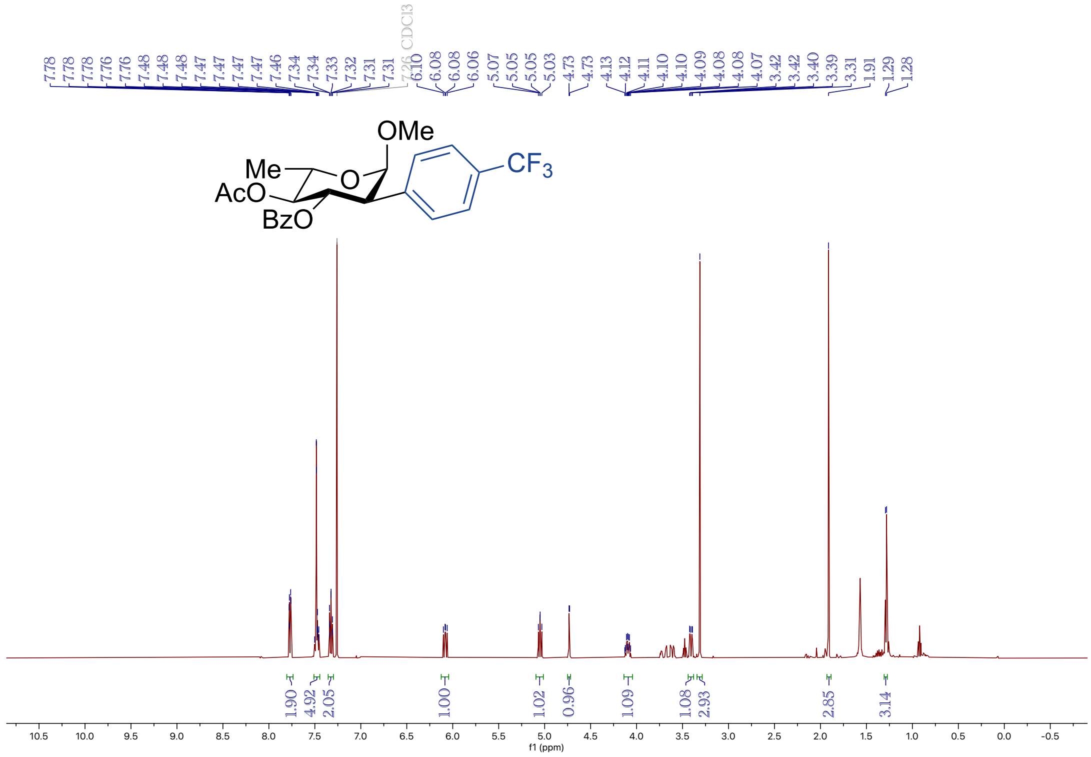

^{1}H NMR (500 MHz, CDCl₃) of 9a′

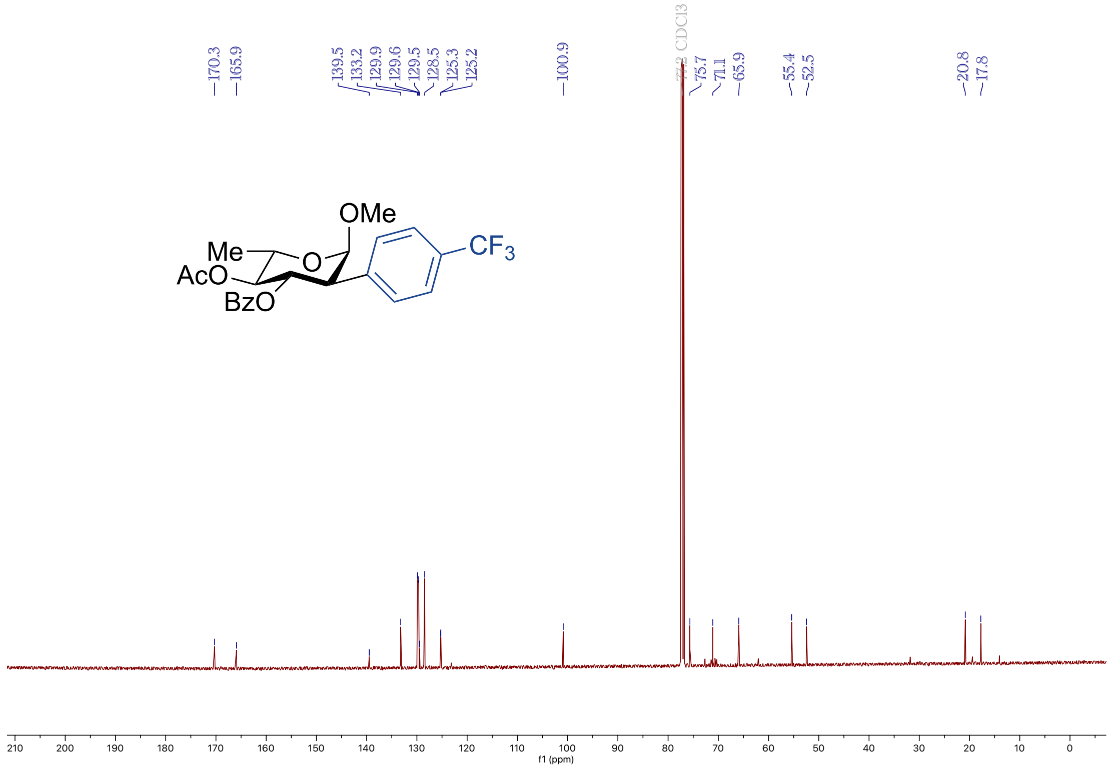

^{13}C NMR (126 MHz, $CDCl_3$) of 9a′

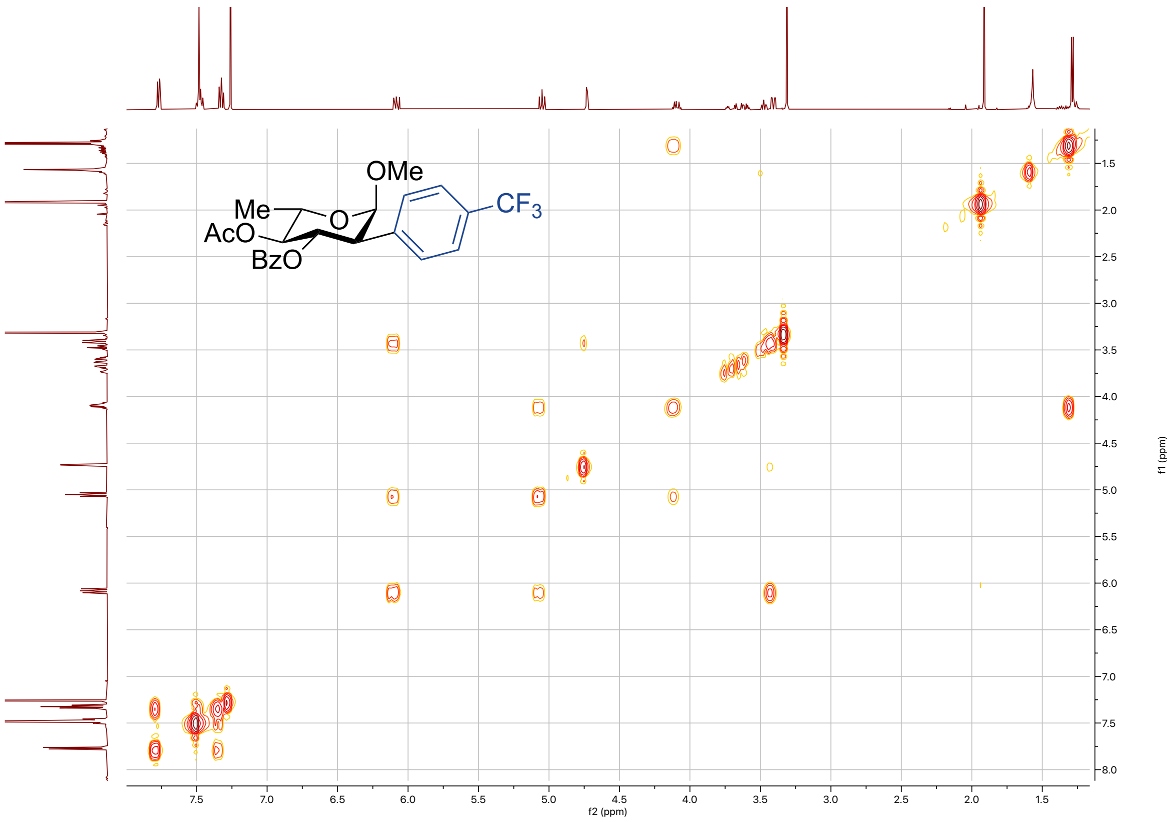

^{1}H-^{1}H COSY of 9a′

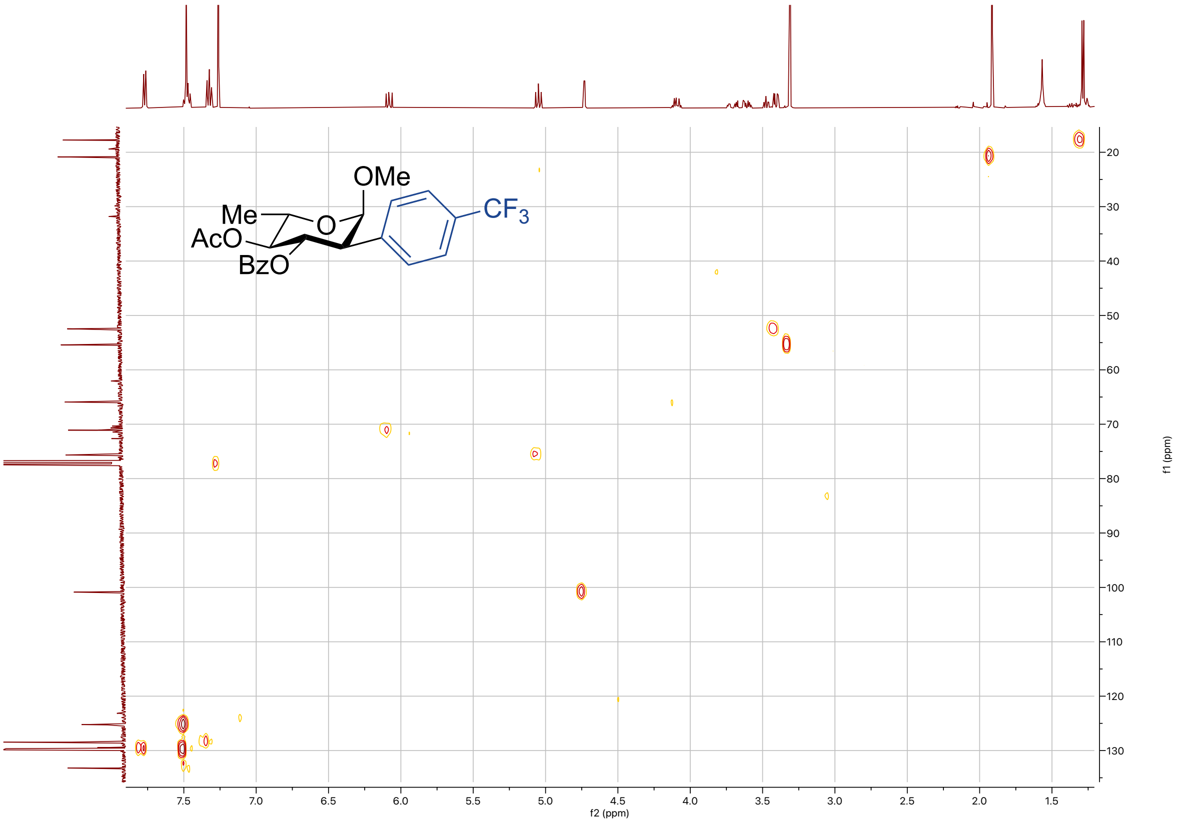

^{1}H-^{13}C HMQCof 9a´

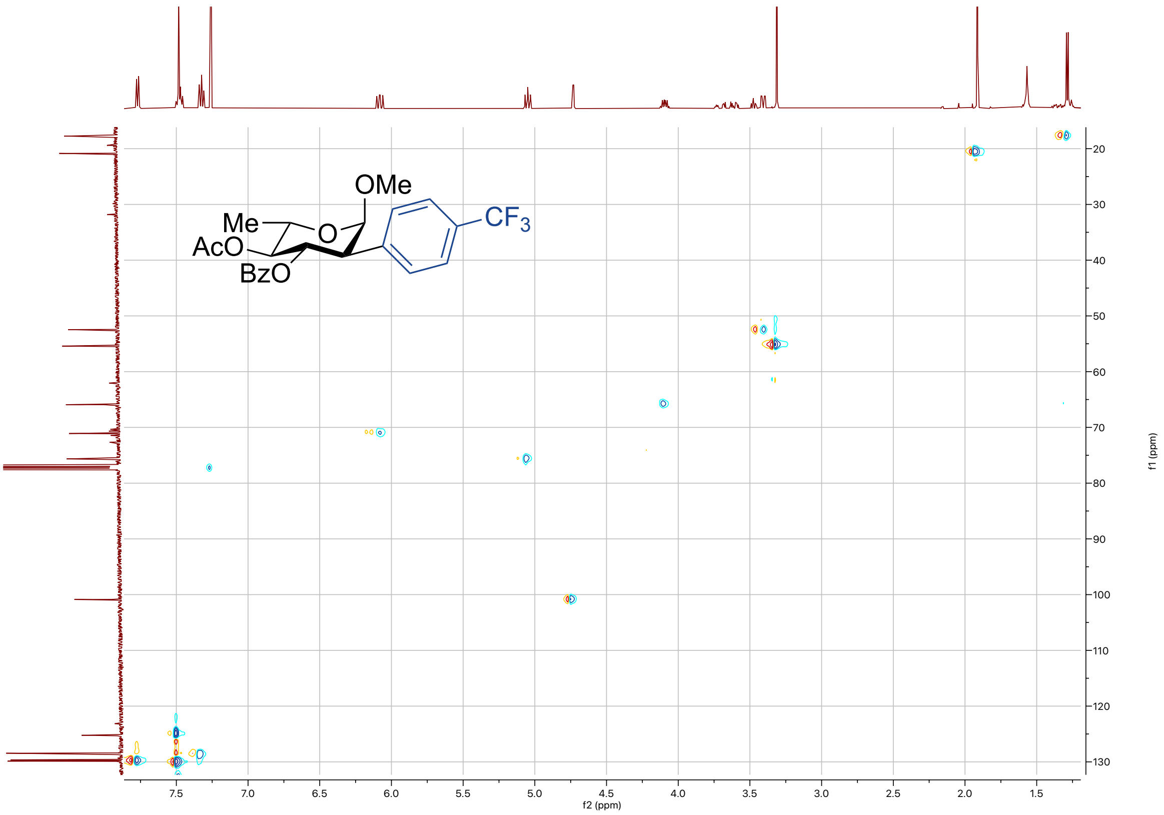

^{1}H-^{13}C HSQC of 9a´

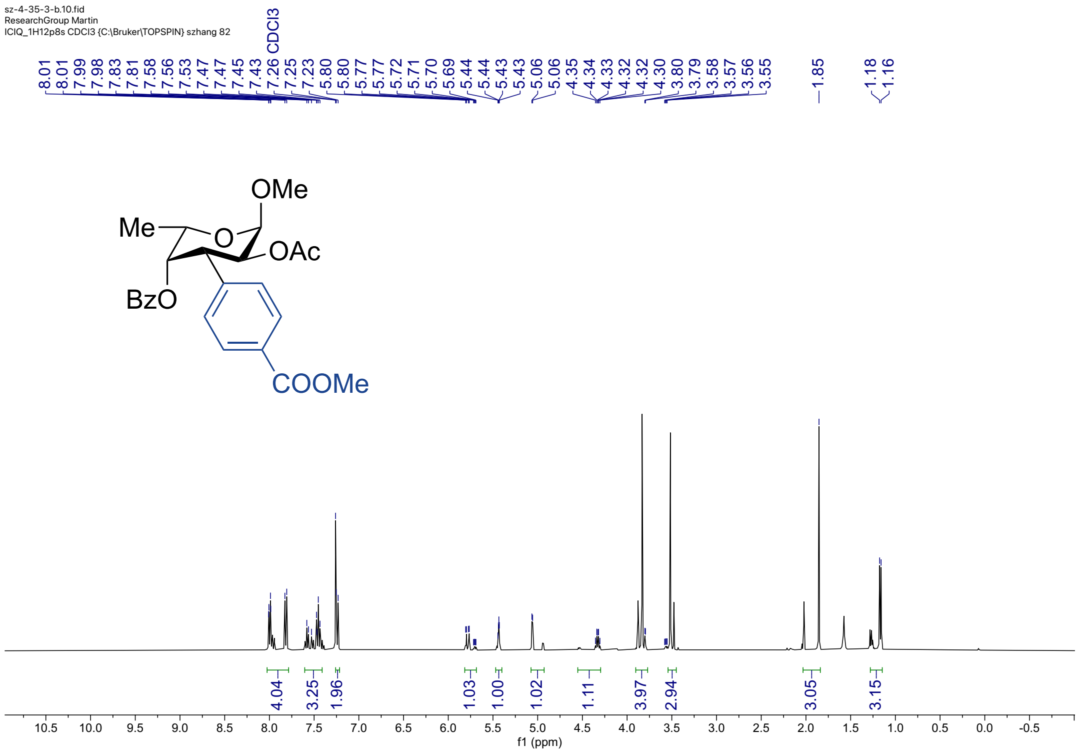

^{1}H NMR (400 MHz, CDCl₃) of 9b

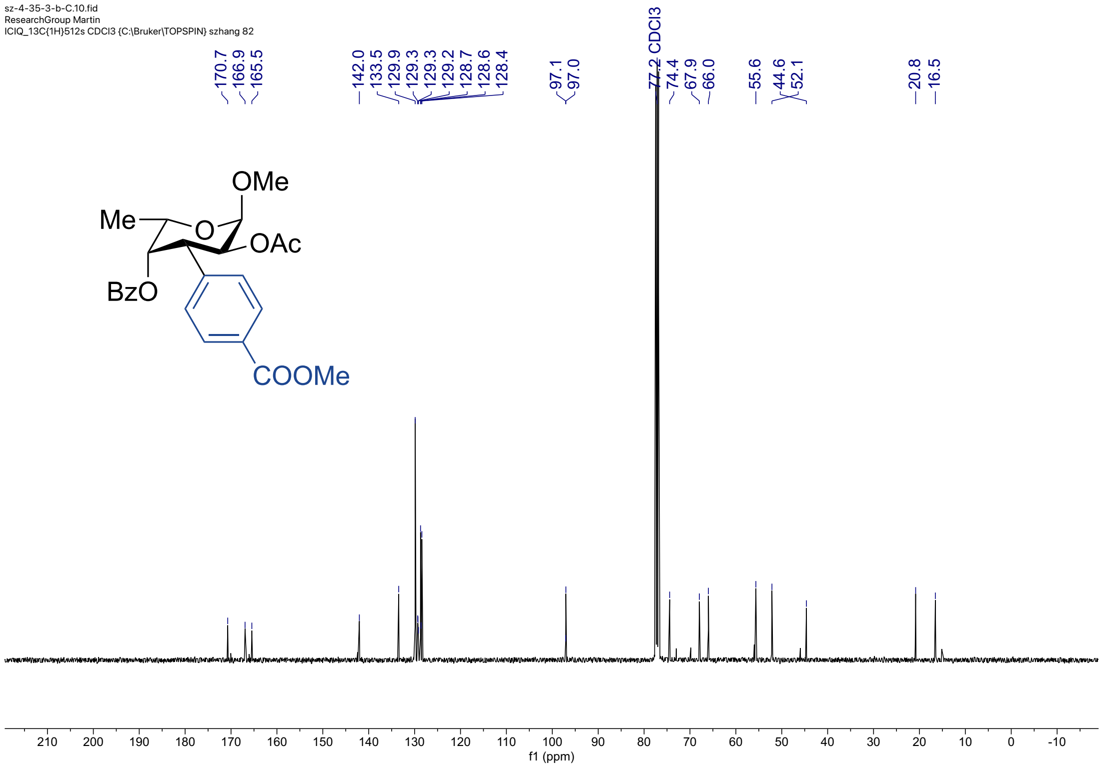

^{13}C NMR (101 MHz, CDCl₃) of 9b

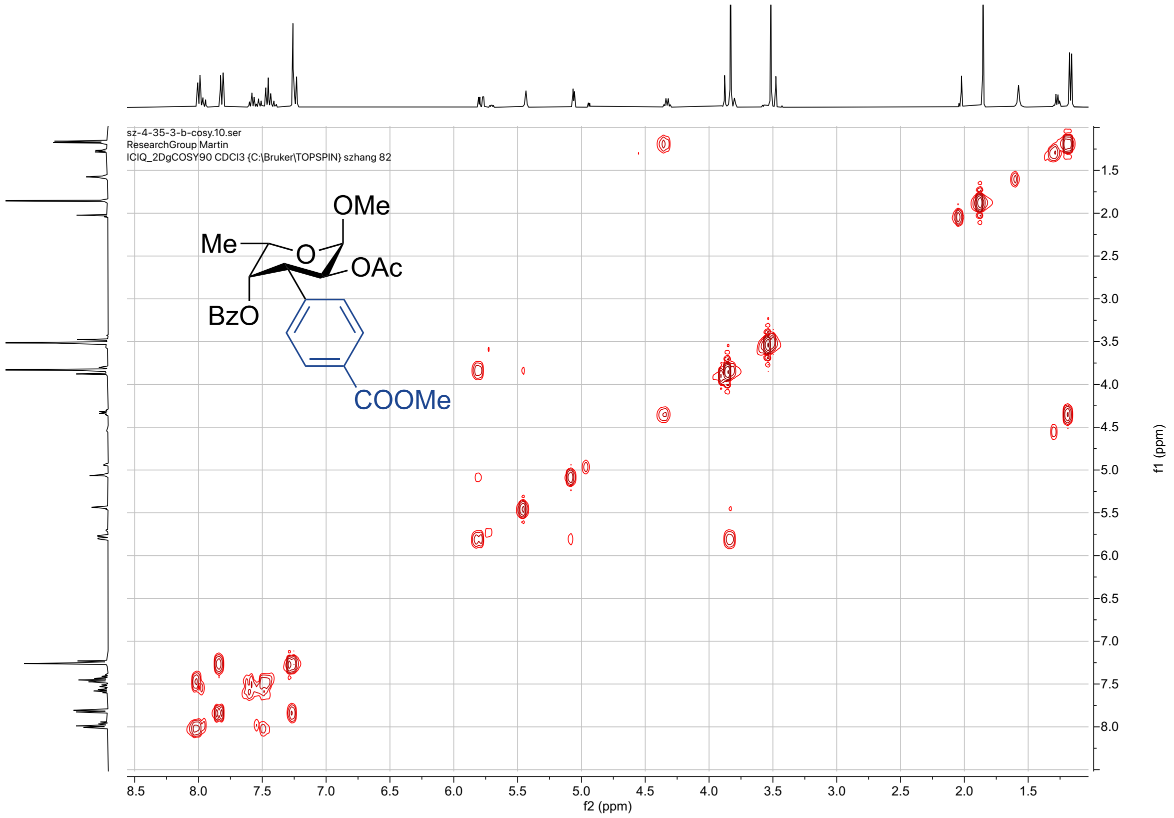

^{1}H-^{1}H COSY of 9b

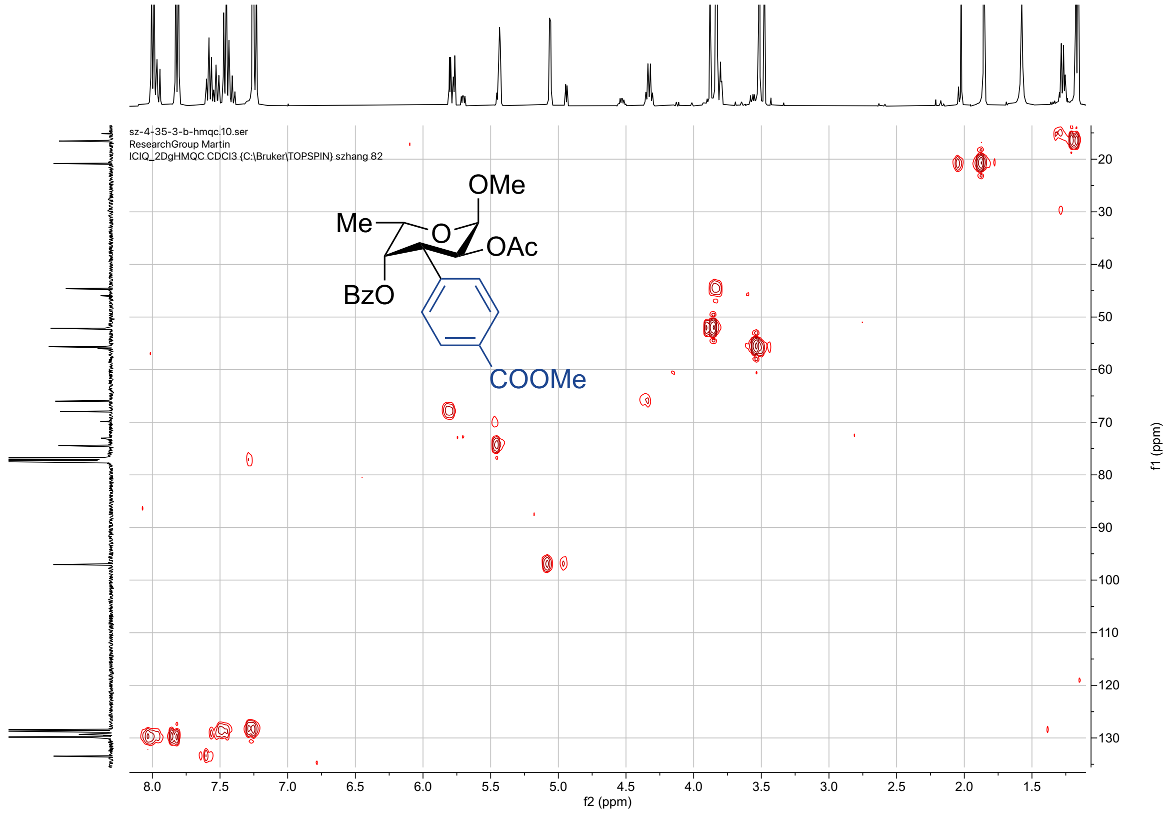

^{1}H-^{13}C HMQC of 9b

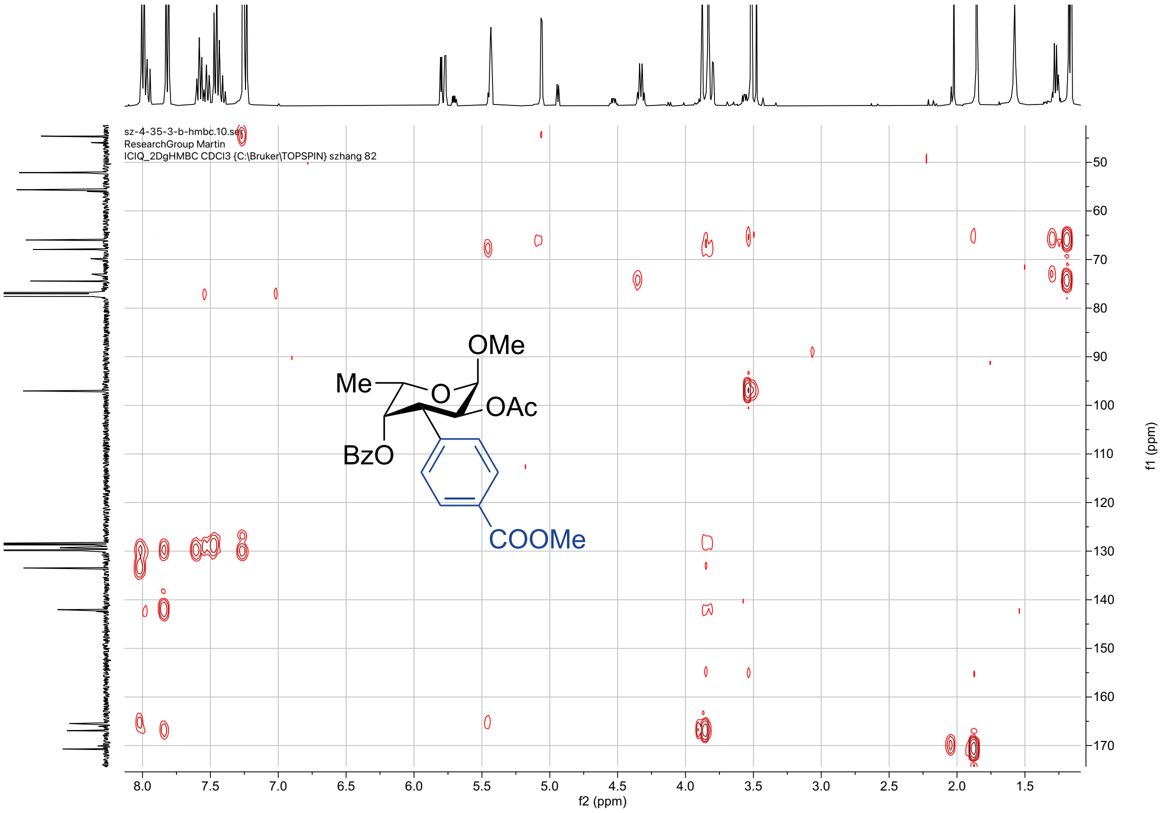

^{1}H-^{13}C HMBCof 9b

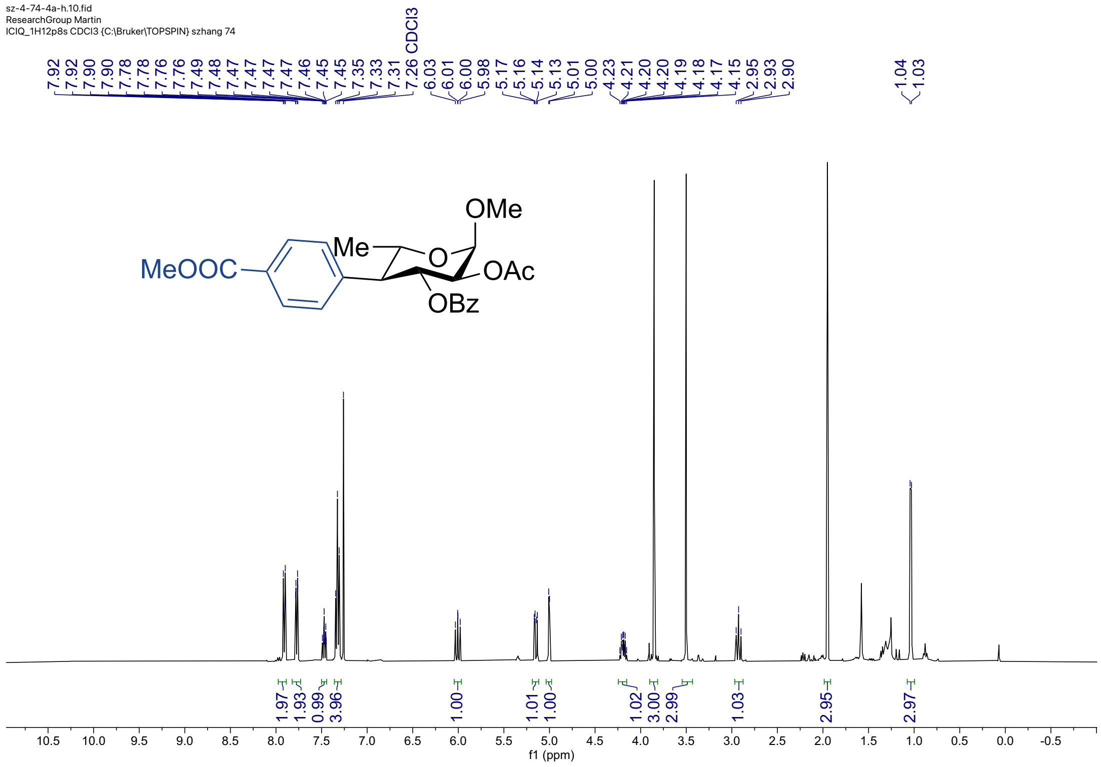

^{1}H NMR (400 MHz, CDCl$_3$) of 9b´

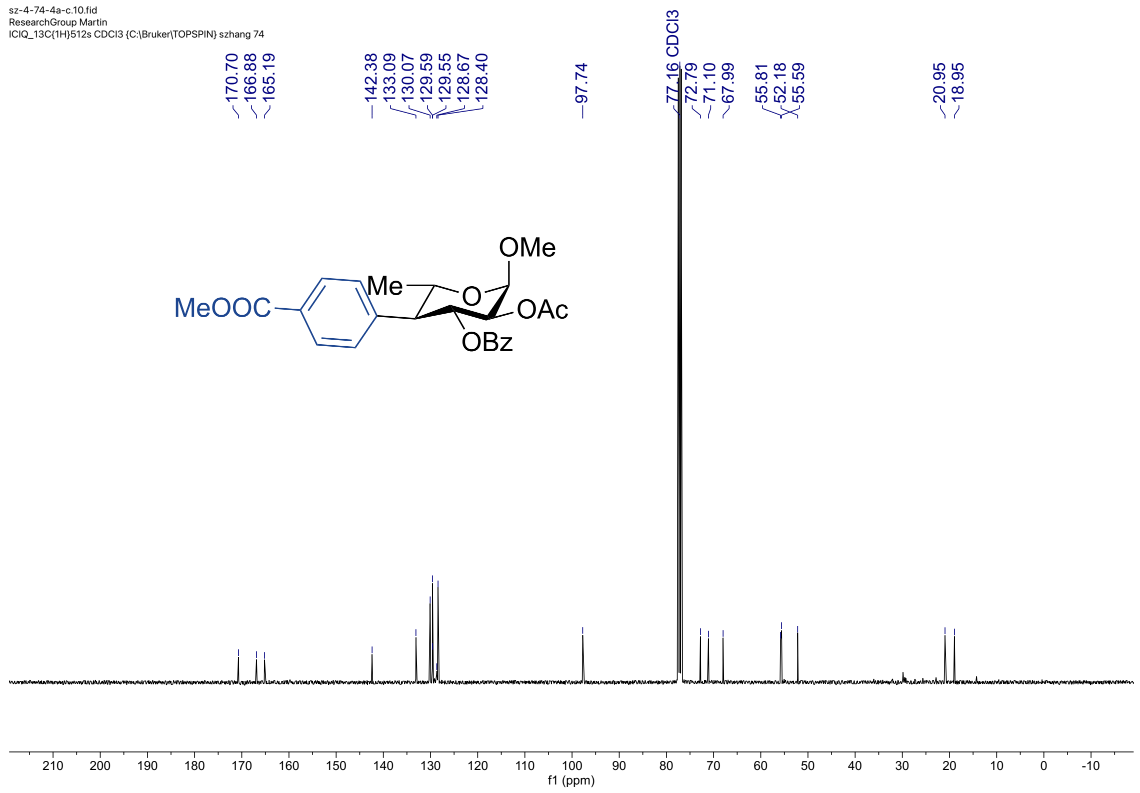

^{13}C NMR (101 MHz, CDCl$_3$) of 9b´

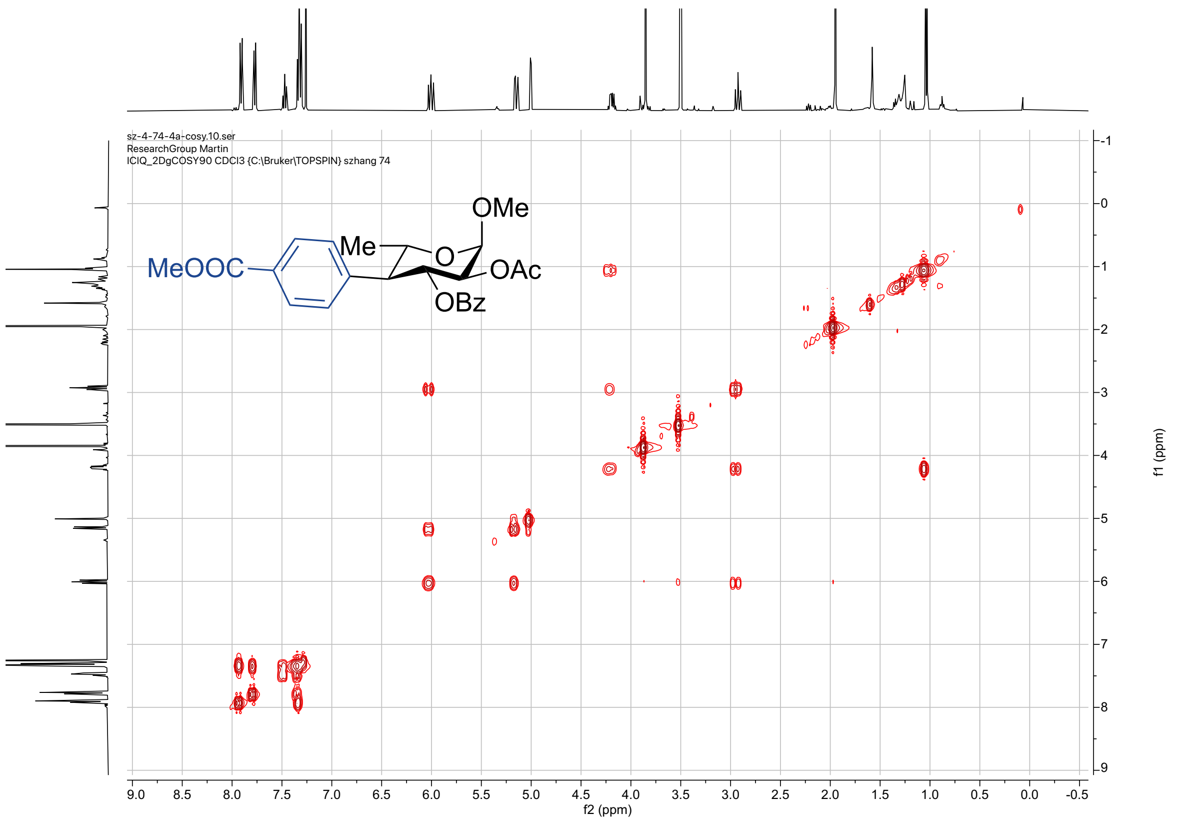

^{1}H-^{1}H COSY of 9b´

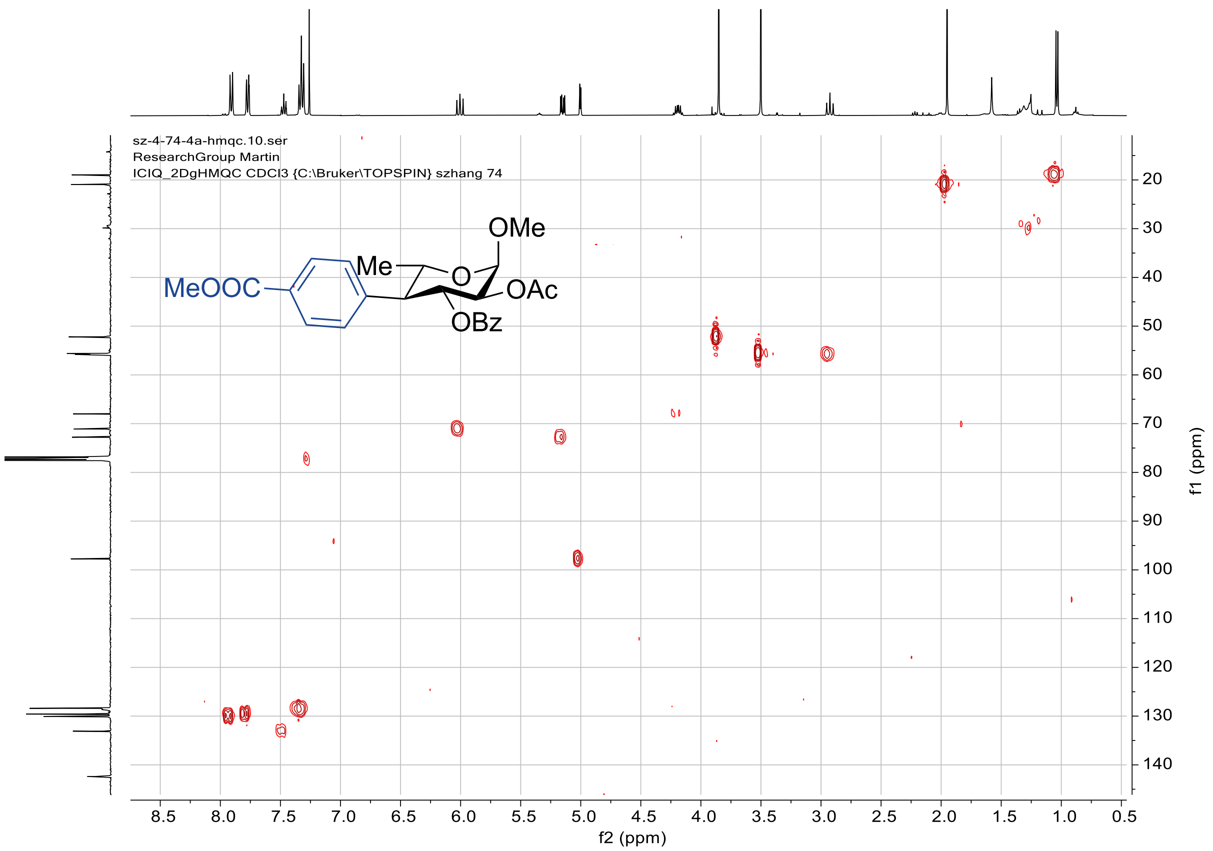

^{1}H-^{13}C HMQC of 9b´

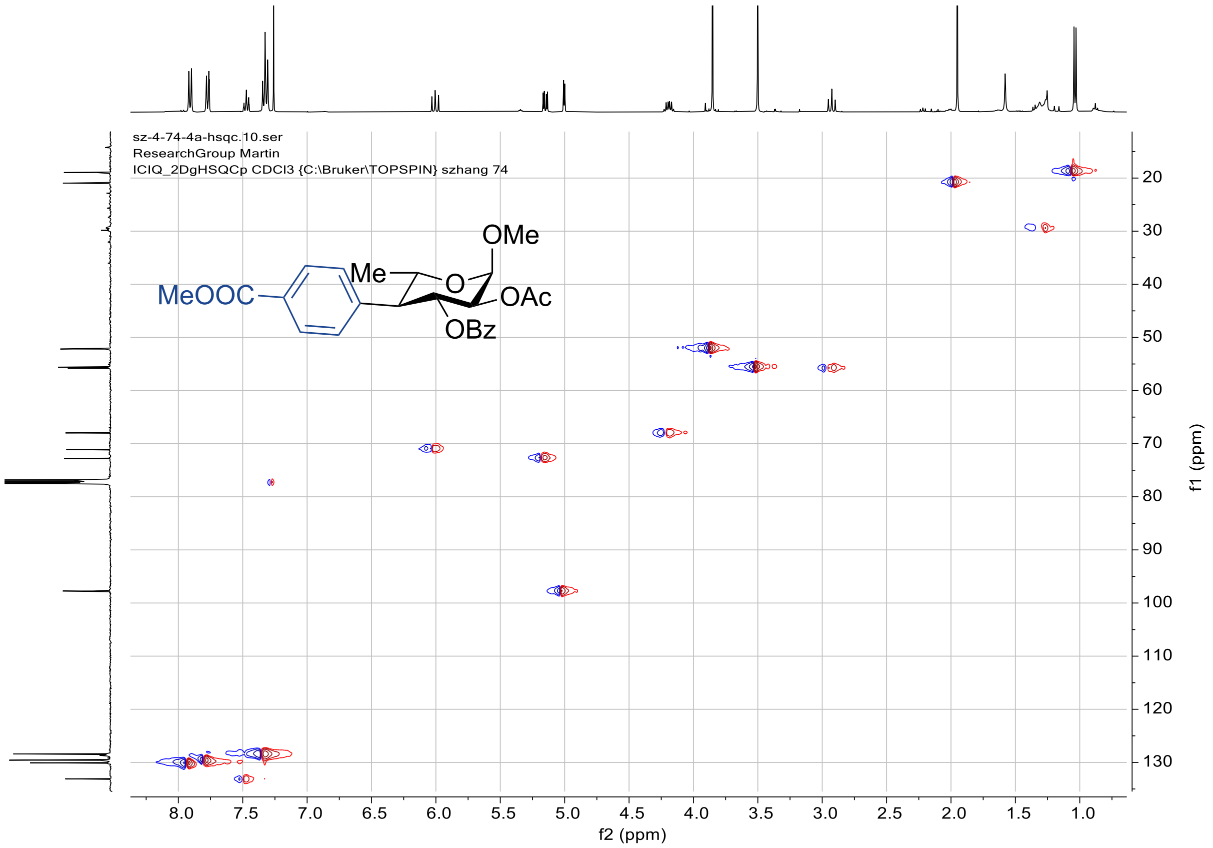

^{1}H-^{13}C HSQC of 9b´

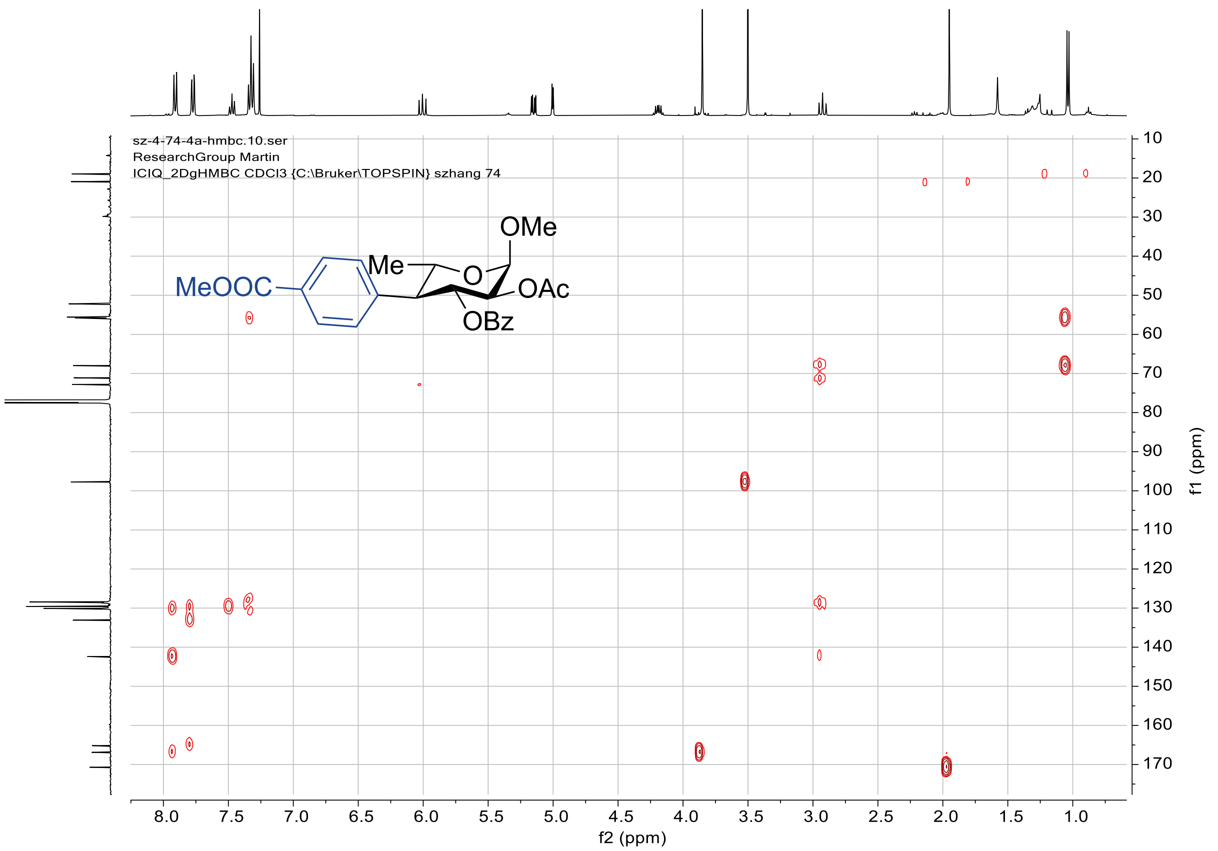

^{1}H-^{13}C HMBCof 9b´

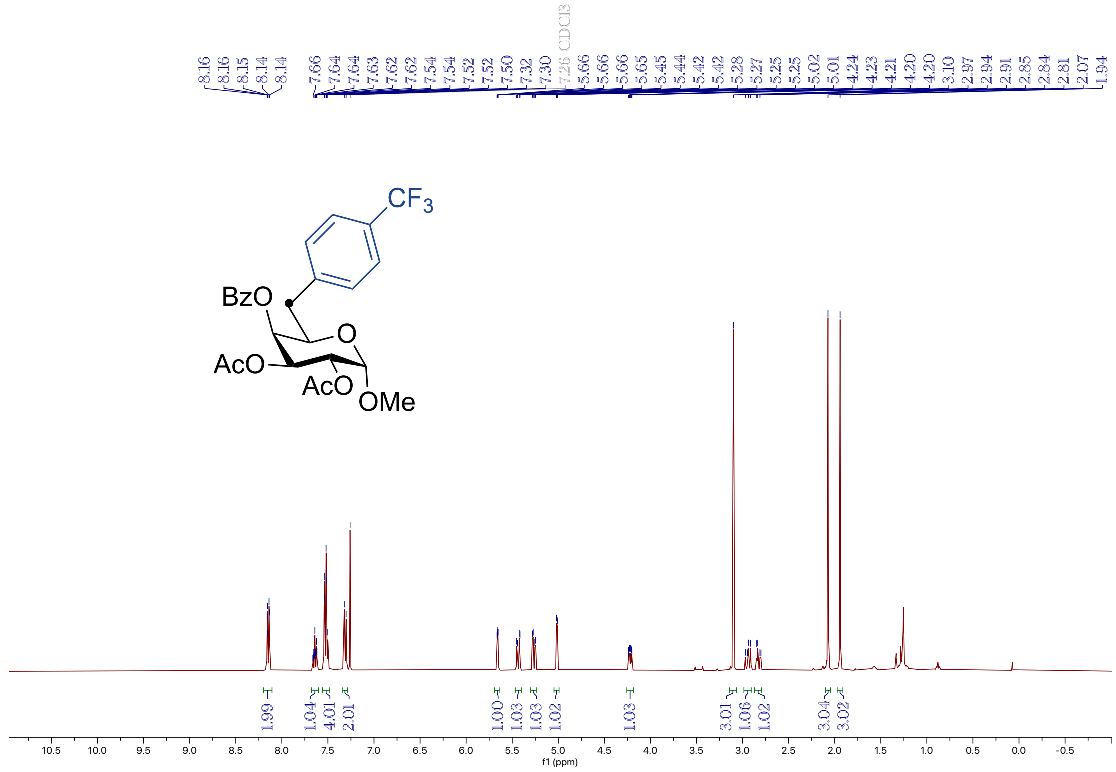

^{1}H NMR (400 MHz, $CDCl_3$) of 5ze

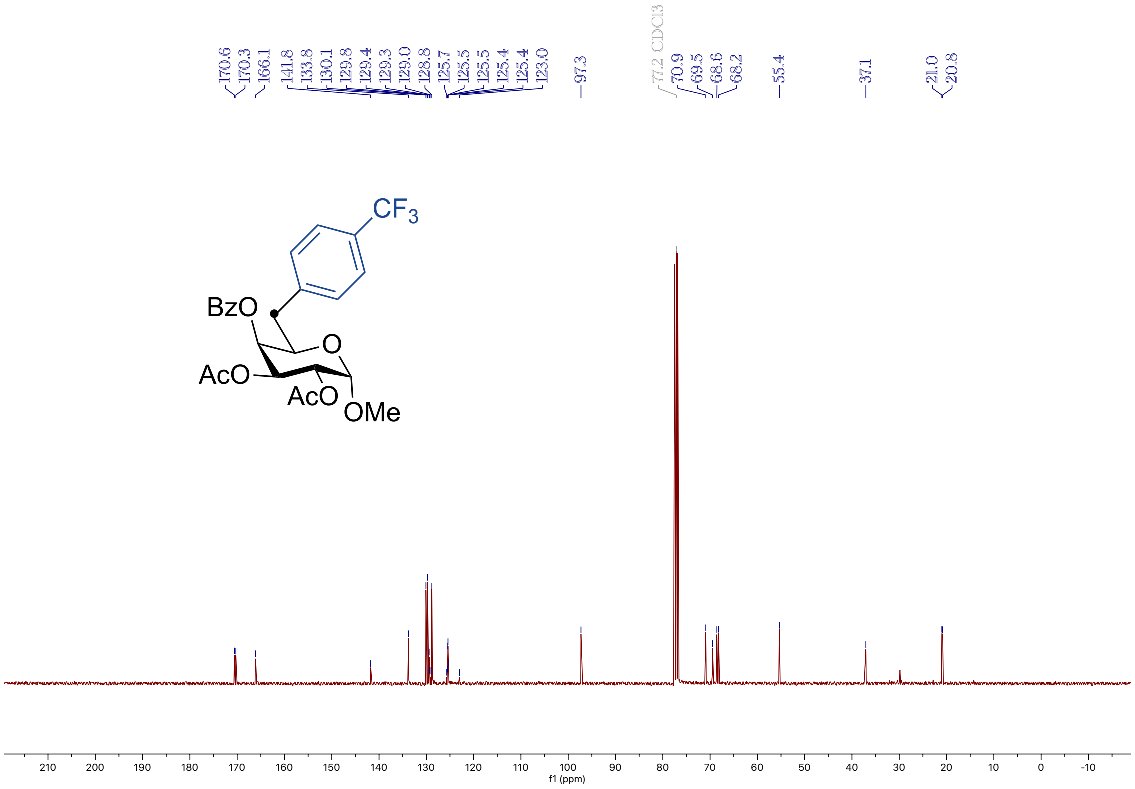

^{13}C NMR (101 MHz, CDCl$_3$) of 5ze

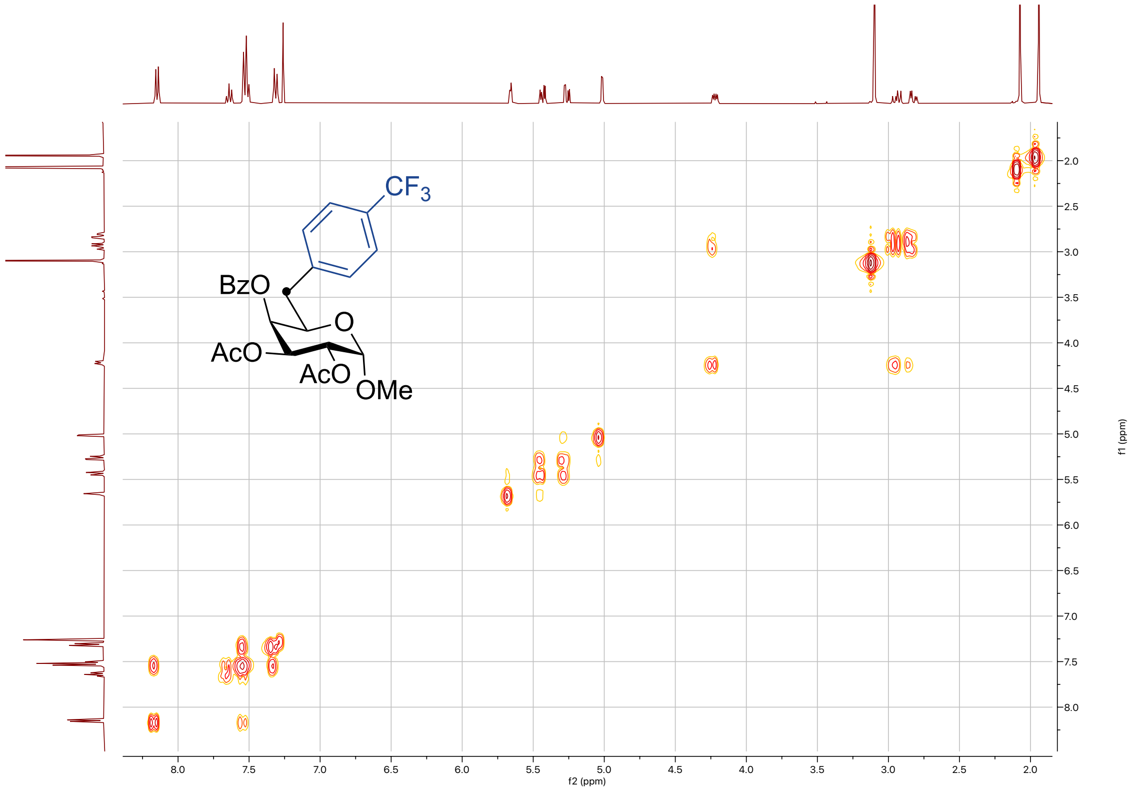

^{1}H-^{1}H COSY of 5ze

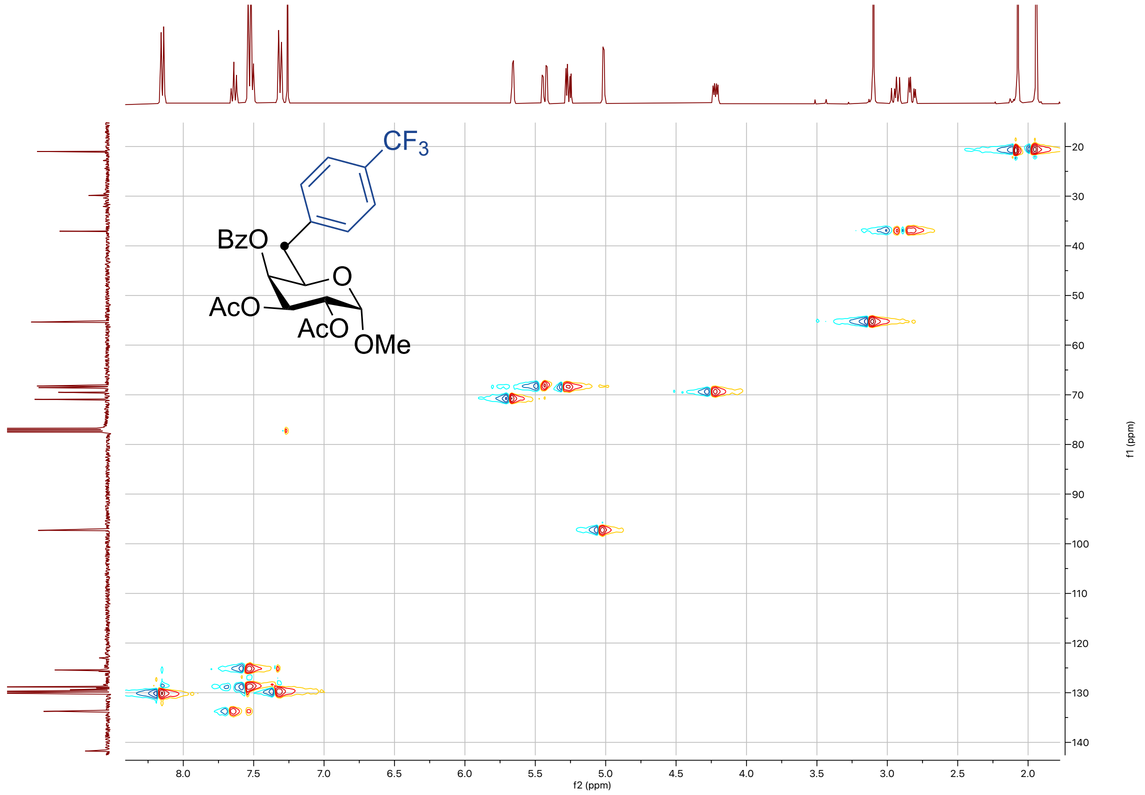

^{1}H-^{13}C HSQC of 5ze

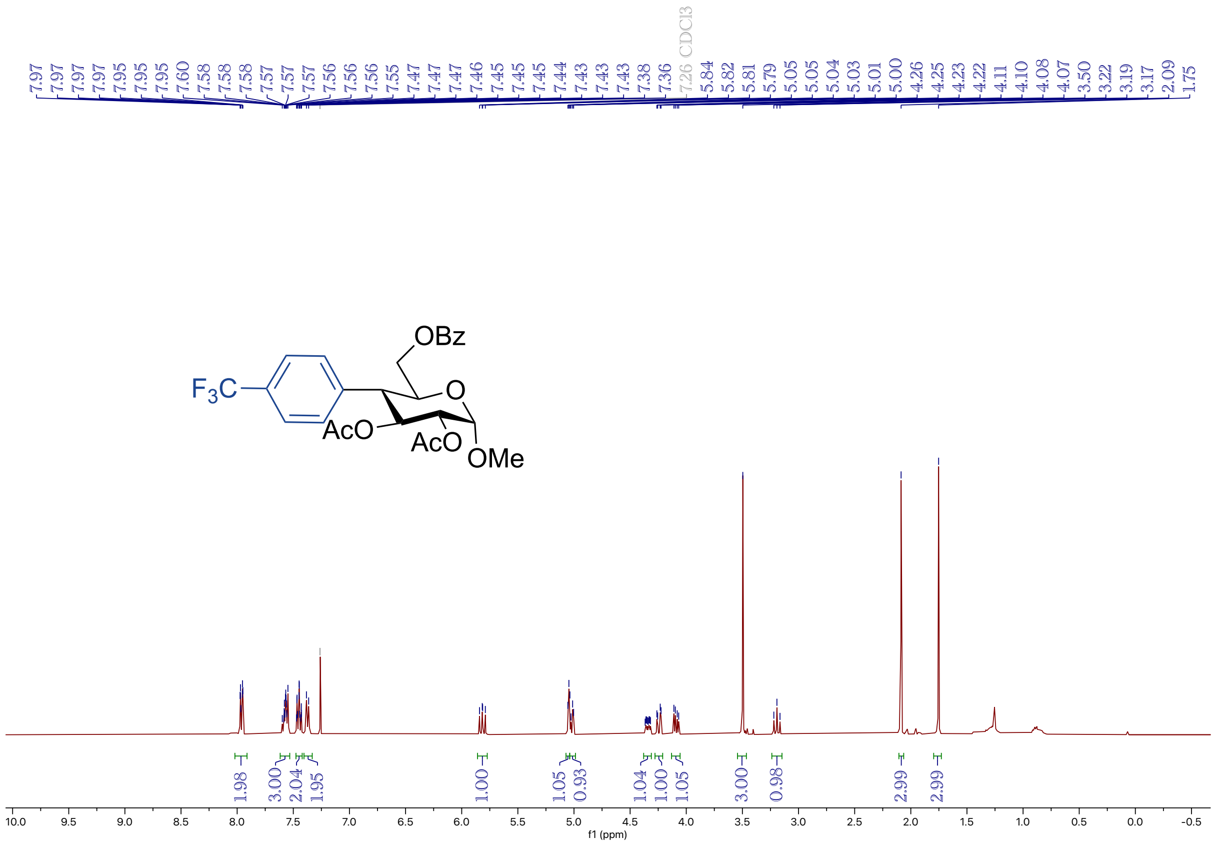

^{1}H NMR (400 MHz, CDCl$_3$) of 5ze´

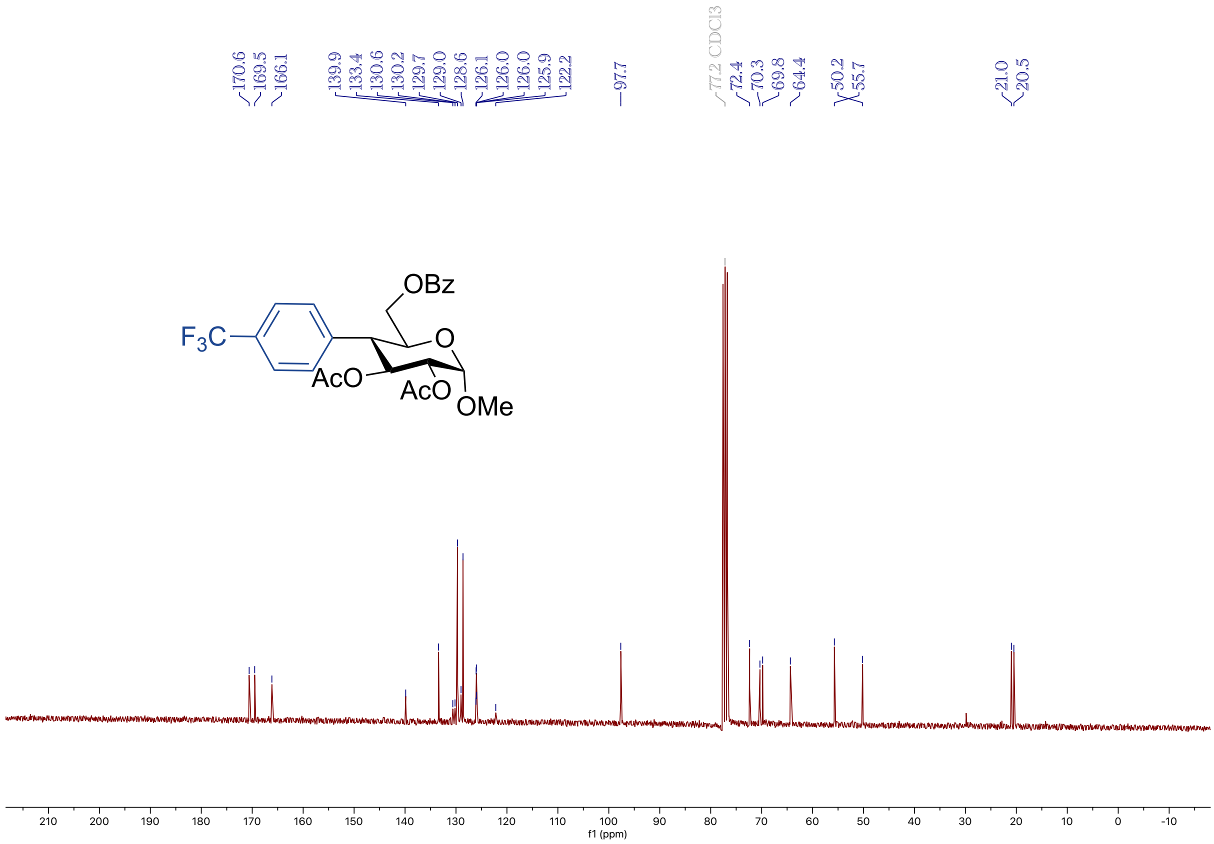

^{13}C NMR (75 MHz, CDCl$_3$) of 5ze´

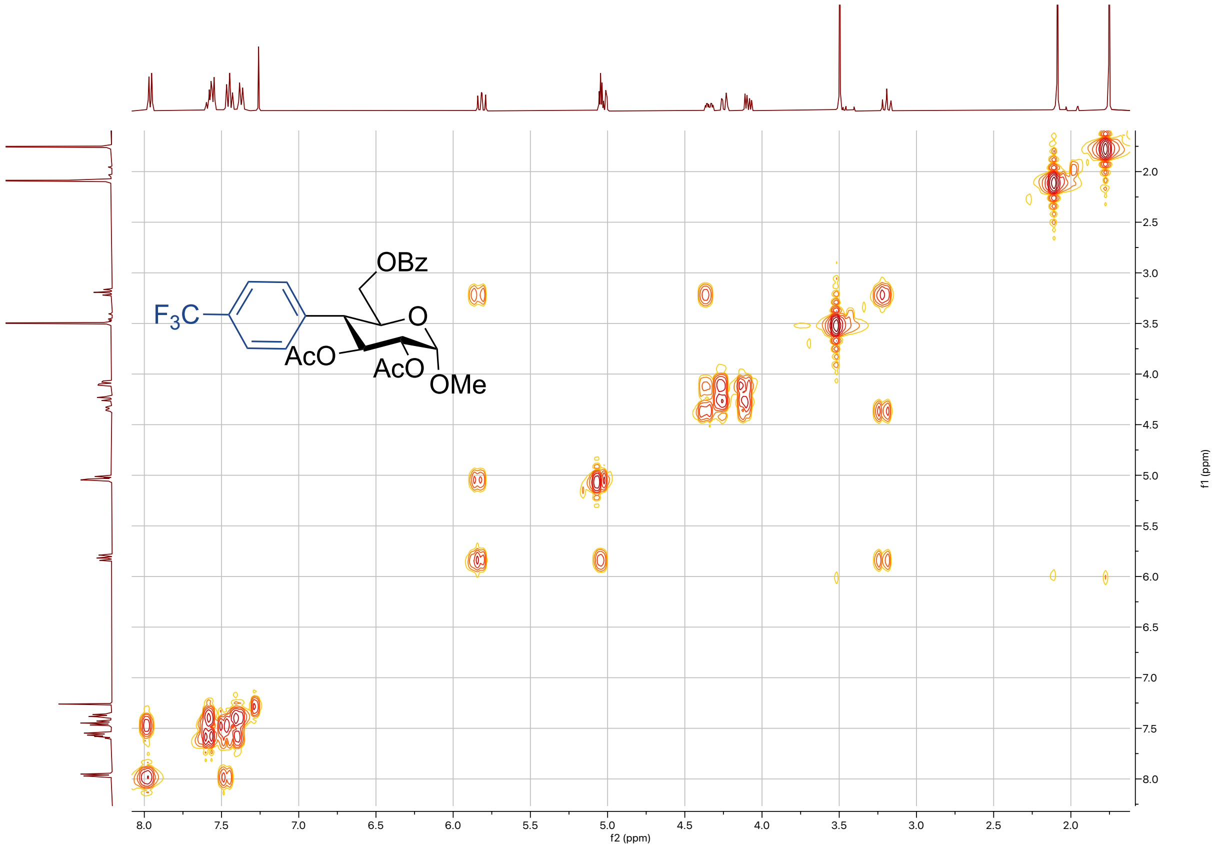

^{1}H-^{1}H COSY of 5ze´

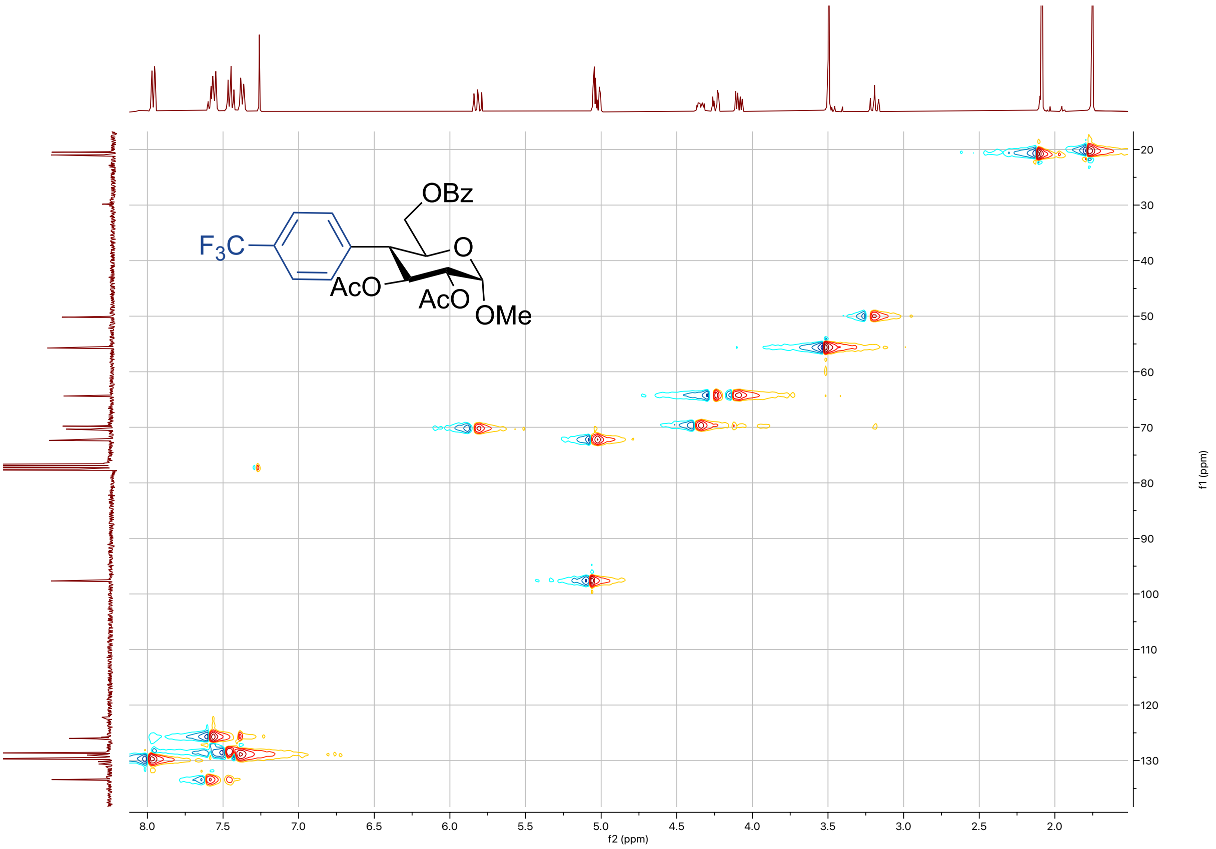

^{1}H-^{13}C HSQC of 5ze´

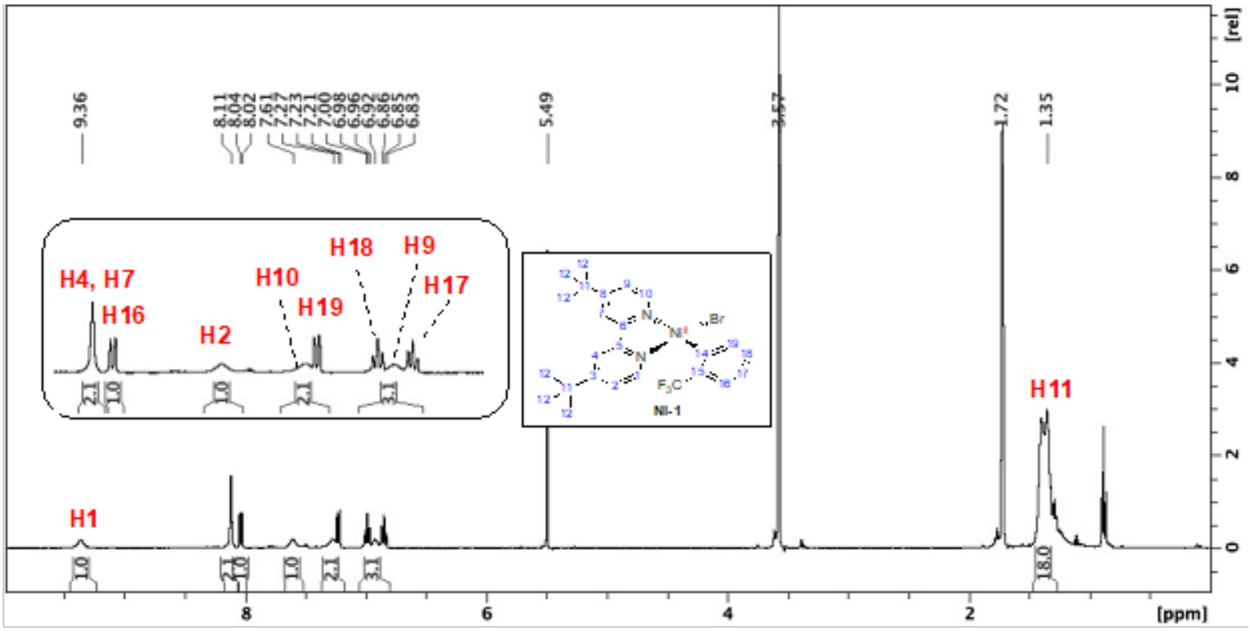

^{1}H NMR (400 MHz, THF-d_8) of Ni-1

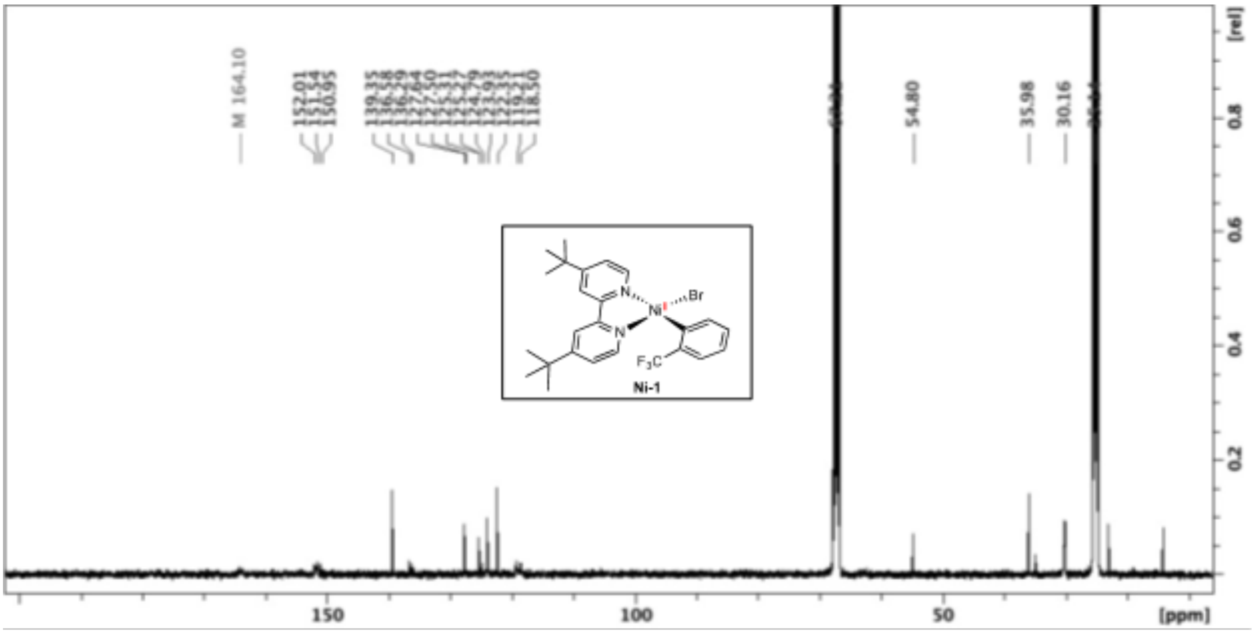

^{13}C NMR (100 MHz, THF-d_8) of Ni-1

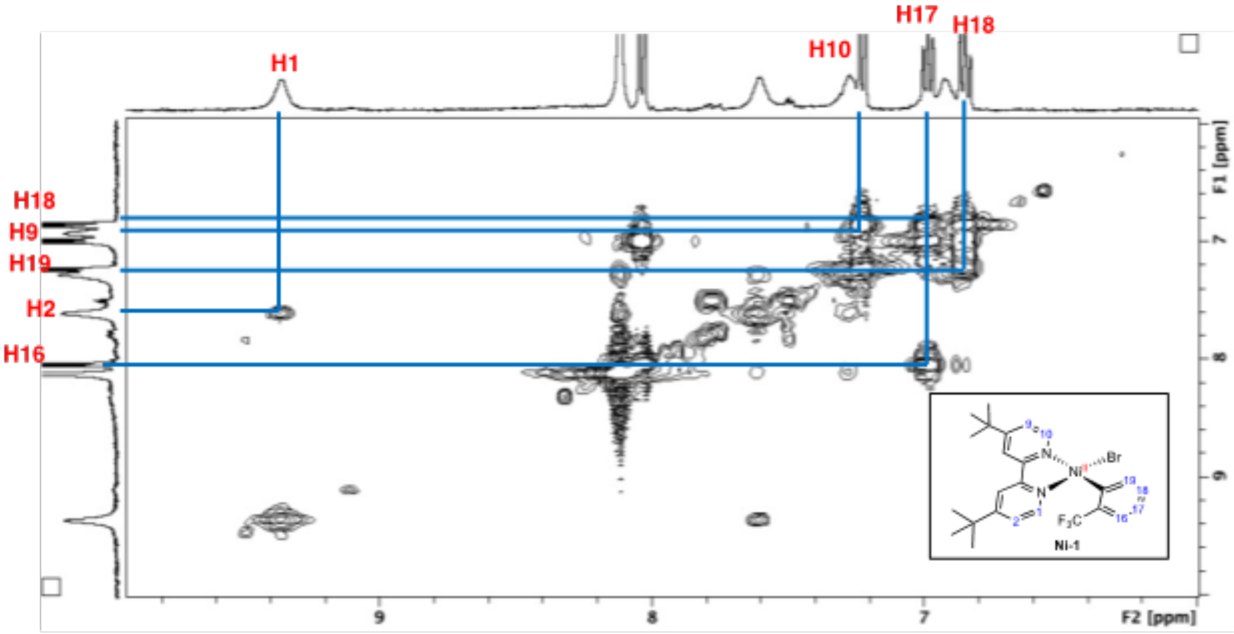

^{1}H-^{1}H COSY NMR (400 MHz, THF-d_8) of Ni-1

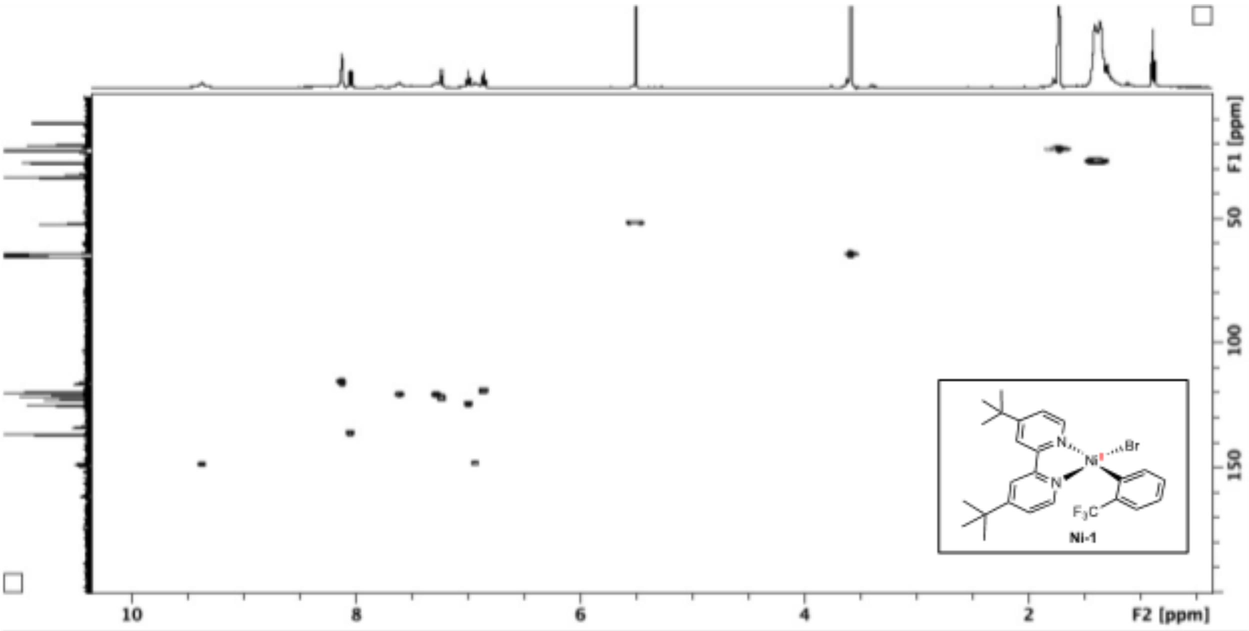

^{1}H-^{13}C HSQC NMR (400 MHz, 100 MHz, THF-d_8) of Ni-1

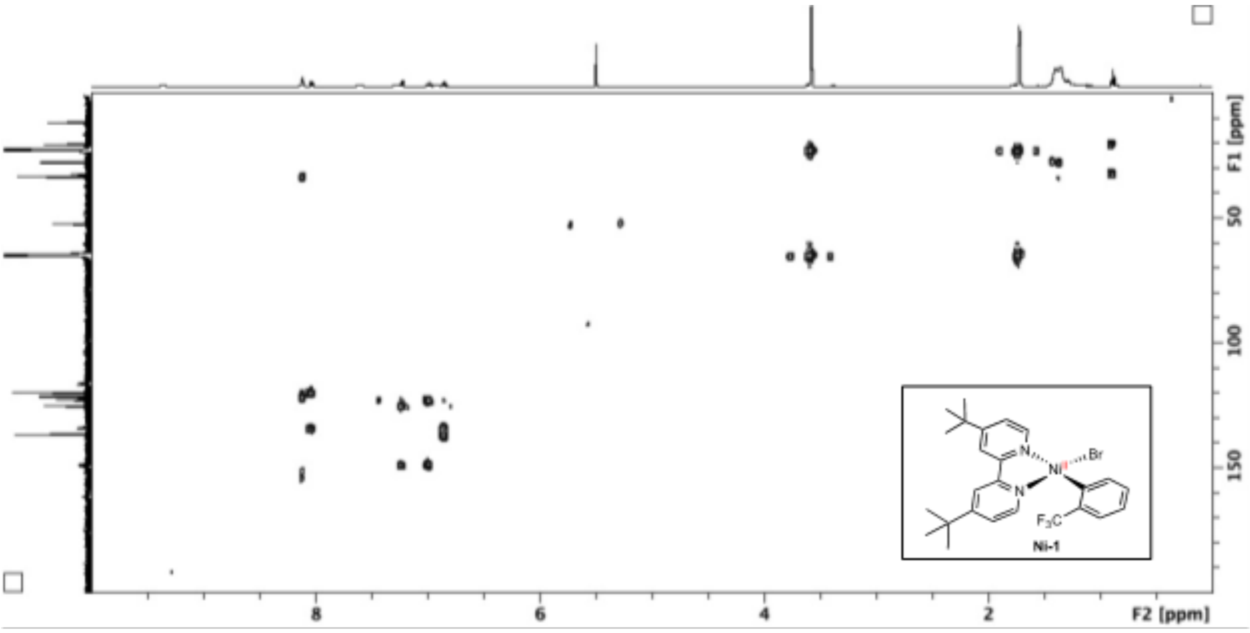

^{1}H-^{13}C HMBC NMR (400 MHz, 100 MHz, THF-d_8) of Ni-1

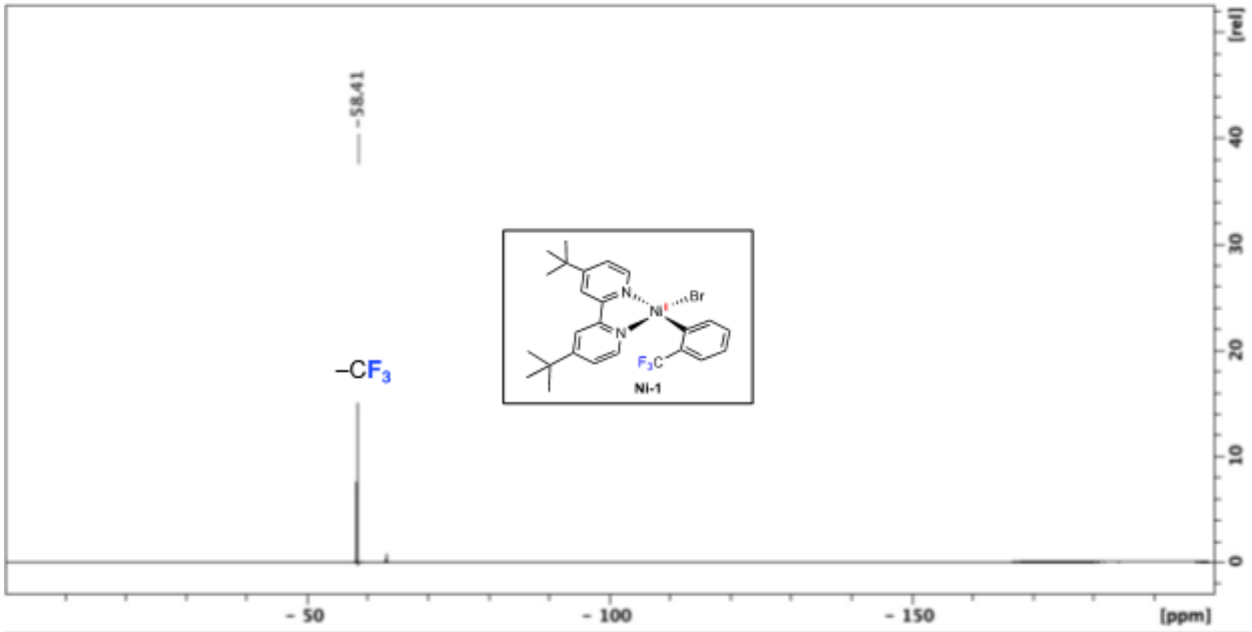

^{19}F NMR (470 MHz, THF-*d*$_8$) of Ni-1
